# Supplementary material for: Effectiveness of interventions designed to mitigate the negative health outcomes of informal caregiving to older adults: an umbrella review of systematic reviews and meta-analyses
Source: BMJ Open. 2023 Apr 21;13(4):e068646. doi: 10.1136/bmjopen-2022-068646 (PMC10124259; doi:10.1136/bmjopen-2022-068646)
Supplement: Supplementary data [file bmjopen-2022-068646supp001.pdf]

Supplementary materials

These supplementary materials are provided by the authors to give readers additional information about the study "Effectiveness of interventions designed to mitigate the negative health outcomes of informal caregiving to older adults: an umbrella review of systematic reviews and meta-analyses".

Kirvalidze M, *et al. BMJ Open* 2023; 13:e068646. doi: 10.1136/bmjopen-2022-068646

Table of Contents

Supplementary Table 1. Amendments made to the published protocol **3**

Supplementary Table 2. Search strategies **4**

Supplementary Table 3. Exclusion decisions based on high overlap among quantitative reviews **8**

Supplementary Table 4. Application of Hendricks et al.'s mega-aggregation framework synthesis method **11**

Supplementary Table 5a. AMSTAR-2 quality assessment tool for quantitative reviews **12**

Supplementary Table 5b. *Ad hoc* quality assessment tool for qualitative reviews **13**

Supplementary Table 6. Repository of all included reviews (*n*=47) **14**

Supplementary Table 7. Summary of findings for interventions involving case management **25**

Supplementary Table 8. Summary of findings for psychosocial, psychoeducational, and skills-building interventions **26**

Supplementary Table 9. Summary of findings for interventions involving respite care **29**

Supplementary Table 10. Summary of findings for interventions involving relaxation, physical activity, or leisure **30**

Supplementary Table 11. Summary of findings for interventions involving mindfulness-based activities **31**

Supplementary Table 12. Coding table for qualitative data **32**

Additional supplementary materials not included within this document:

- Supplementary File 1: Excel spreadsheet for GROOVE citation assessment tool: quantitative reviews
- Supplementary File 2: Excel spreadsheet for GROOVE citation assessment tool: qualitative reviews

Kirvalidze M, *et al. BMJ Open* 2023; 13:e068646. doi: 10.1136/bmjopen-2022-068646

Supplementary Table 1. Amendments made to the published protocol (doi:10.1136/bmjopen-2021-053117)

**Amendment**	**Rationale**
**Research question 4**<br>*Protocol:* (4) How are the proposed interventions experienced by caregivers in terms of their acceptability, feasibility and added value?<br>*Amended:* (4) How are the proposed interventions experienced by caregivers in terms of effectiveness and implementation outcomes?	We amended the fourth research question based on a data-driven approach to encompass a wider range of implementation outcomes. During the qualitative data extraction and categorization exercise, it became clear that data was richer than originally anticipated. We believe that the current formulation better addresses the complexity of intervention experiences, as reported by caregivers.
**Timeframe**<br>*Protocol:* the review is anticipated to be conducted in the period of 1 April 2021– 31 May 2022.	Due to the volume of work required to finalize the umbrella review, we did not fit in the anticipated timeframe. Instead, the final draft of the report was completed in late June of 2022.
**Exclusion criteria**<br>*Amended:* we excluded reviews of critically low quality, as assessed by AMSTAR-2 or the *ad hoc* qualitative tool.<br>*Amended:* we excluded reviews from review pairs with high overlap, as assessed by the Corrected Covered Area methodology.	We introduced two additional exclusion criteria related to the quality of reviews and overlap between reviews. Due to an abundance of reviews of, on average, low quality based on AMSTAR-2, we decided to exclude reviews of critically low quality, thus increasing the certainty of the evidence we subsequently synthesized. Overlap exclusion decisions were made in a data-driven approach, based on the assessment of primary study overlap. We excluded overlapping reviews to avoid double counting and overrepresentation of certain types of studies.
**Data sources and search strategy**<br>*Protocol:* we will use CoCites citation-based search tool to widen the net and retrieve articles that cite eligible systematic reviews. […] Additional searches will be developed for syntheses of effectiveness, implementation or process evaluations published or mandated by official health agencies. We will contact the first and last authors of selected reviews to retrieve grey literature that may otherwise have been missed.<br>*Amended:* we performed a manual search of reference lists of included reviews. The rest of the steps were not undertaken.	Our search strategies led to an overwhelming number of reviews on the topic. Due to an abundance and overrepresentation of available evidence, we decided to be pragmatic and not use any citation search tool. We did not contact experts of official health agencies since the volume of peer-reviewed evidence was already sufficient.
**Change in AMSTAR-2 assessment**<br>Item 10 (*Did the review authors report on the sources of funding for the studies included in the review?*) was not considered as critical.	The team made a consensus-based decision not to include item 10 of AMSTAR-2 as a critical item since this information was exclusively provided by Cochrane reviews. Considering this item as critical would have significantly limited the inclusion of reviews in our umbrella review. In addition, the team considered this criterion too stringent since this is not common practice in non-Cochrane, non-clinical reviews, especially in the field of public health intervention research.
**Change in *ad hoc* quality assessment tool for qualitative reviews**	The team decided to exclude critically low-quality reviews from the qualitative batch as well. Therefore, based on internal consensus, items 3,4,7,10 of the *ad hoc* quality assessment tool were designated as critical, and reviews with more than two critical flaws were excluded.
**Qualitative synthesis methods**<br>*Protocol:* Evidence from syntheses of qualitative research will also be presented in tabular format. Results will be synthesized and illustrated by using verbatim replications from the source review where appropriate. Findings will also be described and interpreted in the research team's own words. Caregivers' experiences and views on barriers to and facilitators of interventions will be identified in terms of acceptability, feasibility and added value (research question 4). The diversity of caregivers, care receivers and/or care contexts will be considered as far as possible.<br>*Amended:* For qualitative reviews, we followed the 'mega-aggregation framework' synthesis method, which has been described elsewhere. First, the extracted verbatim texts from reviews were coded line-by-line (complete coding) by two team members. Codes were then iteratively and deductively categorized based on the adapted version of van Houtven et al.'s framework. Throughout the review, we refer to verbatim texts extracted from reviews and their corresponding codes as third-order constructs, while primary studies inform second-order constructs, and the communication from participants (i.e., caregivers) is considered as first-order construct. Thus, the themes, categories, and sub-categories that we generated based on third-order constructs (i.e., reviews) are termed as fourth-order constructs. In case our fourth-order codes did not fit fully into the framework, they were inductively categorized into new themes. Codes referring to care receivers or staff opinions were removed. A third investigator checked the outcome of the categorization independently, and several consensus meetings were held to resolve disagreements and finalize the synthesis of qualitative materials.	In addition to amending research question 4, methods for synthesizing qualitative data were further developed. Instead of simply providing a narrative summary with verbatim quotes, we undertook a mega-aggregation approach, complemented by "best-fit" framework synthesis. We believe that these methods are better suited for synthesizing review-level qualitative data.

Supplementary Table 2. Search strategies

Databases:
1. Medline (via Ovid)
2. Web of Science Core Collection (via Clarivate)
3. PsycInfo (via Ovid)
4. Cinahl (via EBSCO)

Total number of hits: before deduplication n=11,605 / after deduplication n=6213

1. Search strategy and results in Medline

Interface: Ovid MEDLINE(R) and Epub Ahead of Print, In-Process & Other Non-Indexed Citations and Daily

Date of Search: 26 March 2021

Number of hits: 3,158

Field labels

- exp/ = exploded MeSH term
- / = non exploded MeSH term
- .ti,ab,kf. = title, abstract and author keywords
- adjx = within x words, regardless of order
- * = truncation of word for alternate endings

Database(s): **Ovid MEDLINE(R) and Epub Ahead of Print, In-Process, In-Data-Review & Other Non-Indexed Citations and Daily** 1946 to March 26, 2021

**#**	**Searches**	**Results**
1	exp Aged/	3219373
2	exp Aging/	250613
3	Frailty/	3780
4	(advanced age or aged or ageing or aging or elder* or frail* or geriatr* or gerontolog* or late* life or old age or old* adult* or old* client* or old* individual* or old* man or old* men or old* patient* or old* people or old* person* or old* population* or old* woman or old* women or oldest old or retired or senior*).ti,ab,kf.	1504150
5	Dementia/	53069
6	Alzheimer Disease/	97714
7	(dementia* or alzheimer*).ti,ab,kf.	230766
8	exp Neoplasms/	3433491
9	(neoplasm* or cancer*).ti,ab,kf.	2107334
10	exp Stroke/	141822
11	stroke.ti,ab,kf.	258717
12	Parkinson disease/	68962
13	parkinson*.ti,ab,kf.	124358
14	Multimorbidity/	1267
15	multimorbid*.ti,ab,kf.	5841
16	exp Heart failure/	126175
17	(heart failure or cardiac failure).ti,ab,kf.	193402
18	or/1-17	7707943
19	Caregivers/	38935
20	(caregiv* or care giv* or caretak* or care tak* or carer*).ti,ab,kf.	98680
21	((family or informal or unpaid) adj3 (care or caring)).ti,ab,kf.	17170
22	or/19-21	118701
23	(meta analysis or systematic review).pt.	214329
24	review.ti.	524072
25	systematic* review*.ab,kf.	154180
26	(meta analy* or metaanaly* or meta stud* or meta interpretation* or meta ethnograph* or meta summar* or meta synthes* or meta narrative* or mixed research synthes*).ti,ab,kf.	200564
27	((concept analy* or grounded theory) and review*).ti,ab,kf.	1518
28	or/23-27	699974
29	18 and 22 and 28	3304
30	(english or swedish or spanish or french or italian or german).lg.	29905965
31	29 and 30	3269
32	limit 31 to yr="2000 -Current"	3178
33	limit 32 to (comment or congress or editorial or letter)	20
34	32 not 33	3158

Kirvalidze M, *et al. BMJ Open* 2023; 13:e068646. doi: 10.1136/bmjopen-2022-068646

2. Search strategy and results in Web of Science Core Collection

Interface: Clarivate Analytics

Date of Search: 26 March 2021

Number of hits: 3,991

Field labels

- TS/Topic = title, abstract, author keywords and Keywords Plus
- NEAR/x = within x words, regardless of order
- * = truncation of word for alternate endings

**# 15**	3,991	**#11 AND #6 AND #3**<br>**Refined by: [excluding] DOCUMENT TYPES: (MEETING ABSTRACT OR BOOK REVIEW OR PROCEEDINGS PAPER OR EDITORIAL MATERIAL OR LETTER) AND PUBLICATION YEARS: (2021 OR 2013 OR 2005 OR 2020 OR 2012 OR 2004 OR 2019 OR 2011 OR 2003 OR 2018 OR 2010 OR 2002 OR 2017 OR 2009 OR 2001 OR 2016 OR 2008 OR 2000 OR 2015 OR 2007 OR 2014 OR 2006) AND LANGUAGES: (ENGLISH OR GERMAN OR SPANISH OR ITALIAN OR FRENCH OR SWEDISH)**<br>***Indexes=SCI-EXPANDED, SSCI, A&HCI, CPCI-S, CPCI-SSH, ESCI Timespan=All years***
**# 14**	4,007	**#11 AND #6 AND #3**<br>**Refined by: [excluding] DOCUMENT TYPES: (MEETING ABSTRACT OR BOOK REVIEW OR PROCEEDINGS PAPER OR EDITORIAL MATERIAL OR LETTER) AND PUBLICATION YEARS: (2021 OR 2013 OR 2005 OR 2020 OR 2012 OR 2004 OR 2019 OR 2011 OR 2003 OR 2018 OR 2010 OR 2002 OR 2017 OR 2009 OR 2001 OR 2016 OR 2008 OR 2000 OR 2015 OR 2007 OR 2014 OR 2006)**<br>***Indexes=SCI-EXPANDED, SSCI, A&HCI, CPCI-S, CPCI-SSH, ESCI Timespan=All years***
**# 13**	4,075	**#11 AND #6 AND #3**<br>**Refined by: [excluding] DOCUMENT TYPES: (MEETING ABSTRACT OR BOOK REVIEW OR PROCEEDINGS PAPER OR EDITORIAL MATERIAL OR LETTER)**<br>***Indexes=SCI-EXPANDED, SSCI, A&HCI, CPCI-S, CPCI-SSH, ESCI Timespan=All years***
**# 12**	4,262	**#11 AND #6 AND #3**<br>***Indexes=SCI-EXPANDED, SSCI, A&HCI, CPCI-S, CPCI-SSH, ESCI Timespan=All years***
**# 11**	1,062,768	**#10 OR #9 OR #8 OR #7**<br>***Indexes=SCI-EXPANDED, SSCI, A&HCI, CPCI-S, CPCI-SSH, ESCI Timespan=All years***
**# 10**	2,084	**TS=(("concept analy*" or "grounded theory") and review*)**<br>***Indexes=SCI-EXPANDED, SSCI, A&HCI, CPCI-S, CPCI-SSH, ESCI Timespan=All years***
**# 9**	393,878	**TS=("meta analy*" or metaanaly* or "meta stud*" or "meta interpretation*" or "meta ethnograph*" or "meta summar*" or "meta synthes*" or "meta narrative*" or "mixed research synthes*")**<br>***Indexes=SCI-EXPANDED, SSCI, A&HCI, CPCI-S, CPCI-SSH, ESCI Timespan=All years***
**# 8**	227,798	**TS="systematic* review*"**<br>***Indexes=SCI-EXPANDED, SSCI, A&HCI, CPCI-S, CPCI-SSH, ESCI Timespan=All years***
**# 7**	732,777	**TI=review**<br>***Indexes=SCI-EXPANDED, SSCI, A&HCI, CPCI-S, CPCI-SSH, ESCI Timespan=All years***
**# 6**	131,791	**#5 OR #4**<br>***Indexes=SCI-EXPANDED, SSCI, A&HCI, CPCI-S, CPCI-SSH, ESCI Timespan=All years***
**# 5**	23,282	**TS=(("family" or "informal" or "unpaid") NEAR/3 ("care" or "caring"))**<br>***Indexes=SCI-EXPANDED, SSCI, A&HCI, CPCI-S, CPCI-SSH, ESCI Timespan=All years***
**# 4**	115,945	**TS=(caregiv* or "care giv*" or caretak* or "care tak*" or carer*)**<br>***Indexes=SCI-EXPANDED, SSCI, A&HCI, CPCI-S, CPCI-SSH, ESCI Timespan=All years***
**# 3**	5,155,246	**#2 OR #1**<br>***Indexes=SCI-EXPANDED, SSCI, A&HCI, CPCI-S, CPCI-SSH, ESCI Timespan=All years***
**# 2**	3,790,895	**TS=(alzheimer* or cancer* or "cardiac failure" or dementia* or "heart failure" or multimorbid* or n eoplasm* or parkinson* or "stroke")**<br>***Indexes=SCI-EXPANDED, SSCI, A&HCI, CPCI-S, CPCI-SSH, ESCI Timespan=All years***
**# 1**	1,658,624	**TS=("advanced age" or "aged" or "ageing" or "aging" or elder* or frail* or geriatr* or gerontolog* o r "late* life" or "old age" or "old* adult*" or "old* client*" or "old* individual*" or "old* man" or " old* men" or "old* patient*" or "old* people" or "old* person*" or "old* population*" or "old* wo man" or "old* women" or "oldest old" or "retired" or senior*)**<br>***Indexes=SCI-EXPANDED, SSCI, A&HCI, CPCI-S, CPCI-SSH, ESCI Timespan=All years***

3. Search strategy and results in PsycInfo

Interface: Ovid

Date of Search: 26 March 2021

Number of hits: 1,933

Field labels

- exp/ = exploded controlled term
- / = non exploded controlled term
- .ti,ab,id. = title, abstract and author keywords
- adjx = within x words, regardless of order
- * = truncation of word for alternate endings

Database(s): **APA PsycInfo** 1806 to March Week 3 2021

#	Searches	Results
1	Older Adulthood/	6645
2	Geriatric patients/	13619
3	exp Aging/	77653
4	(advanced age or aged or ageing or aging or elder* or frail* or geriatr* or gerontolog* or late* life or old age or old* adult* or old* client* or old* individual* or old* man or old* men or old* patient* or old* people or old* person* or old* population* or old* woman or old* women or oldest old or retired or senior*).ti,ab,id.	470458
5	Dementia/ or exp Presenile dementia/	76186
6	(dementia* or alzheimer*).ti,ab,id.	104258
7	exp Neoplasms/	54065
8	(neoplasm* or cancer*).ti,ab,id.	65816
9	Cerebrovascular accidents/	21623
10	stroke.ti,ab,id.	34513
11	exp Parkinson's disease/	25813
12	parkinson*.ti,ab,id.	35346
13	Comorbidity/	34018
14	multimorbid*.ti,ab,id.	1051
15	(heart failure or cardiac failure).ti,ab,id.	4153
16	or/1-15	686012
17	Caregivers/	30033
18	Elder care/	4896
19	(caregiv* or care giv* or caretak* or care tak* or carer*).ti,ab,id.	73654
20	((family or informal or unpaid) adj3 (care or caring)).ti,ab,id.	10822
21	17 or 18 or 19 or 20	85126
22	review.ti.	158155
23	systematic* review*.ab,id.	28501
24	(meta analy* or metaanaly* or meta stud* or meta interpretation* or meta ethnograph* or meta summar* or meta synthes* or meta narrative* or mixed research synthes*).ti,ab,id.	41992
25	((concept analy* or grounded theory) and review*).ab,id.	1439
26	22 or 23 or 24 or 25	196607
27	16 and 21 and 26	2148
28	(english or swedish or spanish or french or italian or german).lg.	4774945
29	27 and 28	2064
30	limit 29 to yr="2000 -Current"	1950
31	(editorial or letter).dt.	67669
32	30 not 31	1933

Kirvalidze M, *et al. BMJ Open* 2023; 13:e068646. doi: 10.1136/bmjopen-2022-068646

4. Search strategy and results in Cinahl

Interface: Ebsco

Date of Search: 26 March 2021

Number of hits: 2,523

Field labels

- MH+ = exploded Cinahl Heading
- MH = non exploded Cinahl Heading
- TI = title
- AB = abstract
- Nx = within x words, regardless of order
- * = truncation of word for alternate endings

#	Query	Results
S31	S29 NOT S30	2,523
S30	PT Commentary or Editorial or Letter or Proceedings	854,425
S29	S17 AND S21 AND S28<br><br>Limiters - Published Date: 20000101-20211231;<br>Language: English, French, German, Italian, Swedish	2,590
S28	S22 OR S23 OR S24 OR S25 OR S26 OR S27	294,864
S27	AB (("concept analy*" or "grounded theory") and review*)	1,376
S26	TI ("meta analy*" or metaanaly* or "meta stud*" or "meta interpretation*" or "meta ethnograph*" or "meta summar*" or "meta synthes*" or "meta narrative*" or "mixed research synthes*") OR AB ("meta analy*" or metaanaly* or "meta stud*" or "meta interpretation*" or "meta ethnograph*" or "meta summar*" or "meta synthes*" or "meta narrative*" or "mixed research synthes*")	80,611
S25	AB "systematic* review*"	67,592
S24	TI review	206,732
S23	PT (meta analysis or meta synthesis)	39,376
S22	PT systematic review	110,054
S21	S18 OR S19 OR S20	100,197
S20	TI (((family or informal or unpaid) N3 (care or caring))) OR AB (((family or informal or unpaid) N3 (care or caring)))	21,928
S19	TI (caregiv* or "care giv*" or caretak* or "care tak*" or carer*) OR AB (caregiv* or "care giv*" or caretak* or "care tak*" or carer*)	74,717
S18	(MH "Caregivers")	36,859
S17	S1 OR S2 OR S3 OR S4 OR S5 OR S6 OR S7 OR S8 OR S9 OR S10 OR S11 OR S12 OR S13 OR S14 OR S15 OR S16	1,828,415
S16	TI ("heart failure" or "cardiac failure") OR AB ("heart failure" or "cardiac failure")	55,501
S15	(MH "Heart Failure+")	43,530
S14	TI multimorbid* OR AB multimorbid*	2,720
S13	(MH "Comorbidity")	64,158
S12	TI parkinson* OR AB parkinson*	27,885
S11	(MH "Parkinson Disease")	22,600
S10	TI stroke OR AB stroke	96,983
S9	(MH "Stroke+")	72,157
S8	TI (neoplasm* or cancer*) OR AB (neoplasm* or cancer*)	424,414
S7	(MH "Neoplasms+")	570,301
S6	TI (dementia* or alzheimer*) OR AB (dementia* or alzheimer*)	77,937
S5	(MH "Dementia") OR (MH "Dementia, Presenile+") OR (MH "Dementia, Senile+")	73,337
S4	TI (("advanced age" or aged or ageing or aging or elder* or frail* or geriatr* or gerontolog* or "late* life" or "old age" or "old* adult*" or "old* client*" or "old* individual*" or "old* man" or "old* men" or "old* patient*" or "old* people" or "old* person*" or "old* population*" or "old* woman" or "old* women" or "oldest old" or retired or senior*)) OR AB (("advanced age" or aged or ageing or aging or elder* or frail* or geriatr* or gerontolog* or "late* life" or "old age" or "old* adult*" or "old* client*" or "old* individual*" or "old* man" or "old* men" or "old* patient*" or "old* people" or "old* person*" or "old* population*" or "old* woman" or "old* women" or "oldest old" or retired or senior*))	452,573
S3	(MH "Frailty Syndrome")	2,612
S2	(MH "Aging+")	56,337
S1	(MH "Aged+")	860,457

Kirvalidze M, *et al. BMJ Open* 2023; 13:e068646. doi: 10.1136/bmjopen-2022-068646

Supplementary Table 3. Exclusion decisions based on high overlap among quantitative reviews*

Overlapping clusters (% of overlap)**	Excluded reviews	Rationale for the exclusion of overlaping reviews
Case management: excluded 2 out of 12 reviews		
Cheng 2020 vs Weinbrecht 2016: 24.8%<br>Lee 2020 vs Weinbrecht 2016: 20.5%	Weinbrecht 2016	Weinbrecht did not discuss findings on case management; we identified the category from the summary of included studies table. Lee and Cheng both discussed case management findings Therefore, we decided to exclude Weinbrecht from this category.
Corvol 2017 vs Reilly 2015: 31.8%<br>Reilly 2015 vs Goeman 2016: 15.4%	Corvol 2017	Corvol and Reilly both focused on case management. Reilly was a Cochrane review and better structured for the purposes of this umbrella review. Therefore, we decided to exclude Corvol from this category. Goeman focused on support worker role, which made the review sufficiently different form others.
Psychosocial interventions: excluded 13 out of 35 reviews		
Fu 2017 vs Gabriel 2020: 22.2%	Fu 2017	Fu and Gabriel both focused on caregivers of people with cancer. The quality of the reviews was similar. We decided to include Gabriel since it is more recent.
Hopwood 2018 vs Sin 2018: 22.1%<br>Hopwood 2018 vs Boots 2014: 38.9%<br>Hopwood 2018 vs Egan 2018: 20%<br>Boots 2014 vs Egan 2018: 20%<br>Egan 2018 vs Zhao 2019: 55.6%<br>Hopwood 2018 vs Zhao 2019: 13.5%<br>Hopwood 2018 vs Leng 2020: 24.3%	Boots 2014<br>Egan 2018<br>Zhao 2019	Hopwood, Sin, Boots, Egan, Zhao and Leng all concerned internet-based interventions. All but Sin focused on dementia caregivers, so Sin was sufficiently different. Leng was recent (2020) and included meta-analysis. Egan and Zhao shared 55.6% of studies and were very similar, the only difference being the publication date. Between Hopwood and Boots, Hopwood was more recent. Between Hopwood and Zhao, Hopwood was more informative since it included 36 studies (Zhao includes 6 studies and 5 of them are also included by Hopwood). From this cluster, we decided to include Hopwood, Sin and Leng.
Egan 2018 vs Gonzalez-Fraile 2021: 22.7%<br>Gonzalez-Fraile 2021 vs Lins 2014: 21.1%<br>Gonzalez-Fraile 2021 vs Zhao 2019: 23.8%<br>Gonzalez-Fraile 2021 vs Leng 2020: 34.5%<br>Gonzalez-Fraile 2021 vs Lucero 2019: 15.8%	Leng 2020<br>Lucero 2019	Gonzalez-Fraile was the most recent (2021) and a Cochrane review. We decided to exclude Egan (already excluded above), Leng, Zhao (already excluded above) and Lucero (similar topic, no meta-analysis). We included Lins because of narrower focus (concerns telephone-based interventions specifically).
Lee 2020 vs Amador-Marin 2017: 25%<br>Lee 2020 vs Weinbrecht 2016: 20.5%<br>Lee 2020 vs Teahan 2020: 17.1%	Amador-Marin 2017<br>Weinbrecht 2016	Lee and Tehan were most recent, each had more than 20 RCTs, and overlap between them was low. Thus, we decided to exclude Amador-Marin and Weinbrecht and instead include Teahan and Lee.
Cheng 2020 vs Weinbrecht 2016: 24.8%<br>Cheng 2020 vs Wiegelmann 2021: 23.8%<br>Cheng 2020 vs Vandepitte 2016: 16.7%<br>Cheng 2020 vs Lins 2014: 7.3%<br>Cheng 2020 vs Zabihi 2020: 5%<br>Cheng 2020 vs Akarsu 2019: 5.1%<br>Cheng 2020 vs Hopkinson 2019: 6.8%<br>Cheng 2020 vs Jensen 2015: 7.1%<br>Cheng 2020 vs Reilly 2015: 6.7%<br>Cheng 2020 vs Teahan 2020: 14.8%	Cheng 2020<br>Vandepitte 2016<br>Weinbrecht 2016	Cheng and Wiegelmann were the largest and most recent reviews. However, the overlap between Cheng and most of the smaller reviews was substantial (9 smaller reviews overlapped moderately or highly with Cheng). Therefore, we decided to keep Wiegelmann instead of Cheng, and included the rest of smaller overlapping reviews. In addition, we decided to exclude Vandepitte from this cluster. Weinbrecht was already excluded in the previous cluster.

Cheng 2020 vs GonzalezFraile 2021: 10.7%<br>Cheng 2020 vs Amador-Marin 2017: 7.8%<br>Cheng 2020 vs Lucero 2019: 5.1%<br>Cheng 2020 vs Piersol 2017: 11.5%		
Egan 2018 vs Guay 2017: 17.6%<br>Egan 2018 vs Wang 2020: 25%<br>Egan 2018 vs Leng 2019: 50%<br>Zhao 2019 vs Leng 2019: 50%<br>Leng 2019 vs Guay 2017: 23.5%<br>Zhao 2019 vs Guay 2017: 28.6%<br>Zhao 2019 vs Wang 2020: 30%<br>Guay 2017 vs Wang 2020: 18.8%<br>Guay 2017 vs Sin 2018: 13%	Guay 2017	Zhao, Egan and Leng were already excluded in previous clusters. Between Guay and Sin, we decided to keep Sin, since it had 78 studies, while Guay included only 12. We included Wang from this cluster.
Lins 2014 vs Lucero 2019: 15.8%	Lucero 2019	We already excluded Lucero in previous clusters. Lins was included.
Hopkinson 2019 vs Vernooij-Dassen 2011: 26.3%	Vernooij-Dassen 2011	Between Hopkinson and Vernooij-Dassen, both had meta-analysis and both were about Cognitive Behavioral Therapy exclusively. We decided to include Hopkinson, since it was more recent (2019 vs 2011) and had more studies.
Jensen 2015 vs Wiegelmann 2021: 25%<br>Teahan 2020 vs Wiegelmann 2021: 24.5%<br>Weinbrecht 2016 vs Wiegelmann 2021: 18.4%<br>Jensen 2015 vs Teahan 2020: 14.3%	Jensen 2015<br>Weinbrecht 2016	We already excluded Weinbrecht in previous clusters. Between Jensen and Teahan, Teahan was more recent. Thus, we additionally excluded Jensen from this cluster.
Legg 2011 vs Minshall 2019: 19%	Legg 2011	Between Legg and Minshall, we decided to include Minshall since it was more recent (2019 vs 2011) and included more studies (31 vs 8).
Education and Skills building: excluded 12 out of 32 reviews		
Legg 2011 vs Smith 2019: 22.2%<br>Pucciarelli 2020 vs Smith 2019: 22.2%<br>Legg 2011 vs Minshall 2019: 19%	Legg 2011	Smith had a wider focus, while Legg and Pucciarelli focused on caregivers of patients with stroke. Since the latter two did not have high enough overlap, we decided to include all three reviews. Between Legg and Minshall, we decided to include Minshall only since it was more recent (2019 vs 2011) and included more studies (31 vs 8).
Fu 2017 vs Gabriel 2020: 22.2%	Fu 2017	Fu and Gabriel both focused on caregivers of people with cancer. The quality of the reviews was similar. We decided to include Gabriel since it was more recent.
Hopwood 2018 vs Sin 2018: 22.1%<br>Hopwood 2018 vs Boots 2014: 38.9%<br>Hopwood 2018 vs Egan 2018: 20%<br>Boots 2014 vs Egan 2018: 20%<br>Egan 2018 vs Zhao 2019: 55.6%<br>Hopwood 2018 vs Zhao 2019: 13.5%<br>Hopwood 2018 vs Leng 2020: 24.3%	Boots 2014<br>Egan 2018<br>Zhao 2019	Hopwood, Sin, Boots, Egan, Zhao and Leng all concerned internet-based interventions. All but Sin focused on dementia caregivers, so Sin was sufficiently different. Leng was recent (2020) and included meta-analysis. Egan and Zhao shared 55.6% of studies and were very similar, the only difference being the publication date. Between Hopwood and Boots, Hopwood was more recent. Between Hopwood and Zhao, Hopwood was more informative since it included 36 studies (Zhao includes 6 studies and 5 of them were also included by Hopwood). From this cluster, we decided to include Hopwood, Sin and Leng.
Egan 2018 vs Gonzalez-Fraile 2021: 22.7%<br>Gonzalez-Fraile 2021 vs Lins 2014: 21.1%<br>Gonzalez-Fraile 2021 vs Zhao 2019: 23.8%	Leng 2020<br>Lucero 2019	Gonzalez-Fraile was the most recent (2021) and a Cochrane review. We decided to exclude Egan (already excluded above), Leng, Zhao (already excluded above) and Lucero (similar topic, no meta-

Gonzalez-Fraile 2021 vs Leng 2020: 34.5%<br>Gonzalez-Fraile 2021 vs Lucero 2019: 15.8%		analysis). We included Lins because of a narrower focus (concerns telephone-based interventions specifically).
Lee 2020 vs Amador-Marin 2017: 25%<br>Lee 2020 vs Weinbrecht 2016: 20.5%<br>Lee 2020 vs Teahan 2020: 17.1%	Amador-Marin 2017<br>Weinbrecht 2016	Lee and Tehan were most recent, each had more than 20 RCTs, and overlap between them was low. Thus, we decided to exclude Amador-Marin and Weinbrecht and instead include Teahan and Lee.
Cheng 2020 vs Weinbrecht 2016: 24.8%<br>Cheng 2020 vs Wiegelmann 2021: 23.8%<br>Cheng 2020 vs Vandepitte 2016: 16.7%<br>Cheng 2020 vs Lins 2014: 7.3%<br>Cheng 2020 vs Zabihi 2020: 5%<br>Cheng 2020 vs Akarsu 2019: 5.1%<br>Cheng 2020 vs Hopkinson 2019: 6.8%<br>Cheng 2020 vs Jensen 2015: 7.1%<br>Cheng 2020 vs Reilly 2015: 6.7%<br>Cheng 2020 vs Teahan 2020: 14.8%<br>Cheng 2020 vs GonzalezFraile 2021: 10.7%<br>Cheng 2020 vs Amador-Marin 2017: 7.8%<br>Cheng 2020 vs Lucero 2019: 5.1%<br>Cheng 2020 vs Piersol 2017: 11.5%	Cheng 2020<br>Vandepitte 2016	Cheng and Wiegelmann were the largest and most recent reviews. However, the overlap between Cheng and most of the smaller reviews was substantial (9 smaller reviews overlapped moderately or highly with Cheng). Therefore, we decided to keep Wiegelmann instead of Cheng, and included the rest of smaller overlapping reviews. In addition, we decided to exclude Vandepitte from this cluster. Therefore, we decided to exclude Weinbrecht (already excluded above) and Vandepitte (already excluded above) from this cluster.
Egan 2018 vs Guay 2017: 17.6%<br>Egan 2018 vs Leng 2019: 50%<br>Zhao 2019 vs Leng 2019: 50%<br>Leng 2019 vs Guay 2017: 23.5%<br>Zhao 2019 vs Guay 2017: 28.6%<br>Guay 2017 vs Sin 2018: 13%	Guay 2017	Zhao, Egan and Leng were already excluded in previous clusters. Between Guay and Sin, we decided to keep Sin, since it had 78 studies, while Guay included only 12.
Lins 2014 vs Lucero 2019: 15.8%		We already excluded Lucero in previous clusters. Lins was included.
Jensen 2015 vs Wiegelmann 2021: 25%<br>Teahan 2020 vs Wiegelmann 2021: 24.5%<br>Weinbrecht 2016 vs Wiegelmann 2021: 18.4%<br>Jensen 2015 vs Teahan 2020: 14.3%	Jensen 2015	We already excluded Weinbrecht in previous clusters. Between Jensen and Teahan, Teahan was more recent. Thus, we additionally excluded Jensen from this cluster.
Respite care: excluded 1 out of 6 reviews		
Shaw 2009 vs Mason 2007: 21.7%	Mason 2007	Shaw was more recent and included more studies (104 vs 22). Thus, we decided to exclude Mason from this cluster.
Relaxation, physical activity, and leisure: excluded 1 out of 7 reviews		
Cheng 2020 vs Wiegelmann 2021: 23.8%	Wiegelmann 2021	Cheng and Wiegelmann were the largest and most recent reviews. Cheng included more studies on the topic, so we decided to exclude Wiegelmann from this cluster.
Mindfulness: excluded 1 out of 6 reviews		
Liu 2018 vs Collins 2019: 30.8%<br>Liu 2018 vs Shim 2020: 18.2%	Collins 2019	All three reviews concerned mindfulness interventions for caregivers of people with dementia. Liu was a Cochrane review (but only included 5 studies), Shim included 20 studies. We decided to exclude Collins from this cluster.

*The number of qualitative reviews in each category was low and overlapping clusters were limited. Due to the scarcity of qualitative evidence and low overlap, we decided not to exclude any qualitative review based on this approach. For full information about overlap among qualitative reviews, see the Citation Matrix in Supplementary File 2.

**We assessed clusters of reviews with very high (33 pairs), high (40 pairs) or moderate overlap (only if one is almost fully included in the other); see the Citation Matrix in Supplementary File 1 for the overlap among all included reviews after quality assessment.

Supplementary Table 4. Application of Hendricks et al.'s mega-aggregation framework synthesis method[1]

**Step 1: Identify a clearly defined review question and objectives**		
This umbrella review aims to explore the effectiveness of interventions targeting informal caregivers' physical and mental health. The fourth objective, which is the one mainly feeding on qualitative evidence, is as follows: how are the proposed interventions experienced by caregivers in terms of effectiveness and implementation outcomes?		
**Step 2: Identify a theoretical framework or model**		
Adapted version of Van Houtven et al.'s framework[2,3]	Intervention, Context of caregiving relationship, Caregiver, Care receiver, Caregiver knowledge, attitude and behaviors, Caregivers' experience of the intervention, Caregiver outcomes	The adapted framework describes the different determinants that affect the effectives of support interventions in addressing health outcomes of caregivers, and how they interact by modulating the other determinants and components
**Step 3: Decide on criteria for considering reviews for inclusion**		
Inclusion and exclusion criteria used to select reviews are presented in Table 1 of the published umbrella review protocol[3]		
**Step 4: Conduct searching and screening**		
The following databases were searched from 1 January 2000 to 26 March 2021: Medline, CINAHL, PsycINFO and Web of Science. In addition, we performed a manual search of the reference lists of included reviews	Screening was done independently and in duplicate by MK and ACL; MK and LM; MK and LS; MK and LD	
**Step 5: Conduct quality appraisal of the included studies**		
An *ad hoc* tool was used, developed and piloted by review authors	Quality appraisal was done independently and induplicate by all co-authors	Reviews of critically low quality were excluded (i.e., those that had 2 or more critical items missing or not reported); 18 reviews contributed to the next step
**Step 6: Data extraction and categorization**		
AA, ACL and MK read all reviews	AA and MK extracted the verbatims and each verified the extraction of the other co-author	Construction of fourth-order constructs (codes, categories, and aggregations) was done in consensus by AA and MK, and then reviewed by third co-author ACL. In the case of codes and categories not fitting the framework, they were inductively abstracted and new themes were generated and added to the framework. All three co-authors then reviewed and discussed the process until consensus was reached by all three co-authors. The results of the categorization and aggregation was then shared with the other co-authors and consensus was reached
**Step 7: Present and synthesize the findings**		
Two themes were constructed (intervention outcomes and implementation outcomes) using 200 codes, with further groupings of codes in categories and sub-categories	Evidence gaps and lines of actions were identified following convergence of the qualitative and quantitative findings, and were later presented in the discussion	
**Step 8: Transparent reporting**		
PRIOR[4] reporting guideline were used as far as possible. The protocol was published (doi:10.1136/bmjopen-2021-053117) and the categorization and aggregation of the third-order constructs to develop the fourth-order constructs was shown in supplementary files. Additionally, differences between the protocol and the manuscript were reported in supplementary files.		

References

1. Hendricks L, Eshun-Wilson I, Rohwer A. A mega-aggregation framework synthesis of the barriers and facilitators to linkage, adherence to ART and retention in care among people living with HIV. *Syst Rev*. 2021;10(1):1-28. doi:10.1186/s13643-021-01582-z
2. Van Houtven CH, Voils CI, Weinberger M. An organizing framework for informal caregiver interventions: Detailing caregiving activities and caregiver and care recipient outcomes to optimize evaluation efforts. *BMC Geriatr*. 2011;11. doi:10.1186/1471-2318-11-77
3. Calderón-Larrañaga A, Kirvalidze M, Dahlberg L, Sacco LB, Morin L. Effectiveness of interventions to address the negative health outcomes of informal caregiving to older adults: Protocol for an umbrella review. *BMJ Open*. 2021;11(11). doi:10.1136/bmjopen-2021-053117
4. Gates M, Gates A, Pieper D, et al. Reporting guideline for overviews of reviews of healthcare interventions: The Preferred Reporting Items for Overviews of Reviews (PRIOR) statement. *MetaArXiv*. Published online 2022. doi:10.31222/osf.io/82wau

Supplementary Table 5a. AMSTAR-2 quality assessment tool for quantitative reviews

High	Moderate	Low	Critically low (excluded)	
Maayan, 2014	Cheng, 2020	Amador-Marin, 2017	AlDaken, 2018	Van'tLeven, 2013
Forster, 2012	Minshall, 2019	Egan, 2018	Bergstrom, 2018	Zhu, 2021
Liu, 2018	Smith, 2019	Gabriel, 2020	Brodaty, 2003	Wang, 2017
Reilly, 2015	Gonzalez-Fraile, 2021	Heckel, 2019	Bustillo, 2018	Peacock, 2003
Vernooij-Dassen, 2011	Wang, 2020	Hopwood, 2018	Cheng, 2014	Northouse, 2010
Goeman, 2016	Akarsu, 2019	Irons, 2020	Dam, 2016	Dharmawardene, 2016
	Corry, 2019	Leng, 2020	EluvathingolJose, 2013	Brereton, 2007
	Legg, 2011	Shaw, 2009	Grandi, 2019	Liu, 2017
	Lins, 2014	Shim, 2020	Hopkinson, 2012	Thompson, 2007
	Pucciarelli, 2020	Teahan, 2020	Irani, 2020	Eldred, 2008
	Sin, 2018	Zhao, 2019	Kedia, 2020	Regan, 2012
	Lucero, 2019	Collins, 2019	Kor, 2018	Heynsbergh. 2018
	Treanor, 2019	Guay, 2017	Lee, 2007	Abrahams, 2018
		Lee, 2020	Li, 2020	Li, 2016
		Mason, 2007	Li, 2014	Jackson, 2016
		Vandepitte, 2016	Luo, 2020	You, 2012
		Waldron, 2013	Ruggiano, 2018	Lambert, 2016
		Weinbrecht, 2016	Selwood, 2007	Domingues, 2018
		Zabihi, 2020	Sherifali, 2018	Miles, 2020
		Boots, 2014	Smith, 2014	Scott, 2016
		Corvol, 2017	Spencer, 2019	Hurley, 2014
		Cuthbert, 2017	ZabaleguiYarnoz, 2008	Etxeberria, 2020
		Doyle, 2020	Bourne, 2020	Deeken, 2019
		Fu, 2017	Bunn, 2016	Hempel, 2008
		Hopkinson, 2019	Byeon, 2020	Fange, 2020
		Jensen, 2015	Caress, 2009	Lally, 2016
		Maffioletti, 2019	Cassidy, 2021	Ussher, 2009
		Parkinson, 2019	Epps, 2021	Stahl, 2016
		Bennett, 2019	Frias, 2020	Mantovan, 2010
		Wiegelmann, 2021	Kaddour, 2018	Panzeri, 2019
		Greenwood, 2016	Kaltenbaugh, 2015	Poon, 2019
		Piersol, 2017	Kwon, 2017	Pritchard, 2020
			Laver, 2017	Xu, 2020
			Leung, 2017	Ahn, 2020
			Lorca-Cabrera, 2020	Chien, 2011
			Mason, 2007	Cooper, 2007
			McKechnie, 2014	Corbett, 2012
			Pinquart, 2006	Du Preez, 2018
			Pleasant, 2020	Elvish, 2013
			Pusey, 2001	Kleine, 2019
			Smits, 2007	Lee, 2020
			Vandepitte, 2016	Mason, 2008
			Verreault, 2021	MoleroJurado, 2020
			Waller, 2017	Moon, 2013
			Walter, 2020	Moore, 2020
			Wu, 2019	O'Toole, 2017

The definitions of each quality category, critical items and recommendations on grading are available from the original publication: Shea BJ, Reeves BC, Wells G, et al. AMSTAR 2: A critical appraisal tool for systematic reviews that include randomised or non-randomised studies of healthcare interventions, or both. BMJ. 2017;358:j4008. doi:10.1136/bmj.j4008.

Supplementary Table 5b. ***Ad hoc*** **quality assessment tool* for qualitative reviews**

Qualitative reviews with 2 or more critical flaws**	Qualitative reviews less than 2 critical flaw**
Jackson, 2016	Parkinson, 2019
Sousa, 2016	Sin, 2018
Hempel, 2008	Goeman, 2016
Armstrong, 2019	Li, 2016
Elvish, 2013	Miles, 2020
Moon, 2013	Boots, 2014
Caress, 2009	Du Preez, 2018
EluvathingolJose, 2013	Lins, 2014
Heckel, 2019	Maffioletti, 2019
	Pritchard, 2020
	Bourne, 2020
	Bunn, 2016
	AlDaken, 2018
	Dam, 2016
	Hopwood, 2018
	Irons, 2020
	Shaw, 2009
	Smith, 2014

*The tool can be accessed in the published protocol (doi:10.1136/bmjopen-2021-053117).
**Critical flaws included: transparency and comprehensiveness of the literature search (item #3), using at least two databases for the search (item #4), assessment of methodological quality of primary studies using explicit criteria (item #7), involvement of two or more researchers in data synthesis process (item #11).

Supplementary Table 6. Repository of all included reviews (*n*=47)

Author, year	Countries of primary studies	Study design	Databases searched	End date of literature search	# studies included in the umbrella review	# participants, mean age, % female	Intervention type	Disease of care receiver	Health outcomes of caregiver	Review type and synthesis method	Quantitative, qualitative, or mixed-methods
Akarsu, 2019[1]	US	RCTs	Embase, cinahl, medline, psycinfo, psycarticles, assia, central, sociological abstracts, spp	2015	13	2056 caregivers, mean age 59 (SD=11.36), 86% female	Psychosocial, Education and skills	Dementia	Depression	Systematic review with meta-analysis	Quantitative
Aldaken, 2018[2]	UK, China, Iran	RCTs, incl. some with qualitative interviews	Ebsco, google scholar, science direct	2017	1	NA	Mindfulness	Cancer	NA	Systematic review	Qualitative
Bennett, 2019[3]	US, Germany, Netherlands, UK, Hong Kong, Australia, Brazil	RCTs	Medline, embase, central, cumulative index to nursing and allied health literature, psycinfo, education resources information Centre, otseeker, pedro, clinical trial registries	Feb-2018	15	2063 dyads, mean age 65, 20%-90% of caregivers were spouses	Education and skills	Dementia	Emotional distress, HR-QoL	Systematic review with meta-analysis	Quantitative
Boots, 2014[4]	NA	Any	Pubmed, psycinfo, cinahl, web of science, cochrane library	Jan-2013	4	NA	Psychosocial, Education and skills	Dementia	NA	Systematic review	Qualitative
Bourne, 2020[5]	UK, US, Australia, New Zealand	Qualitative and mixed method	Psycinfo, medline, web of science, assia, google scholar	Mar-2020	10	NA	Relaxation and leisure	Dementia	NA	Systematic review	Qualitative
Bunn, 2016[6]	UK	Any	Medline, cinahl, scopus, nhs evidence, cochrane library, google scholar	Nov-2012	5	NA	Case management, Psychosocial, Education and skills	Dementia	NA	Systematic review	Qualitative

Kirvalidze M, *et al. BMJ Open* 2023; 13:e068646. doi: 10.1136/bmjopen-2022-068646

Author, year	Countries of primary studies	Study design	Databases searched	End date of literature search	# studies included in the umbrella review	# participants, mean age, % female	Intervention type	Disease of care receiver	Health outcomes of caregiver	Review type and synthesis method	Quantitative, qualitative, or mixed-methods
Cheng, 2020[7]	US, Australia, Brazil, Denmark, Norway, UK, Spain, Greece, Finland, India, Netherlands, Germany, Egypt, Japan, Iran, Canada, Taiwan	RCTs, quasi-experimental	Psycinfo, medline, cinahl	Mid-2018	37	NA	Relaxation and leisure, Mindfulness	Dementia	Depression, anxiety, HR-QoL, physical health	Systematic review with meta-analysis	Quantitative
Corry, 2019[8]	NA	RCTs, quasi-experimental	Central, medline, embase, psycinfo, proquest, cinahl	Nov-2018	21	1690 caregivers, age range 19-87, >70.5% female	Psychosocial, Education and skills	Any	HR-QoL, psychological health (depression, anxiety), physical health	Systematic review with meta-analysis	Quantitative
Cuthbert, 2017[9]	NA	RCTs, quasi-experimental	Medline, pubmed, cinahl	Sep-2015	14	12-137 caregivers, age range 41-73.7	Relaxation and leisure	Any	Depression, anxiety, well-being, QoL, physical strengthening, blood pressure, weight	Systematic review	Quantitative
Dam, 2016[10]	NA	Any	Pubmed, psycinfo, cinahl, web of science, cochrane library	May-2015	4	NA	Psychosocial	Dementia	NA	Systematic review	Qualitative
Doyle, 2020[11]	NA	RCTs, quasi-experimental, case-control, and cohort studies	Pubmed, web of science, cinahl plus, cochrane library, ot seeker, psycinfo, scopus	Apr-2017	11	862 caregivers, mean age 67, 69.4% female	Relaxation and leisure	Any	Depression, anxiety, physical health	Systematic review	Quantitative
Du Preez, 2018[12]	NA	Qualitative studies and mixed methods	Pubmed, web of science, medical database (proquest), medline, bmj best practice, scopus, psycinfo, otseeker, cinahl	Nov-2016	10	NA	Respite	Dementia	NA	Integrative review	Qualitative

Author, year	Countries of primary studies	Study design	Databases searched	End date of literature search	# studies included in the umbrella review	# participants, mean age, % female	Intervention type	Disease of care receiver	Health outcomes of caregiver	Review type and synthesis method	Quantitative, qualitative, or mixed-methods
Forster, 2012[13]	US, UK, Australia, Sweden, the Netherlands, Taiwan, Thailand	RCTs	Cochrane library, dare, eed, hta database, medline, embase, cinahl, psycinfo	Jun-2012	21	1290 caregivers	Case management, Psychosocial, Education and skills	Stroke	Depression, HR-QoL	Systematic review	Quantitative
Gabriel, 2020[14]	US, France	RCTs, quasi-experimental	Pubmed, medline, cinahl, psycinfo, web of science, who clinical trials registry, international standard rct registry	2019	12	3390 participants (including caregivers), age range for caregivers 43-61	Psychosocial, Education and skills	Cancer	Psychological/emotional and physical domains of QoL	Systematic review	Quantitative
Goeman, 2016[15]	Hong Kong, Netherlands, US, Italy, Finland, UK, Canada, Australia	Any	Medline, cinahl, psycinfo, google scholar	Dec-2014	36	NA	Case management	Dementia	Depression, HR-QoL, general health	Systematic review	Mixed methods
Gonzalez-Fraile, 2021[16]	US, China, Netherlands, France, Canada, Spain, UK	RCTs	Alois, specialised register of the cochrane dementia and cognitive improvement group	Apr-2020	26	2367 caregivers, median age 63, 72% female	Psychosocial, Education and skills	Dementia	Depression, depressive symptoms, HR-QoL	Systematic review with meta-analysis	Quantitative
Greenwood, 2016[17]	US, Spain	RCTs and quasi-experimental	Medline, embase, cochrane library, psycinfo; cinahl plus, applied social sciences index, abstracts and healthcare management information consortium	Jul-2015	4	447 caregivers, age range 61-72, mostly female	Case management, Psychosocial, Education and skills	Dementia, stroke	Depression, general health	Systematic review	Quantitative
Li, 2016[18]	US, the Netherlands, China, Canada	Any	Cochrane library, pubmed, medline, cinahl, psycinfo, web of science, cnki, wanfang data, vip	Feb-2015	6	NA	Mindfulness	Any	NA	Systematic review	Qualitative
Heckel, 2019[19]	Australia	RCTs and quasi-experimental	Medline, cinahl, psycinfo, embase	May-2018	2	NA	Psychosocial, Education and skills	Cancer	Depression, emotional distress	Systematic review	Quantitative

Author, year	Countries of primary studies	Study design	Databases searched	End date of literature search	# studies included in the umbrella review	# participants, mean age, % female	Intervention type	Disease of care receiver	Health outcomes of caregiver	Review type and synthesis method	Quantitative, qualitative, or mixed-methods
Hopkinson, 2019[20]	Spain, US, UK, Germany, Canada, Brazil, Italy	RCTs and quasi-experimental	Medline, embase, cinahl, psycinfo, cochrane library	Jan-2017	25	Age range 56.6-72.6	Psychosocial	Dementia	Depression, anxiety	Systematic review with meta-analysis	Quantitative
Hopwood, 2018[21]	US, the Netherlands, Spain, Poland, Denmark, Hong Kong, Canada, France, UK, Germany	Any	Cinahl, cochrane library, embase, medline, psycinfo, web of science	Apr-2018	31	NA	Case management, Psychosocial, Education and skills	Dementia	HR-QoL, depression, anxiety, self-rated health	Systematic review	Mixed methods
Irons, 2020[22]	Australia, UK, US, Israel	Any	Medline, pubmed, ebsco, cinahl, embase, psycinfo, cochrane library, scopus, web of science, google scholar	May-2019	17	NA	Relaxation and leisure	Neurological conditions (e.g., dementia, stroke, Parkinson's disease)	HR-QoL, anxiety, depression	Integrative systematic review, meta-ethnography	Mixed methods
Lee, 2020[23]	US, UK, Germany, Spain, Netherlands, Portugal, China, Russia, Peru, Brazil, Denmark, Greece	RCTs	Medline, cinahl, psycinfo, cochrane library	2017	26	3906 caregivers, mean age 60.5, 73.9% female	Case management, Psychosocial, Education and skills, Relaxation and leisure	Dementia	HR-QoL	Systematic review with meta-analysis	Quantitative

**Author, year**	**Countries of primary studies**	**Study design**	**Databases searched**	**End date of literature search**	**# studies included in the umbrella review**	**# participants, mean age, % female**	**Intervention type**	**Disease of care receiver**	**Health outcomes of caregiver**	**Review type and synthesis method**	**Quantitative, qualitative, or mixed-methods**
Lins, 2014[24]	US, Germany, Canada	Any	Cochrane dementia and cognitive improvement group's specialized register, cochrane library, medline, medline in process, embase, cinahl, psyndex, psycinfo, web of science, dimdi databases, springer database, science direct, trial registers, web of science, google scholar	Feb-2013	11	NA	Psychosocial, Education and skills	Dementia	Depression, anxiety	Systematic review with meta-analysis	Mixed methods
Liu, 2018[25]	US, China	RCTs and quasi-experimental	Alois, specialised register of the cochrane dementia and cognitive improvement group	Sep-2017	5	201 caregivers, age range 57.5-71.3, >80% female	Mindfulness	Dementia	Depressive symptoms, anxiety	Systematic review with meta-analysis	Quantitative
Lucero, 2019[26]	NA	RCTs	Pubmed, cinahl, web of science, psycinfo	Jul-2017	12	NA	Case management	Dementia	Depression, anxiety	Systematic review	Quantitative
Maayan, 2014[27]	US, Canada	RCTs	Alois, specialised register of the cochrane dementia and cognitive improvement group	Dec-2012	4	NA	Respite	Dementia	Depression	Systematic review	Quantitative
Maffioletti, 2019[28]	US, the Netherlands, Norway, Scotland, Iceland, HongKong, Australia, Italy, Sweden, Germany	Any	Pubmed, psycinfo, scopus, scielo	Aug-2018	21	NA	Respite	Dementia	Depression, psychological well-being, health status	Systematic review	Mixed methods
Miles, 2020[29]	NA	Any design with comparison group	Medline, embase, web of science, cinahl, pubmed, scopus, psycinfo, cochrane library	Sep-2018	2	NA	Psychosocial, Education and skills	Dementia	NA	Systematic review	Qualitative

**Author, year**	**Countries of primary studies**	**Study design**	**Databases searched**	**End date of literature search**	**# studies included in the umbrella review**	**# participants, mean age, % female**	**Intervention type**	**Disease of care receiver**	**Health outcomes of caregiver**	**Review type and synthesis method**	**Quantitative, qualitative, or mixed-methods**
Minshall, 2019[30]	NA	RCTs	Medline, cinahl, psycinfo, socindex, cochrane library, web of science, Scopus databases, grey literature	Sep-2018	21	2079 caregivers, age range 49-76	Psychosocial, Education and skills	Stroke	Depression, HR-QoL	Systematic review with meta-analysis	Quantitative
Parkinson, 2019[31]	NA	Any	Amed, central, cinahl, medline, nursing and allied health database, psycarticles, psycinfo	2017	9	215 caregivers, age range 48.5-65.6	Mindfulness	Any	Anxiety	Systematic review	Mixed methods
Piersol, 2017[32]	NA	Any	Medline, psycinfo, cinahl, otseeker, cochrane database of systematic reviews	Apr-2014	43	NA	Case management, Psychosocial, Education and skills, Relaxation and leisure	Dementia	HR-QoL, depression, anxiety, physical health	Systematic review	Quantitative
Pritchard, 2020[33]	US, Australia, Europe	Qualitative	Medline, cochrane database of systematic reviews, embase, emcare, cinahl, otseeker	May-2018	7	9-495 caregivers, age range 59.8 (SD=13.9) to 68.7 (SD=8.6)	Education and skills	Dementia	NA	Systematic review with meta-analysis, qualitative synthesis (thematic analysis, framework approach)	Qualitative
Pucciarelli, 2020[34]	UK, Spain, Netherlands, US, Taiwan, Australia	RCTs and quasi-experimental	Pubmed, cinahl, psycinfo	May-2019	16	2187 caregivers, mean age 58, 75% female	Case management, Psychosocial, Education and skills	Stroke	Depression, HR-QoL	Systematic review with meta-analysis	Quantitative
Reilly, 2015[35]	Hong Kong, Netherlands, US, India, Canada, UK	RCTs	Cochrane library, medline, embase, psycinfo, cinahl, lilacs, web of science, campbell collaboration/soro database	Mar-2014	11	NA	Case management	Dementia	HR-QoL, depression, general health	Systematic review with meta-analysis	Quantitative

**Author, year**	**Countries of primary studies**	**Study design**	**Databases searched**	**End date of literature search**	**# studies included in the umbrella review**	**# participants, mean age, % female**	**Intervention type**	**Disease of care receiver**	**Health outcomes of caregiver**	**Review type and synthesis method**	**Quantitative, qualitative, or mixed-methods**
Shaw, 2009[36]	UK, US, Canada, Australia, New Zealand, Germany, Japan	Any	Medline, amed, assia, ibss, bni, cochrane library, dare, hta database, nhs economic evaluation database, cinahl, crisp, econlit, embase, hmic, nrr, psycinfo, scopus, social care online, web of science	2008	174	NA	Respite	Frailty	Depression, anxiety	Systematic review with meta-analysis, meta-synthesis of qualitative studies	Mixed methods
Shim, 2020[37]	NA	RCTs	Pubmed, psycinfo, scopus, cinahl, embase	Feb-2020	9	14-120 caregivers	Mindfulness	Dementia	Cognitive tests, depression, mindfulness, anxiety, biomarkers for stress	Systematic review	Quantitative
Sin, 2018[38]	UK, Australia, US, Hong-Kong, Canada, Sweden, Norway, Netherlands, France, Spain, Denmark, Poland, Germany, Italy, Greece	Any	Medline, psycinfo, cinahl, embase, web of science, assia, central, hta database, dare, eed	Dec-2016	81	4537 caregivers	Psychosocial, Education and skills	Any	Depression, anxiety, distress, HR-QoL	Systematic review	Mixed methods
Smith, 2014[39]	US, UK, Canada	Any	Medline, embase, psycinfo, social policy and practice, cinahl plus, allied and complimentary medicine, social sciences citation index, scopus	Jan-2013	2	NA	Psychosocial, Education and skills	Dementia, stroke	NA	Systematic review	Qualitative

Author, year	Countries of primary studies	Study design	Databases searched	End date of literature search	# studies included in the umbrella review	# participants, mean age, % female	Intervention type	Disease of care receiver	Health outcomes of caregiver	Review type and synthesis method	Quantitative, qualitative, or mixed-methods
Smith, 2019[40]	US, UK, Australia, Sweden, Canada, Ireland, Germany, India, Spain, Taiwan, Thailand	RCTs and quasi-experimental	Embase, medline, cinahl, pubmed, world health organization international clinical trial registry platform registry, clinicaltrials.gov registry	Mar-2019	23	NA	Education and skills	Stroke, frailty	HR-QoL, depression, anxiety	Systematic review with meta-analysis	Quantitative
Teahan, 2020[41]	US, China, Spain, the Netherlands, Taiwan, UK, Peru, Russia, India	RCTs	Cochrane, pubmed, cinahl, psycinfo, embase, assia	Mar-2016	24	NA	Psychosocial, Education and skills	Dementia	Depression, HR-QoL, general health	Systematic review with meta-analysis	Quantitative
Treanor, 2019[42]	US, UK, Denmark, Canada, Netherlands, Australia	RCTs and quasi-experimental	Cochrane central, medline, embase, psycinfo, proquest, open single, web of science	Dec-2018	19	1967 caregivers	Psychosocial, Education and skills	Cancer	HR-QoL, depression, anxiety, emotional distress, physical health status	Systematic review with meta-analysis	Quantitative
Vandepitte, 2016[43]	Canada, US, Germany, Italy	RCTs and quasi-experimental	Pubmed, web of science	Mar-2015	5	NA	Respite	Dementia	Depression, anxiety	Systematic review	Quantitative
Waldron, 2013[44]	N.A.	RCTs	Medline, psycinfo, embase, cinahl, pubmed, cochrane library	Nov-2010	6	1115 caregivers, mean age 56.5 (SD=3.3), 81.9% female	Psychosocial	Cancer	HR-QoL	Systematic review	Quantitative
Wang, 2020[45]	US, the Netherlands, France	RCTs and quasi-experimental	Cinahl, scopus, embase, medline, psycinfo, web of science, world health organization international clinical trials registry platform	Jul-2019	8	NA	Psychosocial	Dementia	Depression, anxiety	Systematic review with meta-analysis	Quantitative
Wiegelmann, 2021[46]	US, UK, Germany, China	RCTs	Pubmed, psycinfo, scopus, cinahl	Aug-2018	37	NA	Psychosocial, Education and skills	Dementia	HR-QoL, depression	Systematic review	Quantitative

Author, year	Countries of primary studies	Study design	Databases searched	End date of literature search	# studies included in the umbrella review	# participants, mean age, % female	Intervention type	Disease of care receiver	Health outcomes of caregiver	Review type and synthesis method	Quantitative, qualitative, or mixed-methods
Zabihi, 2020[47]	Hong Kong, US, Spain, Australia	RCTs	Medline, embase, psycinfo, cinahl, central and online trial registers for randomised controlled clinical trials	Dec-2018	14	NA	Psychosocial	Dementia	Depression (symptoms and diagnosis)	Systematic review with meta-analysis	Quantitative

Abbreviations: RCT, Randomized Controlled Trial; QoL, quality of life; HR-QoL, Health-Related Quality of Life.

Full references of all included reviews (n=47)

1. Akarsu NE, Prince MJ, Lawrence VC, Das-Munshi J. Depression in carers of people with dementia from a minority ethnic background: Systematic review and meta-analysis of randomised controlled trials of psychosocial interventions. *Int J Geriatr Psychiatry*. 2019;34(6):790-806. doi:10.1002/gps.5070
2. Al Daken LI, Ahmad MM. The implementation of mindfulness-based interventions and educational interventions to support family caregivers of patients with cancer: A systematic review. *Perspect Psychiatr Care*. 2018;54(3 PG-441-452):441-452. doi:https://dx.doi.org/10.1111/ppc.12286
3. Bennett S, Laver K, Voigt-Radloff S, et al. Occupational therapy for people with dementia and their family carers provided at home: a systematic review and meta-analysis. *BMJ Open*. 2019;9(11 PG-e026308):e026308. doi:https://dx.doi.org/10.1136/bmjopen-2018-026308
4. Boots LM, de Vugt ME, van Knippenberg RJ, Kempen GI, Verhey FR. A systematic review of Internet-based supportive interventions for caregivers of patients with dementia. *Int J Geriatr Psychiatry*. 2014;29(4 PG-331-344):331-344. doi:https://dx.doi.org/10.1002/gps.4016
5. Bourne P, Camic PM, Crutch SJ. Psychosocial outcomes of dyadic arts interventions for people with a dementia and their informal caregivers: A systematic review. *Heal Soc Care Community*. 2021;29(6):1632-1649. doi:10.1111/hsc.13267
6. Bunn F, Goodman C, Pinkney E, Drennan VM. Specialist nursing and community support for the carers of people with dementia living at home: An evidence synthesis. *Heal Soc Care Community*. 2016;24(1):48-67. doi:10.1111/hsc.12189
7. Cheng ST, Li KK, Losada A, et al. The effectiveness of nonpharmacological interventions for informal dementia caregivers: An updated systematic review and meta-analysis. *Psychol Aging*. 2020;35(1 PG-55-77):55-77. doi:https://dx.doi.org/10.1037/pag0000401
8. Corry M, Neenan K, Brabyn S, Sheaf G, Smith V. Telephone interventions, delivered by healthcare professionals, for providing education and psychosocial support for informal caregivers of adults with diagnosed illnesses. *Cochrane Database Syst Rev*. 2019;5(PG-CD012533):CD012533. doi:https://dx.doi.org/10.1002/14651858.CD012533.pub2
9. Cuthbert CA, King-Shier K, Ruether D, Tapp DM, Culos-Reed SN. What is the state of the science on physical activity interventions for family caregivers? A systematic review and RE-AIM evaluation. *J Phys Act Heal*. 2017;14(7):578-595. doi:10.1123/jpah.2016-0280
10. Dam AE, de Vugt ME, Klinkenberg IP, Verhey FR, van Boxtel MP. A systematic review of social support interventions for caregivers of people with dementia: Are they doing what they promise? *Maturitas*. 2016;85(PG-117-130):117-130. doi:https://dx.doi.org/10.1016/j.maturitas.2015.12.008
11. Doyle KL, Toepfer M, Bradfield AF, et al. Systematic review of exercise for caregiver-care recipient dyads: What is best for spousal caregivers - Exercising together or not at all? *Gerontologist*. 2021;61(6):E283-E301. doi:10.1093/geront/gnaa043
12. Du Preez J, Millsteed J, Marquis R, Richmond J. The Role of Adult Day Services in Supporting the Occupational Participation of People with Dementia and Their Carers: An Integrative Review. *Healthcare*. 2018;6(2 PG-08):8. doi:https://dx.doi.org/10.3390/healthcare6020043
13. Forster A, Brown L, Smith J, et al. Information provision for stroke patients and their caregivers. *Cochrane Database Syst Rev*. Published online November 14, 2012. doi:10.1002/14651858.cd001919.pub3
14. Gabriel I, Creedy D, Coyne E. A systematic review of psychosocial interventions to improve quality of life of people with cancer and their family caregivers. *Nurs Open*. 2020;7(5):1299-1312. doi:10.1002/nop2.543
15. Goeman D, Renehan E, Koch S. What is the effectiveness of the support worker role for people with dementia and their carers? A systematic review. *BMC Health Serv Res*. 2016;16(1). doi:10.1186/s12913-016-1531-2
16. González-Fraile E, Ballesteros J, Rueda JR, Santos-Zorrozúa B, Solà I, McCleery J. Remotely delivered information, training and support for informal caregivers of people with dementia. *Cochrane Database Syst Rev*. 2021;2021(1). doi:10.1002/14651858.CD006440.pub3
17. Greenwood N, Pelone F, Hassenkamp AM. General practice based psychosocial interventions for supporting carers of people with dementia or stroke: a systematic review. *BMC Fam Pract*. 2016;17(PG-3):3. doi:https://dx.doi.org/10.1186/s12875-015-0399-2
18. Li G, Yuan H, Zhang W. The Effects of Mindfulness-Based Stress Reduction for Family Caregivers: Systematic Review. *Arch Psychiatr Nurs*. 2016;30(2):292-299. doi:10.1016/j.apnu.2015.08.014
19. Heckel L, Heynsbergh NL, Livingston PM. Are cancer helplines effective in supporting caregivers? A systematic review. *Support Care Cancer*. 2019;27(9):3219-3231. doi:10.1007/s00520-019-04807-z
20. Hopkinson MD, Reavell J, Lane DA, Mallikarjun P. Cognitive Behavioral Therapy for Depression, Anxiety, and Stress in Caregivers of Dementia Patients: A Systematic Review and Meta-Analysis. *Gerontologist*. 2019;59(4 PG-343-362):e343-e362. doi:https://dx.doi.org/10.1093/geront/gnx217
21. Hopwood J, Walker N, McDonagh L, et al. Internet-Based Interventions Aimed at Supporting Family Caregivers of People With Dementia: Systematic Review. *J Med Internet Res*. 2018;20(6 PG-e216):e216. doi:https://dx.doi.org/10.2196/jmir.9548
22. Yoon Irons J, Garip G, Cross AJ, Sheffield D, Bird J. An integrative systematic review of creative arts interventions for older informal caregivers of people with neurological conditions. *PLoS One*. 2020;15(12 December). doi:10.1371/journal.pone.0243461
23. Lee M, Ryoo JH, Crowder J, Byon HD, Wiiliams IC. A systematic review and meta-analysis on effective interventions for health-related quality of life among caregivers of people with dementia. *J Adv Nurs*. 2020;76(2 PG-475-489):475-489. doi:https://dx.doi.org/10.1111/jan.14262
24. Lins S, Hayder-Beichel D, Rucker G, et al. Efficacy and experiences of telephone counselling for informal carers of people with dementia. *Cochrane Database Syst Rev*. 2014;(9 PG-CD009126):CD009126.

doi:https://dx.doi.org/10.1002/14651858.CD009126.pub2

25. Liu Z, Chen Q lin, Sun Y ying. Mindfulness training for psychological stress in family caregivers of persons with dementia: A systematic review and meta-analysis of randomized controlled trials. *Clin Interv Aging*. 2017;12:1521-1529. doi:10.2147/CIA.S146213
26. Lucero RJ, Fehlberg EA, Patel AGM, et al. The effects of information and communication technologies on informal caregivers of persons living with dementia: A systematic review. *Alzheimer's Dement Transl Res Clin Interv*. 2019;5:1-12. doi:10.1016/j.trci.2018.11.003
27. Maayan N, Soares-Weiser K, Lee H. Respite care for people with dementia and their carers. *Cochrane Database Syst Rev*. 2014;2014(1). doi:10.1002/14651858.CD004396.pub3
28. Maffioletti VLR, Baptista MAT, Santos RL, Rodrigues VM, Dourado MCN. Effectiveness of day care in supporting family caregivers of people with dementia: A systematic review. *Dement Neuropsychol*. 2019;13(3 PG-268-283):268-283. doi:https://dx.doi.org/10.1590/1980-57642018dn13-030003
29. Miles L, McCausland BMS, Patel HP, Amin J, Osman-Hicks VC. A systematic review of the provision and efficacy of patient and carer information and support (PCIS) interventions for patients with dementia and their informal carers. *Aging Clin Exp Res*. 2020;32(12):2439-2448. doi:10.1007/s40520-019-01428-8
30. Minshall C, Pascoe MC, Thompson DR, et al. Psychosocial interventions for stroke survivors, carers and survivor-carer dyads: a systematic review and meta-analysis. *Top Stroke Rehabil*. 2019;26(7 PG-554-564):554-564. doi:https://dx.doi.org/10.1080/10749357.2019.1625173
31. Parkinson B, Lawrence M, McElhinney E, Booth J. Mindfulness for people with long-term conditions and their family caregivers: A systematic review. *Complement Ther Clin Pract*. 2019;34:76-86. doi:10.1016/j.ctcp.2018.10.019
32. Piersol CV, Canton K, Connor SE, Giller I, Lipman S, Sager S. Effectiveness of Interventions for Caregivers of People With Alzheimer's Disease and Related Major Neurocognitive Disorders: A Systematic Review. *Am J Occup Ther*. 2017;71(5):p1-7105180020. doi:10.5014/ajot.2017.027581
33. Pritchard E, Cussen A, Delafosse V, Swift M, Jolliffe L, Yeates H. Interventions supporting caregiver readiness when caring for patients with dementia following discharge home: A mixed-methods systematic review. *Australas J Ageing*. 2020;39(3 PG-239-250):e239-e250. doi:https://dx.doi.org/10.1111/ajag.12765
34. Pucciarelli G, Lommi M, Magwood GS, et al. Effectiveness of dyadic interventions to improve stroke patient-caregiver dyads' outcomes after discharge: A systematic review and meta-analysis study. *Eur J Cardiovasc Nurs*. 2021;20(1):14-33. doi:10.1177/1474515120926069
35. Reilly S, Miranda-Castillo C, Malouf R, et al. Case management approaches to home support for people with dementia. *Cochrane Database Syst Rev*. 2015;2017(6). doi:10.1002/14651858.CD008345.pub2
36. Shaw C, McNamara R, Abrams K, et al. Systematic review of respite care in the frail elderly. *Health Technol Assess*. 2009;13(20 PG-1-224, iii):1-224, iii. doi:https://dx.doi.org/10.3310/hta13200
37. Shim M, Tilley JL, Im S, Price K, Gonzalez A. A Systematic Review of Mindfulness-Based Interventions for Patients with Mild Cognitive Impairment or Dementia and Caregivers. *J Geriatr Psychiatry Neurol*. 2021;34(6):528-554. doi:10.1177/0891988720957104
38. Sin J, Henderson C, Spain D, Cornelius V, Chen T, Gillard S. eHealth interventions for family carers of people with long term illness: A promising approach? *Clin Psychol Rev*. 2018;60:109-125. doi:10.1016/j.cpr.2018.01.008
39. Smith R, Greenwood N. The impact of volunteer mentoring schemes on carers of people with dementia and volunteer mentors: a systematic review. *Am J Alzheimer's Dis Other Dementias*. 2014;29(1 PG-8-17):8-17. doi:https://dx.doi.org/10.1177/1533317513505135
40. Smith TO, Pearson M, Pfeiffer K, Crotty M, Lamb SE. Caregiver Interventions for Adults Discharged from the Hospital: Systematic Review and Meta-Analysis. *J Am Geriatr Soc*. 2019;67(9):1960-1969. doi:10.1111/jgs.16048
41. Teahan Á, Lafferty A, McAuliffe E, et al. Psychosocial Interventions for Family Carers of People With Dementia: A Systematic Review and Meta-Analysis. *J Aging Health*. 2020;32(9):1198-1213. doi:10.1177/0898264319899793
42. Treanor CJ, Santin O, Prue G, et al. Psychosocial interventions for informal caregivers of people living with cancer. *Cochrane Database Syst Rev*. 2019;2019(6). doi:10.1002/14651858.CD009912.pub2
43. Vandepitte S, Van Den Noortgate N, Putman K, Verhaeghe S, Verdonck C, Annemans L. Effectiveness of respite care in supporting informal caregivers of persons with dementia: a systematic review. *Int J Geriatr Psychiatry*. 2016;31(12):1277-1288. doi:10.1002/gps.4504
44. Waldron EA, Janke EA, Bechtel CF, Ramirez M, Cohen A. A systematic review of psychosocial interventions to improve cancer caregiver quality of life. *Psychooncology*. 2013;22(6 PG-1200-1207):1200-1207. doi:https://dx.doi.org/10.1002/pon.3118
45. Wang S, Bressington DT, Leung AYM, Davidson PM, Cheung DSK. The effects of bibliotherapy on the mental well-being of informal caregivers of people with neurocognitive disorder: A systematic review and meta-analysis. *Int J Nurs Stud*. 2020;109. doi:10.1016/j.ijnurstu.2020.103643
46. Wiegelmann H, Speller S, Verhaert LM, Schirra-Weirich L, Wolf-Ostermann K. Psychosocial interventions to support the mental health of informal caregivers of persons living with dementia - a systematic literature review. *BMC Geriatr*. 2021;21(1 PG-94):94. doi:https://dx.doi.org/10.1186/s12877-021-02020-4
47. Zabihi S, Lemmel FK, Orgeta V. Behavioural Activation for Depression in Informal Caregivers: A Systematic Review and Meta-Analysis of Randomised Controlled Clinical Trials. *J Affect Disord*. 2020;274(PG-1173-1183):1173-1183. doi:10.1016/j.jad.2020.03.124

Supplementary Table 7. Summary of findings for interventions involving case management

First author, year	Summary of findings on effectiveness
Goeman, 2016	Inconclusive findings with similar number of studies reporting either positive effects or no effects.
Greenwood, 2016	No significant effect.
Hopwood, 2018	No significant effect. However, some multicomponent interventions in this review show promise for reducing caregivers' depressive symptoms and anxiety.
Lee, 2020	No significant effect: Hedges G was 0.135 (95% CI=-0.076, 0.346, *p*=0.210). However, multicomponent interventions were effective: Hedges G 0.255 (95% CI=0.054, 0.457, *p*=0.013).
Lucero, 2019	Telephone-based intervention showed significant decrease in depression.
Piersol, 2017	No significant effect.
Reilly, 2015	Significant reduction in depression and short-term health (MD=-0.08, 95% CI=-0.16, -0.01, n=2888, *p*=0.03), but no effect on longer follow-ups.
Pucciareli, 2020	No significant effect: SMD=-0.19, 95% CI=-0.40, 0.00, *p*=0.05.

Abbreviations: CI, confidence interval; SMD, standardized mean difference; MD, mean difference.

Kirvalidze M, *et al. BMJ Open* 2023; 13:e068646. doi: 10.1136/bmjopen-2022-068646

Supplementary Table 8. Summary of findings for psychosocial, psychoeducational, and skills-building interventions

First author, year	Summary of findings on effectiveness
**Reviews that included both psychosocial and educational interventions (n=19)**	
Akarsu, 2019	In random effects MA, interventions (psychological, multicomponent, and educational) to address depression in ethnic minority caregivers relative to the control condition were associated with an overall mean reduction in depression scores (SMD=-0.17, 95% CI=-0.29, -0.05, *p*=0.005). Heterogeneity was negligible (I2=0.0%, *p*=0.547).
Gonzalez-Fraile, 2021	Remotely delivered interventions involving training, support, or both (with or without information) do not affect caregiver depressive symptoms (SMD=-0.05, 95% CI=-0.22, 0.12, I2=9%) or caregiver HR-QoL (SMD=0.10, 95% CI=-0.13, 0.32, I2=0%). However, these interventions may result in a slight improvement in caregiver depressive symptoms (SMD=-0.25, 95% CI=-0.43, -0.06, I2 = 53%) compared to a control condition of information alone. Interventions may result in little or no difference in caregiver HR-QoL when compared with information alone (SMD=-0.03, 95% CI=-0.28, 0.21, I2=0%).
Greenwood, 2016	One study reported no significant effects in the proportion of caregivers with scores of CES-D ≥16 (i.e., at risk of depression) between the intervention and control groups. Another study reported significant positive changes over time (*p*=0.009) for improving depression scores.
Hopwood, 2018	Overall, studies assessing psychological support suggested a positive effect on a variety of factors, including improving caregiver distress, depression, anxiety, and strain. Some multiple-component interventions showed promise in reducing stress, anxiety, and depressive symptoms for family caregivers and in increasing self-efficacy.
Lee, 2020	CBT was effective, Hedges G=0.767 (95% CI=0.142, 1.391, *p*=0.016); Psychoeducation was not effective, Hedges G=0.163 (95% CI=-0.001, 0.328, *p*=0.052); Social support and cognitive rehabilitation were not effective, G=0.231 (95% CI=-0.104, 0.567, *p*=0.176 and G=0.010 (95% CI=-0.208, 0.229, *p*=0.926), respectively. However, multicomponent interventions were effective: Hedges G=0.255 (95% CI=0.054, 0.457, *p*=0.013).
Lins, 2014	Without educational material and workbook: significant pooled difference in depressive symptoms between the telephone counselling group and the control group (SMD=0.32, 95% CI=0.01, 0.63, *p*=0.04). With educational material and workbook: reduction of depressive symptoms in the intervention group and an increase in the control group after months: -4.1% points in the intervention group (from 7.20% to 3.10%) and 0.56% points in the control group (from 7.44% to 8%). The difference between the groups was statistically significant (*p*=0.01).
Piersol, 2017	Communication skills training: increased QoL and well-being; Coaching: reduced stress and depression; Psychoeducation: improved QoL, well-being, reduced depression; Group interventions: strong evidence indicates that in person caregiver support groups led by professionals improved caregiver well-being and reduced depression; CBT: compelling evidence that interventions focused on cognitive reframing and skills training reduced caregiver depression and anxiety.
Teahan, 2020	Interventions were shown to have a significant effect (*p*=0.002) on caregivers' depression levels pre- and postintervention (SMD=-0.36, 95% CI=-0.60, -0.13, 11 trials, N=1856 participants). The interventions were shown to have a significantly small to moderate effect size (*p*<0.01) on the general health scores of caregivers pre- and postintervention (SMD=0.34, 95% CI=0.18, 0.51, 7 trials, N=1503 participants). Although the overall effect on QoL was not statistically significant (*p*=0.12), there was a trend in favor of intervention (SMD=0.63, 95% CI=-0.16, 1.43, 3 interventions, N=201).
Wiegelmann, 2021	4 out of 11 psychoeducational interventions described positive results, while 7 reported no significant effect. 1 out of 4 counselling interventions reported improvement in depression. 4 out of six CBT interventions reported positive effects. 3 out of 8 psychoeducational interventions reported improvement in QoL. 1 out of 6 counselling interventions improved QoL. 1 CBT intervention measuring QoL failed to report significant effects. Neither of the 2 befriending interventions reported positive changes in QoL.
Zabihi, 2020	MA of effects of behavioral activation showed that results significantly favored intervention in reducing caregiver depressive symptoms at post-treatment (8 studies, 815 participants, SMD=-0.68, 95% CI=-1.14,

	-0.22). There was high heterogeneity between studies (I2=89%). Long-term effects were also favorable (2 studies, 235 participants, SMD=-0.99, 95% CI=-1.26, -0.71, I2= 92%. Behavioral activation was also associated with decreased risk of depression diagnosis (3 studies, 854 participants, OR=0.35, 95% CI=0.19, 0.67, efficacy at 3-12 months) and was superior to psychoeducation in one study.
Gabriel, 2020	All 12 studies assessed psychological/emotional well-being; 4 studies report statistically significant improvement for dyads, 2 studies report significant improvement for caregivers. Remaining 6 studies report no significant change. As for physical well-being, all 12 studies assessed it; 5report significant improvement for dyads, while 1 reports it for care receivers only, 6 report no change.
Heckel, 2019	No significant effect on depression (1 study), another study showed positive effects for reducing emotional distress.
Treanor, 2019	Considering the pooled and unpooled findings, psychosocial interventions may improve slightly QoL immediately post-intervention, but this benefit may not be maintained in the longer term (6-2 months later). Pooled and unpooled findings indicated that psychosocial interventions probably have little to no effect on depression, anxiety or emotional distress for caregivers. Evidence for physical outcomes is insufficient.
Waldron, 2013	Nil to small effects on QoL outcomes were found in the 4 studies where QoL effect sizes could be calculated, and 2 of these reported significant changes in QoL.
Corry, 2019	There is little or no difference between telephone support interventions and usual care for QoL at the end of intervention (SMD=-0.02, 95% CI=-0.24, 0.19, 4 studies, 364 caregivers) and at short-term follow-up (3 months) (SMD=0.00, 95% CI=-4.43, 4.43, 1 study, 128 caregivers). The effects of telephone interventions on depression at the end of intervention were uncertain (SMD=-0.37, 95% CI=-0.70, -0.05, 9 studies, 792 caregivers). Telephone interventions may have little or no effect at medium-term follow-up (3-6 months) (SMD=-0.05, 95% CI=-0.56, 0.45, 3 studies, 227 caregivers). Telephone interventions compared with usual care may slightly decrease anxiety levels at the end of intervention and short-term follow-up (2 studies, no MA). Telephone support interventions, when compared to usual care may have little or no effect on caregiver physical health at the end of intervention (SMD=-0.09, 95% CI=-0.35, 0.17, 2 studies, 248 caregivers).
Sin, 2018	Of those studies that assessed effectiveness of interventions, 44% reported equivocal findings (no effect), 12% saw negative effects (control group more favorable than intervention) and only 32% reported positive effects. While caregivers reported positive experiences in using the interventions (as per qualitative findings), no significant changes in their objective outcomes were identified.
Forster, 2012	Out of 3 RCTs measuring depression, 2 showed no significant effect, while 1 reported less depression in the intervention group. Out of 4 RCTs measuring QoL, 3 report no effects, while 1 reported higher QoL in the intervention group.
Minshall, 2019	Based on MA of 6 RCTs measuring depression, the effect is insignificant (pooled effect size=-0.20, 95% CI -0.40, 0.00, n=279 in intervention group, n=282 in control group). One trial reported improvements in caregiver QoL.
Pucciarelli, 2020	Caregivers who received an educational intervention were found to have lower depression levels than those in the control group, although no significant differences were observed between these two groups (SMD=-0.19, 95% CI=-0.40, 0.00, *p*=0.05).
**Reviews that included only psychosocial (n=2) or only educational (n=2) interventions**	
Bennett, 2019 (occupational therapy)	Data from six studies that measured the emotional distress family caregivers felt were combined, and demonstrated a small, statistically significant result in favor of those receiving occupational therapy (SMD=-0.23, 95% CI=-0.42, -0.05, I2=41%). Only 3 studies measured overall QoL of the caregivers of people with dementia. Pooled data from 2 of these studies produced a large, significant between-group difference after the intervention (SMD=0.99, 95% CI=0.66, 1.33, I2=2%).
Smith, 2019 (training)	There was no benefit on caregiver HR-QoL compared with the control group at 3-6 months (SMD=0.20; 95% CI=-1.12, 1.52, N=638, I2=98%) or 12 months (SMD=0.46, 95% CI=-0.34, 1.27, N=415, I2=94%). There was no benefit from the caregiver intervention on caregiver depression compared with the control group at 3 to 6 months (SMD=0.03,95% CI=-0.08, 0.14, N=1239, I2=0%) or 12 months (SMD=-0.71, 95% CI=-2.23, 0.81, N=613, I2=99%). For anxiety, 1

	study reported significant effect, while another reported no improvement.
Hopkinson, 2019 (psychosocial)	Caregivers receiving CBT demonstrated statistically significant reductions in depression (n=12; 995 participants; SMD=-0.34; 95% CI=-0.47, -0.21, *p*<0.001 relative to comparator groups immediately after the intervention endpoint and in 3 months (n=3; SMD=-0.99, 95% CI=-1.35, -0.64, *p*<0.001). There was no significant difference in caregiver anxiety (n=10, 829 participants, SMD=0.10; 95% CI=-0.18, 0.39, *p*=0.47).
Wang, 2020 (psychosocial)	For depression, the overall effect for the 2 types of bibliotherapy was significant at Z=1.99 (random effect model, SMD=-0.74, 95% CI=-1.47, -0.01, *p*=0.05). However, when considered separately, only the video based bibliotherapy significantly reduced depression at Z=2.78 (random effect model, SMD=-2.11, 95% CI=-3.6, -0.62, *p*=0.005). For anxiety, the heterogeneity of the 3 studies included in MA was low (I2=22%), the overall effect of bibliotherapy on anxiety was significant at Z=2.30, *p*=0.02 (random effect model, SMD=-0.22, 95% CI=-0.41, -0.33).

Abbreviations: RCT, randomized controlled trial; CI, confidence interval; SMD, standardized mean difference; CBT, cognitive behavioral therapy; QoL, quality of life; HR-QoL, health-related quality of life; MA, meta-analysis.

Supplementary Table 9. Summary of findings for interventions involving respite care

First author, year	Summary of findings on effectiveness
Maayan, 2019	Analysis of the available data showed no significant effects on caregiver outcomes when respite care was compared with no respite care in 3 studies.
Maffioletti, 2019	5 studies reported positive effects on depression, 3 on psychological/psycho-somatic complaints, and 4 studies also report improved health status and greater psychological well-being. 2 studies report no changes in caregiver outcomes.
Vandepitte, 2016	Reported benefits of day care provision for caregivers were small and mixed (33% were effective). The only included in-home respite care program indicated some beneficial effects for caregivers.
Shaw, 2009	MA showed insignificant positive effect in favor of respite care at short term, but no effect in long term. Evidence from narratively reviewed studies indicated that respite does not have a significant impact on psychological well-being or depression when compared with normal care. Few studies (with lower quality of design, not in MA) reported decrease in depression, but at short follow-up times. MA of randomized/quasi-experimental studies measuring caregiver anxiety as an outcome did not demonstrate any significant effects.

Abbreviations: MA, meta-analysis.

Kirvalidze M, *et al. BMJ Open* 2023; 13:e068646. doi: 10.1136/bmjopen-2022-068646

Supplementary Table 10. Summary of findings for interventions involving relaxation, physical activity, or leisure

First author, year	Summary of findings on effectiveness
Cheng, 2020	Miscellaneous interventions were shown to reduce stress and depressive symptoms. For this group of interventions, Hedges G=-0.49, n=12, N=758. Interestingly, in this review, multicomponent and miscellaneous interventions had the largest effects.
Lee, 2020	MA of the 2 studies showed that the Hedges G was 0.576 (95% CI=0.035, 1.118, *p*=0.037), indicating that there was a statistically significant difference between the control group and the intervention group on improving caregiver HR-QoL.
Piersol, 2017	Exercise programs showed improved caregiver physical health and decreased stress. Adapted leisure programs showed improved well-being.
Irons, 2020	For outcomes of interest, pre-post effect sizes revealed some benefits of the intervention: large effect sizes were detected for singing on well-being (Cohen's d=1.04), and positive-negative mood (Cohen's d=1.29); and for music making on relaxation (Cohen's d=1.91), comfort (Cohen's d=1.74), and happiness (Cohen's d=1.19). Some studies indicated decreases in some aspects of well-being which were not consistent with other aspects reported. For example, one study reported increases in stress, but decreases in anxiety and depression.
Cuthbert, 2017	Across all studies, statistically significant results for the following outcomes in the physical activity groups were reported: (1) decrease in depression rates; (2) decrease in anger scores; (3) improvement in sleep quality; (4) lower systolic and diastolic blood pressure reactivity.
Doyle, 2020	Results were mixed across and within DyEx and DySplit studies. Of the DyEx studies, 6 examined caregiver psychosocial well-being. Beneficial outcomes in 4 studies indicated significant improvements in mental health and QoL of caregivers. In 3 DySplit interventions, caregivers experienced significant improvements to mental health when they were offered non-exercise interventions of either respite or a dyadic support group, while their care receivers exercised. 3 DyEx studies measured caregiver physical health. A resistance training intervention noted significant increases in muscle mass, strength, and physical function, but no significant difference in gait speed. Self-reported physical outcomes were equivocal.

Abbreviations: CI, confidence interval; QoL, quality of life; HR-QoL, health-related quality of life; DyEx, dyadic interventions; DySplit, non-dyadic interventions.

Supplementary Table 11. Summary of findings for interventions involving mindfulness-based activities

First author, year	Summary of findings on effectiveness
Cheng, 2020	Mindfulness-based interventions reduced depressive symptoms. For subjective well-being G=0.31, k=6, N=212. For depression G=-0.58, k=7, N=258.
Liu, 2018	MBSR vs active controls immediately after intervention: MBSR decreased depressive symptoms of caregivers compared with the active-control interventions: SMD=-0.63 (95% CI=-0.98, -0.28, *p*<0.001). MBSR could reduce caregivers' anxiety compared with the active-control group immediately after the intervention period: MD=-7.50 (95% CI=-13.11, -1.89, *p*=0.009). MBSR vs inactive controls immediately after intervention: no clear effect on depressive symptoms of caregivers in the MBSR group compared with the inactive controls immediately after the intervention period. MBSR may reduce caregivers' levels of anxiety compared with the inactive control immediately after the intervention period MD=-7.27 (95% CI=-14.92, 0.38, *p*=0.06. MBSR vs controls at follow-up: compared with the active-control intervention, the results for depressive symptoms suggested that there may be little or no effect of MBSR MD=-0.16 (95% CI=-0.71, 0.39, *p*=0.57). Compared with the inactive control, the results for caregivers' depressive symptoms slightly favored MBSR MD=-3.00 (95% CI=-8.52, 2.52, *p*=0.29).
Shim, 2020	3 studies show that cognitive functioning was significantly improved in MBI conditions compared to both active and passive control conditions. 4 studies reported statistically significant improvements in self-reported measures of stress or distress in the MBI condition at post-treatment or follow-up with treatment effects in the small to medium range (G=-0.078 to -0.602). 3 out of 7 studies examining depression/anxiety reported relative improvements. When compared with passive control interventions, caregivers in MBIs reported significant improvements in QoL. Long term effects were not that pronounced. No treatment differences in outcomes were found at 3- and 9-month follow-up assessments.
Parkinson, 2019	Some improvements were noted for patients' and their caregivers' anxiety scores following intervention, but these changes were not significant at post treatment or at follow-up. 1 study provided a narrative account of results and reported no change in anxiety scores following intervention for participants or family caregivers. Significant (*p*=0.01) improvements in the tension/anxiety score were reported in 1 study. Caregivers experienced a mean reduction depression score in 5 studies, but the improvements in depression were often small and only significant in 3 studies.

Abbreviations: MBSR, Mindfulness-Based Stress Reduction; MBI, Mindfulness-Based Interventions; MCI, mild cognitive impairment; SMD, standardized mean difference; MD, mean difference; CI, confidence interval.

Supplementary Table 12. Coding table for qualitative data

Theme (and definition)	Category (and definition)	Sub-category (and definition)	Fourth level construct codes	Third level construct verbatim	References
**Intervention outcomes:** this category describes potential beneficial and/or harmful effects to the caregiver from participating in the interventions.	**Health outcomes:** health benefits experienced and expressed by caregivers because of participating in support interventions.	**NA**	Education/skills: hospital skills-building brings less anxiety, depression, burden, better quality of life	When interventions are provided to the caregiver in the context of the hospital setting to enhance these skills, there is a likelihood of reducing anxiety, depression, caregiver burden and improving QoL.	Pritchard
			Mindfulness: benefits include relaxation, less depression, less worry, and anxiety	Triangulated qualitative and quantitative data suggests MBIs can improve relaxation, ease anxiety, and reduce depression for people with LTCs and their family caregivers. […] The reported psychological benefits included increased relaxation, reduced worry, and reductions in anxiety.	Parkinson
**Intervention outcomes:** this category describes potential beneficial and/or harmful effects to the caregiver from participating in the interventions.	**Social outcomes:** intervention outcomes related to impact on caregiver's social life, day-to-day routine and relationship with care receiver and other people.	**Practical social outcomes:** changes in caregivers' day-to-day routine, resulting from acquiring new skills or knowledge.	Case management: nurse and carer working together decreases burden	A shared approach to care was found to be vital in decreasing burden among family members.	Goeman
			Case management: prevents crises	Case management offered potential benefit to people with dementia, their carers and community-based professionals through continuity of care by a named trusted individual that could act proactively to prevent a crisis.	Goeman
			Case management: admiral nurses have good relationship with carers and care receivers	Admiral nurse: Positive outcomes in the carer/key worker relationship to be linked to the quality of the relationship and involve the carer and professional care worker actively including and working with the person with dementia.	Goeman
			Psychoeducation: brings satisfaction, coping skills, competence, confidence, less burden, less loneliness	Increased satisfaction with support, coping skills, caregiving competence, confidence, and decreased burden and loneliness.	Smith
			Psychoeducation: brings increased knowledge, coping, confidence, less isolation	Increased knowledge of the disease and caring was described in 3 papers, in addition to increased coping with caregiving (2 papers) confidence in caregiver skills (2 papers) and reduced feelings of isolation (1 paper).	Boots
			Education/skills: Strong relationship between health staff and carers needed for continued informal care	When health professionals build strong relationships with the caregiver, they are more likely to be able to support the readiness of the caregiver to continue their role.	Pritchard
			Education/skills: when needs are met, coping is better	Caregivers identified when personal characteristics of both patient and caregiver are considered, caregivers are included as an integral part of the team (as they requested), and they receive timely and effective information, the level of stress is likely to reduce, and their ability to cope to be ready for discharge is likely to increase.	Pritchard

Kirvalidze M, *et al. BMJ Open* 2023; 13:e068646. doi: 10.1136/bmjopen-2022-068646

			Education/skills: carers' coping mechanisms promoted	The interventions addressed the need for clear information about the disease, identification of psychological response, development of coping mechanisms and assessment of the home environment.	Pritchard
			Counselling: counsellors help with seemingly helpless situations	Being able to describe and discuss problematic situations helps carers in their daily lives when they do not know how what to do next, because, from their perspective, they have tried everything but have not been able to resolve the situation.	Lins
			Counselling: counsellors knowledgeable about services is helpful	Carers find the information provided helpful and are grateful for it. The information and advice help them to do certain things better, which leads to them feeling happier with themselves.	Lins
			Respite: free time used for leisure or chores	The respite time gained through this type of care tended to be used for necessary everyday chores. Also used to carry out pleasurable activities on a regular basis.	Shaw
			Respite: carers more in control of time	Shorter-term respite such as day care was felt to give structure to the carer's week along with a sense of normality as the free time matched the ebb and flow of caregiving activities.	Shaw
			Respite: use of service can be more emotionally and physically demanding than not use	There were many hassles (i.e., inconveniences, irritations and frustrations that were troublesome to deal with) involved in the preparation for respite care. These hassles were costly in terms of the physical and emotional energy involved in dealing with them, and these costs were weighed against the benefits received from respite.	Shaw
			Respite: brings organized routine	Regular attendance is also perceived by carers to provide their care recipients with a sense of structure and routine to their daily lives.	Du Preez
			Creative arts: benefits include learning, stimulation, normalizing care receivers' lives	Reported benefits included new learning, stimulation and the accessibility of the activities, which creates a sense of normalcy and altered perceptions about PWD's abilities.	Bourne
			Creative arts: artmaking helps with coping and resilience	Artmaking was seen to cope and practice resilience, where this approach allowed caregivers to make the most of their lives and to deal with an uncertain future: "It slightly reminds me of the turmoil but at the same time it says to me yes, you got through it […] Because they [a series of paintings] were part of the process of me becoming well again."	Irons
**Intervention outcomes:** this category describes potential beneficial and/or harmful effects to the caregiver from participating in the interventions.	**Social outcomes:** intervention outcomes related to impact on caregiver's social life, day-to-day routine and relationship with care receiver and other people.	**Emotional social outcomes:** changes in feelings and emotional reflections of caregivers, resulting from an intervention.	Psychoeducation: Online intervention reduces stress	Caregivers subjectively identified that the online intervention helped them cope with the stress of caregiving.	Sin
			Education/skills: When needs are met, stress is reduced	Caregivers identified when personal characteristics of both patient and caregiver are considered, caregivers are included as an integral part of the team (as they requested), and they receive timely and	Pritchard

	effective information, the level of stress is likely to reduce, and their ability to cope to be ready for discharge is likely to increase.	
Counselling: carers appreciate conversations beyond their nursing duties	Carers also find it helpful to have conversations about other things that do not remind them of their nursing duties.	Lins
Psychosocial: Benefits of peer support include less discomfort and satisfaction	Qualitative measures on well-being reported improvements on subjective satisfaction, and reduced feelings of discomfort and embarrassment.	Dam
Psychosocial: group support brings relief through sharing	Qualitative interview data on support groups for only caregivers demonstrated a sense of relief through sharing problems and new social contact.	Dam
Psychosocial: peer support brings emotional support and enjoyment	Qualitative social support outcomes demonstrated reduced feelings of social isolation, increased emotional support, and mutual sharing and enjoyment after receiving peer support.	Dam
Counselling: simple chit chat brings relief	The participants in the control group had conversations only about general topics such as the weather, television, movies, news, or social activities. Such a conversation about common topics was found to be a helpful alternative to relieve carers.	Lins
Counselling: self-expression beneficial	Carers benefit from being able to express their helplessness.	Lins
Respite: respite gives physical break but not mental break/relaxation	The qualitative review also pointed out that a physical break from the care recipient was probably not sufficient in itself to provide the mental break that was needed by most carers to improve their well-being. A mental break meant freedom from worry and total disengagement from the caring role. This was only achieved in the knowledge that the care recipient was happy, safe and well cared for.	Shaw
Respite: caregivers feel guilty to not care	The strong commitment to caring and eventual realisation that they cannot cope on their own leads not only to feelings of failure but also to feelings of guilt. The negative connotations of respite and the feelings of abandonment also contribute to guilt.	Shaw
Respite: sense of failure when admitting need of help	The types of attitudes discussed above that drive the desire to maintain the care recipient at home can lead to a sense of failure when a need for support is acknowledged, particularly in the presence of negative attitudes to respite care itself.	Shaw
Respite: feelings of guilt and perceived abandonment with respite	In the early stages it was felt by carers that attendance at day care could confront a care recipient's denial of their condition, and so a pretence of normality is maintained as long as possible to maintain the integrity of the care recipient's self-image. The outcome in	Shaw

	relation to many of the issues described above is the carer's feeling of guilt.	
Respite: more guilt among women	If respite is seen as benefiting only the carer this will be viewed in a selfish light, which can trigger guilt, particularly in women who have spent their lives caring for others	Shaw
Respite improves mood and engagement with care recipient	Carers reported increased engagement with their care recipients upon returning from adult day service attendance. This was due to their improved mood and them sharing interesting events that occurred throughout the day.	Du Preez
Creative arts: singing brings enjoyment	All six studies included comments on improved mood or well-being and three on the extended impact in terms of mood, the enjoyment of singing at home and socializing with other members outside of the group.	Bourne
Creative arts: familiar songs promote reminiscence	Participants also reported gaining enjoyment from singing familiar songs that facilitated reminiscence.	Bourne
Creative arts: benefits include self-esteem, positive affect, enjoyment, relaxation, energy, empowerment, confidence	Experiences including improved personal wellbeing, self-esteem, positive affect, enjoyment, relaxation, feeling energized, empowered and having confidence were captured in this meta-theme.	Irons
Creative arts: socioemotional connection brings sense of belonging	Papers highlighted the socio-emotional connection afforded by participation in weekly creative interventions, which gave both caregiver and care-recipient something to look forward to attending and be part of.	Irons
Creative arts: practicing skills gives cognitive stimulation and engagement	The cognitive impact of intervention participation included cognitive stimulation and engagement, through the practice of previously acquired skills (such as demonstrating musical knowledge).	Irons
Creative arts: reminiscence promoted	Creative interventions were useful for reactivating memories and afforded opportunities for memory recall of events.	Irons
Creative arts: dyads working together feel hopeful	In an intervention where caregivers and care-recipients met to create a database, this evoked positive feelings and hope for the caregivers.	Irons
Creative arts: carers reclaim identity through creative interventions	Creative interventions that allow for self-expression offer opportunities to caregivers to reclaim and transform their identity.	Irons
Creative arts: song hearing fosters emotions	In some cases, the meaning behind song lyrics evolved with the carer journey, with the song resonating and created an emotional response upon hearing the song.	Irons

Creative arts: creative interventions help for self-expression and emotional release	Participation in creative interventions allowed participants to have new experiences and to develop confidence to express oneself. For example, as one participant stated: “It probably unloads a lot of the...all the emotional feelings that you do hang on to that you don’t even realise are there sometimes so it helps to get them out...it was good and it was cathartic. Even though I say I don’t hold a lot you probably do have a bit of baggage and just talking about it and bringing out all those different words was, good and just very satisfying.”	Irons
Creative arts: good for mindfulness	Three papers reported the recognition some caregivers had on the importance of mindfulness and living in the moment, and that these interventions created the space for this to be experienced.	Irons
Creative arts: singing decreases distress	Some papers reported that caregivers found singing allowed them to focus and concentrate on the task of singing and develop present moment awareness, allowing them to let go of other distressing or negative thoughts.	Irons
Creative arts: more clarity in thoughts and rational thinking	Themes related to increased lucidity, where caregivers reported clarity of thoughts and increased rational thinking.	Irons
Creative arts: singing benefits include better mood and well-being	All six studies included comments on improved mood or well-being and three on the extended impact in terms of mood.	Bourne
Creative arts: interventions do not reduce negative emotions, but increase positive ones	Interestingly this meta-theme did not mention the reduction of negative emotions and instead highlighted the increase in positive ones. This suggests the negative feelings were not removed by participation in creative caregiving interventions; however, caregivers and care-recipients experienced positive changes as a result of participation in these creative interventions.	Irons
Creative arts: art gallery is empowering	The structure, ambience and environment of an intervention was key to shaping and facilitating participants’ experiences. For example, singing as part of a group was reported as an enabler for participation in interventions aimed at caregivers and care-recipients and an art gallery setting created an empowering space for participants	Irons
Creative arts: carers happy to see care receivers happy	The intervention activities were opportunities for playful experimentation, which was deemed the antithesis of caregiving, bringing about restorative feelings to caregivers and care-recipients. The caregivers experienced an increase in positive mood when they saw care-recipients expressing happiness due to participating in the intervention.	Irons

			Mindfulness: intervention is enjoyable	Qualitatively, participants felt positive about MBSR and enjoyed participating.	Li
			Mindfulness: benefits include compassion and forgiveness	Qualitatively, participants reported the continued use of mindful based exercise and personal benefits of fostered compassion and forgiveness from the program.	Li
**Intervention outcomes:** this category describes potential beneficial and/or harmful effects to the caregiver from participating in the interventions.	**Social outcomes:** intervention outcomes related to impact on caregiver's social life, day-to-day routine and relationship with care receiver and other people.	**Relational social outcomes:** changes in caregivers' relationship with either the care receiver or people around them.	Psychosocial: peer support leads to less isolation through mutual sharing	Qualitative social support outcomes demonstrated reduced feelings of social isolation, increased emotional support, and mutual sharing and enjoyment after receiving peer support.	Dam
			Respite: improved dyadic relationship	Carers reported increased engagement with their care recipients upon returning from adult day service attendance.	Du Preez
			Creative arts: dyadic facilitates reciprocity	As the group was for both partners of the dyad and reported to be accessible, this may have had a positive influence on relationships and communication, facilitating a sense of reciprocity.	Bourne
			Creative arts: singing brings more socializing	All six studies included comments on improved mood or well-being and three on the extended impact in terms of mood, the enjoyment of singing at home and socialising with other members outside of the group.	Bourne
			Creative arts: interventions give a feeling of equal dyadic relationships	Caregivers felt participation in the creative interventions allowed for interactions that were more equal and person-centred, as opposed to the usual caregiver–care-recipient dyad experiences that tended to occur outside the intervention.	Irons
			Creative arts: benefits of dyadic intervention: communication within dyads and others	As a result of participating in the intervention together, improved communication was reported by caregivers, not only within the dyads but also with other participants in the interventions and family members.	Irons
			Creative arts: interventions make dyads forget about the disease	Several papers described the opportunity for joint respite for the caregiver and care-recipient dyad to focus on the experience of the creative intervention, without needing to focus on the condition.	Irons
			Creative arts: dyad to dyad collaboration through singing	Furthermore, in a singing intervention, the social proximity of caregivers and their care-recipients to other caregivers and care-recipients were highly valued. The songs represented a shared experience, and this shared experience and collaboration was considered important.	Irons
			Creative arts: interventions make patients care recipients seem normal again	Participation in creative interventions allowed for caregivers to view the care-recipient in a different light, which led to increased satisfaction with caregiving role.	Irons

			Creative arts: better dyadic relationship	The process of creating a 'time capsule' database of music and photos from couples' lives evoked strong positive feelings and led to improved quality time for caregivers and their care-recipients.	Irons
			Creative arts: improved image of care receivers	The demonstration of memory and recall over the weeks was encouraging for caregivers to see with care-recipients.	Irons
			Creative arts: better relationship based on a new common interest	Meaningful connections made with care-recipient through the creation of art allowed caregivers to build social connections that were based on mutual interests rather than the caregiving.	Irons
			Creative arts: dyadic better for spousal relationships	Additionally, papers reported other social benefits, including enhanced spousal relationship and strengthened reciprocity between caregiver and care-recipient.	Irons
**Implementation outcomes:** the effects of deliberate and purposive actions to implement the interventions.	**Acceptability**: degree to which an intervention is perceived to be agreeable.	**Trust, dignity and values:** the role of trust, respect, ethics and dignity in determining acceptability of the intervention.	Case management: carers trust and appreciate admiral nurses they know well	There was evidence that carers valued interacting with a professional that they knew well, and descriptions of Admiral Nurses included 'my anchor', 'life-saving' 'an angel' and 'worth her weight in gold'.	Bunn
			Counselling: carers want to have a reliable person by their side	Carers express their desire to know they have a reliable person by their side.	Lins
			Counselling: carers reluctant to unknown counsellors' advice	A carer also expressed reservations about the idea of getting good advice from an unknown person since the disease has a great variety of manifestations.	Lins
			Counselling: carers sensitive to judgement	Carers will consciously or unconsciously notice the emotional attitude of the counsellor, which can lead them to trust the counselling less and experience it as unhelpful.	Lins
			Counselling: counsellors should be familiar with cases and caregivers	Carers can appreciate the counsellor as a person and know that the counsellor is familiar with their situation and can understand the situations the carer describes.	Lins
			Counselling: thorough counselling determines trust from caregiver	At the same time, the level of thoroughness with which counsellors convey the content of the discussion has an impact on how reliable and trustworthy the carer experiences the counsellor.	Lins
			Counselling: knowing the counsellors builds rapport and is more effective	The descriptive theme of 'Knowing each other' on the other hand, might have a positive impact on the counselling, partly because when counselling is not anonymous it helps counsellors to manage the frustrations of the carers, and partly because it lays the groundwork for becoming 'Familiar and trusted', which is the relationship desired by the carers.	Lins

Counselling: service should be non-judgmental and appreciative	Non-judgmental/appreciative. This descriptive theme describes how the attitude of the counsellor needs to be non-judgemental towards the 'non-professional' care provided by the carers and needs to convey appreciation for their great efforts.	Lins
Counselling: building rapport with caregivers is important	Carers and counsellors agree on the importance of getting to know each other before counselling.	Lins
Respite: dignity of care receiver more important than symptom management	Phinney and Moody argue that community-based services that endeavor to minimize stigma and normalize attendee's experiences, rather than focus on symptom management, may attract greater utilization.	Du Preez
Respite: respect and dignity for care receiver important	Additionally, carers expect their care recipients to be treated with care, respect, and dignity while attending an adult day service.	Du Preez
Respite: separation promotes stigmatization	Whilst separation may lead to stigmatization, group inclusion for attendees with cognitive decline appeared to promote their disengagement from activities requiring cognitive demand.	Du Preez
Respite: privacy violation is problematic	Factors such as concern for privacy violation and cost of service provision diminishes use of services by carers.	Du Preez
Respite: self-sufficiency, privacy and independence determine dyad's resistance to use the service	Values of self-sufficiency, privacy and independence that were displayed by carers were also held by the care recipients themselves, which contributed to resistance to service use.	Shaw
Respite: staff don't respect care receiver	Other negative attitudes towards respite included the view that it is demeaning for the care recipient, with its connotations of babysitting. This was not helped by experiences of a perceived lack of respect for the care recipient from formal care staff.	Shaw
Respite: service is demeaning for care receiver	Other negative attitudes towards respite included the view that it is demeaning for the care recipient, with its connotations of babysitting.	Shaw
Respite: trust is important to accept sitters	Indeed, this was mentioned by carers in several of the studies – the trust that is required of people coming into the home in this capacity.	Shaw
Creative arts: staff values and perspectives important for participants' experiences	The values and perspectives of the staff and those involved in the delivery of interventions were also pertinent to participants' experience of the interventions.	Irons
Creative arts: non-judgmental and active listening environment is important	A non-judgemental group approach was considered important to enable caregivers to voice their experiences with willing listeners, to share joy and sadness and to feel valued by others.	Irons

Kirvalidze M, *et al. BMJ Open* 2023; 13:e068646. doi: 10.1136/bmjopen-2022-068646

**Implementation outcomes:** the effects of deliberate and purposive actions to implement the interventions.	**Acceptability**: degree to which an intervention is perceived to be agreeable.	**Person-centeredness:** personalization and flexibility of interventions promote acceptability of the intervention by the caregivers.	Case management: flexibility is valued	The importance of the relational aspect of the role including face-to face contact and opportunities to explain their needs and concerns in a time and manner of their preference were found to be integral to the person with dementia and carer's ability to adjust to change.	Goeman
			Case management: person-centered approach is welcome	A distinguishing characteristic of Admiral Nurses was their carer-centred approach and there was evidence that carers welcomed a service which focused on them.	Bunn
			Psychoeducation: flexibility in access is valued	Common elements of the interventions that were repeatedly highlighted and attributed to high satisfaction included: flexibility in access suiting carers' lifestyles and commitments.	Sin
			Psychoeducation: personalization of interventions is valued	Common elements of the interventions that were repeatedly highlighted and attributed to high satisfaction included: […] availability of self-tailored and -paced programme allowing for individualised information and support.	Sin
			Psychoeducation: individualized approach is preferred	The qualitative data supports the use of patient and carer information and support PCIS but suggests that there is not a one-size-fits-all approach which can be used, as every patient with dementia and their carers will have different needs, preferences and responses.	Miles
			Psychoeducation: personalized information highly valued	When information was individualized, it was considered by caregivers as one of the most useful functionalities of the intervention.	Hopwood
			Psychoeducation: carers don't appreciate generic information	[…] with caregivers expressing frustration when required to review information that did not meet their specific needs.	Hopwood
			Respite: carers' and care receivers' needs-based service is more acceptable	For respite to be acceptable it must respond to both the carer's and the care recipient's needs.	Shaw
			Respite: care receiver's happiness, health and safety cause concern	Beisecker et al. reports that 'perceptions about patient unhappiness, safety, physical health, functional levels and behaviour were viewed as barriers to ADC use'.	Shaw
			Respite: distress of care receivers causes concern	Care recipient distress was a commonly reported impact, which frequently took some time to recover from after return home.	Shaw
			Respite: quality of care of care receivers is essential	A barrier to the uptake of respite services was a concern about the quality of care provided and this concern was most notable in relation to nursing homes, although home care was also sometimes problematic.	Shaw

			Respite: care receiver's cooperation is important to participate	Gaining the care recipient's co-operation was one of the major hassles involved, which was reported particularly in relation to day care.	Shaw
			Creative arts: dyadic intervention allows for person-centered interaction	Caregivers felt participation in the creative interventions allowed for interactions that were more equal and person-centred, as opposed to the usual caregiver–care-recipient dyad experiences that tended to occur outside the intervention.	Irons
			Creative arts: flexibility is key	One key recommendation was to engage the assistance of a qualified music therapist, who is trained in adapting an intervention according to individual needs and preferences, including addressing behavioural challenge, thus affording flexibility in structure which was seen as key to caregiver satisfaction and continued participation in the intervention.	Irons
			Creative arts: intervention should be tailored to abilities	One caregiver stated their views about their perceptions of their partner with dementia who also participated in the intervention: ". . .she loves the looking. One of the things she mentioned a number of times is how important it is, the silence at the beginning, where they really get a chance to look. And I think that for people with. . . slow processing skills, not poor but slow, that element is just so important."	Irons
			Creative arts: intervention should be tailored to care receiver's abilities	In one study, participants were critical of the intervention protocol, as some caregivers found it difficult to engage the care-recipient or experienced frustration with the care-recipients' lack of focus, suggesting there were lessons to be learned for future enhancements of the intervention.	Irons
**Implementation outcomes:** the effects of deliberate and purposive actions to implement the interventions.	**Acceptability**: degree to which an intervention is perceived to be agreeable.	**Culture and language:** ethnic, cultural and religious values influence acceptability of the intervention.	Case management: multicultural staff better at supporting diverse communities	A shared approach to care was found to be vital in decreasing burden among family members and that due to their close relationship and knowledge of families, multicultural workers can offer an important perspective that is invaluable in informing the provision of carer education and support within CALD communities.	Goeman
			Counselling: native language is preferred	Receiving counselling in the native language was also shown to contribute to building trust.	Lins
			Counselling: language can be a barrier in telephone counselling	Telephone counselling of carers who do not speak the national language is problematic.	Lins
			Respite: minorities prefer sitters of same ethnicity and gender	In this context this referred to ensuring that carers were of the same ethnic group, spoke the same language and were preferably of the same gender as the care recipient.	Shaw

			Respite: culture is important in determining demand for respite	Personal and cultural attitudes to the caring role and to the function and purpose of respite services may impact on the carers' perceptions of their needs for respite.	Shaw
			Respite: respect for culture and religious identity valued	Other important considerations for carers were that food was appropriate for their religion, for example vegetarian, and that the service was sensitive to other cultural and religious differences.	Shaw
**Implementation outcomes:** the effects of deliberate and purposive actions to implement the interventions.	**Acceptability**: degree to which an intervention is perceived to be agreeable.	**Setting of the intervention:** environmental, social and structural components of the interventions that promote or hinder acceptability.	Psychoeducation: rapport with professionals and peers harder online	In a small number of studies, despite extensive recruitment efforts and provision of equipment and technical support, recruitment and completion rates still struggled as some carers reported finding it difficult to strike up a rapport with the professionals and their carer-peers and would still prefer the conventional delivery media using face to face group or individual meetings.	Sin
			Psychoeducation: support from carer networks and professionals is valued	Common elements of the interventions that were repeatedly highlighted and attributed to high satisfaction included: […] and network support through online forums with other carers and access to professionals.	Sin
			Psychoeducation: interaction among participants is important	Negative from one study: lack of interaction with other participants.	Boots
			Respite: in home care preferred over day care	Because of difficulties with day care many carers in the studies reported a preference for in-home care as being less disruptive for the care recipient.	Shaw
			Respite: turnover of staff is disruptive	One other major area of difficulty was related to the lack of continuity of care and the high turnover of staff in support services.	Shaw
			Respite: caregivers not informed about activities at respite	Family carers have little to no contact with the adult day service other than to ready their care recipient for the day's attendance and have little knowledge of how their care recipient spends their time while attending adult day service.	Du Preez
			Creative arts: carers appreciate having a special place	One paper found that caregivers in an intervention considered themselves ordinary users of a community place that was "somewhere different" and valued as a special place.	Irons
			Creative arts: group singing is better	The structure, ambience and environment of an intervention was key to shaping and facilitating participants' experiences. For example, singing as part of a group was reported as an enabler for participation in interventions aimed at caregivers and care-recipients.	Irons
			Creative arts: creative interventions offer interactive space	Papers presented themes around caregivers' and care-recipients' appreciation of a time and space afforded by the creative interventions to enable greater communication, and a natural	Irons

Kirvalidze M, *et al. BMJ Open* 2023; 13:e068646. doi: 10.1136/bmjopen-2022-068646

				development of social interactions both with the caregiver and other participants in the interventions.	
			Creative arts: gallery setting and facilitator important for art viewing	Two papers highlighted the importance of the gallery setting and facilitator characteristics.	Bourne
**Implementation outcomes:** the effects of deliberate and purposive actions to implement the interventions.	**Acceptability**: degree to which an intervention is perceived to be agreeable.	**Other:** other determinants of acceptability, not groupable.	Psychoeducation: eHealth highly acceptable	In general, carers' perceived acceptability of the eHealth interventions across the studies synthesized was high.	Sin
			Psychoeducation: relatable experiences important	Experiential similarity is seen as important	Smith
			Creative arts: caregivers skeptical about their contributions to the interventions	Prior to joining the interventions, caregivers had deliberations around what to expect, including assumptions that they had nothing to contribute to the intervention.	Irons
			Mindfulness: mindfulness intervention is acceptable	Total of 31 FCs were included in the trial, overall, findings confirmed the feasibility and acceptability of the intervention for FCs, and the interviews, revealed that the intervention was associated with several participant-identified benefits and no severe adverse effects.	Aldaken
**Implementation outcomes:** the effects of deliberate and purposive actions to implement the interventions.	**Feasibility:** the extent to which a new intervention can be successfully used or carried out within a given setting. Typically invoked retrospectively as a potential explanation of an intervention's success or failure, as reflected in poor recruitment, retention, or participation rates.	**Recruitment:** factors related to raising awareness about an intervention and recruiting suitable participants.	Case management: lack of clarity on service content	Carers' lack of clarity about the Admiral Nurse role was also observed; 'never really found out what the Admiral Nurse service was offering'.	Bunn
			Psychoeducation: recruitment issues in tech interventions	In a small number of studies, despite extensive recruitment efforts and provision of equipment and technical support, recruitment and completion rates still struggled.	Sin
			Respite: advise to use comes from primary care	The most accessible location for advice is the primary health-care centre.	Shaw
			Respite: lack of information on available services	Not only was there evidence of limited access to respite services but also there was a low awareness of the availability of services.	Shaw
			Respite: GPs not effective providing support despite being most accessible	The most accessible location for advice is the primary health-care centre, but it was suggested by more than one study that GPs do not appear to be providing appropriate support and information.	Shaw
			Respite: awareness on services low in minorities	Not only was there evidence of limited access to respite services but also there was a low awareness of the availability of services.	Shaw
			Respite: medical staff not informed about services	Medical practitioners were identified as having limited knowledge of community support services and access to information resulting in poor referral processes and therefore, poor utilization by family carers and people living with dementia.	Du Preez

**Implementation outcomes:** the effects of deliberate and purposive actions to implement the interventions.	**Feasibility:** the extent to which a new intervention can be successfully used or carried out within a given setting. Typically invoked retrospectively as a potential explanation of an intervention's success or failure, as reflected in poor recruitment, retention, or participation rates.	**Accessibility:** physical, time-related or other external factors that limit or facilitate access to the interventions.	Case management: limiting admissions to confirmed dementia cases only	The failure factors were: […] and not including patients without a confirmed diagnosis of dementia.	Goeman
			Psychoeducation: access and time may be of concern for tech interventions	A few studies reported difficulties in even recruiting and retaining carers due to obstacles of access, cost, and time regarding use of technology.	Sin
			Counselling: telephone counselling is accessible	Carers find the telephone a good tool for receiving counselling as it helps avoid the stress involved in co-ordinating an appointment.	Lins
			Respite: transport may be an issue	There were a number of reports of complaints concerning transport, which included lack of transport in rural areas and a general unreliability of transport services.	Shaw
			Respite: waiting lists problematic	Waiting lists proved to be an issue for nursing home care and night care.	Shaw
			Respite: transport may be an issue	Transport was reported to be a significant barrier to utilization for carers. Use of public transport to access adult day service as opposed to transport being provided was found to be difficult and time-consuming.	Du Preez
			Creative arts: dyadic art interventions are accessible	As the group was for both partners of the dyad and reported to be accessible, this may have had a positive influence on relationships and communication, facilitating a sense of reciprocity.	Bourne
			Creative arts: singing is accessible for all	Caregivers highlighted other parts of the singing group experience to be important. These included the accessibility of singing for all.	Bourne
			Creative arts: art-viewing is accessible	Reported benefits included new learning, stimulation, and the accessibility of the activities, which creates a sense of normalcy and altered perceptions about PWD's abilities.	Bourne
**Implementation outcomes:** the effects of deliberate and purposive actions to implement the interventions.	**Feasibility:** the extent to which a new intervention can be successfully used or carried out within a given setting. Typically invoked retrospectively as a potential explanation of an intervention's success or failure, as reflected in poor recruitment, retention, or participation rates.	**Service availability:** factors related to coverage of services in terms of offered times and availability of staff.	Case management: access easy, but contact difficult outside working hours	There was evidence that making and maintaining contact with the service initially was found to be easy in the majority of cases, but only just over half of the respondents knew who to contact outside Admiral Nurse's hours.	Bunn
			Counselling: 24/7 service is a must	Carers agreed that 24-hour availability of telephone counselling is necessary.	Lins
			Counselling: answering machines insufficient	Existing arrangements, such as answering machines for the times when counselling is not available, were not sufficient.	Lins
			Counselling: telephone counselling is flexible	It is a very comfortable means of counselling because carers do not have to leave their homes and its flexibility allows sessions to be fitted into their daily nursing routines.	Lins

Kirvalidze M, *et al. BMJ Open* 2023; 13:e068646. doi: 10.1136/bmjopen-2022-068646

			Respite: flexibility of day care is key	flexibility of the DC in relation to available days and times, and the possibility of a regular or intermittent frequency.	Maffioletti
			Respite: timing and flexibility can be problematic	A major barrier to the uptake of respite services was a lack of response to carer needs in terms of timing and flexibility of service provision.	Shaw
			Respite: working carers not accommodated schedule-wise	In another study day care was only offered for 3 days a week and finished at 3pm, which did not accommodate the needs of working carers.	Shaw
			Respite: weekends and evenings not covered	Problems with day care were the lack of weekend and evening provision.	Shaw
			Creative arts: different preferences on length of intervention	Some participants shared disappointment that the intervention was time-limited, whilst others said that it would be difficult to continue the sessions long-term due to care-giving responsibilities.	Bourne
			Mindfulness: carers appreciate informality and availability of service	Chronic conditions: Qualitative results indicated that the informal practice was particularly helpful and could be used at any time.	Li
			Mindfulness: dyadic mindfulness is more practical to attend	Also, it might be more feasible for caregivers because they would not have to leave care recipients alone.	Li
**Implementation outcomes:** the effects of deliberate and purposive actions to implement the interventions.	**Feasibility:** the extent to which a new intervention can be successfully used or carried out within a given setting. Typically invoked retrospectively as a potential explanation of an intervention's success or failure, as reflected in poor recruitment, retention, or participation rates.	**Adoption and retention:** factors related to continued use of services or drop-out from services.	Psychoeducation: technical issues may arise	Usability problems (such as oral communication/chat quality, audio-visual function failure) were also identified as attributing to high drop-out rates (up to 50%) in some studies.	Sin
			Psychoeducation: older spousal carers find technology challenging	Most studies included focused on carers of dementia patients, and this was also the area with the most frequently reported problems in access and usability, as encountered by a group of largely elderly spousal carers who were often not familiar with ICT.	Sin
			Psychoeducation: dropout due to severe patients and carer time	Reasons for participant withdrawal included ill health of the person cared for and carer constraints.	Smith
			Psychoeducation: older adults find technology challenging	There appears to still be a gap between those who use or can use the internet and those who don't, with a study in 2015 highlighting that almost all adults over 70 years of age had difficulty using the intervention.	Hopwood
			Respite: carers need to prepare patients for respite	Carers reported having difficulty assisting their care recipient with their activities of daily living in preparation for the day's attendance.	Du Preez

			Respite: multicomponent interventions increase utilization	Adult day services that offer comprehensive services that engage dementia caregivers by way of phone calls or one-on-one carer meetings to address their areas of concern, invite carer collaboration in planning meetings, provide carer education, counseling and case management were seen to facilitate increased service utilization and delay early institutionalization.	Du Preez
			Respite: severe patients difficult to prepare and reluctant to participate	Carers reported having difficulty assisting their care recipient with their activities of daily living in preparation for the day's attendance. This was exacerbated by their care recipient's co-morbidities and resistance to attendance.	Du Preez
			Respite: decline in function leads to exclusion from service	Another barrier to use is attendance being conditional upon attendees being able to independently self-care.	Du Preez
			Respite: person centered activities increase utilization	Adult day services' ability or inability to offer a variety of activities that were person-centered and held meaning for attendees was a motivating factor for use.	Du Preez
			Respite: services minimizing stigma leads to greater utilization	Whilst separation may lead to stigmatization, group inclusion for attendees with cognitive decline appeared to promote their disengagement from activities requiring cognitive demand. However, Gaugler found staff overcame these issues through validation and one-to-one interaction.	Du Preez
			Respite: timing and flexibility may be barriers	A major barrier to the uptake of respite services was a lack of response to carer needs in terms of timing and flexibility of service provision.	Shaw
			Respite: severe PwD excluded	With confusion comes inappropriate behaviours, which were difficult for both in-home and institutional respite carers to accommodate, resulting in exclusion of the care recipient from the service.	Shaw
			Respite: service is used for maintaining nutrition	As the care recipient's condition deteriorates carers express concern for safety and the need for a sitter may be expressed. There are also concerns about maintaining care recipient health in relation to maintaining adequate nutrition.	Shaw
			Respite: progression of disease triggers use of service	As the care recipient's condition deteriorates carers express concern for safety and the need for a sitter may be expressed.	Shaw
**Implementation outcomes:** the effects of deliberate and purposive actions to implement the interventions.	**Feasibility:** the extent to which a new intervention can be successfully used or carried out within a given setting. Typically invoked retrospectively as a potential	**Systemic factors:** Larger structural factors related to the organization of health and social care services.	Case management: primary care not involved is hindering	The failure factors were: […] little or no involvement of primary care specialists.	Goeman
			Case management: competition to deliver care is a barrier	The failure factors were: […] competition for delivering care.	Goeman

	explanation of an intervention's success or failure, as reflected in poor recruitment, retention, or participation rates.		Case management: medical staff not collaborating with admiral nurses	At times, carers felt that GPs, specialists and care managers did not work with or communicate with Admiral Nurses; 'Care manager doesn't seem to communicate with Admiral Nurse'.	Bunn
			Education/skills: carers appreciate having one source of information	Caregivers emphasised the importance of having one central source for information, for example a key worker to liaise with during visits.	Pritchard
			Respite: systems do not respond to need	Additionally, imperatives of service delivery systems place carers in a vulnerable position seemingly prioritizing these systems above the needs of caregivers. Utilization of services is often determined by effective referral by health professionals.	Du Preez
			Respite: bureaucracy unavoidable and intruding upon care receiver's privacy	Furthermore, interaction with adult day service providers requires carers to adopt institutional processes and intrudes upon their care recipient's privacy, albeit it to promote that person's quality of life.	Du Preez
**Implementation outcomes:** the effects of deliberate and purposive actions to implement the interventions.	**Feasibility:** the extent to which a new intervention can be successfully used or carried out within a given setting. Typically invoked retrospectively as a potential explanation of an intervention's success or failure, as reflected in poor recruitment, retention, or participation rates.	**Other:** other determinants of feasibility, not groupable.	Mindfulness: mindfulness intervention is feasible	Total of 31 FCs were included in the trial, overall, findings confirmed the feasibility and acceptability of the intervention for FCs, and the interviews, revealed that the intervention was associated with several participant-identified benefits and no severe adverse effects.	Aldaken
**Implementation outcomes:** the effects of deliberate and purposive actions to implement the interventions.	**Appropriateness:** perceived suitability and usefulness of intervention to address needs of caregivers.	**N.A.**	Case management: some needs are overlooked	Case management offered potential benefit to people with dementia, their carers and community-based professionals through continuity of care by a named trusted individual that could act proactively to prevent a crisis. However, it was also shown that needs may be overlooked.	Goeman
			Case management: multidisciplinary and continued services meet more needs	The specialist multi-agency home support service demonstrated greater flexibility and responsiveness to the particular needs and circumstances of service users and family carers, who were encouraged to participate in routine decision-making and activities. By sharing responsibilities, the specialist service helped reduce carer stress and prevent crises. These outcomes depended on the configuration of the service, including multidisciplinary health and social services input, care worker autonomy and independence, continuous reassessment of clients' circumstances and preferences and the capacity to develop long-term relationships, through care worker continuity. The standard service, which used a task orientated approach, lacked these characteristics.	Goeman

	Case management: integration of care is necessary	It was found that the success for case management in long-term dementia care concern the expert knowledge of case managers; investment in a strong provider network and coherent conditions for effective inter-organisational cooperation to deliver integrated care.	Goeman
	Case management: patient-centeredness is appropriate	It is suggested that the appropriate way of offering case management is through a patient-centred approach.	Goeman
	Case management: admiral nurse as a liaison among care providers	A commonly reported component of the Admiral Nurse role was facilitation, which included liaison with other health and social care services; 'She makes sure she liaises with all the agencies concerned with mum's care'.	Bunn
	Psychoeducation: Internet based support not for all	Qualitative evaluations of the interventions demonstrated positive views from most caregivers toward internet-based support interventions, although it is clear that not all would benefit from such interventions.	Hopwood
	Psychoeducation: carers don't appreciate general information	[…] with caregivers expressing frustration when required to review information that did not meet their specific needs.	Hopwood
	Psychoeducation: interaction with professionals more beneficial than just information	However, other multiple-component interventions evaluated with qualitative methods found that caregivers found other components, such as interaction with professionals, more beneficial than information.	Hopwood
	Education/skills: Face-to-face and repeated information preferred	Appropriate modality and timing of information means the person may require information to be presented in different ways (e.g., in writing, diagrams) repeated on several occasions and in person, not over the phone.	Pritchard
	Education/skills: differences between carers should be considered	The interaction between the personal characteristics of both the patient and caregiver is not always considered by health professionals. However, because the skills of the caregiver can impact the patient and vice versa, considering the interaction is paramount. Personal characteristics is also influenced by and influences organisational culture and the level of information provision.	Pritchard
	Education/skills: carers' needs addressed related to information, coping mechanisms, assessment of home environment	The interventions addressed the need for clear information about the disease, identification of psychological response, development of coping mechanisms and assessment of the home environment.	Pritchard
	Counselling: counsellors inform carers about other suitable services	Together with the carers, counsellors identify the most suitable services, which helps the carers to understand that they have access to an extensive help system in addition to telephone counselling.	Lins

Kirvalidze M, *et al. BMJ Open* 2023; 13:e068646. doi: 10.1136/bmjopen-2022-068646

			Counselling: more severe cases need more counselling	Counselling for carers is most needed, for example, in the case of an accident or during the final phase of the disease.	Lins
			Counselling: service needed to face potentially escalating crises	Carers need assistance most acutely in difficult situations when they feel insecure and see that an escalation is possible.	Lins
			Respite: respite seen as more appropriate for severe patients	Carers of recipients with dementia felt that day care, for example, was more appropriate for more severely demented people who were unaware of their surroundings.	Shaw
			Creative arts: group art not always beneficial	Two studies reported neutral or negative comments regarding participation in the group. These included some caregivers reporting initial apprehensiveness about attending, no effect on themselves but effects on the PWD.	Bourne
			Creative arts: carers cannot relax due to worry about PwD	These included some caregivers reporting initial apprehensiveness about attending, no effect on themselves but effects on the PWD, and difficulties with relaxation due to concerns about the PWD.	Bourne
			Creative arts: song writing addresses needs not met by other groups	The song writing group filled a gap for caregivers that were not met by other support groups.	Irons
			Creative arts: members of dyads don't have same needs	It may have been difficult to structure and time the delivery of an intervention to appeal to both caregiver and care-recipient, as illustrated by this quote: ''I think it drew him out more than it drew me out. And why that is, I don't know."	Irons
			Mindfulness: not all relaxing or benefitting	Although, these potential benefits were not universal, and some participants did report difficulty fully relaxing during the process.	Parkinson
**Implementation outcomes:** the effects of deliberate and purposive actions to implement the interventions.	**Sustainability:** factors associated with sustained, long-term use of the intervention.	**N.A.**	Psychoeducation: shared experiences in peer support leads to sustainability	Carers were more likely to have successful peer support relationships and to continue meeting after the intervention ended, if they were similar on the shared experience of caring.	Smith
			Respite: experience of benefit and opportunistic use leads to regular use	The need for respite for particular social events or occasions such as conferences, weddings and holidays triggered the use of respite, and once the benefit was experienced and proved successful it was used again on a more regular basis.	Shaw
			Mindfulness: mindfulness interventions sustained by carers	Qualitatively, participants reported the continued use of mindful based exercise and personal benefits of fostered compassion and forgiveness from the program.	Li
**Implementation outcomes:** the effects of deliberate and purposive actions to implement the interventions.	**Cost:** financial costs associated with implementing or using the intervention.	**N.A.**	Case management: funding is an issue	The failure factors were: […] inadequate or no structural funding.	Goeman

Kirvalidze M, *et al. BMJ Open* 2023; 13:e068646. doi: 10.1136/bmjopen-2022-068646

	Psychoeducation: cost is an issue	A few studies reported difficulties in even recruiting and retaining carers due to obstacles of access, cost, and time regarding use of technology.	Sin
	Respite: cost is an issue	Because of the different organizational processes involved in the different countries, cost issues may vary.	Shaw
	Respite: cost is an issue	Factors such as concern for privacy violation and cost of service provision diminishes use of services by carers. […] Affordability is an issue for some carers who constantly weigh their care recipients needs against the economic burden of meeting those need.	Du Preez

Abbreviations: QoL, Quality of Life; MBIs, Mindfulness-Based Interventions; LTCs, Long Term Conditions; PwD, Person with Dementia; MBSR, Mindfulness-Based Stress Reduction; PCIS, Patient and Carer Information and Support; CALD, Culturally And Linguistically Diverse; FC, Family Caregiver; DC, Day Care; ICT, Information and Communication Technology
