# Supplementary material for: Effectiveness of interventions designed to mitigate the negative health outcomes of informal caregiving to older adults: an umbrella review of systematic reviews and meta-analyses
Source: BMJ Open. 2023 Apr 21;13(4):e068646. doi: 10.1136/bmjopen-2022-068646 (PMC10124259; doi:10.1136/bmjopen-2022-068646)
Supplement: Author's manuscript [file bmjopen-2022-068646.draft_revisions.pdf]

BMJ Open is committed to open peer review. As part of this commitment we make the peer review history of every article we publish publicly available.

When an article is published we post the peer reviewers' comments and the authors' responses online. We also post the versions of the paper that were used during peer review. These are the versions that the peer review comments apply to.

The versions of the paper that follow are the versions that were submitted during the peer review process. They are not the versions of record or the final published versions. They should not be cited or distributed as the published version of this manuscript.

BMJ Open is an open access journal and the full, final, typeset and author-corrected version of record of the manuscript is available on our site with no access controls, subscription charges or pay-per-view fees (http://bmjopen.bmj.com).

If you have any questions on BMJ Open's open peer review process please email info.bmjopen@bmj.com

BMJ Open

Effectiveness of interventions designed to mitigate the negative health outcomes of informal caregiving to older adults: an umbrella review of systematic reviews and meta-analyses

Journal:	*BMJ Open*
Manuscript ID	bmjopen-2022-068646
Article Type:	Original research
Date Submitted by the Author:	29-Sep-2022
Complete List of Authors:	Kirvalidze, Mariam; Karolinska Institute Department of Neurobiology Care Sciences and Society, Aging Research Centre<br>Abbadi, Ahmad; Aging Research Center, Department of Neurobiology, Care Sciences and Society, Karolinska Institutet and Stockholm University<br>Dahlberg, Lena; Aging Research Center, Department of Neurobiology, Care Sciences and Society, Karolinska Institutet and Stockholm University; School of Health and Welfare, Dalarna University<br>Sacco, Lawrence; Stress Research Institute, Department of Psychology, Stockholm University<br>Morin, Lucas; Department of Medical Epidemiology and Biostatistics, Karolinska Institutet; Inserm CIC 1431, University Hospital of Besançon<br>Calderón-Larrañaga, Amaia; Aging Research Center, Department of Neurobiology, Care Sciences and Society, Karolinska Institutet and Stockholm University; Stockholm Gerontology Research Center
Keywords:	PUBLIC HEALTH, QUALITATIVE RESEARCH, SOCIAL MEDICINE, STATISTICS & RESEARCH METHODS, Health policy < HEALTH SERVICES ADMINISTRATION & MANAGEMENT

SCHOLARONE™
Manuscripts

I, the Submitting Author has the right to grant and does grant on behalf of all authors of the Work (as defined in the below author licence), an exclusive licence and/or a non-exclusive licence for contributions from authors who are: i) UK Crown employees; ii) where BMJ has agreed a CC-BY licence shall apply, and/or iii) in accordance with the terms applicable for US Federal Government officers or employees acting as part of their official duties; on a worldwide, perpetual, irrevocable, royalty-free basis to BMJ Publishing Group Ltd ("BMJ") its licensees and where the relevant Journal is co-owned by BMJ to the co-owners of the Journal, to publish the Work in this journal and any other BMJ products and to exploit all rights, as set out in our licence.

The Submitting Author accepts and understands that any supply made under these terms is made by BMJ to the Submitting Author unless you are acting as an employee on behalf of your employer or a postgraduate student of an affiliated institution which is paying any applicable article publishing charge ("APC") for Open Access articles. Where the Submitting Author wishes to make the Work available on an Open Access basis (and intends to pay the relevant APC), the terms of reuse of such Open Access shall be governed by a Creative Commons licence – details of these licences and which Creative Commons licence will apply to this Work are set out in our licence referred to above.

Other than as permitted in any relevant BMJ Author's Self Archiving Policies, I confirm this Work has not been accepted for publication elsewhere, is not being considered for publication elsewhere and does not duplicate material already published. I confirm all authors consent to publication of this Work and authorise the granting of this licence.

For peer review only

TITLE

Effectiveness of interventions designed to mitigate the negative health outcomes of informal caregiving to older adults: an umbrella review of systematic reviews and meta-analyses

AUTHORS:

- Mariam Kirvalidze MPH[a] (mariam.kirvalidze@ki.se)
- Ahmad Abbadi MMScPH[a] (ahmad.abbadi@ki.se)
- Lena Dahlberg PhD[a,b] (ldh@du.se)
- Lawrence B. Sacco PhD[c] (lawrence.sacco@su.se)
- Lucas Morin PhD[d,e*] (lucas.morin@inserm.fr)
- Amaia Calderón-Larrañaga PhD[a,f*] (amaia.calderon.larranaga@ki.se)

AFFILIATIONS:

a) Aging Research Center, Department of Neurobiology, Care Sciences and Society, Karolinska Institutet and Stockholm University, Solna, Sweden
b) School of Health and Welfare, Dalarna University, Falun, Sweden
c) Stress Research Institute, Department of Psychology, Stockholm University, Stockholm, Sweden
d) Inserm CIC 1431, University Hospital of Besançon, Besançon, France
e) Department of Medical Epidemiology and Biostatistics, Karolinska Institutet, Solna, Sweden
f) Stockholm Gerontology Research Center, Stockholm, Sweden

*Lucas Morin and Amaia Calderón-Larrañaga share the last authorship

CORRESPONDING AUTHOR

Mariam Kirvalidze
Aging Research Center, Karolinska Institutet
Tomtebodavägen 18 A, 171 65 Solna, Sweden
Mariam.kirvalidze@ki.se

For peer review only

For peer review only - http://bmjopen.bmj.com/site/about/guidelines.xhtml

ABSTRACT

Objectives. Instead of bringing clarity, the proliferation of systematic reviews has fragmented our understanding of the effectiveness of interventions to support informal caregivers. In this umbrella review, we aimed to evaluate whether certain interventions can truly mitigate the negative health consequences of caregiving, which interventions are more effective than others depending on the circumstances, and how these interventions are experienced by caregivers themselves. We included quantitative, qualitative and mixed systematic reviews exploring the effectiveness of caregiver support interventions on health.

Participants. Reviews including primary studies targeting informal (i.e., unpaid) caregivers of older people or persons presenting with aging-related diseases were considered.

Interventions. Reviews focusing on support interventions and assessing either their effectiveness (for quantitative reviews) or their implementation and/or the lived experience of the target population (for qualitative reviews) were considered.

Outcome measures. Reviews including physical or mental health-related outcomes of informal caregivers, including health-related quality of life were considered.

Results. A total of 47 reviews were included, covering 619 distinct primary studies. Each potentially eligible review underwent rigorous critical appraisal and citation overlap assessment. Our synthesis showed that the evidence regarding the effectiveness of interventions on physical and mental health outcomes was inconclusive. Quantitative reviews were highly discordant, whereas qualitative reviews only reported practical, emotional, and relational benefits. Multicomponent and person-centered interventions seemed to yield highest effectiveness and acceptability. Heterogeneity among caregivers, care receivers and care contexts was often overlooked. We found important issues related to the quality of evidence and overproduction of similar reviews.

Conclusions. Lack of robust evidence calls for better intervention research and evaluation practices. Targeting interventions to high-risk groups may be warranted to avoid one-size-fits-all approaches to intervention design. Primary care and other existing resources should be leveraged to support interventions, possibly with increasing contributions from the non-profit sector.

Registration. *PROSPERO*: CRD42021252841**;** *BMJ Open*: doi:10.1136/bmjopen-2021-053117

For peer review only

STRENGTHS AND LIMITATIONS OF THIS STUDY

- The umbrella review methodology enabled us to perform high-level synthesis and describe the state of the evidence on the topic of interventions to mitigate the negative health consequences of informal caregiving.
- The review benefits from the mixed-methods approach, as we included both quantitative reviews on effectiveness and qualitative syntheses exploring complex aspects related to the experiences of caregivers.
- Synthesis is confined to a narrative output due to heterogeneity of our sources.
- More recent primary studies on new interventions were not captured, as they would not have been included in systematic reviews selected for this umbrella review.

For peer review only - http://bmjopen.bmj.com/site/about/guidelines.xhtml

INTRODUCTION

Informal caregivers are defined as any relative, partner, friend or neighbor who provides a broad range of assistance to an older person who lives with a chronic or disabling condition and with whom they have a significant personal relationship.[1] Their role has become increasingly important, as populations age and professional social care services struggle to meet the increasing care demands. Improved life expectancy leads to more years spent with late-life dependency,[2] and this burden often falls on the families of older adults. It is now estimated that informal caregivers contribute to the majority of care for persons aged over 50 in most European countries[3] and long-term care relies heavily on the availability of informal caregivers, especially in the current context of care worker shortages.[4] In the near future, some countries are expected to have 'care gaps' or, in other words, insufficient numbers of informal caregivers to meet the increasingly complex care needs of older adults.[5,6]

Informalization of care has come with important concerns about the potential side effects of caregiving, and with growing interest from decision makers and stakeholders in the effectiveness of existing support interventions to alleviate these negative consequences. The burden of informal care can be determined by various factors, such as the intensity of caregiving, care receiver's dependency level, relationship with the care receiver, available social support from the community, and caregiver's own health.[7–10] Research findings on the health effects of caregiving has been mixed, with some studies reporting a positive association with health and longevity[11] while others reveal a myriad of adverse repercussions, such as increased mortality,[12] poorer well-being,[13] and worse physical and mental health outcomes in general.[14] The socioeconomic status, age and gender of caregivers have also been found to moderate these adverse consequences: women, older people, and those with lower income bear the highest psychological and physical burden of informal caregiving.[15] There is now a broad consensus about the multifactorial nature of this burden and its complex ramifications in terms of health and well-being.[15,16]

There has been a considerable increase in the number of empirical studies (both qualitative and quantitative) on the topic of caregiver support interventions, followed—in recent years—by a large number of systematic reviews. Consequently, it has become challenging to keep a bird's-eye-view of this field of research. To provide decision makers and stakeholders with synthetic and actionable evidence, a logical next step is to conduct a review of systematic reviews, that is, an 'umbrella review'.[17] Umbrella reviews are designed to give a broad and high-level overview of the available evidence on a given topic by compiling existing reviews rather than aggregating findings from the individual studies included in these reviews. By stitching together reviews about different types of interventions, populations or conditions, they provide an opportunity to assess not only the content-related comprehensiveness of these reviews, but also the overall reliability of the available evidence. Although a handful of umbrella reviews or meta-reviews have already examined caregiver support interventions, their scopes have been limited to only one type of intervention and/or disease[18,19] and some were too broad or unsystematic to capture differences between caregiver groups.[20,21] Our understanding of the effectiveness of existing interventions for mitigating the harms of informal caregiving is currently hampered by the lack of up-to-date synthesis of the evidence focusing on more objective physical and mental health outcomes rather than perceived burden or subjective well-being. Moreover, there is a need for integrating quantitative findings about the effectiveness of interventions with qualitative findings on the lived experience of caregivers who received these interventions.

In particular, the following research questions were addressed: (1) Are there effective interventions to prevent and reduce the negative health consequences of informal caregiving? (2) Are certain types of interventions more effective than others? (3) Is there evidence that the effectiveness of interventions depends on caregiver, care receiver, care context and implementation characteristics? (4) How are the proposed interventions experienced by caregivers in terms of effectiveness and implementation outcomes?

For peer review only - http://bmjopen.bmj.com/site/about/guidelines.xhtml

METHODS

Protocol registration

The protocol for this umbrella review was registered *a priori* in the PROSPERO database[22] and published in a peer-reviewed journal (doi:10.1136/bmjopen-2021-053117).[23] **Supplementary Table 1** includes a list of all amendments made to the protocol after its registration, which will be mentioned in relevant sections below. The reporting of this umbrella review is based on the Preferred Reporting Items for Overviews of Reviews (PRIOR) statement.[24]

Eligibility criteria, information sources and search strategy

Inclusion and exclusion criteria used to select reviews are presented in **Box 1** and discussed in detail in the umbrella review protocol.[23] Due to the abundance of published systematic reviews, the eligibility criteria were amended to exclude reviews of critically low quality based on our risk-of-bias assessment. This amendment is documented in **Supplementary Table 1**. The Medline, CINAHL, PsycINFO and Web of Science databases were searched for the period of 1 January 2000 to 26 March 2021. In addition, we performed a manual search of the reference lists of included reviews. **Supplementary table 2** includes detailed search strategies and number of hits from all searched databases. Search strategies were developed by the review team in consultation with university librarians. Even though the first reviews on caregiver intervention research were published in the 1990s, we focused on reviews published since 2000 to capture studies conducted in the context of more current social settings. We also restricted the inclusion to reviews published in languages spoken by research team members: English, Swedish, Spanish, French, Italian and German. The titles and abstracts of all references as well as full texts of preliminarily selected reviews were screened against the eligibility criteria independently by two reviewers using the *Covidence*® software developed by the Cochrane collaboration.[25] Any dissent in abstract screening and/or full-text assessment were resolved by discussion moderated by a third reviewer.

For peer review only - http://bmjopen.bmj.com/site/about/guidelines.xhtml

Box 1. Inclusion and exclusion criteria for quantitative and qualitative reviews.

▸ **Inclusion criteria**

Publication type, date and language

- Reviews published in a peer-reviewed journal OR as a report done or mandated by an official health agency (*e.g., Health Technology Assessment agency*)
- Reviews published between January 1, 2000, and March 26, 2021
- Reviews published in English, Swedish, Spanish, French, Italian or German

Study design

- For quantitative reviews: reviews including a reproducible, systematic search strategy, AND clearly defined inclusion/exclusion criteria AND risk of bias assessment for all included primary studies
- For qualitative reviews: reviews including a reproducible, systematic search strategy, AND defined inclusion/exclusion criteria

Population

- Reviews concerning informal caregivers (*i.e., people who regularly provide unpaid care to a family member, friend or neighbor*) of older people OR of persons presenting with aging-related disease (*e.g., dementia, stroke, Parkinson's disease, cancer, heart failure, multimorbidity, frailty*)

Intervention

- Reviews focusing on interventions and assessing either their effectiveness (for quantitative reviews) or their implementation and/or the lived experience of the target population (for qualitative reviews)

Outcome

- Reviews including physical or mental health-related outcomes of informal caregivers, including health-related quality of life

▸ **Exclusion criteria**

Study quality and overlap

- For quantitative reviews: reviews of 'critically low' quality as per AMSTAR-2 assessment tool
- For qualitative reviews: reviews with two or more critical flaws as per *ad hoc* assessment tool*
- Review pairs with very high or high overlap (as per Corrected Covered Area method) were examined and older, less relevant, or lower quality reviews were excluded

Additional exclusion criteria

- Reviews of interventions focusing *exclusively* on care receivers as the target population
- Reviews focusing *exclusively* on interventions for caregivers of young populations
- Reviews measuring *exclusively* non-health related outcomes, such as caregiver burden, stress/strain, work or financial status, family relations, breakdown of informal care
- Reviews focusing *exclusively* on end-of-life care interventions

*See the published protocol (doi:10.1136/bmjopen-2021-053117) for the *ad hoc* assessment tool for qualitative reviews

Selection of reviews: risk-of-bias assessment and overlap assessment

Each quantitative review was critically appraised by two reviewers working independently, using the AMSTAR-2 checklist.[26] This checklist categorizes the quality of the reviews based on seven 'critical' and nine 'non-critical' domains. The team made a consensus-based decision do downgrade item #10 (*Did the review authors report on the sources of funding for the studies included in the review?*) from 'critical' to 'non-critical' since this information was only available in Cochrane reviews (amendment is documented in **Supplementary Table 1**). Based on our appraisal, the reviews were grouped into 'critically low', 'low', 'moderate', and high-quality categories, and critically low-quality reviews were excluded from the umbrella review. Qualitative reviews were also assessed independently by two team members, who used an *ad hoc* quality appraisal checklist adapted from the Joanna Briggs Institute Checklist for Systematic Reviews and Research Syntheses[17] and available in the published protocol[23] The tool was developed and piloted by all team members on a sample of five randomly selected reviews. Items #3, #4, #7 and #10 were considered as 'critical', with reviews exhibiting more than two critical flaws being excluded. The quality of mixed-methods reviews was assessed using the above-mentioned tools for their quantitative and qualitative components, respectively. Any dissent in the risk-of-bias assessment process was resolved through discussions moderated by a third investigator.

Reporting biases arising from primary studies included in the systematic reviews were assessed using the AMSTAR-2 tool[26] which includes critical items related, among other aspects, to selective reporting of outcomes and publication bias. We were more lenient with qualitative reviews (or parts of reviews related to qualitative data) because risk-of-bias assessment is less common and structured in qualitative research. We did, however, include items related to the quality of primary studies in our *ad hoc* tool for qualitative reviews.

The degree of overlap of primary studies included in the reviews (namely, the fraction of evidence synthesized in two or more reviews) was estimated using the Corrected Covered Area (CCA) methodology[27] and the Graphical Representation of Overlap for OVErviews (GROOVE) open-access tool.[28] We built separate citation matrices for quantitative and qualitative primary studies and we accounted for structural missingness based on publication date (i.e., primary papers published after the review was completed were not marked as missing from the reviews). Guided by the methodology developed by Pollock et al.[29] two team members went through the pairs with "very high (≥15%)" and "high (10-15%)" overlap, and decisions on inclusion were made based on relevance, search dates, potential contribution to the umbrella review, and quality of the publication (described in **Supplementary Table 3**).

Data extraction

Data from included reviews were extracted by two team members in structured spreadsheets designed and validated *a priori* by all the investigators involved in this umbrella review. The quality and validity of the extracted data was assessed through regular cross-checks. For quantitative reviews, we extracted the following information: review objectives, methodological aspects (inclusion/exclusion criteria, search dates and databases, synthesis methods), target population and disease, characteristics of the interventions of interest, sociodemographic background of caregivers, health outcomes, degree of effectiveness of interventions (with/without meta-analysis), implications for practice and research, as well as the full list of primary studies included in the review. The latter was extracted for the purpose of overlap assessment. Qualitative reviews were characterized in terms of methodology, and we extracted all the *verbatim* text related to the reported caregivers' experiences of interventions.

Synthesis methods

For quantitative reviews, we conducted a narrative synthesis and provided findings in a tabular format, organized by intervention type. To provide a data-driven list of intervention types, we adapted Gaugler et al.'s[30] original typology into the following classification: case management, psychosocial and education/skills building, respite care, relaxation

For peer review only - http://bmjopen.bmj.com/site/about/guidelines.xhtml

and leisure, and mindfulness. If reviews compared different types of interventions, the outcome of this comparison was reported. Factors related to the caregiver, care receiver and/or care context affecting the effectiveness of interventions were identified and reported whenever possible.

For qualitative reviews, we followed the 'mega-aggregation framework' synthesis method[31] **Supplementary Table 1** documents post-protocol amendments related to synthesis of qualitative reviews, while **Supplementary Table 4** describes steps involved in the mega-aggregation process. First, the extracted *verbatim* texts from reviews were coded line-by-line (complete coding) by two team members. Codes were then iteratively and deductively categorized based on the adapted version of van Houtven et al.'s framework[32] Throughout the review, we refer to verbatim texts extracted from reviews and their corresponding codes as third-order constructs, while primary studies inform second-order constructs, and the communication from participants (i.e., caregivers) is considered as a first-order construct. Thus, the themes, categories, and sub-categories that we generated based on third-order constructs (i.e., reviews) are termed as fourth-order constructs[31] In case our fourth-order codes did not fit fully into the framework, they were inductively categorized into new themes. Codes referring to care receiver or staff opinions were removed. A third investigator checked the outcome of the categorization independently, and several consensus meetings were held to resolve disagreements and finalize the synthesis of qualitative materials.

As a complementary output, we used parallel convergence approach (i.e., synthesizing quantitative and qualitative evidence separately and bringing them together at the final stage) to update our initial conceptual framework that was based exclusively on expert opinion and/or existing literature (see the published protocol[23]). The resulting framework, *From Support Interventions to Improved Caregiver Outcomes (SIICO)*, will be presented below and aims to substantiate the potential pathway (and its various modifiers and mediators) linking caregiver support interventions to improved health outcomes.

Patient and public involvement (PPI)

No patients or members of the public were involved in the development of this umbrella review. However, the scope and methods of this review were informed by the literature and discussions with experts in the field.

For peer review only - http://bmjopen.bmj.com/site/about/guidelines.xhtml

RESULTS

Selection of reviews: risk-of-bias assessment and overlap assessment

Our search strategy resulted in a total of 6209 unique records, of which 5906 were excluded at the stage of title and abstract screening (**Figure 1**). From the 303 reviews that underwent full-text screening, 158 were excluded and 145 were further assessed for risk of bias. We excluded 92 quantitative and 9 qualitative reviews considered as being of 'critically low' quality. Results of the risk-of-bias assessment for all potentially eligible reviews are reported in **Supplementary Tables 5a and 5b**.

Of the 51 remaining quantitative reviews, 32 fell under the 'low quality' category, 13 were classified as being of 'moderate quality' and 6 were rated as high-quality reviews. Overall, the most common issues were lack of pre-registered protocol, absence of a full list of excluded studies with a rationale for the exclusion of each study, lack of reporting of sources of funding for primary studies, methodological issues related to meta-analyses, and suboptimal assessment of heterogeneity and publication bias. All 18 qualitative reviews eligible at this stage had only one or no critical flaw. Most common pitfalls included: lack of clarity regarding participation of researchers in synthesis process, lack of justification of qualitative synthesis methods, and lack of information on data extraction procedures.

After risk-of-bias assessment, reviews were examined for primary study overlap. Full citation matrices (accounting for structural missingness based on publication date) and resulting pyramids are available in **Supplementary Files 1 and 2**. Among the 18 qualitative and mixed-methods reviews assessed for overlap, we did not exclude any reviews since the degree of overlap was low. However, out of the 51 eligible quantitative reviews, we examined 33 pairs that reported 'very high' level of overlap (≥15%) and further checked 40 pairs of reviews that reported 'high' level of overlap (10–15%). Upon rigorous assessment of overlap, 14 quantitative reviews were finally excluded (see **Supplementary Table 3** for detailed description of the overlap decisions).

Finally, 47 reviews covering 619 primary studies were included in the umbrella review. Of these, 10 contributed to qualitative data only, 8 were included as both qualitative and quantitative data sources, while 29 reviews contributed exclusively to quantitative synthesis.

Characteristics of the included systematic reviews

All included reviews were published in English. Publication dates spanned from 2009 to 2021. Most primary studies were conducted in North America or Western Europe. Reviews set various inclusion and exclusion criteria regarding the design of primary studies, with some reviews focusing on randomized controlled trials, while others casted a wider net including quasi-experimental and qualitative studies. Among quantitative reviews, 18 undertook a meta-analysis while 19 undertook a data synthesis without meta-analysis. In qualitative reviews (or qualitative parts of mixed reviews), the most common synthesis approach was narrative (14 reviews), but four reviews reported a qualitative meta-synthesis. Characteristics of all included reviews, such as search dates, number of studies, demographics of included populations and types of interventions examined are summarized and presented in **Supplementary Table 6**.

Main findings: quantitative reviews

Case management interventions

Eight reviews reported quantitative findings related to the effectiveness of the interventions involving case management (**Table 1 and Supplementary Table 7**).[33–40] Seven of these reviews focused on caregivers of persons with dementia,[33–39] while one took interest in caregivers of stroke survivors.[40] Health-related outcomes varied and included: depression (n=6), health-related quality of life (n=6), anxiety (n=2), general health (n=1) and self-rated

For peer review only - http://bmjopen.bmj.com/site/about/guidelines.xhtml

health (n=1). Three reviews included a meta-analysis.[35,38,40] Five reviews reported no significant effect of case management interventions on caregivers' health outcomes,[34,35,37,39,40] one review provided inconclusive findings,[33] one review reported one primary study showing positive effects on depression,[36] and finally another review[38] showed a significant improvement in short term depressive symptoms and general health (albeit with waning effects at longer-term follow-ups). Two reviews demonstrated that multicomponent interventions that include other types of approaches in addition to case management may have positive effects[34,35] Lee et al.[35] also reported that interventions including caregivers with high quality of life at baseline or those who cared for people with less serious health conditions were less likely to be effective. The other five reviews either did not explore the issue of heterogeneity of caregivers, care receivers and care context, or stated that these data were not reported by primary studies.[40]

For peer review only

For peer review only - http://bmjopen.bmj.com/site/about/guidelines.xhtml

Table 1. Interventions involving case management.

First author, year	Included primary studies	Disease of care receivers	Characteristics of intervention	Control group	Health outcomes of caregiver	Quality of evidence
Goeman, 2016	24 RCTs, 1 cohort study, 1 case study, 2 mixed methods	Dementia	Case management by nurse or dementia care provider involving visits, calls, and emails.	Usual care, home visits, educational sessions, phone calls, helpline, or not reported	Depression, HR-QoL, general health	●●●●
Greenwood, 2016	1 quasi-experimental	Dementia	General practice-based intervention delivered by a nurse, to augment care from primary care physicians.	Usual care and educational materials	Depressive symptoms	●●○○
Hopwood, 2018	2 RCTs, 1 quasi-experimental, 7 mixed methods	Dementia	Internet-based interventions involving nurses, health professionals, social workers using various internet-based media to manage caregivers.	Face-to-face delivered information, usual care	Depression, anxiety, HR-QoL, self-rated health	●●○○
Lee, 2020	4 RCTs	Dementia or MCI	Community-based interventions delivered by various professionals. Duration varied from 3 to 18 months.	Usual care or not reported	HR-QoL	●●○○
Lucero, 2019	2 RCTs	Dementia	Telephone or computer-based health-related planning, monitored by nurses.	Usual care or training on available local resources	Depression, anxiety	●●●○
Piersol, 2017	2 RCTs	Dementia	Case management by nurses or occupational therapists.	Not reported	HR-QoL	●●○○
Reilly, 2015	11 RCTs	Dementia	Dyadic and non-dyadic interventions involving a case manager. Majority face to face, delivered by various professionals. Duration varied from 4 months to 2 years.	Waiting list, usual care, or augmented usual care	HR-QoL, depression, general health	●●●●
Pucciareli, 2020	5 RCTs, 1 quasi-RCT	Stroke	Informational interventions that included home visits and management after discharge.	Usual care or not reported	Depression, HR-QoL	●●●○

Abbreviations: MCI, mild cognitive impairment; RCT, randomized controlled trial; HR-QoL, health-related quality of life
Legend for quality assessment based on AMSTAR-2: ●●●● High; ●●●○ Moderate; ●●○○ Low
A summary of the main findings of each review is available in Supplementary Table 7.

For peer review only - http://bmjopen.bmj.com/site/about/guidelines.xhtml

Psychosocial and education/skills building interventions

A total of 23 reviews reported quantitative findings related to effectiveness of interventions involving psychosocial support, education, and skills-building for caregivers (**Tables 2 and 3 and Supplementary Table 8**). Of these, 19 reviews included and synthesized findings on both psychosocial and educational interventions,[34,35,37,39–54] while two reviews focused solely on psychosocial support,[55,56] and another two only on education/training.[57,58] Care receivers' health problems spanned from dementia (*n*=12) and stroke (*n*=4) to cancer (*n*=4) and other chronic illnesses (*n*=3). Most reviews included multiple interventions (e.g., information provision, psychosocial support, educational training, skills-building), even though the label "multicomponent" was used differently across reviews. Meta-analysis was performed in 14 reviews. Of these, seven reviews reported insignificant effect estimates or little to no effects following psychosocial and/or educational interventions,[35,40,41,44,48,54,59] four reviews reported significant effect estimates related to similar interventions,[47,52,53,58] while three reviews reported significant changes in mental health specifically after cognitive behavioral therapy (CBT) interventions.[49,55,56] Narrative reviews often failed to reach definitive conclusions due to discordant results (similar numbers of studies reporting significant and non-significant estimates),[42,43,45,46,50,51] but some reviews reported overall positive findings.[34,37] Conclusions were discordant regarding remote interventions. For instance, the evidence documenting the benefit of telephone-based psychosocial support was found to be inconclusive by Corry et al.[41] and Gonzalez-Fraile et al.,[48] while Lins et al.[52] reported positive effects on depressive symptoms. Lee et al.[35] and Hopwood et al.[34] grouped certain interventions under the "multicomponent" category and reported that these were associated with significant positive effects on health-related quality of life and anxiety and depression, respectively. Treanor et al.[44] reported significant positive effects on quality of life in the short term, with waning effects over time. Akarsu et al.[47] examined the effectiveness of psychological, multicomponent, and educational interventions in ethnic minority caregivers, reporting an overall mean reduction in depression scores.

Some reviews explored findings from primary studies depending on the caregiver, care receiver, and care context characteristics as well as implementation-related aspects. Successful interventions were reported to be more individualized[45] proactive rather than reactive,[51] developed using user input from the target groups,[39,47] and guided by competent professionals[37] or peer caregivers with relatable experiences.[35] Heckel et al.[43] reported that telephone helplines were mostly utilized by white, higher income, middle-aged females, and lack of participation from other groups of caregivers should be investigated. Corry et al.[41] acknowledged that these data are often not reported by primary studies.

For peer review only

For peer review only - http://bmjopen.bmj.com/site/about/guidelines.xhtml

Table 2. Psychosocial, psychoeducational, and skills-building interventions: reviews that included both psychosocial and educational interventions (n=19).

First author, year	Included primary studies	Disease of care receivers	Characteristics of intervention	Control group	Health outcomes of caregiver	Quality of evidence
Akarsu, 2019	13 RCTs	Dementia	Psychological, multicomponent and educational interventions. Delivered to ethnic minority caregivers.	Minimal support measures	Depression	●●●○
Gonzalez-Fraile, 2021	26 RCTs and quasi-RCTs	Dementia	Remotely delivered interventions only. Predominant components: training with or without information, support with or without information, and interventions including both support and training elements.	Usual treatment or waiting list, minimal support, providing information only	Depression, depressive symptoms, HR-QoL	●●●○
Greenwood, 2016	2 RCTs, 1 quasi-RCT	Dementia	Education, training, CBT, delivered for various durations and locations (home-based, primary care).	Usual care or not reported	Depression, general health	●●○○
Hopwood, 2018	9 RCTs, 20 mixed-method, 7 quasi-RCTs	Dementia	Internet-based only. Information, online sessions or modules, links to resources, training, peer interaction online, small groups peer support. Duration varied from 2 weeks to 12 months.	Information only, waiting list, email newsletter, usual care, telephone support, written information, video, website	HR-QoL, depression, anxiety, self-rated health	●●○○
Lee, 2020	14 RCTs	Dementia or MCI	CBT, group sessions, dyadic sessions, home visits, meetings, website, support calls.	Usual care or information only	HR-QoL	●●○○
Lins, 2014	9 RCTs	Dementia	Telephone counselling with or without educational material and workbook. Varying methods and duration (20-60 minutes per call).	Usual care or friendly calls	Depression, anxiety	●●●○
Piersol, 2017	41 RCTs and quasi-RCTs	Dementia	Group interventions, CBT, single component support interventions, multicomponent psychoeducational interventions. Delivered through various methods.	Not reported	HR-QoL, depression, anxiety	●●○○
Teahan, 2020	24 RCTs	Dementia	Counselling, information, education on dementia, CBT, relaxation techniques, communication skills, emotional control, other skills, MBSR, physical exercise, dyadic or individual.	Usual care, educational material, follow up calls, enhanced respite care	Depression, HR-QoL, general health	●●○○
Wiegelmann, 2021	37 RCTs and quasi-RCTs	Dementia	Psychoeducation, counselling, CBT, and peer support. Delivered either face-to-face or remotely.	Usual care or not reported	HR-QoL, depression	●●○○

For peer review only - http://bmjopen.bmj.com/site/about/guidelines.xhtml

Zabihi, 2020	14 RCTs	Dementia, other illnesses	Behavioral activation, education, group support interventions, among others.	Usual care, waiting lists, phone calls, educational interventions	Depression (symptoms and diagnosis)	●●○○
Gabriel, 2020	6 RCTs, 6 NRSIs	Cancer	CBT, coping theory, psychoeducation, skills building.	Usual care	Psychological/emotional and physical domains of QoL	●●○○
Heckel, 2019	2 RCTs	Cancer	Telephone helplines. Variable duration, number of outcalls, and content of the calls.	Usual care	Depression, emotional distress	●●○○
Treanor, 2019	21 RCTs and quasi-RCTs	Cancer	Ppsychoeducational approaches in the form of coping skills training. Delivered predominantly by nurses. Most face-to-face, some by telephone or video.	Usual care or information only	HR-QoL, depression, anxiety, emotional distress, physical health status	●●●○
Waldron, 2013	6 RCTs	Cancer	Skills-training and CBT. Dyadic or individual. Some interventions delivered face-to-face, some by telephone.	Usual care	HR-QoL	●●○○
Corry, 2019	21 RCTs	Various	Psychosocial, educational and psychoeducational interventions. All interventions were individual and telephone based.	Usual care or non-telephone-based support	HR-QoL, Psychological health (depression, anxiety), physical health	●●●○
Sin, 2018	26 RCTs and quasi-RCTs	Various	Web-based ICT interventions (at least part of an intervention had to be web-based). Varying content and duration (from several days to months).	Usual care or not reported	Depression, anxiety, HR-QoL	●●●○
Forster, 2012	7 RCTs	Stroke	Informational interventions. Delivered in varying formats and durations.	No information, usual care, educational material, waiting list, workbook	Depression, HR-QoL	●●●●
Minshall, 2019	8 RCTs	Stroke	Individual, group, or dyadic interventions. Delivered by various professionals, in person or through telephone. Duration varied from 1 month to 3 years.	Usual care or not reported	Depression, HR-QoL	●●●○
Pucciarelli, 2020	6 RCTs and quasi-RCTs	Stroke	Educational interventions, with components of psychoeducation. Some delivered face-to-face, some by phone/web, some through home visits.	Usual care or not reported	Depression	●●●○

Abbreviations: RCT, randomized controlled trial; NRSI, non-randomized study of intervention; CBT, cognitive behavioral therapy; QoL, quality of life; HR-QoL, health-related quality of life; MBSR, mindfulness-based stress reduction; ICT, information and communication technology.
Legend for quality assessment based on AMSTAR-2: ●●●● High; ●●●○Moderate; ●●○○ Low
A summary of the main findings of each review is available in Supplementary Table 8.

For peer review only - http://bmjopen.bmj.com/site/about/guidelines.xhtml

Table 3. Psychosocial, psychoeducational, and skills-building interventions: Reviews that included only psychosocial (n=2) or only educational (n=2) interventions.

First author, year	Included primary studies	Disease of care receivers	Characteristics of intervention	Control group	Health outcomes of caregiver	Quality of evidence
Bennett, 2019 (occupational therapy)	9 RCTs	Dementia	Occupational therapy delivered at home for dyads. Tailored and goal-oriented interventions.	Usual care, education, collaborative call	Emotional distress, HR-QoL	●●○○
Smith, 2019 (training)	19 RCTs and quasi-RCTs	Stroke and older adults	Training delivered to facilitate care after discharge. Varying delivery mode (face-to-face, by phone, by different professionals) and duration.	Usual care, information only	HR-QoL, depression, anxiety	●●●○
Hopkinson, 2019 (psychosocial)	25 RCTs and quasi-RCTs	Dementia	CBT. Varying delivery mode and duration.	Support group control, psychoeducation control, information support control, usual care	Depression, anxiety	●●○○
Wang, 2020 (psychosocial)	6 RCTs	Neurocognitive diseases	Bibliotherapy. Either web- or video-based. Varying number of sessions and duration of each session.	Usual care, waiting list, educational video	Depression, anxiety	●●●○

Abbreviations: RCT, randomized controlled trial; CBT, cognitive behavioral therapy; QoL, quality of life; HR-QoL, health-related quality of life.
Legend for quality assessment based on AMSTAR-2: ●●●● High; ●●●○Moderate; ●●○○ Low
A summary of the main findings of each review is available in Supplementary Table 8.

For peer review only - http://bmjopen.bmj.com/site/about/guidelines.xhtml

Respite care

Four reviews explored the effectiveness of respite care services (**Table 4 and Supplementary Table 9**).[60–63] Three of these reviews focused on caregivers of persons with dementia,[61–63] while one review took interest in caregivers of people with any chronic illness.[60] Health-related outcomes varied and included: depression (n=4), anxiety (n=2), and general health (n=1). The largest review in this category—and the only one including a meta-analysis—reported no significant effects on caregivers' mental health outcomes.[60] However, narrative findings from studies not eligible for meta-analysis in the same review and another review by Maffioletti et al.[61] were rather positive, although they remained discordant.[60,61] Vandepitte et al.[62] and Maayan et al.[63] reported small or insignificant effects in relation to caregivers' health outcomes. Maayan et al.[63] reported that care receivers' disease severity could have been positively correlated with the effectiveness of respite, with caregivers of patients with milder symptoms not requiring as many breaks. The price of respite care (if privately purchased) was identified as an important factor for effectiveness and access.[60] Shaw et al.[60] found that longer interventions tended to have stronger benefit than shorter ones, and that the short-term incidence of depression was reduced among people who received home respite care but not in trials that evaluated day care.

For peer review only

For peer review only - http://bmjopen.bmj.com/site/about/guidelines.xhtml

Table 4. Interventions involving respite care.

First author, year	Included primary studies	Disease of care receivers	Characteristics of intervention	Control group	Health outcomes of caregiver	Quality of evidence
Maayan, 2019	3 RCTs	Dementia	In-home respite, day care, institutional care. Delivered by trained cares as well as volunteers.	Usual care, waiting list	Depression	●●●●
Maffioletti, 2019	10 quasi-experimental, 4 cross-sectional	Dementia	Self-financed or paid day care services by professionals or volunteers. Caregivers were supported in some studies with music therapy, socializing, or just free time.	Usual care or not reported	Depression, psychological well-being, general health	●●○○
Vandepitte, 2016	5 RCTs and NRSIs	Dementia	Day care, in-home delivery of respite. Varying duration and frequency.	No respite	Depression, anxiety	●●○○
Shaw, 2009	26 RCTs and quasi-RCTs, 79 observational studies	Various	Day care, mixed interventions, in-home and institutional care.	Usual care or no respite	Depression, anxiety	●●○○

Abbreviations: RCT, randomized controlled trial; NRSI, non-randomized study of intervention.
Legend for quality assessment based on AMSTAR-2: ●●●● High; ●●●○Moderate; ●●○○ Low
A summary of the main findings of each review is available in Supplementary Table 9.

For peer review only

For peer review only - http://bmjopen.bmj.com/site/about/guidelines.xhtml

Relaxation and leisure

A total of six reviews were grouped under this category (**Table 5 and Supplementary Table 10**),[35,37,64–67] including ones focusing on caregivers to people with dementia (*n*=3), neurological diseases (*n*=1), and various chronic illnesses (*n*=2). Health-related outcomes varied and included: depression (*n*=4), health-related quality of life (*n*=4), anxiety (*n*=4), physical health (*n*=3), and blood pressure and weight (*n*=1). Two reviews included a meta-analysis.[67,68] Interventions were heterogenous: two reviews included interventions related to physical activity/exercise,[64,65] one review included relaxation/alternative medicine interventions,[35] one review focused on creative arts interventions,[66] while two other reviews focused on "miscellaneous" interventions, including exercise, leisure programs and relaxation.[37,67] These interventions had generally positive effects. Meta-analyses by both Lee et al.[35] and Cheng et al.[67] showed large positive effect sizes for the effect of relaxation and miscellaneous activities on health-related quality of life and depressive symptoms. Reviews with narrative synthesis reported that creative arts interventions tended to have positive effects on well-being,[66] while exercise programs resulted in lower blood pressure and less depressive symptoms,[64] as well as increased muscle mass, strength[65] and better physical health.[37]

Most of the reviews in this category did not consider heterogeneity associated with caregiving factors or implementation characteristics. According to Doyle et al.,[65] spousal and family caregivers may gain more from engaging in dyadic exercise compared to when their care receivers exercise independently. Miscellaneous interventions examined by Cheng et al.[67] showed that caregivers' mean age significantly moderated the intervention effects, whereby younger caregivers benefited more in terms of reducing depressive symptoms.

For peer review only - http://bmjopen.bmj.com/site/about/guidelines.xhtml

Table 5. Interventions involving relaxation, physical activity, or leisure.

First author, year	Included primary studies	Disease of care receivers	Characteristics of intervention	Control group	Health outcomes of caregiver	Quality of evidence
Cheng, 2020	12 RCTs	Dementia	Miscellaneous: physical activity, spiritual care, complementary/alternative therapies (e.g., religious activities, expressive writing).	Usual care or alternative sessions	Depression, anxiety, HR-QoL, physical health	●●●○
Lee, 2020	2 RCTs	Dementia or MCI	Complementary and alternative medicine including yoga, massage, and meditation.	No treatment or respite care	HR-QoL	●●○○
Piersol, 2017	3 experimental studies	Dementia	Exercise program, adapted leisure program, nighttime monitoring system.	Not reported	Physical health	●●○○
Irons, 2020	8 pre-post trials	Neurological diseases	Creative arts interventions, such as music, drama, dance, song writing.	Usual care or not reported	HR-QoL, anxiety, depression	●●○○
Cuthbert, 2017	9 RCTs	Various	Physical activity interventions: walking, aerobics, yoga. Varying intensities and formats.	Waiting list, usual care or not reported	Depression, anxiety; well-being, physical strengthening, blood pressure, weight	●●○○
Doyle, 2020	5 RCTs, 6 quasi-RCTs	Various	Physical activity dyadic (DyEx) and non-dyadic (DySplit) interventions.	DyEx versus DySplit (i.e., exercising together or not)	Depression, anxiety, physical health	●●○○

Abbreviations: MCI, mild cognitive impairment; RCT, randomized controlled trial; HR-QoL, health-related quality of life.
Legend for quality assessment based on AMSTAR-2: ●●●● High; ●●●○Moderate; ●●○○ Low
A summary of the main findings of each review is available in Supplementary Table 10.

For peer review only - http://bmjopen.bmj.com/site/about/guidelines.xhtml

Mindfulness-based interventions

Four reviews included interventions using mindfulness-based stress reduction techniques (**Table 6 and Supplementary Table 11**).[67,69–71] Three reviews focused on caregivers of persons with dementia,[67,69,70] while one review took interest in caregivers of people with various illnesses.[71] Health-related outcomes varied and included: depression (*n*=3), anxiety (*n*=3), and cognition and biomarkers for stress (*n*=1). Two reviews included meta-analysis.[67,69] Mindfulness-based interventions showed significant positive effects for reducing depressive symptoms and anxiety levels immediately after interventions, but the effects were largely attenuated at follow-up.[69,70] Cheng et al.[67] reported a significant positive effect on depression in grouped meta-analysis. Parkinson et al.[71] reported mixed results, with interventions showing some positive changes in anxiety and depression scores, but with small and waning effects. While some authors acknowledged the importance of potential moderating factors and heterogeneity among caregivers,[70,71] there were no findings to report regarding this question.

For peer review only

For peer review only - http://bmjopen.bmj.com/site/about/guidelines.xhtml

Table 6. Interventions involving mindfulness-based activities.

First author, year	Included primary studies	Disease of care receivers	Characteristics of intervention	Control group	Health outcomes of caregiver	Quality of evidence
Cheng, 2020	7 RCTs	Dementia	MBSR and its modifications.	Usual care or alternative sessions	Subjective well-being, depression	●●●○
Liu, 2018	5 RCTs	Dementia	MBSR and its modifications.	Usual care or active comparison (respite, social support)	Depressive symptoms, anxiety	●●●●
Shim, 2020	9 RCTs	Dementia and MCI	MBSR and its modifications.	Usual care or active comparison (psychoeducation, music-listening)	Cognition, depression, mindfulness, anxiety, biomarkers for stress	●●○○
Parkinson, 2019	1 RCT, 5 quasi-RCTs	Various	MBSR and its modifications.	Usual care or not reported	Anxiety	●●○○

Abbreviations: MBSR, Mindfulness-Based Stress Reduction; MCI, mild cognitive impairment.
Legend for quality assessment based on AMSTAR-2: ●●●● High; ●●●○Moderate; ●●○○ Low
A summary of the main findings of each review is available in Supplementary Table 11.

For peer review only

Comparison across categories of interventions

Three reviews compared the effect sizes across different types of interventions.[47,67,68] Lee et al.[68] provided a separate meta-analysis on multicomponent interventions (including social support, education, and skills-building), which showed the largest positive effect on health-related quality of life compared to single-component interventions. Similarly, Cheng et al.[67] reported that multicomponent and miscellaneous interventions had the strongest effects on depressive symptoms. However, Akarsu et al.[47] reported that effect sizes across multicomponent, psychological and educational interventions are broadly similar.

Main findings: qualitative reviews

For the qualitative part of our umbrella review, we extracted and analyzed data from 18 reviews. **Supplementary Table 4** describes the steps undertaken to apply mega-aggregation framework synthesis[31] to our data. The results of our convergent synthesis of quantitative and qualitative evidence are presented in **Figure 2.** Two themes were identified based on the findings from the 18 reviews providing qualitative data: (1) intervention outcomes and (2) implementation outcomes. These two themes reflect two main domains of intervention implementation research.[72] A detailed list of all fourth-order constructs, accompanying the third-order constructs (*verbatim* text) and references is available in **Supplementary Table 12**.

Theme 1: Intervention outcomes

This theme described the potential benefits and/or harmful effects to caregivers from participating in the interventions (i.e., efficacy and effectiveness), and was informed by 13 reviews.[33,46,52,59,60,66,71,73–78] Only two reviews[71,73] reported health-related benefits, namely decreased depression and anxiety[71,73] and better quality of life.[73] Twelve reviews documented on social outcomes of interventions, i.e., outcomes related to the caregiver's social life, day-to-day routine and relationship with care receivers and other people.[33,46,52,59,60,66,73–78] Based on these reviews, we divided social outcomes into three sub-categories: practical, emotional, and relational outcomes.

Practical outcomes were explored in nine reviews[33,52,59,60,66,73–76] examining how support interventions could introduce changes in the caregivers' day-to-day lives. According to caregivers, interventions were effective in providing them with new knowledge that enhanced coping, ensured better management of burden, and improved their caregiving skills.[59,73,74] Acquired skills increased their readiness for care, allowed them to build strategies to solve problems, and gave them a sense of normality.[52,66,73,74,76] Additionally, respite care was found by Shaw et al.[60] to "*give structure to the carer's week along with a sense of normality*" as the caregiver was able to "*match the ebb and flow of caregiving activities*." However, occasionally, there were additional burdens related to respite care use: "*many hassles [...] involved in the preparation for respite care"* that would lead to loss of *"the physical and emotional energy*."

Participation in the interventions also brought about emotional outcomes, which were described by nine reviews.[46,52,60,66,73,75–78] Across several reviews, caregivers reflected on the positive impacts of talking about their daily arduous challenges, sharing experiences, or simply providing a moment to escape from their various duties through singing, arts or just chit-chat.[52,66,76,78] These positive impacts included gaining a sense of relief, better coping with stress, enjoyment, reduced social isolation and gaining emotional support.[46,52,66,76–78] Lins et al.[52] highlighted that even the caregivers in the control group of the intervention, where participants had "*conversations only about general topics such as the weather, television, movies, news, or social activities*", reported the social interactions to be a "*helpful alternative to relieve carers*." However, some reviews described negative emotional consequences to the participation in interventions. Shaw et al.[60] argued that the physical break due to respite was "*not sufficient in itself to provide the mental break that was needed by most carers to improve their well-being*." It would require that the participants had "*total disengagement from the caring role*"[60] Moreover, they described that

For peer review only - http://bmjopen.bmj.com/site/about/guidelines.xhtml

the use of respite care can be perceived as selfish by the caregiver and bring about feelings of guilt.[60] Irons et al.[66] asserted that, while creative arts interventions may increase positive feelings, negative feelings might not be completely removed.

Finally, four reviews investigated the relational outcomes of support interventions, namely how these interventions modified the caregivers' relationships with their care receiver or peers.[66,75–77] Dyadic interventions were reported to promote increased engagement and deepened relationships between the caregiver and the care receiver.[66,76] The practice of creative arts like singing, viewing art, writing music, or creating memory albums brought a sense of reciprocity, fostered better communication, allowed for seeing the care receiver under a new light, and improved quality time.[66,76] Du Preez at al.[75] also reported increased engagement between caregivers and care receivers after returning from respite day care services.

Theme 2: Implementation outcomes

A total of 17 included reviews examined to what extent the adoption of effective interventions was determined by implementation-related barriers and facilitators that could render them ineffective in certain circumstances.[72,79] Following the classification of Proctor et al.[79] and Hull et al.[80], we synthesized the findings from these reviews in terms of (1) acceptability, (2) feasibility, (3) appropriateness, (4) sustainability, and (5) implementation costs.

Acceptability. Although the included reviews covered studies with varying designs and heterogenous interventions, we found similarities in the description of the determinants of perceived caregiver acceptability. First, seven reviews spanning almost all intervention types demonstrated that the *person-centeredness, flexibility and personalization* of the interventions promoted higher acceptability as expressed by caregivers.[33,34,46,60,66,81,82] Caregivers appreciated if interventions were able to accommodate their lifestyles and needs.[33,34,46,66,81] Some caregivers might prefer face-to-face meetings compared to online meetings,[33] while others might appreciate a self-paced program.[46] Additionally, caregivers expressed more favorability towards interventions when the deliverer considered not only their preferences, but also those of the care receiver, including their medical conditions, availability, and commitment level.[33,46,60,66,82] In a review focused on respite[60] caregivers highlighted accepting or rejecting the interventions through the lens of care receivers, and whether aspects ranging from their physical and mental health to their cooperation and approval of participation were fully considered by deliverers. Manifestations of lack of patient-centeredness affected care receivers not only during the period of respite, but also after the respite, as it took some time for care receivers to recover. Along the same lines, Miles et al.[82] indicated that caregivers were positive towards the use of patient and caregiver information and support services but suggested that *"there is not a one-size-fits-all approach which can be used, as every patient […] and their carers will have different needs, preferences and responses*."

Second, three reviews document the importance of cultural and linguistic aspects in explaining the success of support interventions,[33,52,60] in particular whether interventions were able accommodate the needs and values of different ethnic and religious caregiver groups. Language was a barrier for those who did not speak the national language that the intervention was delivered in.[52] This prevented minorities and those who did not have a full grasp of the language from participating, especially if the intervention required advanced understanding and usage of the language, such as counselling.[52] Lins et al. also explained that "*receiving counselling in the native language was also shown to contribute to building trust*." Additionally, having multicultural staff facilitated the acceptability of the interventions because better relationships could be built with the families, and the needs of the different communities could be better understood.[33,60] For example, in the use of in-home respite, minority groups would prefer working with same gender and ethnicity personnel, who also spoke the same language.[60] Ensuring the appropriateness of food based on religious restrictions, and being mindful of cultural and religious differences were also highlighted in the same review.[60]

For peer review only - http://bmjopen.bmj.com/site/about/guidelines.xhtml

Third, six reviews examined how the physical, social, and structural characteristics of interventions promoted or hindered their acceptability by informal caregivers and care receivers.[46,60,66,74–76] Settings that facilitated communication between caregivers and receivers seemed to provide caregivers with a feeling of having a ‘special place’ and thus encouraged their engagement with the intervention.[66,74] On the other hand, online-based interventions were sometimes perceived by caregivers as implying technical difficulties and hindering rapport with the staff.[46] In other words, caregivers expressed their preference for certain delivery methods, as they better suited their needs. For example, in-home respite care was seen as less disruptive compared to day care.[60] Moreover, high rates of staff turnover were reported to disrupt the continuity of care.[60] Du Preez et al.[75] asserted caregiver concerns related to a lack of knowledge about activities taking place at respite day care : "*family carers have little to no contact with the adult day service other than to ready their care recipient for the day's attendance and have little knowledge of how their care recipient spends their time while attending adult day service*."

Finally, five reviews built on qualitative findings to address the role of trust, dignity and ethical values in the degree of acceptability of support interventions.[52,60,66,75,81] Caregivers seemed to appreciate the intervention staff who they knew well, and were reluctant to accept the advice from those who they had never met.[52,81] Additionally, caregivers appraised the emotional attitude of the staff and the latter's investment in their case.[52] The more familiar the staff was with the case and needs of the caregivers, the more appreciated the intervention was.[52,81] Moreover, respecting the privacy of caregivers and receivers[60,75] and treating care receivers with care and dignity were especially valued by caregivers.[52,60,66,75] The moral values embodied by the staff were also important for caregivers.[66]

Feasibility. Intervention feasibility is defined by Proctor et al.[79] as "the extent to which a new intervention can be successfully used or carried out within a given setting. [...] It is invoked as a potential explanation of an intervention's success or failure, as reflected in recruitment, retention, or participation rates". Feasibility of support interventions was explored in 13 of the 18 qualitative reviews included in our umbrella review. We further divided this category into five dimensions: (1) recruitment, (2) accessibility, (3) availability, (4) adoption and retention, and (5) systemic factors.

First, challenges to *recruitment* were mentioned in four reviews.[46,60,75,81] Awareness about the availability of interventions was indeed deemed essential for ensuring participation.[60,75] Du Preez et al.[75] explained that "*medical practitioners were identified as having limited knowledge of community support services and access to information resulting in poor referral processes and therefore, poor utilization by family carers and people living with dementia*". In other words, the lack of knowledge about these services at the primary care level was highlighted by caregivers across reviews.[60,75] Yet, the most preferred and accessible location for dissemination and advice on support services was precisely the primary care center, as expressed by caregivers.[60]

Second, the feasibility of interventions was questioned in terms of their *accessibility* by six reviews,[33,46,52,60,75,76] which examined how both physical and non-physical external factors were potentially affecting the degree of accessibility of various interventions. For instance, some interventions excluded care receivers if they did not have a confirmed diagnosis,[33] while others, like singing and creative arts, included care receivers with diverse stages of disease.[76] Additionally, intervention sites were sometimes described as being "*too far away*", with no available or reasonable transportation to reach the site.[60,75] In such cases, the use of alternative methods like telephone-based interventions were reported to be useful, as they avoided the hustle of transportation.[52]

Third, *service availability*—that is, the coverage of support interventions in terms of time schedules and availability of staff—was broached in six reviews.[52,60,61,76,78,81] Caregivers across several studies expressed the need for the interventions to be available outside working hours, to include weekends, and even up to 24/7 availability in the case of counselling.[52,60,76,81] The availability outside of working hours enables usage of the interventions by

For peer review only - http://bmjopen.bmj.com/site/about/guidelines.xhtml

working caregivers.[60] Alternative measures, such as the use of answering machines outside counselling hours, were perceived as insufficient.[52]

Fourth, we found five reviews that looked at aspects related to *adoption and retention*, i.e., what factors affect the initiation and the continued use of the services provided as part of an intervention.[34,46,59,60,75] The use of internet-based interventions was sometimes accompanied by technical difficulties that increased the risk of dropping out, especially among older caregivers.[34,46] Sin et al.[46] reported that for internet-based psychoeducation interventions, "*usability problems (such as oral communication/chat quality, audio-visual function failure) were also identified as attributing to high drop-out rates (up to 50%) in some studies*". Additionally, with the progression of care receivers' disease, some caregivers reported not being able to leave the care receivers alone in respite care, leading to withdrawal from the interventions.[60,75] On the other hand, flexible, multicomponent and holistic approaches addressing the complex needs of caregivers and care receivers resulted in higher utilization rates.[60,75]

Finally, four reviews investigated *systemic factors* (i.e., health and social care system features that can affect the delivery and utilization of support interventions).[33,73,75,81] Caregivers reported that their experience of not feeling prioritized by the staff over system-related factors hindered the use of interventions.[75] Additionally, caregivers found it difficult to retain all the information and coordinate with different practitioners and institutions.[81] They expressed the need for a central source of information to consult with.[73] Having several providers to coordinate with was linked to other problems according to caregivers, like competition for delivery or lack of involvement of the other providers.[33]

Appropriateness. This category, which was documented in nine reviews[33,34,52,60,66,71,73,76,81] is defined by Proctor et al.[79] as the perceived suitability and usefulness of interventions to address the needs of caregivers. The appropriate delivery of interventions was largely dependent on the adoption of a patient-centered approach, and the existence of a multiagency and inter-organizational cooperation to address the specific needs of the caregivers.[33,34] However, it was emphasized that not all caregivers would benefit from all interventions.[34,66,71,76] As caregivers' needs and preferences differ, intervention components should be tailored to each case.[33,34,73] This referred to the type of intervention (e.g., psychoeducational, relaxation, etc.), the delivery mode (e.g., via phone, in person), the setting (e.g., at home, in the clinic), and type of participation (e.g., individual, in groups, dyadic).[33,34,73] Pritchard et al.[73] asserted that providing "*appropriate modality and timing of information*" to caregivers requires "*information to be presented in different ways (e.g., in writing, diagrams) repeated on several occasions and in person, not over the phone*". Additionally, some interventions such as counselling were deemed more needed during specific times, for example during crises or in acute conditions.[52] Moreover, caregivers did not always find dyadic interventions that were effective for care receivers suited to their needs.[66,76]

Sustainability. This category grouped those factors associated with a sustained, long-term use of the intervention[79] and was based on three reviews.[59,60,78] The sustainability of the interventions was claimed to depend on the needs and experiences of caregivers.[60] Smith et al.[59] described that caregivers who had encountered peers with similar experiences were more likely to continue the peer support after the intervention had ended. The fact that mindfulness-based exercises can be practiced anytime and are not limited to a certain setting seemed to facilitate its continuous use.[78] Shaw et al.[60] reported that the opportunistic use of respite care could lead to a more regular use once its potential benefits had been experienced.

Implementation costs. This category was linked to the financial costs associated with implementing or using the intervention[79] and was developed based on four reviews.[33,46,60,75] In general, interventions that were not provided free of charge made them less accessible, as affordability differed between individuals. This barrier was reported from reviews that included case management,[33] psychoeducational,[46] and respite care interventions.[60,75]

For peer review only - http://bmjopen.bmj.com/site/about/guidelines.xhtml

DISCUSSION

Summary of findings

Addressing the negative health outcomes of informal caregiving is a major challenge. In this umbrella review on support interventions for people providing informal care to older adults, we synthesized data from 47 systematic reviews covering 619 distinct primary studies. This is, to date, the most comprehensive map of the available evidence. Four main conclusions stem from our analysis.

First, whether existing interventions are effective at reducing the negative impact of caregiving on the physical and mental health of caregivers remains uncertain. Quantitative reviews provided largely discordant findings, with reviews rated as being at low risk of bias reporting trivial or no benefits.[33,38,51,63,69] Also, systematic reviews that included a meta-analysis were more likely to report a lack of effectiveness. While some case management, psychosocial and mindfulness interventions with more than one follow-up timepoint seemed to demonstrate short-term benefits, their positive effect waned as time elapsed.[38,44,69–71] Qualitative reviews provided only limited insight: although informal caregivers mentioned social and practical benefits,[52,66,73–77] they rarely spoke about how support interventions impacted their own health.[71,73]

Second, we found that multicomponent interventions showed more consistent positive effects on health outcomes across reviews, despite a large heterogeneity in what these interventions actually entailed.[34,35,67] Moreover, in the two reviews that performed meta-analyses by type of intervention, multicomponent interventions showed the largest effect sizes.[35,67] In this respect, our findings corroborate the conclusions of two previously published umbrella reviews.[18,20]

The third conclusion is that the available evidence on support interventions relies on the simplistic assumption that informal caregivers represent a homogenous target population, with little attention being paid to the variability in caregiver, care receiver, care context and implementation characteristics. Hence, while a number of reviews reported valuable information about the characteristics of the interventions being evaluated,[35,37,39,45,47,51] the socioeconomic and ethnic background of caregivers and care receivers and the nature of their relationship were largely overlooked. Others have emphasized the need to better account for the social determinants of health among informal caregivers.[83] Their diversity goes beyond the obvious differences in the underlying health conditions of the persons their provide care to, and this reality should come under greater scrutiny in future studies designed to assess the effectiveness of support interventions.

Finally, our overview casts new light onto how support interventions are experienced by informal caregivers. By synthesizing the qualitative findings of 18 distinct reviews, we showed that caregivers mention a myriad of social benefits, for instance improved relationships with care receivers, better organized routine, and less stress/burden associated with caregiving.[33,45,52,59,60,66,73–78] Across multiple reviews, caregivers were found to favor flexible, person-centered, and needs-based interventions rather than ‘off-the-rack’ support services.[33,34,45,60,66,81,82] This serves to further emphasize that one-size-fits-all approaches are unwarranted since different caregivers have different preferences in terms of, among others, mode of delivery and duration of what constitute adequate support. Interestingly, we were able to find evidence across most implementation outcomes highlighted in the framework by Proctor et al.[79] (acceptability, feasibility, appropriateness, sustainability, and cost). However, *fidelity* of the interventions, i.e., the degree to which an intervention was implemented as it was intended, was not reported.

The extensive set of qualitative findings incorporated in this umbrella review shows that support interventions targeting informal caregivers seem to improve a wide range of practical, emotional, and relational outcomes. It is likely that these benefits translate indirectly into positive changes in caregivers’ mental and physical health, even though high-quality evidence for this connection was lacking from quantitative reviews. To better visualize this pathway, we organized the findings from our umbrella review into a framework, *From Support Interventions to Improved Caregiver Outcomes (SIICO)*, as presented in **Figure 2**. While relations in this framework are only

For peer review only - http://bmjopen.bmj.com/site/about/guidelines.xhtml

hypothesized and remain untested, it represents an attempt to visualize the numerous mechanisms implicated in previous research that link interventions to caregivers' health. However, our hypothetical connections between the different boxes of the framework should be interpreted with caution and deserve further scrutiny, especially concerning the potential mediating effect and transition from social and practical benefits to the improvement of objective health outcomes. It is possible that the majority of these interventions are only effective on outcomes related to health but not considered herein (e.g., burden, life satisfaction, wellbeing) and on other aspects of caregivers' lives (e.g., ability to reconcile caregiving and employment, volunteering, socializing and/or leisure). It is also possible that the observed social and relational benefits simply do not translate into measurable improvements in health outcomes, or that these improvements remain partly invisible due to methodological issues (e.g., lack of statistical power, suboptimal control groups, inconsistent outcome measurements, or insufficiently long follow-ups).

Beyond its initial goals, our umbrella review identified several important knowledge gaps in caregiver intervention research that, we believe, could serve as a roadmap for future studies in this field. Hence, there was a clear overrepresentation of certain types of interventions (i.e., psychosocial interventions such as cognitive therapies, group or individual support and psychoeducation), care receiver diseases (i.e., dementia), and outcomes (i.e., mental health). Our umbrella review also highlights the overproduction of systematic reviews and meta-analyses on the effectiveness of support interventions for informal caregivers: more than 145 reviews have been published in the last two decades and, as expected, the overlap between them is substantial. This raises questions about the potential waste of valuable research resources, especially since the overall quality of published reviews was poor. Approximately two-thirds of the reviews that we identified were rated as being of 'critically low' quality (although one should keep in mind that some were carried out before current guidelines and risk-of-bias appraisal tools became mainstream). Of particular note, qualitative synthesis and reporting practices were often subpar, with important contextual and methodological items missing from an unexpectedly large share of included reviews. The lack of consensus regarding the classification of support interventions was yet another struggle: much like Gaugler et al.,[30] we found that not only were certain types of interventions described differently across primary studies, but even the same primary studies (i.e., same interventions) were sometimes put into different categories from one review to another. This hinders the comparability of findings across primary studies, reviews, and umbrella reviews, and makes any attempt at drawing robust conclusions about the effectiveness of interventions challenging. Others have already mentioned these incoherencies, together with other methodological shortcomings such as incomplete reporting in the included primary studies, which has an inevitable ripple effect on the degree of completeness of reviews.[20,21,84] Finally, our mostly inconclusive findings despite an abundance of published literature highlight the important methodological flaws that afflict a large number of primary studies. We believe that this should prompt a discussion between academics, stakeholders and public funding agencies. Maybe it is now time to take the advice from English methodologist Doug Altman seriously: we need less research, better research, and research done for the right reasons.[85]

Implications for public health and practice

Against the backdrop of staff shortages and budget restrictions worldwide (exacerbated by the ongoing Covid-19 pandemic), certain public health services will need to be prioritized over others. Four main implications for public health and practice emerged from our review. First, better intervention research and evaluation practices are warranted to create an evidence-base for resource allocation: we won't know what works best in different contexts if we keep relying on low-quality studies. Second, time has come for a more targeted approach to intervention design. While caregiver interventions may not work for the 'average caregiver', targeting high-risk groups—e.g., caregivers with preexisting conditions, multimorbidity or frailty—might deliver more convincing and cost-effective results. Third, since our healthcare systems are already under considerable pressure, support interventions should build on existing resources rather than entirely new services. To our surprise, very few of the interventions included in the systematic reviews and meta-analyses that we synthesized mustered primary care professionals to support informal caregivers. Yet, family physicians and home care nurses (among others) have frequent contacts with caregivers, with whom they often share a long-lasting relationship. They should have the means to remain vigilant, identify high-risk subgroups of caregivers and enlist them in relevant support programs. Fourth, our umbrella review also highlights the potential

For peer review only - http://bmjopen.bmj.com/site/about/guidelines.xhtml

for non-profit organizations: trained volunteers could, for instance, take a larger role in delivering social care services to alleviate the burden of caregivers.

Implications for research

Our findings may be useful for decision makers trying to untangle the state of the evidence on this complex topic, but also for those interested in more specific intervention types, given the broadness of our review. There are moreover several implications for future research stemming from our umbrella review. First, the enforcement of *a priori* protocol registration could avoid the wasteful production of reviews. Second, the consistent application of systematic review guidelines (e.g., PRISMA guidelines, Cochrane methods, guidance by Joanna Briggs Institute) is warranted to ensure higher quality of evidence synthesis. Third, at the primary study level, a better definition of intervention components and a clear harmonization of intervention types is needed to ensure comparability of the generated evidence. Last, future interventions should identify and report results across subgroups defined by caregiver, care receiver and care context characteristics, and consider including not only social and relational outcomes, but also objective mental and physical health outcomes, measured comprehensively and over an extended period of time. This will be essential to better understand the potential pathways connecting social to physical health outcomes, although the latter will require a mixed-method evaluation approach.

Strengths and limitations

The primary strength of this umbrella review is the rigorous study selection process, initiating from a generous search strategy identifying over six thousand abstracts. Such process was completed in duplicate and included quality and overlap assessment. Further, we included both quantitative and qualitative evidence to better understand the complex phenomenon of caregiver support interventions and their impact on health. In addition, data synthesis and framework elaboration were guided by existing theory and expertise within the group, adding to the rigor of the review. However, umbrella reviews are limited in terms of drawing conclusive statements, given that they do not assess primary studies, nor re-test meta-analyses provided by single systematic reviews. Thus, the interpretability of our findings is limited by the quality and conclusions of included reviews, which are considerably heterogeneous across reviews. In addition, recent primary studies on new interventions may not be captured, as they may not yet have been included in systematic reviews. Finally, our umbrella review focused on caregiver-centered interventions and, thus, could not shed light on the potentially positive spillover effect of formal care services delivered to patients on informal caregivers' health. Yet, the importance of well-organized and sufficiently staffed professional services for household chores and personal care should not be underestimated, and the expansion of support services for informal caregivers cannot come to the detriment of formal care.

Conclusions

Despite an abundance of systematic reviews, whether certain support interventions are effective at improving informal caregivers' physical and/or mental health is uncertain due to a lack of high-quality evidence. It seems that multicomponent and flexible interventions are more likely to address the complex needs of caregivers, making them more acceptable and thus leading to more tangible effects on objective health outcomes. To confirm this, we do not need more reviews: we need more carefully designed intervention studies that look at both subjective and objective health outcomes, and account for heterogeneity in caregiving.

For peer review only - http://bmjopen.bmj.com/site/about/guidelines.xhtml

CONTRIBUTORSHIP STATEMENT: ACL obtained the funding and drafted the protocol including the initial methodology for the umbrella review. ACL, LM, MK, LD and LBS participated in further conceptualizing the protocol and critically reviewing it before publication. For the umbrella review, all authors participated in study selection and quality appraisal of the reviews in duplicate. MK and AA extracted and cross-checked data from selected reviews. MK and AA drafted the initial manuscript, which was critically revised several times by ACL, LM, LD and LBS. The review team held several meetings regarding methodology, interpretation of data and desired output of the project. All authors gave final approval to this version of the protocol. All authors agree to be accountable for all aspects of the work in ensuring that questions related to the accuracy or integrity of any part of the work are appropriately investigated and resolved.

COMPETING INTERESTS STATEMENT: None declared.

ETHICS APPROVAL: this review is a tertiary synthesis of published systematic reviews (secondary synthesis). Thus, no ethical approval was sought and no contact was made with participants of primary studies included in systematic reviews.

FUNDING STATEMENT: This work was supported by the Swedish Research Council for Health, Working Life and Welfare (FORTE) grant number 2020-01544.

DATA SHARING STATEMENT: Data sharing is not applicable as no datasets were generated and/or analysed for this study.

ACKNOWLEDGMENTS: We wish to thank Emma-Lotta Säätelä and Jonas Pettersson, librarians at Karolinska Institutet, for preparing the search strategy and performing the searches across databases.

WORD COUNT: 8005

For peer review only

For peer review only - http://bmjopen.bmj.com/site/about/guidelines.xhtml

REFERENCES

1 AARP and National Alliance for Caregiving. Caregiving in the United States. 2020. doi:https://doi.org/10.26419/ppi.00103.001

2 Kingston A, Wohland P, Wittenberg R, *et al.* Is late-life dependency increasing or not? A comparison of the Cognitive Function and Ageing Studies (CFAS). *The Lancet* 2017;**390**:1676–84. doi:10.1016/S0140-6736(17)31575-1

3 Verbeek-Oudijk D, Woittiez I, Eggink E, *et al.* Who cares in Europe? A comparison of long-term care fore the over-50s in sixteen European countries. *The Hague: The Netherlands Institute for Social Research (SCP)* 2014;:1–109.http://www.google.nl/url?sa=t&rct=j&q=&esrc=s&source=web&cd=1&ved=0CCUQFjAA&url=http://www.scp.nl/dsresource?objectid=36940&type=org&ei=J5G_VNHoAoHeUOnpgoAC&usg=AFQjCNGLqTpygPklGLJ6Xsaat5OnvYyHtQ&sig2=X9Db9df5KLvp3NNslz-Gfg&bvm=bv.83829542

4 Haakenstad A, Irvine CMS, Knight M, *et al.* Measuring the availability of human resources for health and its relationship to universal health coverage for 204 countries and territories from 1990 to 2019: a systematic analysis for the Global Burden of Disease Study 2019. *The Lancet* 2022;**399**:2129–54. doi:10.1016/s0140-6736(22)00532-3

5 European Commission. *Informal care in Europe*. 2018.

6 Pickard L. A growing care gap' the supply of unpaid care for older people by their adult children in England to 2032. *Ageing Soc* 2015;**35**:96–123. doi:10.1017/S0144686X13000512

7 Lindt N, Van Berkel J, Mulder BC. Determinants of overburdening among informal carers: A systematic review. *BMC Geriatr* 2020;**20**:1–12. doi:10.1186/s12877-020-01708-3

8 Mello J de A, Macq J, Van Durme T, *et al.* The determinants of informal caregivers' burden in the care of frail older persons: a dynamic and role-related perspective. *Aging Ment Health* 2017;**21**:838–43. doi:10.1080/13607863.2016.1168360

9 Conde-Sala JL, Garre-Olmo J, Turró-Garriga O, *et al.* Differential features of burden between spouse and adult-child caregivers of patients with Alzheimer's disease: An exploratory comparative design. *Int J Nurs Stud* 2010;**47**:1262–73. doi:10.1016/j.ijnurstu.2010.03.001

10 Johansson MF, McKee KJ, Dahlberg L, *et al.* Negative Impact and Positive Value of Caregiving in Spouse Carers of Persons with Dementia in Sweden. *Int J Environ Res Public Health* 2022;**19**. doi:10.3390/ijerph19031788

11 Roth DL, Fredman L, Haley WE. Informal caregiving and its impact on health: A reappraisal from population-based studies. *Gerontologist* 2015;**55**:309–19. doi:10.1093/geront/gnu177

12 Schulz R, Beach SR. Caregiving as a risk factor for mortality: The caregiver health effects study. *J Am Med Assoc* 1999;**282**:2215–9. doi:10.1001/jama.282.23.2215

13 Yuan J, Grühn D. Time Effects of Informal Caregiving on Cognitive Function and Well-Being: Evidence From ELSA. *Gerontologist* 2021;**61**:341–51. doi:10.1093/geront/gnaa114

14 Bom J, Bakx P, Schut F, *et al.* The Impact of Informal Caregiving for Older Adults on the Health of Various Types of Caregivers: A Systematic Review. *Gerontologist* 2019;**59**:e629–42. doi:10.1093/geront/gny137

15 Pinquart M, Sörensen S. Correlates of physical health of informal caregivers: A meta-analysis. *Journals of Gerontology - Series B Psychological Sciences and Social Sciences* 2007;**62**:126–37. doi:10.1093/geronb/62.2.P126

16 Pinquart M, Sörensen S. Gender differences in caregiver stressors, social resources, and health: An updated meta-analysis. *Journals of Gerontology - Series B Psychological Sciences and Social Sciences* 2006;**61**:33–45. doi:10.1093/geronb/61.1.P33

17 Aromataris E, Fernandez R, Godfrey CM, *et al.* Summarizing systematic reviews: Methodological development, conduct and reporting of an umbrella review approach. *Int J Evid Based Healthc* 2015;**13**:132–40. doi:10.1097/XEB.0000000000000055

18 Dickinson C, Dow J, Gibson G, *et al.* Psychosocial intervention for carers of people with dementia: What components are most effective and when? A systematic review of systematic reviews. *Int Psychogeriatr* 2017;**29**:31–43. doi:10.1017/S1041610216001447

19 World Health Organization. Evidence profile: caregiver support. *Integrated Care for Older People: Guidelines on Community-Level Interventions to Manage Declines in Intrinsic Capacity* 2017;:35.

20 Dalton J, Thomas S, Harden M, *et al.* Updated meta-review of evidence on support for carers. *J Health Serv Res Policy* 2018;**23**:196–207. doi:10.1177/1355819618766559

For peer review only - http://bmjopen.bmj.com/site/about/guidelines.xhtml

21 Spiers GF, Liddle J, Kunonga TP, *et al.* What are the consequences of caring for older people and what interventions are effective for supporting unpaid carers? A rapid review of systematic reviews. *BMJ Open* 2021;**11**:1–10. doi:10.1136/bmjopen-2020-046187

22 Calderón-Larrañaga A, Kirvalidze M, Dahlberg L, *et al.* Effectiveness of interventions to address the negative health outcomes of informal caregiving to older adults: protocol for an umbrella review. *PROSPERO CRD42021252841* 2021.

23 Calderón-Larrañaga A, Kirvalidze M, Dahlberg L, *et al.* Effectiveness of interventions to address the negative health outcomes of informal caregiving to older adults: Protocol for an umbrella review. *BMJ Open* 2021;**11**. doi:10.1136/bmjopen-2021-053117

24 Gates M, Gates A, Pieper D, *et al.* Reporting guideline for overviews of reviews of healthcare interventions: The Preferred Reporting Items for Overviews of Reviews (PRIOR) statement. *MetaArXiv* Published Online First: 2022. doi:10.31222/osf.io/82wau

25 Veritas Health Innovation. Covidence systematic review software. www.covidence.org

26 Shea BJ, Reeves BC, Wells G, *et al.* AMSTAR 2: A critical appraisal tool for systematic reviews that include randomised or non-randomised studies of healthcare interventions, or both. *BMJ (Online)* 2017;**358**:j4008. doi:10.1136/bmj.j4008

27 Pieper D, Antoine SL, Mathes T, *et al.* Systematic review finds overlapping reviews were not mentioned in every other overview. *J Clin Epidemiol* 2014;**67**:368–75. doi:10.1016/j.jclinepi.2013.11.007

28 Pérez-Bracchiglione J, Meza N, Bangdiwala SI, *et al.* Graphical Representation of Overlap for OVErviews : GROOVE tool . *Res Synth Methods* 2022;:1–8. doi:10.1002/jrsm.1557

29 Pollock M, Fernandes RM, Newton AS, *et al.* A decision tool to help researchers make decisions about including systematic reviews in overviews of reviews of healthcare interventions. *Syst Rev* 2019;**8**:1–8. doi:10.1186/s13643-018-0768-8

30 Gaugler JE, Jutkowitz E, Shippee TP, *et al.* Consistency of dementia caregiver intervention classification: An evidence-based synthesis. *Int Psychogeriatr* 2017;**29**:19–30. doi:10.1017/S1041610216001514

31 Hendricks L, Eshun-Wilson I, Rohwer A. A mega-aggregation framework synthesis of the barriers and facilitators to linkage, adherence to ART and retention in care among people living with HIV. *Syst Rev* 2021;**10**:1–28. doi:10.1186/s13643-021-01582-z

32 Van Houtven CH, Voils CI, Weinberger M. An organizing framework for informal caregiver interventions: Detailing caregiving activities and caregiver and care recipient outcomes to optimize evaluation efforts. *BMC Geriatr* 2011;**11**. doi:10.1186/1471-2318-11-77

33 Goeman D, Renehan E, Koch S. What is the effectiveness of the support worker role for people with dementia and their carers? A systematic review. BMC Health Serv Res. 2016;**16**. doi:10.1186/s12913-016-1531-2

34 Hopwood J, Walker N, McDonagh L, *et al.* Internet-Based Interventions Aimed at Supporting Family Caregivers of People With Dementia: Systematic Review. *J Med Internet Res* 2018;**20**:e216. doi:https://dx.doi.org/10.2196/jmir.9548

35 Lee M, Ryoo JH, Crowder J, *et al.* A systematic review and meta-analysis on effective interventions for health-related quality of life among caregivers of people with dementia. *J Adv Nurs* 2020;**76**:475–89. doi:https://dx.doi.org/10.1111/jan.14262

36 Lucero RJ, Fehlberg EA, Patel AGM, *et al.* The effects of information and communication technologies on informal caregivers of persons living with dementia: A systematic review. *Alzheimer's and Dementia: Translational Research and Clinical Interventions* 2019;**5**:1–12. doi:10.1016/j.trci.2018.11.003

37 Piersol CV, Canton K, Connor SE, *et al.* Effectiveness of Interventions for Caregivers of People With Alzheimer's Disease and Related Major Neurocognitive Disorders: A Systematic Review. Am J Occup Ther. 2017;**71**:p1-7105180020. doi:10.5014/ajot.2017.027581

38 Reilly S, Miranda-Castillo C, Malouf R, *et al.* Case management approaches to home support for people with dementia. Cochrane Database of Systematic Reviews. 2015;**2017**. doi:10.1002/14651858.CD008345.pub2

39 Greenwood N, Pelone F, Hassenkamp AM. General practice based psychosocial interventions for supporting carers of people with dementia or stroke: a systematic review. *BMC Fam Pract* 2016;**17**:3. doi:https://dx.doi.org/10.1186/s12875-015-0399-2

40 Pucciarelli G, Lommi M, Magwood GS, *et al.* Effectiveness of dyadic interventions to improve stroke patient-caregiver dyads' outcomes after discharge: A systematic review and meta-analysis study. European Journal of Cardiovascular Nursing. 2021;**20**:14–33. doi:10.1177/1474515120926069

For peer review only - http://bmjopen.bmj.com/site/about/guidelines.xhtml

41 Corry M, Neenan K, Brabyn S, *et al.* Telephone interventions, delivered by healthcare professionals, for providing education and psychosocial support for informal caregivers of adults with diagnosed illnesses. *Cochrane Database of Systematic Reviews* 2019;**5**:CD012533. doi:https://dx.doi.org/10.1002/14651858.CD012533.pub2
42 Gabriel I, Creedy D, Coyne E. A systematic review of psychosocial interventions to improve quality of life of people with cancer and their family caregivers. Nurs Open. 2020;**7**:1299–312. doi:10.1002/nop2.543
43 Heckel L, Heynsbergh NL, Livingston PM. Are cancer helplines effective in supporting caregivers? A systematic review. Supportive Care in Cancer. 2019;**27**:3219–31. doi:10.1007/s00520-019-04807-z
44 Treanor CJ, Santin O, Prue G, *et al.* Psychosocial interventions for informal caregivers of people living with cancer. *Cochrane Database of Systematic Reviews* 2019;**2019**. doi:10.1002/14651858.CD009912.pub2
45 Waldron EA, Janke EA, Bechtel CF, *et al.* A systematic review of psychosocial interventions to improve cancer caregiver quality of life. *Psychooncology* 2013;**22**:1200–7. doi:https://dx.doi.org/10.1002/pon.3118
46 Sin J, Henderson C, Spain D, *et al.* eHealth interventions for family carers of people with long term illness: A promising approach? Clin Psychol Rev. 2018;**60**:109–25. doi:10.1016/j.cpr.2018.01.008
47 Akarsu NE, Prince MJ, Lawrence VC, *et al.* Depression in carers of people with dementia from a minority ethnic background: Systematic review and meta-analysis of randomised controlled trials of psychosocial interventions. Int J Geriatr Psychiatry. 2019;**34**:790–806. doi:10.1002/gps.5070
48 González-Fraile E, Ballesteros J, Rueda JR, *et al.* Remotely delivered information, training and support for informal caregivers of people with dementia. Cochrane Database of Systematic Reviews. 2021;**2021**. doi:10.1002/14651858.CD006440.pub3
49 Zabihi S, Lemmel FK, Orgeta V. Behavioural Activation for Depression in Informal Caregivers: A Systematic Review and Meta-Analysis of Randomised Controlled Clinical Trials. *J Affect Disord* 2020;**274**:1173–83. doi:10.1016/j.jad.2020.03.124
50 Wiegelmann H, Speller S, Verhaert LM, *et al.* Psychosocial interventions to support the mental health of informal caregivers of persons living with dementia - a systematic literature review. *BMC Geriatr* 2021;**21**:94. doi:https://dx.doi.org/10.1186/s12877-021-02020-4
51 Forster A, Brown L, Smith J, *et al.* Information provision for stroke patients and their caregivers. *Cochrane Database of Systematic Reviews* Published Online First: 14 November 2012. doi:10.1002/14651858.cd001919.pub3
52 Lins S, Hayder-Beichel D, Rucker G, *et al.* Efficacy and experiences of telephone counselling for informal carers of people with dementia. *Cochrane Database of Systematic Reviews* 2014;:CD009126. doi:https://dx.doi.org/10.1002/14651858.CD009126.pub2
53 Teahan Á, Lafferty A, McAuliffe E, *et al.* Psychosocial Interventions for Family Carers of People With Dementia: A Systematic Review and Meta-Analysis. *J Aging Health* 2020;**32**:1198–213. doi:10.1177/0898264319899793
54 Minshall C, Pascoe MC, Thompson DR, *et al.* Psychosocial interventions for stroke survivors, carers and survivor-carer dyads: a systematic review and meta-analysis. *Top Stroke Rehabil* 2019;**26**:554–64. doi:https://dx.doi.org/10.1080/10749357.2019.1625173
55 Wang S, Bressington DT, Leung AYM, *et al.* The effects of bibliotherapy on the mental well-being of informal caregivers of people with neurocognitive disorder: A systematic review and meta-analysis. *Int J Nurs Stud* 2020;**109**. doi:10.1016/j.ijnurstu.2020.103643
56 Hopkinson MD, Reavell J, Lane DA, *et al.* Cognitive Behavioral Therapy for Depression, Anxiety, and Stress in Caregivers of Dementia Patients: A Systematic Review and Meta-Analysis. *Gerontologist* 2019;**59**:e343–62. doi:https://dx.doi.org/10.1093/geront/gnx217
57 Smith TO, Pearson M, Pfeiffer K, *et al.* Caregiver Interventions for Adults Discharged from the Hospital: Systematic Review and Meta-Analysis. J Am Geriatr Soc. 2019;**67**:1960–9. doi:10.1111/jgs.16048
58 Bennett S, Laver K, Voigt-Radloff S, *et al.* Occupational therapy for people with dementia and their family carers provided at home: a systematic review and meta-analysis. *BMJ Open* 2019;**9**:e026308. doi:https://dx.doi.org/10.1136/bmjopen-2018-026308
59 Smith R, Greenwood N. The impact of volunteer mentoring schemes on carers of people with dementia and volunteer mentors: a systematic review. *Am J Alzheimers Dis Other Demen* 2014;**29**:8–17. doi:https://dx.doi.org/10.1177/1533317513505135
60 Shaw C, McNamara R, Abrams K, *et al.* Systematic review of respite care in the frail elderly. *Health Technol Assess* 2009;**13**:1–224, iii. doi:https://dx.doi.org/10.3310/hta13200

For peer review only - http://bmjopen.bmj.com/site/about/guidelines.xhtml

61 Maffioletti VLR, Baptista MAT, Santos RL, *et al.* Effectiveness of day care in supporting family caregivers of people with dementia: A systematic review. *Dement Neuropsychol* 2019;**13**:268–83. doi:https://dx.doi.org/10.1590/1980-57642018dn13-030003

62 Vandepitte S, Van Den Noortgate N, Putman K, *et al.* Effectiveness of respite care in supporting informal caregivers of persons with dementia: a systematic review. Int J Geriatr Psychiatry. 2016;**31**:1277–88. doi:10.1002/gps.4504

63 Maayan N, Soares-Weiser K, Lee H. Respite care for people with dementia and their carers. Cochrane Database of Systematic Reviews. 2014;**2014**. doi:10.1002/14651858.CD004396.pub3

64 Cuthbert CA, King-Shier K, Ruether D, *et al.* What is the state of the science on physical activity interventions for family caregivers? A systematic review and RE-AIM evaluation. J Phys Act Health. 2017;**14**:578–95. doi:10.1123/jpah.2016-0280

65 Doyle KL, Toepfer M, Bradfield AF, *et al.* Systematic review of exercise for caregiver-care recipient dyads: What is best for spousal caregivers - Exercising together or not at all? Gerontologist. 2021;**61**:E283–301. doi:10.1093/geront/gnaa043

66 Yoon Irons J, Garip G, Cross AJ, *et al.* An integrative systematic review of creative arts interventions for older informal caregivers of people with neurological conditions. PLoS One. 2020;**15**. doi:10.1371/journal.pone.0243461

67 Cheng ST, Li KK, Losada A, *et al.* The effectiveness of nonpharmacological interventions for informal dementia caregivers: An updated systematic review and meta-analysis. *Psychol Aging* 2020;**35**:55–77. doi:https://dx.doi.org/10.1037/pag0000401

68 Lee M, Ryoo JH, Chung M, *et al.* Effective interventions for depressive symptoms among caregivers of people with dementia: A systematic review and meta-analysis. *Dementia* 2020;**19**:2368–98. doi:https://dx.doi.org/10.1177/1471301218822640

69 Liu Z, Sun YY, Zhong BL. Mindfulness-based stress reduction for family carers of people with dementia. *Cochrane Database of Systematic Reviews* 2018;**8**:CD012791. doi:https://dx.doi.org/10.1002/14651858.CD012791.pub2

70 Shim M, Tilley JL, Im S, *et al.* A Systematic Review of Mindfulness-Based Interventions for Patients with Mild Cognitive Impairment or Dementia and Caregivers. J Geriatr Psychiatry Neurol. 2021;**34**:528–54. doi:10.1177/0891988720957104

71 Parkinson B, Lawrence M, McElhinney E, *et al.* Mindfulness for people with long-term conditions and their family caregivers: A systematic review. Complement Ther Clin Pract. 2019;**34**:76–86. doi:10.1016/j.ctcp.2018.10.019

72 Damschroder LJ, Aron DC, Keith RE, *et al.* Fostering implementation of health services research findings into practice: A consolidated framework for advancing implementation science. *Implementation Science* 2009;**4**:1–15. doi:10.1186/1748-5908-4-50

73 Pritchard E, Cussen A, Delafosse V, *et al.* Interventions supporting caregiver readiness when caring for patients with dementia following discharge home: A mixed-methods systematic review. *Australas J Ageing* 2020;**39**:e239–50. doi:https://dx.doi.org/10.1111/ajag.12765

74 Boots LM, de Vugt ME, van Knippenberg RJ, *et al.* A systematic review of Internet-based supportive interventions for caregivers of patients with dementia. *Int J Geriatr Psychiatry* 2014;**29**:331–44. doi:https://dx.doi.org/10.1002/gps.4016

75 Du Preez J, Millsteed J, Marquis R, *et al.* The Role of Adult Day Services in Supporting the Occupational Participation of People with Dementia and Their Carers: An Integrative Review. *Healthcare* 2018;**6**:8. doi:https://dx.doi.org/10.3390/healthcare6020043

76 Bourne P, Camic PM, Crutch SJ. Psychosocial outcomes of dyadic arts interventions for people with a dementia and their informal caregivers: A systematic review. Health Soc Care Community. 2021;**29**:1632–49. doi:10.1111/hsc.13267

77 Dam AE, de Vugt ME, Klinkenberg IP, *et al.* A systematic review of social support interventions for caregivers of people with dementia: Are they doing what they promise? *Maturitas* 2016;**85**:117–30. doi:https://dx.doi.org/10.1016/j.maturitas.2015.12.008

78 Li G, Yuan H, Zhang W. The Effects of Mindfulness-Based Stress Reduction for Family Caregivers: Systematic Review. Arch Psychiatr Nurs. 2016;**30**:292–9. doi:10.1016/j.apnu.2015.08.014

79 Proctor E, Silmere H, Raghavan R, *et al.* Outcomes for implementation research: Conceptual distinctions, measurement challenges, and research agenda. *Administration and Policy in Mental Health and Mental Health Services Research* 2011;**38**:65–76. doi:10.1007/s10488-010-0319-7

80 Hull L. Implementation science research development tool (ImpRes).

For peer review only - http://bmjopen.bmj.com/site/about/guidelines.xhtml

81 Bunn F, Goodman C, Pinkney E, *et al.* Specialist nursing and community support for the carers of people with dementia living at home: An evidence synthesis. *Health Soc Care Community* 2016;**24**:48–67. doi:10.1111/hsc.12189

82 Miles L, McCausland BMS, Patel HP, *et al.* A systematic review of the provision and efficacy of patient and carer information and support (PCIS) interventions for patients with dementia and their informal carers. Aging Clin Exp Res. 2020;**32**:2439–48. doi:10.1007/s40520-019-01428-8

83 Young HM, Bell JF, Whitney RL, *et al.* Social Determinants of Health: Underreported Heterogeneity in Systematic Reviews of Caregiver Interventions. *Gerontologist* 2020;**60**:S14–28. doi:10.1093/geront/gnz148

84 Cheng ST, Zhang F. A comprehensive meta-review of systematic reviews and meta-analyses on nonpharmacological interventions for informal dementia caregivers. *BMC Geriatr* 2020;**20**. doi:10.1186/s12877-020-01547-2

85 Altman DG. The scandal of poor medical research. *BMJ* 1994;**308**:283–4. doi:10.1136/bmj.308.6924.283

86 Page MJ, McKenzie JE, Bossuyt PM, *et al.* The PRISMA 2020 statement: An updated guideline for reporting systematic reviews. *The BMJ* 2021;**372**. doi:10.1136/bmj.n71

For peer review only

For peer review only - http://bmjopen.bmj.com/site/about/guidelines.xhtml

FIGURE LEGEND

Figure 1.
Caption: PRISMA 2020 flow diagram.[86]
Footnote:
*Quantitative reviews with AMSTAR-2 category of "critically low" quality were excluded; Qualitative reviews with two or more critical flaws were excluded.
**Overlap assessment was completed for quantitative and qualitative reviews separately and in distinct subgroups based on support intervention types.
***Out of 47 distinct reviews, 10 contributed to qualitative data only, 8 were included as both qualitative and quantitative data sources, while 29 reviews contributed exclusively to quantitative synthesis.

Figure 2.
Caption: *From Support Interventions to Improved Caregiver Outcomes* (*SIICO)* framework.
Footnote:
The list of potential modifying factors and sources of heterogeneity of intervention effects, such as relationship context, caregiver, and care receiver characteristics, were inspired by Van Houtven et al.'s organizing framework for caregiver interventions and amended by review authors based on expert opinion and review of broader caregiving literature (boxes *Context of the caregiving relationship*; *Caregiver;* and *Care receiver*).
[1]The typology of interventions by Gaugler et al. was reorganized in a data-driven fashion and currently includes six types of interventions (box *Intervention type*).
[2]Based on qualitative findings of our umbrella review, there is evidence that interventions affect practical, emotional, and relational aspects of caregivers' lives (box I*ntervention social outcomes*).
[3]According to mainly quantitative data, some of the interventions might also have effects on caregivers' mental health, physical health, and health-related quality of life (box *Intervention health outcomes*).
[4]Intervention effects are also influenced by implementation outcomes, which were selected based on Procter et al.'s outcomes for implementation research, and derived from qualitative data, as expressed by caregivers (box *Implementation outcomes*).

For peer review only

For peer review only - http://bmjopen.bmj.com/site/about/guidelines.xhtml

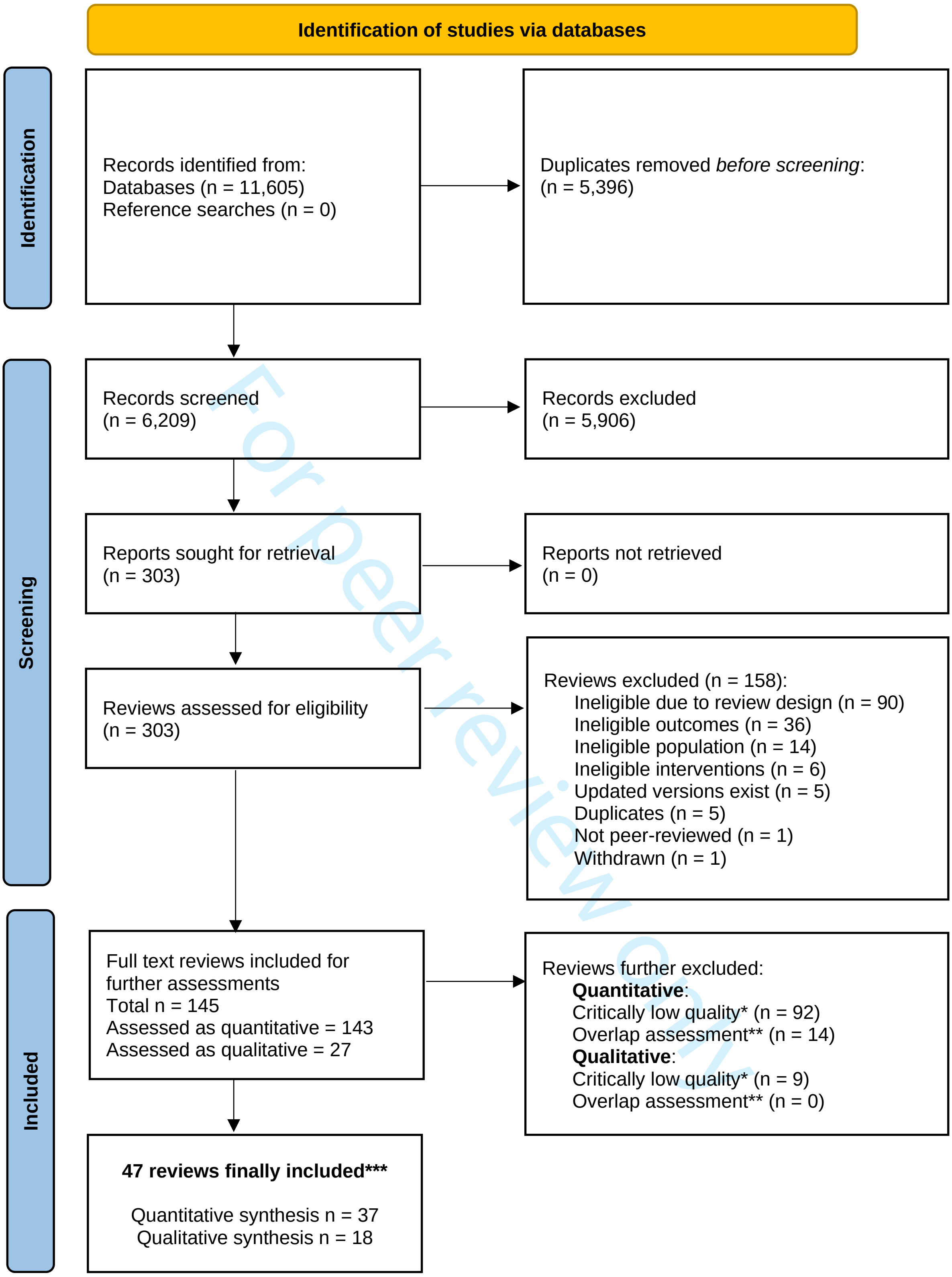

For peer review only

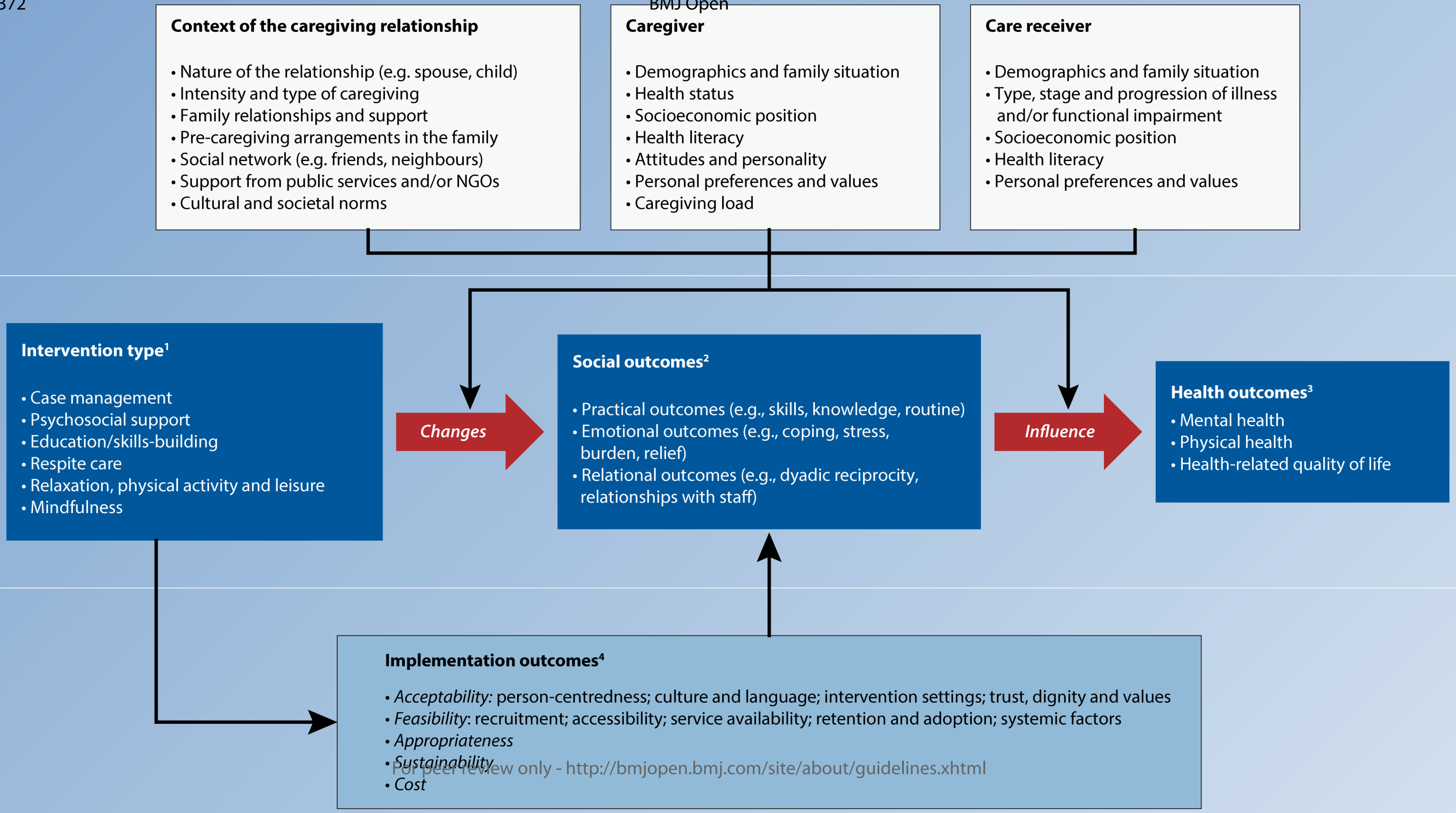

For peer review only - http://bmjopen.bmj.com/site/about/guidelines.xhtml

Supplementary materials

These supplementary materials are provided by the authors to give readers additional information about their study “Effectiveness of interventions designed to mitigate the negative health outcomes of informal caregiving to older adults: an umbrella review of systematic reviews and meta-analyses”.

For peer review only

Table of Contents

Supplementary Table 1. Amendments made to the published protocol 3

Supplementary Table 2. Search strategies 4

Supplementary Table 3. Exclusion decisions based on high overlap among quantitative reviews 8

Supplementary Table 4. Application of Hendricks et al.'s mega-aggregation framework synthesis method 11

Supplementary Table 5a. AMSTAR-2 quality assessment tool for quantitative reviews 12

Supplementary Table 5b. *Ad hoc* quality assessment tool for qualitative reviews 13

Supplementary Table 6. Repository of all included reviews (*n*=47) 14

Supplementary Table 7. Summary of findings for interventions involving case management 25

Supplementary Table 8. Summary of findings for psychosocial, psychoeducational, and skills-building interventions 26

Supplementary Table 9. Summary of findings for interventions involving respite care 29

Supplementary Table 10. Summary of findings for interventions involving relaxation, physical activity, or leisure 30

Supplementary Table 11. Summary of findings for interventions involving mindfulness-based activities 31

Supplementary Table 12. Coding table for qualitative data 32

Additional supplementary materials not included within this document:

- Supplementary File 1 : Excel spreadsheet for GROOVE citation assessment tool: quantitative reviews
- Supplementary File 2 : Excel spreadsheet for GROOVE citation assessment tool: qualitative reviews

For peer review only

Supplementary Table 1. Amendments made to the published protocol (doi:10.1136/bmjopen-2021-053117)

Amendment	Rationale
**Research question 4**<br>*Protocol:* (4) How are the proposed interventions experienced by caregivers in terms of their acceptability, feasibility and added value?<br>*Amended:* (4) How are the proposed interventions experienced by caregivers in terms of effectiveness and implementation outcomes?	We amended the fourth research question based on a data-driven approach to encompass a wider range of implementation outcomes. During the qualitative data extraction and categorization exercise, it became clear that data was richer than originally anticipated. We believe that the current formulation better addresses the complexity of intervention experiences, as reported by caregivers.
**Timeframe**<br>*Protocol:* the review is anticipated to be conducted in the period of 1 April 2021– 31 May 2022.	Due to the volume of work required to finalize the umbrella review, we did not fit in the anticipated timeframe. Instead, the final draft of the report was completed in late June of 2022.
**Exclusion criteria**<br>*Amended:* we excluded reviews of critically low quality, as assessed by AMSTAR-2 or the *ad hoc* qualitative tool.<br>*Amended:* we excluded reviews from review pairs with high overlap, as assessed by the Corrected Covered Area methodology.	We introduced two additional exclusion criteria related to the quality of reviews and overlap between reviews. Due to an abundance of reviews of, on average, low quality based on AMSTAR-2, we decided to exclude reviews of critically low quality, thus increasing the certainty of the evidence we subsequently synthesized. Overlap exclusion decisions were made in a data-driven approach, based on the assessment of primary study overlap. We excluded overlapping reviews to avoid double counting and overrepresentation of certain types of studies.
**Data sources and search strategy**<br>*Protocol:* we will use CoCites citation-based search tool to widen the net and retrieve articles that cite eligible systematic reviews. […] Additional searches will be developed for syntheses of effectiveness, implementation or process evaluations published or mandated by official health agencies. We will contact the first and last authors of selected reviews to retrieve grey literature that may otherwise have been missed.<br>*Amended:* we performed a manual search of reference lists of included reviews. The rest of the steps were not undertaken.	Our search strategies led to an overwhelming number of reviews on the topic. Due to an abundance and overrepresentation of available evidence, we decided to be pragmatic and not use any citation search tool. We did not contact experts of official health agencies since the volume of peer-reviewed evidence was already sufficient.
**Change in AMSTAR-2 assessment**<br>Item 10 (*Did the review authors report on the sources of funding for the studies included in the review?*) was not considered as critical.	The team made a consensus-based decision not to include item 10 of AMSTAR-2 as a critical item since this information was exclusively provided by Cochrane reviews. Considering this item as critical would have significantly limited the inclusion of reviews in our umbrella review. In addition, the team considered this criterion too stringent since this is not common practice in non-Cochrane, non-clinical reviews, especially in the field of public health intervention research.
**Change in *ad hoc* quality assessment tool for qualitative reviews**	The team decided to exclude critically low-quality reviews from the qualitative batch as well. Therefore, based on internal consensus, items 3,4,7,10 of the *ad hoc* quality assessment tool were designated as critical, and reviews with more than two critical flaws were excluded.
**Qualitative synthesis methods**<br>*Protocol:* Evidence from syntheses of qualitative research will also be presented in tabular format. Results will be synthesized and illustrated by using verbatim replications from the source review where appropriate. Findings will also be described and interpreted in the research team's own words. Caregivers' experiences and views on barriers to and facilitators of interventions will be identified in terms of acceptability, feasibility and added value (research question 4). The diversity of caregivers, care receivers and/or care contexts will be considered as far as possible.<br>*Amended:* For qualitative reviews, we followed the 'mega-aggregation framework' synthesis method, which has been described elsewhere. First, the extracted verbatim texts from reviews were coded line-by-line (complete coding) by two team members. Codes were then iteratively and deductively categorized based on the adapted version of van Houtven et al.'s framework. Throughout the review, we refer to verbatim texts extracted from reviews and their corresponding codes as third-order constructs, while primary studies inform second-order constructs, and the communication from participants (i.e., caregivers) is considered as first-order construct. Thus, the themes, categories, and sub-categories that we generated based on third-order constructs (i.e., reviews) are termed as fourth-order constructs. In case our fourth-order codes did not fit fully into the framework, they were inductively categorized into new themes. Codes referring to care receivers or staff opinions were removed. A third investigator checked the outcome of the categorization independently, and several consensus meetings were held to resolve disagreements and finalize the synthesis of qualitative materials.	In addition to amending research question 4, methods for synthesizing qualitative data were further developed. Instead of simply providing a narrative summary with verbatim quotes, we undertook a mega-aggregation approach, complemented by "best-fit" framework synthesis. We believe that these methods are better suited for synthesizing review-level qualitative data.

For peer review only

For peer review only - http://bmjopen.bmj.com/site/about/guidelines.xhtml

Supplementary Table 2. Search strategies

Databases:
1. Medline (via Ovid)
2. Web of Science Core Collection (via Clarivate)
3. PsycInfo (via Ovid)
4. Cinahl (via EBSCO)

Total number of hits: before deduplication n=11,605 / after deduplication n=6213

1. Search strategy and results in Medline

Interface: Ovid MEDLINE(R) and Epub Ahead of Print, In-Process & Other Non-Indexed Citations and Daily

Date of Search: 26 March 2021

Number of hits: 3,158

Field labels

- exp/ = exploded MeSH term
- / = non exploded MeSH term
- .ti,ab,kf. = title, abstract and author keywords
- adjx = within x words, regardless of order
- * = truncation of word for alternate endings

Database(s): **Ovid MEDLINE(R) and Epub Ahead of Print, In-Process, In-Data-Review & Other Non-Indexed Citations and Daily** 1946 to March 26, 2021

#	**Searches**	**Results**
1	exp Aged/	3219373
2	exp Aging/	250613
3	Frailty/	3780
4	(advanced age or aged or ageing or aging or elder* or frail* or geriatr* or gerontolog* or late* life or old age or old* adult* or old* client* or old* individual* or old* man or old* men or old* patient* or old* people or old* person* or old* population* or old* woman or old* women or oldest old or retired or senior*).ti,ab,kf.	1504150
5	Dementia/	53069
6	Alzheimer Disease/	97714
7	(dementia* or alzheimer*).ti,ab,kf.	230766
8	exp Neoplasms/	3433491
9	(neoplasm* or cancer*).ti,ab,kf.	2107334
10	exp Stroke/	141822
11	stroke.ti,ab,kf.	258717
12	Parkinson disease/	68962
13	parkinson*.ti,ab,kf.	124358
14	Multimorbidity/	1267
15	multimorbid*.ti,ab,kf.	5841
16	exp Heart failure/	126175
17	(heart failure or cardiac failure).ti,ab,kf.	193402
18	or/1-17	7707943
19	Caregivers/	38935
20	(caregiv* or care giv* or caretak* or care tak* or carer*).ti,ab,kf.	98680
21	((family or informal or unpaid) adj3 (care or caring)).ti,ab,kf.	17170
22	or/19-21	118701
23	(meta analysis or systematic review).pt.	214329
24	review.ti.	524072
25	systematic* review*.ab,kf.	154180
26	(meta analy* or metaanaly* or meta stud* or meta interpretation* or meta ethnograph* or meta summar* or meta synthes* or meta narrative* or mixed research synthes*).ti,ab,kf.	200564
27	((concept analy* or grounded theory) and review*).ti,ab,kf.	1518
28	or/23-27	699974
29	18 and 22 and 28	3304
30	(english or swedish or spanish or french or italian or german).lg.	29905965
31	29 and 30	3269
32	limit 31 to yr="2000 -Current"	3178
33	limit 32 to (comment or congress or editorial or letter)	20
34	32 not 33	3158

For peer review only - http://bmjopen.bmj.com/site/about/guidelines.xhtml

2. Search strategy and results in Web of Science Core Collection

Interface: Clarivate Analytics

Date of Search: 26 March 2021

Number of hits: 3,991

Field labels

- TS/Topic = title, abstract, author keywords and Keywords Plus
- NEAR/x = within x words, regardless of order
- * = truncation of word for alternate endings

# 15	3,991	**#11 AND #6 AND #3**<br>**Refined by: [excluding] DOCUMENT TYPES: (MEETING ABSTRACT OR BOOK REVIEW OR PROCEEDINGS PAPER OR EDITORIAL MATERIAL OR LETTER) AND PUBLICATION YEARS: (2021 OR 2013 OR 2005 OR 2020 OR 2012 OR 2004 OR 2019 OR 2011 OR 2003 OR 2018 OR 2010 OR 2002 OR 2017 OR 2009 OR 2001 OR 2016 OR 2008 OR 2000 OR 2015 OR 2007 OR 2014 OR 2006) AND LANGUAGES: (ENGLISH OR GERMAN OR SPANISH OR ITALIAN OR FRENCH OR SWEDISH)**<br>*Indexes=SCI-EXPANDED, SSCI, A&HCI, CPCI-S, CPCI-SSH, ESCI Timespan=All years*
# 14	4,007	**#11 AND #6 AND #3**<br>**Refined by: [excluding] DOCUMENT TYPES: (MEETING ABSTRACT OR BOOK REVIEW OR PROCEEDINGS PAPER OR EDITORIAL MATERIAL OR LETTER) AND PUBLICATION YEARS: (2021 OR 2013 OR 2005 OR 2020 OR 2012 OR 2004 OR 2019 OR 2011 OR 2003 OR 2018 OR 2010 OR 2002 OR 2017 OR 2009 OR 2001 OR 2016 OR 2008 OR 2000 OR 2015 OR 2007 OR 2014 OR 2006)**<br>*Indexes=SCI-EXPANDED, SSCI, A&HCI, CPCI-S, CPCI-SSH, ESCI Timespan=All years*
# 13	4,075	**#11 AND #6 AND #3**<br>**Refined by: [excluding] DOCUMENT TYPES: (MEETING ABSTRACT OR BOOK REVIEW OR PROCEEDINGS PAPER OR EDITORIAL MATERIAL OR LETTER)**<br>*Indexes=SCI-EXPANDED, SSCI, A&HCI, CPCI-S, CPCI-SSH, ESCI Timespan=All years*
# 12	4,262	**#11 AND #6 AND #3**<br>*Indexes=SCI-EXPANDED, SSCI, A&HCI, CPCI-S, CPCI-SSH, ESCI Timespan=All years*
# 11	1,062,768	**#10 OR #9 OR #8 OR #7**<br>*Indexes=SCI-EXPANDED, SSCI, A&HCI, CPCI-S, CPCI-SSH, ESCI Timespan=All years*
# 10	2,084	**TS=(("concept analy*" or "grounded theory") and review*)**<br>*Indexes=SCI-EXPANDED, SSCI, A&HCI, CPCI-S, CPCI-SSH, ESCI Timespan=All years*
# 9	393,878	**TS=("meta analy*" or metaanaly* or "meta stud*" or "meta interpretation*" or "meta ethnograph*" or "meta summar*" or "meta synthes*" or "meta narrative*" or "mixed research synthes*")**<br>*Indexes=SCI-EXPANDED, SSCI, A&HCI, CPCI-S, CPCI-SSH, ESCI Timespan=All years*
# 8	227,798	**TS="systematic* review*"**<br>*Indexes=SCI-EXPANDED, SSCI, A&HCI, CPCI-S, CPCI-SSH, ESCI Timespan=All years*
# 7	732,777	**TI=review**<br>*Indexes=SCI-EXPANDED, SSCI, A&HCI, CPCI-S, CPCI-SSH, ESCI Timespan=All years*
# 6	131,791	**#5 OR #4**<br>*Indexes=SCI-EXPANDED, SSCI, A&HCI, CPCI-S, CPCI-SSH, ESCI Timespan=All years*
# 5	23,282	**TS=(("family" or "informal" or "unpaid") NEAR/3 ("care" or "caring"))**<br>*Indexes=SCI-EXPANDED, SSCI, A&HCI, CPCI-S, CPCI-SSH, ESCI Timespan=All years*
# 4	115,945	**TS=(caregiv* or "care giv*" or caretak* or "care tak*" or carer*)**<br>*Indexes=SCI-EXPANDED, SSCI, A&HCI, CPCI-S, CPCI-SSH, ESCI Timespan=All years*
# 3	5,155,246	**#2 OR #1**<br>*Indexes=SCI-EXPANDED, SSCI, A&HCI, CPCI-S, CPCI-SSH, ESCI Timespan=All years*
# 2	3,790,895	**TS=(alzheimer* or cancer* or "cardiac failure" or dementia* or "heart failure" or multimorbid* or n eoplasm* or parkinson* or "stroke")**<br>*Indexes=SCI-EXPANDED, SSCI, A&HCI, CPCI-S, CPCI-SSH, ESCI Timespan=All years*
# 1	1,658,624	**TS=("advanced age" or "aged" or "ageing" or "aging" or elder* or frail* or geriatr* or gerontolog* o r "late* life" or "old age" or "old* adult*" or "old* client*" or "old* individual*" or "old* man" or " old* men" or "old* patient*" or "old* people" or "old* person*" or "old* population*" or "old* wo man" or "old* women" or "oldest old" or "retired" or senior*)**<br>*Indexes=SCI-EXPANDED, SSCI, A&HCI, CPCI-S, CPCI-SSH, ESCI Timespan=All years*

For peer review only - http://bmjopen.bmj.com/site/about/guidelines.xhtml

3. Search strategy and results in PsycInfo

Interface: Ovid

Date of Search: 26 March 2021

Number of hits: 1,933

Field labels

- exp/ = exploded controlled term
- / = non exploded controlled term
- .ti,ab,id. = title, abstract and author keywords
- adjx = within x words, regardless of order
- * = truncation of word for alternate endings

Database(s): **APA PsycInfo** 1806 to March Week 3 2021

#	**Searches**	**Results**
1	Older Adulthood/	6645
2	Geriatric patients/	13619
3	exp Aging/	77653
4	(advanced age or aged or ageing or aging or elder* or frail* or geriatr* or gerontolog* or late* life or old age or old* adult* or old* client* or old* individual* or old* man or old* men or old* patient* or old* people or old* person* or old* population* or old* woman or old* women or oldest old or retired or senior*).ti,ab,id.	470458
5	Dementia/ or exp Presenile dementia/	76186
6	(dementia* or alzheimer*).ti,ab,id.	104258
7	exp Neoplasms/	54065
8	(neoplasm* or cancer*).ti,ab,id.	65816
9	Cerebrovascular accidents/	21623
10	stroke.ti,ab,id.	34513
11	exp Parkinson's disease/	25813
12	parkinson*.ti,ab,id.	35346
13	Comorbidity/	34018
14	multimorbid*.ti,ab,id.	1051
15	(heart failure or cardiac failure).ti,ab,id.	4153
16	or/1-15	686012
17	Caregivers/	30033
18	Elder care/	4896
19	(caregiv* or care giv* or caretak* or care tak* or carer*).ti,ab,id.	73654
20	((family or informal or unpaid) adj3 (care or caring)).ti,ab,id.	10822
21	17 or 18 or 19 or 20	85126
22	review.ti.	158155
23	systematic* review*.ab,id.	28501
24	(meta analy* or metaanaly* or meta stud* or meta interpretation* or meta ethnograph* or meta summar* or meta synthes* or meta narrative* or mixed research synthes*).ti,ab,id.	41992
25	((concept analy* or grounded theory) and review*).ab,id.	1439
26	22 or 23 or 24 or 25	196607
27	16 and 21 and 26	2148
28	(english or swedish or spanish or french or italian or german).lg.	4774945
29	27 and 28	2064
30	limit 29 to yr="2000 -Current"	1950
31	(editorial or letter).dt.	67669
32	30 not 31	1933

For peer review only

4. Search strategy and results in Cinahl

Interface: Ebsco

Date of Search: 26 March 2021

Number of hits: 2,523

Field labels

- MH+ = exploded Cinahl Heading
- MH = non exploded Cinahl Heading
- TI = title
- AB = abstract
- Nx = within x words, regardless of order
- * = truncation of word for alternate endings

#	Query	Results
S31	S29 NOT S30	2,523
S30	PT Commentary or Editorial or Letter or Proceedings	854,425
S29	S17 AND S21 AND S28<br><br>Limiters - Published Date: 20000101-20211231;<br>Language: English, French, German, Italian, Swedish	2,590
S28	S22 OR S23 OR S24 OR S25 OR S26 OR S27	294,864
S27	AB (("concept analy*" or "grounded theory") and review*)	1,376
S26	TI ("meta analy*" or metaanaly* or "meta stud*" or "meta interpretation*" or "meta ethnograph*" or "meta summar*" or "meta synthes*" or "meta narrative*" or "mixed research synthes*") OR AB ("meta analy*" or metaanaly* or "meta stud*" or "meta interpretation*" or "meta ethnograph*" or "meta summar*" or "meta synthes*" or "meta narrative*" or "mixed research synthes*")	80,611
S25	AB "systematic* review*"	67,592
S24	TI review	206,732
S23	PT (meta analysis or meta synthesis)	39,376
S22	PT systematic review	110,054
S21	S18 OR S19 OR S20	100,197
S20	TI (((family or informal or unpaid) N3 (care or caring))) OR AB (((family or informal or unpaid) N3 (care or caring)))	21,928
S19	TI (caregiv* or "care giv*" or caretak* or "care tak*" or carer*) OR AB (caregiv* or "care giv*" or caretak* or "care tak*" or carer*)	74,717
S18	(MH "Caregivers")	36,859
S17	S1 OR S2 OR S3 OR S4 OR S5 OR S6 OR S7 OR S8 OR S9 OR S10 OR S11 OR S12 OR S13 OR S14 OR S15 OR S16	1,828,415
S16	TI ("heart failure" or "cardiac failure") OR AB ("heart failure" or "cardiac failure")	55,501
S15	(MH "Heart Failure+")	43,530
S14	TI multimorbid* OR AB multimorbid*	2,720
S13	(MH "Comorbidity")	64,158
S12	TI parkinson* OR AB parkinson*	27,885
S11	(MH "Parkinson Disease")	22,600
S10	TI stroke OR AB stroke	96,983
S9	(MH "Stroke+")	72,157
S8	TI (neoplasm* or cancer*) OR AB (neoplasm* or cancer*)	424,414
S7	(MH "Neoplasms+")	570,301
S6	TI (dementia* or alzheimer*) OR AB (dementia* or alzheimer*)	77,937
S5	(MH "Dementia") OR (MH "Dementia, Presenile+") OR (MH "Dementia, Senile+")	73,337
S4	TI (("advanced age" or aged or ageing or aging or elder* or frail* or geriatr* or gerontolog* or "late* life" or "old age" or "old* adult*" or "old* client*" or "old* individual*" or "old* man" or "old* men" or "old* patient*" or "old* people" or "old* person*" or "old* population*" or "old* woman" or "old* women" or "oldest old" or retired or senior*)) OR AB (("advanced age" or aged or ageing or aging or elder* or frail* or geriatr* or gerontolog* or "late* life" or "old age" or "old* adult*" or "old* client*" or "old* individual*" or "old* man" or "old* men" or "old* patient*" or "old* people" or "old* person*" or "old* population*" or "old* woman" or "old* women" or "oldest old" or retired or senior*))	452,573
S3	(MH "Frailty Syndrome")	2,612
S2	(MH "Aging+")	56,337
S1	(MH "Aged+")	860,457

For peer review only - http://bmjopen.bmj.com/site/about/guidelines.xhtml

Supplementary Table 3. Exclusion decisions based on high overlap among quantitative reviews*

Overlapping clusters (% of overlap)**	Excluded reviews	Rationale for the exclusion of overlaping reviews
Case management: excluded 2 out of 12 reviews		
Cheng 2020 vs Weinbrecht 2016: 24.8%<br>Lee 2020 vs Weinbrecht 2016: 20.5%	Weinbrecht 2016	Weinbrecht did not discuss findings on case management; we identified the category from the summary of included studies table. Lee and Cheng both discussed case management findings Therefore, we decided to exclude Weinbrecht from this category.
Corvol 2017 vs Reilly 2015: 31.8%<br>Reilly 2015 vs Goeman 2016: 15.4%	Corvol 2017	Corvol and Reilly both focused on case management. Reilly was a Cochrane review and better structured for the purposes of this umbrella review. Therefore, we decided to exclude Corvol from this category. Goeman focused on support worker role, which made the review sufficiently different form others.
Psychosocial interventions: excluded 13 out of 35 reviews		
Fu 2017 vs Gabriel 2020: 22.2%	Fu 2017	Fu and Gabriel both focused on caregivers of people with cancer. The quality of the reviews was similar. We decided to include Gabriel since it is more recent.
Hopwood 2018 vs Sin 2018: 22.1%<br>Hopwood 2018 vs Boots 2014: 38.9%<br>Hopwood 2018 vs Egan 2018: 20%<br>Boots 2014 vs Egan 2018: 20%<br>Egan 2018 vs Zhao 2019: 55.6%<br>Hopwood 2018 vs Zhao 2019: 13.5%<br>Hopwood 2018 vs Leng 2020: 24.3%	Boots 2014<br>Egan 2018<br>Zhao 2019	Hopwood, Sin, Boots, Egan, Zhao and Leng all concerned internet-based interventions. All but Sin focused on dementia caregivers, so Sin was sufficiently different. Leng was recent (2020) and included meta-analysis. Egan and Zhao shared 55.6% of studies and were very similar, the only difference being the publication date. Between Hopwood and Boots, Hopwood was more recent. Between Hopwood and Zhao, Hopwood was more informative since it included 36 studies (Zhao includes 6 studies and 5 of them are also included by Hopwood). From this cluster, we decided to include Hopwood, Sin and Leng.
Egan 2018 vs Gonzalez-Fraile 2021: 22.7%<br>Gonzalez-Fraile 2021 vs Lins 2014: 21.1%<br>Gonzalez-Fraile 2021 vs Zhao 2019: 23.8%<br>Gonzalez-Fraile 2021 vs Leng 2020: 34.5%<br>Gonzalez-Fraile 2021 vs Lucero 2019: 15.8%	Leng 2020<br>Lucero 2019	Gonzalez-Fraile was the most recent (2021) and a Cochrane review. We decided to exclude Egan (already excluded above), Leng, Zhao (already excluded above) and Lucero (similar topic, no meta-analysis). We included Lins because of narrower focus (concerns telephone-based interventions specifically).
Lee 2020 vs Amador-Marin 2017: 25%<br>Lee 2020 vs Weinbrecht 2016: 20.5%<br>Lee 2020 vs Teahan 2020: 17.1%	Amador-Marin 2017<br>Weinbrecht 2016	Lee and Tehan were most recent, each had more than 20 RCTs, and overlap between them was low. Thus, we decided to exclude Amador-Marin and Weinbrecht and instead include Teahan and Lee.
Cheng 2020 vs Weinbrecht 2016: 24.8%<br>Cheng 2020 vs Wiegelmann 2021: 23.8%<br>Cheng 2020 vs Vandepitte 2016: 16.7%<br>Cheng 2020 vs Lins 2014: 7.3%<br>Cheng 2020 vs Zabihi 2020: 5%<br>Cheng 2020 vs Akarsu 2019: 5.1%<br>Cheng 2020 vs Hopkinson 2019: 6.8%<br>Cheng 2020 vs Jensen 2015: 7.1%<br>Cheng 2020 vs Reilly 2015: 6.7%<br>Cheng 2020 vs Teahan 2020: 14.8%	Cheng 2020<br>Vandepitte 2016<br>Weinbrecht 2016	Cheng and Wiegelmann were the largest and most recent reviews. However, the overlap between Cheng and most of the smaller reviews was substantial (9 smaller reviews overlapped moderately or highly with Cheng). Therefore, we decided to keep Wiegelmann instead of Cheng, and included the rest of smaller overlapping reviews. In addition, we decided to exclude Vandepitte from this cluster. Weinbrecht was already excluded in the previous cluster.

For peer review only - http://bmjopen.bmj.com/site/about/guidelines.xhtml

Cheng 2020 vs GonzalezFraile 2021: 10.7%<br>Cheng 2020 vs Amador-Marin 2017: 7.8%<br>Cheng 2020 vs Lucero 2019: 5.1%<br>Cheng 2020 vs Piersol 2017: 11.5%		
Egan 2018 vs Guay 2017: 17.6%<br>Egan 2018 vs Wang 2020: 25%<br>Egan 2018 vs Leng 2019: 50%<br>Zhao 2019 vs Leng 2019: 50%<br>Leng 2019 vs Guay 2017: 23.5%<br>Zhao 2019 vs Guay 2017: 28.6%<br>Zhao 2019 vs Wang 2020: 30%<br>Guay 2017 vs Wang 2020: 18.8%<br>Guay 2017 vs Sin 2018: 13%	Guay 2017	Zhao, Egan and Leng were already excluded in previous clusters. Between Guay and Sin, we decided to keep Sin, since it had 78 studies, while Guay included only 12. We included Wang from this cluster.
Lins 2014 vs Lucero 2019: 15.8%	Lucero 2019	We already excluded Lucero in previous clusters. Lins was included.
Hopkinson 2019 vs Vernooij-Dassen 2011: 26.3%	Vernooij-Dassen 2011	Between Hopkinson and Vernooij-Dassen, both had meta-analysis and both were about Cognitive Behavioral Therapy exclusively. We decided to include Hopkinson, since it was more recent (2019 vs 2011) and had more studies.
Jensen 2015 vs Wiegelmann 2021: 25%<br>Teahan 2020 vs Wiegelmann 2021: 24.5%<br>Weinbrecht 2016 vs Wiegelmann 2021: 18.4%<br>Jensen 2015 vs Teahan 2020: 14.3%	Jensen 2015<br>Weinbrecht 2016	We already excluded Weinbrecht in previous clusters. Between Jensen and Teahan, Teahan was more recent. Thus, we additionally excluded Jensen from this cluster.
Legg 2011 vs Minshall 2019: 19%	Legg 2011	Between Legg and Minshall, we decided to include Minshall since it was more recent (2019 vs 2011) and included more studies (31 vs 8).
Education and Skills building: excluded 12 out of 32 reviews		
Legg 2011 vs Smith 2019: 22.2%<br>Pucciarelli 2020 vs Smith 2019: 22.2%<br>Legg 2011 vs Minshall 2019: 19%	Legg 2011	Smith had a wider focus, while Legg and Pucciarelli focused on caregivers of patients with stroke. Since the latter two did not have high enough overlap, we decided to include all three reviews. Between Legg and Minshall, we decided to include Minshall only since it was more recent (2019 vs 2011) and included more studies (31 vs 8).
Fu 2017 vs Gabriel 2020: 22.2%	Fu 2017	Fu and Gabriel both focused on caregivers of people with cancer. The quality of the reviews was similar. We decided to include Gabriel since it was more recent.
Hopwood 2018 vs Sin 2018: 22.1%<br>Hopwood 2018 vs Boots 2014: 38.9%<br>Hopwood 2018 vs Egan 2018: 20%<br>Boots 2014 vs Egan 2018: 20%<br>Egan 2018 vs Zhao 2019: 55.6%<br>Hopwood 2018 vs Zhao 2019: 13.5%<br>Hopwood 2018 vs Leng 2020: 24.3%	Boots 2014<br>Egan 2018<br>Zhao 2019	Hopwood, Sin, Boots, Egan, Zhao and Leng all concerned internet-based interventions. All but Sin focused on dementia caregivers, so Sin was sufficiently different. Leng was recent (2020) and included meta-analysis. Egan and Zhao shared 55.6% of studies and were very similar, the only difference being the publication date. Between Hopwood and Boots, Hopwood was more recent. Between Hopwood and Zhao, Hopwood was more informative since it included 36 studies (Zhao includes 6 studies and 5 of them were also included by Hopwood). From this cluster, we decided to include Hopwood, Sin and Leng.
Egan 2018 vs Gonzalez-Fraile 2021: 22.7%<br>Gonzalez-Fraile 2021 vs Lins 2014: 21.1%<br>Gonzalez-Fraile 2021 vs Zhao 2019: 23.8%	Leng 2020<br>Lucero 2019	Gonzalez-Fraile was the most recent (2021) and a Cochrane review. We decided to exclude Egan (already excluded above), Leng, Zhao (already excluded above) and Lucero (similar topic, no meta-

For peer review only - http://bmjopen.bmj.com/site/about/guidelines.xhtml
For peer review only

Gonzalez-Fraile 2021 vs Leng 2020: 34.5%<br>Gonzalez-Fraile 2021 vs Lucero 2019: 15.8%		analysis). We included Lins because of a narrower focus (concerns telephone-based interventions specifically).
Lee 2020 vs Amador-Marin 2017: 25%<br>Lee 2020 vs Weinbrecht 2016: 20.5%<br>Lee 2020 vs Teahan 2020: 17.1%	Amador-Marin 2017<br>Weinbrecht 2016	Lee and Tehan were most recent, each had more than 20 RCTs, and overlap between them was low. Thus, we decided to exclude Amador-Marin and Weinbrecht and instead include Teahan and Lee.
Cheng 2020 vs Weinbrecht 2016: 24.8%<br>Cheng 2020 vs Wiegelmann 2021: 23.8%<br>Cheng 2020 vs Vandepitte 2016: 16.7%<br>Cheng 2020 vs Lins 2014: 7.3%<br>Cheng 2020 vs Zabihi 2020: 5%<br>Cheng 2020 vs Akarsu 2019: 5.1%<br>Cheng 2020 vs Hopkinson 2019: 6.8%<br>Cheng 2020 vs Jensen 2015: 7.1%<br>Cheng 2020 vs Reilly 2015: 6.7%<br>Cheng 2020 vs Teahan 2020: 14.8%<br>Cheng 2020 vs GonzalezFraile 2021: 10.7%<br>Cheng 2020 vs Amador-Marin 2017: 7.8%<br>Cheng 2020 vs Lucero 2019: 5.1%<br>Cheng 2020 vs Piersol 2017: 11.5%	Cheng 2020<br>Vandepitte 2016	Cheng and Wiegelmann were the largest and most recent reviews. However, the overlap between Cheng and most of the smaller reviews was substantial (9 smaller reviews overlapped moderately or highly with Cheng). Therefore, we decided to keep Wiegelmann instead of Cheng, and included the rest of smaller overlapping reviews. In addition, we decided to exclude Vandepitte from this cluster. Therefore, we decided to exclude Weinbrecht (already excluded above) and Vandepitte (already excluded above) from this cluster.
Egan 2018 vs Guay 2017: 17.6%<br>Egan 2018 vs Leng 2019: 50%<br>Zhao 2019 vs Leng 2019: 50%<br>Leng 2019 vs Guay 2017: 23.5%<br>Zhao 2019 vs Guay 2017: 28.6%<br>Guay 2017 vs Sin 2018: 13%	Guay 2017	Zhao, Egan and Leng were already excluded in previous clusters. Between Guay and Sin, we decided to keep Sin, since it had 78 studies, while Guay included only 12.
Lins 2014 vs Lucero 2019: 15.8%		We already excluded Lucero in previous clusters. Lins was included.
Jensen 2015 vs Wiegelmann 2021: 25%<br>Teahan 2020 vs Wiegelmann 2021: 24.5%<br>Weinbrecht 2016 vs Wiegelmann 2021: 18.4%<br>Jensen 2015 vs Teahan 2020: 14.3%	Jensen 2015	We already excluded Weinbrecht in previous clusters. Between Jensen and Teahan, Teahan was more recent. Thus, we additionally excluded Jensen from this cluster.
Respite care: excluded 1 out of 6 reviews		
Shaw 2009 vs Mason 2007: 21.7%	Mason 2007	Shaw was more recent and included more studies (104 vs 22). Thus, we decided to exclude Mason from this cluster.
Relaxation, physical activity, and leisure: excluded 1 out of 7 reviews		
Cheng 2020 vs Wiegelmann 2021: 23.8%	Wiegelmann 2021	Cheng and Wiegelmann were the largest and most recent reviews. Cheng included more studies on the topic, so we decided to exclude Wiegelmann from this cluster.
Mindfulness: excluded 1 out of 6 reviews		
Liu 2018 vs Collins 2019: 30.8%<br>Liu 2018 vs Shim 2020: 18.2%	Collins 2019	All three reviews concerned mindfulness interventions for caregivers of people with dementia. Liu was a Cochrane review (but only included 5 studies), Shim included 20 studies. We decided to exclude Collins from this cluster.

*The number of qualitative reviews in each category was low and overlapping clusters were limited. Due to the scarcity of qualitative evidence and low overlap, we decided not to exclude any qualitative review based on this approach. For full information about overlap among qualitative reviews, see the Citation Matrix in Supplementary File 2.
**We assessed clusters of reviews with very high (33 pairs), high (40 pairs) or moderate overlap (only if one is almost fully included in the other); see the Citation Matrix in Supplementary File 1 for the overlap among all included reviews after quality assessment.

For peer review only - http://bmjopen.bmj.com/site/about/guidelines.xhtml

For peer review only

Supplementary Table 4. Application of Hendricks et al.'s mega-aggregation framework synthesis method[1]

**Step 1: Identify a clearly defined review question and objectives**		
This umbrella review aims to explore the effectiveness of interventions targeting informal caregivers' physical and mental health. The fourth objective, which is the one mainly feeding on qualitative evidence, is as follows: how are the proposed interventions experienced by caregivers in terms of effectiveness and implementation outcomes?		
**Step 2: Identify a theoretical framework or model**		
Adapted version of Van Houtven et al.'s framework[2,3]	Intervention, Context of caregiving relationship, Caregiver, Care receiver, Caregiver knowledge, attitude and behaviors, Caregivers' experience of the intervention, Caregiver outcomes	The adapted framework describes the different determinants that affect the effectives of support interventions in addressing health outcomes of caregivers, and how they interact by modulating the other determinants and components
**Step 3: Decide on criteria for considering reviews for inclusion**		
Inclusion and exclusion criteria used to select reviews are presented in Table 1 of the published umbrella review protocol[3]		
**Step 4: Conduct searching and screening**		
The following databases were searched from 1 January 2000 to 26 March 2021: Medline, CINAHL, PsycINFO and Web of Science. In addition, we performed a manual search of the reference lists of included reviews	Screening was done independently and in duplicate by MK and ACL; MK and LM; MK and LS; MK and LD	
**Step 5: Conduct quality appraisal of the included studies**		
An *ad hoc* tool was used, developed and piloted by review authors	Quality appraisal was done independently and induplicate by all co-authors	Reviews of critically low quality were excluded (i.e., those that had 2 or more critical items missing or not reported); 18 reviews contributed to the next step
**Step 6: Data extraction and categorization**		
AA, ACL and MK read all reviews	AA and MK extracted the verbatims and each verified the extraction of the other co-author	Construction of fourth-order constructs (codes, categories, and aggregations) was done in consensus by AA and MK, and then reviewed by third co-author ACL. In the case of codes and categories not fitting the framework, they were inductively abstracted and new themes were generated and added to the framework. All three co-authors then reviewed and discussed the process until consensus was reached by all three co-authors. The results of the categorization and aggregation was then shared with the other co-authors and consensus was reached
**Step 7: Present and synthesize the findings**		
Two themes were constructed (intervention outcomes and implementation outcomes) using 200 codes, with further groupings of codes in categories and sub-categories	Evidence gaps and lines of actions were identified following convergence of the qualitative and quantitative findings, and were later presented in the discussion	
**Step 8: Transparent reporting**		
PRIOR[4] reporting guideline were used as far as possible. The protocol was published (doi:10.1136/bmjopen-2021-053117) and the categorization and aggregation of the third-order constructs to develop the fourth-order constructs was shown in supplementary files. Additionally, differences between the protocol and the manuscript were reported in supplementary files.		

References

1. Hendricks L, Eshun-Wilson I, Rohwer A. A mega-aggregation framework synthesis of the barriers and facilitators to linkage, adherence to ART and retention in care among people living with HIV. *Syst Rev*. 2021;10(1):1-28. doi:10.1186/s13643-021-01582-z
2. Van Houtven CH, Voils CI, Weinberger M. An organizing framework for informal caregiver interventions: Detailing caregiving activities and caregiver and care recipient outcomes to optimize evaluation efforts. *BMC Geriatr*. 2011;11. doi:10.1186/1471-2318-11-77
3. Calderón-Larrañaga A, Kirvalidze M, Dahlberg L, Sacco LB, Morin L. Effectiveness of interventions to address the negative health outcomes of informal caregiving to older adults: Protocol for an umbrella review. *BMJ Open*. 2021;11(11). doi:10.1136/bmjopen-2021-053117
4. Gates M, Gates A, Pieper D, et al. Reporting guideline for overviews of reviews of healthcare interventions: The Preferred Reporting Items for Overviews of Reviews (PRIOR) statement. *MetaArXiv*. Published online 2022. doi:10.31222/osf.io/82wau

For peer review only - http://bmjopen.bmj.com/site/about/guidelines.xhtml

Supplementary Table 5a. AMSTAR-2 quality assessment tool for quantitative reviews

High	Moderate	Low	Critically low (excluded)	
Maayan, 2014	Cheng, 2020	Amador-Marin, 2017	AlDaken, 2018	Van'tLeven, 2013
Forster, 2012	Minshall, 2019	Egan, 2018	Bergstrom, 2018	Zhu, 2021
Liu, 2018	Smith, 2019	Gabriel, 2020	Brodaty, 2003	Wang, 2017
Reilly, 2015	Gonzalez-Fraile, 2021	Heckel, 2019	Bustillo, 2018	Peacock, 2003
Vernooij-Dassen, 2011	Wang, 2020	Hopwood, 2018	Cheng, 2014	Northouse, 2010
Goeman, 2016	Akarsu, 2019	Irons, 2020	Dam, 2016	Dharmawardene, 2016
	Corry, 2019	Leng, 2020	EluvathingolJose, 2013	Brereton, 2007
	Legg, 2011	Shaw, 2009	Grandi, 2019	Liu, 2017
	Lins, 2014	Shim, 2020	Hopkinson, 2012	Thompson, 2007
	Pucciarelli, 2020	Teahan, 2020	Irani, 2020	Eldred, 2008
	Sin, 2018	Zhao, 2019	Kedia, 2020	Regan, 2012
	Lucero, 2019	Collins, 2019	Kor, 2018	Heynsbergh. 2018
	Treanor, 2019	Guay, 2017	Lee, 2007	Abrahams, 2018
		Lee, 2020	Li, 2020	Li, 2016
		Mason, 2007	Li, 2014	Jackson, 2016
		Vandepitte, 2016	Luo, 2020	You, 2012
		Waldron, 2013	Ruggiano, 2018	Lambert, 2016
		Weinbrecht, 2016	Selwood, 2007	Domingues, 2018
		Zabihi, 2020	Sherifali, 2018	Miles, 2020
		Boots, 2014	Smith, 2014	Scott, 2016
		Corvol, 2017	Spencer, 2019	Hurley, 2014
		Cuthbert, 2017	ZabaleguiYarnoz, 2008	Etxeberria, 2020
		Doyle, 2020	Bourne, 2020	Deeken, 2019
		Fu, 2017	Bunn, 2016	Hempel, 2008
		Hopkinson, 2019	Byeon, 2020	Fange, 2020
		Jensen, 2015	Caress, 2009	Lally, 2016
		Maffioletti, 2019	Cassidy, 2021	Ussher, 2009
		Parkinson, 2019	Epps, 2021	Stahl, 2016
		Bennett, 2019	Frias, 2020	Mantovan, 2010
		Wiegelmann, 2021	Kaddour, 2018	Panzeri, 2019
		Greenwood, 2016	Kaltenbaugh, 2015	Poon, 2019
		Piersol, 2017	Kwon, 2017	Pritchard, 2020
			Laver, 2017	Xu, 2020
			Leung, 2017	Ahn, 2020
			Lorca-Cabrera, 2020	Chien, 2011
			Mason, 2007	Cooper, 2007
			McKechnie, 2014	Corbett, 2012
			Pinquart, 2006	Du Preez, 2018
			Pleasant, 2020	Elvish, 2013
			Pusey, 2001	Kleine, 2019
			Smits, 2007	Lee, 2020
			Vandepitte, 2016	Mason, 2008
			Verreault, 2021	MoleroJurado, 2020
			Waller, 2017	Moon, 2013
			Walter, 2020	Moore, 2020
			Wu, 2019	O'Toole, 2017

The definitions of each quality category, critical items and recommendations on grading are available from the original publication: Shea BJ, Reeves BC, Wells G, et al. AMSTAR 2: A critical appraisal tool for systematic reviews that include randomised or non-randomised studies of healthcare interventions, or both. BMJ. 2017;358:j4008. doi:10.1136/bmj.j4008.

For peer review only

Supplementary Table 5b. ***Ad hoc*** **quality assessment tool* for qualitative reviews**

Qualitative reviews with 2 or more critical flaws**	Qualitative reviews less than 2 critical flaw**
Jackson, 2016	Parkinson, 2019
Sousa, 2016	Sin, 2018
Hempel, 2008	Goeman, 2016
Armstrong, 2019	Li, 2016
Elvish, 2013	Miles, 2020
Moon, 2013	Boots, 2014
Caress, 2009	Du Preez, 2018
EluvathingolJose, 2013	Lins, 2014
Heckel, 2019	Maffioletti, 2019
	Pritchard, 2020
	Bourne, 2020
	Bunn, 2016
	AlDaken, 2018
	Dam, 2016
	Hopwood, 2018
	Irons, 2020
	Shaw, 2009
	Smith, 2014

*The tool can be accessed in the published protocol (doi:10.1136/bmjopen-2021-053117).
**Critical flaws included: transparency and comprehensiveness of the literature search (item #3), using at least two databases for the search (item #4), assessment of methodological quality of primary studies using explicit criteria (item #7), involvement of two or more researchers in data synthesis process (item #11).

For peer review only

Supplementary Table 6. Repository of all included reviews (*n*=47)

Author, year	Countries of primary studies	Study design	Databases searched	End date of literature search	# studies included in the umbrella review	# participants, mean age, % female	Intervention type	Disease of care receiver	Health outcomes of caregiver	Review type and synthesis method	Quantitative, qualitative, or mixed-methods
Akarsu, 2019[1]	US	RCTs	Embase, cinahl, medline, psycinfo, psycarticles, assia, central, sociological abstracts, spp	2015	13	2056 caregivers, mean age 59 (SD=11.36), 86% female	Psychosocial, Education and skills	Dementia	Depression	Systematic review with meta-analysis	Quantitative
Aldaken, 2018[2]	UK, China, Iran	RCTs, incl. some with qualitative interviews	Ebsco, google scholar, science direct	2017	1	NA	Mindfulness	Cancer	NA	Systematic review	Qualitative
Bennett, 2019[3]	US, Germany, Netherlands, UK, Hong Kong, Australia, Brazil	RCTs	Medline, embase, central, cumulative index to nursing and allied health literature, psycinfo, education resources information Centre, otseeker, pedro, clinical trial registries	Feb-2018	15	2063 dyads, mean age 65, 20%-90% of caregivers were spouses	Education and skills	Dementia	Emotional distress, HR-QoL	Systematic review with meta-analysis	Quantitative
Boots, 2014[4]	NA	Any	Pubmed, psycinfo, cinahl, web of science, cochrane library	Jan-2013	4	NA	Psychosocial, Education and skills	Dementia	NA	Systematic review	Qualitative
Bourne, 2020[5]	UK, US, Australia, New Zealand	Qualitative and mixed method	Psycinfo, medline, web of science, assia, google scholar	Mar-2020	10	NA	Relaxation and leisure	Dementia	NA	Systematic review	Qualitative
Bunn, 2016[6]	UK	Any	Medline, cinahl, scopus, nhs evidence, cochrane library, google scholar	Nov-2012	5	NA	Case management, Psychosocial, Education and skills	Dementia	NA	Systematic review	Qualitative

For peer review only - http://bmjopen.bmj.com/site/about/guidelines.xhtml

Author, year	Countries of primary studies	Study design	Databases searched	End date of literature search	# studies included in the umbrella review	# participants, mean age, % female	Intervention type	Disease of care receiver	Health outcomes of caregiver	Review type and synthesis method	Quantitative, qualitative, or mixed-methods
Cheng, 2020[7]	US, Australia, Brazil, Denmark, Norway, UK, Spain, Greece, Finland, India, Netherlands, Germany, Egypt, Japan, Iran, Canada, Taiwan	RCTs, quasi-experimental	Psycinfo, medline, cinahl	Mid-2018	37	NA	Relaxation and leisure, Mindfulness	Dementia	Depression, anxiety, HR-QoL, physical health	Systematic review with meta-analysis	Quantitative
Corry, 2019[8]	NA	RCTs, quasi-experimental	Central, medline, embase, psycinfo, proquest, cinahl	Nov-2018	21	1690 caregivers, age range 19-87, >70.5% female	Psychosocial, Education and skills	Any	HR-QoL, psychological health (depression, anxiety), physical health	Systematic review with meta-analysis	Quantitative
Cuthbert, 2017[9]	NA	RCTs, quasi-experimental	Medline, pubmed, cinahl	Sep-2015	14	12-137 caregivers, age range 41-73.7	Relaxation and leisure	Any	Depression, anxiety, well-being, QoL, physical strengthening, blood pressure, weight	Systematic review	Quantitative
Dam, 2016[10]	NA	Any	Pubmed, psycinfo, cinahl, web of science, cochrane library	May-2015	4	NA	Psychosocial	Dementia	NA	Systematic review	Qualitative
Doyle, 2020[11]	NA	RCTs, quasi-experimental, case-control, and cohort studies	Pubmed, web of science, cinahl plus, cochrane library, ot seeker, psycinfo, scopus	Apr-2017	11	862 caregivers, mean age 67, 69.4% female	Relaxation and leisure	Any	Depression, anxiety, physical health	Systematic review	Quantitative
Du Preez, 2018[12]	NA	Qualitative studies and mixed methods	Pubmed, web of science, medical database (proquest), medline, bmj best practice, scopus, psycinfo, otseeker, cinahl	Nov-2016	10	NA	Respite	Dementia	NA	Integrative review	Qualitative

For peer review only - http://bmjopen.bmj.com/site/about/guidelines.xhtml

Author, year	Countries of primary studies	Study design	Databases searched	End date of literature search	# studies included in the umbrella review	# participants, mean age, % female	Intervention type	Disease of care receiver	Health outcomes of caregiver	Review type and synthesis method	Quantitative, qualitative, or mixed-methods
Forster, 2012[13]	US, UK, Australia, Sweden, the Netherlands, Taiwan, Thailand	RCTs	Cochrane library, dare, eed, hta database, medline, embase, cinahl, psycinfo	Jun-2012	21	1290 caregivers	Case management, Psychosocial, Education and skills	Stroke	Depression, HR-QoL	Systematic review	Quantitative
Gabriel, 2020[14]	US, France	RCTs, quasi-experimental	Pubmed, medline, cinahl, psycinfo, web of science, who clinical trials registry, international standard rct registry	2019	12	3390 participants (including caregivers), age range for caregivers 43-61	Psychosocial, Education and skills	Cancer	Psychological/emotional and physical domains of QoL	Systematic review	Quantitative
Goeman, 2016[15]	Hong Kong, Netherlands, US, Italy, Finland, UK, Canada, Australia	Any	Medline, cinahl, psycinfo, google scholar	Dec-2014	36	NA	Case management	Dementia	Depression, HR-QoL, general health	Systematic review	Mixed methods
Gonzalez-Fraile, 2021[16]	US, China, Netherlands, France, Canada, Spain, UK	RCTs	Alois, specialised register of the cochrane dementia and cognitive improvement group	Apr-2020	26	2367 caregivers, median age 63, 72% female	Psychosocial, Education and skills	Dementia	Depression, depressive symptoms, HR-QoL	Systematic review with meta-analysis	Quantitative
Greenwood, 2016[17]	US, Spain	RCTs and quasi-experimental	Medline, embase, cochrane library, psycinfo; cinahl plus, applied social sciences index, abstracts and healthcare management information consortium	Jul-2015	4	447 caregivers, age range 61-72, mostly female	Case management, Psychosocial, Education and skills	Dementia, stroke	Depression, general health	Systematic review	Quantitative
Li, 2016[18]	US, the Netherlands, China, Canada	Any	Cochrane library, pubmed, medline, cinahl, psycinfo, web of science, cnki, wanfang data, vip	Feb-2015	6	NA	Mindfulness	Any	NA	Systematic review	Qualitative
Heckel, 2019[19]	Australia	RCTs and quasi-experimental	Medline, cinahl, psycinfo, embase	May-2018	2	NA	Psychosocial, Education and skills	Cancer	Depression, emotional distress	Systematic review	Quantitative

For peer review only - http://bmjopen.bmj.com/site/about/guidelines.xhtml

Author, year	Countries of primary studies	Study design	Databases searched	End date of literature search	# studies included in the umbrella review	# participants, mean age, % female	Intervention type	Disease of care receiver	Health outcomes of caregiver	Review type and synthesis method	Quantitative, qualitative, or mixed-methods
Hopkinson, 2019[20]	Spain, US, UK, Germany, Canada, Brazil, Italy	RCTs and quasi-experimental	Medline, embase, cinahl, psycinfo, cochrane library	Jan-2017	25	Age range 56.6-72.6	Psychosocial	Dementia	Depression, anxiety	Systematic review with meta-analysis	Quantitative
Hopwood, 2018[21]	US, the Netherlands, Spain, Poland, Denmark, Hong Kong, Canada, France, UK, Germany	Any	Cinahl, cochrane library, embase, medline, psycinfo, web of science	Apr-2018	31	NA	Case management, Psychosocial, Education and skills	Dementia	HR-QoL, depression, anxiety, self-rated health	Systematic review	Mixed methods
Irons, 2020[22]	Australia, UK, US, Israel	Any	Medline, pubmed, ebsco, cinahl, embase, psycinfo, cochrane library, scopus, web of science, google scholar	May-2019	17	NA	Relaxation and leisure	Neurological conditions (e.g., dementia, stroke, Parkinson's disease)	HR-QoL, anxiety, depression	Integrative systematic review, meta-ethnography	Mixed methods
Lee, 2020[23]	US, UK, Germany, Spain, Netherlands, Portugal, China, Russia, Peru, Brazil, Denmark, Greece	RCTs	Medline, cinahl, psycinfo, cochrane library	2017	26	3906 caregivers, mean age 60.5, 73.9% female	Case management, Psychosocial, Education and skills, Relaxation and leisure	Dementia	HR-QoL	Systematic review with meta-analysis	Quantitative

For peer review only

For peer review only - http://bmjopen.bmj.com/site/about/guidelines.xhtml

Author, year	Countries of primary studies	Study design	Databases searched	End date of literature search	# studies included in the umbrella review	# participants, mean age, % female	Intervention type	Disease of care receiver	Health outcomes of caregiver	Review type and synthesis method	Quantitative, qualitative, or mixed-methods
Lins, 2014[24]	US, Germany, Canada	Any	Cochrane dementia and cognitive improvement group's specialized register, cochrane library, medline, medline in process, embase, cinahl, psyndex, psycinfo, web of science, dimdi databases, springer database, science direct, trial registers, web of science, google scholar	Feb-2013	11	NA	Psychosocial, Education and skills	Dementia	Depression, anxiety	Systematic review with meta-analysis	Mixed methods
Liu, 2018[25]	US, China	RCTs and quasi-experimental	Alois, specialised register of the cochrane dementia and cognitive improvement group	Sep-2017	5	201 caregivers, age range 57.5-71.3, >80% female	Mindfulness	Dementia	Depressive symptoms, anxiety	Systematic review with meta-analysis	Quantitative
Lucero, 2019[26]	NA	RCTs	Pubmed, cinahl, web of science, psycinfo	Jul-2017	12	NA	Case management	Dementia	Depression, anxiety	Systematic review	Quantitative
Maayan, 2014[27]	US, Canada	RCTs	Alois, specialised register of the cochrane dementia and cognitive improvement group	Dec-2012	4	NA	Respite	Dementia	Depression	Systematic review	Quantitative
Maffioletti, 2019[28]	US, the Netherlands, Norway, Scotland, Iceland, HongKong, Australia, Italy, Sweden, Germany	Any	Pubmed, psycinfo, scopus, scielo	Aug-2018	21	NA	Respite	Dementia	Depression, psychological well-being, health status	Systematic review	Mixed methods
Miles, 2020[29]	NA	Any design with comparison group	Medline, embase, web of science, cinahl, pubmed, scopus, psycinfo, cochrane library	Sep-2018	2	NA	Psychosocial, Education and skills	Dementia	NA	Systematic review	Qualitative

For peer review only

Author, year	Countries of primary studies	Study design	Databases searched	End date of literature search	# studies included in the umbrella review	# participants, mean age, % female	Intervention type	Disease of care receiver	Health outcomes of caregiver	Review type and synthesis method	Quantitative, qualitative, or mixed-methods
Minshall, 2019[30]	NA	RCTs	Medline, cinahl, psycinfo, socindex, cochrane library, web of science, Scopus databases, grey literature	Sep-2018	21	2079 caregivers, age range 49-76	Psychosocial, Education and skills	Stroke	Depression, HR-QoL	Systematic review with meta-analysis	Quantitative
Parkinson, 2019[31]	NA	Any	Amed, central, cinahl, medline, nursing and allied health database, psycarticles, psycinfo	2017	9	215 caregivers, age range 48.5-65.6	Mindfulness	Any	Anxiety	Systematic review	Mixed methods
Piersol, 2017[32]	NA	Any	Medline, psycinfo, cinahl, otseeker, cochrane database of systematic reviews	Apr-2014	43	NA	Case management, Psychosocial, Education and skills, Relaxation and leisure	Dementia	HR-QoL, depression, anxiety, physical health	Systematic review	Quantitative
Pritchard, 2020[33]	US, Australia, Europe	Qualitative	Medline, cochrane database of systematic reviews, embase, emcare, cinahl, otseeker	May-2018	7	9-495 caregivers, age range 59.8 (SD=13.9) to 68.7 (SD=8.6)	Education and skills	Dementia	NA	Systematic review with meta-analysis, qualitative synthesis (thematic analysis, framework approach)	Qualitative
Pucciarelli, 2020[34]	UK, Spain, Netherlands, US, Taiwan, Australia	RCTs and quasi-experimental	Pubmed, cinahl, psycinfo	May-2019	16	2187 caregivers, mean age 58, 75% female	Case management, Psychosocial, Education and skills	Stroke	Depression, HR-QoL	Systematic review with meta-analysis	Quantitative
Reilly, 2015[35]	Hong Kong, Netherlands, US, India, Canada, UK	RCTs	Cochrane library, medline, embase, psycinfo, cinahl, lilacs, web of science, campbell collaboration/soro database	Mar-2014	11	NA	Case management	Dementia	HR-QoL, depression, general health	Systematic review with meta-analysis	Quantitative

For peer review only

Author, year	Countries of primary studies	Study design	Databases searched	End date of literature search	# studies included in the umbrella review	# participants, mean age, % female	Intervention type	Disease of care receiver	Health outcomes of caregiver	Review type and synthesis method	Quantitative, qualitative, or mixed-methods
Shaw, 2009[36]	UK, US, Canada, Australia, New Zealand, Germany, Japan	Any	Medline, amed, assia, ibss, bni, cochrane library, dare, hta database, nhs economic evaluation database, cinahl, crisp, econlit, embase, hmic, nrr, psycinfo, scopus, social care online, web of science	2008	174	NA	Respite	Frailty	Depression, anxiety	Systematic review with meta-analysis, meta-synthesis of qualitative studies	Mixed methods
Shim, 2020[37]	NA	RCTs	Pubmed, psycinfo, scopus, cinahl, embase	Feb-2020	9	14-120 caregivers	Mindfulness	Dementia	Cognitive tests, depression, mindfulness, anxiety, biomarkers for stress	Systematic review	Quantitative
Sin, 2018[38]	UK, Australia, US, Hong-Kong, Canada, Sweden, Norway, Netherlands, France, Spain, Denmark, Poland, Germany, Italy, Greece	Any	Medline, psycinfo, cinahl, embase, web of science, assia, central, hta database, dare, eed	Dec-2016	81	4537 caregivers	Psychosocial, Education and skills	Any	Depression, anxiety, distress, HR-QoL	Systematic review	Mixed methods
Smith, 2014[39]	US, UK, Canada	Any	Medline, embase, psycinfo, social policy and practice, cinahl plus, allied and complimentary medicine, social sciences citation index, scopus	Jan-2013	2	NA	Psychosocial, Education and skills	Dementia, stroke	NA	Systematic review	Qualitative

For peer review only - http://bmjopen.bmj.com/site/about/guidelines.xhtml

Author, year	Countries of primary studies	Study design	Databases searched	End date of literature search	# studies included in the umbrella review	# participants, mean age, % female	Intervention type	Disease of care receiver	Health outcomes of caregiver	Review type and synthesis method	Quantitative, qualitative, or mixed-methods
Smith, 2019[40]	US, UK, Australia, Sweden, Canada, Ireland, Germany, India, Spain, Taiwan, Thailand	RCTs and quasi-experimental	Embase, medline, cinahl, pubmed, world health organization international clinical trial registry platform registry, clinicaltrials.gov registry	Mar-2019	23	NA	Education and skills	Stroke, frailty	HR-QoL, depression, anxiety	Systematic review with meta-analysis	Quantitative
Teahan, 2020[41]	US, China, Spain, the Netherlands, Taiwan, UK, Peru, Russia, India	RCTs	Cochrane, pubmed, cinahl, psycinfo, embase, assia	Mar-2016	24	NA	Psychosocial, Education and skills	Dementia	Depression, HR-QoL, general health	Systematic review with meta-analysis	Quantitative
Treanor, 2019[42]	US, UK, Denmark, Canada, Netherlands, Australia	RCTs and quasi-experimental	Cochrane central, medline, embase, psycinfo, proquest, open single, web of science	Dec-2018	19	1967 caregivers	Psychosocial, Education and skills	Cancer	HR-QoL, depression, anxiety, emotional distress, physical health status	Systematic review with meta-analysis	Quantitative
Vandepitte, 2016[43]	Canada, US, Germany, Italy	RCTs and quasi-experimental	Pubmed, web of science	Mar-2015	5	NA	Respite	Dementia	Depression, anxiety	Systematic review	Quantitative
Waldron, 2013[44]	N.A.	RCTs	Medline, psycinfo, embase, cinahl, pubmed, cochrane library	Nov-2010	6	1115 caregivers, mean age 56.5 (SD=3.3), 81.9% female	Psychosocial	Cancer	HR-QoL	Systematic review	Quantitative
Wang, 2020[45]	US, the Netherlands, France	RCTs and quasi-experimental	Cinahl, scopus, embase, medline, psycinfo, web of science, world health organization international clinical trials registry platform	Jul-2019	8	NA	Psychosocial	Dementia	Depression, anxiety	Systematic review with meta-analysis	Quantitative
Wiegelmann, 2021[46]	US, UK, Germany, China	RCTs	Pubmed, psycinfo, scopus, cinahl	Aug-2018	37	NA	Psychosocial, Education and skills	Dementia	HR-QoL, depression	Systematic review	Quantitative

For peer review only - http://bmjopen.bmj.com/site/about/guidelines.xhtml

Author, year	Countries of primary studies	Study design	Databases searched	End date of literature search	# studies included in the umbrella review	# participants, mean age, % female	Intervention type	Disease of care receiver	Health outcomes of caregiver	Review type and synthesis method	Quantitative, qualitative, or mixed-methods
Zabihi, 2020[47]	Hong Kong, US, Spain, Australia	RCTs	Medline, embase, psycinfo, cinahl, central and online trial registers for randomised controlled clinical trials	Dec-2018	14	NA	Psychosocial	Dementia	Depression (symptoms and diagnosis)	Systematic review with meta-analysis	Quantitative

Abbreviations: RCT, Randomized Controlled Trial; QoL, quality of life; HR-QoL, Health-Related Quality of Life.

For peer review only

Full references of all included reviews (n=47)

1. Akarsu NE, Prince MJ, Lawrence VC, Das-Munshi J. Depression in carers of people with dementia from a minority ethnic background: Systematic review and meta-analysis of randomised controlled trials of psychosocial interventions. *Int J Geriatr Psychiatry*. 2019;34(6):790-806. doi:10.1002/gps.5070
2. Al Daken LI, Ahmad MM. The implementation of mindfulness-based interventions and educational interventions to support family caregivers of patients with cancer: A systematic review. *Perspect Psychiatr Care*. 2018;54(3 PG-441-452):441-452. doi:https://dx.doi.org/10.1111/ppc.12286
3. Bennett S, Laver K, Voigt-Radloff S, et al. Occupational therapy for people with dementia and their family carers provided at home: a systematic review and meta-analysis. *BMJ Open*. 2019;9(11 PG-e026308):e026308. doi:https://dx.doi.org/10.1136/bmjopen-2018-026308
4. Boots LM, de Vugt ME, van Knippenberg RJ, Kempen GI, Verhey FR. A systematic review of Internet-based supportive interventions for caregivers of patients with dementia. *Int J Geriatr Psychiatry*. 2014;29(4 PG-331-344):331-344. doi:https://dx.doi.org/10.1002/gps.4016
5. Bourne P, Camic PM, Crutch SJ. Psychosocial outcomes of dyadic arts interventions for people with a dementia and their informal caregivers: A systematic review. *Heal Soc Care Community*. 2021;29(6):1632-1649. doi:10.1111/hsc.13267
6. Bunn F, Goodman C, Pinkney E, Drennan VM. Specialist nursing and community support for the carers of people with dementia living at home: An evidence synthesis. *Heal Soc Care Community*. 2016;24(1):48-67. doi:10.1111/hsc.12189
7. Cheng ST, Li KK, Losada A, et al. The effectiveness of nonpharmacological interventions for informal dementia caregivers: An updated systematic review and meta-analysis. *Psychol Aging*. 2020;35(1 PG-55-77):55-77. doi:https://dx.doi.org/10.1037/pag0000401
8. Corry M, Neenan K, Brabyn S, Sheaf G, Smith V. Telephone interventions, delivered by healthcare professionals, for providing education and psychosocial support for informal caregivers of adults with diagnosed illnesses. *Cochrane Database Syst Rev*. 2019;5(PG-CD012533):CD012533. doi:https://dx.doi.org/10.1002/14651858.CD012533.pub2
9. Cuthbert CA, King-Shier K, Ruether D, Tapp DM, Culos-Reed SN. What is the state of the science on physical activity interventions for family caregivers? A systematic review and RE-AIM evaluation. *J Phys Act Heal*. 2017;14(7):578-595. doi:10.1123/jpah.2016-0280
10. Dam AE, de Vugt ME, Klinkenberg IP, Verhey FR, van Boxtel MP. A systematic review of social support interventions for caregivers of people with dementia: Are they doing what they promise? *Maturitas*. 2016;85(PG-117-130):117-130. doi:https://dx.doi.org/10.1016/j.maturitas.2015.12.008
11. Doyle KL, Toepfer M, Bradfield AF, et al. Systematic review of exercise for caregiver-care recipient dyads: What is best for spousal caregivers - Exercising together or not at all? *Gerontologist*. 2021;61(6):E283-E301. doi:10.1093/geront/gnaa043
12. Du Preez J, Millsteed J, Marquis R, Richmond J. The Role of Adult Day Services in Supporting the Occupational Participation of People with Dementia and Their Carers: An Integrative Review. *Healthcare*. 2018;6(2 PG-08):8. doi:https://dx.doi.org/10.3390/healthcare6020043
13. Forster A, Brown L, Smith J, et al. Information provision for stroke patients and their caregivers. *Cochrane Database Syst Rev*. Published online November 14, 2012. doi:10.1002/14651858.cd001919.pub3
14. Gabriel I, Creedy D, Coyne E. A systematic review of psychosocial interventions to improve quality of life of people with cancer and their family caregivers. *Nurs Open*. 2020;7(5):1299-1312. doi:10.1002/nop2.543
15. Goeman D, Renehan E, Koch S. What is the effectiveness of the support worker role for people with dementia and their carers? A systematic review. *BMC Health Serv Res*. 2016;16(1). doi:10.1186/s12913-016-1531-2
16. González-Fraile E, Ballesteros J, Rueda JR, Santos-Zorrozúa B, Solà I, McCleery J. Remotely delivered information, training and support for informal caregivers of people with dementia. *Cochrane Database Syst Rev*. 2021;2021(1). doi:10.1002/14651858.CD006440.pub3
17. Greenwood N, Pelone F, Hassenkamp AM. General practice based psychosocial interventions for supporting carers of people with dementia or stroke: a systematic review. *BMC Fam Pract*. 2016;17(PG-3):3. doi:https://dx.doi.org/10.1186/s12875-015-0399-2
18. Li G, Yuan H, Zhang W. The Effects of Mindfulness-Based Stress Reduction for Family Caregivers: Systematic Review. *Arch Psychiatr Nurs*. 2016;30(2):292-299. doi:10.1016/j.apnu.2015.08.014
19. Heckel L, Heynsbergh NL, Livingston PM. Are cancer helplines effective in supporting caregivers? A systematic review. *Support Care Cancer*. 2019;27(9):3219-3231. doi:10.1007/s00520-019-04807-z
20. Hopkinson MD, Reavell J, Lane DA, Mallikarjun P. Cognitive Behavioral Therapy for Depression, Anxiety, and Stress in Caregivers of Dementia Patients: A Systematic Review and Meta-Analysis. *Gerontologist*. 2019;59(4 PG-343-362):e343-e362. doi:https://dx.doi.org/10.1093/geront/gnx217
21. Hopwood J, Walker N, McDonagh L, et al. Internet-Based Interventions Aimed at Supporting Family Caregivers of People With Dementia: Systematic Review. *J Med Internet Res*. 2018;20(6 PG-e216):e216. doi:https://dx.doi.org/10.2196/jmir.9548
22. Yoon Irons J, Garip G, Cross AJ, Sheffield D, Bird J. An integrative systematic review of creative arts interventions for older informal caregivers of people with neurological conditions. *PLoS One*. 2020;15(12 December). doi:10.1371/journal.pone.0243461
23. Lee M, Ryoo JH, Crowder J, Byon HD, Wiiliams IC. A systematic review and meta-analysis on effective interventions for health-related quality of life among caregivers of people with dementia. *J Adv Nurs*. 2020;76(2 PG-475-489):475-489. doi:https://dx.doi.org/10.1111/jan.14262
24. Lins S, Hayder-Beichel D, Rucker G, et al. Efficacy and experiences of telephone counselling for informal carers of people with dementia. *Cochrane Database Syst Rev*. 2014;(9 PG-CD009126):CD009126.

For peer review only

doi:https://dx.doi.org/10.1002/14651858.CD009126.pub2
25. Liu Z, Chen Q lin, Sun Y ying. Mindfulness training for psychological stress in family caregivers of persons with dementia: A systematic review and meta-analysis of randomized controlled trials. *Clin Interv Aging*. 2017;12:1521-1529. doi:10.2147/CIA.S146213
26. Lucero RJ, Fehlberg EA, Patel AGM, et al. The effects of information and communication technologies on informal caregivers of persons living with dementia: A systematic review. *Alzheimer's Dement Transl Res Clin Interv*. 2019;5:1-12. doi:10.1016/j.trci.2018.11.003
27. Maayan N, Soares-Weiser K, Lee H. Respite care for people with dementia and their carers. *Cochrane Database Syst Rev*. 2014;2014(1). doi:10.1002/14651858.CD004396.pub3
28. Maffioletti VLR, Baptista MAT, Santos RL, Rodrigues VM, Dourado MCN. Effectiveness of day care in supporting family caregivers of people with dementia: A systematic review. *Dement Neuropsychol*. 2019;13(3 PG-268-283):268-283. doi:https://dx.doi.org/10.1590/1980-57642018dn13-030003
29. Miles L, McCausland BMS, Patel HP, Amin J, Osman-Hicks VC. A systematic review of the provision and efficacy of patient and carer information and support (PCIS) interventions for patients with dementia and their informal carers. *Aging Clin Exp Res*. 2020;32(12):2439-2448. doi:10.1007/s40520-019-01428-8
30. Minshall C, Pascoe MC, Thompson DR, et al. Psychosocial interventions for stroke survivors, carers and survivor-carer dyads: a systematic review and meta-analysis. *Top Stroke Rehabil*. 2019;26(7 PG-554-564):554-564. doi:https://dx.doi.org/10.1080/10749357.2019.1625173
31. Parkinson B, Lawrence M, McElhinney E, Booth J. Mindfulness for people with long-term conditions and their family caregivers: A systematic review. *Complement Ther Clin Pract*. 2019;34:76-86. doi:10.1016/j.ctcp.2018.10.019
32. Piersol CV, Canton K, Connor SE, Giller I, Lipman S, Sager S. Effectiveness of Interventions for Caregivers of People With Alzheimer's Disease and Related Major Neurocognitive Disorders: A Systematic Review. *Am J Occup Ther*. 2017;71(5):p1-7105180020. doi:10.5014/ajot.2017.027581
33. Pritchard E, Cussen A, Delafosse V, Swift M, Jolliffe L, Yeates H. Interventions supporting caregiver readiness when caring for patients with dementia following discharge home: A mixed-methods systematic review. *Australas J Ageing*. 2020;39(3 PG-239-250):e239-e250. doi:https://dx.doi.org/10.1111/ajag.12765
34. Pucciarelli G, Lommi M, Magwood GS, et al. Effectiveness of dyadic interventions to improve stroke patient-caregiver dyads' outcomes after discharge: A systematic review and meta-analysis study. *Eur J Cardiovasc Nurs*. 2021;20(1):14-33. doi:10.1177/1474515120926069
35. Reilly S, Miranda-Castillo C, Malouf R, et al. Case management approaches to home support for people with dementia. *Cochrane Database Syst Rev*. 2015;2017(6). doi:10.1002/14651858.CD008345.pub2
36. Shaw C, McNamara R, Abrams K, et al. Systematic review of respite care in the frail elderly. *Health Technol Assess*. 2009;13(20 PG-1-224, iii):1-224, iii. doi:https://dx.doi.org/10.3310/hta13200
37. Shim M, Tilley JL, Im S, Price K, Gonzalez A. A Systematic Review of Mindfulness-Based Interventions for Patients with Mild Cognitive Impairment or Dementia and Caregivers. *J Geriatr Psychiatry Neurol*. 2021;34(6):528-554. doi:10.1177/0891988720957104
38. Sin J, Henderson C, Spain D, Cornelius V, Chen T, Gillard S. eHealth interventions for family carers of people with long term illness: A promising approach? *Clin Psychol Rev*. 2018;60:109-125. doi:10.1016/j.cpr.2018.01.008
39. Smith R, Greenwood N. The impact of volunteer mentoring schemes on carers of people with dementia and volunteer mentors: a systematic review. *Am J Alzheimer's Dis Other Dementias*. 2014;29(1 PG-8-17):8-17. doi:https://dx.doi.org/10.1177/1533317513505135
40. Smith TO, Pearson M, Pfeiffer K, Crotty M, Lamb SE. Caregiver Interventions for Adults Discharged from the Hospital: Systematic Review and Meta-Analysis. *J Am Geriatr Soc*. 2019;67(9):1960-1969. doi:10.1111/jgs.16048
41. Teahan Á, Lafferty A, McAuliffe E, et al. Psychosocial Interventions for Family Carers of People With Dementia: A Systematic Review and Meta-Analysis. *J Aging Health*. 2020;32(9):1198-1213. doi:10.1177/0898264319899793
42. Treanor CJ, Santin O, Prue G, et al. Psychosocial interventions for informal caregivers of people living with cancer. *Cochrane Database Syst Rev*. 2019;2019(6). doi:10.1002/14651858.CD009912.pub2
43. Vandepitte S, Van Den Noortgate N, Putman K, Verhaeghe S, Verdonck C, Annemans L. Effectiveness of respite care in supporting informal caregivers of persons with dementia: a systematic review. *Int J Geriatr Psychiatry*. 2016;31(12):1277-1288. doi:10.1002/gps.4504
44. Waldron EA, Janke EA, Bechtel CF, Ramirez M, Cohen A. A systematic review of psychosocial interventions to improve cancer caregiver quality of life. *Psychooncology*. 2013;22(6 PG-1200-1207):1200-1207. doi:https://dx.doi.org/10.1002/pon.3118
45. Wang S, Bressington DT, Leung AYM, Davidson PM, Cheung DSK. The effects of bibliotherapy on the mental well-being of informal caregivers of people with neurocognitive disorder: A systematic review and meta-analysis. *Int J Nurs Stud*. 2020;109. doi:10.1016/j.ijnurstu.2020.103643
46. Wiegelmann H, Speller S, Verhaert LM, Schirra-Weirich L, Wolf-Ostermann K. Psychosocial interventions to support the mental health of informal caregivers of persons living with dementia - a systematic literature review. *BMC Geriatr*. 2021;21(1 PG-94):94. doi:https://dx.doi.org/10.1186/s12877-021-02020-4
47. Zabihi S, Lemmel FK, Orgeta V. Behavioural Activation for Depression in Informal Caregivers: A Systematic Review and Meta-Analysis of Randomised Controlled Clinical Trials. *J Affect Disord*. 2020;274(PG-1173-1183):1173-1183. doi:10.1016/j.jad.2020.03.124

For peer review only - http://bmjopen.bmj.com/site/about/guidelines.xhtml

Supplementary Table 7. Summary of findings for interventions involving case management

First author, year	Summary of findings on effectiveness
Goeman, 2016	Inconclusive findings with similar number of studies reporting either positive effects or no effects.
Greenwood, 2016	No significant effect.
Hopwood, 2018	No significant effect. However, some multicomponent interventions in this review show promise for reducing caregivers' depressive symptoms and anxiety.
Lee, 2020	No significant effect: Hedges G was 0.135 (95% CI=-0.076, 0.346, *p*=0.210). However, multicomponent interventions were effective: Hedges G 0.255 (95% CI=0.054, 0.457, *p*=0.013).
Lucero, 2019	Telephone-based intervention showed significant decrease in depression.
Piersol, 2017	No significant effect.
Reilly, 2015	Significant reduction in depression and short-term health (MD=-0.08, 95% CI=-0.16, -0.01, n=2888, *p*=0.03), but no effect on longer follow-ups.
Pucciareli, 2020	No significant effect: SMD=-0.19, 95% CI=-0.40, 0.00, *p*=0.05.

Abbreviations: CI, confidence interval; SMD, standardized mean difference; MD, mean difference.

For peer review only

Supplementary Table 8. Summary of findings for psychosocial, psychoeducational, and skills-building interventions

First author, year	Summary of findings on effectiveness
**Reviews that included both psychosocial and educational interventions (n=19)**	
Akarsu, 2019	In random effects MA, interventions (psychological, multicomponent, and educational) to address depression in ethnic minority caregivers relative to the control condition were associated with an overall mean reduction in depression scores (SMD=-0.17, 95% CI=-0.29, -0.05, *p*=0.005). Heterogeneity was negligible (I2=0.0%, *p*=0.547).
Gonzalez-Fraile, 2021	Remotely delivered interventions involving training, support, or both (with or without information) do not affect caregiver depressive symptoms (SMD=-0.05, 95% CI=-0.22, 0.12, I2=9%) or caregiver HR-QoL (SMD=0.10, 95% CI=-0.13, 0.32, I2=0%). However, these interventions may result in a slight improvement in caregiver depressive symptoms (SMD=-0.25, 95% CI=-0.43, -0.06, I2 = 53%) compared to a control condition of information alone. Interventions may result in little or no difference in caregiver HR-QoL when compared with information alone (SMD=-0.03, 95% CI=-0.28, 0.21, I2=0%).
Greenwood, 2016	One study reported no significant effects in the proportion of caregivers with scores of CES-D ≥16 (i.e., at risk of depression) between the intervention and control groups. Another study reported significant positive changes over time (*p*=0.009) for improving depression scores.
Hopwood, 2018	Overall, studies assessing psychological support suggested a positive effect on a variety of factors, including improving caregiver distress, depression, anxiety, and strain. Some multiple-component interventions showed promise in reducing stress, anxiety, and depressive symptoms for family caregivers and in increasing self-efficacy.
Lee, 2020	CBT was effective, Hedges G=0.767 (95% CI=0.142, 1.391, *p*=0.016); Psychoeducation was not effective, Hedges G=0.163 (95% CI=-0.001, 0.328, *p*=0.052); Social support and cognitive rehabilitation were not effective, G=0.231 (95% CI=-0.104, 0.567, *p*=0.176 and G=0.010 (95% CI=-0.208, 0.229, *p*=0.926), respectively. However, multicomponent interventions were effective: Hedges G=0.255 (95% CI=0.054, 0.457, *p*=0.013).
Lins, 2014	Without educational material and workbook: significant pooled difference in depressive symptoms between the telephone counselling group and the control group (SMD=0.32, 95% CI=0.01, 0.63, *p*=0.04). With educational material and workbook: reduction of depressive symptoms in the intervention group and an increase in the control group after months: -4.1% points in the intervention group (from 7.20% to 3.10%) and 0.56% points in the control group (from 7.44% to 8%). The difference between the groups was statistically significant (*p*=0.01).
Piersol, 2017	Communication skills training: increased QoL and well-being; Coaching: reduced stress and depression; Psychoeducation: improved QoL, well-being, reduced depression; Group interventions: strong evidence indicates that in person caregiver support groups led by professionals improved caregiver well-being and reduced depression; CBT: compelling evidence that interventions focused on cognitive reframing and skills training reduced caregiver depression and anxiety.
Teahan, 2020	Interventions were shown to have a significant effect (*p*=0.002) on caregivers' depression levels pre- and postintervention (SMD=-0.36, 95% CI=-0.60, -0.13, 11 trials, N=1856 participants). The interventions were shown to have a significantly small to moderate effect size (*p*<0.01) on the general health scores of caregivers pre- and postintervention (SMD=0.34, 95% CI=0.18, 0.51, 7 trials, N=1503 participants). Although the overall effect on QoL was not statistically significant (*p*=0.12), there was a trend in favor of intervention (SMD=0.63, 95% CI=-0.16, 1.43, 3 interventions, N=201).
Wiegelmann, 2021	4 out of 11 psychoeducational interventions described positive results, while 7 reported no significant effect. 1 out of 4 counselling interventions reported improvement in depression. 4 out of six CBT interventions reported positive effects. 3 out of 8 psychoeducational interventions reported improvement in QoL. 1 out of 6 counselling interventions improved QoL. 1 CBT intervention measuring QoL failed to report significant effects. Neither of the 2 befriending interventions reported positive changes in QoL.
Zabihi, 2020	MA of effects of behavioral activation showed that results significantly favored intervention in reducing caregiver depressive symptoms at post-treatment (8 studies, 815 participants, SMD=-0.68, 95% CI=-1.14,

For peer review only - http://bmjopen.bmj.com/site/about/guidelines.xhtml

	-0.22). There was high heterogeneity between studies (I2=89%). Long-term effects were also favorable (2 studies, 235 participants, SMD=-0.99, 95% CI=-1.26, -0.71, I2= 92%. Behavioral activation was also associated with decreased risk of depression diagnosis (3 studies, 854 participants, OR=0.35, 95% CI=0.19, 0.67, efficacy at 3-12 months) and was superior to psychoeducation in one study.
Gabriel, 2020	All 12 studies assessed psychological/emotional well-being; 4 studies report statistically significant improvement for dyads, 2 studies report significant improvement for caregivers. Remaining 6 studies report no significant change. As for physical well-being, all 12 studies assessed it; 5report significant improvement for dyads, while 1 reports it for care receivers only, 6 report no change.
Heckel, 2019	No significant effect on depression (1 study), another study showed positive effects for reducing emotional distress.
Treanor, 2019	Considering the pooled and unpooled findings, psychosocial interventions may improve slightly QoL immediately post-intervention, but this benefit may not be maintained in the longer term (6-2 months later). Pooled and unpooled findings indicated that psychosocial interventions probably have little to no effect on depression, anxiety or emotional distress for caregivers. Evidence for physical outcomes is insufficient.
Waldron, 2013	Nil to small effects on QoL outcomes were found in the 4 studies where QoL effect sizes could be calculated, and 2 of these reported significant changes in QoL.
Corry, 2019	There is little or no difference between telephone support interventions and usual care for QoL at the end of intervention (SMD=-0.02, 95% CI=-0.24, 0.19, 4 studies, 364 caregivers) and at short-term follow-up (3 months) (SMD=0.00, 95% CI=-4.43, 4.43, 1 study, 128 caregivers). The effects of telephone interventions on depression at the end of intervention were uncertain (SMD=-0.37, 95% CI=-0.70, -0.05, 9 studies, 792 caregivers). Telephone interventions may have little or no effect at medium-term follow-up (3-6 months) (SMD=-0.05, 95% CI=-0.56, 0.45, 3 studies, 227 caregivers). Telephone interventions compared with usual care may slightly decrease anxiety levels at the end of intervention and short-term follow-up (2 studies, no MA). Telephone support interventions, when compared to usual care may have little or no effect on caregiver physical health at the end of intervention (SMD=-0.09, 95% CI=-0.35, 0.17, 2 studies, 248 caregivers).
Sin, 2018	Of those studies that assessed effectiveness of interventions, 44% reported equivocal findings (no effect), 12% saw negative effects (control group more favorable than intervention) and only 32% reported positive effects. While caregivers reported positive experiences in using the interventions (as per qualitative findings), no significant changes in their objective outcomes were identified.
Forster, 2012	Out of 3 RCTs measuring depression, 2 showed no significant effect, while 1 reported less depression in the intervention group. Out of 4 RCTs measuring QoL, 3 report no effects, while 1 reported higher QoL in the intervention group.
Minshall, 2019	Based on MA of 6 RCTs measuring depression, the effect is insignificant (pooled effect size=-0.20, 95% CI -0.40, 0.00, n=279 in intervention group, n=282 in control group). One trial reported improvements in caregiver QoL.
Pucciarelli, 2020	Caregivers who received an educational intervention were found to have lower depression levels than those in the control group, although no significant differences were observed between these two groups (SMD=-0.19, 95% CI=-0.40, 0.00, *p*=0.05).

Reviews that included only psychosocial (n=2) or only educational (n=2) interventions

Bennett, 2019 (occupational therapy)	Data from six studies that measured the emotional distress family caregivers felt were combined, and demonstrated a small, statistically significant result in favor of those receiving occupational therapy (SMD=-0.23, 95% CI=-0.42, -0.05, I2=41%). Only 3 studies measured overall QoL of the caregivers of people with dementia. Pooled data from 2 of these studies produced a large, significant between-group difference after the intervention (SMD=0.99, 95% CI=0.66, 1.33, I2=2%).
Smith, 2019 (training)	There was no benefit on caregiver HR-QoL compared with the control group at 3-6 months (SMD=0.20; 95% CI=-1.12, 1.52, N=638, I2=98%) or 12 months (SMD=0.46, 95% CI=-0.34, 1.27, N=415, I2=94%). There was no benefit from the caregiver intervention on caregiver depression compared with the control group at 3 to 6 months (SMD=0.03,95% CI=-0.08, 0.14, N=1239, I2=0%) or 12 months (SMD=-0.71, 95% CI=-2.23, 0.81, N=613, I2=99%). For anxiety, 1

For peer review only - http://bmjopen.bmj.com/site/about/guidelines.xhtml

	study reported significant effect, while another reported no improvement.
Hopkinson, 2019 (psychosocial)	Caregivers receiving CBT demonstrated statistically significant reductions in depression (n=12; 995 participants; SMD=-0.34; 95% CI=-0.47, -0.21, *p*<0.001 relative to comparator groups immediately after the intervention endpoint and in 3 months (n=3; SMD=-0.99, 95% CI=-1.35, -0.64, *p*<0.001). There was no significant difference in caregiver anxiety (n=10, 829 participants, SMD=0.10; 95% CI=-0.18, 0.39, *p*=0.47).
Wang, 2020 (psychosocial)	For depression, the overall effect for the 2 types of bibliotherapy was significant at Z=1.99 (random effect model, SMD=-0.74, 95% CI=-1.47, -0.01, *p*=0.05). However, when considered separately, only the video based bibliotherapy significantly reduced depression at Z=2.78 (random effect model, SMD=-2.11, 95% CI=-3.6, -0.62, *p*=0.005). For anxiety, the heterogeneity of the 3 studies included in MA was low (I2=22%), the overall effect of bibliotherapy on anxiety was significant at Z=2.30, *p*=0.02 (random effect model, SMD=-0.22, 95% CI=-0.41, -0.33).

Abbreviations: RCT, randomized controlled trial; CI, confidence interval; SMD, standardized mean difference; CBT, cognitive behavioral therapy; QoL, quality of life; HR-QoL, health-related quality of life; MA, meta-analysis.

For peer review only

Supplementary Table 9. Summary of findings for interventions involving respite care

First author, year	Summary of findings on effectiveness
Maayan, 2019	Analysis of the available data showed no significant effects on caregiver outcomes when respite care was compared with no respite care in 3 studies.
Maffioletti, 2019	5 studies reported positive effects on depression, 3 on psychological/psycho-somatic complaints, and 4 studies also report improved health status and greater psychological well-being. 2 studies report no changes in caregiver outcomes.
Vandepitte, 2016	Reported benefits of day care provision for caregivers were small and mixed (33% were effective). The only included in-home respite care program indicated some beneficial effects for caregivers.
Shaw, 2009	MA showed insignificant positive effect in favor of respite care at short term, but no effect in long term. Evidence from narratively reviewed studies indicated that respite does not have a significant impact on psychological well-being or depression when compared with normal care. Few studies (with lower quality of design, not in MA) reported decrease in depression, but at short follow-up times. MA of randomized/quasi-experimental studies measuring caregiver anxiety as an outcome did not demonstrate any significant effects.

Abbreviations: MA, meta-analysis.

For peer review only

Supplementary Table 10. Summary of findings for interventions involving relaxation, physical activity, or leisure

First author, year	Summary of findings on effectiveness
Cheng, 2020	Miscellaneous interventions were shown to reduce stress and depressive symptoms. For this group of interventions, Hedges G=-0.49, n=12, N=758. Interestingly, in this review, multicomponent and miscellaneous interventions had the largest effects.
Lee, 2020	MA of the 2 studies showed that the Hedges G was 0.576 (95% CI=0.035, 1.118, *p*=0.037), indicating that there was a statistically significant difference between the control group and the intervention group on improving caregiver HR-QoL.
Piersol, 2017	Exercise programs showed improved caregiver physical health and decreased stress. Adapted leisure programs showed improved well-being.
Irons, 2020	For outcomes of interest, pre-post effect sizes revealed some benefits of the intervention: large effect sizes were detected for singing on well-being (Cohen's d=1.04), and positive-negative mood (Cohen's d=1.29); and for music making on relaxation (Cohen's d=1.91), comfort (Cohen's d=1.74), and happiness (Cohen's d=1.19). Some studies indicated decreases in some aspects of well-being which were not consistent with other aspects reported. For example, one study reported increases in stress, but decreases in anxiety and depression.
Cuthbert, 2017	Across all studies, statistically significant results for the following outcomes in the physical activity groups were reported: (1) decrease in depression rates; (2) decrease in anger scores; (3) improvement in sleep quality; (4) lower systolic and diastolic blood pressure reactivity.
Doyle, 2020	Results were mixed across and within DyEx and DySplit studies. Of the DyEx studies, 6 examined caregiver psychosocial well-being. Beneficial outcomes in 4 studies indicated significant improvements in mental health and QoL of caregivers. In 3 DySplit interventions, caregivers experienced significant improvements to mental health when they were offered non-exercise interventions of either respite or a dyadic support group, while their care receivers exercised. 3 DyEx studies measured caregiver physical health. A resistance training intervention noted significant increases in muscle mass, strength, and physical function, but no significant difference in gait speed. Self-reported physical outcomes were equivocal.

Abbreviations: CI, confidence interval; QoL, quality of life; HR-QoL, health-related quality of life; DyEx, dyadic interventions; DySplit, non-dyadic interventions.

For peer review only

Supplementary Table 11. Summary of findings for interventions involving mindfulness-based activities

First author, year	Summary of findings on effectiveness
Cheng, 2020	Mindfulness-based interventions reduced depressive symptoms. For subjective well-being G=0.31, k=6, N=212. For depression G=-0.58, k=7, N=258.
Liu, 2018	MBSR vs active controls immediately after intervention: MBSR decreased depressive symptoms of caregivers compared with the active-control interventions: SMD=-0.63 (95% CI=-0.98, -0.28, *p*<0.001). MBSR could reduce caregivers' anxiety compared with the active-control group immediately after the intervention period: MD=-7.50 (95% CI=-13.11, -1.89, *p*=0.009). MBSR vs inactive controls immediately after intervention: no clear effect on depressive symptoms of caregivers in the MBSR group compared with the inactive controls immediately after the intervention period. MBSR may reduce caregivers' levels of anxiety compared with the inactive control immediately after the intervention period MD=-7.27 (95% CI=-14.92, 0.38, *p*=0.06. MBSR vs controls at follow-up: compared with the active-control intervention, the results for depressive symptoms suggested that there may be little or no effect of MBSR MD=-0.16 (95% CI=-0.71, 0.39, *p*=0.57). Compared with the inactive control, the results for caregivers' depressive symptoms slightly favored MBSR MD=-3.00 (95% CI=-8.52, 2.52, *p*=0.29).
Shim, 2020	3 studies show that cognitive functioning was significantly improved in MBI conditions compared to both active and passive control conditions. 4 studies reported statistically significant improvements in self-reported measures of stress or distress in the MBI condition at post-treatment or follow-up with treatment effects in the small to medium range (G=-0.078 to -0.602). 3 out of 7 studies examining depression/anxiety reported relative improvements. When compared with passive control interventions, caregivers in MBIs reported significant improvements in QoL. Long term effects were not that pronounced. No treatment differences in outcomes were found at 3- and 9-month follow-up assessments.
Parkinson, 2019	Some improvements were noted for patients' and their caregivers' anxiety scores following intervention, but these changes were not significant at post treatment or at follow-up. 1 study provided a narrative account of results and reported no change in anxiety scores following intervention for participants or family caregivers. Significant (*p*=0.01) improvements in the tension/anxiety score were reported in 1 study. Caregivers experienced a mean reduction depression score in 5 studies, but the improvements in depression were often small and only significant in 3 studies.

Abbreviations: MBSR, Mindfulness-Based Stress Reduction; MBI, Mindfulness-Based Interventions; MCI, mild cognitive impairment; SMD, standardized mean difference; MD, mean difference; CI, confidence interval.

For peer review only

For peer review only - http://bmjopen.bmj.com/site/about/guidelines.xhtml

Supplementary Table 12. Coding table for qualitative data

Theme (and definition)	Category (and definition)	Sub-category (and definition)	Fourth level construct codes	Third level construct verbatim	References
**Intervention outcomes:** This category describes potential beneficial and/or harmful effects to the caregiver from participating in the interventions	**Health outcomes:** Health benefits experienced and expressed by caregivers because of participating in support interventions.	**NA**	Education/skills: hospital skills-building brings less anxiety, depression, burden, better quality of life	When interventions are provided to the caregiver in the context of the hospital setting to enhance these skills, there is a likelihood of reducing anxiety, depression, caregiver burden and improving QoL.	Pritchard
			Mindfulness: benefits include relaxation, less depression, less worry, and anxiety	Triangulated qualitative and quantitative data suggests MBIs can improve relaxation, ease anxiety, and reduce depression for people with LTCs and their family caregivers. […] The reported psychological benefits included increased relaxation, reduced worry, and reductions in anxiety.	Parkinson
**Intervention outcomes:** This category describes potential beneficial and/or harmful effects to the caregiver from participating in the interventions	**Social outcomes:** intervention outcomes related to impact on caregiver's social life, day-to-day routine and relationship with care receiver and other people.	**Practical social outcomes:** changes in caregivers' day-to-day routine, resulting from acquiring new skills or knowledge.	Case management: nurse and carer working together decreases burden	A shared approach to care was found to be vital in decreasing burden among family members.	Goeman
			Case management: prevents crises	Case management offered potential benefit to people with dementia, their carers and community-based professionals through continuity of care by a named trusted individual that could act proactively to prevent a crisis.	Goeman
			Case management: admiral nurses have good relationship with carers and care receivers	Admiral nurse: Positive outcomes in the carer/key worker relationship to be linked to the quality of the relationship and involve the carer and professional care worker actively including and working with the person with dementia.	Goeman
			Psychoeducation: brings satisfaction, coping skills, competence, confidence, less burden, less loneliness	Increased satisfaction with support, coping skills, caregiving competence, confidence, and decreased burden and loneliness.	Smith
			Psychoeducation: brings increased knowledge, coping, confidence, less isolation	Increased knowledge of the disease and caring was described in 3 papers, in addition to increased coping with caregiving (2 papers) confidence in caregiver skills (2 papers) and reduced feelings of isolation (1 paper).	Boots
			Education/skills: Strong relationship between health staff and carers needed for continued informal care	When health professionals build strong relationships with the caregiver, they are more likely to be able to support the readiness of the caregiver to continue their role.	Pritchard
			Education/skills: when needs are met, coping is better	Caregivers identified when personal characteristics of both patient and caregiver are considered, caregivers are included as an integral part of the team (as they requested), and they receive timely and effective information, the level of stress is likely to reduce, and their ability to cope to be ready for discharge is likely to increase.	Pritchard

For peer review only - http://bmjopen.bmj.com/site/about/guidelines.xhtml

			Education/skills: carers' coping mechanisms promoted	The interventions addressed the need for clear information about the disease, identification of psychological response, development of coping mechanisms and assessment of the home environment.	Pritchard
			Counselling: counsellors help with seemingly helpless situations	Being able to describe and discuss problematic situations helps carers in their daily lives when they do not know how what to do next, because, from their perspective, they have tried everything but have not been able to resolve the situation.	Lins
			Counselling: counsellors knowledgeable about services is helpful	Carers find the information provided helpful and are grateful for it. The information and advice help them to do certain things better, which leads to them feeling happier with themselves.	Lins
			Respite: free time used for leisure or chores	The respite time gained through this type of care tended to be used for necessary everyday chores. Also used to carry out pleasurable activities on a regular basis.	Shaw
			Respite: carers more in control of time	Shorter-term respite such as day care was felt to give structure to the carer's week along with a sense of normality as the free time matched the ebb and flow of caregiving activities.	Shaw
			Respite: use of service can be more emotionally and physically demanding than not use	There were many hassles (i.e., inconveniences, irritations and frustrations that were troublesome to deal with) involved in the preparation for respite care. These hassles were costly in terms of the physical and emotional energy involved in dealing with them, and these costs were weighed against the benefits received from respite.	Shaw
			Respite: brings organized routine	Regular attendance is also perceived by carers to provide their care recipients with a sense of structure and routine to their daily lives.	Du Preez
			Creative arts: benefits include learning, stimulation, normalizing care receivers' lives	Reported benefits included new learning, stimulation and the accessibility of the activities, which creates a sense of normalcy and altered perceptions about PWD's abilities.	Bourne
			Creative arts: artmaking helps with coping and resilience	Artmaking was seen to cope and practice resilience, where this approach allowed caregivers to make the most of their lives and to deal with an uncertain future: "It slightly reminds me of the turmoil but at the same time it says to me yes, you got through it […] Because they [a series of paintings] were part of the process of me becoming well again."	Irons
**Intervention outcomes:** This category describes potential beneficial and/or harmful effects to the caregiver from	**Social outcomes:** intervention outcomes related to impact on caregiver's social life, day-to-day routine and relationship	**Emotional social outcomes:** changes in feelings and emotional reflections of caregivers, resulting from an intervention.	Psychoeducation: Online intervention reduces stress	Caregivers subjectively identified that the online intervention helped them cope with the stress of caregiving.	Sin
			Education/skills: When needs are met, stress is reduced	Caregivers identified when personal characteristics of both patient and caregiver are considered, caregivers are included as an integral	Pritchard

For peer review only - http://bmjopen.bmj.com/site/about/guidelines.xhtml
For peer review only

participating in the interventions	with care receiver and other people.		part of the team (as they requested), and they receive timely and effective information, the level of stress is likely to reduce, and their ability to cope to be ready for discharge is likely to increase.	
		Counselling: carers appreciate conversations beyond their nursing duties	Carers also find it helpful to have conversations about other things that do not remind them of their nursing duties.	Lins
		Psychosocial: Benefits of peer support include less discomfort and satisfaction	Qualitative measures on well-being reported improvements on subjective satisfaction, and reduced feelings of discomfort and embarrassment.	Dam
		Psychosocial: group support brings relief through sharing	Qualitative interview data on support groups for only caregivers demonstrated a sense of relief through sharing problems and new social contact.	Dam
		Psychosocial: peer support brings emotional support and enjoyment	Qualitative social support outcomes demonstrated reduced feelings of social isolation, increased emotional support, and mutual sharing and enjoyment after receiving peer support.	Dam
		Counselling: simple chit chat brings relief	The participants in the control group had conversations only about general topics such as the weather, television, movies, news, or social activities. Such a conversation about common topics was found to be a helpful alternative to relieve carers.	Lins
		Counselling: self-expression beneficial	Carers benefit from being able to express their helplessness.	Lins
		Respite: respite gives physical break but not mental break/relaxation	The qualitative review also pointed out that a physical break from the care recipient was probably not sufficient in itself to provide the mental break that was needed by most carers to improve their well-being. A mental break meant freedom from worry and total disengagement from the caring role. This was only achieved in the knowledge that the care recipient was happy, safe and well cared for.	Shaw
		Respite: caregivers feel guilty to not care	The strong commitment to caring and eventual realisation that they cannot cope on their own leads not only to feelings of failure but also to feelings of guilt. The negative connotations of respite and the feelings of abandonment also contribute to guilt.	Shaw
		Respite: sense of failure when admitting need of help	The types of attitudes discussed above that drive the desire to maintain the care recipient at home can lead to a sense of failure when a need for support is acknowledged, particularly in the presence of negative attitudes to respite care itself.	Shaw
		Respite: feelings of guilt and perceived abandonment with respite	In the early stages it was felt by carers that attendance at day care could confront a care recipient's denial of their condition, and so a pretence of normality is maintained as long as possible to maintain	Shaw

For peer review only

	the integrity of the care recipient's self-image. The outcome in relation to many of the issues described above is the carer's feeling of guilt.	
Respite: more guilt among women	If respite is seen as benefiting only the carer this will be viewed in a selfish light, which can trigger guilt, particularly in women who have spent their lives caring for others	Shaw
Respite improves mood and engagement with care recipient	Carers reported increased engagement with their care recipients upon returning from adult day service attendance. This was due to their improved mood and them sharing interesting events that occurred throughout the day.	Du Preez
Creative arts: singing brings enjoyment	All six studies included comments on improved mood or well-being and three on the extended impact in terms of mood, the enjoyment of singing at home and socializing with other members outside of the group.	Bourne
Creative arts: familiar songs promote reminiscence	Participants also reported gaining enjoyment from singing familiar songs that facilitated reminiscence.	Bourne
Creative arts: benefits include self-esteem, positive affect, enjoyment, relaxation, energy, empowerment, confidence	Experiences including improved personal wellbeing, self-esteem, positive affect, enjoyment, relaxation, feeling energized, empowered and having confidence were captured in this meta-theme.	Irons
Creative arts: socioemotional connection brings sense of belonging	Papers highlighted the socio-emotional connection afforded by participation in weekly creative interventions, which gave both caregiver and care-recipient something to look forward to attending and be part of.	Irons
Creative arts: practicing skills gives cognitive stimulation and engagement	The cognitive impact of intervention participation included cognitive stimulation and engagement, through the practice of previously acquired skills (such as demonstrating musical knowledge).	Irons
Creative arts: reminiscence promoted	Creative interventions were useful for reactivating memories and afforded opportunities for memory recall of events.	Irons
Creative arts: dyads working together feel hopeful	In an intervention where caregivers and care-recipients met to create a database, this evoked positive feelings and hope for the caregivers.	Irons
Creative arts: carers reclaim identity through creative interventions	Creative interventions that allow for self-expression offer opportunities to caregivers to reclaim and transform their identity.	Irons

For peer review only - http://bmjopen.bmj.com/site/about/guidelines.xhtml
For peer review only

Creative arts: song hearing fosters emotions	In some cases, the meaning behind song lyrics evolved with the carer journey, with the song resonating and created an emotional response upon hearing the song.	Irons
Creative arts: creative interventions help for self-expression and emotional release	Participation in creative interventions allowed participants to have new experiences and to develop confidence to express oneself. For example, as one participant stated: "It probably unloads a lot of the...all the emotional feelings that you do hang on to that you don't even realise are there sometimes so it helps to get them out...it was good and it was cathartic. Even though I say I don't hold a lot you probably do have a bit of baggage and just talking about it and bringing out all those different words was, good and just very satisfying."	Irons
Creative arts: good for mindfulness	Three papers reported the recognition some caregivers had on the importance of mindfulness and living in the moment, and that these interventions created the space for this to be experienced.	Irons
Creative arts: singing decreases distress	Some papers reported that caregivers found singing allowed them to focus and concentrate on the task of singing and develop present moment awareness, allowing them to let go of other distressing or negative thoughts.	Irons
Creative arts: more clarity in thoughts and rational thinking	Themes related to increased lucidity, where caregivers reported clarity of thoughts and increased rational thinking.	Irons
Creative arts: singing benefits include better mood and well-being	All six studies included comments on improved mood or well-being and three on the extended impact in terms of mood.	Bourne
Creative arts: interventions do not reduce negative emotions, but increase positive ones	Interestingly this meta-theme did not mention the reduction of negative emotions and instead highlighted the increase in positive ones. This suggests the negative feelings were not removed by participation in creative caregiving interventions; however, caregivers and care-recipients experienced positive changes as a result of participation in these creative interventions.	Irons
Creative arts: art gallery is empowering	The structure, ambience and environment of an intervention was key to shaping and facilitating participants' experiences. For example, singing as part of a group was reported as an enabler for participation in interventions aimed at caregivers and care-recipients and an art gallery setting created an empowering space for participants	Irons
Creative arts: carers happy to see care receivers happy	The intervention activities were opportunities for playful experimentation, which was deemed the antithesis of caregiving, bringing about restorative feelings to caregivers and care-recipients. The caregivers experienced an increase in positive mood	Irons

For peer review only - http://bmjopen.bmj.com/site/about/guidelines.xhtml
For peer review only

				when they saw care-recipients expressing happiness due to participating in the intervention.	
			Mindfulness: intervention is enjoyable	Qualitatively, participants felt positive about MBSR and enjoyed participating.	Li
			Mindfulness: benefits include compassion and forgiveness	Qualitatively, participants reported the continued use of mindful based exercise and personal benefits of fostered compassion and forgiveness from the program.	Li
**Intervention outcomes:** This category describes potential beneficial and/or harmful effects to the caregiver from participating in the interventions	**Social outcomes:** intervention outcomes related to impact on caregiver's social life, day-to-day routine and relationship with care receiver and other people.	**Relational social outcomes:** changes in caregivers' relationship with either the care receiver or people around them.	Psychosocial: peer support leads to less isolation through mutual sharing	Qualitative social support outcomes demonstrated reduced feelings of social isolation, increased emotional support, and mutual sharing and enjoyment after receiving peer support.	Dam
			Respite: improved dyadic relationship	Carers reported increased engagement with their care recipients upon returning from adult day service attendance.	Du Preez
			Creative arts: dyadic facilitates reciprocity	As the group was for both partners of the dyad and reported to be accessible, this may have had a positive influence on relationships and communication, facilitating a sense of reciprocity.	Bourne
			Creative arts: singing brings more socializing	All six studies included comments on improved mood or well-being and three on the extended impact in terms of mood, the enjoyment of singing at home and socialising with other members outside of the group.	Bourne
			Creative arts: interventions give a feeling of equal dyadic relationships	Caregivers felt participation in the creative interventions allowed for interactions that were more equal and person-centred, as opposed to the usual caregiver–care-recipient dyad experiences that tended to occur outside the intervention.	Irons
			Creative arts: benefits of dyadic intervention: communication within dyads and others	As a result of participating in the intervention together, improved communication was reported by caregivers, not only within the dyads but also with other participants in the interventions and family members.	Irons
			Creative arts: interventions make dyads forget about the disease	Several papers described the opportunity for joint respite for the caregiver and care-recipient dyad to focus on the experience of the creative intervention, without needing to focus on the condition.	Irons
			Creative arts: dyad to dyad collaboration through singing	Furthermore, in a singing intervention, the social proximity of caregivers and their care-recipients to other caregivers and care-recipients were highly valued. The songs represented a shared experience, and this shared experience and collaboration was considered important.	Irons
			Creative arts: interventions make patients care recipients seem normal again	Participation in creative interventions allowed for caregivers to view the care-recipient in a different light, which led to increased satisfaction with caregiving role.	Irons

For peer review only - http://bmjopen.bmj.com/site/about/guidelines.xhtml

			Creative arts: better dyadic relationship	The process of creating a 'time capsule' database of music and photos from couples' lives evoked strong positive feelings and led to improved quality time for caregivers and their care-recipients.	Irons
			Creative arts: improved image of care receivers	The demonstration of memory and recall over the weeks was encouraging for caregivers to see with care-recipients.	Irons
			Creative arts: better relationship based on a new common interest	Meaningful connections made with care-recipient through the creation of art allowed caregivers to build social connections that were based on mutual interests rather than the caregiving.	Irons
			Creative arts: dyadic better for spousal relationships	Additionally, papers reported other social benefits, including enhanced spousal relationship and strengthened reciprocity between caregiver and care-recipient.	Irons
**Implementation outcomes:** the effects of deliberate and purposive actions to implement the interventions	**Acceptability**: degree to which an intervention is perceived to be agreeable.	**Trust, dignity and values:** the role of trust, respect, ethics and dignity in determining acceptability of the intervention.	Case management: carers trust and appreciate admiral nurses they know well	There was evidence that carers valued interacting with a professional that they knew well, and descriptions of Admiral Nurses included 'my anchor', 'life-saving' 'an angel' and 'worth her weight in gold'.	Bunn
			Counselling: carers want to have a reliable person by their side	Carers express their desire to know they have a reliable person by their side.	Lins
			Counselling: carers reluctant to unknown counsellors' advice	A carer also expressed reservations about the idea of getting good advice from an unknown person since the disease has a great variety of manifestations.	Lins
			Counselling: carers sensitive to judgement	Carers will consciously or unconsciously notice the emotional attitude of the counsellor, which can lead them to trust the counselling less and experience it as unhelpful.	Lins
			Counselling: counsellors should be familiar with cases and caregivers	Carers can appreciate the counsellor as a person and know that the counsellor is familiar with their situation and can understand the situations the carer describes.	Lins
			Counselling: thorough counselling determines trust from caregiver	At the same time, the level of thoroughness with which counsellors convey the content of the discussion has an impact on how reliable and trustworthy the carer experiences the counsellor.	Lins
			Counselling: knowing the counsellors builds rapport and is more effective	The descriptive theme of 'Knowing each other' on the other hand, might have a positive impact on the counselling, partly because when counselling is not anonymous it helps counsellors to manage the frustrations of the carers, and partly because it lays the groundwork for becoming 'Familiar and trusted', which is the relationship desired by the carers.	Lins

For peer review only - http://bmjopen.bmj.com/site/about/guidelines.xhtml
For peer review only

Counselling: service should be non-judgmental and appreciative	Non-judgmental/appreciative. This descriptive theme describes how the attitude of the counsellor needs to be non-judgemental towards the 'non-professional' care provided by the carers and needs to convey appreciation for their great efforts.	Lins
Counselling: building rapport with caregivers is important	Carers and counsellors agree on the importance of getting to know each other before counselling.	Lins
Respite: dignity of care receiver more important than symptom management	Phinney and Moody argue that community-based services that endeavor to minimize stigma and normalize attendee's experiences, rather than focus on symptom management, may attract greater utilization.	Du Preez
Respite: respect and dignity for care receiver important	Additionally, carers expect their care recipients to be treated with care, respect, and dignity while attending an adult day service.	Du Preez
Respite: separation promotes stigmatization	Whilst separation may lead to stigmatization, group inclusion for attendees with cognitive decline appeared to promote their disengagement from activities requiring cognitive demand.	Du Preez
Respite: privacy violation is problematic	Factors such as concern for privacy violation and cost of service provision diminishes use of services by carers.	Du Preez
Respite: self-sufficiency, privacy and independence determine dyad's resistance to use the service	Values of self-sufficiency, privacy and independence that were displayed by carers were also held by the care recipients themselves, which contributed to resistance to service use.	Shaw
Respite: staff don't respect care receiver	Other negative attitudes towards respite included the view that it is demeaning for the care recipient, with its connotations of babysitting. This was not helped by experiences of a perceived lack of respect for the care recipient from formal care staff.	Shaw
Respite: service is demeaning for care receiver	Other negative attitudes towards respite included the view that it is demeaning for the care recipient, with its connotations of babysitting.	Shaw
Respite: trust is important to accept sitters	Indeed, this was mentioned by carers in several of the studies – the trust that is required of people coming into the home in this capacity.	Shaw
Creative arts: staff values and perspectives important for participants' experiences	The values and perspectives of the staff and those involved in the delivery of interventions were also pertinent to participants' experience of the interventions.	Irons
Creative arts: non-judgmental and active listening environment is important	A non-judgemental group approach was considered important to enable caregivers to voice their experiences with willing listeners, to share joy and sadness and to feel valued by others.	Irons

For peer review only - http://bmjopen.bmj.com/site/about/guidelines.xhtml

For peer review only

**Implementation outcomes:** the effects of deliberate and purposive actions to implement the interventions	**Acceptability**: degree to which an intervention is perceived to be agreeable.	**Person-centeredness:** personalization and flexibility of interventions promote acceptability of the intervention by the caregivers.	Case management: flexibility is valued	The importance of the relational aspect of the role including face-to face contact and opportunities to explain their needs and concerns in a time and manner of their preference were found to be integral to the person with dementia and carer's ability to adjust to change.	Goeman
			Case management: person-centered approach is welcome	A distinguishing characteristic of Admiral Nurses was their carer-centred approach and there was evidence that carers welcomed a service which focused on them.	Bunn
			Psychoeducation: flexibility in access is valued	Common elements of the interventions that were repeatedly highlighted and attributed to high satisfaction included: flexibility in access suiting carers' lifestyles and commitments.	Sin
			Psychoeducation: personalization of interventions is valued	Common elements of the interventions that were repeatedly highlighted and attributed to high satisfaction included: […] availability of self-tailored and -paced programme allowing for individualised information and support.	Sin
			Psychoeducation: individualized approach is preferred	The qualitative data supports the use of patient and carer information and support PCIS but suggests that there is not a one-size-fits-all approach which can be used, as every patient with dementia and their carers will have different needs, preferences and responses.	Miles
			Psychoeducation: personalized information highly valued	When information was individualized, it was considered by caregivers as one of the most useful functionalities of the intervention.	Hopwood
			Psychoeducation: carers don't appreciate generic information	[…] with caregivers expressing frustration when required to review information that did not meet their specific needs.	Hopwood
			Respite: carers' and care receivers' needs-based service is more acceptable	For respite to be acceptable it must respond to both the carer's and the care recipient's needs.	Shaw
			Respite: care receiver's happiness, health and safety cause concern	Beisecker et al. reports that 'perceptions about patient unhappiness, safety, physical health, functional levels and behaviour were viewed as barriers to ADC use'.	Shaw
			Respite: distress of care receivers causes concern	Care recipient distress was a commonly reported impact, which frequently took some time to recover from after return home.	Shaw
			Respite: quality of care of care receivers is essential	A barrier to the uptake of respite services was a concern about the quality of care provided and this concern was most notable in relation to nursing homes, although home care was also sometimes problematic.	Shaw

For peer review only

			Respite: care receiver's cooperation is important to participate	Gaining the care recipient's co-operation was one of the major hassles involved, which was reported particularly in relation to day care.	Shaw
			Creative arts: dyadic intervention allows for person-centered interaction	Caregivers felt participation in the creative interventions allowed for interactions that were more equal and person-centred, as opposed to the usual caregiver–care-recipient dyad experiences that tended to occur outside the intervention.	Irons
			Creative arts: flexibility is key	One key recommendation was to engage the assistance of a qualified music therapist, who is trained in adapting an intervention according to individual needs and preferences, including addressing behavioural challenge, thus affording flexibility in structure which was seen as key to caregiver satisfaction and continued participation in the intervention.	Irons
			Creative arts: intervention should be tailored to abilities	One caregiver stated their views about their perceptions of their partner with dementia who also participated in the intervention: ". . .she loves the looking. One of the things she mentioned a number of times is how important it is, the silence at the beginning, where they really get a chance to look. And I think that for people with. . . slow processing skills, not poor but slow, that element is just so important."	Irons
			Creative arts: intervention should be tailored to care receiver's abilities	In one study, participants were critical of the intervention protocol, as some caregivers found it difficult to engage the care-recipient or experienced frustration with the care-recipients' lack of focus, suggesting there were lessons to be learned for future enhancements of the intervention.	Irons
**Implementation outcomes:** the effects of deliberate and purposive actions to implement the interventions	**Acceptability**: degree to which an intervention is perceived to be agreeable.	**Culture and language:** ethnic, cultural and religious values influence acceptability of the intervention.	Case management: multicultural staff better at supporting diverse communities	A shared approach to care was found to be vital in decreasing burden among family members and that due to their close relationship and knowledge of families, multicultural workers can offer an important perspective that is invaluable in informing the provision of carer education and support within CALD communities.	Goeman
			Counselling: native language is preferred	Receiving counselling in the native language was also shown to contribute to building trust.	Lins
			Counselling: language can be a barrier in telephone counselling	Telephone counselling of carers who do not speak the national language is problematic.	Lins
			Respite: minorities prefer sitters of same ethnicity and gender	In this context this referred to ensuring that carers were of the same ethnic group, spoke the same language and were preferably of the same gender as the care recipient.	Shaw

For peer review only - http://bmjopen.bmj.com/site/about/guidelines.xhtml

			Respite: culture is important in determining demand for respite	Personal and cultural attitudes to the caring role and to the function and purpose of respite services may impact on the carers' perceptions of their needs for respite.	Shaw
			Respite: respect for culture and religious identity valued	Other important considerations for carers were that food was appropriate for their religion, for example vegetarian, and that the service was sensitive to other cultural and religious differences.	Shaw
**Implementation outcomes:** the effects of deliberate and purposive actions to implement the interventions	**Acceptability**: degree to which an intervention is perceived to be agreeable.	**Setting of the intervention:** environmental, social and structural components of the interventions that promote or hinder acceptability.	Psychoeducation: rapport with professionals and peers harder online	In a small number of studies, despite extensive recruitment efforts and provision of equipment and technical support, recruitment and completion rates still struggled as some carers reported finding it difficult to strike up a rapport with the professionals and their carer-peers and would still prefer the conventional delivery media using face to face group or individual meetings.	Sin
			Psychoeducation: support from carer networks and professionals is valued	Common elements of the interventions that were repeatedly highlighted and attributed to high satisfaction included: […] and network support through online forums with other carers and access to professionals.	Sin
			Psychoeducation: interaction among participants is important	Negative from one study: lack of interaction with other participants.	Boots
			Respite: in home care preferred over day care	Because of difficulties with day care many carers in the studies reported a preference for in-home care as being less disruptive for the care recipient.	Shaw
			Respite: turnover of staff is disruptive	One other major area of difficulty was related to the lack of continuity of care and the high turnover of staff in support services.	Shaw
			Respite: caregivers not informed about activities at respite	Family carers have little to no contact with the adult day service other than to ready their care recipient for the day's attendance and have little knowledge of how their care recipient spends their time while attending adult day service.	Du Preez
			Creative arts: carers appreciate having a special place	One paper found that caregivers in an intervention considered themselves ordinary users of a community place that was "somewhere different" and valued as a special place.	Irons
			Creative arts: group singing is better	The structure, ambience and environment of an intervention was key to shaping and facilitating participants' experiences. For example, singing as part of a group was reported as an enabler for participation in interventions aimed at caregivers and care-recipients.	Irons
			Creative arts: creative interventions offer interactive space	Papers presented themes around caregivers' and care-recipients' appreciation of a time and space afforded by the creative interventions to enable greater communication, and a natural	Irons

For peer review only

				development of social interactions both with the caregiver and other participants in the interventions.	
			Creative arts: gallery setting and facilitator important for art viewing	Two papers highlighted the importance of the gallery setting and facilitator characteristics.	Bourne
**Implementation outcomes:** the effects of deliberate and purposive actions to implement the interventions	**Acceptability**: degree to which an intervention is perceived to be agreeable.	**Other:** other determinants of acceptability, not groupable.	Psychoeducation: eHealth highly acceptable	In general, carers' perceived acceptability of the eHealth interventions across the studies synthesized was high.	Sin
			Psychoeducation: relatable experiences important	Experiential similarity is seen as important	Smith
			Creative arts: caregivers skeptical about their contributions to the interventions	Prior to joining the interventions, caregivers had deliberations around what to expect, including assumptions that they had nothing to contribute to the intervention.	Irons
			Mindfulness: mindfulness intervention is acceptable	Total of 31 FCs were included in the trial, overall, findings confirmed the feasibility and acceptability of the intervention for FCs, and the interviews, revealed that the intervention was associated with several participant-identified benefits and no severe adverse effects.	Aldaken
**Implementation outcomes:** the effects of deliberate and purposive actions to implement the interventions	**Feasibility:** the extent to which a new intervention can be successfully used or carried out within a given setting. Typically invoked retrospectively as a potential explanation of an intervention's success or failure, as reflected in poor recruitment, retention, or participation rates.	**Recruitment:** factors related to raising awareness about an intervention and recruiting suitable participants.	Case management: lack of clarity on service content	Carers' lack of clarity about the Admiral Nurse role was also observed; 'never really found out what the Admiral Nurse service was offering'.	Bunn
			Psychoeducation: recruitment issues in tech interventions	In a small number of studies, despite extensive recruitment efforts and provision of equipment and technical support, recruitment and completion rates still struggled.	Sin
			Respite: advise to use comes from primary care	The most accessible location for advice is the primary health-care centre.	Shaw
			Respite: lack of information on available services	Not only was there evidence of limited access to respite services but also there was a low awareness of the availability of services.	Shaw
			Respite: GPs not effective providing support despite being most accessible	The most accessible location for advice is the primary health-care centre, but it was suggested by more than one study that GPs do not appear to be providing appropriate support and information.	Shaw
			Respite: awareness on services low in minorities	Not only was there evidence of limited access to respite services but also there was a low awareness of the availability of services.	Shaw
			Respite: medical staff not informed about services	Medical practitioners were identified as having limited knowledge of community support services and access to information resulting in poor referral processes and therefore, poor utilization by family carers and people living with dementia.	Du Preez

For peer review only

**Implementation outcomes:** the effects of deliberate and purposive actions to implement the interventions	**Feasibility:** the extent to which a new intervention can be successfully used or carried out within a given setting. Typically invoked retrospectively as a potential explanation of an intervention's success or failure, as reflected in poor recruitment, retention, or participation rates.	**Accessibility:** physical, time-related or other external factors that limit or facilitate access to the interventions.	Case management: limiting admissions to confirmed dementia cases only	The failure factors were: […] and not including patients without a confirmed diagnosis of dementia.	Goeman
			Psychoeducation: access and time may be of concern for tech interventions	A few studies reported difficulties in even recruiting and retaining carers due to obstacles of access, cost, and time regarding use of technology.	Sin
			Counselling: telephone counselling is accessible	Carers find the telephone a good tool for receiving counselling as it helps avoid the stress involved in co-ordinating an appointment.	Lins
			Respite: transport may be an issue	There were a number of reports of complaints concerning transport, which included lack of transport in rural areas and a general unreliability of transport services.	Shaw
			Respite: waiting lists problematic	Waiting lists proved to be an issue for nursing home care and night care.	Shaw
			Respite: transport may be an issue	Transport was reported to be a significant barrier to utilization for carers. Use of public transport to access adult day service as opposed to transport being provided was found to be difficult and time-consuming.	Du Preez
			Creative arts: dyadic art interventions are accessible	As the group was for both partners of the dyad and reported to be accessible, this may have had a positive influence on relationships and communication, facilitating a sense of reciprocity.	Bourne
			Creative arts: singing is accessible for all	Caregivers highlighted other parts of the singing group experience to be important. These included the accessibility of singing for all.	Bourne
			Creative arts: art-viewing is accessible	Reported benefits included new learning, stimulation, and the accessibility of the activities, which creates a sense of normalcy and altered perceptions about PWD's abilities.	Bourne
**Implementation outcomes:** the effects of deliberate and purposive actions to implement the interventions	**Feasibility:** the extent to which a new intervention can be successfully used or carried out within a given setting. Typically invoked retrospectively as a potential explanation of an intervention's success or failure, as reflected in poor recruitment, retention, or participation rates.	**Service availability:** factors related to coverage of services in terms of offered times and availability of staff.	Case management: access easy, but contact difficult outside working hours	There was evidence that making and maintaining contact with the service initially was found to be easy in the majority of cases, but only just over half of the respondents knew who to contact outside Admiral Nurse's hours.	Bunn
			Counselling: 24/7 service is a must	Carers agreed that 24-hour availability of telephone counselling is necessary.	Lins
			Counselling: answering machines insufficient	Existing arrangements, such as answering machines for the times when counselling is not available, were not sufficient.	Lins
			Counselling: telephone counselling is flexible	It is a very comfortable means of counselling because carers do not have to leave their homes and its flexibility allows sessions to be fitted into their daily nursing routines.	Lins

For peer review only

For peer review only - http://bmjopen.bmj.com/site/about/guidelines.xhtml

			Respite: flexibility of day care is key	flexibility of the DC in relation to available days and times, and the possibility of a regular or intermittent frequency.	Maffioletti
			Respite: timing and flexibility can be problematic	A major barrier to the uptake of respite services was a lack of response to carer needs in terms of timing and flexibility of service provision.	Shaw
			Respite: working carers not accommodated schedule-wise	In another study day care was only offered for 3 days a week and finished at 3pm, which did not accommodate the needs of working carers.	Shaw
			Respite: weekends and evenings not covered	Problems with day care were the lack of weekend and evening provision.	Shaw
			Creative arts: different preferences on length of intervention	Some participants shared disappointment that the intervention was time-limited, whilst others said that it would be difficult to continue the sessions long-term due to care-giving responsibilities.	Bourne
			Mindfulness: carers appreciate informality and availability of service	Chronic conditions: Qualitative results indicated that the informal practice was particularly helpful and could be used at any time.	Li
			Mindfulness: dyadic mindfulness is more practical to attend	Also, it might be more feasible for caregivers because they would not have to leave care recipients alone.	Li
**Implementation outcomes:** the effects of deliberate and purposive actions to implement the interventions	**Feasibility:** the extent to which a new intervention can be successfully used or carried out within a given setting. Typically invoked retrospectively as a potential explanation of an intervention's success or failure, as reflected in poor recruitment, retention, or participation rates.	**Adoption and retention:** factors related to continued use of services or drop-out from services.	Psychoeducation: technical issues may arise	Usability problems (such as oral communication/chat quality, audio-visual function failure) were also identified as attributing to high drop-out rates (up to 50%) in some studies.	Sin
			Psychoeducation: older spousal carers find technology challenging	Most studies included focused on carers of dementia patients, and this was also the area with the most frequently reported problems in access and usability, as encountered by a group of largely elderly spousal carers who were often not familiar with ICT.	Sin
			Psychoeducation: dropout due to severe patients and carer time	Reasons for participant withdrawal included ill health of the person cared for and carer constraints.	Smith
			Psychoeducation: older adults find technology challenging	There appears to still be a gap between those who use or can use the internet and those who don't, with a study in 2015 highlighting that almost all adults over 70 years of age had difficulty using the intervention.	Hopwood
			Respite: carers need to prepare patients for respite	Carers reported having difficulty assisting their care recipient with their activities of daily living in preparation for the day's attendance.	Du Preez

For peer review only - http://bmjopen.bmj.com/site/about/guidelines.xhtml
For peer review only

			Respite: multicomponent interventions increase utilization	Adult day services that offer comprehensive services that engage dementia caregivers by way of phone calls or one-on-one carer meetings to address their areas of concern, invite carer collaboration in planning meetings, provide carer education, counseling and case management were seen to facilitate increased service utilization and delay early institutionalization.	Du Preez
			Respite: severe patients difficult to prepare and reluctant to participate	Carers reported having difficulty assisting their care recipient with their activities of daily living in preparation for the day's attendance. This was exacerbated by their care recipient's co-morbidities and resistance to attendance.	Du Preez
			Respite: decline in function leads to exclusion from service	Another barrier to use is attendance being conditional upon attendees being able to independently self-care.	Du Preez
			Respite: person centered activities increase utilization	Adult day services' ability or inability to offer a variety of activities that were person-centered and held meaning for attendees was a motivating factor for use.	Du Preez
			Respite: services minimizing stigma leads to greater utilization	Whilst separation may lead to stigmatization, group inclusion for attendees with cognitive decline appeared to promote their disengagement from activities requiring cognitive demand. However, Gaugler found staff overcame these issues through validation and one-to-one interaction.	Du Preez
			Respite: timing and flexibility may be barriers	A major barrier to the uptake of respite services was a lack of response to carer needs in terms of timing and flexibility of service provision.	Shaw
			Respite: severe PwD excluded	With confusion comes inappropriate behaviours, which were difficult for both in-home and institutional respite carers to accommodate, resulting in exclusion of the care recipient from the service.	Shaw
			Respite: service is used for maintaining nutrition	As the care recipient's condition deteriorates carers express concern for safety and the need for a sitter may be expressed. There are also concerns about maintaining care recipient health in relation to maintaining adequate nutrition.	Shaw
			Respite: progression of disease triggers use of service	As the care recipient's condition deteriorates carers express concern for safety and the need for a sitter may be expressed.	Shaw
**Implementation outcomes:** the effects of deliberate and purposive actions to implement the interventions	**Feasibility:** the extent to which a new intervention can be successfully used or carried out within a given setting. Typically invoked retrospectively as a potential	**Systemic factors:** Larger structural factors related to the organization of health and social care services.	Case management: primary care not involved is hindering	The failure factors were: […] little or no involvement of primary care specialists.	Goeman
			Case management: competition to deliver care is a barrier	The failure factors were: […] competition for delivering care.	Goeman

For peer review only - http://bmjopen.bmj.com/site/about/guidelines.xhtml

	explanation of an intervention's success or failure, as reflected in poor recruitment, retention, or participation rates.		Case management: medical staff not collaborating with admiral nurses	At times, carers felt that GPs, specialists and care managers did not work with or communicate with Admiral Nurses; 'Care manager doesn't seem to communicate with Admiral Nurse'.	Bunn
			Education/skills: carers appreciate having one source of information	Caregivers emphasised the importance of having one central source for information, for example a key worker to liaise with during visits.	Pritchard
			Respite: systems do not respond to need	Additionally, imperatives of service delivery systems place carers in a vulnerable position seemingly prioritizing these systems above the needs of caregivers. Utilization of services is often determined by effective referral by health professionals.	Du Preez
			Respite: bureaucracy unavoidable and intruding upon care receiver's privacy	Furthermore, interaction with adult day service providers requires carers to adopt institutional processes and intrudes upon their care recipient's privacy, albeit it to promote that person's quality of life.	Du Preez
**Implementation outcomes:** the effects of deliberate and purposive actions to implement the interventions	**Feasibility:** the extent to which a new intervention can be successfully used or carried out within a given setting. Typically invoked retrospectively as a potential explanation of an intervention's success or failure, as reflected in poor recruitment, retention, or participation rates.	**Other:** other determinants of feasibility, not groupable.	Mindfulness: mindfulness intervention is feasible	Total of 31 FCs were included in the trial, overall, findings confirmed the feasibility and acceptability of the intervention for FCs, and the interviews, revealed that the intervention was associated with several participant-identified benefits and no severe adverse effects.	Aldaken
**Implementation outcomes:** the effects of deliberate and purposive actions to implement the interventions	**Appropriateness:** perceived suitability and usefulness of intervention to address needs of caregivers.	**N.A.**	Case management: some needs are overlooked	Case management offered potential benefit to people with dementia, their carers and community-based professionals through continuity of care by a named trusted individual that could act proactively to prevent a crisis. However, it was also shown that needs may be overlooked.	Goeman
			Case management: multidisciplinary and continued services meet more needs	The specialist multi-agency home support service demonstrated greater flexibility and responsiveness to the particular needs and circumstances of service users and family carers, who were encouraged to participate in routine decision-making and activities. By sharing responsibilities, the specialist service helped reduce carer stress and prevent crises. These outcomes depended on the configuration of the service, including multidisciplinary health and social services input, care worker autonomy and independence, continuous reassessment of clients' circumstances and preferences and the capacity to develop long-term relationships, through care worker continuity. The standard service, which used a task orientated approach, lacked these characteristics.	Goeman

For peer review only

Case management: integration of care is necessary	It was found that the success for case management in long-term dementia care concern the expert knowledge of case managers; investment in a strong provider network and coherent conditions for effective inter-organisational cooperation to deliver integrated care.	Goeman
Case management: patient-centeredness is appropriate	It is suggested that the appropriate way of offering case management is through a patient-centred approach.	Goeman
Case management: admiral nurse as a liaison among care providers	A commonly reported component of the Admiral Nurse role was facilitation, which included liaison with other health and social care services; 'She makes sure she liaises with all the agencies concerned with mum's care'.	Bunn
Psychoeducation: Internet based support not for all	Qualitative evaluations of the interventions demonstrated positive views from most caregivers toward internet-based support interventions, although it is clear that not all would benefit from such interventions.	Hopwood
Psychoeducation: carers don't appreciate general information	[…] with caregivers expressing frustration when required to review information that did not meet their specific needs.	Hopwood
Psychoeducation: interaction with professionals more beneficial than just information	However, other multiple-component interventions evaluated with qualitative methods found that caregivers found other components, such as interaction with professionals, more beneficial than information.	Hopwood
Education/skills: Face-to-face and repeated information preferred	Appropriate modality and timing of information means the person may require information to be presented in different ways (e.g., in writing, diagrams) repeated on several occasions and in person, not over the phone.	Pritchard
Education/skills: differences between carers should be considered	The interaction between the personal characteristics of both the patient and caregiver is not always considered by health professionals. However, because the skills of the caregiver can impact the patient and vice versa, considering the interaction is paramount. Personal characteristics is also influenced by and influences organisational culture and the level of information provision.	Pritchard
Education/skills: carers' needs addressed related to information, coping mechanisms, assessment of home environment	The interventions addressed the need for clear information about the disease, identification of psychological response, development of coping mechanisms and assessment of the home environment.	Pritchard
Counselling: counsellors inform carers about other suitable services	Together with the carers, counsellors identify the most suitable services, which helps the carers to understand that they have access to an extensive help system in addition to telephone counselling.	Lins

For peer review only - http://bmjopen.bmj.com/site/about/guidelines.xhtml

			Counselling: more severe cases need more counselling	Counselling for carers is most needed, for example, in the case of an accident or during the final phase of the disease.	Lins
			Counselling: service needed to face potentially escalating crises	Carers need assistance most acutely in difficult situations when they feel insecure and see that an escalation is possible.	Lins
			Respite: respite seen as more appropriate for severe patients	Carers of recipients with dementia felt that day care, for example, was more appropriate for more severely demented people who were unaware of their surroundings.	Shaw
			Creative arts: group art not always beneficial	Two studies reported neutral or negative comments regarding participation in the group. These included some caregivers reporting initial apprehensiveness about attending, no effect on themselves but effects on the PWD.	Bourne
			Creative arts: carers cannot relax due to worry about PwD	These included some caregivers reporting initial apprehensiveness about attending, no effect on themselves but effects on the PWD, and difficulties with relaxation due to concerns about the PWD.	Bourne
			Creative arts: song writing addresses needs not met by other groups	The song writing group filled a gap for caregivers that were not met by other support groups.	Irons
			Creative arts: members of dyads don't have same needs	It may have been difficult to structure and time the delivery of an intervention to appeal to both caregiver and care-recipient, as illustrated by this quote: ''I think it drew him out more than it drew me out. And why that is, I don't know."	Irons
			Mindfulness: not all relaxing or benefitting	Although, these potential benefits were not universal, and some participants did report difficulty fully relaxing during the process.	Parkinson
**Implementation outcomes:** the effects of deliberate and purposive actions to implement the interventions	**Sustainability:** factors associated with sustained, long-term use of the intervention.	**N.A.**	Psychoeducation: shared experiences in peer support leads to sustainability	Carers were more likely to have successful peer support relationships and to continue meeting after the intervention ended, if they were similar on the shared experience of caring.	Smith
			Respite: experience of benefit and opportunistic use leads to regular use	The need for respite for particular social events or occasions such as conferences, weddings and holidays triggered the use of respite, and once the benefit was experienced and proved successful it was used again on a more regular basis.	Shaw
			Mindfulness: mindfulness interventions sustained by carers	Qualitatively, participants reported the continued use of mindful based exercise and personal benefits of fostered compassion and forgiveness from the program.	Li
**Implementation outcomes:** the effects of deliberate and purposive actions to implement the interventions	**Cost:** financial costs associated with implementing or using the intervention.	**N.A.**	Case management: funding is an issue	The failure factors were: […] inadequate or no structural funding.	Goeman

For peer review only

Psychoeducation: cost is an issue	A few studies reported difficulties in even recruiting and retaining carers due to obstacles of access, cost, and time regarding use of technology.	Sin
Respite: cost is an issue	Because of the different organizational processes involved in the different countries, cost issues may vary.	Shaw
Respite: cost is an issue	Factors such as concern for privacy violation and cost of service provision diminishes use of services by carers. […] Affordability is an issue for some carers who constantly weigh their care recipients needs against the economic burden of meeting those need.	Du Preez

Abbreviations: QoL, Quality of Life; MBIs, Mindfulness-Based Interventions; LTCs, Long Term Conditions; PwD, Person with Dementia; MBSR, Mindfulness-Based Stress Reduction; PCIS, Patient and Carer Information and Support; CALD, Culturally And Linguistically Diverse; FC, Family Caregiver; DC, Day Care; ICT, Information and Communication Technology

For peer review only

For peer review only - http://bmjopen.bmj.com/site/about/guidelines.xhtml

GROOVE tool: Graphica

DOI: 10.17605/OSF.IO/U2MS4

What is GROOVE?

GROOVE (**G**raphical **R**epresentation of **O**verlap for **OVE**rviews) is a tool that provides a visual display

Starting with a simple matrix of evidence, this tool will automatically inform:
- The corrected covered area for the whole matrix.
- The number of unique primary studies.
- The number of overlapped primary studies (and in how many reviews they are included).
- A detailed assessment of the corrected covered area between every possible pair of reviews (nodes
- The number of nodes with low, moderate, high and very high overlap.
- Optionally, an assessment of the corrected covered area corrected by structural missingness in the

This tool is based on a previously published approach. If you are not familiar with the quantitative ass
Neugebauer EAM, Eikermann M. Systematic review finds overlapping reviews were not mentioned in

How to use this tool:

You only have to enter data on the "1. Matrix" sheet. The matrix supports up to 70 systematic reviews
You will get all the results on the "GROOVE" sheets (colour or greyscale version).

1. Go to the "1. Matrix" sheet.
2. Write all the systematic reviews ID in the columns, starting in the C5 cell.
3. Write all the primary studies ID in the rows, starting in the A6 cell.
4. Optionally, you can write the topic of your overview in the B2 cell, and the references of each prim
5. Mark with a "1" the intersections representing primary studies included in their respective system
6. Go to the "2.1 GROOVE - colour version" or "2.2 GROOVE - grayscale version" tab. You will find the moderate, high and very high overlap, and the number of unique primary studies and overlapped pri

Advanced usage: Incorporating structural missingness

While you are building the matrix of evidence, you will notice that it should be impossible to mark a ' published in 2015 can not be included in a systematic review published in 2013; this would be a "chro

The GROOVE tool provides the option to incorporate structural missingness into the results. In order
1. Follow all the steps mentioned in the previous section.
2. When you finish populating the matrix of evidence with "1"s, you can mark the cells with structura
3. Once you finish, go to the "2.1 GROOVE - colour version" or "2.2 GROOVE - grayscale version" tab.
4. The Graphical Representation of Overlap for OVErviews incorporating structural missingness will b structural missingness in the matrix, this figure will be the same as the one displayed in F1.

Example: How to enter data into the matrix

MATRIX OF EVIDENCE

TOPIC:

Study ID	Reference	Bonfill 2012	Urrútia 2014
	**Primary Studies**	**Systematic**	
Bonham 1980		1	
Lennon 1980		1	1
Mercury 1991		1	
Harrison 2001			1
Jones 2007			1
McCartney 2010			1
Taylor 2011			
Page 2012			
Deacon 2013		X	
May 2013		X	1
Starr 2019		X	X
Plant 2020		X	X

2. Write all the primary studies ID here (references are optional)

Overall results

Number of columns (number of reviews)	c	4
Number of rows (number of index publications)	r	12
Number of included primary studies (including double counting)	N	20
Covered area	N/(rc)	41,67%
Corrected covered area	(N-r)/(rc-r)	22,22%
Interpretation of overlap	Very High overlap	
Structural Zeros	X	8
Corrected covered area (adjusting by structural zeros)	(N-r)/(rc-r-X)	28,57%
N° of non-overlapped primary studies	In 1 SR	7
Number of overlapped primary studies	In 2 SRs	2
	In 3 SRs	3
	In 4 SRs	0
	In 5 SRs	0
	In 6 SRs	0
	In 7 SRs	0
	In 8 SRs	0
	In 9 SRs	0
	In 10 SRs	0
	In 11 SRs	0
	In 12 SRs	0
	In 13 SRs	0
	In 14 SRs	0
	In 15 or more SRs	0

5. Go to the "2.1 GROOV grayscale version" sheet.

Graphical Representat

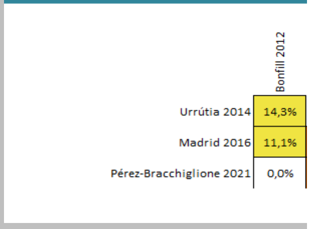

Graphical Represent

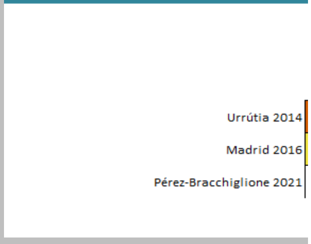

Cochrane Chile

Cochrane Iberoamérica

For peer review only

al Representation of Overl

of the overlap of primary studies among systematic reviev

s), and a graphical representation of this assessment.

matrix.

sessment of overlap using the corrected covered area form
every other overview. Journal of Clinical Epidemiology201

rs and 1000 unique primary studies.

nary study in the B column, starting with B6.
natic reviews. It is expected for a single primary study to be
e corrected covered area (CCA) for the whole matrix, the C
imary studies.

"1" in some cells. A cell that can not acquire other value th
onological structural missingness".

r to do this, you will have to:

al missingness with an "X". You will notice that the cell will
You will notice that the D9 cell will provide the CCA formu
be displayed in the "2.1 GROOVE - colour version" and "2.2

view only

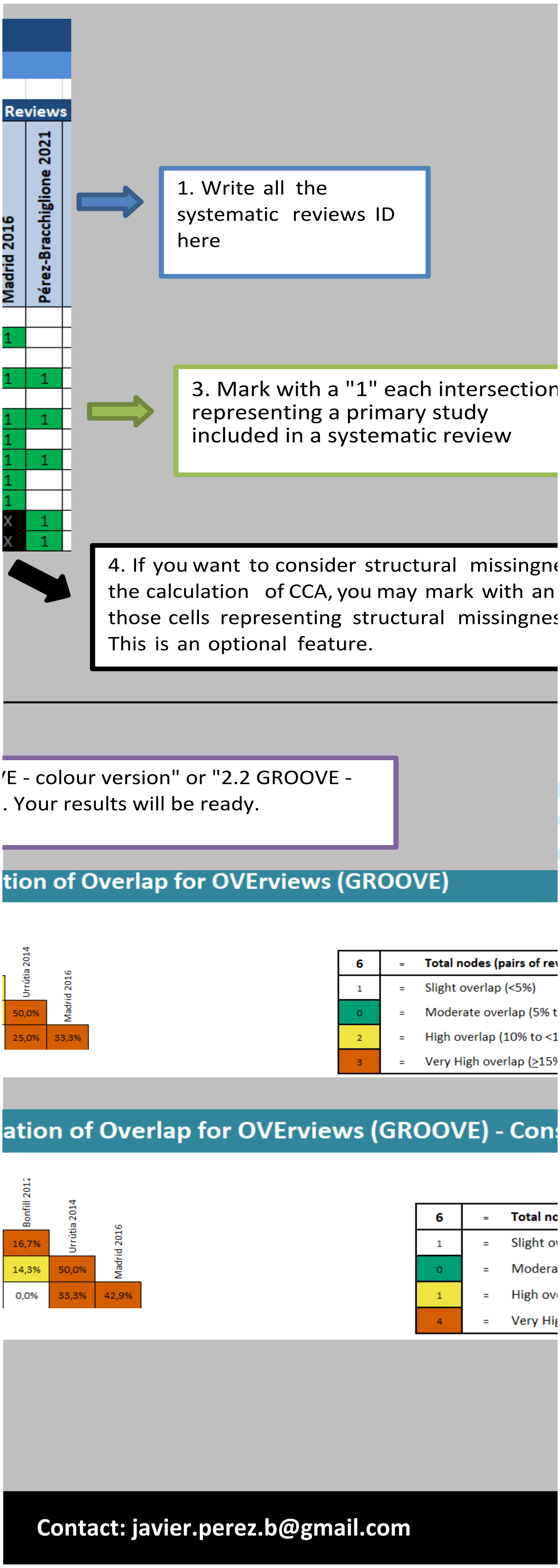

For peer review only

Universidad de Valparaíso
CHILE

U[illegible]

For peer review only

ap for OVErviews

ws.

ıula, we suggest you to read the original publication: Pieper D, Antoine S-L, Mathes T,
ı4;67(4):368–75.

e included in multiple systematic reviews.
CA for each pair (or nodes) of systematic reviews, the number of nodes with low,

han 0 is called "structural missingness" or "structural zero". For example, a primary study

l turn black.
ula corrected by structural missingness.
2 GROOVE - grayscale version" tabs, starting in the cell CA1. If you do not mark any

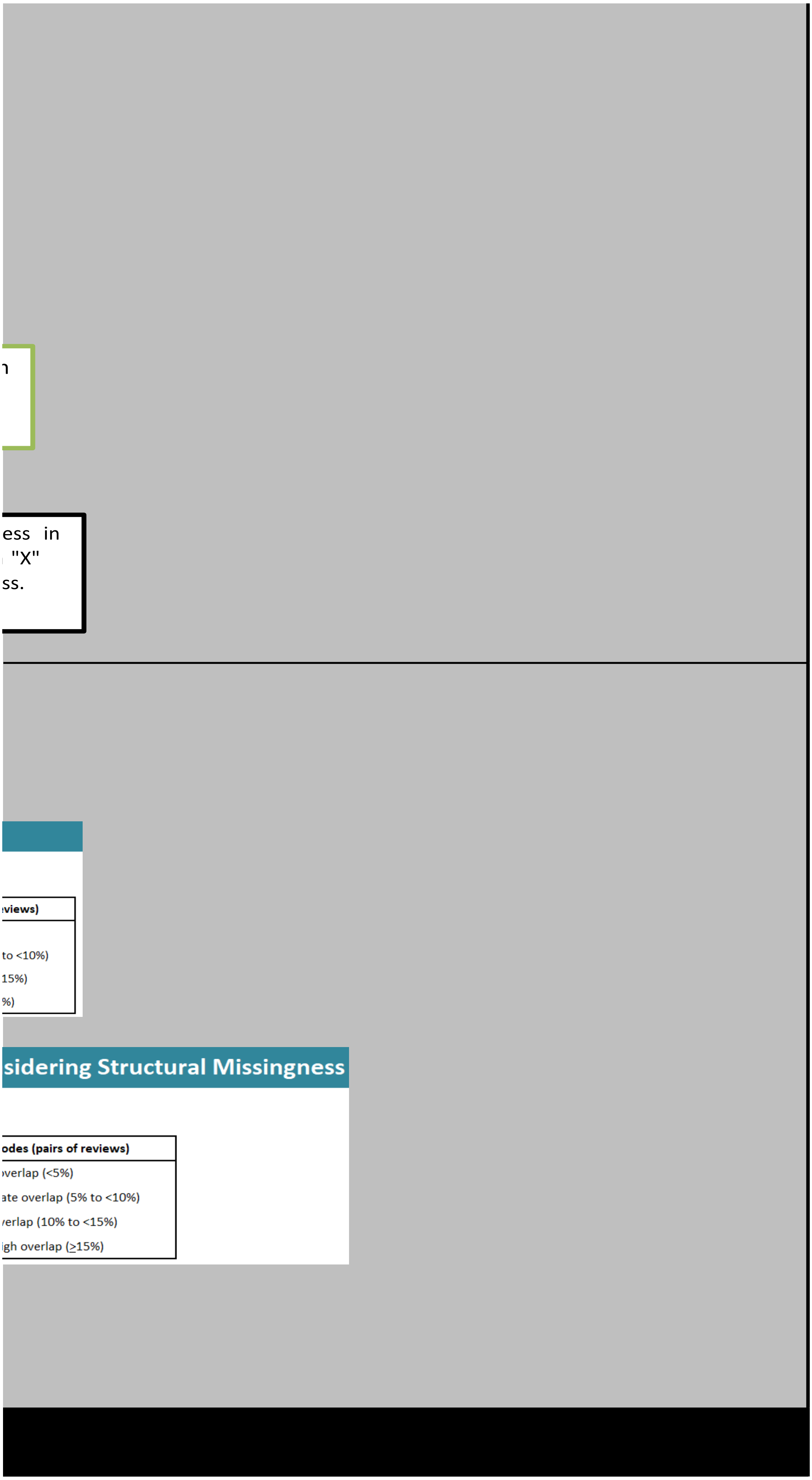
ess in
"X"
ss.
views)
to <10%)
15%)
%)
sidering Structural Missingness
odes (pairs of reviews)
verlap (<5%)
ate overlap (5% to <10%)
erlap (10% to <15%)
igh overlap (≥15%)

UAB
niversitat Autònoma
de Barcelona

CIESAL
CENTRO INTERDISCIPLINARIO
DE ESTUDIOS EN SALUD
UNIVERSIDAD DE VALPARAÍSO

For peer review only

MATRIX OF EVIDENCE

TOPIC: Informal caregiving UR

	A	B	C	D	E	F	G	H	I	J
4	Primary Studies		Systematic Reviews							
5	Study ID	Reference (optional)	807 Irons 2020	1048 Gabriel 202	2195 Shim 2020	2367 Hopwood 2	2370 Guay 2017	2491 Vandepitte	2524 Zabihi 2020	3197 Mason 2007
6	Camic2014		1							X
7	Bourne2019		1			X	X	X		X
8	Tamplin2018		1				X	X		X
9	Hanser2011		1							X
10	Davidson2014		1							X
11	Camic2011		1							X
12	Baker2018		1							X
13	Baker2012		1							X
14	Badger2013	Segrin, Hepworth		1						X
15	Badger2013	Segrin, Pasvogel		1						X
16	Badger2011			1						X
17	Belgacem2013			1						X
18	Clark2013			1						X
19	Kayser2010			1						X
20	Meyers2011			1						X
21	Northouse2014			1						X
22	Northouse2013			1						X
23	Shaw2016			1						X
24	Titler2017			1				X		X
25	Mosher2018			1			X	X		X
26	Brown2016				1			X		X
27	Oken2010				1					X
28	Black2013				1					X
29	Danucalov2017				1			X		X
30	Lavretsky2013				1					X
31	Leach2015				1					X
32	Waelde2017				1			X		X
33	Dowling2014				1					X
34	Davis2011									X
35	Ducharme2005									
36	Gaugler2015									X
37	McCallion1999									
38	Paun2015									X
39	Robison2007									X
40	Beauchamp2005					1	1			
41	Blom2015					1	1			X
42	Chih2013						1			X
43	Cristancho-Lacroix2015					1	1			X
44	DuBenske2014						1			X
45	Eames2013						1			X
46	Kim2013						1			X
47	McLaughlin2013						1			X

	A	B	C	D	E	F	G	H	I	J
48	Namkoong2012						1			X
49	Pierce2009						1			X
50	Smith2012						1			X
51	Torkamani2014						1			X
52	Lai2012									X
53	Hendrix2013									X
54	Porter2011									X
55	Sherwood2012									X
56	Boele2013									X
57	Fegg2013									X
58	Ledderer2013									X
59	McLean2013									X
60	Mitchell2013									X
61	Barrera2014									X
62	Badr2015									X
63	Dionne-Odom2015									X
64	Leow2015									X
65	Sun2015									X
66	Hendrix2016									X
67	Belle2006								1	
68	Burgio2003									
69	Chodosh2015									X
70	Czaja2013									X
71	Eisdorfer2003									
72	Gallagher-Thompson2003								1	
73	Gallagher-Thompson2007								1	X
74	Gallagher-Thompson2008									X
75	Gallagher-Thompson2015									X
76	Glueckauf2012									X
77	Gonyea2014									X
78	Winter2007							1		X
79	Au2014								1	X
80	Au2015								1	X
81	Coon2003								1	
82	Gallagher-Thompson2000								1	
83	Gonyea2006							1	1	
84	Losada2011								1	X
85	Moore2013								1	X
86	Read2016								1	X
87	Steffen2016								1	X
88	Vasquez2016								1	X
89	ArtasoIrigoyen2002									1
90	Baumgarten2002							1		1
91	Burch2001									1
92	Donaldson1989									1
93	Hedrick1993									1
94	Quayhagen2000									1
95	Zank2002							1		1
96	Zarit1998									1
97	Conlin1992									1
98	Kosloski1993									1
99	Montgomery1989									1
100	Lawton1989									1

	A	B	C	D	E	F	G	H	I	J
101	Grant2003							1		1
102	Johnson1988									1
103	Montgomery1985									1
104	Neibuhr1989									1
105	Wishart2000									1
106	Robertson2002									1
107	Holm2003									1
108	Wells1987									1
109	Clark2000									1
110	Lund1995									1
111	deRotrou2011							1		X
112	Gavrilova2009							1		X
113	Guerra2011									X
114	Hepburn2001							1		
115	Kurz2010							1		X
116	Martin-Carrasco 2009							1		X
117	Pahlavanzadeh2010									X
118	Newcomer1999									
119	Challis2002									
120	Leung2004									
121	Beland2006									
122	Vickrey2006							1		
123	Callahan2006									
124	ChienLee2008									X
125	Morales-Asencio2008									X
126	Wolff2009									X
127	Hebert2010									X
128	Lam2010									X
129	ChienLee2011									X
130	Jansen2011									X
131	Parsons2012									X
132	Janse2014									X
133	McNeilVroomen2015									X
134	Akkerman2004									
135	Arango-Lasprilla2014									X
136	Bourgeois2002									
137	EtxeberriaArritxabal2005									
138	EtxeberriaArritxabal2014									X
139	Gendron1996									
140	Gonyea2016									X
141	Gonzalez2014									X
142	Hebert2003									
143	Kamkhagi2015									X
144	Livingston2013							1		X
145	Livingston2014							1		X
146	Losada2005									
147	Losada2015									X
148	LosadaBaltar2004									
149	Marquez-Gonzalez2007									X
150	Passoni2014							1		X
151	WilzSoellner2016									X
152	Wilz2017									X
153	Forster2009									X

	A	B	C	D	E	F	G	H	I	J
154	Marsden2010									X
155	Perrin2010									X
156	Shyu2010									X
157	Galvin2011									X
158	Forster2013									X
159	Nayeri2014									X
160	Ostwald2014									X
161	Forster2015									X
162	Wang2015									X
163	Duncan2017									X
164	Aguirrezabal2013									X
165	Bishop2014									X
166	Fens2014									X
167	Chang1999									
168	Farran2007									X
169	Hepburn2005									
170	Marriott2000									
171	Zarit1987									
172	Bass2003									
173	Chu2000									
174	Dias2008									X
175	Eloniemi-Sulkava2001									
176	Eloniemi-Sulkava2009							1		X
177	Hinchliffe1995									
178	Hou2014									X
179	O'Donnel2013									X
180	Whitebird2013									X
181	Chang2004									
182	Davis2004									
183	Finkel2007									X
184	Gant2007									X
185	Glueckauf2007									X
186	Salfi2015									X
187	Steffen2000							1		
188	Tremont2008							1		X
189	Wilz2011									X
190	Draper2007									X
191	Grant2002									
192	Hartke2003									
193	Kalra2004									
194	Larson2005									
195	Mant2000									
196	Pierce2004									
197	Yoo2007									X
198	Banet1997									
199	Chinchai2010									X
200	Chiu2008					1				X
201	Downes1993									
202	Ellis2005									
203	Evans1988									
204	Frank2000									
205	Hoffmann2007									X
206	Johnson2000									

	A	B	C	D	E	F	G	H	I	J
207	Johnston2007									X
208	Lomer1987									
209	Lowe2007									X
210	Maasland2007									X
211	Mant1998									
212	O'Connel2009									X
213	Pain1990									
214	Rodgers1999									
215	Smith2004									
216	Connell2009							1		X
217	Corry2015									X
218	Kwok2013									X
219	Martindale-Adams2013									X
220	NCT00646217, 2008									X
221	Pfeiffer2014									X
222	Piamjariyakul2015									X
223	Powell2014									X
224	Shum2014									X
225	SmithToseland2006									
226	Toye2016									X
227	Wray2010									X
228	Kohle2017									X
229	Lloyd2018									X
230	Badger2007									X
231	Castro2002									
232	Danucalov2013									X
233	Hirano2011									X
234	King1997									
235	King2002									
236	Lowery2013									X
237	VanPuymbroeck2007									X
238	Campbell2007									X
239	Walsh2007									X
240	Carter2006									
241	McMilan2006									
242	Northouse2005									
243	Northouse2007									X
244	Dioquino2016									X
245	Drossel2011									X
246	Epstein-Lubow2011									X
247	Franco2010									X
248	Hoppes2012									X
249	Norouzi2014									X
250	Paller2015									X
251	Burgener2011									X
252	Milbury2015									X
253	Milbury2018									X
254	Winters-Stone2016									X
255	Barnes2015									X
256	Lamb2018									X
257	Maci2012									X
258	Marques2015									X
259	Yu2015									X

	A	B	C	D	E	F	G	H	I	J
260	Brennan1995					1				
261	Coulehan2011									X
262	Ducharme2011							1		X
263	Glueckauf2004					1				
264	Kelly2004									
265	Lai2013									X
266	Lewis2010					1				X
267	MarzialiGarcia2011					1				X
268	Torp2008									X
269	VanderRoest2010					1				X
270	Logsdon2016									X
271	Kim2012									X
272	Cho2009									X
273	Mossello2008							1		X
274	Droes2006									
275	Gitlin2006							1		
276	Droes2004									
277	Gaugler2003									
278	Zarit2011									X
279	Mavall2007									X
280	Higgins2005							1		
281	Menn2012							1		X
282	Mittelman2006							1		
283	Phung2013							1		X
284	Gitlin2003							1		
285	Andren2008	Scand J caring						1		X
286	Andren2008	Int Psychogeriatr						1		X
287	Drentea2006							1		
288	Berger2004							1		
289	Dahlrup2011							1		X
290	Charlesworth2008							1		X
291	Graff2006							1		
292	Femia2007							1		X
293	Woods2012							1		X
294	Ulstein2007							1		X
295	Ducharme2015							1		X
296	Teri2003							1		
297	Graff2007							1		X
298	Martin-Carrasco2015							1		X
299	RodriguezSanchez2013							1		X
300	Nobili2004							1		
301	Nichols2008							1		
302	Mahoney2003							1		
303	Liddle2012							1		X
304	Joling2012							1		X
305	Gitlin2008							1		X
306	Melis2009							1		X
307	Gitlin2001							1		
308	Mittelman2004	Zarit						1		
309	Mittelman2004	Haley						1		
310	Hepburn2007							1		X
311	Gitlin2010							1		X
312	Bass1998					1				

	A	B	C	D	E	F	G	H	I	J
313	Fowler2016					1				X
314	Hattink2015					1				X
315	Hicken2017					1	X	X		X
316	Kajiyama2013					1				X
317	NunezNaveira2016					1		X		X
318	VanMierlo2015					1				X
319	Davis2015					1				X
320	Griffiths2018					1	X	X		X
321	Kaijiyama2018					1	X	X		X
322	Kwok2014					1				X
323	Lorig2013					1				X
324	Austrom2015					1				X
325	Boots2017					1	X	X		X
326	Boots2016					1		X		X
327	Dam2017					1	X	X		X
328	Hattink2016					1		X		X
329	Jajor2016					1		X		X
330	MarzialiDonahue2006					1				
331	Mckechnie2014					1				X
332	O'Connor2014					1				X
333	Pagan-Ortiz2014					1				X
334	Pot2015					1				X
335	Rentz2010					1				X
336	Schaller2015					1				X
337	Schaller2016					1		X		X
338	Weitzman2016					1		X		X
339	Wilz2017						X	X		X
340	Brijoux2016							X		X
341	Wang2012									X
342	WangChien2011									X
343	Tanner2015									X
344	Orgeta2015									X
345	Schmitter2014									X
346	Korn2009									X
347	Berwig2017						X	X		X
348	Koivisto2016							X		X
349	Chu 2011									X
350	Bruvik2013									X
351	Fortinsky2009									X
352	Gallagher-Thompson2010									X
353	Garand2014									X
354	Judge2013									X
355	Kurz2012									X
356	Logsdon2011									X
357	Spijker2013									X
358	Teri2005									
359	Williams2010									X
360	Alexopoulos2012									X
361	Bakas2009									X
362	Bakas2015									X
363	Cheng2018						X	X		X
364	Clark2003									
365	Ertel2007									X

	A	B	C	D	E	F	G	H	I	J
366	Fang2017						X	X		X
367	Glass2004									
368	Harrington2010									X
369	Hoffmann2015									X
370	Inci2016									X
371	Kendall2007									
372	Markle-Reid2011									X
373	Mitchell2009									X
374	VandenHeuvel2000									
375	VandenHeuvel2002									
376	Watkins2007									X
377	Wong2015									X
378	Au2010									X
379	Barnes2018					X	X	X		X
380	Basu2015									X
381	Butcher2016							X		X
382	Canonici2012									X
383	Charlesworth2016							X		X
384	Chen2015									X
385	Cheng2014									X
386	Cheng2017							X		X
387	Cheng2016							X		X
388	Ducharme2012									X
389	Duggleby2018					X	X	X		X
390	Farran2016							X		X
391	Gitlin 2018					X		X		X
392	Foss2007									X
393	Heydari2017						X	X		X
394	Hirano2016							X		X
395	Horvath2013									X
396	Huang2013									X
397	Kales2018					X	X	X		X
398	KlodnickaKouri2011									X
399	Kunik2017						X	X		X
400	Kuo2016							X		X
401	Kuo2013									X
402	Kuo2017						X	X		X
403	Lee2012									X
404	Li2014									X
405	Lok2017						X	X		X
406	Lord2017						X	X		X
407	Mackenzie2007									X
408	Mahdavi2017						X	X		X
409	Moniz-Cook2008									X
410	Orrell2017						X	X		X
411	Perren2006									X
412	Pankong2018					X	X	X		X
413	Prick2015									X
414	Regan2017						X	X		X
415	Salamizadeh2017						X	X		X
416	Salva2011									X
417	Sarkamo2014									X
418	Schoenmakers2010									X

	A	B	C	D	E	F	G	H	I	J
419	Sepe-Monti2016							X		X
420	Shata2017						X	X		X
421	Spector2015									X
422	Stirling2012									X
423	Thyrian2017						X	X		X
424	Tremont2015									X
425	VanMierlo2012									X
426	Villareal-Reyna2012									X
427	Voigt-Radloff2011									X
428	Waldorff2012									X
429	Wesson2013									X
430	Woods2016							X		X
431	Xiao2016							X		X
432	Zwingmann2017						X	X		X
433	Cameron2015									X
434	Clarke2014									X
435	Franzen-Dahlin2008									X
436	Grasel2005									
437	Lindley2017						X	X		X
438	Pandian2015									X
439	Mant2005									
440	Martinez-Velilla2016							X		X
441	Oupra2010									X
442	Vandenberg2016							X		X
443	Shyu2008									X
444	Boltong2017						X	X		X
445	Boudioni1999									
446	Boudioni2004									
447	Bright2005									
448	Browman1995									
449	Byrne2012									X
450	Chambers2012									X
451	Darrow1998									
452	Davis1998									
453	Dean2007									
454	Ekberg2014									X
455	Fennell2016							X		X
456	Freimuth1993									
457	Hawkes2010									X
458	Heckel2016							X		X
459	Heidrich2003									
460	Hughes2011									X
461	Jefford2005									
462	Klikovac2015									X
463	Lechner1996									
464	Linehan2017						X	X		X
465	Maibach1998									
466	Manning2002									
467	Marcus1993									
468	Marcus2002									
469	Meissner1990									
470	Morris2015									X
471	Namboodiri1993									

	A	B	C	D	E	F	G	H	I	J
472	Reiches1982									
473	Reubsaet2006									
474	Rimer1993									
475	Finney2007									X
476	Salako2016							X		X
477	Slevin1988									
478	Sanders2008									X
479	Vanderpool2011									X
480	Venn1996									
481	Dewing1998									
482	Heckel2018						X	X		X
483	Sutherland2005									
484	Williams2019					X	X	X		X
485	Possin2019					X	X	X		X
486	Metcalfe2019					X	X	X		X
487	Meichsner2019					X	X	X		X
488	Gustafson2019					X	X	X		X
489	Elliott2010									X
490	Mittelman2007									X
491	Au2019					X	X	X		X
492	Hayden2012									X
493	HuisinhetVeld2020		X	X	X	X	X	X	X	X
494	Brodaty1997									
495	Burch1999									
496	Burdz1988									
497	Conlin1992									
498	Milne1993									
499	Richards2003									
500	Riordan1998									
501	Rolleston1994									
502	Rothman1993									
503	Schwarz1997									
504	Leitsch2001									
505	Adler1993									
506	Chi1994									
507	Cox1998									
508	Deimling1992									
509	Desroisers2004									
510	Gilleard1987									
511	Gilleard1984									
512	Harper1993									
513	Homer1994									
514	Hoskins2005									
515	Theis1994									
516	Tremont2013									X
517	Duncan2011									X
518	Candell2003									
519	Cernvall2015									X
520	Grover2011									X
521	Hoyle2013									X
522	Klemm2014									X
523	Petranovich2015									X
524	Rotonti2005									

	A	B	C	D	E	F	G	H	I	J
525	Swallow2015									X
526	Wade2012									X
527	Antonini2012									X
528	Barbabella2016							X		X
529	Berk2013									X
530	Binford2013									X
531	Blusi2014									X
532	Chiu2009									X
533	Clifford2013									X
534	Dew2004									
535	Fidika2015									X
536	Lorig2012									X
537	Lucas2011									X
538	Sander2009									X
539	Scott2013									X
540	Song2015									X
541	Zimmerman2014									X
542	Paller2015									X
543	Birnie2010									X
544	Cash2016						X			X
545	Hankin2010									X
546	Lengacher2012									X
547	Dooley2004									
548	Gerner2013									X
549	Clare2010									
550	O'connor2019					X	X	X		X
551	Winter2006									
552	Baucom2009									X
553	Blanchard1996									
554	Budin2008									X
555	Bultz2000									
556	Couper2015									X
557	Heinrich1985									
558	Kozachik2001									
559	Kuijer2004									
560	Kurtz2005									
561	Manne2004									
562	Manne2005									
563	Manne2011									X
564	McCorkle2007									X
565	Nicolaisen2018						X	X		X
566	Tsianakas2015									X
567	Bierlein2006									
568	Bird2007									X
569	Brodaty2009									X
570	Brown2012									X
571	Burns2003									
572	Cartmell2011									X
573	Chodosh2012									X
574	Clare2005									
575	Clark2004									
576	Duru2009									X
577	Edgecombe2009									X

	A	B	C	D	E	F	G	H	I	J
578	Fischer2003									
579	Gaugler2008									X
580	Iliffe2014									X
581	King2011									X
582	LaFontaine2011									X
583	Marek2006									
584	McAiney2010									X
585	Moore2011									X
586	Onder2008									X
587	Onder2007									X
588	Specht2009									X
589	Stevenson2006									
590	Tam-Tham2013									X
591	VandenBosch2009									X
592	Weaver2008									X
593	Woods2003									
594	Nichols2011									X
595	Fortinsky2014									X
596	Andren2008									X
597	Bartfay2013									X
598	Bass2013									X
599	Carbonneau2011									X
600	Corbett2012									X
601	Donofrio2015									X
602	Egan2010									X
603	Eggenberger2013									X
604	Elvish2013									X
605	Farran2008									X
606	Gaugler2011									X
607	Gitlin2010	Gerontechnology								X
608	Gitlin2010	JAMA								X
609	Kouri2011									X
610	Marim2013									X
611	Ponce2011									X
612	Rowe2010									X
613	Soylemez2016							X		X
614	Tang2018					X	X	X		X
615	Lowery2014									X
616	Gaugler2016							X		X
617	Geschke2012									X
618	Meichsner2018					X	X	X		X
619	Wilz2018					X	X	X		X
620										
621										
622										
623										
624										
625										
626										
627										
628										
629										
630										

	A	B	C	D	E	F	G	H	I	J
631										
632										
633										
634										
635										
636										
637										
638										
639										
640										
641										
642										
643										
644										
645										
646										
647										
648										
649										
650										
651										
652										
653										
654										
655										
656										
657										
658										
659										
660										
661										
662										
663										
664										
665										
666										
667										
668										
669										
670										
671										
672										
673										
674										
675										
676										
677										
678										
679										
680										
681										
682										
683										

ew only

	A	B	C	D	E	F	G	H	I	J
684										
685										
686										
687										
688										
689										
690										
691										
692										
693										
694										
695										
696										
697										
698										
699										
700										
701										
702										
703										
704										
705										
706										
707										
708										
709										
710										
711										
712										
713										
714										
715										
716										
717										
718										
719										
720										
721										
722										
723										
724										
725										
726										
727										
728										
729										
730										
731										
732										
733										
734										
735										
736										

For peer review only - http://bmjopen.bmj.com/site/about/guidelines.xhtml

	A	B	C	D	E	F	G	H	I	J
737										
738										
739										
740										
741										
742										
743										
744										
745										
746										
747										
748										
749										
750										
751										
752										
753										
754										
755										
756										
757										
758										
759										
760										
761										
762										
763										
764										
765										
766										
767										
768										
769										
770										
771										
772										
773										
774										
775										
776										
777										
778										
779										
780										
781										
782										
783										
784										
785										
786										
787										
788										
789										

	A	B	C	D	E	F	G	H	I	J
790										
791										
792										
793										
794										
795										
796										
797										
798										
799										
800										
801										
802										
803										
804										
805										
806										
807										
808										
809										
810										
811										
812										
813										
814										
815										
816										
817										
818										
819										
820										
821										
822										
823										
824										
825										
826										
827										
828										
829										
830										
831										
832										
833										
834										
835										
836										
837										
838										
839										
840										
841										
842										

For peer review only - http://bmjopen.bmj.com/site/about/guidelines.xhtml

	A	B	C	D	E	F	G	H	I	J
843										
844										
845										
846										
847										
848										
849										
850										
851										
852										
853										
854										
855										
856										
857										
858										
859										
860										
861										
862										
863										
864										
865										
866										
867										
868										
869										
870										
871										
872										
873										
874										
875										
876										
877										
878										
879										
880										
881										
882										
883										
884										
885										
886										
887										
888										
889										
890										
891										
892										
893										
894										
895										

	A	B	C	D	E	F	G	H	I	J
896										
897										
898										
899										
900										
901										
902										
903										
904										
905										
906										
907										
908										
909										
910										
911										
912										
913										
914										
915										
916										
917										
918										
919										
920										
921										
922										
923										
924										
925										
926										
927										
928										
929										
930										
931										
932										
933										
934										
935										
936										
937										
938										
939										
940										
941										
942										
943										
944										
945										
946										
947										
948										

ew only

	A	B	C	D	E	F	G	H	I	J
949										
950										
951										
952										
953										
954										
955										
956										
957										
958										
959										
960										
961										
962										
963										
964										
965										
966										
967										
968										
969										
970										
971										
972										
973										
974										
975										
976										
977										
978										
979										
980										
981										
982										
983										
984										
985										
986										
987										
988										
989										
990										
991										
992										
993										
994										
995										
996										
997										
998										
999										
1000										
1001										

For peer review only - http://bmjopen.bmj.com/site/about/guidelines.xhtml

	A	B	C	D	E	F	G	H	I	J
1002										
1003										
1004										
1005										
1008										

For peer review only

	K	L	M	N	O	P	Q	R	S	T	U	V	W	X	Y	Z	AA	AB	AC
1																			
2																			
3																			
4																			
5	**5257 Wiegelmanı**	**3552 Akarsu 2019**	**3734 Fu 2017**	**3939 Jensen 2015**	**3965 Corvol 2017**	**3970 Hopkinson 2**	**4267 Pucciarelli 2**	**4415 Vernooij-Da**	**4452 Reilly 2015**	**4530 Liu 2018**	**4531 Lins 2014**	**4534 Legg 2011**	**4567 Forster 2012**	**4588 Corry 2019**	**5731 Piersol 2017**	**2104 Cuthbert 20**	**3453 Collins 2019**	**615 Waldron 201**	**3544 Boots 2014**
6								X				X	X						
7		X	X	X	X	X		X	X	X	X	X	X	X	X	X	X	X	X
8			X	X	X			X	X	X	X	X	X		X	X		X	X
9								X				X							
10								X				X	X						
11																			
12				X				X		X	X	X	X		X			X	X
13								X				X	X						
14			1					X				X	X						
15								X				X	X						
16																			
17			1					X				X	X						
18			1					X				X	X						
19																			
20			1					X				X							
21								X				X	X						
22			1					X				X	X						
23			1	X				X	X		X	X	X	1				X	X
24				X				X	X		X	X	X					X	X
25			X	X	X			X	X	X	X	X	X		X	X		X	X
26								X	X	1	X						1	X	X
27										1					1		1		
28								X				X	X					X	
29	1		X	X	X			X	X		X	X	X		X	X		X	X
30								X				X	X						
31				X				X	X		X	X	X					X	X
32			X	X	X			X	X		X	X	X		X	X		X	X
33								X			X	X	X					X	X
34								X				X		1	1				
35																			
36				X				X	X	X		X	X						
37																			
38				X				X	X	X		X	X						
39																			
40								1											1
41	1			X				X				X	X						
42								X				X	X						
43				X				X	X		X	X	X					X	X
44			1					X			X	X	X					X	X
45							1	X				X	X						
46								X				X	X						
47												X	X					X	

For peer review only

	K	L	M	N	O	P	Q	R	S	T	U	V	W	X	Y	Z	AA	AB	AC
48												X	X						
49																			
50							1	X				X	X						
51								X			X	X	X					X	X
52			1					X				X	X						
53			1					X				X	X						
54			1					X				X							
55			1					X				X	X						
56			1					X				X	X						
57			1					X				X	X						
58			1					X				X	X					X	
59			1					X				X	X					X	
60			1					X				X	X					X	
61			1					X			X	X	X					X	X
62			1	X				X	X		X	X	X					X	X
63			1	X				X	X		X	X	X					X	X
64			1	X				X	X		X	X	X					X	X
65			1	X				X	X		X	X	X					X	X
66			1	X				X	X		X	X	X					X	X
67		1																	
68		1																	
69		1		X				X	X		X	X	X					X	X
70	1	1						X				X	X					X	
71		1																	
72		1				1													
73		1				1		1						1					
74		1				1													
75		1		X				X	X		X	X	X					X	X
76		1						X				X	X	1	1				
77		1						X			X	X	X					X	X
78		1									1			1					
79								X			X							X	X
80	1			X				X	X		X	X	X					X	X
81						1		1											
82																			
83																			
84						1		X	X			X							
85	1							X				X	X		1			X	
86				X				X	X		X	X	X					X	X
87	1			X				X	X		X	X	X					X	X
88				X				X	X		X	X	X	1				X	X
89																			
90																			
91																			
92																			
93																			
94						1													
95																			
96																			
97																			
98																			
99																			
100																			

For peer review only

	K	L	M	N	O	P	Q	R	S	T	U	V	W	X	Y	Z	AA	AB	AC
101																			
102																			
103																			
104																			
105																			
106																			
107																			
108																			
109																			
110																			
111	1			1															
112	1			1															
113	1			1															
114				1															
115	1			1															
116	1			1															
117				1															
118					1				1										
119					1														
120					1														
121					1														
122					1				1										
123					1				1										
124					1				1						1				
125					1														
126					1														
127					1														
128					1				1						1				
129					1			X	1			X			1				
130					1			X	1			X			1				
131					1			X				X	X						
132					1			X				X	X						
133					1			X			X	X	X					X	X
134						1		1											
135						1		X			X	X	X					X	X
136						1													
137						1													
138						1		X			X	X	X					X	X
139						1													
140				X		1		X	X		X	X	X					X	X
141						1		X			X	X	X					X	X
142						1		1											
143	1			X		1		X	X		X	X	X					X	X
144	1					1		X				X	X		1			X	
145	1					1		X			X	X	X					X	X
146						1													
147	1			X		1		X	X		X	X	X				1	X	X
148						1													
149						1		1											
150						1		X			X	X	X					X	X
151				X		1		X	X		X	X	X	1				X	X
152				X		1		X	X		X	X	X			X		X	X
153							1												

For peer review only

	K	L	M	N	O	P	Q	R	S	T	U	V	W	X	Y	Z	AA	AB	AC
154							1												
155							1												
156							1												
157							1	X				X							
158							1	X				X	X					X	
159							1	X			X	X	X					X	X
160							1	X			X	X	X					X	X
161				X			1	X	X		X	X	X					X	X
162				X			1	X	X		X	X	X					X	X
163				X			1	X	X		X	X	X			X		X	X
164							1	X				X	X					X	
165							1	X			X	X	X	1				X	X
166							1	X			X	X	X					X	X
167								1			1								
168								1											
169								1											
170								1											
171								1											
172									1										
173									1										
174									1										
175									1										
176									1										
177									1										
178								X		1	X	X	X					X	X
179								X		1		X	X				1	X	
180								X		1		X	X		1		1	X	
181											1								
182											1								
183											1								
184											1								
185											1								
186				X				X	X		1	X	X					X	
187											1								
188											1			1					
189								X			1	X							
190												1	1						
191												1							
192												1							
193												1	1						
194												1	1						
195												1							
196												1							
197												1							
198													1						
199													1						
200													1						1
201													1						
202													1						
203													1						
204													1						
205													1						
206													1						

For peer review only

	K	L	M	N	O	P	Q	R	S	T	U	V	W	X	Y	Z	AA	AB	AC
207													1						
208													1						
209													1						
210													1						
211													1						
212													1						
213													1						
214													1						
215													1						
216	1													1	1	1			
217				X				X				X	X	1				X	
218	1							X				X	X	1				X	
219								X				X	X	1				X	
220														1					
221								X				X	X	1				X	
222				X				X				X	X	1				X	
223								X				X	X	1				X	
224								X				X	X	1				X	
225														1					
226				X				X	X		X	X	X	1				X	X
227								X				X	X	1					
228				X				X			X	X	X			X		X	X
229				X				X			X	X	X			X		X	X
230																1			
231																1			
232								X				X				1		X	
233								X				X				1			
234																1			
235																1			
236								X				X				1		X	
237																1			
238																		1	
239																		1	
240																		1	
241																		1	
242																		1	
243																		1	
244				X				X	X		X	X	X				1	X	X
245								X				X					1		
246								X				X					1		
247																	1		
248								X				X	X		1		1		
249								X			X	X	X				1	X	X
250				X				X	X			X	X				1	X	
251								X											
252				X				X	X		X	X	X					X	X
253				X				X	X		X	X	X					X	X
254				X				X	X		X	X	X					X	X
255				X				X	X		X	X	X					X	X
256				X				X	X		X	X	X					X	X
257								X				X	X						
258				X				X	X		X	X	X					X	X
259				X				X	X		X	X	X					X	X

For peer review only

	K	L	M	N	O	P	Q	R	S	T	U	V	W	X	Y	Z	AA	AB	AC
260																			1
261								X				X							1
262								X				X			1				1
263																			1
264																			1
265								X				X	X					X	1
266																			1
267								X				X			1				1
268																			1
269																			1
270								X	X			X	X					X	
271								X				X	X						
272																			
273																			
274																			
275																			
276																			
277																			
278								X				X							
279																			
280																			
281								X				X							
282																			
283								X				X			1			X	
284																			
285															1				
286															1				
287																			
288																			
289								X				X							
290																			
291																			
292																			
293								X				X	X						
294																			
295				X				X	X		X	X	X					X	X
296																			
297																			
298	1			X				X	X		X	X	X					X	X
299								X				X	X					X	
300																			
301																			
302																			
303								X				X	X						
304	1							X				X	X		1				
305																			
306																			
307																			
308																			
309																			
310																			
311	1														1				
312																			

For peer review only

	K	L	M	N	O	P	Q	R	S	T	U	V	W	X	Y	Z	AA	AB	AC
313				X				X	X		X	X	X					X	X
314				X				X	X		X	X	X					X	X
315			X	X	X			X	X		X	X	X		X			X	X
316								X				X	X		1			X	
317				X				X	X		X	X	X					X	X
318				X				X	X		X	X	X					X	X
319				X				X	X		X	X	X					X	X
320			X	X	X			X	X	X	X	X	X		X			X	X
321			X	X	X			X	X	X	X	X	X		X			X	X
322								X				X	X					X	X
323								X				X	X					X	
324				X				X	X		X	X	X					X	X
325			X	X	X			X	X		X	X	X		X			X	X
326				X				X	X		X	X	X					X	X
327			X	X	X			X	X		X	X	X		X			X	X
328				X				X	X		X	X	X					X	X
329				X				X	X		X	X	X					X	X
330																			
331								X			X	X	X					X	X
332								X			X	X	X					X	X
333								X			X	X	X					X	X
334				X				X	X		X	X	X					X	X
335																			
336				X				X	X		X	X	X					X	X
337				X				X	X		X	X	X					X	X
338				X				X	X		X	X	X					X	X
339			X	X	X			X	X		X	X	X		X	X		X	X
340	1			X				X	X		X	X	X					X	X
341								X				X	X		1				
342								X				X							
343				X				X	X		X	X	X					X	X
344				X				X	X		X	X	X					X	X
345								X			X	X	X					X	X
346																			
347	1		X	X	X			X	X		X	X	X		X	X		X	X
348				X				X	X		X	X	X					X	X
349								X				X							
350								X				X	X					X	
351	1																		
352															1				
353								X			X	X	X					X	X
354	1							X				X	X					X	
355								X				X							
356								X				X							
357								X				X	X					X	
358																			
359																			
360								X				X	X						
361																			
362				X				X	X		X	X	X					X	X
363			X	X	X			X	X		X	X	X		X	X		X	X
364																			
365																			

For peer review only

	K	L	M	N	O	P	Q	R	S	T	U	V	W	X	Y	Z	AA	AB	AC
366			X	X	X			X	X		X	X	X		X	X		X	X
367																			
368																			
369				X				X	X		X	X	X					X	X
370				X				X	X		X	X	X					X	X
371																			
372								X				X							
373																			
374																			
375																			
376																			
377				X				X	X		X	X	X					X	X
378																			
379			X	X	X			X	X	X	X	X	X		X	X		X	X
380				X				X	X		X	X	X					X	X
381				X				X	X		X	X	X					X	X
382								X				X	X						
383	1			X				X	X		X	X	X					X	X
384	1			X				X	X		X	X	X					X	X
385								X			X	X	X					X	X
386	1		X	X	X			X	X		X	X	X		X	X		X	X
387	1			X				X	X		X	X	X					X	X
388								X				X	X						
389			X	X	X			X	X	X	X	X	X		X	X		X	X
390				X				X	X		X	X	X					X	X
391	1		X	X	X			X	X	X	X	X	X		X	X		X	X
392																			
393			X	X	X			X	X		X	X	X		X	X		X	X
394	1			X				X	X		X	X	X					X	X
395								X				X	X					X	
396								X				X	X					X	
397			X	X	X			X	X	X	X	X	X		X	X		X	X
398								X				X							
399	1		X	X	X			X	X		X	X	X		X	X		X	X
400				X				X	X		X	X	X					X	X
401								X				X	X		1			X	
402	1		X	X	X			X	X		X	X	X		X	X		X	X
403								X				X	X		1				
404								X			X	X	X					X	X
405			X	X	X			X	X		X	X	X		X	X		X	X
406			X	X	X			X	X		X	X	X		X	X		X	X
407																			
408	1		X	X	X			X	X		X	X	X		X	X		X	X
409																			
410			X	X	X			X	X		X	X	X		X	X		X	X
411																			
412			X	X	X			X	X	X	X	X	X		X	X		X	X
413	1			X				X	X		X	X	X					X	X
414			X	X	X			X	X		X	X	X		X	X		X	X
415			X	X	X			X	X		X	X	X		X	X		X	X
416								X				X							
417								X			X	X	X					X	X
418																			

For peer review only

	K	L	M	N	O	P	Q	R	S	T	U	V	W	X	Y	Z	AA	AB	AC
419				X				X	X		X	X	X					X	X
420			X	X	X			X	X		X	X	X		X	X		X	X
421				X				X	X		X	X	X					X	X
422								X				X	X						
423			X	X	X			X	X		X	X	X		X	X		X	X
424	1			X				X	X		X	X	X					X	X
425								X				X	X						
426								X				X	X						
427								X				X							
428								X				X	X						
429								X				X	X					X	
430	1			X				X	X		X	X	X					X	X
431				X				X	X		X	X	X					X	X
432			X	X	X			X	X		X	X	X		X	X		X	X
433				X				X	X		X	X	X					X	X
434								X			X	X	X					X	X
435																			
436																			
437			X	X	X			X	X		X	X	X		X	X		X	X
438				X				X	X		X	X	X					X	X
439																			
440				X				X	X		X	X	X					X	X
441																			
442				X				X	X		X	X	X					X	X
443																			
444			X	X	X			X	X		X	X	X		X	X		X	X
445																			
446																			
447																			
448																			
449								X				X	X						
450								X				X	X						
451																			
452																			
453																			
454								X			X	X	X					X	X
455				X				X	X		X	X	X					X	X
456																			
457																			
458				X				X	X		X	X	X					X	X
459																			
460								X				X							
461																			
462				X				X	X		X	X	X					X	X
463																			
464			X	X	X			X	X		X	X	X		X	X		X	X
465																			
466																			
467																			
468																			
469																			
470				X					X		X	X	X					X	X
471																			

	K	L	M	N	O	P	Q	R	S	T	U	V	W	X	Y	Z	AA	AB	AC
472																			
473																			
474																			
475																			
476				X					X		X	X	X					X	X
477																			
478																			
479								X				X							
480																			
481																			
482			X	X	X				X	X	X	X	X		X	X		X	X
483																			
484		X	X	X	X	X		X	X	X	X	X	X	X	X	X	X	X	X
485		X	X	X	X	X		X	X	X	X	X	X	X	X	X	X	X	X
486		X	X	X	X	X		X	X	X	X	X	X	X	X	X	X	X	X
487		X	X	X	X	X		X	X	X	X	X	X	X	X	X	X	X	X
488		X	X	X	X	X		X	X	X	X	X	X	X	X	X	X	X	X
489															1				
490																			
491		X	X	X	X	X		X	X	X	X	X	X	X	X	X	X	X	X
492								X				X	X						
493		X	X	X	X	X	X	X	X	X	X	X	X	X	X	X	X	X	X
494																			
495																			
496																			
497																			
498																			
499																			
500																			
501																			
502																			
503																			
504																			
505																			
506																			
507																			
508																			
509																			
510																			
511																			
512																			
513																			
514																			
515																			
516								X				X						X	
517								X				X							
518																			
519				X				X		X	X	X	X					X	X
520								X				X							
521								X				X	X					X	
522								X			X	X	X					X	X
523				X				X		X	X	X	X					X	X
524																			

For peer review only

	K	L	M	N	O	P	Q	R	S	T	U	V	W	X	Y	Z	AA	AB	AC
525				X				X		X	X	X	X					X	X
526								X				X	X						
527								X				X	X						
528				X				X	X		X	X	X					X	X
529								X				X	X					X	
530								X				X	X					X	
531								X			X	X	X					X	X
532																			
533								X				X	X					X	
534																			
535				X				X	X		X	X	X					X	X
536								X				X	X						
537								X				X							
538																			
539								X				X	X					X	
540				X				X	X		X	X	X					X	X
541								X			X	X	X					X	X
542				X				X	X		X	X	X					X	X
543																			
544				X				X	X		X	X	X					X	X
545																			
546								X				X	X						
547																			
548								X				X	X					X	
549																			
550		X	X	X	X	X		X	X	X	X	X	X	X	X	X	X	X	X
551																			
552																			
553																			
554																			
555																			
556				X				X	X		X	X	X					X	X
557																			
558																			
559																			
560																			
561																			
562																			
563								X				X							
564																			
565			X	X	X			X		X	X	X	X		X			X	X
566				X				X	X		X	X	X					X	X
567																			
568																			
569																			
570								X				X	X						
571																			
572								X				X							
573								X				X	X						
574																			
575																			
576																			
577																			

For peer review only

	K	L	M	N	O	P	Q	R	S	T	U	V	W	X	Y	Z	AA	AB	AC
578																			
579																			
580								X			X	X	X					X	X
581								X				X							
582								X				X							
583																			
584																			
585								X				X							
586																			
587																			
588																			
589																			
590								X				X	X					X	
591																			
592																			
593																			
594								X											
595								X				X	X					X	
596															1				
597								X				X	X		1			X	
598								X				X	X		1			X	
599								X				X			1				
600								X				X	X		1				
601				X				X	X			X	X		1			X	X
602															1				
603								X				X	X		1			X	
604								X				X	X		1			X	
605															1				
606								X				X			1				
607															1				
608	1														1				
609								X				X			1				
610								X				X	X		1			X	
611								X				X			1				
612															1				
613	1			X				X	X		X	X	X					X	X
614	1		X	X	X			X	X	X	X	X	X		X	X		X	X
615	1							X			X	X	X					X	X
616	1			X				X	X		X	X	X					X	X
617	1							X				X	X						
618	1		X	X	X			X	X	X	X	X	X		X	X		X	X
619	1		X	X	X			X	X	X	X	X	X		X	X		X	X
620																			
621																			
622																			
623																			
624																			
625																			
626																			
627																			
628																			
629																			
630																			

For peer review only

	K	L	M	N	O	P	Q	R	S	T	U	V	W	X	Y	Z	AA	AB	AC
631																			
632																			
633																			
634																			
635																			
636																			
637																			
638																			
639																			
640																			
641																			
642																			
643																			
644																			
645																			
646																			
647																			
648																			
649																			
650																			
651																			
652																			
653																			
654																			
655																			
656																			
657																			
658																			
659																			
660																			
661																			
662																			
663																			
664																			
665																			
666																			
667																			
668																			
669																			
670																			
671																			
672																			
673																			
674																			
675																			
676																			
677																			
678																			
679																			
680																			
681																			
682																			
683																			

For peer review only

	K	L	M	N	O	P	Q	R	S	T	U	V	W	X	Y	Z	AA	AB	AC
684																			
685																			
686																			
687																			
688																			
689																			
690																			
691																			
692																			
693																			
694																			
695																			
696																			
697																			
698																			
699																			
700																			
701																			
702																			
703																			
704																			
705																			
706																			
707																			
708																			
709																			
710																			
711																			
712																			
713																			
714																			
715																			
716																			
717																			
718																			
719																			
720																			
721																			
722																			
723																			
724																			
725																			
726																			
727																			
728																			
729																			
730																			
731																			
732																			
733																			
734																			
735																			
736																			

For peer review only

	K	L	M	N	O	P	Q	R	S	T	U	V	W	X	Y	Z	AA	AB	AC
737																			
738																			
739																			
740																			
741																			
742																			
743																			
744																			
745																			
746																			
747																			
748																			
749																			
750																			
751																			
752																			
753																			
754																			
755																			
756																			
757																			
758																			
759																			
760																			
761																			
762																			
763																			
764																			
765																			
766																			
767																			
768																			
769																			
770																			
771																			
772																			
773																			
774																			
775																			
776																			
777																			
778																			
779																			
780																			
781																			
782																			
783																			
784																			
785																			
786																			
787																			
788																			
789																			

For peer review only

	K	L	M	N	O	P	Q	R	S	T	U	V	W	X	Y	Z	AA	AB	AC
790																			
791																			
792																			
793																			
794																			
795																			
796																			
797																			
798																			
799																			
800																			
801																			
802																			
803																			
804																			
805																			
806																			
807																			
808																			
809																			
810																			
811																			
812																			
813																			
814																			
815																			
816																			
817																			
818																			
819																			
820																			
821																			
822																			
823																			
824																			
825																			
826																			
827																			
828																			
829																			
830																			
831																			
832																			
833																			
834																			
835																			
836																			
837																			
838																			
839																			
840																			
841																			
842																			

For peer review only

	K	L	M	N	O	P	Q	R	S	T	U	V	W	X	Y	Z	AA	AB	AC
843																			
844																			
845																			
846																			
847																			
848																			
849																			
850																			
851																			
852																			
853																			
854																			
855																			
856																			
857																			
858																			
859																			
860																			
861																			
862																			
863																			
864																			
865																			
866																			
867																			
868																			
869																			
870																			
871																			
872																			
873																			
874																			
875																			
876																			
877																			
878																			
879																			
880																			
881																			
882																			
883																			
884																			
885																			
886																			
887																			
888																			
889																			
890																			
891																			
892																			
893																			
894																			
895																			

For peer review only

	K	L	M	N	O	P	Q	R	S	T	U	V	W	X	Y	Z	AA	AB	AC
896																			
897																			
898																			
899																			
900																			
901																			
902																			
903																			
904																			
905																			
906																			
907																			
908																			
909																			
910																			
911																			
912																			
913																			
914																			
915																			
916																			
917																			
918																			
919																			
920																			
921																			
922																			
923																			
924																			
925																			
926																			
927																			
928																			
929																			
930																			
931																			
932																			
933																			
934																			
935																			
936																			
937																			
938																			
939																			
940																			
941																			
942																			
943																			
944																			
945																			
946																			
947																			
948																			

For peer review only

	K	L	M	N	O	P	Q	R	S	T	U	V	W	X	Y	Z	AA	AB	AC
949																			
950																			
951																			
952																			
953																			
954																			
955																			
956																			
957																			
958																			
959																			
960																			
961																			
962																			
963																			
964																			
965																			
966																			
967																			
968																			
969																			
970																			
971																			
972																			
973																			
974																			
975																			
976																			
977																			
978																			
979																			
980																			
981																			
982																			
983																			
984																			
985																			
986																			
987																			
988																			
989																			
990																			
991																			
992																			
993																			
994																			
995																			
996																			
997																			
998																			
999																			
1000																			
1001																			

For peer review only

	K	L	M	N	O	P	Q	R	S	T	U	V	W	X	Y	Z	AA	AB	AC
1002																			
1003																			
1004																			
1005																			
1008	[redacted]																		

For peer review only

	AD	AE	AF	AG	AH	AI	AJ	AK	AL	AM	AN	AO	AP	AQ	AR	AS	AT	AU	AV
1																			
2																			
3																			
4																			
5	3984 Doyle 2020	4285 Maffioletti	2570 Lee 2020	2809 Weinbrecht	3014 Wang 2020	152 Minshall 201	588 Cheng 2020	1611 Smith 2019	248 Heckel 2019	1596 Egan 2018	1977 Zhao 2019	1992 Leng 2020	XT Maayan 2014	3195 Shaw 2009	2520 Teahan 202	4562 GonzalezFra	3528 Amador-Ma	5049 Sin 2018	4806 Parkinson 2
6														X					
7				X		X		X	X	X	X		X	X			X	X	X
8				X						X			X	X			X	X	
9														X					
10														X					
11														X					
12										X			X	X				X	
13														X					
14														X					
15														X					
16														X					
17														X					
18														X					
19														X					
20														X					
21														X				1	
22														X					
23				X									X	X					
24				X									X	X					
25				X						X			X	X			X	X	
26				X			1						X	X					
27							1							X					
28														X					
29			1	X									X	X			X		
30							1							X					
31							1						X	X					
32				X			1						X	X			X		
33							1						X	X		1			
34														X					
35																			
36			1				1							X					
37																			
38														X					
39																			
40				1	1					1	1	1							
41					1		1			1	1	1		X		1		1	
42														X					
43					1		1			1	1	1	X	X		1		1	
44													X	X				1	
45								1						X					
46														X					
47														X				1	

	AD	AE	AF	AG	AH	AI	AJ	AK	AL	AM	AN	AO	AP	AQ	AR	AS	AT	AU	AV
48														X					
49								1						X				1	
50						1								X				1	
51			1	1			1				1	1	X	X		1		1	
52														X					
53														X					
54														X					
55														X					
56														X					
57														X					
58														X					
59														X					
60														X					
61													X	X					
62													X	X					
63													X	X					
64													X	X					
65													X	X					
66				X									X	X					
67				1			1												
68					1														
69							1						X	X					
70				1			1							X		1			
71																		1	
72																			
73							1												
74				1			1												
75							1						X	X					
76														X					
77													X	X					
78				1			1									1			
79													X	X					
80							1						X	X		1			
81																			
82																			
83							1												
84				1			1							X					
85							1							X	1				
86				X									X	X					
87				X	1		1						X	X					
88				X									X	X					
89																			
90														1					
91																			
92																			
93																			
94														1					
95		1												1					
96		1												1					
97																			
98														1					
99														1					
100													1	1					

	AD	AE	AF	AG	AH	AI	AJ	AK	AL	AM	AN	AO	AP	AQ	AR	AS	AT	AU	AV
101													1	1					
102														1					
103																			
104																			
105													1						
106																			
107																			
108														1					
109																			
110																			
111				1			1							X					
112			1				1							X	1				
113			1				1							X	1				
114																			
115				1			1							X			1		
116				1			1							X	1				
117							1							X					
118																			
119																			
120																			
121																			
122			1				1												
123				1			1												
124							1												
125																			
126														X					
127														X					
128				1										X					
129			1				1							X	1		1		
130			1	1			1							X					
131														X					
132														X					
133													X	X					
134																			
135													X	X					
136																			
137																			
138													X	X					
139																			
140				X			1						X	X					
141							1						X	X					
142																			
143							1						X	X					
144			1	1			1							X	1		1		
145							1						X	X			1		
146																			
147							1						X	X					
148																			
149							1												
150													X	X					
151				X									X	X					
152				X									X	X					
153														X					

For peer review only

	AD	AE	AF	AG	AH	AI	AJ	AK	AL	AM	AN	AO	AP	AQ	AR	AS	AT	AU	AV
154														X					
155								1						X					
156						1		1						X					
157								1						X					
158								1						X					
159													X	X					
160						1		1					X	X					
161						1							X	X					
162								1					X	X					
163				X									X	X					
164														X					
165						1		1					X	X					
166													X	X					
167																			
168																			
169																			
170																			
171																			
172																			
173																			
174				1			1								1				
175																			
176														X					
177																			
178													X	X					
179														X					
180							1							X	1				
181																			
182									1							1			
183							1											1	
184							1									1			
185							1												
186														X					
187					1														
188							1									1			
189							1							X					
190						1													
191						1		1											
192						1													
193								1											
194						1		1											
195								1											
196																			
197																			
198																			
199																			
200																			
201																			
202																			
203																			
204																			
205																			
206																			

For peer review only

	AD	AE	AF	AG	AH	AI	AJ	AK	AL	AM	AN	AO	AP	AQ	AR	AS	AT	AU	AV
207						1													
208																			
209																			
210																			
211																			
212																			
213																			
214						1													
215								1											
216							1												
217														X					
218		1					1							X	1	1			
219							1							X		1			
220																			
221														X					
222														X					
223														X					
224														X					
225																			
226				X				1					X	X					
227														X					
228													X	X					
229													X	X				X	
230	1																		
231																			
232							1							X					
233							1							X					
234																			
235																			
236	1													X					
237																			
238																			
239																			
240																			
241																			
242																			
243																			
244				X									X	X					
245														X					
246														X					
247														X					
248														X					
249													X	X					
250														X					
251	1													X					
252	1												X	X					
253	1			X									X	X				X	
254	1			X									X	X					
255	1												X	X					
256	1			X									X	X				X	
257	1													X					
258	1												X	X					
259	1												X	X					

For peer review only

	AD	AE	AF	AG	AH	AI	AJ	AK	AL	AM	AN	AO	AP	AQ	AR	AS	AT	AU	AV
260										1		1				1			
261														X					
262							1							X					
263																		1	
264																			
265										1				X				1	
266														X					
267														X				1	
268																			
269																			
270		1		X										X					
271		1												X					
272		1												X					
273		1																	
274		1																	
275		1																	
276		1																	
277		1												1					
278		1												X					
279		1																	
280		1												1					
281														X					
282																			
283			1	1										X			1		
284																			
285																			
286																			
287																			
288																			
289														X					
290			1	1			1												
291			1	1			1												
292																			
293				1			1							X					
294							1												
295													X	X					
296																			
297							1								1				
298			1	1			1						X	X	1		1		
299			1											X					
300																			
301																			
302																1		1	
303							1							X					
304			1	1			1							X	1				
305							1												
306														X					
307																			
308																			
309																			
310																			
311							1							X	1				
312																			

For peer review only

	AD	AE	AF	AG	AH	AI	AJ	AK	AL	AM	AN	AO	AP	AQ	AR	AS	AT	AU	AV
313				X									X	X				1	
314										1	1	1	X	X		1			
315				X								1	X	X			X		
316			1	1			1			1	1	1		X		1	1	1	
317				X			1					1	X	X		1		1	
318												1	X	X					
319													X	X					
320				X						X			X	X			X	X	
321				X						X			X	X			X	X	
322														X				1	
323														X					
324													X	X				1	
325				X									X	X			X		
326				X									X	X				1	
327				X									X	X			X		
328				X									X	X					
329				X									X	X					
330										1								1	
331													X	X				1	
332													X	X				1	
333													X	X				1	
334													X	X					
335														X					
336													X	X					
337				X									X	X				1	
338				X									X	X					
339			1	X									X	X			X		
340			1	X									X	X					
341			1				1							X			1		
342			1				1							X					
343			1				1						X	X			1		
344			1										X	X					
345			1										X	X					
346			1				1						1	X	1				
347			1	X			1						X	X			X		
348			1	X			1						X	X					
349				1			1							X					
350				1			1							X					
351				1			1							X					
352				1			1							X					
353				1			1						X	X					
354				1			1							X	1				
355				1			1							X					
356				1										X					
357				1										X					
358				1															
359					1									X					
360						1								X					
361						1								X					
362						1		1					X	X					
363				X		1							X	X			X	X	
364						1		1											
365						1													

For peer review only

	AD	AE	AF	AG	AH	AI	AJ	AK	AL	AM	AN	AO	AP	AQ	AR	AS	AT	AU	AV
366				X		1							X	X			X		
367						1													
368						1								X					
369						1							X	X					
370				X		1							X	X					
371						1													
372						1								X					
373						1								X					
374						1													
375						1													
376						1													
377						1							X	X					
378							1							X					
379				X			1			X			X	X			X	X	
380							1						X	X	1				
381				X			1						X	X					
382							1							X					
383				X			1						X	X					
384							1						X	X	1				
385							1						X	X					
386				X			1						X	X			X		
387				X			1						X	X					
388							1							X					
389				X			1			X		1	X	X		1	X	X	
390				X			1						X	X					
391				X			1			X			X	X			X	X	
392							1												
393				X			1						X	X			X		
394				X			1						X	X					
395							1							X					
396							1							X					
397				X			1			X		1	X	X			X	X	
398							1							X					
399				X			1						X	X			X		
400				X			1						X	X					
401							1							X	1				
402				X			1						X	X			X		
403							1							X					
404							1						X	X					
405				X			1						X	X			X		
406				X			1						X	X			X		
407							1												
408				X			1						X	X			X		
409							1												
410				X			1						X	X			X		
411							1												
412				X			1						X	X			X	X	
413							1						X	X	1				
414				X			1						X	X			X		
415				X			1						X	X			X		
416							1							X					
417							1						X	X					
418							1							X					

	AD	AE	AF	AG	AH	AI	AJ	AK	AL	AM	AN	AO	AP	AQ	AR	AS	AT	AU	AV
419				X			1						X	X					
420				X			1						X	X			X		
421							1						X	X					
422							1							X					
423				X			1						X	X			X		
424							1						X	X	1	1			
425							1							X					
426							1							X					
427							1							X					
428							1							X			1		
429							1							X					
430				X			1						X	X					
431				X			1						X	X					
432				X			1						X	X			X		
433								1					X	X					
434								1					X	X					
435								1											
436								1											
437				X				1					X	X			X		
438								1					X	X					
439								1											
440				X				1					X	X					
441								1						X					
442				X				1					X	X					
443								1											
444				X					1				X	X			X		
445									1										
446									1										
447									1										
448									1										
449									1					X					
450									1					X					
451									1										
452									1										
453									1										
454									1				X	X					
455				X					1				X	X					
456									1										
457									1					X					
458				X					1				X	X					
459									1										
460									1					X					
461									1										
462									1				X	X					
463									1										
464				X					1				X	X			X		
465									1										
466									1										
467									1										
468									1										
469									1										
470									1				X	X					
471									1										

For peer review only

	AD	AE	AF	AG	AH	AI	AJ	AK	AL	AM	AN	AO	AP	AQ	AR	AS	AT	AU	AV
472									1										
473									1										
474									1										
475									1										
476				X					1				X						
477									1										
478									1										
479									1					X					
480									1										
481									1										
482				X					1	X			X	X			X		
483									1										
484		X		X		X		X	X	X	X	1	X	X			X	X	X
485		X		X		X		X	X	X	X	1	X	X			X	X	X
486		X		X		X		X	X	X	X	1	X	X		1	X	X	X
487		X		X		X		X	X	X	X	1	X	X			X	X	X
488		X		X		X		X	X	X	X	1	X	X		1	X	X	X
489														X	1				
490															1				
491		X		X		X		X	X	X	X		X	X			X	X	X
492														X		1			
493	X	X	X	X	X	X	X	X	X	X	X	X	X	X	X	1	X	X	X
494														1					
495														1					
496														1					
497														1					
498														1					
499														1					
500														1					
501														1					
502														1					
503														1					
504														1					
505														1					
506														1					
507														1					
508														1					
509														1					
510														1					
511														1					
512														1					
513														1					
514														1					
515														1					
516														X			1		
517														X			1		
518																		1	
519													X	X				1	
520														X				1	
521														X				1	
522													X	X				1	
523													X	X				1	
524																		1	

For peer review only

	AD	AE	AF	AG	AH	AI	AJ	AK	AL	AM	AN	AO	AP	AQ	AR	AS	AT	AU	AV
525													X	X				1	
526														X				1	
527														X				1	
528				X									X	X				1	
529														X				1	
530														X				1	
531													X	X				1	
532														X				1	
533														X				1	
534																		1	
535													X	X				1	
536														X				1	
537														X				1	
538														X				1	
539														X				1	
540													X	X				1	
541													X	X				1	
542													X	X					1
543														X					1
544				X									X	X					1
545														X					1
546														X					1
547																			
548														X					
549														X					
550		X		X		X		X	X	X	X		X	X			X	X	X
551																			
552														X					
553																			
554																			
555																			
556													X	X					
557																			
558																			
559																			
560																			
561																			
562																			
563														X					
564																			
565										X			X	X			X	X	
566													X	X					
567																			
568																			
569														X					
570														X					
571																			
572														X					
573														X					
574																			
575																			
576														X					
577																			

For peer review only

	AD	AE	AF	AG	AH	AI	AJ	AK	AL	AM	AN	AO	AP	AQ	AR	AS	AT	AU	AV
578														X					
579																			
580													X	X					
581														X					
582														X					
583																			
584														X					
585														X					
586																			
587																			
588														X					
589																			
590														X					
591														X					
592																			
593																			
594														X					
595													X	X					
596																			
597														X					
598														X					
599														X					
600														X					
601													X	X					
602														X					
603														X					
604														X					
605																			
606														X					
607														X					
608														X					
609														X					
610														X					
611														X					
612														X					
613				X									X	X					
614				X						X			X	X			X	X	
615													X	X					
616				X									X	X					
617														X					
618				X						X			X	X			X	X	
619				X						X			X	X			X	X	
620																			
621																			
622																			
623																			
624																			
625																			
626																			
627																			
628																			
629																			
630																			

For peer review only

	AD	AE	AF	AG	AH	AI	AJ	AK	AL	AM	AN	AO	AP	AQ	AR	AS	AT	AU	AV
631																			
632																			
633																			
634																			
635																			
636																			
637																			
638																			
639																			
640																			
641																			
642																			
643																			
644																			
645																			
646																			
647																			
648																			
649																			
650																			
651																			
652																			
653																			
654																			
655																			
656																			
657																			
658																			
659																			
660																			
661																			
662																			
663																			
664																			
665																			
666																			
667																			
668																			
669																			
670																			
671																			
672																			
673																			
674																			
675																			
676																			
677																			
678																			
679																			
680																			
681																			
682																			
683																			

For peer review only

	AD	AE	AF	AG	AH	AI	AJ	AK	AL	AM	AN	AO	AP	AQ	AR	AS	AT	AU	AV
684																			
685																			
686																			
687																			
688																			
689																			
690																			
691																			
692																			
693																			
694																			
695																			
696																			
697																			
698																			
699																			
700																			
701																			
702																			
703																			
704																			
705																			
706																			
707																			
708																			
709																			
710																			
711																			
712																			
713																			
714																			
715																			
716																			
717																			
718																			
719																			
720																			
721																			
722																			
723																			
724																			
725																			
726																			
727																			
728																			
729																			
730																			
731																			
732																			
733																			
734																			
735																			
736																			

For peer review only

	AD	AE	AF	AG	AH	AI	AJ	AK	AL	AM	AN	AO	AP	AQ	AR	AS	AT	AU	AV
737																			
738																			
739																			
740																			
741																			
742																			
743																			
744																			
745																			
746																			
747																			
748																			
749																			
750																			
751																			
752																			
753																			
754																			
755																			
756																			
757																			
758																			
759																			
760																			
761																			
762																			
763																			
764																			
765																			
766																			
767																			
768																			
769																			
770																			
771																			
772																			
773																			
774																			
775																			
776																			
777																			
778																			
779																			
780																			
781																			
782																			
783																			
784																			
785																			
786																			
787																			
788																			
789																			

For peer review only

	AD	AE	AF	AG	AH	AI	AJ	AK	AL	AM	AN	AO	AP	AQ	AR	AS	AT	AU	AV
790																			
791																			
792																			
793																			
794																			
795																			
796																			
797																			
798																			
799																			
800																			
801																			
802																			
803																			
804																			
805																			
806																			
807																			
808																			
809																			
810																			
811																			
812																			
813																			
814																			
815																			
816																			
817																			
818																			
819																			
820																			
821																			
822																			
823																			
824																			
825																			
826																			
827																			
828																			
829																			
830																			
831																			
832																			
833																			
834																			
835																			
836																			
837																			
838																			
839																			
840																			
841																			
842																			

For peer review only

	AD	AE	AF	AG	AH	AI	AJ	AK	AL	AM	AN	AO	AP	AQ	AR	AS	AT	AU	AV
843																			
844																			
845																			
846																			
847																			
848																			
849																			
850																			
851																			
852																			
853																			
854																			
855																			
856																			
857																			
858																			
859																			
860																			
861																			
862																			
863																			
864																			
865																			
866																			
867																			
868																			
869																			
870																			
871																			
872																			
873																			
874																			
875																			
876																			
877																			
878																			
879																			
880																			
881																			
882																			
883																			
884																			
885																			
886																			
887																			
888																			
889																			
890																			
891																			
892																			
893																			
894																			
895																			

For peer review only

	AD	AE	AF	AG	AH	AI	AJ	AK	AL	AM	AN	AO	AP	AQ	AR	AS	AT	AU	AV
896																			
897																			
898																			
899																			
900																			
901																			
902																			
903																			
904																			
905																			
906																			
907																			
908																			
909																			
910																			
911																			
912																			
913																			
914																			
915																			
916																			
917																			
918																			
919																			
920																			
921																			
922																			
923																			
924																			
925																			
926																			
927																			
928																			
929																			
930																			
931																			
932																			
933																			
934																			
935																			
936																			
937																			
938																			
939																			
940																			
941																			
942																			
943																			
944																			
945																			
946																			
947																			
948																			

For peer review only

	AD	AE	AF	AG	AH	AI	AJ	AK	AL	AM	AN	AO	AP	AQ	AR	AS	AT	AU	AV
949																			
950																			
951																			
952																			
953																			
954																			
955																			
956																			
957																			
958																			
959																			
960																			
961																			
962																			
963																			
964																			
965																			
966																			
967																			
968																			
969																			
970																			
971																			
972																			
973																			
974																			
975																			
976																			
977																			
978																			
979																			
980																			
981																			
982																			
983																			
984																			
985																			
986																			
987																			
988																			
989																			
990																			
991																			
992																			
993																			
994																			
995																			
996																			
997																			
998																			
999																			
1000																			
1001																			

For peer review only

	AD	AE	AF	AG	AH	AI	AJ	AK	AL	AM	AN	AO	AP	AQ	AR	AS	AT	AU	AV
1002																			
1003																			
1004																			
1005																			
1008	[redacted]																		

For peer review only

	AW	AX	AY	AZ	BA	BB	BC	BD	BE	BF	BG	BH	BI	BJ	BK	BL	BM	BN	BO
1																			
2																			
3																			
4																			
5	**5174 Bennett 201**	**5810 Lucero 2019**	**4421 Treanor 201**	**5244 Goeman 20**	**5375 Greenwood**														
6																			
7	X	X	X	X	X														
8				X	X														
9																			
10																			
11																			
12				X	X														
13																			
14																			
15																			
16																			
17																			
18																			
19																			
20																			
21																			
22																			
23																			
24				X	X														
25				X	X														
26				X	X														
27																			
28																			
29				X	X														
30																			
31																			
32				X	X														
33																			
34																			
35																			
36																			
37																			
38																			
39																			
40																			
41																			
42																			
43																			
44																			
45																			
46																			
47																			

For peer review only

	AW	AX	AY	AZ	BA	BB	BC	BD	BE	BF	BG	BH	BI	BJ	BK	BL	BM	BN	BO
48																			
49																			
50																			
51																			
52																			
53																			
54																			
55																			
56																			
57																			
58			1																
59																			
60																			
61																			
62																			
63																			
64																			
65																			
66																			
67																			
68																			
69																			
70																			
71				1															
72																			
73																			
74																			
75																			
76																			
77																			
78																			
79																			
80																			
81																			
82																			
83																			
84																			
85																			
86																			
87																			
88																			
89																			
90																			
91																			
92																			
93																			
94																			
95																			
96																			
97																			
98																			
99																			
.00																			

For peer review only

	AW	AX	AY	AZ	BA	BB	BC	BD	BE	BF	BG	BH	BI	BJ	BK	BL	BM	BN	BO
101																			
102																			
103																			
104																			
105																			
106																			
107																			
108																			
109																			
110																			
111																			
112																			
113																			
114																			
115																			
116																			
117																			
118																			
119																			
120																			
121				1															
122				1															
123				1															
124				1															
125				1															
126																			
127																			
128	1			1															
129				1															
130				1															
131																			
132																			
133																			
134																			
135																			
136																			
137																			
138																			
139																			
140																			
141																			
142				1															
143																			
144																			
145																			
146																			
147																			
148																			
149																			
150																			
151				X	X														
152				X	X														
153																			

For peer review only

	AW	AX	AY	AZ	BA	BB	BC	BD	BE	BF	BG	BH	BI	BJ	BK	BL	BM	BN	BO
154																			
155																			
156																			
157																			
158																			
159																			
160																			
161																			
162																			
163				X	X														
164																			
165																			
166																			
167		1																	
168																			
169																			
170																			
171																			
172		1		1															
173																			
174																			
175																			
176				1															
177																			
178																			
179																			
180																			
181																			
182																			
183																			
184		1																	
185																			
186																			
187																			
188		1																	
189																			
190																			
191																			
192																			
193																			
194																			
195																			
196																			
197																			
198																			
199																			
200																			
201																			
202																			
203																			
204																			
205																			
206																			

For peer review only

	AW	AX	AY	AZ	BA	BB	BC	BD	BE	BF	BG	BH	BI	BJ	BK	BL	BM	BN	BO
207																			
208																			
209																			
210																			
211																			
212																			
213																			
214																			
215																			
216		1																	
217																			
218																			
219		1																	
220																			
221																			
222																			
223																			
224																			
225																			
226																			
227				1															
228																			
229																			
230																			
231																			
232																			
233																			
234																			
235																			
236																			
237																			
238			1																
239																			
240																			
241																			
242																			
243			1																
244																			
245																			
246																			
247																			
248																			
249																			
250																			
251																			
252																			
253																			
254																			
255																			
256																			
257																			
258																			
259																			

For peer review only

	AW	AX	AY	AZ	BA	BB	BC	BD	BE	BF	BG	BH	BI	BJ	BK	BL	BM	BN	BO
260		1																	
261																			
262																			
263																			
264																			
265																			
266																			
267																			
268																			
269																			
270																			
271																			
272																			
273																			
274																			
275																			
276																			
277																			
278																			
279																			
280																			
281																			
282				1															
283																			
284	1																		
285																			
286																			
287																			
288																			
289																			
290																			
291	1																		
292																			
293																			
294																			
295																			
296				1															
297																			
298																			
299					1														
300				1															
301																			
302		1		1															
303																			
304																			
305	1																		
306																			
307	1																		
308				1															
309																			
310																			
311	1																		
312																			

For peer review only

	AW	AX	AY	AZ	BA	BB	BC	BD	BE	BF	BG	BH	BI	BJ	BK	BL	BM	BN	BO
313				X	X														
314																			
315				X	X														
316		1																	
317				X	X														
318																			
319																			
320				X	X														
321				X	X														
322																			
323																			
324																			
325				X	X														
326				X	X														
327				X	X														
328				X	X														
329				X	X														
330																			
331																			
332																			
333																			
334																			
335																			
336																			
337				X	X														
338				X	X														
339				X	X														
340				X	X														
341																			
342																			
343																			
344																			
345																			
346																			
347				X	X														
348				X	X														
349																			
350																			
351				1															
352		1																	
353																			
354																			
355																			
356																			
357																			
358																			
359																			
360																			
361																			
362																			
363				X	X														
364																			
365																			

For peer review only

	AW	AX	AY	AZ	BA	BB	BC	BD	BE	BF	BG	BH	BI	BJ	BK	BL	BM	BN	BO
366				X	X														
367																			
368																			
369																			
370																			
371																			
372																			
373																			
374																			
375																			
376																			
377																			
378																			
379				X	X														
380																			
381				X	X														
382																			
383				X	X														
384																			
385																			
386				X	X														
387				X	X														
388																			
389				X	X														
390				X	X														
391	1			X	X														
392																			
393				X	X														
394				X	X														
395																			
396																			
397				X	X														
398																			
399				X	X														
400				X	X														
401																			
402				X	X														
403																			
404																			
405				X	X														
406				X	X														
407																			
408				X	X														
409																			
410				X	X														
411																			
412				X	X														
413																			
414				X	X														
415				X	X														
416																			
417																			
418																			

For peer review only

	AW	AX	AY	AZ	BA	BB	BC	BD	BE	BF	BG	BH	BI	BJ	BK	BL	BM	BN	BO
419				X	X														
420				X	X														
421																			
422																			
423				X	X														
424		1																	
425																			
426																			
427	1																		
428																			
429																			
430				X	X														
431				X	X														
432				X	X														
433																			
434				1															
435																			
436																			
437				X	X														
438																			
439																			
440				X	X														
441																			
442				X	X														
443																			
444				X	X														
445																			
446																			
447																			
448																			
449																			
450																			
451																			
452																			
453																			
454																			
455				X	X														
456																			
457																			
458				X	X														
459																			
460																			
461																			
462																			
463																			
464				X	X														
465																			
466																			
467																			
468																			
469																			
470																			
471																			

For peer review only

	AW	AX	AY	AZ	BA	BB	BC	BD	BE	BF	BG	BH	BI	BJ	BK	BL	BM	BN	BO
472																			
473																			
474																			
475																			
476				X	X														
477																			
478																			
479																			
480																			
481																			
482				X	X														
483																			
484	X	X	X	X	X														
485	X	X	X	X	X														
486	X	X	X	X	X														
487	X	X	X	X	X														
488	X	X	X	X	X														
489																			
490																			
491	X	X	X	X	X														
492																			
493	X	X	X	X	X														
494																			
495																			
496																			
497																			
498																			
499																			
500																			
501																			
502																			
503																			
504																			
505																			
506																			
507																			
508																			
509																			
510																			
511																			
512																			
513																			
514																			
515																			
516																			
517																			
518																			
519																			
520																			
521																			
522																			
523																			
524																			

For peer review only

For peer review only - http://bmjopen.bmj.com/site/about/guidelines.xhtml

	AW	AX	AY	AZ	BA	BB	BC	BD	BE	BF	BG	BH	BI	BJ	BK	BL	BM	BN	BO
525																			
526																			
527																			
528				X	X														
529																			
530																			
531																			
532																			
533																			
534																			
535																			
536																			
537																			
538																			
539																			
540																			
541																			
542																			
543																			
544																			
545																			
546																			
547	1																		
548	1																		
549	1																		
550	1	X	X	X	X														
551		1																	
552			1																
553			1																
554			1																
555			1																
556			1																
557			1																
558			1																
559			1																
560			1																
561			1																
562			1																
563			1																
564			1																
565			1	X	X														
566			1																
567				1															
568				1															
569				1															
570				1															
571				1	1														
572				1															
573				1															
574				1															
575				1															
576				1															
577				1															

For peer review only

	AW	AX	AY	AZ	BA	BB	BC	BD	BE	BF	BG	BH	BI	BJ	BK	BL	BM	BN	BO
578				1															
579				1															
580				1															
581				1															
582				1															
583				1															
584				1															
585				1															
586				1															
587				1															
588				1															
589				1															
590				1															
591				1															
592				1															
593				1															
594					1														
595					1														
596																			
597																			
598																			
599																			
600																			
601																			
602																			
603																			
604																			
605																			
606																			
607																			
608																			
609																			
610																			
611																			
612																			
613				X	X														
614				X	X														
615																			
616				X	X														
617																			
618				X	X														
619				X	X														
620																			
621																			
622																			
623																			
624																			
625																			
626																			
627																			
628																			
629																			
630																			

For peer review only

	AW	AX	AY	AZ	BA	BB	BC	BD	BE	BF	BG	BH	BI	BJ	BK	BL	BM	BN	BO
631																			
632																			
633																			
634																			
635																			
636																			
637																			
638																			
639																			
640																			
641																			
642																			
643																			
644																			
645																			
646																			
647																			
648																			
649																			
650																			
651																			
652																			
653																			
654																			
655																			
656																			
657																			
658																			
659																			
660																			
661																			
662																			
663																			
664																			
665																			
666																			
667																			
668																			
669																			
670																			
671																			
672																			
673																			
674																			
675																			
676																			
677																			
678																			
679																			
680																			
681																			
682																			
683																			

For peer review only

	AW	AX	AY	AZ	BA	BB	BC	BD	BE	BF	BG	BH	BI	BJ	BK	BL	BM	BN	BO
684																			
685																			
686																			
687																			
688																			
689																			
690																			
691																			
692																			
693																			
694																			
695																			
696																			
697																			
698																			
699																			
700																			
701																			
702																			
703																			
704																			
705																			
706																			
707																			
708																			
709																			
710																			
711																			
712																			
713																			
714																			
715																			
716																			
717																			
718																			
719																			
720																			
721																			
722																			
723																			
724																			
725																			
726																			
727																			
728																			
729																			
730																			
731																			
732																			
733																			
734																			
735																			
736																			

For peer review only

	AW	AX	AY	AZ	BA	BB	BC	BD	BE	BF	BG	BH	BI	BJ	BK	BL	BM	BN	BO
737																			
738																			
739																			
740																			
741																			
742																			
743																			
744																			
745																			
746																			
747																			
748																			
749																			
750																			
751																			
752																			
753																			
754																			
755																			
756																			
757																			
758																			
759																			
760																			
761																			
762																			
763																			
764																			
765																			
766																			
767																			
768																			
769																			
770																			
771																			
772																			
773																			
774																			
775																			
776																			
777																			
778																			
779																			
780																			
781																			
782																			
783																			
784																			
785																			
786																			
787																			
788																			
789																			

For peer review only

	AW	AX	AY	AZ	BA	BB	BC	BD	BE	BF	BG	BH	BI	BJ	BK	BL	BM	BN	BO
790																			
791																			
792																			
793																			
794																			
795																			
796																			
797																			
798																			
799																			
800																			
801																			
802																			
803																			
804																			
805																			
806																			
807																			
808																			
809																			
810																			
811																			
812																			
813																			
814																			
815																			
816																			
817																			
818																			
819																			
820																			
821																			
822																			
823																			
824																			
825																			
826																			
827																			
828																			
829																			
830																			
831																			
832																			
833																			
834																			
835																			
836																			
837																			
838																			
839																			
840																			
841																			
842																			

For peer review only

	AW	AX	AY	AZ	BA	BB	BC	BD	BE	BF	BG	BH	BI	BJ	BK	BL	BM	BN	BO
843																			
844																			
845																			
846																			
847																			
848																			
849																			
850																			
851																			
852																			
853																			
854																			
855																			
856																			
857																			
858																			
859																			
860																			
861																			
862																			
863																			
864																			
865																			
866																			
867																			
868																			
869																			
870																			
871																			
872																			
873																			
874																			
875																			
876																			
877																			
878																			
879																			
880																			
881																			
882																			
883																			
884																			
885																			
886																			
887																			
888																			
889																			
890																			
891																			
892																			
893																			
894																			
895																			

For peer review only

	AW	AX	AY	AZ	BA	BB	BC	BD	BE	BF	BG	BH	BI	BJ	BK	BL	BM	BN	BO
896																			
897																			
898																			
899																			
900																			
901																			
902																			
903																			
904																			
905																			
906																			
907																			
908																			
909																			
910																			
911																			
912																			
913																			
914																			
915																			
916																			
917																			
918																			
919																			
920																			
921																			
922																			
923																			
924																			
925																			
926																			
927																			
928																			
929																			
930																			
931																			
932																			
933																			
934																			
935																			
936																			
937																			
938																			
939																			
940																			
941																			
942																			
943																			
944																			
945																			
946																			
947																			
948																			

For peer review only

	AW	AX	AY	AZ	BA	BB	BC	BD	BE	BF	BG	BH	BI	BJ	BK	BL	BM	BN	BO
949																			
950																			
951																			
952																			
953																			
954																			
955																			
956																			
957																			
958																			
959																			
960																			
961																			
962																			
963																			
964																			
965																			
966																			
967																			
968																			
969																			
970																			
971																			
972																			
973																			
974																			
975																			
976																			
977																			
978																			
979																			
980																			
981																			
982																			
983																			
984																			
985																			
986																			
987																			
988																			
989																			
990																			
991																			
992																			
993																			
994																			
995																			
996																			
997																			
998																			
999																			
1000																			
1001																			

For peer review only

	AW	AX	AY	AZ	BA	BB	BC	BD	BE	BF	BG	BH	BI	BJ	BK	BL	BM	BN	BO
1002																			
1003																			
1004																			
1005																			
1008	[redacted]																		

For peer review only

	BP	BQ	BR	BS	BT
1					
2					
3					
4					
5					
6					
7					
8					
9					
10					
11					
12					
13					
14					
15					
16					
17					
18					
19					
20					
21					
22					
23					
24					
25					
26					
27					
28					
29					
30					
31					
32					
33					
34					
35					
36					
37					
38					
39					
40					
41					
42					
43					
44					
45					
46					
47					

For peer review only

	BP	BQ	BR	BS	BT
48					
49					
50					
51					
52					
53					
54					
55					
56					
57					
58					
59					
60					
61					
62					
63					
64					
65					
66					
67					
68					
69					
70					
71					
72					
73					
74					
75					
76					
77					
78					
79					
80					
81					
82					
83					
84					
85					
86					
87					
88					
89					
90					
91					
92					
93					
94					
95					
96					
97					
98					
99					
100					

For peer review only

	BP	BQ	BR	BS	BT
101					
102					
103					
104					
105					
106					
107					
108					
109					
110					
111					
112					
113					
114					
115					
116					
117					
118					
119					
120					
121					
122					
123					
124					
125					
126					
127					
128					
129					
130					
131					
132					
133					
134					
135					
136					
137					
138					
139					
140					
141					
142					
143					
144					
145					
146					
147					
148					
149					
150					
151					
152					
153					

For peer review only

	BP	BQ	BR	BS	BT
154					
155					
156					
157					
158					
159					
160					
161					
162					
163					
164					
165					
166					
167					
168					
169					
170					
171					
172					
173					
174					
175					
176					
177					
178					
179					
180					
181					
182					
183					
184					
185					
186					
187					
188					
189					
190					
191					
192					
193					
194					
195					
196					
197					
198					
199					
200					
201					
202					
203					
204					
205					
206					

For peer review only

	BP	BQ	BR	BS	BT
207					
208					
209					
210					
211					
212					
213					
214					
215					
216					
217					
218					
219					
220					
221					
222					
223					
224					
225					
226					
227					
228					
229					
230					
231					
232					
233					
234					
235					
236					
237					
238					
239					
240					
241					
242					
243					
244					
245					
246					
247					
248					
249					
250					
251					
252					
253					
254					
255					
256					
257					
258					
259					

For peer review only

	BP	BQ	BR	BS	BT
260					
261					
262					
263					
264					
265					
266					
267					
268					
269					
270					
271					
272					
273					
274					
275					
276					
277					
278					
279					
280					
281					
282					
283					
284					
285					
286					
287					
288					
289					
290					
291					
292					
293					
294					
295					
296					
297					
298					
299					
300					
301					
302					
303					
304					
305					
306					
307					
308					
309					
310					
311					
312					

For peer review only

	BP	BQ	BR	BS	BT
313					
314					
315					
316					
317					
318					
319					
320					
321					
322					
323					
324					
325					
326					
327					
328					
329					
330					
331					
332					
333					
334					
335					
336					
337					
338					
339					
340					
341					
342					
343					
344					
345					
346					
347					
348					
349					
350					
351					
352					
353					
354					
355					
356					
357					
358					
359					
360					
361					
362					
363					
364					
365					

For peer review only

	BP	BQ	BR	BS	BT
366					
367					
368					
369					
370					
371					
372					
373					
374					
375					
376					
377					
378					
379					
380					
381					
382					
383					
384					
385					
386					
387					
388					
389					
390					
391					
392					
393					
394					
395					
396					
397					
398					
399					
400					
401					
402					
403					
404					
405					
406					
407					
408					
409					
410					
411					
412					
413					
414					
415					
416					
417					
418					

For peer review only

	BP	BQ	BR	BS	BT
419					
420					
421					
422					
423					
424					
425					
426					
427					
428					
429					
430					
431					
432					
433					
434					
435					
436					
437					
438					
439					
440					
441					
442					
443					
444					
445					
446					
447					
448					
449					
450					
451					
452					
453					
454					
455					
456					
457					
458					
459					
460					
461					
462					
463					
464					
465					
466					
467					
468					
469					
470					
471					

For peer review only

	BP	BQ	BR	BS	BT
472					
473					
474					
475					
476					
477					
478					
479					
480					
481					
482					
483					
484					
485					
486					
487					
488					
489					
490					
491					
492					
493					
494					
495					
496					
497					
498					
499					
500					
501					
502					
503					
504					
505					
506					
507					
508					
509					
510					
511					
512					
513					
514					
515					
516					
517					
518					
519					
520					
521					
522					
523					
524					

For peer review only

	BP	BQ	BR	BS	BT
525					
526					
527					
528					
529					
530					
531					
532					
533					
534					
535					
536					
537					
538					
539					
540					
541					
542					
543					
544					
545					
546					
547					
548					
549					
550					
551					
552					
553					
554					
555					
556					
557					
558					
559					
560					
561					
562					
563					
564					
565					
566					
567					
568					
569					
570					
571					
572					
573					
574					
575					
576					
577					

For peer review only

	BP	BQ	BR	BS	BT
578					
579					
580					
581					
582					
583					
584					
585					
586					
587					
588					
589					
590					
591					
592					
593					
594					
595					
596					
597					
598					
599					
600					
601					
602					
603					
604					
605					
606					
607					
608					
609					
610					
611					
612					
613					
614					
615					
616					
617					
618					
619					
620					
621					
622					
623					
624					
625					
626					
627					
628					
629					
630					

For peer review only

	BP	BQ	BR	BS	BT
631					
632					
633					
634					
635					
636					
637					
638					
639					
640					
641					
642					
643					
644					
645					
646					
647					
648					
649					
650					
651					
652					
653					
654					
655					
656					
657					
658					
659					
660					
661					
662					
663					
664					
665					
666					
667					
668					
669					
670					
671					
672					
673					
674					
675					
676					
677					
678					
679					
680					
681					
682					
683					

For peer review only

	BP	BQ	BR	BS	BT
684					
685					
686					
687					
688					
689					
690					
691					
692					
693					
694					
695					
696					
697					
698					
699					
700					
701					
702					
703					
704					
705					
706					
707					
708					
709					
710					
711					
712					
713					
714					
715					
716					
717					
718					
719					
720					
721					
722					
723					
724					
725					
726					
727					
728					
729					
730					
731					
732					
733					
734					
735					
736					

For peer review only

	BP	BQ	BR	BS	BT
737					
738					
739					
740					
741					
742					
743					
744					
745					
746					
747					
748					
749					
750					
751					
752					
753					
754					
755					
756					
757					
758					
759					
760					
761					
762					
763					
764					
765					
766					
767					
768					
769					
770					
771					
772					
773					
774					
775					
776					
777					
778					
779					
780					
781					
782					
783					
784					
785					
786					
787					
788					
789					

For peer review only

	BP	BQ	BR	BS	BT
790					
791					
792					
793					
794					
795					
796					
797					
798					
799					
800					
801					
802					
803					
804					
805					
806					
807					
808					
809					
810					
811					
812					
813					
814					
815					
816					
817					
818					
819					
820					
821					
822					
823					
824					
825					
826					
827					
828					
829					
830					
831					
832					
833					
834					
835					
836					
837					
838					
839					
840					
841					
842					

For peer review only

	BP	BQ	BR	BS	BT
843					
844					
845					
846					
847					
848					
849					
850					
851					
852					
853					
854					
855					
856					
857					
858					
859					
860					
861					
862					
863					
864					
865					
866					
867					
868					
869					
870					
871					
872					
873					
874					
875					
876					
877					
878					
879					
880					
881					
882					
883					
884					
885					
886					
887					
888					
889					
890					
891					
892					
893					
894					
895					

For peer review only

	BP	BQ	BR	BS	BT
896					
897					
898					
899					
900					
901					
902					
903					
904					
905					
906					
907					
908					
909					
910					
911					
912					
913					
914					
915					
916					
917					
918					
919					
920					
921					
922					
923					
924					
925					
926					
927					
928					
929					
930					
931					
932					
933					
934					
935					
936					
937					
938					
939					
940					
941					
942					
943					
944					
945					
946					
947					
948					

For peer review only

	BP	BQ	BR	BS	BT
949					
950					
951					
952					
953					
954					
955					
956					
957					
958					
959					
960					
961					
962					
963					
964					
965					
966					
967					
968					
969					
970					
971					
972					
973					
974					
975					
976					
977					
978					
979					
980					
981					
982					
983					
984					
985					
986					
987					
988					
989					
990					
991					
992					
993					
994					
995					
996					
997					
998					
999					
1000					
1001					

For peer review only

	BP	BQ	BR	BS	BT
1002					
1003					
1004					
1005					
1008					

For peer review only

Overall results		
Number of columns (number of reviews)	c	51
Number of rows (number of index publications)	r	614
Number of included primary studies (including double counting)	N	1071
Covered area	N/(rc)	3.42%
Corrected covered area	(N-r)/(rc-r)	1.49%
Interpretation of overlap	Slight overlap	
Structural Zeros	X	3598
Corrected covered area (adjusting by structural zeros)	(N-r)/(rc-r-X)	1.69%
N° of non-overlapped primary studies	In 1 SR	395
Number of overlapped primary studies	In 2 SRs	107
	In 3 SRs	45
	In 4 SRs	23
	In 5 SRs	15
	In 6 SRs	10
	In 7 SRs	5
	In 8 SRs	2
	In 9 SRs	3
	In 10 SRs	1
	In 11 SRs	0
	In 12 SRs	1
	In 13 SRs	0
	In 14 SRs	0
	In 15 or more SRs	0

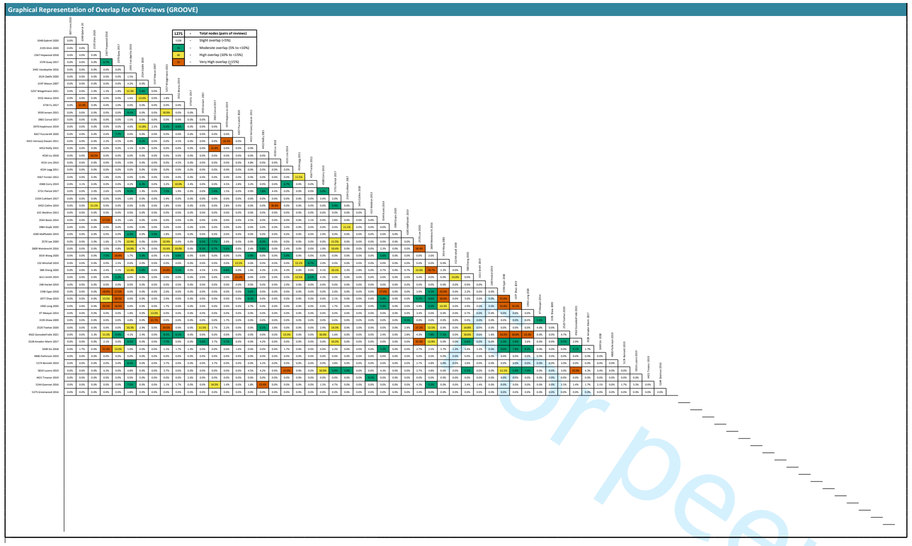

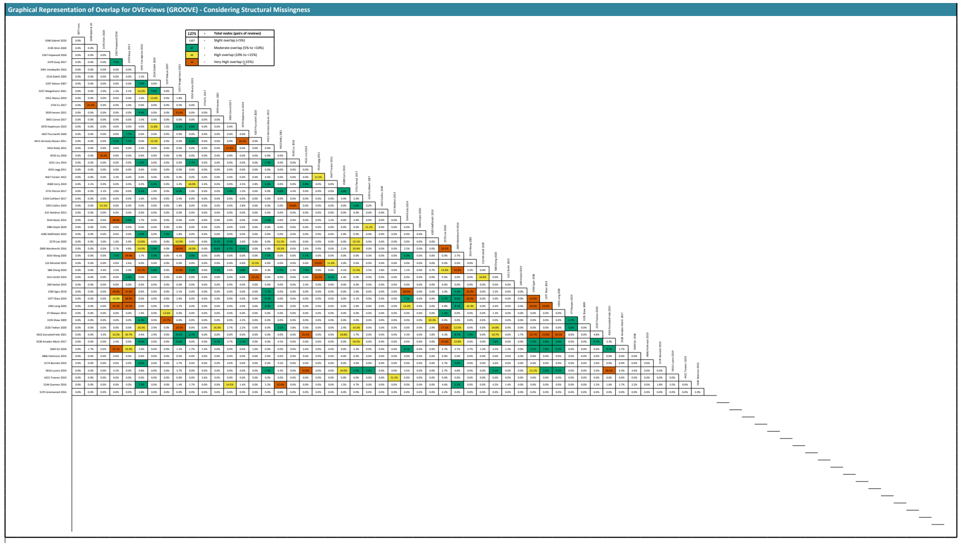

Overall results

Number of columns (number of reviews)	c	51
Number of rows (number of index publications)	r	614
Number of included primary studies (including double counting)	N	1071
Covered area	N/(rc)	3.42%
Corrected covered area	(N-r)/(rc-r)	1.49%
Interpretation of overlap	**Slight overlap**	
Structural Zeros	X	3598
Corrected covered area (adjusting by structural zeros)	(N-r)/(rc-r-X)	1.69%

N° of non-overlapped primary studies	In 1 SR	395
Number of overlapped primary studies	In 2 SRs	107
	In 3 SRs	45
	In 4 SRs	23
	In 5 SRs	15
	In 6 SRs	10
	In 7 SRs	5
	In 8 SRs	2
	In 9 SRs	3
	In 10 SRs	1
	In 11 SRs	0
	In 12 SRs	1
	In 13 SRs	0
	In 14 SRs	0
	In 15 or more SRs	0

G

For peer review only

raphical Representation of Overlap for OVErviews (GRO

	807 Irons 2020	1048 Gabriel 202	2195 Shim 2020	2367 Hopwood 2018	2370 Guay 2017	2491 Vandepitte 2016	2524 Zabihi 2020	3197 Mason 2007	5257 Wiegelmann 2021	3552 Akarsu 2019
1048 Gabriel 2020	0.0%									
2195 Shim 2020	0.0%	0.0%								
2367 Hopwood 2018	0.0%	0.0%	0.0%							
2370 Guay 2017	0.0%	0.0%	0.0%	6.7%						
2491 Vandepitte 2016	0.0%	0.0%	0.0%	0.0%	0.0%					
2524 Zabihi 2020	0.0%	0.0%	0.0%	0.0%	0.0%	1.5%				
3197 Mason 2007	0.0%	0.0%	0.0%	0.0%	0.0%	4.2%	0.0%			
5257 Wiegelmann 2021	0.0%	0.0%	2.0%	1.3%	1.8%	11.5%	5.6%	0.0%		
3552 Akarsu 2019	0.0%	0.0%	0.0%	0.0%	0.0%	1.6%	13.6%	0.0%	1.8%	
3734 Fu 2017	0.0%	21.4%	0.0%	0.0%	3.0%	0.0%	0.0%	0.0%	0.0%	0.0%
3939 Jensen 2015	0.0%	0.0%	0.0%	0.0%	0.0%	9.1%	0.0%	0.0%	10.9%	0.0%
3965 Corvol 2017	0.0%	0.0%	0.0%	0.0%	0.0%	1.5%	0.0%	0.0%	0.0%	0.0%
3970 Hopkinson 2019	0.0%	0.0%	0.0%	0.0%	0.0%	4.0%	11.8%	2.2%	6.2%	8.8%
4267 Pucciarelli 2020	0.0%	0.0%	0.0%	0.0%	7.7%	0.0%	0.0%	0.0%	0.0%	0.0%
4415 Vernooij-Dassen 2011	0.0%	0.0%	0.0%	2.2%	4.5%	0.0%	9.1%	0.0%	0.0%	4.5%
4452 Reilly 2015	0.0%	0.0%	0.0%	0.0%	0.0%	3.1%	0.0%	0.0%	0.0%	0.0%
4530 Liu 2018	0.0%	0.0%	18.2%	0.0%	0.0%	0.0%	0.0%	0.0%	0.0%	0.0%
4531 Lins 2014	0.0%	0.0%	0.0%	0.0%	0.0%	4.9%	0.0%	0.0%	0.0%	4.5%
4534 Legg 2011	0.0%	0.0%	0.0%	0.0%	0.0%	0.0%	0.0%	0.0%	0.0%	0.0%
4567 Forster 2012	0.0%	0.0%	0.0%	1.8%	0.0%	0.0%	0.0%	0.0%	0.0%	0.0%
4588 Corry 2019	0.0%	3.1%	0.0%	0.0%	0.0%	4.2%	6.3%	0.0%	3.2%	10.0%
5731 Piersol 2017	0.0%	0.0%	2.0%	2.6%	0.0%	9.2%	1.9%	0.0%	7.5%	1.9%
2104 Cuthbert 2017	0.0%	0.0%	0.0%	0.0%	0.0%	1.6%	0.0%	0.0%	1.9%	0.0%
3453 Collins 2019	0.0%	0.0%	11.1%	0.0%	0.0%	0.0%	0.0%	0.0%	1.8%	0.0%
615 Waldron 2013	0.0%	0.0%	0.0%	0.0%	0.0%	0.0%	0.0%	0.0%	0.0%	0.0%
3544 Boots 2014	0.0%	0.0%	0.0%	17.1%	4.3%	1.6%	0.0%	0.0%	0.0%	0.0%
3984 Doyle 2020	0.0%	0.0%	0.0%	0.0%	0.0%	0.0%	0.0%	0.0%	0.0%	0.0%
4285 Maffioletti 2019	0.0%	0.0%	0.0%	0.0%	0.0%	6.3%	0.0%	5.9%	1.8%	0.0%
2570 Lee 2020	0.0%	0.0%	3.0%	1.6%	2.7%	12.9%	0.0%	0.0%	12.9%	0.0%
2809 Weinbrecht 2016	0.0%	0.0%	0.0%	3.0%	4.8%	14.9%	4.7%	0.0%	13.4%	10.0%
3014 Wang 2020	0.0%	0.0%	0.0%	7.5%	18.8%	1.7%	5.3%	0.0%	4.1%	5.6%
152 Minshall 2019	0.0%	0.0%	0.0%	0.0%	2.5%	0.0%	0.0%	0.0%	0.0%	0.0%
588 Cheng 2020	0.0%	0.0%	4.4%	2.4%	2.1%	13.4%	5.0%	0.0%	23.8%	5.1%

**1275**
1128
74
40
33

For peer review only

1611 Smith 2019	0.0%	0.0%	0.0%	0.0%	5.3%	0.0%	0.0%	0.0%	0.0%	0.0%
248 Heckel 2019	0.0%	0.0%	0.0%	0.0%	0.0%	0.0%	0.0%	0.0%	0.0%	0.0%
1596 Egan 2018	0.0%	0.0%	0.0%	18.9%	17.6%	0.0%	0.0%	0.0%	2.0%	0.0%
1977 Zhao 2019	0.0%	0.0%	0.0%	13.5%	28.6%	0.0%	0.0%	0.0%	2.0%	0.0%
1992 Leng 2020	0.0%	0.0%	0.0%	20.5%	16.0%	0.0%	0.0%	0.0%	1.7%	0.0%
XT Maayan 2014	0.0%	0.0%	0.0%	0.0%	0.0%	1.8%	0.0%	13.0%	0.0%	0.0%
3195 Shaw 2009	0.0%	0.0%	0.0%	0.0%	0.0%	4.8%	0.0%	21.7%	0.0%	0.0%
2520 Teahan 2020	0.0%	0.0%	0.0%	0.0%	0.0%	10.3%	2.9%	0.0%	24.5%	0.0%
4562 GonzalezFraile 2021	0.0%	0.0%	3.3%	11.3%	9.4%	4.1%	2.9%	0.0%	8.1%	6.1%
3528 Amador-Marin 2017	0.0%	0.0%	0.0%	2.1%	0.0%	8.3%	0.0%	0.0%	7.7%	0.0%
5049 Sin 2018	0.0%	1.7%	0.0%	21.4%	13.0%	1.0%	0.0%	0.0%	1.1%	1.7%
4806 Parkinson 2019	0.0%	0.0%	0.0%	0.0%	0.0%	0.0%	0.0%	0.0%	0.0%	0.0%
5174 Bennett 2019	0.0%	0.0%	0.0%	0.0%	0.0%	8.3%	0.0%	0.0%	3.7%	0.0%
5810 Lucero 2019	0.0%	0.0%	0.0%	4.3%	0.0%	4.8%	0.0%	0.0%	3.7%	0.0%
4421 Treanor 2019	0.0%	0.0%	0.0%	0.0%	0.0%	0.0%	0.0%	0.0%	0.0%	0.0%
5244 Goeman 2016	0.0%	0.0%	0.0%	0.0%	0.0%	7.5%	0.0%	0.0%	1.1%	1.7%
5375 Greenwood 2016	0.0%	0.0%	0.0%	0.0%	0.0%	1.8%	0.0%	0.0%	0.0%	0.0%

For peer review only

OVE)

=	**Total nodes (pairs of reviews)**
=	Slight overlap (<5%)
=	Moderate overlap (5% to <10%)
=	High overlap (10% to <15%)
=	Very High overlap (>15%)

3734 Fu 2017	3939 Jensen 2015	3965 Corvol 2017	3970 Hopkinson 2019	4267 Pucciarelli 2020	4415 Vernooij-Dassen 2011	4452 Reilly 2015	4530 Liu 2018	4531 Lins 2014	4534 Legg 2011	4567 Forster 2012	4588 Corry 2019	5731 Piersol 2017	2104 Cuthbert 2017	3453 Collins 2019
0.0%														
0.0%	0.0%													
0.0%	0.0%	0.0%												
0.0%	0.0%	0.0%	0.0%											
0.0%	0.0%	0.0%	16.1%	0.0%										
0.0%	0.0%	31.8%	0.0%	0.0%	0.0%									
0.0%	0.0%	0.0%	0.0%	0.0%	0.0%	0.0%								
0.0%	0.0%	0.0%	0.0%	0.0%	4.8%	0.0%	0.0%							
0.0%	0.0%	0.0%	0.0%	0.0%	0.0%	0.0%	0.0%	0.0%						
0.0%	0.0%	0.0%	0.0%	0.0%	0.0%	0.0%	0.0%	0.0%	11.5%					
2.4%	0.0%	0.0%	4.5%	2.8%	3.2%	0.0%	0.0%	6.7%	0.0%	0.0%				
0.0%	0.0%	7.4%	1.5%	0.0%	0.0%	7.8%	4.4%	0.0%	0.0%	0.0%	5.0%			
0.0%	0.0%	0.0%	0.0%	0.0%	0.0%	0.0%	0.0%	0.0%	0.0%	0.0%	3.4%	2.0%		
0.0%	0.0%	0.0%	2.8%	0.0%	0.0%	0.0%	30.8%	0.0%	0.0%	0.0%	0.0%	5.9%	0.0%	
0.0%	0.0%	0.0%	0.0%	0.0%	0.0%	0.0%	0.0%	0.0%	0.0%	0.0%	0.0%	0.0%	0.0%	0.0%
0.0%	0.0%	0.0%	0.0%	0.0%	4.5%	0.0%	0.0%	0.0%	0.0%	3.1%	0.0%	3.8%	0.0%	0.0%
0.0%	0.0%	0.0%	0.0%	0.0%	0.0%	0.0%	0.0%	0.0%	0.0%	0.0%	0.0%	0.0%	11.1%	0.0%
0.0%	0.0%	0.0%	0.0%	0.0%	0.0%	0.0%	0.0%	0.0%	0.0%	0.0%	2.9%	0.0%	0.0%	0.0%
0.0%	6.5%	7.7%	2.0%	0.0%	0.0%	8.3%	0.0%	0.0%	0.0%	0.0%	0.0%	11.5%	0.0%	0.0%
0.0%	8.3%	6.7%	5.6%	0.0%	2.4%	9.8%	0.0%	2.4%	0.0%	0.0%	1.9%	10.4%	0.0%	0.0%
0.0%	0.0%	0.0%	0.0%	0.0%	5.9%	0.0%	0.0%	5.9%	0.0%	0.0%	0.0%	0.0%	0.0%	0.0%
0.0%	0.0%	0.0%	0.0%	12.5%	0.0%	0.0%	0.0%	0.0%	12.1%	8.7%	2.0%	0.0%	0.0%	0.0%
0.0%	4.5%	3.5%	6.8%	0.0%	1.4%	4.3%	2.2%	4.3%	0.0%	0.0%	4.1%	10.1%	2.2%	2.8%

For peer review only

0.0%	0.0%	0.0%	0.0%	22.2%	0.0%	0.0%	0.0%	0.0%	12.5%	6.5%	4.3%	0.0%	0.0%	0.0%
0.0%	0.0%	0.0%	0.0%	0.0%	0.0%	0.0%	0.0%	2.0%	0.0%	0.0%	0.0%	0.0%	0.0%	0.0%
0.0%	0.0%	0.0%	0.0%	0.0%	5.6%	0.0%	0.0%	0.0%	0.0%	0.0%	0.0%	2.0%	0.0%	0.0%
0.0%	0.0%	0.0%	0.0%	0.0%	6.3%	0.0%	0.0%	0.0%	0.0%	0.0%	0.0%	2.1%	0.0%	0.0%
0.0%	0.0%	0.0%	0.0%	0.0%	3.7%	0.0%	0.0%	0.0%	0.0%	0.0%	0.0%	1.7%	0.0%	0.0%
0.0%	0.0%	0.0%	0.0%	0.0%	0.0%	0.0%	0.0%	0.0%	0.0%	0.0%	0.0%	0.0%	0.0%	0.0%
0.0%	0.0%	0.0%	1.7%	0.0%	0.0%	0.0%	0.0%	0.0%	0.0%	0.0%	0.0%	0.0%	0.0%	0.0%
0.0%	11.5%	2.7%	2.2%	0.0%	0.0%	6.1%	3.8%	0.0%	0.0%	0.0%	2.4%	14.3%	0.0%	3.0%
0.0%	0.0%	0.0%	0.0%	0.0%	0.0%	0.0%	0.0%	13.3%	0.0%	0.0%	10.0%	1.6%	0.0%	0.0%
0.0%	5.6%	3.7%	5.7%	0.0%	0.0%	4.2%	0.0%	0.0%	0.0%	0.0%	0.0%	10.2%	0.0%	0.0%
1.4%	0.0%	0.0%	0.0%	1.6%	0.0%	0.0%	0.0%	1.7%	0.0%	0.0%	0.0%	2.2%	0.0%	0.0%
0.0%	0.0%	0.0%	0.0%	0.0%	0.0%	0.0%	0.0%	0.0%	0.0%	0.0%	0.0%	0.0%	0.0%	0.0%
0.0%	0.0%	3.7%	0.0%	0.0%	0.0%	4.2%	0.0%	0.0%	0.0%	0.0%	0.0%	3.8%	0.0%	0.0%
0.0%	0.0%	0.0%	0.0%	0.0%	4.5%	4.2%	0.0%	15.0%	0.0%	0.0%	10.0%	5.9%	5.0%	0.0%
2.6%	0.0%	0.0%	0.0%	0.0%	0.0%	0.0%	0.0%	0.0%	0.0%	0.0%	0.0%	0.0%	0.0%	0.0%
0.0%	0.0%	14.5%	1.4%	0.0%	1.8%	15.4%	0.0%	0.0%	0.0%	0.0%	1.5%	4.7%	0.0%	0.0%
0.0%	0.0%	0.0%	0.0%	0.0%	0.0%	0.0%	0.0%	0.0%	0.0%	0.0%	0.0%	0.0%	0.0%	0.0%

For peer review only

[redacted]

For peer review only

615 Waldron 2013	3544 Boots 2014	3984 Doyle 2020	4285 Maffioletti 2019	2570 Lee 2020	2809 Weinbrecht 2016	3014 Wang 2020	152 Minshall 2019	588 Cheng 2020	Smith 2019	2019
0.0%										
0.0%	0.0%									
0.0%	0.0%	0.0%								
0.0%	0.0%	0.0%	0.0%							
0.0%	2.3%	0.0%	0.0%	18.4%						
0.0%	5.6%	0.0%	0.0%	0.0%	2.6%					
0.0%	0.0%	0.0%	0.0%	0.0%	0.0%	0.0%				
0.0%	0.7%	0.0%	0.7%	13.6%	18.7%	2.2%	0.0%			

									1611 :	248 Heckel	1596 Egan 2018	1977 Zhao 2019	1992 Leng 2020	XT Maayan 2014
0.0%	0.0%	0.0%	0.0%	0.0%	0.0%	0.0%	14.0%	0.0%						
0.0%	0.0%	0.0%	0.0%	0.0%	0.0%	0.0%	0.0%	0.0%	0.0%					
0.0%	17.6%	0.0%	0.0%	3.0%	5.3%	25.0%	0.0%	2.2%	0.0%	0.0%				
0.0%	5.9%	0.0%	0.0%	6.7%	8.6%	30.0%	0.0%	3.0%	0.0%	0.0%	55.6%			
0.0%	7.4%	0.0%	0.0%	4.9%	6.5%	14.3%	0.0%	4.9%	0.0%	0.0%	31.6%	35.3%		
0.0%	0.0%	0.0%	0.0%	3.4%	0.0%	0.0%	0.0%	0.7%	0.0%	0.0%	0.0%	0.0%	0.0%	
0.0%	0.0%	0.0%	9.1%	0.0%	0.0%	0.0%	0.0%	0.0%	0.0%	0.0%	0.0%	0.0%	0.0%	5.6%
0.0%	0.0%	0.0%	2.9%	17.1%	12.5%	0.0%	0.0%	14.8%	0.0%	0.0%	0.0%	0.0%	0.0%	4.0%
0.0%	2.9%	0.0%	2.8%	4.3%	7.8%	7.1%	0.0%	10.6%	0.0%	1.6%	19.2%	20.8%	33.3%	0.0%
0.0%	0.0%	0.0%	0.0%	22.6%	12.8%	0.0%	0.0%	6.6%	0.0%	0.0%	5.3%	5.9%	3.6%	0.0%
0.0%	5.2%	0.0%	0.0%	2.7%	2.5%	3.7%	1.3%	3.4%	1.3%	0.0%	9.6%	7.8%	8.2%	0.0%
0.0%	0.0%	0.0%	0.0%	0.0%	0.0%	0.0%	0.0%	0.0%	0.0%	0.0%	0.0%	0.0%	0.0%	0.0%
0.0%	0.0%	0.0%	0.0%	2.7%	4.8%	0.0%	0.0%	3.6%	0.0%	0.0%	0.0%	0.0%	0.0%	0.0%
0.0%	4.3%	0.0%	0.0%	2.7%	4.8%	0.0%	0.0%	5.1%	0.0%	0.0%	11.1%	5.9%	7.4%	0.0%
9.1%	0.0%	0.0%	0.0%	0.0%	0.0%	0.0%	0.0%	0.0%	0.0%	0.0%	0.0%	0.0%	0.0%	0.0%
0.0%	0.0%	0.0%	0.0%	4.3%	5.3%	0.0%	0.0%	3.4%	1.4%	0.0%	0.0%	0.0%	0.0%	0.0%
0.0%	0.0%	0.0%	0.0%	3.4%	0.0%	0.0%	0.0%	0.0%	0.0%	0.0%	0.0%	0.0%	0.0%	0.0%

For peer review only

[redacted]

For peer review only

3195 Shaw 2009	2520 Teahan 2020	4562 GonzalezFraile 2021	3528 Amador-Marin 2017	5049 Sin 2018	4806 Parkinson 2019	5174 Bennett 2019	5810 Lucero 2019	4421 Treanor 2019	5244 Goeman 2016
0.0%									
0.0%	4.7%								
0.0%	9.7%	2.9%							
0.0%	0.0%	9.1%	1.7%						
0.0%	0.0%	0.0%	0.0%	0.0%					
0.0%	3.0%	0.0%	0.0%	0.0%	0.0%				
0.0%	3.0%	25.0%	4.3%	3.4%	0.0%	0.0%			
0.0%	0.0%	0.0%	0.0%	0.0%	0.0%	0.0%	0.0%		
0.0%	1.5%	1.4%	1.7%	2.1%	0.0%	1.7%	3.5%	0.0%	
0.0%	0.0%	0.0%	0.0%	0.0%	0.0%	0.0%	0.0%	0.0%	2.0%

For peer review only

For peer review only

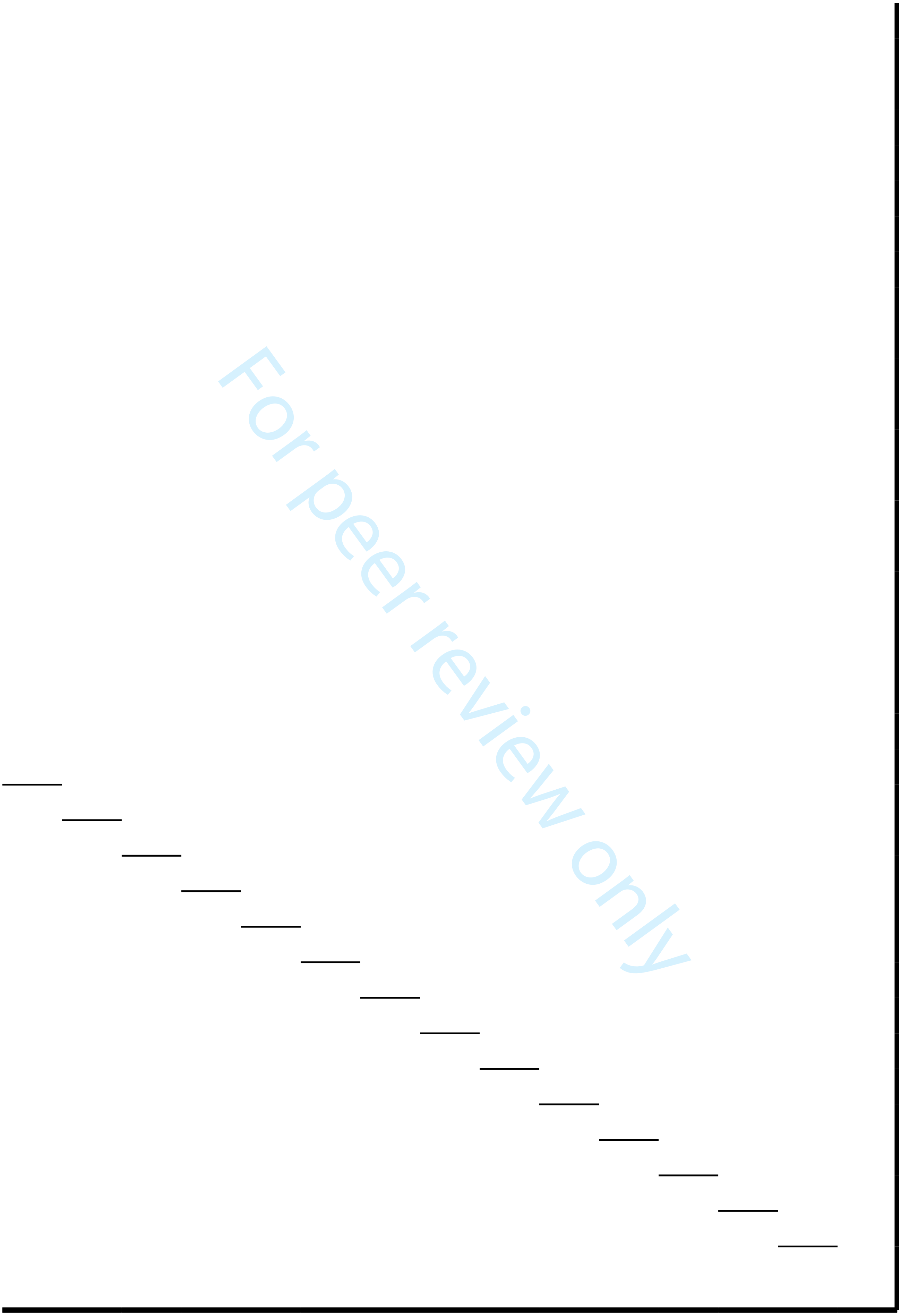

For peer review only

Graphical Representation of Overlap for OVErviews (G

	807 Irons 2	1048 Gabriel 202	2195 Shim 2020	2367 Hopwood 2018	2370 Guay 2017	2491 Vandepitte 2016	2524 Zabihi 2020	3197 Mason 2007
1048 Gabriel 2020	0.0%							
2195 Shim 2020	0.0%	0.0%						
2367 Hopwood 2018	0.0%	0.0%	0.0%					
2370 Guay 2017	0.0%	0.0%	0.0%	7.5%				
2491 Vandepitte 2016	0.0%	0.0%	0.0%	0.0%	0.0%			
2524 Zabihi 2020	0.0%	0.0%	0.0%	0.0%	0.0%	1.5%		
3197 Mason 2007	0.0%	0.0%	0.0%	0.0%	0.0%	7.5%	0.0%	
5257 Wiegelmann 2021	0.0%	0.0%	2.0%	1.3%	2.1%	14.3%	5.6%	0.0%
3552 Akarsu 2019	0.0%	0.0%	0.0%	0.0%	0.0%	1.6%	13.6%	0.0%
3734 Fu 2017	0.0%	22.2%	0.0%	0.0%	3.0%	0.0%	0.0%	0.0%
3939 Jensen 2015	0.0%	0.0%	0.0%	0.0%	0.0%	9.4%	0.0%	0.0%
3965 Corvol 2017	0.0%	0.0%	0.0%	0.0%	0.0%	1.5%	0.0%	0.0%
3970 Hopkinson 2019	0.0%	0.0%	0.0%	0.0%	0.0%	4.0%	11.8%	3.2%
4267 Pucciarelli 2020	0.0%	0.0%	0.0%	0.0%	7.7%	0.0%	0.0%	0.0%
4415 Vernooij-Dassen 2011	0.0%	0.0%	0.0%	5.3%	7.1%	0.0%	13.3%	0.0%
4452 Reilly 2015	0.0%	0.0%	0.0%	0.0%	0.0%	3.2%	0.0%	0.0%
4530 Liu 2018	0.0%	0.0%	18.2%	0.0%	0.0%	0.0%	0.0%	0.0%
4531 Lins 2014	0.0%	0.0%	0.0%	0.0%	0.0%	5.3%	0.0%	0.0%
4534 Legg 2011	0.0%	0.0%	0.0%	0.0%	0.0%	0.0%	0.0%	0.0%
4567 Forster 2012	0.0%	0.0%	0.0%	3.3%	0.0%	0.0%	0.0%	0.0%
4588 Corry 2019	0.0%	3.1%	0.0%	0.0%	0.0%	4.2%	6.3%	0.0%
5731 Piersol 2017	0.0%	0.0%	2.1%	2.8%	0.0%	9.2%	1.9%	0.0%
2104 Cuthbert 2017	0.0%	0.0%	0.0%	0.0%	0.0%	1.6%	0.0%	0.0%
3453 Collins 2019	0.0%	0.0%	11.1%	0.0%	0.0%	0.0%	0.0%	0.0%
615 Waldron 2013	0.0%	0.0%	0.0%	0.0%	0.0%	0.0%	0.0%	0.0%
3544 Boots 2014	0.0%	0.0%	0.0%	38.9%	5.0%	1.7%	0.0%	0.0%
3984 Doyle 2020	0.0%	0.0%	0.0%	0.0%	0.0%	0.0%	0.0%	0.0%
4285 Maffioletti 2019	0.0%	0.0%	0.0%	0.0%	0.0%	6.3%	0.0%	7.4%
2570 Lee 2020	0.0%	0.0%	3.0%	1.6%	2.9%	13.8%	0.0%	0.0%
2809 Weinbrecht 2016	0.0%	0.0%	0.0%	3.7%	4.8%	14.9%	5.0%	0.0%
3014 Wang 2020	0.0%	0.0%	0.0%	7.5%	18.8%	1.7%	5.3%	0.0%
152 Minshall 2019	0.0%	0.0%	0.0%	0.0%	2.6%	0.0%	0.0%	0.0%
588 Cheng 2020	0.0%	0.0%	4.4%	2.5%	2.4%	16.7%	5.0%	0.0%

For peer review only

1611 Smith 2019	0.0%	0.0%	0.0%	0.0%	5.4%	0.0%	0.0%	0.0%
248 Heckel 2019	0.0%	0.0%	0.0%	0.0%	0.0%	0.0%	0.0%	0.0%
1596 Egan 2018	0.0%	0.0%	0.0%	20.0%	17.6%	0.0%	0.0%	0.0%
1977 Zhao 2019	0.0%	0.0%	0.0%	13.5%	28.6%	0.0%	0.0%	0.0%
1992 Leng 2020	0.0%	0.0%	0.0%	24.3%	23.5%	0.0%	0.0%	0.0%
XT Maayan 2014	0.0%	0.0%	0.0%	0.0%	0.0%	1.9%	0.0%	13.6%
3195 Shaw 2009	0.0%	0.0%	0.0%	0.0%	0.0%	6.3%	0.0%	21.7%
2520 Teahan 2020	0.0%	0.0%	0.0%	0.0%	0.0%	10.3%	2.9%	0.0%
4562 GonzalezFraile 2021	0.0%	0.0%	3.4%	12.2%	10.7%	4.4%	2.9%	0.0%
3528 Amador-Marin 2017	0.0%	0.0%	0.0%	2.4%	0.0%	8.3%	0.0%	0.0%
5049 Sin 2018	0.0%	1.7%	0.0%	22.1%	13.0%	1.0%	0.0%	0.0%
4806 Parkinson 2019	0.0%	0.0%	0.0%	0.0%	0.0%	0.0%	0.0%	0.0%
5174 Bennett 2019	0.0%	0.0%	0.0%	0.0%	0.0%	8.6%	0.0%	0.0%
5810 Lucero 2019	0.0%	0.0%	0.0%	4.3%	0.0%	4.8%	0.0%	0.0%
4421 Treanor 2019	0.0%	0.0%	0.0%	0.0%	0.0%	0.0%	0.0%	0.0%
5244 Goeman 2016	0.0%	0.0%	0.0%	0.0%	0.0%	7.5%	0.0%	0.0%
5375 Greenwood 2016	0.0%	0.0%	0.0%	0.0%	0.0%	1.8%	0.0%	0.0%

For peer review only

ROOVE) - Considering Structural Missingness

1275	=	**Total nodes (pairs of reviews)**
1107	=	Slight overlap (<5%)
87	=	Moderate overlap (5% to <10%)
42	=	High overlap (10% to <15%)
39	=	Very High overlap (>15%)

5257 Wiegelmann 2021	3552 Akarsu 2019	3734 Fu 2017	3939 Jensen 2015	3965 Corvol 2017	3970 Hopkinson 2019	4267 Pucciarelli 2020	4415 Vernooij-Dassen 2011	4452 Reilly 2015	4530 Liu 2018	4531 Lins 2014	4534 Legg 2011	4567 Forster 2012	4588 Corry 2019
1.8%													
0.0%	0.0%												
25.0%	0.0%	0.0%											
0.0%	0.0%	0.0%	0.0%										
6.2%	8.8%	0.0%	0.0%	0.0%									
0.0%	0.0%	0.0%	0.0%	0.0%	0.0%								
0.0%	5.9%	0.0%	0.0%	0.0%	26.3%	0.0%							
0.0%	0.0%	0.0%	0.0%	31.8%	0.0%	0.0%	0.0%						
0.0%	0.0%	0.0%	0.0%	0.0%	0.0%	0.0%	0.0%	0.0%					
0.0%	5.3%	0.0%	0.0%	0.0%	0.0%	0.0%	5.3%	0.0%	0.0%				
0.0%	0.0%	0.0%	0.0%	0.0%	0.0%	0.0%	0.0%	0.0%	0.0%	0.0%			
0.0%	0.0%	0.0%	0.0%	0.0%	0.0%	0.0%	0.0%	0.0%	0.0%	0.0%	11.5%		
3.2%	10.0%	2.4%	0.0%	0.0%	4.5%	2.8%	6.3%	0.0%	0.0%	8.0%	0.0%	0.0%	
8.6%	1.9%	0.0%	0.0%	7.4%	1.5%	0.0%	0.0%	8.0%	4.4%	0.0%	0.0%	0.0%	5.0%
2.4%	0.0%	0.0%	0.0%	0.0%	0.0%	0.0%	0.0%	0.0%	0.0%	0.0%	0.0%	0.0%	3.4%
1.8%	0.0%	0.0%	0.0%	0.0%	2.8%	0.0%	0.0%	0.0%	30.8%	0.0%	0.0%	0.0%	0.0%
0.0%	0.0%	0.0%	0.0%	0.0%	0.0%	0.0%	0.0%	0.0%	0.0%	0.0%	0.0%	0.0%	0.0%
0.0%	0.0%	0.0%	0.0%	0.0%	0.0%	0.0%	5.6%	0.0%	0.0%	0.0%	0.0%	3.2%	0.0%
0.0%	0.0%	0.0%	0.0%	0.0%	0.0%	0.0%	0.0%	0.0%	0.0%	0.0%	0.0%	0.0%	0.0%
1.8%	0.0%	0.0%	0.0%	0.0%	0.0%	0.0%	0.0%	0.0%	0.0%	0.0%	0.0%	0.0%	2.9%
12.9%	0.0%	0.0%	9.1%	8.3%	2.0%	0.0%	0.0%	11.1%	0.0%	0.0%	0.0%	0.0%	0.0%
18.4%	10.0%	0.0%	8.6%	6.7%	5.9%	0.0%	4.0%	10.3%	0.0%	2.6%	0.0%	0.0%	2.1%
4.1%	5.6%	0.0%	0.0%	0.0%	0.0%	0.0%	7.1%	0.0%	0.0%	6.7%	0.0%	0.0%	0.0%
0.0%	0.0%	0.0%	0.0%	0.0%	0.0%	12.5%	0.0%	0.0%	0.0%	0.0%	19.0%	11.4%	2.0%
23.8%	5.1%	0.0%	7.1%	4.0%	6.8%	0.0%	4.3%	6.7%	2.3%	7.3%	0.0%	0.0%	4.1%

For peer review only

0.0%	0.0%	0.0%	0.0%	0.0%	0.0%	22.2%	0.0%	0.0%	0.0%	0.0%	22.2%	9.1%	4.3%
0.0%	0.0%	0.0%	0.0%	0.0%	0.0%	0.0%	0.0%	0.0%	0.0%	2.4%	0.0%	0.0%	0.0%
2.1%	0.0%	0.0%	0.0%	0.0%	0.0%	0.0%	7.7%	0.0%	0.0%	0.0%	0.0%	0.0%	0.0%
2.0%	0.0%	0.0%	0.0%	0.0%	0.0%	0.0%	9.1%	0.0%	0.0%	0.0%	0.0%	0.0%	0.0%
1.7%	0.0%	0.0%	0.0%	0.0%	0.0%	0.0%	8.3%	0.0%	0.0%	0.0%	0.0%	0.0%	0.0%
0.0%	0.0%	0.0%	0.0%	0.0%	0.0%	0.0%	0.0%	0.0%	0.0%	0.0%	0.0%	0.0%	0.0%
0.0%	0.0%	0.0%	0.0%	0.0%	2.2%	0.0%	0.0%	0.0%	0.0%	0.0%	0.0%	0.0%	0.0%
24.5%	0.0%	0.0%	14.3%	2.7%	2.2%	0.0%	0.0%	7.1%	3.8%	0.0%	0.0%	0.0%	2.4%
8.1%	6.7%	0.0%	0.0%	0.0%	0.0%	0.0%	0.0%	0.0%	0.0%	21.1%	0.0%	0.0%	10.8%
9.5%	0.0%	0.0%	6.3%	3.7%	5.7%	0.0%	0.0%	4.5%	0.0%	0.0%	0.0%	0.0%	0.0%
1.1%	1.7%	1.4%	0.0%	0.0%	0.0%	1.6%	0.0%	0.0%	0.0%	2.6%	0.0%	0.0%	0.0%
0.0%	0.0%	0.0%	0.0%	0.0%	0.0%	0.0%	0.0%	0.0%	0.0%	0.0%	0.0%	0.0%	0.0%
3.7%	0.0%	0.0%	0.0%	4.0%	0.0%	0.0%	0.0%	4.5%	0.0%	0.0%	0.0%	0.0%	0.0%
3.7%	0.0%	0.0%	0.0%	0.0%	0.0%	0.0%	5.3%	4.3%	0.0%	15.8%	0.0%	0.0%	10.0%
0.0%	0.0%	2.6%	0.0%	0.0%	0.0%	0.0%	0.0%	0.0%	0.0%	0.0%	0.0%	0.0%	0.0%
1.4%	1.7%	0.0%	0.0%	14.5%	1.4%	0.0%	2.2%	15.4%	0.0%	0.0%	0.0%	0.0%	1.5%
0.0%	0.0%	0.0%	0.0%	0.0%	0.0%	0.0%	0.0%	0.0%	0.0%	0.0%	0.0%	0.0%	0.0%

For peer review only

[redacted]

For peer review only

5731 Piersol 2017	2104 Cuthbert 2017	3453 Collins 2019	615 Waldron 2013	3544 Boots 2014	3984 Doyle 2020	4285 Maffioletti 2019	2570 Lee 2020	2809 Weinbrecht 2016	3014 Wang 2020	152 Minshall 2019	588 Cheng 2020	Smith 2019	2019
2.0%													
5.9%	0.0%												
0.0%	0.0%	0.0%											
3.9%	0.0%	0.0%	0.0%										
0.0%	11.1%	0.0%	0.0%	0.0%									
0.0%	0.0%	0.0%	0.0%	0.0%	0.0%								
12.1%	0.0%	0.0%	0.0%	0.0%	0.0%	0.0%							
10.4%	0.0%	0.0%	0.0%	2.5%	0.0%	0.0%	20.5%						
0.0%	0.0%	0.0%	0.0%	6.3%	0.0%	0.0%	0.0%	2.7%					
0.0%	0.0%	0.0%	0.0%	0.0%	0.0%	0.0%	0.0%	0.0%	0.0%				
11.5%	2.5%	2.8%	0.0%	1.1%	0.0%	0.7%	13.6%	24.8%	2.2%	0.0%			

0.0%	0.0%	0.0%	0.0%	0.0%	0.0%	0.0%	0.0%	0.0%	0.0%	14.0%	0.0%	1611 :	248 Heckel
0.0%	0.0%	0.0%	0.0%	0.0%	0.0%	0.0%	0.0%	0.0%	0.0%	0.0%	0.0%	0.0%	
2.0%	0.0%	0.0%	0.0%	20.0%	0.0%	0.0%	3.0%	5.3%	25.0%	0.0%	2.2%	0.0%	0.0%
2.1%	0.0%	0.0%	0.0%	7.1%	0.0%	0.0%	6.7%	8.6%	30.0%	0.0%	3.0%	0.0%	0.0%
2.0%	0.0%	0.0%	0.0%	14.3%	0.0%	0.0%	4.9%	8.1%	14.3%	0.0%	4.9%	0.0%	0.0%
0.0%	0.0%	0.0%	0.0%	0.0%	0.0%	0.0%	5.3%	0.0%	0.0%	0.0%	1.3%	0.0%	0.0%
0.0%	0.0%	0.0%	0.0%	0.0%	0.0%	10.3%	0.0%	0.0%	0.0%	0.0%	0.0%	0.0%	0.0%
14.3%	0.0%	3.0%	0.0%	0.0%	0.0%	2.9%	17.1%	12.5%	0.0%	0.0%	14.8%	0.0%	0.0%
1.7%	0.0%	0.0%	0.0%	4.3%	0.0%	3.0%	4.3%	8.7%	7.4%	0.0%	10.7%	0.0%	1.7%
10.2%	0.0%	0.0%	0.0%	0.0%	0.0%	0.0%	25.0%	12.8%	0.0%	0.0%	7.8%	0.0%	0.0%
2.2%	0.0%	0.0%	0.0%	8.1%	0.0%	0.0%	2.7%	2.7%	3.7%	1.3%	3.5%	1.3%	0.0%
0.0%	0.0%	0.0%	0.0%	0.0%	0.0%	0.0%	0.0%	0.0%	0.0%	0.0%	0.0%	0.0%	0.0%
4.0%	0.0%	0.0%	0.0%	0.0%	0.0%	0.0%	2.7%	5.0%	0.0%	0.0%	3.6%	0.0%	0.0%
5.9%	5.0%	0.0%	0.0%	4.5%	0.0%	0.0%	2.7%	4.8%	0.0%	0.0%	5.1%	0.0%	0.0%
0.0%	0.0%	0.0%	11.1%	0.0%	0.0%	0.0%	0.0%	0.0%	0.0%	0.0%	0.0%	0.0%	0.0%
4.7%	0.0%	0.0%	0.0%	0.0%	0.0%	0.0%	4.6%	5.3%	0.0%	0.0%	4.2%	1.4%	0.0%
0.0%	0.0%	0.0%	0.0%	0.0%	0.0%	0.0%	4.2%	0.0%	0.0%	0.0%	0.0%	0.0%	0.0%

For peer review only

[redacted]

For peer review only

1596 Egan 2018	1977 Zhao 2019	1992 Leng 2020	XT Maayan 2014	3195 Shaw 2009	2520 Teahan 2020	4562 GonzalezFraile 2021	3528 Amador-Marin 2017	5049 Sin 2018	4806 Parkinson 2019	5174 Bennett 2019	5810 Lucero 2019	4421 Treanor 2019	5244 Goeman 2016
55.6%													
50.0%	50.0%												
0.0%	0.0%	0.0%											
0.0%	0.0%	0.0%	5.7%										
0.0%	0.0%	0.0%	5.0%	0.0%									
22.7%	23.8%	34.5%	0.0%	0.0%	4.8%								
5.3%	5.9%	5.0%	0.0%	0.0%	9.7%	3.3%							
9.6%	7.8%	9.3%	0.0%	0.0%	0.0%	9.7%	1.7%						
0.0%	0.0%	0.0%	0.0%	0.0%	0.0%	0.0%	0.0%	0.0%					
0.0%	0.0%	0.0%	0.0%	0.0%	3.0%	0.0%	0.0%	0.0%	0.0%				
11.1%	5.9%	9.1%	0.0%	0.0%	3.0%	28.0%	4.3%	3.4%	0.0%	0.0%			
0.0%	0.0%	0.0%	0.0%	0.0%	0.0%	0.0%	0.0%	0.0%	0.0%	0.0%	0.0%		
0.0%	0.0%	0.0%	0.0%	0.0%	1.5%	1.6%	1.7%	2.2%	0.0%	1.8%	3.5%	0.0%	
0.0%	0.0%	0.0%	0.0%	0.0%	0.0%	0.0%	0.0%	0.0%	0.0%	0.0%	0.0%	0.0%	2.0%

For peer review only

[redacted]

For peer review only

For peer review only

For peer review only

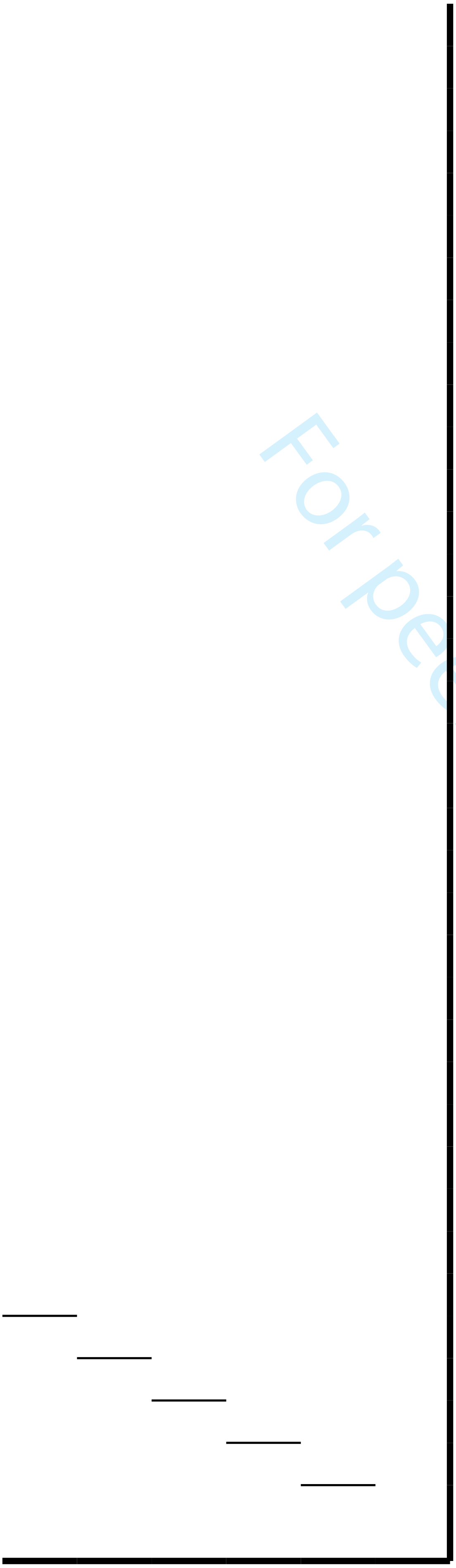

For peer review only

GROOVE tool: Graphica

DOI: 10.17605/OSF.IO/U2MS4

What is GROOVE?

GROOVE (**G**raphical **R**epresentation of **O**verlap for **OVE**rviews) is a tool that provides a visual display o

Starting with a simple matrix of evidence, this tool will automatically inform:
- The corrected covered area for the whole matrix.
- The number of unique primary studies.
- The number of overlapped primary studies (and in how many reviews they are included).
- A detailed assessment of the corrected covered area between every possible pair of reviews (nodes),
- The number of nodes with low, moderate, high and very high overlap.
- Optionally, an assessment of the corrected covered area corrected by structural missingness in the m

This tool is based on a previously published approach. If you are not familiar with the quantitative asse
Neugebauer EAM, Eikermann M. Systematic review finds overlapping reviews were not mentioned in e

How to use this tool:

You only have to enter data on the "1. Matrix" sheet. The matrix supports up to 70 systematic reviews
You will get all the results on the "GROOVE" sheets (colour or greyscale version).

1. Go to the "1. Matrix" sheet.
2. Write all the systematic reviews ID in the columns, starting in the C5 cell.
3. Write all the primary studies ID in the rows, starting in the A6 cell.
4. Optionally, you can write the topic of your overview in the B2 cell, and the references of each prima
5. Mark with a "1" the intersections representing primary studies included in their respective systema
6. Go to the "2.1 GROOVE - colour version" or "2.2 GROOVE - grayscale version" tab. You will find the c
moderate, high and very high overlap, and the number of unique primary studies and overlapped prim

Advanced usage: Incorporating structural missingness

While you are building the matrix of evidence, you will notice that it should be impossible to mark a "1
published in 2015 can not be included in a systematic review published in 2013; this would be a "chro

The GROOVE tool provides the option to incorporate structural missingness into the results. In order t
1. Follow all the steps mentioned in the previous section.
2. When you finish populating the matrix of evidence with "1"s, you can mark the cells with structural
3. Once you finish, go to the "2.1 GROOVE - colour version" or "2.2 GROOVE - grayscale version" tab. Y
4. The Graphical Representation of Overlap for OVErviews incorporating structural missingness will be
structural missingness in the matrix, this figure will be the same as the one displayed in F1.

Example: How to enter data into the matrix

MATRIX OF EVIDENCE

TOPIC:

		Systematic [illegible]		
**Primary Studies**				
**Study ID**	**Reference**	**Bonfill 2012**	**Urrútia 2014**	[illegible]
Bonham 1980		1		
Lennon 1980		1	1	[illegible]
Mercury 1991		1		
Harrison 2001			1	[illegible]
Jones 2007			1	
McCartney 2010			1	[illegible]
Taylor 2011				[illegible]
Page 2012				[illegible]
Deacon 2013		X		[illegible]
May 2013		X	1	[illegible]
Starr 2019		X	X	[illegible]
Plant 2020		X	X	[illegible]

2. Write all the primary studies ID here (references are optional)

Overall results

Number of columns (number of reviews)	c	4
Number of rows (number of index publications)	r	12
Number of included primary studies (including double counting)	N	20
Covered area	N/(rc)	41,67%
Corrected covered area	(N-r)/(rc-r)	22,22%
Interpretation of overlap	**Very High overlap**	
Structural Zeros	X	8
Corrected covered area (adjusting by structural zeros)	(N-r)/(rc-r-X)	28,57%

N° of non-overlapped primary studies	In 1 SR	7
Number of overlapped primary studies	In 2 SRs	2
	In 3 SRs	3
	In 4 SRs	0
	In 5 SRs	0
	In 6 SRs	0
	In 7 SRs	0
	In 8 SRs	0
	In 9 SRs	0
	In 10 SRs	0
	In 11 SRs	0
	In 12 SRs	0
	In 13 SRs	0
	In 14 SRs	0
	In 15 or more SRs	0

5. Go to the "2.1 GROO[illegible]
- grayscale version" she[illegible]

Graphical Representat[illegible]

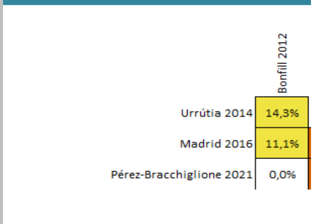

Graphical Representa[illegible]

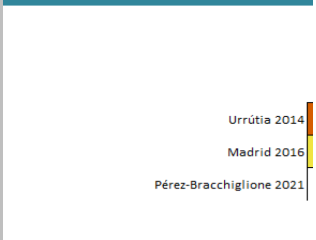

For peer review only

al Representation of Overla

[redacted]

of the overlap of primary studies among systematic reviews

, and a graphical representation of this assessment.

natrix.

essment of overlap using the corrected covered area formul
every other overview. Journal of Clinical Epidemiology2014;

and 1000 unique primary studies.

ary study in the B column, starting with B6.
tic reviews. It is expected for a single primary study to be ir
corrected covered area (CCA) for the whole matrix, the CCA
nary studies.

1" in some cells. A cell that can not acquire other value thar
nological structural missingness".

to do this, you will have to:

missingness with an "X". You will notice that the cell will tu
You will notice that the D9 cell will provide the CCA formula
displayed in the "2.1 GROOVE - colour version" and "2.2 G

[redacted]

view only

Reviews

Madrid 2016	Pérez-Bracchiglione 2021
1	
1	1
1	1
1	
1	1
1	
1	
X	1
X	1

1. Write all the systematic reviews ID here

3. Mark with a "1" each intersection representing a primary study included in a systematic review

4. If you want to consider structural missingne the calculation of CCA, you may mark with an those cells representing structural missingnes This is an optional feature.

VE - colour version" or "2.2 GROOVE eet. Your results will be ready.

tion of Overlap for OVErviews (GROOVE)

	Urrútia 2014	Madrid 2016
	50,0%	
	25,0%	33,3%

6	=	Total nodes (pairs of rev
1	=	Slight overlap (<5%)
0	=	Moderate overlap (5% t
2	=	High overlap (10% to <1
3	=	Very High overlap (≥15%

ation of Overlap for OVErviews (GROOVE) - Cons

Bonfill 2012	Urrútia 2014	Madrid 2016
16,7%		
14,3%	50,0%	
0,0%	33,3%	42,9%

6	=	Total no
1	=	Slight ov
0	=	Moderat
1	=	High ove
4	=	Very Hig

Contact: javier.perez.b@gmail.com

view only

Universidad
de Valparaíso
CHILE

U[illegible]

For peer review only

ap for OVErviews

s.

la, we suggest you to read the original publication: Pieper D, Antoine S-L, Mathes T,
:67(4):368–75.

ncluded in multiple systematic reviews.
A for each pair (or nodes) of systematic reviews, the number of nodes with low,

n 0 is called "structural missingness" or "structural zero". For example, a primary study

urn black.
a corrected by structural missingness.
GROOVE - grayscale version" tabs, starting in the cell CA1. If you do not mark any

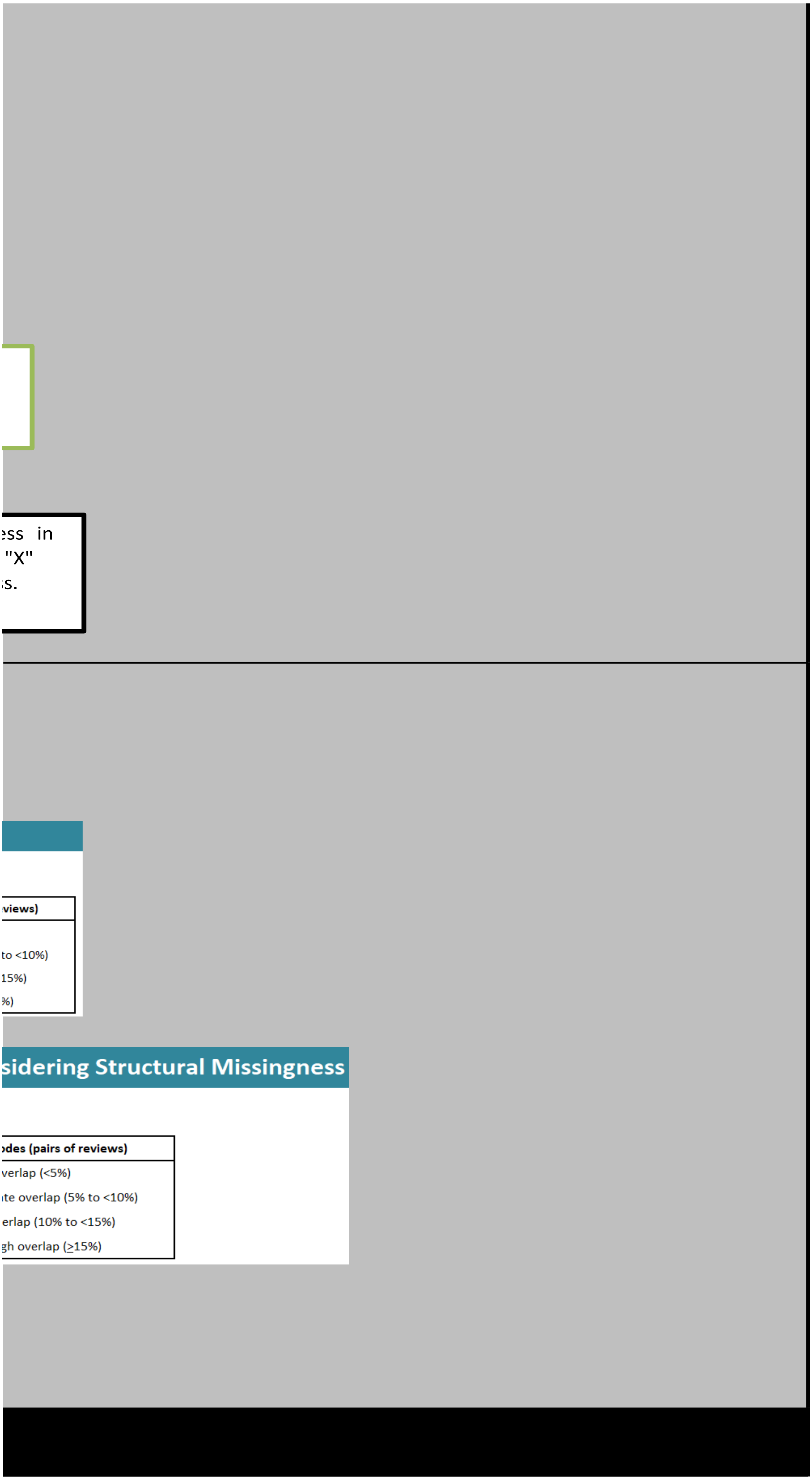
ess in
"X"
s.
views)
to <10%)
15%)
%)
sidering Structural Missingness
odes (pairs of reviews)
verlap (<5%)
te overlap (5% to <10%)
erlap (10% to <15%)
gh overlap (≥15%)

UAB
niversitat Autònoma
de Barcelona

CIESAL
CENTRO INTERDISCIPLINARIO
DE ESTUDIOS EN SALUD
UNIVERSIDAD DE VALPARAÍSO

For peer review only

MATRIX OF EVIDENCE

TOPIC: Informal caregiving UR

Primary Studies		Systematic Reviews								
**Study ID**	**Reference**	**5244 Goeman 20**	**3366 Bunn 2016**	**3544 Boots 2014**	**4285 Maffioletti 2**	**4531 Lins 2014**	**5481 Pritchard 20**	**3368 Bourne 202**	**3718 DuPreez 20**	**807 Irons 2020**
BustonHope2005			1							
Hibberd2011			1							
Quinn2009			1							
WoodsAlgar2009			1							
Ducharme2011				1						
Kelly2004				1						
Torp2008				1						
Chiu2009				1						
Rokstad2017		X	X	X	1	X				
Tretteteig2017		X	X	X	1	X				
Liu2015				X	1	X				
Zarit2014				X	1	X				
Gustafsdottir2014				X	1	X			1	
Philipson2012					1					
deJong2009					1					
Chang2004						1				
Salfi2005						1				
Acharya2015				X		X	1			
Bauer2011							1			
DouglasDunbar2007				X		X	1			
Jamieson2014				X		X	1			
Mockford2017		X	X	X		X	1			
Moyle2016		X	X				1			
Naylor2005							1			
Camic2014				X		X		1		1
Eekelaar2012								1		
McGuigan2015				X		X		1		
Flatt2015				X		X		1		1
Camic2016		X	X	X		X		1		
Phinney2011									1	
Donath2011									1	
Gill2011									1	
Lloyd2011									1	
Robinson2012									1	
Gaugler2014				X		X			1	
Stirling2014				X		X			1	
Hynes2016				X		X			1	
Logsdon2016		X	X	X		X			1	
Unadkat2017		X	X			X				1
Osman2016		X	X			X				1

Hunt2018						X				1
Dassa2018						X				1
Clark2018						X				1
Burnside2015						X				1
Baker2017		X	X			X				1
Stockle2016		X	X			X				
Kovaleva2017		X	X			X				
Marziali2018		X	X			X				
Marziali2006										
Span2015						X				
Stewart2006										
Ledderer2013										
Vandenhurk2015						X				
Wittenberg2017		X	X			X				
Bamford2000										
Bowes2003										
Butterworth1995										
Gillies2000										
Innes2005										
Koffman1997										
Pearson1988										
Pickard2001										
Upton2005										
Watkins1997										
Wenger2002										
Ashworth2000										
Ashworth1996										
Hanson1999										
Heenan2000										
Jarrett1999										
Kerr2001										
Lane2003										
Low2004										
Low2005										
McGarry2001										
McLean1991										
Netto1998										
Skilbeck2005										
Ward1997										
Beisecker1996										
Brody1989										
Cotrell1996										
Dziegielewski2000										
Farran2003										
King1995										
Lampley2001										
Liken1995										
MaloneBeach1992										
Martin-Cook2000										
McGrath2000										
Tietelman2004										
Winslow2003										
Wykle1991										
Bedini1996										

For peer review only

Caserta1987										
Hennessy1996										
Lewis1995										
Piercy2004										
Valadez2005										
Worcester1997										
Morgan2002										
Perry2001										
Strang1998										
Strang2000										
Strang2001										
Wuest2001										
Michallet2001										
Ritchie2003										
Strang2002										
Wiles2003										
Gilmour2002										
Leong2001										
King2005										
Kristjanson2004										
Witt2004										
Yamamoto1998										
Young2002										
Ericson2001										
Jansson1998										
Bergs2002										
Berthold1991										
Johansson1991										
Hawranick2007										
Adams2006										
Scharlach2006										
Zepart2007										
Salin2007										
Mcllfatrick2006										
Gerdner2007										
Greenwood2013										
Cohen1998										
Diehl2003										
McKechnie2014				X		X				
Woods2014				X		X				
Sommerland2014				X		X				
Epstein-Lubow2011		1								
Hoppes2012		1								
Marconi2016		X	X	X		X				
Jain2014				X		X				
Kubo2018		X	X	X		X				
Cottingham2018		X	X	X		X				
Bazzano2015				X		X				
Hou2014				X		X				
Minor2006										
Vandenhurk2013										
Lliffe2014		1		X		X				
Verkade2010		1								
Minkman2009		1								

For peer review only - http://bmjopen.bmj.com/site/about/guidelines.xhtml

Rothera2008		1								
Boughtwood2011		1								
Burton2005		1								
Dewing2005		1								
Duane2013		1								
McGhee2010		1								
Quinn2013		1								
Blusi2013										
Damianakis2016		X		X		X				
Lichenstein2013										
Marziali2005										
Perron2002										
Torp2013										
Trondsen2014				X		X				
Smith2015				X		X				

For peer review only - http://bmjopen.bmj.com/site/about/guidelines.xhtml

iew only

view only

iew only

For peer review only - http://bmjopen.bmj.com/site/about/guidelines.xhtml

iew only

view only

iew only

view only

iew only

iew only

iew only

iew only

897 AlDaken 201	2367 Hopwood 2	6171 Smith 2014	5049 Sin 2018	3195 Shaw 2009	1459 Dam 2016	5843 Miles 2020	4806 Parkinson 2	5510 Li 2016											
				X															
				X															
				X															
				X															
				X															
		X		X	X			X											
		X		X	X			X											
		X		X															
		X		X															
		X		X															
				X															
				X															
		X		X															
				X															
		X		X															
		X		X	X			X											
		X		X	X			X											
		X		X															
				X															
		X		X															
		X		X															
		X		X	X			X											
				X															
				X															
				X															
				X															
				X															
		X		X															
		X		X															
		X		X															
		X		X	X			X											
		X		X	X			X											
		X		X	X			X											

For peer review only

		X		X															
		X		X															
		X		X															
		X		X															
		X		X	X			X											
1		X		X	X			X											
	1	X		X	X			X											
	1	X		X	X			X											
	1																		
	1	X		X															
		1																	
				X															
		X		X															
		X		X	X		1	X											
				1															
				1															
				1															
				1															
				1															
				1															
				1															
				1															
				1															
				1															
				1															
				1															
				1															
				1															
				1															
				1															
				1															
				1															
				1															
				1															
				1															
				1															
				1															
				1															
				1															
				1															
				1															
				1															
				1															
				1															
				1															
				1															
				1															
				1															
				1															
				1															
				1															
				1															
				1															
				1															

For peer review only

				1															
				1															
				1															
				1															
				1															
				1															
				1															
				1															
				1															
				1															
				1															
				1															
				1															
				1															
				1															
				1															
				1															
				1															
				1															
				1															
				1															
				1															
				1															
				1															
				1															
				1															
				1															
				1															
				1															
				1															
				1															
				1															
				1															
				1															
				1															
					1														
					1														
					1														
		X		X															
		X		X		1													
		X		X		1													
				X															
				X															
		X		X	X		1	X											
		X		X															
X	X	X	X	X	X			X											
X	X	X	X	X	X			X											
		X		X				1											
		X		X				1											
								1											
				X				1											
		X		X															
				X															
				X															

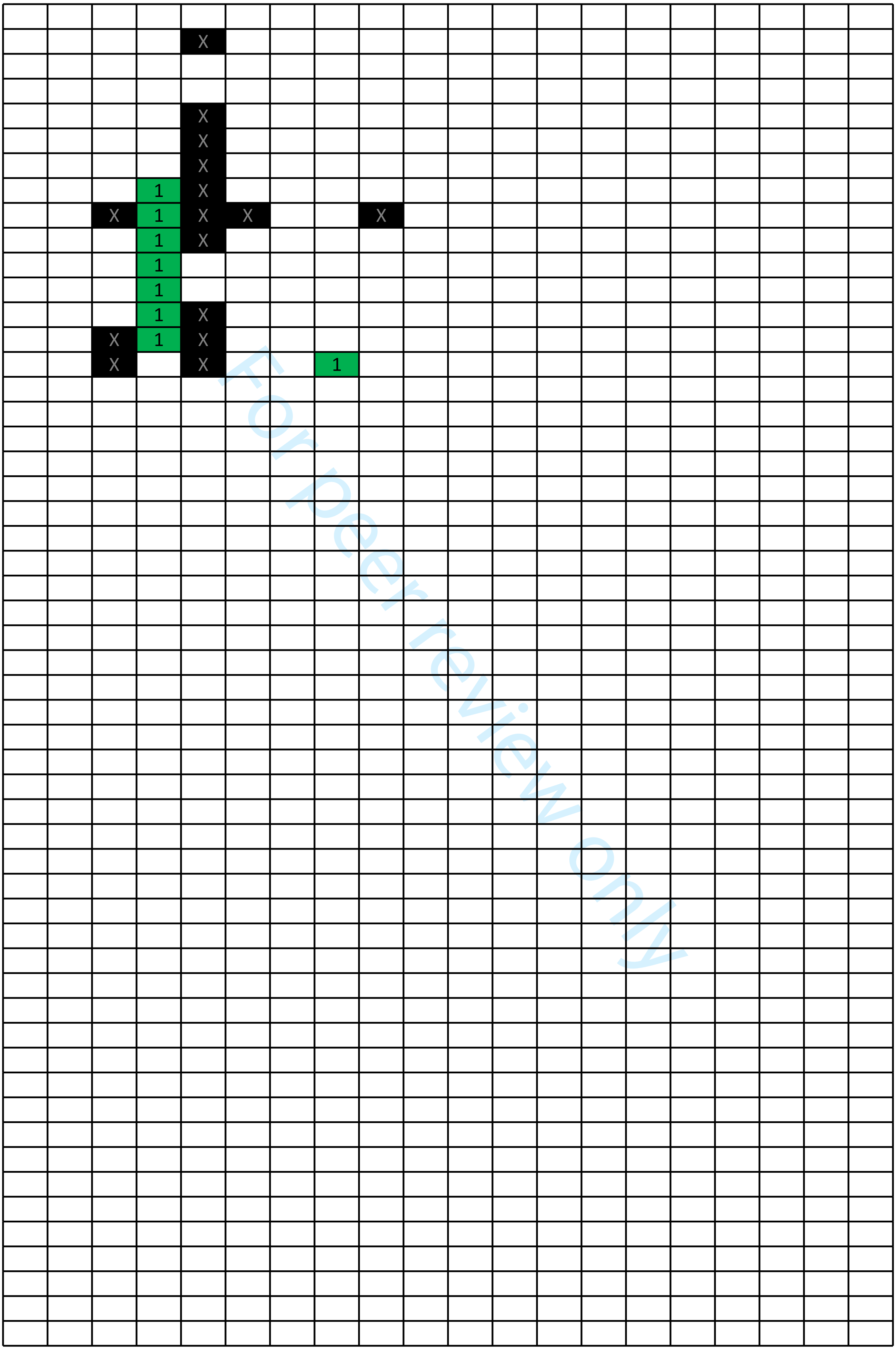

For peer review only

For peer review only

For peer review only

For peer review only

For peer review only

For peer review only

For peer review only

For peer review only

For peer review only

For peer review only

For peer review only

For peer review only

For peer review only

For peer review only

For peer review only

For peer review only

For peer review only

For peer review only

For peer review only

For peer review only

For peer review only

For peer review only

For peer review only

For peer review only

For peer review only

For peer review only

For peer review only

For peer review only

For peer review only

For peer review only

For peer review only

For peer review only

For peer review only

For peer review only

For peer review only

For peer review only

For peer review only

For peer review only

For peer review only

For peer review only

For peer review only

For peer review only

For peer review only

For peer review only

For peer review only

For peer review only

For peer review only

For peer review only

For peer review only

For peer review only

For peer review only

For peer review only

For peer review only

For peer review only

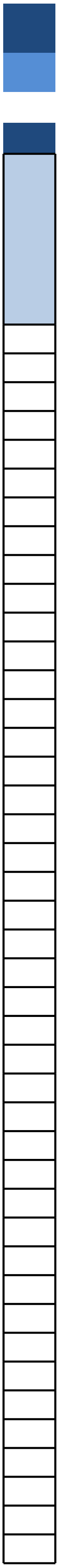

For peer review only

For peer review only

For peer review only

For peer review only

For peer review only

For peer review only

For peer review only

For peer review only

For peer review only

For peer review only

For peer review only

For peer review only

For peer review only

For peer review only

For peer review only

For peer review only

For peer review only

For peer review only

For peer review only

Overall results

Number of columns (number of reviews)	c	18
Number of rows (number of index publications)	r	163
Number of included primary studies (including double counting)	N	160
Covered area	N/(rc)	5.45%
Corrected covered area	(N-r)/(rc-r)	-0.11%
Interpretation of overlap	Slight overlap	
Structural Zeros	X	259
Corrected covered area (adjusting by structural zeros)	(N-r)/(rc-r-X)	-0.12%

N° of non-overlapped primary studies	In 1 SR	154
Number of overlapped primary studies	In 2 SRs	3
	In 3 SRs	0
	In 4 SRs	0
	In 5 SRs	0
	In 6 SRs	0
	In 7 SRs	0
	In 8 SRs	0
	In 9 SRs	0
	In 10 SRs	0
	In 11 SRs	0
	In 12 SRs	0
	In 13 SRs	0
	In 14 SRs	0
	In 15 or more SRs	0

G

For peer review only

iraphical Representation of Overlap for OVErviews (GROO

	5244 Goeman 20	3366 Bunn 2016	3544 Boots 2014	4285 Maffioletti 2019	4531 Lins 2014	5481 Pritchard 2020	3368 Bourne 2020	3718 DuPreez 2018	807 Irons 2020	897 AlDaken 2018
3366 Bunn 2016	0.0%									
3544 Boots 2014	0.0%	0.0%								
4285 Maffioletti 2019	0.0%	0.0%	0.0%							
4531 Lins 2014	0.0%	0.0%	0.0%	0.0%						
5481 Pritchard 2020	0.0%	0.0%	0.0%	0.0%	0.0%					
3368 Bourne 2020	0.0%	0.0%	0.0%	0.0%	0.0%	0.0%				
3718 DuPreez 2018	0.0%	0.0%	0.0%	6.3%	0.0%	0.0%	0.0%			
807 Irons 2020	0.0%	0.0%	0.0%	0.0%	0.0%	0.0%	16.7%	0.0%		
897 AlDaken 2018	0.0%	0.0%	0.0%	0.0%	0.0%	0.0%	0.0%	0.0%	0.0%	
2367 Hopwood 2018	0.0%	0.0%	0.0%	0.0%	0.0%	0.0%	0.0%	0.0%	0.0%	0.0%
6171 Smith 2014	0.0%	0.0%	0.0%	0.0%	0.0%	0.0%	0.0%	0.0%	0.0%	0.0%
5049 Sin 2018	0.0%	0.0%	0.0%	0.0%	0.0%	0.0%	0.0%	0.0%	0.0%	0.0%
3195 Shaw 2009	0.0%	0.0%	0.0%	0.0%	0.0%	0.0%	0.0%	0.0%	0.0%	0.0%
1459 Dam 2016	0.0%	0.0%	0.0%	0.0%	0.0%	0.0%	0.0%	0.0%	0.0%	0.0%
5843 Miles 2020	0.0%	0.0%	0.0%	0.0%	0.0%	0.0%	0.0%	0.0%	0.0%	0.0%
4806 Parkinson 2019	0.0%	0.0%	0.0%	0.0%	0.0%	0.0%	0.0%	0.0%	0.0%	0.0%
5510 Li 2016	0.0%	0.0%	0.0%	0.0%	0.0%	0.0%	0.0%	0.0%	0.0%	0.0%

153
151
1
0
1

For peer review only

For peer review only

OVE)

=	Total nodes (pairs of reviews)
=	Slight overlap (<5%)
=	Moderate overlap (5% to <10%)
=	High overlap (10% to <15%)
=	Very High overlap (>15%)

2367 Hopwood 2018	6171 Smith 2014	5049 Sin 2018	3195 Shaw 2009	1459 Dam 2016	5843 Miles 2020	4806 Parkinson 2019
0.0%						
0.0%	0.0%					
0.0%	0.0%	0.0%				
0.0%	0.0%	0.0%	0.0%			
0.0%	0.0%	0.0%	0.0%	0.0%		
0.0%	0.0%	0.0%	0.0%	0.0%	0.0%	
0.0%	0.0%	0.0%	0.0%	0.0%	0.0%	0.0%

For peer review only

For peer review only

For peer review only

For peer review only

For peer review only

For peer review only

For peer review only

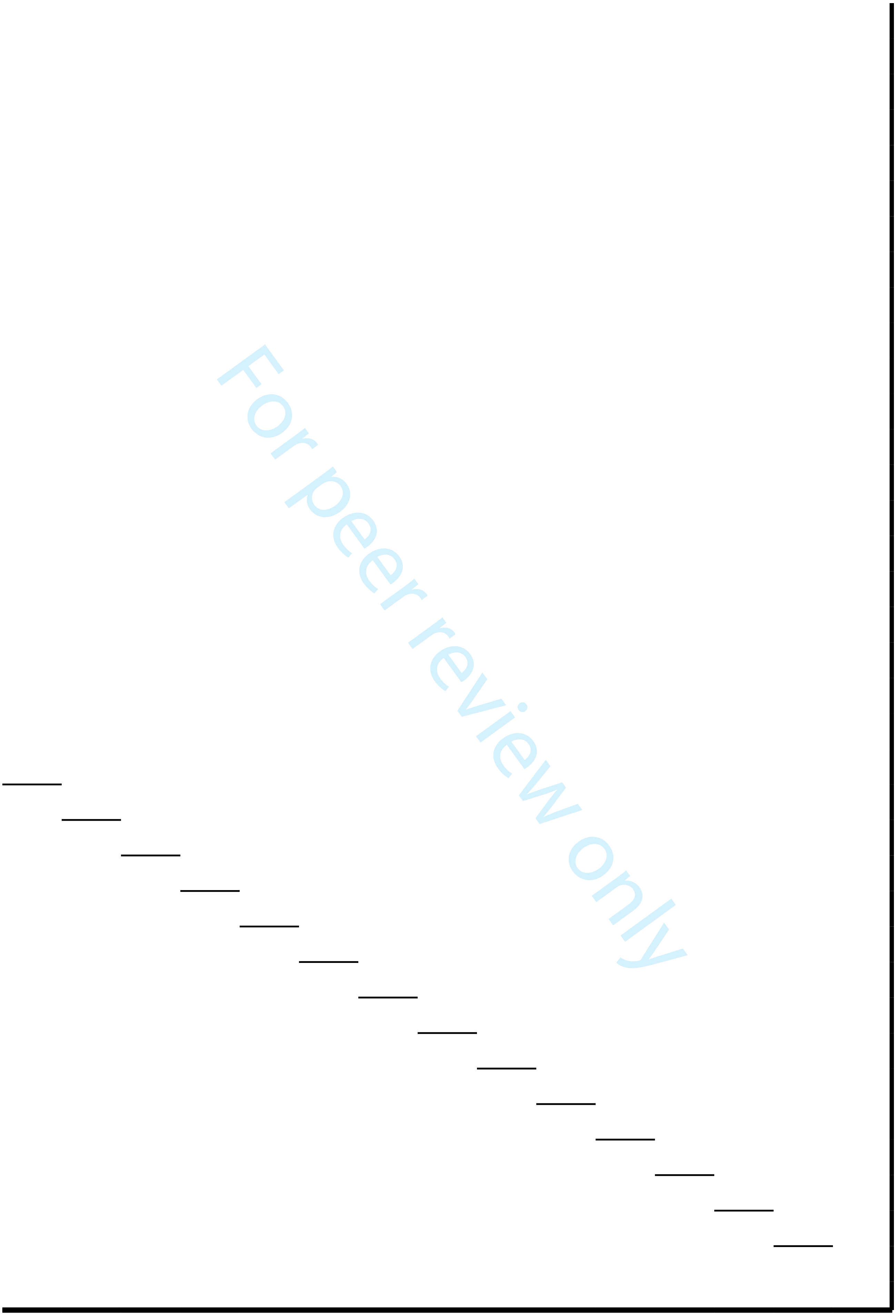

For peer review only

Graphical Representation of Overlap for OVErviews (G

	5244 Goem	3366 Bunn 2016	3544 Boots 2014	4285 Maffioletti 2019	4531 Lins 2014	5481 Pritchard 2020	3368 Bourne 2020	3718 DuPreez 2018
3366 Bunn 2016	0.0%							
3544 Boots 2014	0.0%	0.0%						
4285 Maffioletti 2019	0.0%	0.0%	0.0%					
4531 Lins 2014	0.0%	0.0%	0.0%	0.0%				
5481 Pritchard 2020	0.0%	0.0%	0.0%	0.0%	0.0%			
3368 Bourne 2020	0.0%	0.0%	0.0%	0.0%	0.0%	0.0%		
3718 DuPreez 2018	0.0%	0.0%	0.0%	6.3%	0.0%	0.0%	0.0%	
807 Irons 2020	0.0%	0.0%	0.0%	0.0%	0.0%	0.0%	16.7%	0.0%
897 AlDaken 2018	0.0%	0.0%	0.0%	0.0%	0.0%	0.0%	0.0%	0.0%
2367 Hopwood 2018	0.0%	0.0%	0.0%	0.0%	0.0%	0.0%	0.0%	0.0%
6171 Smith 2014	0.0%	0.0%	0.0%	0.0%	0.0%	0.0%	0.0%	0.0%
5049 Sin 2018	0.0%	0.0%	0.0%	0.0%	0.0%	0.0%	0.0%	0.0%
3195 Shaw 2009	0.0%	0.0%	0.0%	0.0%	0.0%	0.0%	0.0%	0.0%
1459 Dam 2016	0.0%	0.0%	0.0%	0.0%	0.0%	0.0%	0.0%	0.0%
5843 Miles 2020	0.0%	0.0%	0.0%	0.0%	0.0%	0.0%	0.0%	0.0%
4806 Parkinson 2019	0.0%	0.0%	0.0%	0.0%	0.0%	0.0%	0.0%	0.0%
5510 Li 2016	0.0%	0.0%	0.0%	0.0%	0.0%	0.0%	0.0%	0.0%

For peer review only

For peer review only

ROOVE) - Considering Structural Missingness

**153**	=	**Total nodes (pairs of reviews)**
151	=	Slight overlap (<5%)
1	=	Moderate overlap (5% to <10%)
0	=	High overlap (10% to <15%)
1	=	Very High overlap (≥15%)

807 Irons 2020	897 AlDaken 2018	2367 Hopwood 2018	6171 Smith 2014	5049 Sin 2018	3195 Shaw 2009	1459 Dam 2016	5843 Miles 2020	4806 Parkinson 2019
0.0%								
0.0%	0.0%							
0.0%	0.0%	0.0%						
0.0%	0.0%	0.0%	0.0%					
0.0%	0.0%	0.0%	0.0%	0.0%				
0.0%	0.0%	0.0%	0.0%	0.0%	0.0%			
0.0%	0.0%	0.0%	0.0%	0.0%	0.0%	0.0%		
0.0%	0.0%	0.0%	0.0%	0.0%	0.0%	0.0%	0.0%	
0.0%	0.0%	0.0%	0.0%	0.0%	0.0%	0.0%	0.0%	0.0%

For peer review only

For peer review only - http://bmjopen.bmj.com/site/about/guidelines.xhtml

For peer review only

For peer review only

For peer review only

For peer review only

For peer review only

For peer review only

For peer review only

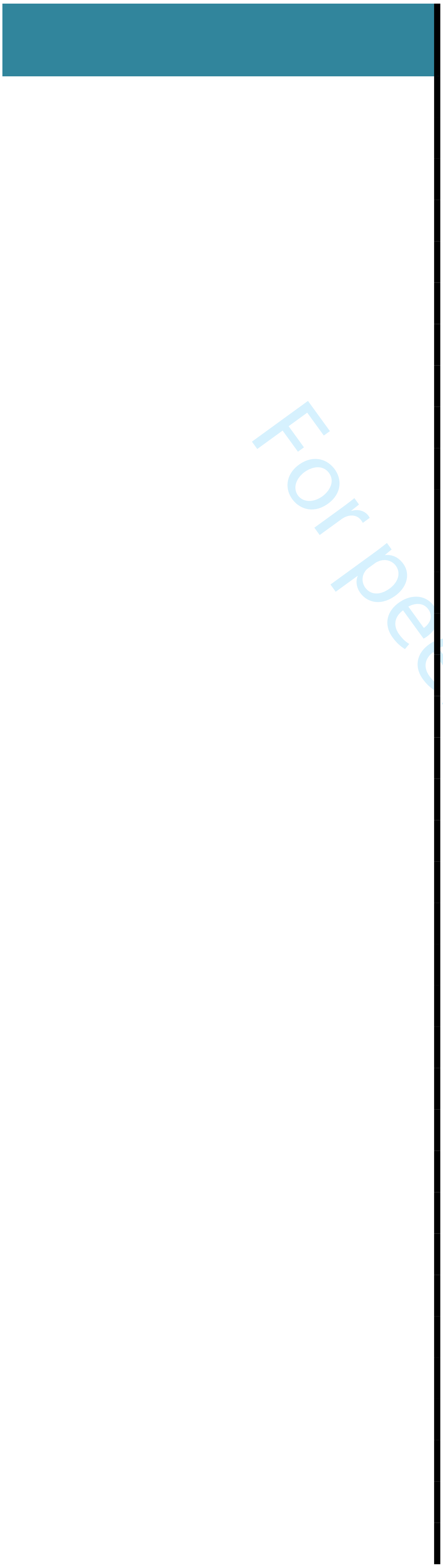

For peer review only

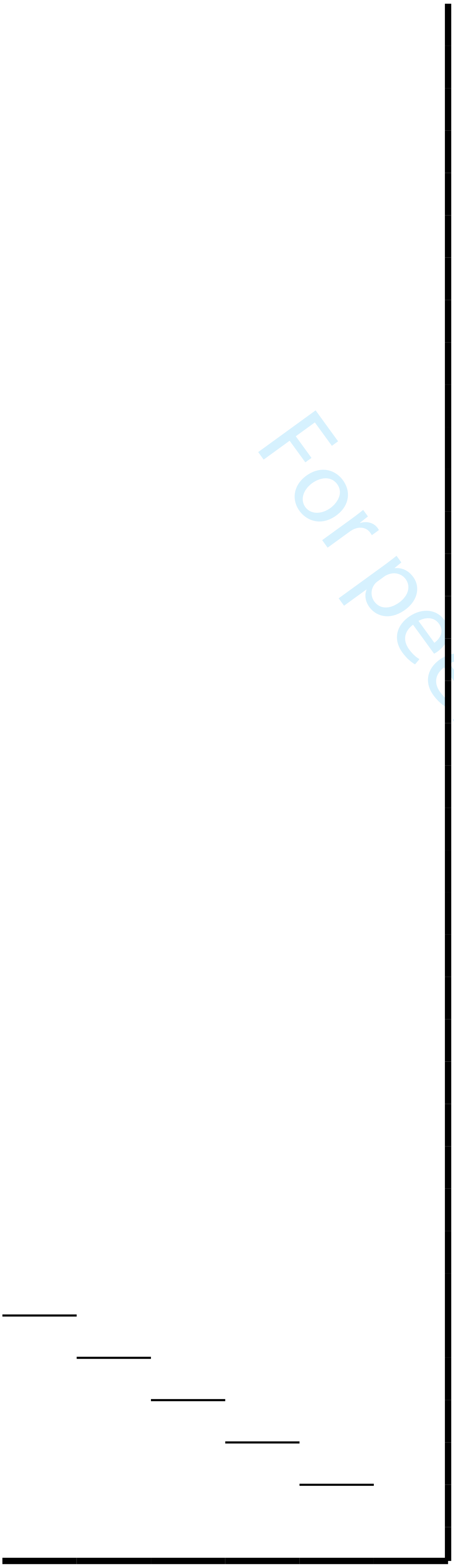

For peer review only

Overall results

G

Number of columns (number of reviews)	c	18
Number of rows (number of index publications)	r	163
Number of included primary studies (including double counting)	N	160
Covered area	N/(rc)	5.45%
Corrected covered area	(N-r)/(rc-r)	-0.11%
Interpretation of overlap	**Slight overlap**	
Structural Zeros	X	259
Corrected covered area (adjusting by structural zeros)	(N-r)/(rc-r-X)	-0.12%

N° of non-overlapped primary studies	In 1 SR	154
Number of overlapped primary studies	In 2 SRs	3
	In 3 SRs	0
	In 4 SRs	0
	In 5 SRs	0
	In 6 SRs	0
	In 7 SRs	0
	In 8 SRs	0
	In 9 SRs	0
	In 10 SRs	0
	In 11 SRs	0
	In 12 SRs	0
	In 13 SRs	0
	In 14 SRs	0
	In 15 or more SRs	0

For peer review only

raphical Representation of Overlap for OVErviews (GRO

	5244 Goeman 20	3366 Bunn 2016	3544 Boots 2014	4285 Maffioletti 2019	4531 Lins 2014	5481 Pritchard 2020	3368 Bourne 2020	3718 DuPreez 2018	807 Irons 2020	897 AlDaken 2018
3366 Bunn 2016	0.0%									
3544 Boots 2014	0.0%	0.0%								
4285 Maffioletti 2019	0.0%	0.0%	0.0%							
4531 Lins 2014	0.0%	0.0%	0.0%	0.0%						
5481 Pritchard 2020	0.0%	0.0%	0.0%	0.0%	0.0%					
3368 Bourne 2020	0.0%	0.0%	0.0%	0.0%	0.0%	0.0%				
3718 DuPreez 2018	0.0%	0.0%	0.0%	6.3%	0.0%	0.0%	0.0%			
807 Irons 2020	0.0%	0.0%	0.0%	0.0%	0.0%	0.0%	16.7%	0.0%		
897 AlDaken 2018	0.0%	0.0%	0.0%	0.0%	0.0%	0.0%	0.0%	0.0%	0.0%	
2367 Hopwood 2018	0.0%	0.0%	0.0%	0.0%	0.0%	0.0%	0.0%	0.0%	0.0%	0.0%
6171 Smith 2014	0.0%	0.0%	0.0%	0.0%	0.0%	0.0%	0.0%	0.0%	0.0%	0.0%
5049 Sin 2018	0.0%	0.0%	0.0%	0.0%	0.0%	0.0%	0.0%	0.0%	0.0%	0.0%
3195 Shaw 2009	0.0%	0.0%	0.0%	0.0%	0.0%	0.0%	0.0%	0.0%	0.0%	0.0%
1459 Dam 2016	0.0%	0.0%	0.0%	0.0%	0.0%	0.0%	0.0%	0.0%	0.0%	0.0%
5843 Miles 2020	0.0%	0.0%	0.0%	0.0%	0.0%	0.0%	0.0%	0.0%	0.0%	0.0%
4806 Parkinson 2019	0.0%	0.0%	0.0%	0.0%	0.0%	0.0%	0.0%	0.0%	0.0%	0.0%
5510 Li 2016	0.0%	0.0%	0.0%	0.0%	0.0%	0.0%	0.0%	0.0%	0.0%	0.0%

**153**
151
1
0
1

For peer review only

For peer review only

OVE)

=	**Total nodes (pairs of reviews)**
=	Slight overlap (<5%)
=	Moderate overlap (5% to <10%)
=	High overlap (10% to <15%)
=	Very High overlap (>15%)

2367 Hopwood 2018	6171 Smith 2014	5049 Sin 2018	3195 Shaw 2009	1459 Dam 2016	5843 Miles 2020	4806 Parkinson 2019
0.0%						
0.0%	0.0%					
0.0%	0.0%	0.0%				
0.0%	0.0%	0.0%	0.0%			
0.0%	0.0%	0.0%	0.0%	0.0%		
0.0%	0.0%	0.0%	0.0%	0.0%	0.0%	
0.0%	0.0%	0.0%	0.0%	0.0%	0.0%	0.0%

For peer review only

For peer review only

[redacted]

For peer review only

For peer review only

[redacted]

For peer review only

For peer review only

For peer review only

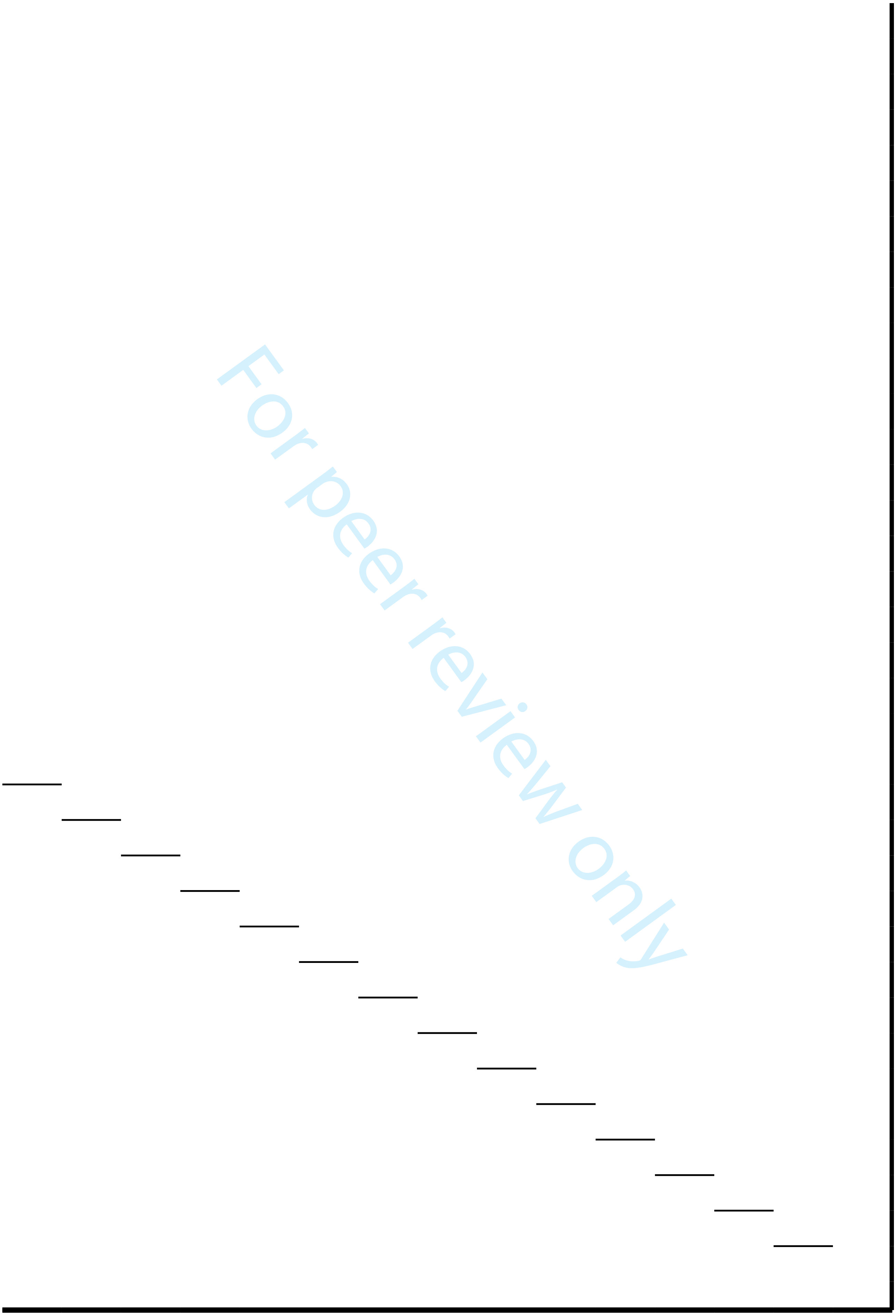

For peer review only

Graphical Representation of Overlap for OVErviews (G

	5244 Goem	3366 Bunn 2016	3544 Boots 2014	4285 Maffioletti 2019	4531 Lins 2014	5481 Pritchard 2020	3368 Bourne 2020	3718 DuPreez 2018
3366 Bunn 2016	0.0%							
3544 Boots 2014	0.0%	0.0%						
4285 Maffioletti 2019	0.0%	0.0%	0.0%					
4531 Lins 2014	0.0%	0.0%	0.0%	0.0%				
5481 Pritchard 2020	0.0%	0.0%	0.0%	0.0%	0.0%			
3368 Bourne 2020	0.0%	0.0%	0.0%	0.0%	0.0%	0.0%		
3718 DuPreez 2018	0.0%	0.0%	0.0%	6.3%	0.0%	0.0%	0.0%	
807 Irons 2020	0.0%	0.0%	0.0%	0.0%	0.0%	0.0%	16.7%	0.0%
897 AlDaken 2018	0.0%	0.0%	0.0%	0.0%	0.0%	0.0%	0.0%	0.0%
2367 Hopwood 2018	0.0%	0.0%	0.0%	0.0%	0.0%	0.0%	0.0%	0.0%
6171 Smith 2014	0.0%	0.0%	0.0%	0.0%	0.0%	0.0%	0.0%	0.0%
5049 Sin 2018	0.0%	0.0%	0.0%	0.0%	0.0%	0.0%	0.0%	0.0%
3195 Shaw 2009	0.0%	0.0%	0.0%	0.0%	0.0%	0.0%	0.0%	0.0%
1459 Dam 2016	0.0%	0.0%	0.0%	0.0%	0.0%	0.0%	0.0%	0.0%
5843 Miles 2020	0.0%	0.0%	0.0%	0.0%	0.0%	0.0%	0.0%	0.0%
4806 Parkinson 2019	0.0%	0.0%	0.0%	0.0%	0.0%	0.0%	0.0%	0.0%
5510 Li 2016	0.0%	0.0%	0.0%	0.0%	0.0%	0.0%	0.0%	0.0%

For peer review only

For peer review only

ROOVE) - Considering Structural Missingness

**153**	=	**Total nodes (pairs of reviews)**
151	=	Slight overlap (<5%)
1	=	Moderate overlap (5% to <10%)
0	=	High overlap (10% to <15%)
1	=	Very High overlap (≥15%)

807 Irons 2020	897 AlDaken 2018	2367 Hopwood 2018	6171 Smith 2014	5049 Sin 2018	3195 Shaw 2009	1459 Dam 2016	5843 Miles 2020	4806 Parkinson 2019
0.0%								
0.0%	0.0%							
0.0%	0.0%	0.0%						
0.0%	0.0%	0.0%	0.0%					
0.0%	0.0%	0.0%	0.0%	0.0%				
0.0%	0.0%	0.0%	0.0%	0.0%	0.0%			
0.0%	0.0%	0.0%	0.0%	0.0%	0.0%	0.0%		
0.0%	0.0%	0.0%	0.0%	0.0%	0.0%	0.0%	0.0%	
0.0%	0.0%	0.0%	0.0%	0.0%	0.0%	0.0%	0.0%	0.0%

For peer review only

For peer review only

[redacted]

For peer review only

For peer review only

[redacted]

For peer review only

For peer review only

[redacted]

For peer review only

For peer review only

For peer review only

BMJ Open

Effectiveness of interventions designed to mitigate the negative health outcomes of informal caregiving to older adults: an umbrella review of systematic reviews and meta-analyses

Journal:	*BMJ Open*
Manuscript ID	bmjopen-2022-068646.R1
Article Type:	Original research
Date Submitted by the Author:	08-Mar-2023

Complete List of Authors:	Kirvalidze, Mariam; Karolinska Institute Department of Neurobiology Care Sciences and Society, Aging Research Centre<br>Abbadi, Ahmad; Aging Research Center, Department of Neurobiology, Care Sciences and Society, Karolinska Institutet and Stockholm University<br>Dahlberg, Lena; Aging Research Center, Department of Neurobiology, Care Sciences and Society, Karolinska Institutet and Stockholm University; School of Health and Welfare, Dalarna University<br>Sacco, Lawrence; Stress Research Institute, Department of Psychology, Stockholm University<br>Morin, Lucas; Department of Medical Epidemiology and Biostatistics, Karolinska Institutet; Inserm CIC 1431, University Hospital of Besançon<br>Calderón-Larrañaga, Amaia; Aging Research Center, Department of Neurobiology, Care Sciences and Society, Karolinska Institutet and Stockholm University; Stockholm Gerontology Research Center

**Primary Subject Heading**:	Public health
Secondary Subject Heading:	Evidence based practice, Health policy, Health services research, Qualitative research, Research methods
Keywords:	PUBLIC HEALTH, QUALITATIVE RESEARCH, SOCIAL MEDICINE, STATISTICS & RESEARCH METHODS, Health policy < HEALTH SERVICES ADMINISTRATION & MANAGEMENT

SCHOLARONE™ Manuscripts

I, the Submitting Author has the right to grant and does grant on behalf of all authors of the Work (as defined in the below author licence), an exclusive licence and/or a non-exclusive licence for contributions from authors who are: i) UK Crown employees; ii) where BMJ has agreed a CC-BY licence shall apply, and/or iii) in accordance with the terms applicable for US Federal Government officers or employees acting as part of their official duties; on a worldwide, perpetual, irrevocable, royalty-free basis to BMJ Publishing Group Ltd ("BMJ") its licensees and where the relevant Journal is co-owned by BMJ to the co-owners of the Journal, to publish the Work in this journal and any other BMJ products and to exploit all rights, as set out in our licence.

The Submitting Author accepts and understands that any supply made under these terms is made by BMJ to the Submitting Author unless you are acting as an employee on behalf of your employer or a postgraduate student of an affiliated institution which is paying any applicable article publishing charge ("APC") for Open Access articles. Where the Submitting Author wishes to make the Work available on an Open Access basis (and intends to pay the relevant APC), the terms of reuse of such Open Access shall be governed by a Creative Commons licence – details of these licences and which Creative Commons licence will apply to this Work are set out in our licence referred to above.

Other than as permitted in any relevant BMJ Author's Self Archiving Policies, I confirm this Work has not been accepted for publication elsewhere, is not being considered for publication elsewhere and does not duplicate material already published. I confirm all authors consent to publication of this Work and authorise the granting of this licence.

For peer review only

TITLE

Effectiveness of interventions designed to mitigate the negative health outcomes of informal caregiving to older adults: an umbrella review of systematic reviews and meta-analyses

AUTHORS:

- Mariam Kirvalidze MPH[a] (mariam.kirvalidze@ki.se)
- Ahmad Abbadi MMScPH[a] (ahmad.abbadi@ki.se)
- Lena Dahlberg PhD[a,b] (ldh@du.se)
- Lawrence B. Sacco PhD[c] (lawrence.sacco@su.se)
- Lucas Morin PhD[d,e*] (lucas.morin@inserm.fr)
- Amaia Calderón-Larrañaga PhD[a,f*] (amaia.calderon.larranaga@ki.se)

AFFILIATIONS:

a) Aging Research Center, Department of Neurobiology, Care Sciences and Society, Karolinska Institutet and Stockholm University, Solna, Sweden
b) School of Health and Welfare, Dalarna University, Falun, Sweden
c) Stress Research Institute, Department of Psychology, Stockholm University, Stockholm, Sweden
d) Inserm CIC 1431, University Hospital of Besançon, Besançon, France
e) Department of Medical Epidemiology and Biostatistics, Karolinska Institutet, Solna, Sweden
f) Stockholm Gerontology Research Center, Stockholm, Sweden

*Lucas Morin and Amaia Calderón-Larrañaga share the last authorship

CORRESPONDING AUTHOR

Mariam Kirvalidze
Aging Research Center, Karolinska Institutet
Tomtebodavägen 18 A, 171 65 Solna, Sweden
Mariam.kirvalidze@ki.se

For peer review only

For peer review only - http://bmjopen.bmj.com/site/about/guidelines.xhtml

ABSTRACT

Objectives. This umbrella review aimed to evaluate whether certain interventions can mitigate the negative health consequences of caregiving, which interventions are more effective than others depending on the circumstances, and how these interventions are experienced by caregivers themselves.

Design. An umbrella review of systematic reviews was conducted.

Data sources. Quantitative (with or without meta-analyses), qualitative and mixed-method systematic reviews were included.

Eligibility Criteria. Reviews were considered eligible if they met the following criteria: included primary studies targeting informal (i.e., unpaid) caregivers of older people or persons presenting with aging-related diseases; focused on support interventions and assessed their effectiveness (quantitative reviews) or their implementation and/or the lived experience of the target population (qualitative reviews); included physical or mental health-related outcomes of informal caregivers.

Data extraction and synthesis. A total of 47 reviews were included, covering 619 distinct primary studies. Each potentially eligible review underwent critical appraisal and citation overlap assessment. Data were extracted independently by two reviewers and cross-checked. Quantitative review results were synthesized narratively and presented in tabular format, while qualitative findings were compiled using the mega-aggregation framework synthesis method.

Results. The evidence regarding the effectiveness of interventions on physical and mental health outcomes was inconclusive. Quantitative reviews were highly discordant, whereas qualitative reviews only reported practical, emotional, and relational benefits. Multicomponent and person-centered interventions seemed to yield highest effectiveness and acceptability. Heterogeneity among caregivers, care receivers and care contexts was often overlooked. Important issues related to the low quality of evidence and futile overproduction of similar reviews were identified.

Conclusions. Lack of robust evidence calls for better intervention research and evaluation practices. It may be warranted to avoid one-size-fits-all approaches to intervention design. Primary care and other existing resources should be leveraged to support interventions, possibly with increasing contributions from the non-profit sector.

Registration. *PROSPERO*: CRD42021252841**;** *BMJ Open*: doi:10.1136/bmjopen-2021-053117

STRENGTHS AND LIMITATIONS OF THIS STUDY

- The umbrella review methodology enabled us to synthesize and describe the state of the evidence on the topic of interventions to mitigate the negative health consequences of informal caregiving.
- The review benefits from the mixed-methods approach, as we included both quantitative reviews on effectiveness and qualitative syntheses exploring complex aspects related to the experiences of caregivers.
- Synthesis is confined to a descriptive, narrative output due to heterogeneity of included reviews.
  More recent primary studies on new interventions were not captured, as they would not have been included in systematic reviews selected for this umbrella review.

For peer review only - http://bmjopen.bmj.com/site/about/guidelines.xhtml

INTRODUCTION

Informal caregivers are defined as any relative, partner, friend or neighbor who provides a broad range of assistance to an older person who lives with a chronic or disabling condition and with whom they have a significant personal relationship.[1] Their role has become increasingly important, as populations age and professional social care services struggle to meet the increasing care demands. Improved life expectancy leads to more years spent with late-life dependency,[2] and this burden often falls on the families of older adults. It is now estimated that informal caregivers contribute to the majority of care for persons aged over 50 in most European countries[3] and long-term care relies heavily on the availability of informal caregivers, especially in the current context of care worker shortages.[4] In the near future, some countries are expected to have 'care gaps' or, in other words, insufficient numbers of informal caregivers to meet the increasingly complex care needs of older adults.[5,6]

Informalization of care has come with important concerns about the potential side effects of caregiving, and with growing interest from decision makers and stakeholders in the effectiveness of existing support interventions to alleviate these negative consequences. The burden of informal care can be determined by various factors, such as the intensity of caregiving, care receiver's dependency level, relationship with the care receiver, available social support from the community, and caregiver's own health.[7–10] Research findings on the health effects of caregiving has been mixed, with some studies reporting a positive association with health and longevity[11] while others reveal a myriad of adverse repercussions, such as increased mortality,[12] poorer well-being,[13] and worse physical and mental health outcomes in general.[14] The socioeconomic status, age and gender of caregivers have also been found to moderate these adverse consequences: women, older people, and those with lower income bear the highest psychological and physical burden of informal caregiving.[15] There is now a broad consensus about the multifactorial nature of this burden and its complex ramifications in terms of health and well-being.[15,16]

There has been a considerable increase in the number of empirical studies (both qualitative and quantitative) on the topic of caregiver support interventions, followed—in recent years—by a large number of systematic reviews. Consequently, it has become challenging to keep a bird's-eye-view of this field of research. To provide decision makers and stakeholders with synthetic and actionable evidence, a logical next step is to conduct a review of systematic reviews, that is, an 'umbrella review'.[17] Umbrella reviews are designed to give a broad and high-level overview of the available evidence on a given topic by compiling existing reviews rather than aggregating findings from the individual studies included in these reviews. By stitching together reviews about different types of interventions, populations or conditions, they provide an opportunity to assess not only the content-related comprehensiveness of these reviews, but also the overall reliability of the available evidence. Although a handful of umbrella reviews or meta-reviews have already examined caregiver support interventions, their scopes have been limited to only one type of intervention and/or disease[18,19] and some were too broad or unsystematic to capture differences between caregiver groups.[20,21] Our understanding of the effectiveness of existing interventions for mitigating the harms of informal caregiving is currently hampered by the lack of up-to-date synthesis of the evidence focusing on more objective physical and mental health outcomes rather than perceived burden or subjective well-being. Moreover, there is a need for integrating quantitative findings about the effectiveness of interventions with qualitative findings on the lived experience of caregivers who received these interventions.

In particular, the following research questions were addressed: (1) Are there effective interventions to prevent and reduce the negative health consequences of informal caregiving? (2) Are certain types of interventions more effective than others? (3) Is there evidence that the effectiveness of interventions depends on caregiver, care receiver, care context and implementation characteristics? (4) How are the proposed interventions experienced by caregivers in terms of effectiveness and implementation outcomes?

For peer review only - http://bmjopen.bmj.com/site/about/guidelines.xhtml

METHODS

Protocol registration

The protocol for this umbrella review was registered *a priori* in the PROSPERO database[22] and published in a peer-reviewed journal (doi:10.1136/bmjopen-2021-053117).[23] **Supplementary Table 1** includes a list of all amendments made to the protocol after its registration, which will be mentioned in relevant sections below. The reporting of this umbrella review is based on the Preferred Reporting Items for Overviews of Reviews (PRIOR) statement.[24]

Eligibility criteria, information sources and search strategy

Inclusion and exclusion criteria used to select reviews are presented in **Box 1** and discussed in detail in the umbrella review protocol.[23] Due to the abundance of published systematic reviews, the eligibility criteria were amended to exclude reviews of critically low quality based on our risk-of-bias assessment. This amendment is documented in **Supplementary Table 1**. The Medline, CINAHL, PsycINFO and Web of Science databases were initially searched from 1 January 2000 to 26 March 2021. In addition, we performed a manual search of the reference lists of included reviews. **Supplementary table 2** includes detailed search strategies and number of hits from all searched databases. Search strategies were developed by the review team in consultation with university librarians. Even though the first reviews on caregiver intervention research were published in the 1990s, we focused on reviews published since 2000 to capture studies conducted in the context of more current social settings. We also restricted the inclusion to reviews published in languages spoken by research team members: English, Swedish, Spanish, French, Italian and German. The titles and abstracts of all references as well as full texts of preliminarily selected reviews were screened against the eligibility criteria independently by two reviewers using the *Covidence®* software developed by the Cochrane collaboration.[25] Any dissent in abstract screening and/or full-text assessment were resolved by discussion moderated by a third reviewer. Finally, our search was updated to capture any eligible review published between 26 March 2021 and 31 January 2023.

Selection of reviews: risk-of-bias assessment and overlap assessment

Each quantitative review was critically appraised by two reviewers working independently, using the AMSTAR-2 checklist.[26] This checklist categorizes the quality of the reviews based on seven 'critical' and nine 'non-critical' domains. The research team made a consensus-based decision do downgrade item #10 (*Did the review authors report on the sources of funding for the studies included in the review?*) from 'critical' to 'non-critical' since this information was only available in Cochrane reviews (amendment is documented in **Supplementary Table 1**). Based on our appraisal, the reviews were grouped into 'critically low', 'low', 'moderate', and high-quality categories, and critically low-quality reviews were excluded from the umbrella review. Qualitative reviews were also assessed independently by two team members, who used an *ad hoc* quality appraisal checklist adapted from the Joanna Briggs Institute Checklist for Systematic Reviews and Research Syntheses[17] and available in the published protocol[23] The tool was developed and piloted by all team members on a sample of five randomly selected reviews. Items #3, #4, #7 and #10 were considered as 'critical', with reviews exhibiting more than two critical flaws being excluded. The quality of mixed-methods reviews was assessed using the above-mentioned tools for their quantitative and qualitative components, respectively. Any dissent in the risk-of-bias assessment process was resolved through discussions moderated by a third investigator.

Reporting biases arising from primary studies included in the systematic reviews were assessed using the AMSTAR-2 tool[26] which includes critical items related, among other aspects, to selective reporting of outcomes and publication bias. We were more lenient with qualitative reviews (or parts of reviews related to qualitative data) because risk-of-bias assessment is less common and structured in qualitative research. We did, however, include items related to the quality of primary studies in our *ad hoc* tool for qualitative reviews.

For peer review only - http://bmjopen.bmj.com/site/about/guidelines.xhtml

Box 1. Inclusion and exclusion criteria for quantitative and qualitative reviews.

▸ Inclusion criteria

Publication type, date and language

- Reviews published in a peer-reviewed journal OR as a report done or mandated by an official health agency (*e.g., Health Technology Assessment agency*)
- Reviews published between January 1, 2000, and March 26, 2021
- Reviews published in English, Swedish, Spanish, French, Italian or German

Study design

- For quantitative reviews: reviews including a reproducible, systematic search strategy, AND clearly defined inclusion/exclusion criteria AND risk of bias assessment for all included primary studies
- For qualitative reviews: reviews including a reproducible, systematic search strategy, AND defined inclusion/exclusion criteria

Population

- Reviews concerning informal caregivers (*i.e., people who regularly provide unpaid care to a family member, friend or neighbor*) of older people OR of persons presenting with aging-related disease (*e.g., dementia, stroke, Parkinson's disease, cancer, heart failure, multimorbidity, frailty*)

Intervention

- Reviews focusing on interventions and assessing either their effectiveness (for quantitative reviews) or their implementation and/or the lived experience of the target population (for qualitative reviews)

Outcome

- Reviews including physical or mental health-related outcomes of informal caregivers, including health-related quality of life

▸ Exclusion criteria

Study quality and overlap

- For quantitative reviews: reviews of 'critically low' quality as per AMSTAR-2 assessment tool
- For qualitative reviews: reviews with two or more critical flaws as per *ad hoc* assessment tool*
- Review pairs with very high or high overlap (as per Corrected Covered Area method) were examined and older, less relevant, or lower quality reviews were excluded

Additional exclusion criteria

- Reviews of interventions focusing *exclusively* on care receivers as the target population
- Reviews focusing *exclusively* on interventions for caregivers of young populations
- Reviews measuring *exclusively* non-health related outcomes, such as caregiver burden, stress/strain, work or financial status, family relations, breakdown of informal care
- Reviews focusing *exclusively* on end-of-life care interventions

*See the published protocol (doi:10.1136/bmjopen-2021-053117) for the *ad hoc* assessment tool for qualitative reviews

For peer review only - http://bmjopen.bmj.com/site/about/guidelines.xhtml

The degree of overlap of primary studies included in the reviews (namely, the fraction of evidence synthesized in two or more reviews) was estimated using the Corrected Covered Area (CCA) methodology[27] and the Graphical Representation of Overlap for OVErviews (GROOVE) open-access tool.[28] We built separate citation matrices for quantitative and qualitative primary studies and we accounted for structural missingness based on publication date (i.e., primary papers published after the review was completed were not marked as missing from the reviews). Guided by the methodology developed by Pollock et al.[29] two team members went through the pairs with "very high (≥15%)" and "high (10-15%)" overlap, and decisions on inclusion were made based on relevance, search dates, potential contribution to the umbrella review, and quality of the publication (described in **Supplementary Table 3**).

Data extraction

Data from included reviews were extracted by two team members in structured spreadsheets designed and validated *a priori* by all the investigators involved in this umbrella review. The quality and validity of the extracted data was assessed through regular cross-checks. For quantitative reviews, we extracted the following information: review objectives, methodological aspects (inclusion/exclusion criteria, search dates and databases, synthesis methods), target population and disease, characteristics of the interventions of interest, sociodemographic background of caregivers, health outcomes, degree of effectiveness of interventions (with/without meta-analysis), implications for practice and research, as well as the full list of primary studies included in the review. The latter was extracted for the purpose of overlap assessment. Qualitative reviews were characterized in terms of methodology, and we extracted all the *verbatim* text related to the reported caregivers' experiences of interventions.

Synthesis methods

For quantitative reviews, we conducted a narrative synthesis and provided findings in a tabular format, organized by intervention type. To provide a data-driven list of intervention types, we adapted Gaugler et al.'s[30] original typology into the following classification: case management, psychosocial and education/skills building, respite care, relaxation and leisure, and mindfulness. If reviews compared different types of interventions, the outcome of this comparison was reported. Factors related to the caregiver, care receiver and/or care context affecting the effectiveness of interventions were identified and reported whenever possible.

For qualitative reviews, we followed the 'mega-aggregation framework' synthesis method[31] **Supplementary Table 1** documents post-protocol amendments related to synthesis of qualitative reviews, while **Supplementary Table 4** describes steps involved in the mega-aggregation process. First, the extracted *verbatim* texts from reviews were coded line-by-line (complete coding) by two team members. Codes were then iteratively and deductively categorized based on the adapted version of van Houtven et al.'s framework[32] Throughout the review, we refer to verbatim texts extracted from reviews and their corresponding codes as third-order constructs, while primary studies inform second-order constructs, and the communication from participants (i.e., caregivers) is considered as a first-order construct. Thus, the themes, categories, and sub-categories that we generated based on third-order constructs (i.e., reviews) are termed as fourth-order constructs[31] In case our fourth-order codes did not fit fully into the framework, they were inductively categorized into new themes. Codes referring to care receiver or staff opinions were removed. A third investigator checked the outcome of the categorization independently, and several consensus meetings were held to resolve disagreements and finalize the synthesis of qualitative materials.

As a complementary output, we used parallel convergence approach (i.e., synthesizing quantitative and qualitative evidence separately and bringing them together at the final stage) to update our initial conceptual framework that was based exclusively on expert opinion and/or existing literature (see the published protocol[23]). The resulting framework, *From Support Interventions to Improved Caregiver Outcomes (SIICO)*, will be presented below and aims to substantiate the potential pathway (and its various modifiers and mediators) linking caregiver support interventions to improved health outcomes.

For peer review only - http://bmjopen.bmj.com/site/about/guidelines.xhtml

Patient and public involvement (PPI)

No patients or members of the public were involved in the development of this umbrella review. However, the scope and methods of this review were informed by the literature and discussions with experts in the field.

For peer review only

For peer review only - http://bmjopen.bmj.com/site/about/guidelines.xhtml

RESULTS

Selection of reviews: risk-of-bias assessment and overlap assessment

Our search strategy resulted in a total of 6209 unique records, of which 5906 were excluded at the stage of title and abstract screening (**Figure 1**). From the 303 reviews that underwent full-text screening, 158 were excluded and 145 were further assessed for risk of bias. We excluded 92 quantitative and 9 qualitative reviews considered as being of 'critically low' quality. Results of the risk-of-bias assessment for all potentially eligible reviews are reported in **Supplementary Tables 5a and 5b**.

Of the 51 remaining quantitative reviews, 32 fell under the 'low quality' category, 13 were classified as being of 'moderate quality' and 6 were rated as high-quality reviews. Overall, the most common issues were lack of pre-registered protocol, absence of a full list of excluded studies with a rationale for the exclusion of each study, lack of reporting of sources of funding for primary studies, methodological issues related to meta-analyses, and suboptimal assessment of heterogeneity and publication bias. All 18 qualitative reviews eligible at this stage had only one or no critical flaw. Most common pitfalls included: lack of clarity regarding participation of researchers in synthesis process, lack of justification of qualitative synthesis methods, and lack of information on data extraction procedures.

After risk-of-bias assessment, reviews were examined for primary study overlap. Full citation matrices (accounting for structural missingness based on publication date) and resulting pyramids are available in **Supplementary Files 1 and 2**. Among the 18 qualitative and mixed-methods reviews assessed for overlap, we did not exclude any reviews since the degree of overlap was low. However, out of the 51 eligible quantitative reviews, we examined 33 pairs that reported 'very high' level of overlap (≥15%) and further checked 40 pairs of reviews that reported 'high' level of overlap (10–15%). Upon rigorous assessment of overlap, 14 quantitative reviews were finally excluded (see **Supplementary Table 3** for detailed description of the overlap decisions).

Finally, 47 reviews covering 619 primary studies were included in the umbrella review. Of these, 10 contributed to qualitative data only, 8 were included as both qualitative and quantitative data sources, while 29 reviews contributed exclusively to quantitative synthesis.

Characteristics of the included systematic reviews

All included reviews were published in English. Publication dates spanned from 2009 to 2021. Most primary studies were conducted in North America or Western Europe. Reviews set various inclusion and exclusion criteria regarding the design of primary studies, with some reviews focusing on randomized controlled trials, while others casted a wider net including quasi-experimental and qualitative studies. Among quantitative reviews, 18 undertook a meta-analysis while 19 undertook a data synthesis without meta-analysis. In qualitative reviews (or qualitative parts of mixed reviews), the most common synthesis approach was narrative (14 reviews), but four reviews reported a qualitative meta-synthesis. Characteristics of all included reviews, such as search dates, number of studies, demographics of included populations and types of interventions examined are summarized and presented in **Supplementary Table 6**.

Main findings: quantitative reviews

Case management interventions

Eight reviews reported quantitative findings related to the effectiveness of the interventions involving case management (**Table 1 and Supplementary Table 7**).[33–40] Seven of these reviews focused on caregivers of persons with dementia,[33–39] while one took interest in caregivers of stroke survivors.[40] Health-related outcomes varied and included: depression (n=6), health-related quality of life (n=6), anxiety (n=2), general health (n=1) and self-rated

For peer review only - http://bmjopen.bmj.com/site/about/guidelines.xhtml

health (*n*=1). Three reviews included a meta-analysis.[35,38,40] Five reviews reported no significant effect of case management interventions on caregivers' health outcomes,[34,35,37,39,40] one review provided inconclusive findings,[33] one review reported one primary study showing positive effects on depression,[36] and finally another review[38] showed a significant improvement in short term depressive symptoms and general health (albeit with waning effects at longer-term follow-ups). Two reviews demonstrated that multicomponent interventions that include other types of approaches in addition to case management may have positive effects[34,35] Lee et al.[35] also reported that interventions including caregivers with high quality of life at baseline or those who cared for people with less serious health conditions were less likely to be effective. The other five reviews either did not explore the issue of heterogeneity of caregivers, care receivers and care context, or stated that these data were not reported by primary studies.[40]

For peer review only

For peer review only - http://bmjopen.bmj.com/site/about/guidelines.xhtml

Table 1. Interventions involving case management.

First author, year	Included primary studies	Disease of care receivers	Characteristics of intervention	Control group	Health outcomes of caregiver	Quality of evidence
**Goeman, 2016**	24 RCTs, 1 cohort study, 1 case study, 2 mixed methods	Dementia	Case management by nurse or dementia care provider involving visits, calls, and emails.	Usual care, home visits, educational sessions, phone calls, helpline, or not reported	Depression, HR-QoL, general health	●●●●
**Greenwood, 2016**	1 quasi-experimental	Dementia	General practice-based intervention delivered by a nurse, to augment care from primary care physicians.	Usual care and educational materials	Depressive symptoms	●●○○
**Hopwood, 2018**	2 RCTs, 1 quasi-experimental, 7 mixed methods	Dementia	Internet-based interventions involving nurses, health professionals, social workers using various internet-based media to manage caregivers.	Face-to-face delivered information, usual care	Depression, anxiety, HR-QoL, self-rated health	●●○○
**Lee, 2020**	4 RCTs	Dementia or MCI	Community-based interventions delivered by various professionals. Duration varied from 3 to 18 months.	Usual care or not reported	HR-QoL	●●○○
**Lucero, 2019**	2 RCTs	Dementia	Telephone or computer-based health-related planning, monitored by nurses.	Usual care or training on available local resources	Depression, anxiety	●●●○
**Piersol, 2017**	2 RCTs	Dementia	Case management by nurses or occupational therapists.	Not reported	HR-QoL	●●○○
**Reilly, 2015**	11 RCTs	Dementia	Dyadic and non-dyadic interventions involving a case manager. Majority face to face, delivered by various professionals. Duration varied from 4 months to 2 years.	Waiting list, usual care, or augmented usual care	HR-QoL, depression, general health	●●●●
**Pucciareli, 2020**	5 RCTs, 1 quasi-RCT	Stroke	Informational interventions that included home visits and management after discharge.	Usual care or not reported	Depression, HR-QoL	●●●○

Abbreviations: MCI, mild cognitive impairment; RCT, randomized controlled trial; HR-QoL, health-related quality of life
Legend for quality assessment based on AMSTAR-2: ●●●● High; ●●●○ Moderate; ●●○○ Low
A summary of the main findings of each review is available in Supplementary Table 7.

For peer review only - http://bmjopen.bmj.com/site/about/guidelines.xhtml

Psychosocial and education/skills building interventions

A total of 23 reviews reported quantitative findings related to effectiveness of interventions involving psychosocial support, education, and skills-building for caregivers (**Tables 2 and 3 and Supplementary Table 8**). Of these, 19 reviews included and synthesized findings on both psychosocial and educational interventions,[34,35,37,39–54] while two reviews focused solely on psychosocial support,[55,56] and another two only on education/training.[57,58] Care receivers' health problems spanned from dementia (*n*=12) and stroke (*n*=4) to cancer (*n*=4) and other chronic illnesses (*n*=3). Most reviews included multiple interventions (e.g., information provision, psychosocial support, educational training, skills-building), even though the label "multicomponent" was used differently across reviews. Meta-analysis was performed in 14 reviews. Of these, seven reviews reported insignificant effect estimates or little to no effects following psychosocial and/or educational interventions,[35,40,41,44,48,54,59] four reviews reported significant effect estimates related to similar interventions,[47,52,53,58] while three reviews reported significant changes in mental health specifically after cognitive behavioral therapy (CBT) interventions.[49,55,56] Narrative reviews often failed to reach definitive conclusions due to discordant results (similar numbers of studies reporting significant and non-significant estimates),[42,43,45,46,50,51] but some reviews reported overall positive findings.[34,37] Conclusions were discordant regarding remote interventions. For instance, the evidence documenting the benefit of telephone-based psychosocial support was found to be inconclusive by Corry et al.[41] and Gonzalez-Fraile et al.,[48] while Lins et al.[52] reported positive effects on depressive symptoms. Lee et al.[35] and Hopwood et al.[34] grouped certain interventions under the "multicomponent" category and reported that these were associated with significant positive effects on health-related quality of life and anxiety and depression, respectively. Treanor et al.[44] reported significant positive effects on quality of life in the short term, with waning effects over time. Akarsu et al.[47] examined the effectiveness of psychological, multicomponent, and educational interventions in ethnic minority caregivers, reporting an overall mean reduction in depression scores.

Some reviews explored findings from primary studies depending on the caregiver, care receiver, and care context characteristics as well as implementation-related aspects. Successful interventions were reported to be more individualized[45] proactive rather than reactive,[51] developed using user input from the target groups,[39,47] and guided by competent professionals[37] or peer caregivers with relatable experiences.[35] Heckel et al.[43] reported that telephone helplines were mostly utilized by white, higher income, middle-aged females, and lack of participation from other groups of caregivers should be investigated. Corry et al.[41] acknowledged that these data are often not reported by primary studies.

For peer review only - http://bmjopen.bmj.com/site/about/guidelines.xhtml

Table 2. Psychosocial, psychoeducational, and skills-building interventions: reviews that included both psychosocial and educational interventions (n=19).

First author, year	Included primary studies	Disease of care receivers	Characteristics of intervention	Control group	Health outcomes of caregiver	Quality of evidence
**Akarsu, 2019**	13 RCTs	Dementia	Psychological, multicomponent and educational interventions. Delivered to ethnic minority caregivers.	Minimal support measures	Depression	●●●○
**Gonzalez-Fraile, 2021**	26 RCTs and quasi-RCTs	Dementia	Remotely delivered interventions only. Predominant components: training with or without information, support with or without information, and interventions including both support and training elements.	Usual treatment or waiting list, minimal support, providing information only	Depression, depressive symptoms, HR-QoL	●●●○
**Greenwood, 2016**	2 RCTs, 1 quasi-RCT	Dementia	Education, training, CBT, delivered for various durations and locations (home-based, primary care).	Usual care or not reported	Depression, general health	●●○○
**Hopwood, 2018**	9 RCTs, 20 mixed-method, 7 quasi-RCTs	Dementia	Internet-based only. Information, online sessions or modules, links to resources, training, peer interaction online, small groups peer support. Duration varied from 2 weeks to 12 months.	Information only, waiting list, email newsletter, usual care, telephone support, written information, video, website	HR-QoL, depression, anxiety, self-rated health	●●○○
**Lee, 2020**	14 RCTs	Dementia or MCI	CBT, group sessions, dyadic sessions, home visits, meetings, website, support calls.	Usual care or information only	HR-QoL	●●○○
**Lins, 2014**	9 RCTs	Dementia	Telephone counselling with or without educational material and workbook. Varying methods and duration (20-60 minutes per call).	Usual care or friendly calls	Depression, anxiety	●●●○
**Piersol, 2017**	41 RCTs and quasi-RCTs	Dementia	Group interventions, CBT, single component support interventions, multicomponent psychoeducational interventions. Delivered through various methods.	Not reported	HR-QoL, depression, anxiety	●●○○
**Teahan, 2020**	24 RCTs	Dementia	Counselling, information, education on dementia, CBT, relaxation techniques, communication skills, emotional control, other skills, MBSR, physical exercise, dyadic or individual.	Usual care, educational material, follow up calls, enhanced respite care	Depression, HR-QoL, general health	●●○○
**Wiegelmann, 2021**	37 RCTs and quasi-RCTs	Dementia	Psychoeducation, counselling, CBT, and peer support. Delivered either face-to-face or remotely.	Usual care or not reported	HR-QoL, depression	●●○○
**Zabihi, 2020**	14 RCTs	Dementia, other illnesses	Behavioral activation, education, group support interventions, among others.	Usual care, waiting lists, phone calls, educational interventions	Depression (symptoms and diagnosis)	●●○○
**Gabriel, 2020**	6 RCTs, 6 NRSIs	Cancer	CBT, coping theory, psychoeducation, skills building.	Usual care	Psychological/emotional and physical domains of QoL	●●○○
**Heckel, 2019**	2 RCTs	Cancer	Telephone helplines. Variable duration, number of outcalls, and content of the calls.	Usual care	Depression, emotional distress	●●○○

For peer review only - http://bmjopen.bmj.com/site/about/guidelines.xhtml

**Treanor, 2019**	21 RCTs and quasi-RCTs	Cancer	Ppsychoeducational approaches in the form of coping skills training. Delivered predominantly by nurses. Most face-to-face, some by telephone or video.	Usual care or information only	HR-QoL, depression, anxiety, emotional distress, physical health status	●●●○
**Waldron, 2013**	6 RCTs	Cancer	Skills-training and CBT. Dyadic or individual. Some interventions delivered face-to-face, some by telephone.	Usual care	HR-QoL	●●○○
**Corry, 2019**	21 RCTs	Various	Psychosocial, educational and psychoeducational interventions. All interventions were individual and telephone based.	Usual care or non-telephone-based support	HR-QoL, Psychological health (depression, anxiety), physical health	●●●○
**Sin, 2018**	26 RCTs and quasi-RCTs	Various	Web-based ICT interventions (at least part of an intervention had to be web-based). Varying content and duration (from several days to months).	Usual care or not reported	Depression, anxiety, HR-QoL	●●●○
**Forster, 2012**	7 RCTs	Stroke	Informational interventions. Delivered in varying formats and durations.	No information, usual care, educational material, waiting list, workbook	Depression, HR-QoL	●●●●
**Minshall, 2019**	8 RCTs	Stroke	Individual, group, or dyadic interventions. Delivered by various professionals, in person or through telephone. Duration varied from 1 month to 3 years.	Usual care or not reported	Depression, HR-QoL	●●●○
**Pucciarelli, 2020**	6 RCTs and quasi-RCTs	Stroke	Educational interventions, with components of psychoeducation. Some delivered face-to-face, some by phone/web, some through home visits.	Usual care or not reported	Depression	●●●○

Abbreviations: RCT, randomized controlled trial; NRSI, non-randomized study of intervention; CBT, cognitive behavioral therapy; QoL, quality of life; HR-QoL, health-related quality of life; MBSR, mindfulness-based stress reduction; ICT, information and communication technology.
Legend for quality assessment based on AMSTAR-2: ●●●● High; ●●●○Moderate; ●●○○ Low
A summary of the main findings of each review is available in Supplementary Table 8.

For peer review only

For peer review only - http://bmjopen.bmj.com/site/about/guidelines.xhtml

Table 3. Psychosocial, psychoeducational, and skills-building interventions: Reviews that included only psychosocial (n=2) or only educational (n=2) interventions.

First author, year	Included primary studies	Disease of care receivers	Characteristics of intervention	Control group	Health outcomes of caregiver	Quality of evidence
**Bennett, 2019 (occupational therapy)**	9 RCTs	Dementia	Occupational therapy delivered at home for dyads. Tailored and goal-oriented interventions.	Usual care, education, collaborative call	Emotional distress, HR-QoL	●●○○
**Smith, 2019 (training)**	19 RCTs and quasi-RCTs	Stroke and older adults	Training delivered to facilitate care after discharge. Varying delivery mode (face-to-face, by phone, by different professionals) and duration.	Usual care, information only	HR-QoL, depression, anxiety	●●●○
**Hopkinson, 2019 (psychosocial)**	25 RCTs and quasi-RCTs	Dementia	CBT. Varying delivery mode and duration.	Support group control, psychoeducation control, information support control, usual care	Depression, anxiety	●●○○
**Wang, 2020 (psychosocial)**	6 RCTs	Neurocognitive diseases	Bibliotherapy. Either web- or video-based. Varying number of sessions and duration of each session.	Usual care, waiting list, educational video	Depression, anxiety	●●●○

Abbreviations: RCT, randomized controlled trial; CBT, cognitive behavioral therapy; QoL, quality of life; HR-QoL, health-related quality of life.
Legend for quality assessment based on AMSTAR-2: ●●●● High; ●●●○Moderate; ●●○○ Low
A summary of the main findings of each review is available in Supplementary Table 8.

For peer review only

For peer review only - http://bmjopen.bmj.com/site/about/guidelines.xhtml

Respite care

Four reviews explored the effectiveness of respite care services (**Table 4 and Supplementary Table 9**).[60–63] Three of these reviews focused on caregivers of persons with dementia,[61–63] while one review took interest in caregivers of people with any chronic illness.[60] Health-related outcomes varied and included: depression (n=4), anxiety (n=2), and general health (n=1). The largest review in this category—and the only one including a meta-analysis—reported no significant effects on caregivers' mental health outcomes.[60] However, narrative findings from studies not eligible for meta-analysis in the same review and another review by Maffioletti et al.[61] were rather positive, although they remained discordant.[60,61] Vandepitte et al.[62] and Maayan et al.[63] reported small or insignificant effects in relation to caregivers' health outcomes. Maayan et al.[63] reported that care receivers' disease severity could have been positively correlated with the effectiveness of respite, with caregivers of patients with milder symptoms not requiring as many breaks. The price of respite care (if privately purchased) was identified as an important factor for effectiveness and access.[60] Shaw et al.[60] found that longer interventions tended to have stronger benefit than shorter ones, and that the short-term incidence of depression was reduced among people who received home respite care but not in trials that evaluated day care.

For peer review only

For peer review only - http://bmjopen.bmj.com/site/about/guidelines.xhtml

Table 4. Interventions involving respite care.

First author, year	Included primary studies	Disease of care receivers	Characteristics of intervention	Control group	Health outcomes of caregiver	Quality of evidence
**Maayan, 2019**	3 RCTs	Dementia	In-home respite, day care, institutional care. Delivered by trained cares as well as volunteers.	Usual care, waiting list	Depression	●●●●
**Maffioletti, 2019**	10 quasi-experimental, 4 cross-sectional	Dementia	Self-financed or paid day care services by professionals or volunteers. Caregivers were supported in some studies with music therapy, socializing, or just free time.	Usual care or not reported	Depression, psychological well-being, general health	●●○○
**Vandepitte, 2016**	5 RCTs and NRSIs	Dementia	Day care, in-home delivery of respite. Varying duration and frequency.	No respite	Depression, anxiety	●●○○
**Shaw, 2009**	26 RCTs and quasi-RCTs, 79 observational studies	Various	Day care, mixed interventions, in-home and institutional care.	Usual care or no respite	Depression, anxiety	●●○○

Abbreviations: RCT, randomized controlled trial; NRSI, non-randomized study of intervention.
Legend for quality assessment based on AMSTAR-2: ●●●● High; ●●●○Moderate; ●●○○ Low
A summary of the main findings of each review is available in Supplementary Table 9.

For peer review only - http://bmjopen.bmj.com/site/about/guidelines.xhtml

Relaxation and leisure

A total of six reviews were grouped under this category (**Table 5 and Supplementary Table 10**),[35,37,64–67] including ones focusing on caregivers to people with dementia (*n*=3), neurological diseases (*n*=1), and various chronic illnesses (*n*=2). Health-related outcomes varied and included: depression (*n*=4), health-related quality of life (*n*=4), anxiety (*n*=4), physical health (*n*=3), and blood pressure and weight (*n*=1). Two reviews included a meta-analysis.[67,68] Interventions were heterogenous: two reviews included interventions related to physical activity/exercise,[64,65] one review included relaxation/alternative medicine interventions,[35] one review focused on creative arts interventions,[66] while two other reviews focused on "miscellaneous" interventions, including exercise, leisure programs and relaxation.[37,67] These interventions had generally positive effects. Meta-analyses by both Lee et al.[35] and Cheng et al.[67] showed large positive effect sizes for the effect of relaxation and miscellaneous activities on health-related quality of life and depressive symptoms. Reviews with narrative synthesis reported that creative arts interventions tended to have positive effects on well-being,[66] while exercise programs resulted in lower blood pressure and less depressive symptoms,[64] as well as increased muscle mass, strength[65] and better physical health.[37]

Most of the reviews in this category did not consider heterogeneity associated with caregiving factors or implementation characteristics. According to Doyle et al.,[65] spousal and family caregivers may gain more from engaging in dyadic exercise compared to when their care receivers exercise independently. Miscellaneous interventions examined by Cheng et al.[67] showed that caregivers' mean age significantly moderated the intervention effects, whereby younger caregivers benefited more in terms of reducing depressive symptoms.

For peer review only

For peer review only - http://bmjopen.bmj.com/site/about/guidelines.xhtml

Table 5. Interventions involving relaxation, physical activity, or leisure.

First author, year	Included primary studies	Disease of care receivers	Characteristics of intervention	Control group	Health outcomes of caregiver	Quality of evidence
**Cheng, 2020**	12 RCTs	Dementia	Miscellaneous: physical activity, spiritual care, complementary/alternative therapies (e.g., religious activities, expressive writing).	Usual care or alternative sessions	Depression, anxiety, HR-QoL, physical health	●●●○
**Lee, 2020**	2 RCTs	Dementia or MCI	Complementary and alternative medicine including yoga, massage, and meditation.	No treatment or respite care	HR-QoL	●●○○
**Piersol, 2017**	3 experimental studies	Dementia	Exercise program, adapted leisure program, nighttime monitoring system.	Not reported	Physical health	●●○○
**Irons, 2020**	8 pre-post trials	Neurological diseases	Creative arts interventions, such as music, drama, dance, song writing.	Usual care or not reported	HR-QoL, anxiety, depression	●●○○
**Cuthbert, 2017**	9 RCTs	Various	Physical activity interventions: walking, aerobics, yoga. Varying intensities and formats.	Waiting list, usual care or not reported	Depression, anxiety; well-being, physical strengthening, blood pressure, weight	●●○○
**Doyle, 2020**	5 RCTs, 6 quasi-RCTs	Various	Physical activity dyadic (DyEx) and non-dyadic (DySplit) interventions.	DyEx versus DySplit (i.e., exercising together or not)	Depression, anxiety, physical health	●●○○

Abbreviations: MCI, mild cognitive impairment; RCT, randomized controlled trial; HR-QoL, health-related quality of life.
Legend for quality assessment based on AMSTAR-2: ●●●● High; ●●●○Moderate; ●●○○ Low
A summary of the main findings of each review is available in Supplementary Table 10.

For peer review only

For peer review only - http://bmjopen.bmj.com/site/about/guidelines.xhtml

Mindfulness-based interventions

Four reviews included interventions using mindfulness-based stress reduction techniques (**Table 6 and Supplementary Table 11**).[67,69–71] Three reviews focused on caregivers of persons with dementia,[67,69,70] while one review took interest in caregivers of people with various illnesses.[71] Health-related outcomes varied and included: depression (n=3), anxiety (n=3), and cognition and biomarkers for stress (n=1). Two reviews included meta-analysis.[67,69] Mindfulness-based interventions showed significant positive effects for reducing depressive symptoms and anxiety levels immediately after interventions, but the effects were largely attenuated at follow-up.[69,70] Cheng et al.[67] reported a significant positive effect on depression in grouped meta-analysis. Parkinson et al.[71] reported mixed results, with interventions showing some positive changes in anxiety and depression scores, but with small and waning effects. While some authors acknowledged the importance of potential moderating factors and heterogeneity among caregivers,[70,71] there were no findings to report regarding this question.

For peer review only

For peer review only - http://bmjopen.bmj.com/site/about/guidelines.xhtml

Table 6. Interventions involving mindfulness-based activities.

First author, year	Included primary studies	Disease of care receivers	Characteristics of intervention	Control group	Health outcomes of caregiver	Quality of evidence
**Cheng, 2020**	7 RCTs	Dementia	MBSR and its modifications.	Usual care or alternative sessions	Subjective well-being, depression	●●●○
**Liu, 2018**	5 RCTs	Dementia	MBSR and its modifications.	Usual care or active comparison (respite, social support)	Depressive symptoms, anxiety	●●●●
**Shim, 2020**	9 RCTs	Dementia and MCI	MBSR and its modifications.	Usual care or active comparison (psychoeducation, music-listening)	Cognition, depression, mindfulness, anxiety, biomarkers for stress	●●○○
**Parkinson, 2019**	1 RCT, 5 quasi-RCTs	Various	MBSR and its modifications.	Usual care or not reported	Anxiety	●●○○

Abbreviations: MBSR, Mindfulness-Based Stress Reduction; MCI, mild cognitive impairment.
Legend for quality assessment based on AMSTAR-2: ●●●● High; ●●●○Moderate; ●●○○ Low
A summary of the main findings of each review is available in Supplementary Table 11.

For peer review only

For peer review only - http://bmjopen.bmj.com/site/about/guidelines.xhtml

Comparison across categories of interventions

Three reviews compared the effect sizes across different types of interventions.[47,67,68] Lee et al.[68] provided a separate meta-analysis on multicomponent interventions (including social support, education, and skills-building), which showed the largest positive effect on health-related quality of life compared to single-component interventions. Similarly, Cheng et al.[67] reported that multicomponent and miscellaneous interventions had the strongest effects on depressive symptoms. However, Akarsu et al.[47] reported that effect sizes across multicomponent, psychological and educational interventions are broadly similar.

Main findings: qualitative reviews

For the qualitative part of our umbrella review, we extracted and analyzed data from 18 reviews. **Supplementary Table 4** describes the steps undertaken to apply mega-aggregation framework synthesis[31] to our data. The results of our convergent synthesis of quantitative and qualitative evidence are presented in **Figure 2.** Two themes were identified based on the findings from the 18 reviews providing qualitative data: (1) intervention outcomes and (2) implementation outcomes. These two themes reflect two main domains of intervention implementation research.[72] A detailed list of all fourth-order constructs, accompanying the third-order constructs (*verbatim* text) and references is available in **Supplementary Table 12**.

Theme 1: Intervention outcomes

This theme described the potential benefits and/or harmful effects to caregivers from participating in the interventions (i.e., efficacy and effectiveness), and was informed by 13 reviews.[33,46,52,59,60,66,71,73–78] Only two reviews[71,73] reported health-related benefits, namely decreased depression and anxiety[71,73] and better quality of life.[73] Twelve reviews documented on social outcomes of interventions, i.e., outcomes related to the caregiver's social life, day-to-day routine and relationship with care receivers and other people.[33,46,52,59,60,66,73–78] Based on these reviews, we divided social outcomes into three sub-categories: practical, emotional, and relational outcomes.

Practical outcomes were explored in nine reviews[33,52,59,60,66,73–76] examining how support interventions could introduce changes in the caregivers' day-to-day lives. According to caregivers, interventions were effective in providing them with new knowledge that enhanced coping, ensured better management of burden, and improved their caregiving skills.[59,73,74] Acquired skills increased their readiness for care, allowed them to build strategies to solve problems, and gave them a sense of normality.[52,66,73,74,76] Additionally, respite care was found by Shaw et al.[60] to "*give structure to the carer's week along with a sense of normality*" as the caregiver was able to "*match the ebb and flow of caregiving activities*." However, occasionally, there were additional burdens related to respite care use: "*many hassles [...] involved in the preparation for respite care"* that would lead to loss of *"the physical and emotional energy*."

Participation in the interventions also brought about emotional outcomes, which were described by nine reviews.[46,52,60,66,73,75–78] Across several reviews, caregivers reflected on the positive impacts of talking about their daily arduous challenges, sharing experiences, or simply providing a moment to escape from their various duties through singing, arts or just chit-chat.[52,66,76,78] These positive impacts included gaining a sense of relief, better coping with stress, enjoyment, reduced social isolation and gaining emotional support.[46,52,66,76–78] Lins et al.[52] highlighted that even the caregivers in the control group of the intervention, where participants had "*conversations only about general topics such as the weather, television, movies, news, or social activities*", reported the social interactions to be a "*helpful alternative to relieve carers*." However, some reviews described negative emotional consequences to the participation in interventions. Shaw et al.[60] argued that the physical break due to respite was "*not sufficient in itself to provide the mental break that was needed by most carers to improve their well-being*." It would require that the participants had "*total disengagement from the caring role*"[60] Moreover, they described that

For peer review only - http://bmjopen.bmj.com/site/about/guidelines.xhtml

the use of respite care can be perceived as selfish by the caregiver and bring about feelings of guilt.[60] Irons et al.[66] asserted that, while creative arts interventions may increase positive feelings, negative feelings might not be completely removed.

Finally, four reviews investigated the relational outcomes of support interventions, namely how these interventions modified the caregivers' relationships with their care receiver or peers.[66,75–77] Dyadic interventions were reported to promote increased engagement and deepened relationships between the caregiver and the care receiver.[66,76] The practice of creative arts like singing, viewing art, writing music, or creating memory albums brought a sense of reciprocity, fostered better communication, allowed for seeing the care receiver under a new light, and improved quality time.[66,76] Du Preez at al.[75] also reported increased engagement between caregivers and care receivers after returning from respite day care services.

Theme 2: Implementation outcomes

A total of 17 included reviews examined to what extent the adoption of effective interventions was determined by implementation-related barriers and facilitators that could render them ineffective in certain circumstances.[72,79] Following the classification of Proctor et al.[79] and Hull et al.[80], we synthesized the findings from these reviews in terms of (1) acceptability, (2) feasibility, (3) appropriateness, (4) sustainability, and (5) implementation costs.

Acceptability. Although the included reviews covered studies with varying designs and heterogenous interventions, we found similarities in the description of the determinants of perceived caregiver acceptability. First, seven reviews spanning almost all intervention types demonstrated that the *person-centeredness, flexibility and personalization* of the interventions promoted higher acceptability as expressed by caregivers.[33,34,46,60,66,81,82] Caregivers appreciated if interventions were able to accommodate their lifestyles and needs.[33,34,46,66,81] Some caregivers might prefer face-to-face meetings compared to online meetings[33] while others might appreciate a self-paced program.[46] Additionally, caregivers expressed more favorability towards interventions when the deliverer considered not only their preferences, but also those of the care receiver, including their medical conditions, availability, and commitment level.[33,46,60,66,82] In a review focused on respite[60] caregivers highlighted accepting or rejecting the interventions through the lens of care receivers, and whether aspects ranging from their physical and mental health to their cooperation and approval of participation were fully considered by deliverers. Manifestations of lack of patient-centeredness affected care receivers not only during the period of respite, but also after the respite, as it took some time for care receivers to recover. Along the same lines, Miles et al.[82] indicated that caregivers were positive towards the use of patient and caregiver information and support services but suggested that *"there is not a one-size-fits-all approach which can be used, as every patient […] and their carers will have different needs, preferences and responses*."

Second, three reviews document the importance of cultural and linguistic aspects in explaining the success of support interventions,[33,52,60] in particular whether interventions were able accommodate the needs and values of different ethnic and religious caregiver groups. Language was a barrier for those who did not speak the national language that the intervention was delivered in.[52] This prevented minorities and those who did not have a full grasp of the language from participating, especially if the intervention required advanced understanding and usage of the language, such as counselling.[52] Lins et al. also explained that "*receiving counselling in the native language was also shown to contribute to building trust*." Additionally, having multicultural staff facilitated the acceptability of the interventions because better relationships could be built with the families, and the needs of the different communities could be better understood.[33,60] For example, in the use of in-home respite, minority groups would prefer working with same gender and ethnicity personnel, who also spoke the same language.[60] Ensuring the appropriateness of food based on religious restrictions, and being mindful of cultural and religious differences were also highlighted in the same review.[60]

For peer review only - http://bmjopen.bmj.com/site/about/guidelines.xhtml

Third, six reviews examined how the physical, social, and structural characteristics of interventions promoted or hindered their acceptability by informal caregivers and care receivers.[46,60,66,74–76] Settings that facilitated communication between caregivers and receivers seemed to provide caregivers with a feeling of having a ‘special place’ and thus encouraged their engagement with the intervention.[66,74] On the other hand, online-based interventions were sometimes perceived by caregivers as implying technical difficulties and hindering rapport with the staff.[46] In other words, caregivers expressed their preference for certain delivery methods, as they better suited their needs. For example, in-home respite care was seen as less disruptive compared to day care.[60] Moreover, high rates of staff turnover were reported to disrupt the continuity of care.[60] Du Preez et al.[75] asserted caregiver concerns related to a lack of knowledge about activities taking place at respite day care : “*family carers have little to no contact with the adult day service other than to ready their care recipient for the day's attendance and have little knowledge of how their care recipient spends their time while attending adult day service*.”

Finally, five reviews built on qualitative findings to address the role of trust, dignity and ethical values in the degree of acceptability of support interventions.[52,60,66,75,81] Caregivers seemed to appreciate the intervention staff who they knew well, and were reluctant to accept the advice from those who they had never met.[52,81] Additionally, caregivers appraised the emotional attitude of the staff and the latter's investment in their case.[52] The more familiar the staff was with the case and needs of the caregivers, the more appreciated the intervention was.[52,81] Moreover, respecting the privacy of caregivers and receivers[60,75] and treating care receivers with care and dignity were especially valued by caregivers.[52,60,66,75] The moral values embodied by the staff were also important for caregivers.[66]

Feasibility. Intervention feasibility is defined by Proctor et al.[79] as “the extent to which a new intervention can be successfully used or carried out within a given setting. [...] It is invoked as a potential explanation of an intervention's success or failure, as reflected in recruitment, retention, or participation rates”. Feasibility of support interventions was explored in 13 of the 18 qualitative reviews included in our umbrella review. We further divided this category into five dimensions: (1) recruitment, (2) accessibility, (3) availability, (4) adoption and retention, and (5) systemic factors.

First, challenges to *recruitment* were mentioned in four reviews.[46,60,75,81] Awareness about the availability of interventions was indeed deemed essential for ensuring participation.[60,75] Du Preez et al.[75] explained that “*medical practitioners were identified as having limited knowledge of community support services and access to information resulting in poor referral processes and therefore, poor utilization by family carers and people living with dementia*”. In other words, the lack of knowledge about these services at the primary care level was highlighted by caregivers across reviews.[60,75] Yet, the most preferred and accessible location for dissemination and advice on support services was precisely the primary care center, as expressed by caregivers.[60]

Second, the feasibility of interventions was questioned in terms of their *accessibility* by six reviews,[33,46,52,60,75,76] which examined how both physical and non-physical external factors were potentially affecting the degree of accessibility of various interventions. For instance, some interventions excluded care receivers if they did not have a confirmed diagnosis,[33] while others, like singing and creative arts, included care receivers with diverse stages of disease.[76] Additionally, intervention sites were sometimes described as being “*too far away*”, with no available or reasonable transportation to reach the site.[60,75] In such cases, the use of alternative methods like telephone-based interventions were reported to be useful, as they avoided the hustle of transportation.[52]

Third, *service availability*—that is, the coverage of support interventions in terms of time schedules and availability of staff—was broached in six reviews.[52,60,61,76,78,81] Caregivers across several studies expressed the need for the interventions to be available outside working hours, to include weekends, and even up to 24/7 availability in the case of counselling.[52,60,76,81] The availability outside of working hours enables usage of the interventions by

For peer review only - http://bmjopen.bmj.com/site/about/guidelines.xhtml

working caregivers.[60] Alternative measures, such as the use of answering machines outside counselling hours, were perceived as insufficient.[52]

Fourth, we found five reviews that looked at aspects related to *adoption and retention*, i.e., what factors affect the initiation and the continued use of the services provided as part of an intervention.[34,46,59,60,75] The use of internet-based interventions was sometimes accompanied by technical difficulties that increased the risk of dropping out, especially among older caregivers.[34,46] Sin et al.[46] reported that for internet-based psychoeducation interventions, "*usability problems (such as oral communication/chat quality, audio-visual function failure) were also identified as attributing to high drop-out rates (up to 50%) in some studies*". Additionally, with the progression of care receivers' disease, some caregivers reported not being able to leave the care receivers alone in respite care, leading to withdrawal from the interventions.[60,75] On the other hand, flexible, multicomponent and holistic approaches addressing the complex needs of caregivers and care receivers resulted in higher utilization rates.[60,75]

Finally, four reviews investigated *systemic factors* (i.e., health and social care system features that can affect the delivery and utilization of support interventions).[33,73,75,81]Caregivers reported that their experience of not feeling prioritized by the staff over system-related factors hindered the use of interventions.[75] Additionally, caregivers found it difficult to retain all the information and coordinate with different practitioners and institutions.[81] They expressed the need for a central source of information to consult with.[73] Having several providers to coordinate with was linked to other problems according to caregivers, like competition for delivery or lack of involvement of the other providers.[33]

Appropriateness. This category, which was documented in nine reviews[33,34,52,60,66,71,73,76,81] is defined by Proctor et al.[79] as the perceived suitability and usefulness of interventions to address the needs of caregivers. The appropriate delivery of interventions was largely dependent on the adoption of a patient-centered approach, and the existence of a multiagency and inter-organizational cooperation to address the specific needs of the caregivers.[33,34] However, it was emphasized that not all caregivers would benefit from all interventions.[34,66,71,76] As caregivers' needs and preferences differ, intervention components should be tailored to each case.[33,34,73] This referred to the type of intervention (e.g., psychoeducational, relaxation, etc.), the delivery mode (e.g., via phone, in person), the setting (e.g., at home, in the clinic), and type of participation (e.g., individual, in groups, dyadic).[33,34,73] Pritchard et al.[73] asserted that providing "*appropriate modality and timing of information*" to caregivers requires "*information to be presented in different ways (e.g., in writing, diagrams) repeated on several occasions and in person, not over the phone*". Additionally, some interventions such as counselling were deemed more needed during specific times, for example during crises or in acute conditions.[52] Moreover, caregivers did not always find dyadic interventions that were effective for care receivers suited to their needs.[66,76]

Sustainability. This category grouped those factors associated with a sustained, long-term use of the intervention[79] and was based on three reviews.[59,60,78] The sustainability of the interventions was claimed to depend on the needs and experiences of caregivers.[60] Smith et al.[59] described that caregivers who had encountered peers with similar experiences were more likely to continue the peer support after the intervention had ended. The fact that mindfulness-based exercises can be practiced anytime and are not limited to a certain setting seemed to facilitate its continuous use.[78] Shaw et al.[60] reported that the opportunistic use of respite care could lead to a more regular use once its potential benefits had been experienced.

Implementation costs. This category was linked to the financial costs associated with implementing or using the intervention[79] and was developed based on four reviews.[33,46,60,75] In general, interventions that were not provided free of charge made them less accessible, as affordability differed between individuals. This barrier was reported from reviews that included case management,[33] psychoeducational,[46] and respite care interventions.[60,75]

For peer review only - http://bmjopen.bmj.com/site/about/guidelines.xhtml

Search update in January 2023

The literature search was updated by the university librarians to capture reviews published between 26 March 2021 (end of our initial search) and 31 January 2023. This search yielded 1920 additional entries. A single reviewer completed title/abstract screening, leading to 57 potentially relevant reviews. After a thorough assessment of the full-text articles, a total of 26 reviews were found to meet all eligibility criteria. Finally, we excluded 14 reviews of critically low quality. The 12 remaining reviews are listed and summarized in **Table 7**. Their key findings and recommendations are in line with the main findings of our umbrella review.

For peer review only

Table 7. Summary of the new reviews published between March 2021 and January 2023

First author, year	Title	Design(s)	Included primary studies	Main findings and health outcomes
**Andrades-Gonzalez, 2021[83]**	e-Health as a tool to improve the quality of life of informal caregivers dealing with stroke patients: Systematic review with meta-analysis	Quantitative	12 RCTs	Findings across studies are heterogeneous. However, approximately two thirds of the studies that were part of the meta-analysis showed a decrease in depressive symptoms and a substantial improvement in the quality of life with the use of e-Health. Measures on physical health were either inconclusive or non-significant.
**Boyt, 2022[84]**	Internet-facilitated interventions for informal caregivers of patients with neurodegenerative disorders: Systematic review and meta-analysis	Quantitative	20 RCTs, 31 pre-post evaluative studies	Internet-delivered interventions were superior in reducing anxiety, compared to controls. Findings were inconclusive for quality-of-life outcomes. Ten studies reported depression outcomes. The random-effects meta-analysis demonstrated that there was no significant difference between groups at post-intervention measurement.
**Crocker, 2022[85]**	Information provision for stroke survivors and their carers: Cochrane review	Quantitative	12 new RCTs (update)	Authors are uncertain whether active information provision reduces or increases cases of carer anxiety, however it might slightly reduce anxiety symptoms. Findings on depression are similarly inconclusive. Active information provision may have little to no effect on carer quality of life.
**Garnett, 2022[86]**	mHealth interventions to support caregivers of older adults: Equity-focused systematic review	Both	14 experimental, 7 qualitative, 7 mixed methods	mHealth interventions were positively received by study participants. Impacts on caregivers' mental and psychological health status were generally positive. Some participants reported challenges associated with participation; for example, interventions were too complex or difficult to understand, interventions included questions that were overly obtrusive or confronting, while some questions triggered painful memories. Some participants prefered in-person interventions.
**Ghosh, 2022[87]**	Systematic review of dyadic psychoeducational programs for persons with dementia and their family caregivers	Qualitative	1 qualitative study involving multiple case studies	Dyadic psychoeducational programs that were goal oriented and tailored to address individual needs had consistent benefits on various aspects of health and quality of life for the dyads. Findings on caregivers' physical and mental health outcomes were inconclusive, with similar numbers of studies reporting positive and non-significant effects.
**He, 2022[88]**	The effectiveness of multi-component interventions on the positive and negative aspects of well-being among informal caregivers of people with dementia: A systematic review and meta-analysis	Quantitative	31 RCTs	Meta-analyses showed small to moderate effects on depression, and a moderate to high effect on caregiver anxiety. This review suggests that individualized multicomponent interventions for caregivers may be one of the ways to promote their well-being.
**Kusi, 2022[89]**	The effectiveness of psychoeducational interventions on caregiver-oriented outcomes in caregivers of adult cancer patients: A systematic review and meta-analysis	Quantitative	28 controlled trials	Psychoeducational interventions had beneficial effects on depression, anxiety and quality of life at the immediate post-intervention period. At longer-term follow-up, the effectiveness of interventions was maintained on quality of life and anxiety, but not on depression.
**Mårtensson, 2023[90]**	Psychological interventions for symptoms of depression among informal caregivers of older adult populations: A systematic review and meta-analysis of randomized controlled trials	Quantitative	15 controlled trials	A small effect size favoring the intervention was found for symptoms of depression, and interventions were effective in reducing incidence of major depression and psychological distress. Authors warn that, given the high heterogeneity and high risk of bias, findings should be interpreted with caution.

For peer review only - http://bmjopen.bmj.com/site/about/guidelines.xhtml

**Sun, 2022[91]**	Comparative efficacy of 11 non-pharmacological interventions on depression, anxiety, quality of life, and caregiver burden for informal caregivers of people with dementia: A systematic review and network meta-analysis	Quantitative	85 RCTs	Acceptance and commitment therapy, behavioral activation, mindfulness-based intervention, multicomponent intervention, psychoeducation, and cognitive behavioral therapy might reduce depression. Notably, psychoeducation was the only effective intervention against anxiety. Only support groups had a statistically significant effect on the quality of life.
**Thompson, 2021[92]**	How singing can help people with dementia and their family care-partners: A mixed studies systematic review with narrative synthesis, thematic synthesis, and meta-integration	Both	26 experimental, 9 qualitative, 5 mixed methods	Results from the syntheses suggest that singing can positively impact the lives of people with dementia and their care-partners, although due to heterogeneity of study design and outcome measures, it is difficult to draw conclusions based on quantitative data alone. Qualitative data provides further context and insights from participant perspectives. For instance, participants report enjoyment, improvement in mood, social belonging and dyadic relationship.
**Wallace, 2021[93]**	Do caregivers who connect online have better outcomes? A systematic review of online peer-support interventions for caregivers of people with stroke, dementia, traumatic brain injury, Parkinson's disease and multiple sclerosis	Qualitative	7 mixed methods, 4 case series	Overall, participants responded positively to the psychosocial elements of the interventions. Some participants felt less lonely and more supported, while others noted that they found reading other users' posts distressing or felt that sharing their story with others was a betrayal to their family members. Participants identified convenience as a major benefit of the online platform, noting that it reduced the need to travel, take time off work or leave vulnerable family members on their own. Anonymity was identified as both a benefit and disadvantage to the use of online platforms.
**Watt, 2022[94]**	Systematic review of group-based creative arts interventions in support of informal caregivers of adults: a narrative synthesis	Qualitative	12 qualitative, 7 mixed methods	Positive themes emerging from qualitative data included: creative arts as unique, enjoyable and supporting expression, meaningful connection and support between caregivers, and a positive impact on dyad relationship. Some participants identified barriers related to interventions, such as emotional exhaustion, getting upset, not enough time to complete the activity, burden of caring and difficulty getting to the art gallery.

Abbreviations: RCT, Randomized Controlled Trial.

For peer review only - http://bmjopen.bmj.com/site/about/guidelines.xhtml

DISCUSSION

Summary of findings

Addressing the negative health outcomes of informal caregiving is a major challenge. In this umbrella review on support interventions for people providing informal care to older adults, we synthesized data from 47 systematic reviews covering 619 distinct primary studies. This is, to date, the most comprehensive map of the available evidence. Four main conclusions stem from our analysis.

First, whether existing interventions are effective at reducing the negative impact of caregiving on the physical and mental health of caregivers remains uncertain. Quantitative reviews provided largely discordant findings, with reviews rated as being at low risk of bias reporting trivial or no benefits.[33,38,51,63,69] Also, systematic reviews that included a meta-analysis were more likely to report a lack of effectiveness. While some case management, psychosocial and mindfulness interventions with more than one follow-up timepoint seemed to demonstrate short-term benefits, their positive effect waned as time elapsed.[38,44,69–71] Qualitative reviews provided only limited insight: although informal caregivers mentioned social and practical benefits,[52,66,73–77] they rarely spoke about how support interventions impacted their own health.[71,73]

Second, we found that multicomponent interventions showed more consistent positive effects on health outcomes across reviews, despite a large heterogeneity in what these interventions actually entailed.[34,35,67] Moreover, in the two reviews that performed meta-analyses by type of intervention, multicomponent interventions showed the largest effect sizes.[35,67] In this respect, our findings corroborate the conclusions of two previously published umbrella reviews.[18,20]

The third conclusion is that the available evidence on support interventions relies on the simplistic assumption that informal caregivers represent a homogenous target population, with little attention being paid to the variability in caregiver, care receiver, care context and implementation characteristics. Hence, while a number of reviews reported valuable information about the characteristics of the interventions being evaluated,[35,37,39,45,47,51] the socioeconomic and ethnic background of caregivers and care receivers and the nature of their relationship were largely overlooked. Others have emphasized the need to better account for the social determinants of health among informal caregivers.[95] Their diversity goes beyond the obvious differences in the underlying health conditions of the persons their provide care to, and this reality should come under greater scrutiny in future studies designed to assess the effectiveness of support interventions.

Finally, our overview casts new light onto how support interventions are experienced by informal caregivers. By synthesizing the qualitative findings of 18 distinct reviews, we showed that caregivers mention a myriad of social benefits, for instance improved relationships with care receivers, better organized routine, and less stress/burden associated with caregiving.[33,45,52,59,60,66,73–78] Across multiple reviews, caregivers were found to favor flexible, person-centered, and needs-based interventions rather than 'off-the-rack' support services.[33,34,45,60,66,81,82] This serves to further emphasize that one-size-fits-all approaches are unwarranted since different caregivers have different preferences in terms of, among others, mode of delivery and duration of what constitute adequate support. Interestingly, we were able to find evidence across most implementation outcomes highlighted in the framework by Proctor et al.[79] (acceptability, feasibility, appropriateness, sustainability, and cost). However, *fidelity* of the interventions, i.e., the degree to which an intervention was implemented as it was intended, was not reported.

The extensive set of qualitative findings incorporated in this umbrella review shows that support interventions targeting informal caregivers seem to improve a wide range of practical, emotional, and relational outcomes. It is likely that these benefits translate indirectly into positive changes in caregivers' mental and physical health, even though high-quality evidence for this connection was lacking from quantitative reviews. To better visualize this pathway, we organized the findings from our umbrella review into a framework, *From Support Interventions to Improved Caregiver Outcomes (SIICO)*, as presented in **Figure 2**. While relations in this framework are only

For peer review only - http://bmjopen.bmj.com/site/about/guidelines.xhtml

hypothesized and remain untested, it represents an attempt to visualize the numerous mechanisms implicated in previous research that link interventions to caregivers' health. However, our hypothetical connections between the different boxes of the framework should be interpreted with caution and deserve further scrutiny, especially concerning the potential mediating effect and transition from social and practical benefits to the improvement of objective health outcomes. It is possible that the majority of these interventions are only effective on outcomes related to health but not considered herein (e.g., burden, life satisfaction, wellbeing) and on other aspects of caregivers' lives (e.g., ability to reconcile caregiving and employment, volunteering, socializing and/or leisure). It is also possible that the observed social and relational benefits simply do not translate into measurable improvements in health outcomes, or that these improvements remain partly invisible due to methodological issues (e.g., lack of statistical power, suboptimal control groups, inconsistent outcome measurements, or insufficiently long follow-ups).

Beyond its initial goals, our umbrella review identified several important knowledge gaps in caregiver intervention research that, we believe, could serve as a roadmap for future studies in this field. Hence, there was a clear overrepresentation of certain types of interventions (i.e., psychosocial interventions such as cognitive therapies, group or individual support and psychoeducation), care receiver diseases (i.e., dementia), and outcomes (i.e., mental health). Our umbrella review also highlights the overproduction of systematic reviews and meta-analyses on the effectiveness of support interventions for informal caregivers: more than 145 reviews have been published in the last two decades and, as expected, the overlap between them is substantial. This overproduction has become even more evident upon updating our search: between March 2021 and January 2023, a total of 57 additional reviews (compared with 303 for 2000 to 2021) were published. This raises questions about the potential waste of valuable research resources, especially since the overall quality of published reviews was poor. Approximately two-thirds of the reviews that we identified were rated as being of 'critically low' quality. However, one should keep in mind that some of these reviews were carried out before current guidelines and risk-of-bias appraisal tools became mainstream; in fact, more recent reviews demonstrated higher adherence to the such guidelines. Qualitative synthesis and reporting practices were often subpar, with important contextual and methodological items missing from an unexpectedly large share of included reviews. The lack of consensus regarding the classification of support interventions was yet another struggle: much like Gaugler et al.,[30] we found that not only were certain types of interventions described differently across primary studies, but even the same primary studies (i.e., same interventions) were sometimes put into different categories from one review to another. This hinders the comparability of findings across primary studies, reviews, and umbrella reviews, and makes any attempt at drawing robust conclusions about the effectiveness of interventions challenging. Others have already mentioned these incoherencies, together with other methodological shortcomings such as incomplete reporting in the included primary studies, which has an inevitable ripple effect on the degree of completeness of reviews.[20,21,96] Finally, the fact that our findings are mostly inconclusive despite an abundance of published literature highlights the methodological flaws that afflict a large number of primary studies. We believe that this should prompt a discussion between academics, stakeholders and public funding agencies. Maybe it is now time to take the advice from English methodologist Doug Altman seriously: we need less research, better research, and research done for the right reasons.[97]

Implications for public health and practice

Against the backdrop of staff shortages and budget restrictions worldwide (exacerbated by the ongoing Covid-19 pandemic), certain public health services will need to be prioritized over others. Four main implications for public health and practice emerged from our review. First, better intervention research and evaluation practices are warranted to create an evidence-base for resource allocation: we won't know what works best in different contexts if we keep relying on low-quality studies. Second, time has come for a more targeted approach to intervention design. While caregiver interventions may not work for the 'average caregiver', targeting high-risk groups—e.g., caregivers with preexisting conditions, multimorbidity or frailty—might deliver more convincing and cost-effective results. Third, since our healthcare systems are already under considerable pressure, support interventions should build on existing resources rather than entirely new services. To our surprise, very few of the interventions included in the systematic reviews and meta-analyses that we synthesized mustered primary care professionals to support informal caregivers.

For peer review only - http://bmjopen.bmj.com/site/about/guidelines.xhtml

Yet, family physicians and home care nurses (among others) have frequent contacts with caregivers, with whom they often share a long-lasting relationship. They should have the means to remain vigilant, identify high-risk subgroups of caregivers and enlist them in relevant support programs. Fourth, our umbrella review also highlights the potential for non-profit organizations: trained volunteers could, for instance, take a larger role in delivering social care services to alleviate the burden of caregivers.

Implications for research

Our findings may be useful for decision makers trying to untangle the state of the evidence on this complex topic, but also for those interested in more specific intervention types, given the broadness of our review. There are moreover several implications for future research stemming from our umbrella review. First, the enforcement of *a priori* protocol registration could avoid the wasteful production of reviews. Second, the consistent application of systematic review guidelines (e.g., PRISMA guidelines, Cochrane methods, guidance by Joanna Briggs Institute) is warranted to ensure higher quality of evidence synthesis. Third, at the primary study level, a better definition of intervention components and a clear harmonization of intervention types is needed to ensure comparability of the generated evidence. Last, future interventions should identify and report results across subgroups defined by caregiver, care receiver and care context characteristics, and consider including not only social and relational outcomes, but also objective mental and physical health outcomes, measured comprehensively and over an extended period of time. This will be essential to better understand the potential pathways connecting social to physical health outcomes, although the latter will require a mixed-method evaluation approach.

Strengths and limitations

The primary strength of this umbrella review is the rigorous study selection process, initiating from a generous search strategy identifying over six thousand abstracts. Such process was completed in duplicate and included quality and overlap assessment. Further, we included both quantitative and qualitative evidence to better understand the complex phenomenon of caregiver support interventions and their impact on health. In addition, data synthesis and framework elaboration were guided by existing theory and expertise within the group, adding to the rigor of the review. However, umbrella reviews are limited in terms of drawing conclusive statements, given that they do not assess primary studies, nor re-test meta-analyses provided by single systematic reviews. Thus, the interpretability of our findings is limited by the quality and conclusions of included reviews, which are considerably heterogeneous across reviews. In addition, recent primary studies on new interventions may not be captured, as they may not yet have been included in systematic reviews. Finally, our umbrella review focused on caregiver-centered interventions and, thus, could not shed light on the potentially positive spillover effect of formal care services delivered to patients on informal caregivers' health. Yet, the importance of well-organized and sufficiently staffed professional services for household chores and personal care should not be underestimated, and the expansion of support services for informal caregivers cannot come to the detriment of formal care.

Conclusions

Despite an abundance of systematic reviews, whether certain support interventions are effective at improving informal caregivers' physical and/or mental health is uncertain due to a lack of high-quality evidence. It seems that multicomponent and flexible interventions are more likely to address the complex needs of caregivers, making them more acceptable and thus leading to more tangible effects on objective health outcomes. To confirm this, we do not need more reviews: we need more carefully designed intervention studies that look at both subjective and objective health outcomes, and account for heterogeneity in caregiving.

For peer review only - http://bmjopen.bmj.com/site/about/guidelines.xhtml

CONTRIBUTORSHIP STATEMENT: ACL obtained the funding and drafted the protocol including the initial methodology for the umbrella review. ACL, LM, MK, LD and LBS participated in further conceptualizing the protocol and critically reviewing it before publication. For the umbrella review, all authors participated in study selection and quality appraisal of the reviews in duplicate. MK and AA extracted and cross-checked data from selected reviews. MK and AA drafted the initial manuscript, which was critically revised several times by ACL, LM, LD and LBS. The review team held several meetings regarding methodology, interpretation of data and desired output of the project. All authors gave final approval to this version of the protocol. All authors agree to be accountable for all aspects of the work in ensuring that questions related to the accuracy or integrity of any part of the work are appropriately investigated and resolved.

COMPETING INTERESTS STATEMENT: None declared.

ETHICS APPROVAL: this review is a tertiary synthesis of published systematic reviews (secondary synthesis). Thus, no ethical approval was sought and no contact was made with participants of primary studies included in systematic reviews.

FUNDING STATEMENT: This work was supported by the Swedish Research Council for Health, Working Life and Welfare (FORTE) grant number 2020-01544.

DATA SHARING STATEMENT: Data sharing is not applicable as no datasets were generated and/or analyzed for this study.

ACKNOWLEDGMENTS: We wish to thank Emma-Lotta Säätelä and Jonas Pettersson, librarians at Karolinska Institutet, for preparing the search strategy and performing the searches across databases.

WORD COUNT: 8176

For peer review only

REFERENCES

1 AARP and National Alliance for Caregiving. Caregiving in the United States. 2020. doi:https://doi.org/10.26419/ppi.00103.001

2 Kingston A, Wohland P, Wittenberg R, *et al.* Is late-life dependency increasing or not? A comparison of the Cognitive Function and Ageing Studies (CFAS). *The Lancet* 2017;**390**:1676–84. doi:10.1016/S0140-6736(17)31575-1

3 Verbeek-Oudijk D, Woittiez I, Eggink E, *et al.* Who cares in Europe? A comparison of long-term care fore the over-50s in sixteen European countries. *The Hague: The Netherlands Institute for Social Research (SCP)* 2014;:1–109.http://www.google.nl/url?sa=t&rct=j&q=&esrc=s&source=web&cd=1&ved=0CCUQFjAA&url=http://www.scp.nl/dsresource?objectid=36940&type=org&ei=J5G_VNHoAoHeUOnpgoAC&usg=AFQjCNGLqTpygPklGLJ6Xsaat5OnvYyHtQ&sig2=X9Db9df5KLvp3NNslz-Gfg&bvm=bv.83829542

4 Haakenstad A, Irvine CMS, Knight M, *et al.* Measuring the availability of human resources for health and its relationship to universal health coverage for 204 countries and territories from 1990 to 2019: a systematic analysis for the Global Burden of Disease Study 2019. *The Lancet* 2022;**399**:2129–54. doi:10.1016/s0140-6736(22)00532-3

5 European Commission. *Informal care in Europe*. 2018.

6 Pickard L. A growing care gap' the supply of unpaid care for older people by their adult children in England to 2032. *Ageing Soc* 2015;**35**:96–123. doi:10.1017/S0144686X13000512

7 Lindt N, Van Berkel J, Mulder BC. Determinants of overburdening among informal carers: A systematic review. *BMC Geriatr* 2020;**20**:1–12. doi:10.1186/s12877-020-01708-3

8 Mello J de A, Macq J, Van Durme T, *et al.* The determinants of informal caregivers' burden in the care of frail older persons: a dynamic and role-related perspective. *Aging Ment Health* 2017;**21**:838–43. doi:10.1080/13607863.2016.1168360

9 Conde-Sala JL, Garre-Olmo J, Turró-Garriga O, *et al.* Differential features of burden between spouse and adult-child caregivers of patients with Alzheimer's disease: An exploratory comparative design. *Int J Nurs Stud* 2010;**47**:1262–73. doi:10.1016/j.ijnurstu.2010.03.001

10 Johansson MF, McKee KJ, Dahlberg L, *et al.* Negative Impact and Positive Value of Caregiving in Spouse Carers of Persons with Dementia in Sweden. *Int J Environ Res Public Health* 2022;**19**. doi:10.3390/ijerph19031788

11 Roth DL, Fredman L, Haley WE. Informal caregiving and its impact on health: A reappraisal from population-based studies. *Gerontologist* 2015;**55**:309–19. doi:10.1093/geront/gnu177

12 Schulz R, Beach SR. Caregiving as a risk factor for mortality: The caregiver health effects study. *J Am Med Assoc* 1999;**282**:2215–9. doi:10.1001/jama.282.23.2215

13 Yuan J, Grühn D. Time Effects of Informal Caregiving on Cognitive Function and Well-Being: Evidence From ELSA. *Gerontologist* 2021;**61**:341–51. doi:10.1093/geront/gnaa114

14 Bom J, Bakx P, Schut F, *et al.* The Impact of Informal Caregiving for Older Adults on the Health of Various Types of Caregivers: A Systematic Review. *Gerontologist* 2019;**59**:e629–42. doi:10.1093/geront/gny137

15 Pinquart M, Sörensen S. Correlates of physical health of informal caregivers: A meta-analysis. *Journals of Gerontology - Series B Psychological Sciences and Social Sciences* 2007;**62**:126–37. doi:10.1093/geronb/62.2.P126

16 Pinquart M, Sörensen S. Gender differences in caregiver stressors, social resources, and health: An updated meta-analysis. *Journals of Gerontology - Series B Psychological Sciences and Social Sciences* 2006;**61**:33–45. doi:10.1093/geronb/61.1.P33

17 Aromataris E, Fernandez R, Godfrey CM, *et al.* Summarizing systematic reviews: Methodological development, conduct and reporting of an umbrella review approach. *Int J Evid Based Healthc* 2015;**13**:132–40. doi:10.1097/XEB.0000000000000055

18 Dickinson C, Dow J, Gibson G, *et al.* Psychosocial intervention for carers of people with dementia: What components are most effective and when? A systematic review of systematic reviews. *Int Psychogeriatr* 2017;**29**:31–43. doi:10.1017/S1041610216001447

19 World Health Organization. Evidence profile: caregiver support. *Integrated Care for Older People: Guidelines on Community-Level Interventions to Manage Declines in Intrinsic Capacity* 2017;:35.

20 Dalton J, Thomas S, Harden M, *et al.* Updated meta-review of evidence on support for carers. *J Health Serv Res Policy* 2018;**23**:196–207. doi:10.1177/1355819618766559

For peer review only - http://bmjopen.bmj.com/site/about/guidelines.xhtml

21 Spiers GF, Liddle J, Kunonga TP, *et al.* What are the consequences of caring for older people and what interventions are effective for supporting unpaid carers? A rapid review of systematic reviews. *BMJ Open* 2021;**11**:1–10. doi:10.1136/bmjopen-2020-046187

22 Calderón-Larrañaga A, Kirvalidze M, Dahlberg L, *et al.* Effectiveness of interventions to address the negative health outcomes of informal caregiving to older adults: protocol for an umbrella review. *PROSPERO CRD42021252841* 2021.

23 Calderón-Larrañaga A, Kirvalidze M, Dahlberg L, *et al.* Effectiveness of interventions to address the negative health outcomes of informal caregiving to older adults: Protocol for an umbrella review. *BMJ Open* 2021;**11**. doi:10.1136/bmjopen-2021-053117

24 Gates M, Gates A, Pieper D, *et al.* Reporting guideline for overviews of reviews of healthcare interventions: The Preferred Reporting Items for Overviews of Reviews (PRIOR) statement. *MetaArXiv* Published Online First: 2022. doi:10.31222/osf.io/82wau

25 Veritas Health Innovation. Covidence systematic review software. www.covidence.org

26 Shea BJ, Reeves BC, Wells G, *et al.* AMSTAR 2: A critical appraisal tool for systematic reviews that include randomised or non-randomised studies of healthcare interventions, or both. *BMJ (Online)* 2017;**358**:j4008. doi:10.1136/bmj.j4008

27 Pieper D, Antoine SL, Mathes T, *et al.* Systematic review finds overlapping reviews were not mentioned in every other overview. *J Clin Epidemiol* 2014;**67**:368–75. doi:10.1016/j.jclinepi.2013.11.007

28 Pérez-Bracchiglione J, Meza N, Bangdiwala SI, *et al.* Graphical Representation of Overlap for OVErviews : GROOVE tool . *Res Synth Methods* 2022;:1–8. doi:10.1002/jrsm.1557

29 Pollock M, Fernandes RM, Newton AS, *et al.* A decision tool to help researchers make decisions about including systematic reviews in overviews of reviews of healthcare interventions. *Syst Rev* 2019;**8**:1–8. doi:10.1186/s13643-018-0768-8

30 Gaugler JE, Jutkowitz E, Shippee TP, *et al.* Consistency of dementia caregiver intervention classification: An evidence-based synthesis. *Int Psychogeriatr* 2017;**29**:19–30. doi:10.1017/S1041610216001514

31 Hendricks L, Eshun-Wilson I, Rohwer A. A mega-aggregation framework synthesis of the barriers and facilitators to linkage, adherence to ART and retention in care among people living with HIV. *Syst Rev* 2021;**10**:1–28. doi:10.1186/s13643-021-01582-z

32 Van Houtven CH, Voils CI, Weinberger M. An organizing framework for informal caregiver interventions: Detailing caregiving activities and caregiver and care recipient outcomes to optimize evaluation efforts. *BMC Geriatr* 2011;**11**. doi:10.1186/1471-2318-11-77

33 Goeman D, Renehan E, Koch S. What is the effectiveness of the support worker role for people with dementia and their carers? A systematic review. BMC Health Serv Res. 2016;**16**. doi:10.1186/s12913-016-1531-2

34 Hopwood J, Walker N, McDonagh L, *et al.* Internet-Based Interventions Aimed at Supporting Family Caregivers of People With Dementia: Systematic Review. *J Med Internet Res* 2018;**20**:e216. doi:https://dx.doi.org/10.2196/jmir.9548

35 Lee M, Ryoo JH, Crowder J, *et al.* A systematic review and meta-analysis on effective interventions for health-related quality of life among caregivers of people with dementia. *J Adv Nurs* 2020;**76**:475–89. doi:https://dx.doi.org/10.1111/jan.14262

36 Lucero RJ, Fehlberg EA, Patel AGM, *et al.* The effects of information and communication technologies on informal caregivers of persons living with dementia: A systematic review. *Alzheimer's and Dementia: Translational Research and Clinical Interventions* 2019;**5**:1–12. doi:10.1016/j.trci.2018.11.003

37 Piersol CV, Canton K, Connor SE, *et al.* Effectiveness of Interventions for Caregivers of People With Alzheimer's Disease and Related Major Neurocognitive Disorders: A Systematic Review. Am J Occup Ther. 2017;**71**:p1-7105180020. doi:10.5014/ajot.2017.027581

38 Reilly S, Miranda-Castillo C, Malouf R, *et al.* Case management approaches to home support for people with dementia. Cochrane Database of Systematic Reviews. 2015;**2017**. doi:10.1002/14651858.CD008345.pub2

39 Greenwood N, Pelone F, Hassenkamp AM. General practice based psychosocial interventions for supporting carers of people with dementia or stroke: a systematic review. *BMC Fam Pract* 2016;**17**:3. doi:https://dx.doi.org/10.1186/s12875-015-0399-2

40 Pucciarelli G, Lommi M, Magwood GS, *et al.* Effectiveness of dyadic interventions to improve stroke patient-caregiver dyads' outcomes after discharge: A systematic review and meta-analysis study. European Journal of Cardiovascular Nursing. 2021;**20**:14–33. doi:10.1177/1474515120926069

For peer review only - http://bmjopen.bmj.com/site/about/guidelines.xhtml

41 Corry M, Neenan K, Brabyn S, *et al.* Telephone interventions, delivered by healthcare professionals, for providing education and psychosocial support for informal caregivers of adults with diagnosed illnesses. *Cochrane Database of Systematic Reviews* 2019;**5**:CD012533. doi:https://dx.doi.org/10.1002/14651858.CD012533.pub2

42 Gabriel I, Creedy D, Coyne E. A systematic review of psychosocial interventions to improve quality of life of people with cancer and their family caregivers. Nurs Open. 2020;**7**:1299–312. doi:10.1002/nop2.543

43 Heckel L, Heynsbergh NL, Livingston PM. Are cancer helplines effective in supporting caregivers? A systematic review. Supportive Care in Cancer. 2019;**27**:3219–31. doi:10.1007/s00520-019-04807-z

44 Treanor CJ, Santin O, Prue G, *et al.* Psychosocial interventions for informal caregivers of people living with cancer. *Cochrane Database of Systematic Reviews* 2019;**2019**. doi:10.1002/14651858.CD009912.pub2

45 Waldron EA, Janke EA, Bechtel CF, *et al.* A systematic review of psychosocial interventions to improve cancer caregiver quality of life. *Psychooncology* 2013;**22**:1200–7. doi:https://dx.doi.org/10.1002/pon.3118

46 Sin J, Henderson C, Spain D, *et al.* eHealth interventions for family carers of people with long term illness: A promising approach? Clin Psychol Rev. 2018;**60**:109–25. doi:10.1016/j.cpr.2018.01.008

47 Akarsu NE, Prince MJ, Lawrence VC, *et al.* Depression in carers of people with dementia from a minority ethnic background: Systematic review and meta-analysis of randomised controlled trials of psychosocial interventions. Int J Geriatr Psychiatry. 2019;**34**:790–806. doi:10.1002/gps.5070

48 González-Fraile E, Ballesteros J, Rueda JR, *et al.* Remotely delivered information, training and support for informal caregivers of people with dementia. Cochrane Database of Systematic Reviews. 2021;**2021**. doi:10.1002/14651858.CD006440.pub3

49 Zabihi S, Lemmel FK, Orgeta V. Behavioural Activation for Depression in Informal Caregivers: A Systematic Review and Meta-Analysis of Randomised Controlled Clinical Trials. *J Affect Disord* 2020;**274**:1173–83. doi:10.1016/j.jad.2020.03.124

50 Wiegelmann H, Speller S, Verhaert LM, *et al.* Psychosocial interventions to support the mental health of informal caregivers of persons living with dementia - a systematic literature review. *BMC Geriatr* 2021;**21**:94. doi:https://dx.doi.org/10.1186/s12877-021-02020-4

51 Forster A, Brown L, Smith J, *et al.* Information provision for stroke patients and their caregivers. *Cochrane Database of Systematic Reviews* Published Online First: 14 November 2012. doi:10.1002/14651858.cd001919.pub3

52 Lins S, Hayder-Beichel D, Rucker G, *et al.* Efficacy and experiences of telephone counselling for informal carers of people with dementia. *Cochrane Database of Systematic Reviews* 2014;:CD009126. doi:https://dx.doi.org/10.1002/14651858.CD009126.pub2

53 Teahan Á, Lafferty A, McAuliffe E, *et al.* Psychosocial Interventions for Family Carers of People With Dementia: A Systematic Review and Meta-Analysis. *J Aging Health* 2020;**32**:1198–213. doi:10.1177/0898264319899793

54 Minshall C, Pascoe MC, Thompson DR, *et al.* Psychosocial interventions for stroke survivors, carers and survivor-carer dyads: a systematic review and meta-analysis. *Top Stroke Rehabil* 2019;**26**:554–64. doi:https://dx.doi.org/10.1080/10749357.2019.1625173

55 Wang S, Bressington DT, Leung AYM, *et al.* The effects of bibliotherapy on the mental well-being of informal caregivers of people with neurocognitive disorder: A systematic review and meta-analysis. *Int J Nurs Stud* 2020;**109**. doi:10.1016/j.ijnurstu.2020.103643

56 Hopkinson MD, Reavell J, Lane DA, *et al.* Cognitive Behavioral Therapy for Depression, Anxiety, and Stress in Caregivers of Dementia Patients: A Systematic Review and Meta-Analysis. *Gerontologist* 2019;**59**:e343–62. doi:https://dx.doi.org/10.1093/geront/gnx217

57 Smith TO, Pearson M, Pfeiffer K, *et al.* Caregiver Interventions for Adults Discharged from the Hospital: Systematic Review and Meta-Analysis. J Am Geriatr Soc. 2019;**67**:1960–9. doi:10.1111/jgs.16048

58 Bennett S, Laver K, Voigt-Radloff S, *et al.* Occupational therapy for people with dementia and their family carers provided at home: a systematic review and meta-analysis. *BMJ Open* 2019;**9**:e026308. doi:https://dx.doi.org/10.1136/bmjopen-2018-026308

59 Smith R, Greenwood N. The impact of volunteer mentoring schemes on carers of people with dementia and volunteer mentors: a systematic review. *Am J Alzheimers Dis Other Demen* 2014;**29**:8–17. doi:https://dx.doi.org/10.1177/1533317513505135

60 Shaw C, McNamara R, Abrams K, *et al.* Systematic review of respite care in the frail elderly. *Health Technol Assess* 2009;**13**:1–224, iii. doi:https://dx.doi.org/10.3310/hta13200

For peer review only - http://bmjopen.bmj.com/site/about/guidelines.xhtml

61 Maffioletti VLR, Baptista MAT, Santos RL, *et al.* Effectiveness of day care in supporting family caregivers of people with dementia: A systematic review. *Dement Neuropsychol* 2019;**13**:268–83. doi:https://dx.doi.org/10.1590/1980-57642018dn13-030003

62 Vandepitte S, Van Den Noortgate N, Putman K, *et al.* Effectiveness of respite care in supporting informal caregivers of persons with dementia: a systematic review. Int J Geriatr Psychiatry. 2016;**31**:1277–88. doi:10.1002/gps.4504

63 Maayan N, Soares-Weiser K, Lee H. Respite care for people with dementia and their carers. Cochrane Database of Systematic Reviews. 2014;**2014**. doi:10.1002/14651858.CD004396.pub3

64 Cuthbert CA, King-Shier K, Ruether D, *et al.* What is the state of the science on physical activity interventions for family caregivers? A systematic review and RE-AIM evaluation. J Phys Act Health. 2017;**14**:578–95. doi:10.1123/jpah.2016-0280

65 Doyle KL, Toepfer M, Bradfield AF, *et al.* Systematic review of exercise for caregiver-care recipient dyads: What is best for spousal caregivers - Exercising together or not at all? Gerontologist. 2021;**61**:E283–301. doi:10.1093/geront/gnaa043

66 Yoon Irons J, Garip G, Cross AJ, *et al.* An integrative systematic review of creative arts interventions for older informal caregivers of people with neurological conditions. PLoS One. 2020;**15**. doi:10.1371/journal.pone.0243461

67 Cheng ST, Li KK, Losada A, *et al.* The effectiveness of nonpharmacological interventions for informal dementia caregivers: An updated systematic review and meta-analysis. *Psychol Aging* 2020;**35**:55–77. doi:https://dx.doi.org/10.1037/pag0000401

68 Lee M, Ryoo JH, Chung M, *et al.* Effective interventions for depressive symptoms among caregivers of people with dementia: A systematic review and meta-analysis. *Dementia* 2020;**19**:2368–98. doi:https://dx.doi.org/10.1177/1471301218822640

69 Liu Z, Sun YY, Zhong BL. Mindfulness-based stress reduction for family carers of people with dementia. *Cochrane Database of Systematic Reviews* 2018;**8**:CD012791. doi:https://dx.doi.org/10.1002/14651858.CD012791.pub2

70 Shim M, Tilley JL, Im S, *et al.* A Systematic Review of Mindfulness-Based Interventions for Patients with Mild Cognitive Impairment or Dementia and Caregivers. J Geriatr Psychiatry Neurol. 2021;**34**:528–54. doi:10.1177/0891988720957104

71 Parkinson B, Lawrence M, McElhinney E, *et al.* Mindfulness for people with long-term conditions and their family caregivers: A systematic review. Complement Ther Clin Pract. 2019;**34**:76–86. doi:10.1016/j.ctcp.2018.10.019

72 Damschroder LJ, Aron DC, Keith RE, *et al.* Fostering implementation of health services research findings into practice: A consolidated framework for advancing implementation science. *Implementation Science* 2009;**4**:1–15. doi:10.1186/1748-5908-4-50

73 Pritchard E, Cussen A, Delafosse V, *et al.* Interventions supporting caregiver readiness when caring for patients with dementia following discharge home: A mixed-methods systematic review. *Australas J Ageing* 2020;**39**:e239–50. doi:https://dx.doi.org/10.1111/ajag.12765

74 Boots LM, de Vugt ME, van Knippenberg RJ, *et al.* A systematic review of Internet-based supportive interventions for caregivers of patients with dementia. *Int J Geriatr Psychiatry* 2014;**29**:331–44. doi:https://dx.doi.org/10.1002/gps.4016

75 Du Preez J, Millsteed J, Marquis R, *et al.* The Role of Adult Day Services in Supporting the Occupational Participation of People with Dementia and Their Carers: An Integrative Review. *Healthcare* 2018;**6**:8. doi:https://dx.doi.org/10.3390/healthcare6020043

76 Bourne P, Camic PM, Crutch SJ. Psychosocial outcomes of dyadic arts interventions for people with a dementia and their informal caregivers: A systematic review. Health Soc Care Community. 2021;**29**:1632–49. doi:10.1111/hsc.13267

77 Dam AE, de Vugt ME, Klinkenberg IP, *et al.* A systematic review of social support interventions for caregivers of people with dementia: Are they doing what they promise? *Maturitas* 2016;**85**:117–30. doi:https://dx.doi.org/10.1016/j.maturitas.2015.12.008

78 Li G, Yuan H, Zhang W. The Effects of Mindfulness-Based Stress Reduction for Family Caregivers: Systematic Review. Arch Psychiatr Nurs. 2016;**30**:292–9. doi:10.1016/j.apnu.2015.08.014

79 Proctor E, Silmere H, Raghavan R, *et al.* Outcomes for implementation research: Conceptual distinctions, measurement challenges, and research agenda. *Administration and Policy in Mental Health and Mental Health Services Research* 2011;**38**:65–76. doi:10.1007/s10488-010-0319-7

80 Hull L. Implementation science research development tool (ImpRes).

For peer review only - http://bmjopen.bmj.com/site/about/guidelines.xhtml

81 Bunn F, Goodman C, Pinkney E, *et al.* Specialist nursing and community support for the carers of people with dementia living at home: An evidence synthesis. *Health Soc Care Community* 2016;**24**:48–67. doi:10.1111/hsc.12189
82 Miles L, McCausland BMS, Patel HP, *et al.* A systematic review of the provision and efficacy of patient and carer information and support (PCIS) interventions for patients with dementia and their informal carers. Aging Clin Exp Res. 2020;**32**:2439–48. doi:10.1007/s40520-019-01428-8
83 Andrades-González I, Romero-Franco N, Molina-Mula J. e-Health as a tool to improve the quality of life of informal caregivers dealing with stroke patients: Systematic review with meta-analysis. *Journal of Nursing Scholarship* 2021;**53**:790–802. doi:10.1111/jnu.12699
84 Boyt N, Ho AK, Morris-Bankole H, *et al.* Internet-facilitated interventions for informal caregivers of patients with neurodegenerative disorders: Systematic review and meta-analysis. *Digit Health* 2022;**8**. doi:10.1177/20552076221129069
85 Crocker TF, Brown L, Lam N, *et al.* Information Provision for Stroke Survivors and Their Carers: Cochrane Review. *Stroke* 2022;**53**:E269–70. doi:10.1161/STROKEAHA.121.038162
86 Garnett A, Northwood M, Ting J, *et al.* mHealth Interventions to Support Caregivers of Older Adults: Equity-Focused Systematic Review. *JMIR Aging* 2022;**5**:1–24. doi:10.2196/33085
87 Ghosh M, Dunham M, O'Connell B. Systematic review of dyadic psychoeducational programs for persons with dementia and their family caregivers. *J Clin Nurs* 2022;:1–21. doi:10.1111/jocn.16570
88 He J, Wang J, Zhong H, *et al.* The Effectiveness of Multi-Component Interventions on the Positive and Negative Aspects of Well-Being among Informal Caregivers of People with Dementia: A Systematic Review and Meta-Analysis. *Int J Environ Res Public Health* 2022;**19**. doi:10.3390/ijerph19126973
89 Kusi G, Atenafu EG, Boamah Mensah AB, *et al.* The effectiveness of psychoeducational interventions on caregiver-oriented outcomes in caregivers of adult cancer patients: A systematic review and meta-analysis. *Psychooncology* 2022;:189–202. doi:10.1002/pon.6050
90 Mårtensson E, Coumoundouros C, Sörensdotter R, *et al.* Psychological interventions for symptoms of depression among informal caregivers of older adult populations: A systematic review and meta-analysis of randomized controlled trials. *J Affect Disord* 2023;**320**:474–98. doi:10.1016/j.jad.2022.09.093
91 Sun Y, Ji M, Leng M, *et al.* Comparative efficacy of 11 non-pharmacological interventions on depression, anxiety, quality of life, and caregiver burden for informal caregivers of people with dementia: A systematic review and network meta-analysis. *Int J Nurs Stud* 2022;**129**:104204. doi:10.1016/j.ijnurstu.2022.104204
92 Thompson Z, Baker FA, Tamplin J, *et al.* How Singing can Help People With Dementia and Their Family Care-Partners: A Mixed Studies Systematic Review With Narrative Synthesis, Thematic Synthesis, and Meta-Integration. *Front Psychol* 2021;**12**. doi:10.3389/fpsyg.2021.764372
93 Wallace SJ, Kothari J, Jayasekera A, *et al.* Do caregivers who connect online have better outcomes? A systematic review of online peer-support interventions for caregivers of people with stroke, dementia, traumatic brain injury, Parkinson's disease and multiple sclerosis. *Brain Impairment* 2021;**22**:233–59. doi:10.1017/BrImp.2021.5
94 Young HM, Bell JF, Whitney RL, *et al.* Social Determinants of Health: Underreported Heterogeneity in Systematic Reviews of Caregiver Interventions. *Gerontologist* 2020;**60**:S14–28. doi:10.1093/geront/gnz148
95 Cheng ST, Zhang F. A comprehensive meta-review of systematic reviews and meta-analyses on nonpharmacological interventions for informal dementia caregivers. *BMC Geriatr* 2020;**20**. doi:10.1186/s12877-020-01547-2
96 Altman DG. The scandal of poor medical research. *BMJ* 1994;**308**:283–4. doi:10.1136/bmj.308.6924.283
97 Page MJ, McKenzie JE, Bossuyt PM, *et al.* The PRISMA 2020 statement: An updated guideline for reporting systematic reviews. *The BMJ* 2021;**372**. doi:10.1136/bmj.n71

For peer review only - http://bmjopen.bmj.com/site/about/guidelines.xhtml

FIGURE LEGEND

Figure 1.
Caption: PRISMA 2020 flow diagram.[98]
Footnote:
*Quantitative reviews with AMSTAR-2 category of "critically low" quality were excluded; Qualitative reviews with two or more critical flaws were excluded.
**Overlap assessment was completed for quantitative and qualitative reviews separately and in distinct subgroups based on support intervention types.
***Out of 47 distinct reviews, 10 contributed to qualitative data only, 8 were included as both qualitative and quantitative data sources, while 29 reviews contributed exclusively to quantitative synthesis.

Figure 2.
Caption: *From Support Interventions to Improved Caregiver Outcomes* (*SIICO)* framework.
Footnote:
The list of potential modifying factors and sources of heterogeneity of intervention effects, such as relationship context, caregiver, and care receiver characteristics, were inspired by Van Houtven et al.'s organizing framework for caregiver interventions and amended by review authors based on expert opinion and review of broader caregiving literature (boxes *Context of the caregiving relationship*; *Caregiver;* and *Care receiver*).
[1]The typology of interventions by Gaugler et al. was reorganized in a data-driven fashion and currently includes six types of interventions (box *Intervention type*).
[2]Based on qualitative findings of our umbrella review, there is evidence that interventions affect practical, emotional, and relational aspects of caregivers' lives (box I*ntervention social outcomes*).
[3]According to mainly quantitative data, some of the interventions might also have effects on caregivers' mental health, physical health, and health-related quality of life (box *Intervention health outcomes*).
[4]Intervention effects are also influenced by implementation outcomes, which were selected based on Procter et al.'s outcomes for implementation research, and derived from qualitative data, as expressed by caregivers (box *Implementation outcomes*).

For peer review only - http://bmjopen.bmj.com/site/about/guidelines.xhtml

For peer review only

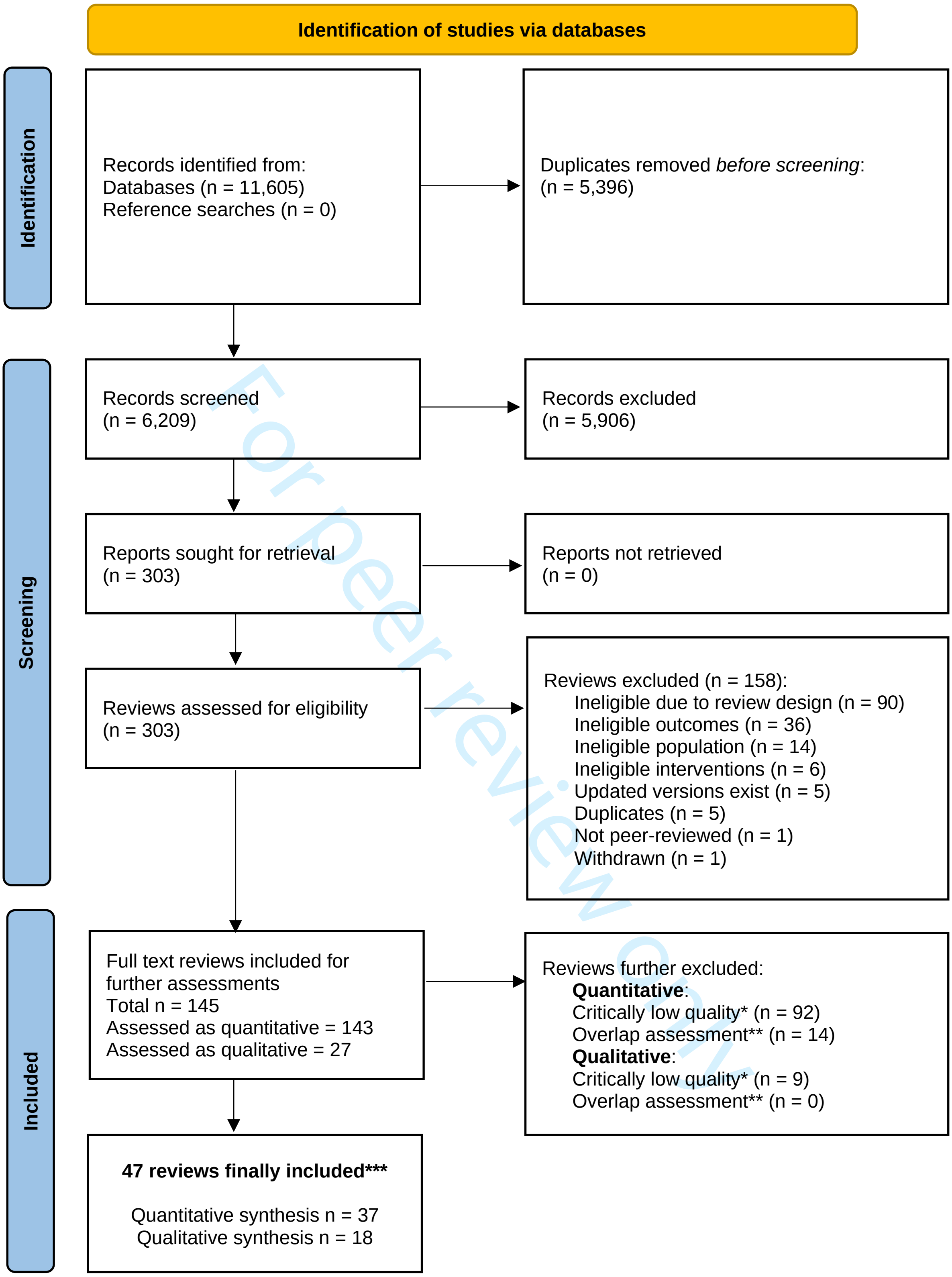

For peer review only

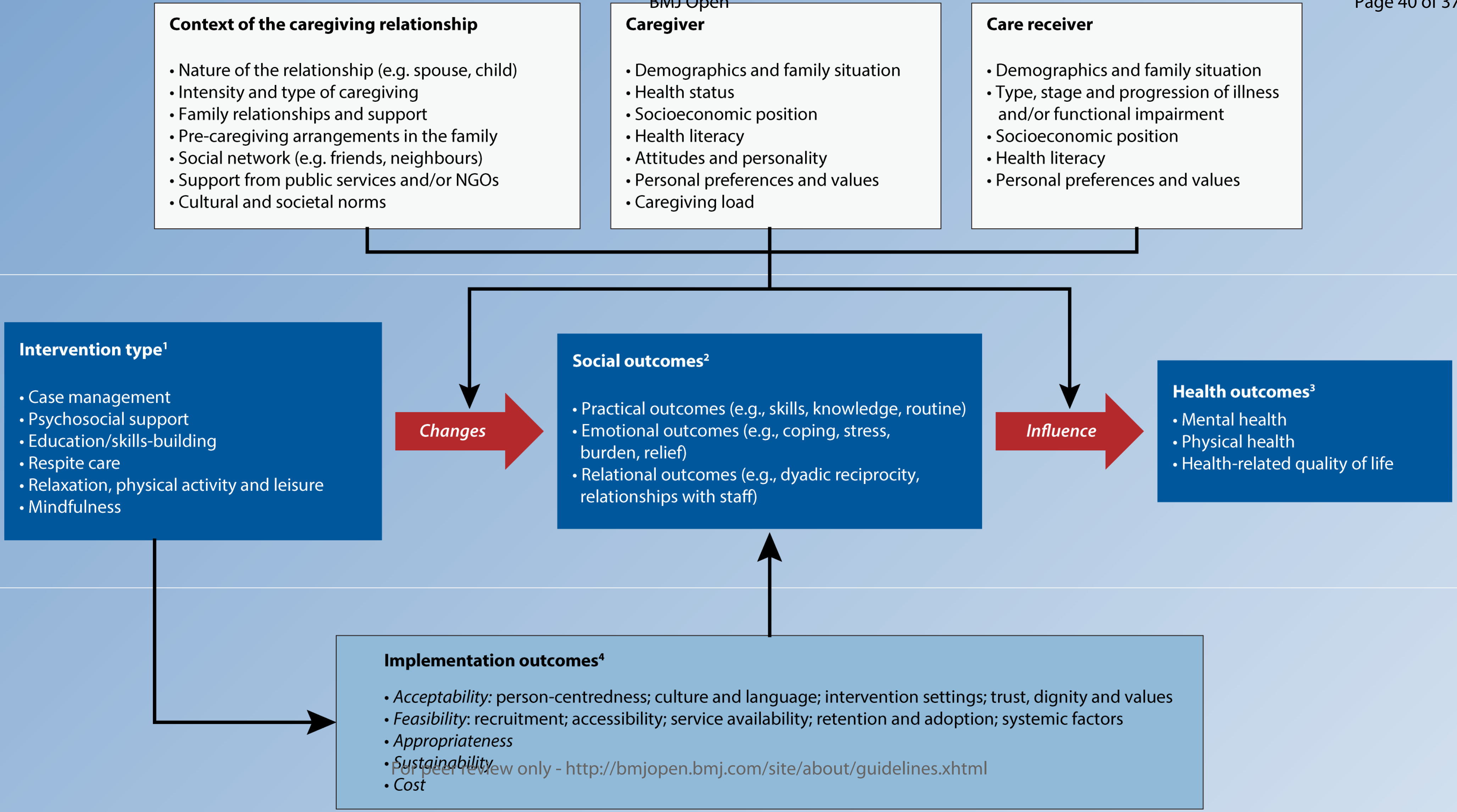

For peer review only - http://bmjopen.bmj.com/site/about/guidelines.xhtml

Supplementary materials

These supplementary materials are provided by the authors to give readers additional information about their study “Effectiveness of interventions designed to mitigate the negative health outcomes of informal caregiving to older adults: an umbrella review of systematic reviews and meta-analyses”.

For peer review only

For peer review only - http://bmjopen.bmj.com/site/about/guidelines.xhtml

Table of Contents

Supplementary Table 1. Amendments made to the published protocol........ **3**

Supplementary Table 2. Search strategies **4**

Supplementary Table 3. Exclusion decisions based on high overlap among quantitative reviews **8**

Supplementary Table 4. Application of Hendricks et al.'s mega-aggregation framework synthesis method **11**

Supplementary Table 5a. AMSTAR-2 quality assessment tool for quantitative reviews........ **12**

Supplementary Table 5b. *Ad hoc* quality assessment tool for qualitative reviews **13**

Supplementary Table 6. Repository of all included reviews (*n*=47) **14**

Supplementary Table 7. Summary of findings for interventions involving case management **25**

Supplementary Table 8. Summary of findings for psychosocial, psychoeducational, and skills-building interventions........ **26**

Supplementary Table 9. Summary of findings for interventions involving respite care **29**

Supplementary Table 10. Summary of findings for interventions involving relaxation, physical activity, or leisure........ **30**

Supplementary Table 11. Summary of findings for interventions involving mindfulness-based activities **31**

Supplementary Table 12. Coding table for qualitative data........ **32**

Additional supplementary materials not included within this document:

- Supplementary File 1 : Excel spreadsheet for GROOVE citation assessment tool: quantitative reviews
- Supplementary File 2 : Excel spreadsheet for GROOVE citation assessment tool: qualitative reviews

For peer review only

Supplementary Table 1. Amendments made to the published protocol (doi:10.1136/bmjopen-2021-053117)

Amendment	Rationale
**Research question 4**<br>*Protocol:* (4) How are the proposed interventions experienced by caregivers in terms of their acceptability, feasibility and added value?<br>*Amended:* (4) How are the proposed interventions experienced by caregivers in terms of effectiveness and implementation outcomes?	We amended the fourth research question based on a data-driven approach to encompass a wider range of implementation outcomes. During the qualitative data extraction and categorization exercise, it became clear that data was richer than originally anticipated. We believe that the current formulation better addresses the complexity of intervention experiences, as reported by caregivers.
**Timeframe**<br>*Protocol:* the review is anticipated to be conducted in the period of 1 April 2021– 31 May 2022.	Due to the volume of work required to finalize the umbrella review, we did not fit in the anticipated timeframe. Instead, the final draft of the report was completed in late June of 2022.
**Exclusion criteria**<br>*Amended:* we excluded reviews of critically low quality, as assessed by AMSTAR-2 or the *ad hoc* qualitative tool.<br>*Amended:* we excluded reviews from review pairs with high overlap, as assessed by the Corrected Covered Area methodology.	We introduced two additional exclusion criteria related to the quality of reviews and overlap between reviews. Due to an abundance of reviews of, on average, low quality based on AMSTAR-2, we decided to exclude reviews of critically low quality, thus increasing the certainty of the evidence we subsequently synthesized. Overlap exclusion decisions were made in a data-driven approach, based on the assessment of primary study overlap. We excluded overlapping reviews to avoid double counting and overrepresentation of certain types of studies.
**Data sources and search strategy**<br>*Protocol:* we will use CoCites citation-based search tool to widen the net and retrieve articles that cite eligible systematic reviews. […] Additional searches will be developed for syntheses of effectiveness, implementation or process evaluations published or mandated by official health agencies. We will contact the first and last authors of selected reviews to retrieve grey literature that may otherwise have been missed.<br>*Amended:* we performed a manual search of reference lists of included reviews. The rest of the steps were not undertaken.	Our search strategies led to an overwhelming number of reviews on the topic. Due to an abundance and overrepresentation of available evidence, we decided to be pragmatic and not use any citation search tool. We did not contact experts of official health agencies since the volume of peer-reviewed evidence was already sufficient.
**Change in AMSTAR-2 assessment**<br>Item 10 (*Did the review authors report on the sources of funding for the studies included in the review?*) was not considered as critical.	The team made a consensus-based decision not to include item 10 of AMSTAR-2 as a critical item since this information was exclusively provided by Cochrane reviews. Considering this item as critical would have significantly limited the inclusion of reviews in our umbrella review. In addition, the team considered this criterion too stringent since this is not common practice in non-Cochrane, non-clinical reviews, especially in the field of public health intervention research.
**Change in *ad hoc* quality assessment tool for qualitative reviews**	The team decided to exclude critically low-quality reviews from the qualitative batch as well. Therefore, based on internal consensus, items 3,4,7,10 of the *ad hoc* quality assessment tool were designated as critical, and reviews with more than two critical flaws were excluded.
**Qualitative synthesis methods**<br>*Protocol:* Evidence from syntheses of qualitative research will also be presented in tabular format. Results will be synthesized and illustrated by using verbatim replications from the source review where appropriate. Findings will also be described and interpreted in the research team's own words. Caregivers' experiences and views on barriers to and facilitators of interventions will be identified in terms of acceptability, feasibility and added value (research question 4). The diversity of caregivers, care receivers and/or care contexts will be considered as far as possible.<br>*Amended:* For qualitative reviews, we followed the 'mega-aggregation framework' synthesis method, which has been described elsewhere. First, the extracted verbatim texts from reviews were coded line-by-line (complete coding) by two team members. Codes were then iteratively and deductively categorized based on the adapted version of van Houtven et al.'s framework. Throughout the review, we refer to verbatim texts extracted from reviews and their corresponding codes as third-order constructs, while primary studies inform second-order constructs, and the communication from participants (i.e., caregivers) is considered as first-order construct. Thus, the themes, categories, and sub-categories that we generated based on third-order constructs (i.e., reviews) are termed as fourth-order constructs. In case our fourth-order codes did not fit fully into the framework, they were inductively categorized into new themes. Codes referring to care receivers or staff opinions were removed. A third investigator checked the outcome of the categorization independently, and several consensus meetings were held to resolve disagreements and finalize the synthesis of qualitative materials.	In addition to amending research question 4, methods for synthesizing qualitative data were further developed. Instead of simply providing a narrative summary with verbatim quotes, we undertook a mega-aggregation approach, complemented by "best-fit" framework synthesis. We believe that these methods are better suited for synthesizing review-level qualitative data.

For peer review only - http://bmjopen.bmj.com/site/about/guidelines.xhtml

Supplementary Table 2. Search strategies

Databases:
1. Medline (via Ovid)
2. Web of Science Core Collection (via Clarivate)
3. PsycInfo (via Ovid)
4. Cinahl (via EBSCO)

Total number of hits: before deduplication n=11,605 / after deduplication n=6213

1. Search strategy and results in Medline

Interface: Ovid MEDLINE(R) and Epub Ahead of Print, In-Process & Other Non-Indexed Citations and Daily

Date of Search: 26 March 2021

Number of hits: 3,158

Field labels

- exp/ = exploded MeSH term
- / = non exploded MeSH term
- .ti,ab,kf. = title, abstract and author keywords
- adjx = within x words, regardless of order
- * = truncation of word for alternate endings

Database(s): **Ovid MEDLINE(R) and Epub Ahead of Print, In-Process, In-Data-Review & Other Non-Indexed Citations and Daily** 1946 to March 26, 2021

#	Searches	Results
1	exp Aged/	3219373
2	exp Aging/	250613
3	Frailty/	3780
4	(advanced age or aged or ageing or aging or elder* or frail* or geriatr* or gerontolog* or late* life or old age or old* adult* or old* client* or old* individual* or old* man or old* men or old* patient* or old* people or old* person* or old* population* or old* woman or old* women or oldest old or retired or senior*).ti,ab,kf.	1504150
5	Dementia/	53069
6	Alzheimer Disease/	97714
7	(dementia* or alzheimer*).ti,ab,kf.	230766
8	exp Neoplasms/	3433491
9	(neoplasm* or cancer*).ti,ab,kf.	2107334
10	exp Stroke/	141822
11	stroke.ti,ab,kf.	258717
12	Parkinson disease/	68962
13	parkinson*.ti,ab,kf.	124358
14	Multimorbidity/	1267
15	multimorbid*.ti,ab,kf.	5841
16	exp Heart failure/	126175
17	(heart failure or cardiac failure).ti,ab,kf.	193402
18	or/1-17	7707943
19	Caregivers/	38935
20	(caregiv* or care giv* or caretak* or care tak* or carer*).ti,ab,kf.	98680
21	((family or informal or unpaid) adj3 (care or caring)).ti,ab,kf.	17170
22	or/19-21	118701
23	(meta analysis or systematic review).pt.	214329
24	review.ti.	524072
25	systematic* review*.ab,kf.	154180
26	(meta analy* or metaanaly* or meta stud* or meta interpretation* or meta ethnograph* or meta summar* or meta synthes* or meta narrative* or mixed research synthes*).ti,ab,kf.	200564
27	((concept analy* or grounded theory) and review*).ti,ab,kf.	1518
28	or/23-27	699974
29	18 and 22 and 28	3304
30	(english or swedish or spanish or french or italian or german).lg.	29905965
31	29 and 30	3269
32	limit 31 to yr="2000 -Current"	3178
33	limit 32 to (comment or congress or editorial or letter)	20
34	32 not 33	3158

For peer review only - http://bmjopen.bmj.com/site/about/guidelines.xhtml

2. Search strategy and results in Web of Science Core Collection

Interface: Clarivate Analytics

Date of Search: 26 March 2021

Number of hits: 3,991

Field labels

- TS/Topic = title, abstract, author keywords and Keywords Plus
- NEAR/x = within x words, regardless of order
- * = truncation of word for alternate endings

# 15	3,991	**#11 AND #6 AND #3**<br>**Refined by: [excluding] DOCUMENT TYPES: (MEETING ABSTRACT OR BOOK REVIEW OR PROCEEDINGS PAPER OR EDITORIAL MATERIAL OR LETTER) AND PUBLICATION YEARS: (2021 OR 2013 OR 2005 OR 2020 OR 2012 OR 2004 OR 2019 OR 2011 OR 2003 OR 2018 OR 2010 OR 2002 OR 2017 OR 2009 OR 2001 OR 2016 OR 2008 OR 2000 OR 2015 OR 2007 OR 2014 OR 2006) AND LANGUAGES: (ENGLISH OR GERMAN OR SPANISH OR ITALIAN OR FRENCH OR SWEDISH)**<br>*Indexes=SCI-EXPANDED, SSCI, A&HCI, CPCI-S, CPCI-SSH, ESCI Timespan=All years*
# 14	4,007	**#11 AND #6 AND #3**<br>**Refined by: [excluding] DOCUMENT TYPES: (MEETING ABSTRACT OR BOOK REVIEW OR PROCEEDINGS PAPER OR EDITORIAL MATERIAL OR LETTER) AND PUBLICATION YEARS: (2021 OR 2013 OR 2005 OR 2020 OR 2012 OR 2004 OR 2019 OR 2011 OR 2003 OR 2018 OR 2010 OR 2002 OR 2017 OR 2009 OR 2001 OR 2016 OR 2008 OR 2000 OR 2015 OR 2007 OR 2014 OR 2006)**<br>*Indexes=SCI-EXPANDED, SSCI, A&HCI, CPCI-S, CPCI-SSH, ESCI Timespan=All years*
# 13	4,075	**#11 AND #6 AND #3**<br>**Refined by: [excluding] DOCUMENT TYPES: (MEETING ABSTRACT OR BOOK REVIEW OR PROCEEDINGS PAPER OR EDITORIAL MATERIAL OR LETTER)**<br>*Indexes=SCI-EXPANDED, SSCI, A&HCI, CPCI-S, CPCI-SSH, ESCI Timespan=All years*
# 12	4,262	**#11 AND #6 AND #3**<br>*Indexes=SCI-EXPANDED, SSCI, A&HCI, CPCI-S, CPCI-SSH, ESCI Timespan=All years*
# 11	1,062,768	**#10 OR #9 OR #8 OR #7**<br>*Indexes=SCI-EXPANDED, SSCI, A&HCI, CPCI-S, CPCI-SSH, ESCI Timespan=All years*
# 10	2,084	**TS=(("concept analy*" or "grounded theory") and review*)**<br>*Indexes=SCI-EXPANDED, SSCI, A&HCI, CPCI-S, CPCI-SSH, ESCI Timespan=All years*
# 9	393,878	**TS=("meta analy*" or metaanaly* or "meta stud*" or "meta interpretation*" or "meta ethnograph*" or "meta summar*" or "meta synthes*" or "meta narrative*" or "mixed research synthes*")**<br>*Indexes=SCI-EXPANDED, SSCI, A&HCI, CPCI-S, CPCI-SSH, ESCI Timespan=All years*
# 8	227,798	**TS="systematic* review*"**<br>*Indexes=SCI-EXPANDED, SSCI, A&HCI, CPCI-S, CPCI-SSH, ESCI Timespan=All years*
# 7	732,777	**TI=review**<br>*Indexes=SCI-EXPANDED, SSCI, A&HCI, CPCI-S, CPCI-SSH, ESCI Timespan=All years*
# 6	131,791	**#5 OR #4**<br>*Indexes=SCI-EXPANDED, SSCI, A&HCI, CPCI-S, CPCI-SSH, ESCI Timespan=All years*
# 5	23,282	**TS=(("family" or "informal" or "unpaid") NEAR/3 ("care" or "caring"))**<br>*Indexes=SCI-EXPANDED, SSCI, A&HCI, CPCI-S, CPCI-SSH, ESCI Timespan=All years*
# 4	115,945	**TS=(caregiv* or "care giv*" or caretak* or "care tak*" or carer*)**<br>*Indexes=SCI-EXPANDED, SSCI, A&HCI, CPCI-S, CPCI-SSH, ESCI Timespan=All years*
# 3	5,155,246	**#2 OR #1**<br>*Indexes=SCI-EXPANDED, SSCI, A&HCI, CPCI-S, CPCI-SSH, ESCI Timespan=All years*
# 2	3,790,895	**TS=(alzheimer* or cancer* or "cardiac failure" or dementia* or "heart failure" or multimorbid* or n eoplasm* or parkinson* or "stroke")**<br>*Indexes=SCI-EXPANDED, SSCI, A&HCI, CPCI-S, CPCI-SSH, ESCI Timespan=All years*
# 1	1,658,624	**TS=("advanced age" or "aged" or "ageing" or "aging" or elder* or frail* or geriatr* or gerontolog* o r "late* life" or "old age" or "old* adult*" or "old* client*" or "old* individual*" or "old* man" or " old* men" or "old* patient*" or "old* people" or "old* person*" or "old* population*" or "old* wo man" or "old* women" or "oldest old" or "retired" or senior*)**<br>*Indexes=SCI-EXPANDED, SSCI, A&HCI, CPCI-S, CPCI-SSH, ESCI Timespan=All years*

For peer review only - http://bmjopen.bmj.com/site/about/guidelines.xhtml

3. Search strategy and results in PsycInfo

Interface: Ovid

Date of Search: 26 March 2021

Number of hits: 1,933

Field labels

- exp/ = exploded controlled term
- / = non exploded controlled term
- .ti,ab,id. = title, abstract and author keywords
- adjx = within x words, regardless of order
- * = truncation of word for alternate endings

Database(s): **APA PsycInfo** 1806 to March Week 3 2021

#	**Searches**	**Results**
1	Older Adulthood/	6645
2	Geriatric patients/	13619
3	exp Aging/	77653
4	(advanced age or aged or ageing or aging or elder* or frail* or geriatr* or gerontolog* or late* life or old age or old* adult* or old* client* or old* individual* or old* man or old* men or old* patient* or old* people or old* person* or old* population* or old* woman or old* women or oldest old or retired or senior*).ti,ab,id.	470458
5	Dementia/ or exp Presenile dementia/	76186
6	(dementia* or alzheimer*).ti,ab,id.	104258
7	exp Neoplasms/	54065
8	(neoplasm* or cancer*).ti,ab,id.	65816
9	Cerebrovascular accidents/	21623
10	stroke.ti,ab,id.	34513
11	exp Parkinson's disease/	25813
12	parkinson*.ti,ab,id.	35346
13	Comorbidity/	34018
14	multimorbid*.ti,ab,id.	1051
15	(heart failure or cardiac failure).ti,ab,id.	4153
16	or/1-15	686012
17	Caregivers/	30033
18	Elder care/	4896
19	(caregiv* or care giv* or caretak* or care tak* or carer*).ti,ab,id.	73654
20	((family or informal or unpaid) adj3 (care or caring)).ti,ab,id.	10822
21	17 or 18 or 19 or 20	85126
22	review.ti.	158155
23	systematic* review*.ab,id.	28501
24	(meta analy* or metaanaly* or meta stud* or meta interpretation* or meta ethnograph* or meta summar* or meta synthes* or meta narrative* or mixed research synthes*).ti,ab,id.	41992
25	((concept analy* or grounded theory) and review*).ab,id.	1439
26	22 or 23 or 24 or 25	196607
27	16 and 21 and 26	2148
28	(english or swedish or spanish or french or italian or german).lg.	4774945
29	27 and 28	2064
30	limit 29 to yr="2000 -Current"	1950
31	(editorial or letter).dt.	67669
32	30 not 31	1933

For peer review only

For peer review only - http://bmjopen.bmj.com/site/about/guidelines.xhtml

4. Search strategy and results in Cinahl

Interface: Ebsco

Date of Search: 26 March 2021

Number of hits: 2,523

Field labels

- MH+ = exploded Cinahl Heading
- MH = non exploded Cinahl Heading
- TI = title
- AB = abstract
- Nx = within x words, regardless of order
- * = truncation of word for alternate endings

#	Query	Results
S31	S29 NOT S30	2,523
S30	PT Commentary or Editorial or Letter or Proceedings	854,425
S29	S17 AND S21 AND S28<br><br>Limiters - Published Date: 20000101-20211231;<br>Language: English, French, German, Italian, Swedish	2,590
S28	S22 OR S23 OR S24 OR S25 OR S26 OR S27	294,864
S27	AB (("concept analy*" or "grounded theory") and review*)	1,376
S26	TI ("meta analy*" or metaanaly* or "meta stud*" or "meta interpretation*" or "meta ethnograph*" or "meta summar*" or "meta synthes*" or "meta narrative*" or "mixed research synthes*") OR AB ("meta analy*" or metaanaly* or "meta stud*" or "meta interpretation*" or "meta ethnograph*" or "meta summar*" or "meta synthes*" or "meta narrative*" or "mixed research synthes*")	80,611
S25	AB "systematic* review*"	67,592
S24	TI review	206,732
S23	PT (meta analysis or meta synthesis)	39,376
S22	PT systematic review	110,054
S21	S18 OR S19 OR S20	100,197
S20	TI (((family or informal or unpaid) N3 (care or caring))) OR AB (((family or informal or unpaid) N3 (care or caring)))	21,928
S19	TI (caregiv* or "care giv*" or caretak* or "care tak*" or carer*) OR AB (caregiv* or "care giv*" or caretak* or "care tak*" or carer*)	74,717
S18	(MH "Caregivers")	36,859
S17	S1 OR S2 OR S3 OR S4 OR S5 OR S6 OR S7 OR S8 OR S9 OR S10 OR S11 OR S12 OR S13 OR S14 OR S15 OR S16	1,828,415
S16	TI ("heart failure" or "cardiac failure") OR AB ("heart failure" or "cardiac failure")	55,501
S15	(MH "Heart Failure+")	43,530
S14	TI multimorbid* OR AB multimorbid*	2,720
S13	(MH "Comorbidity")	64,158
S12	TI parkinson* OR AB parkinson*	27,885
S11	(MH "Parkinson Disease")	22,600
S10	TI stroke OR AB stroke	96,983
S9	(MH "Stroke+")	72,157
S8	TI (neoplasm* or cancer*) OR AB (neoplasm* or cancer*)	424,414
S7	(MH "Neoplasms+")	570,301
S6	TI (dementia* or alzheimer*) OR AB (dementia* or alzheimer*)	77,937
S5	(MH "Dementia") OR (MH "Dementia, Presenile+") OR (MH "Dementia, Senile+")	73,337
S4	TI (("advanced age" or aged or ageing or aging or elder* or frail* or geriatr* or gerontolog* or "late* life" or "old age" or "old* adult*" or "old* client*" or "old* individual*" or "old* man" or "old* men" or "old* patient*" or "old* people" or "old* person*" or "old* population*" or "old* woman" or "old* women" or "oldest old" or retired or senior*)) OR AB (("advanced age" or aged or ageing or aging or elder* or frail* or geriatr* or gerontolog* or "late* life" or "old age" or "old* adult*" or "old* client*" or "old* individual*" or "old* man" or "old* men" or "old* patient*" or "old* people" or "old* person*" or "old* population*" or "old* woman" or "old* women" or "oldest old" or retired or senior*))	452,573
S3	(MH "Frailty Syndrome")	2,612
S2	(MH "Aging+")	56,337
S1	(MH "Aged+")	860,457

For peer review only - http://bmjopen.bmj.com/site/about/guidelines.xhtml

Supplementary Table 3. Exclusion decisions based on high overlap among quantitative reviews*

Overlapping clusters (% of overlap)**	Excluded reviews	Rationale for the exclusion of overlaping reviews
Case management: excluded 2 out of 12 reviews		
Cheng 2020 vs Weinbrecht 2016: 24.8%<br>Lee 2020 vs Weinbrecht 2016: 20.5%	Weinbrecht 2016	Weinbrecht did not discuss findings on case management; we identified the category from the summary of included studies table. Lee and Cheng both discussed case management findings Therefore, we decided to exclude Weinbrecht from this category.
Corvol 2017 vs Reilly 2015: 31.8%<br>Reilly 2015 vs Goeman 2016: 15.4%	Corvol 2017	Corvol and Reilly both focused on case management. Reilly was a Cochrane review and better structured for the purposes of this umbrella review. Therefore, we decided to exclude Corvol from this category. Goeman focused on support worker role, which made the review sufficiently different form others.
Psychosocial interventions: excluded 13 out of 35 reviews		
Fu 2017 vs Gabriel 2020: 22.2%	Fu 2017	Fu and Gabriel both focused on caregivers of people with cancer. The quality of the reviews was similar. We decided to include Gabriel since it is more recent.
Hopwood 2018 vs Sin 2018: 22.1%<br>Hopwood 2018 vs Boots 2014: 38.9%<br>Hopwood 2018 vs Egan 2018: 20%<br>Boots 2014 vs Egan 2018: 20%<br>Egan 2018 vs Zhao 2019: 55.6%<br>Hopwood 2018 vs Zhao 2019: 13.5%<br>Hopwood 2018 vs Leng 2020: 24.3%	Boots 2014<br>Egan 2018<br>Zhao 2019	Hopwood, Sin, Boots, Egan, Zhao and Leng all concerned internet-based interventions. All but Sin focused on dementia caregivers, so Sin was sufficiently different. Leng was recent (2020) and included meta-analysis. Egan and Zhao shared 55.6% of studies and were very similar, the only difference being the publication date. Between Hopwood and Boots, Hopwood was more recent. Between Hopwood and Zhao, Hopwood was more informative since it included 36 studies (Zhao includes 6 studies and 5 of them are also included by Hopwood). From this cluster, we decided to include Hopwood, Sin and Leng.
Egan 2018 vs Gonzalez-Fraile 2021: 22.7%<br>Gonzalez-Fraile 2021 vs Lins 2014: 21.1%<br>Gonzalez-Fraile 2021 vs Zhao 2019: 23.8%<br>Gonzalez-Fraile 2021 vs Leng 2020: 34.5%<br>Gonzalez-Fraile 2021 vs Lucero 2019: 15.8%	Leng 2020<br>Lucero 2019	Gonzalez-Fraile was the most recent (2021) and a Cochrane review. We decided to exclude Egan (already excluded above), Leng, Zhao (already excluded above) and Lucero (similar topic, no meta-analysis). We included Lins because of narrower focus (concerns telephone-based interventions specifically).
Lee 2020 vs Amador-Marin 2017: 25%<br>Lee 2020 vs Weinbrecht 2016: 20.5%<br>Lee 2020 vs Teahan 2020: 17.1%	Amador-Marin 2017<br>Weinbrecht 2016	Lee and Tehan were most recent, each had more than 20 RCTs, and overlap between them was low. Thus, we decided to exclude Amador-Marin and Weinbrecht and instead include Teahan and Lee.
Cheng 2020 vs Weinbrecht 2016: 24.8%<br>Cheng 2020 vs Wiegelmann 2021: 23.8%<br>Cheng 2020 vs Vandepitte 2016: 16.7%<br>Cheng 2020 vs Lins 2014: 7.3%<br>Cheng 2020 vs Zabihi 2020: 5%<br>Cheng 2020 vs Akarsu 2019: 5.1%<br>Cheng 2020 vs Hopkinson 2019: 6.8%<br>Cheng 2020 vs Jensen 2015: 7.1%<br>Cheng 2020 vs Reilly 2015: 6.7%<br>Cheng 2020 vs Teahan 2020: 14.8%	Cheng 2020<br>Vandepitte 2016<br>Weinbrecht 2016	Cheng and Wiegelmann were the largest and most recent reviews. However, the overlap between Cheng and most of the smaller reviews was substantial (9 smaller reviews overlapped moderately or highly with Cheng). Therefore, we decided to keep Wiegelmann instead of Cheng, and included the rest of smaller overlapping reviews. In addition, we decided to exclude Vandepitte from this cluster. Weinbrecht was already excluded in the previous cluster.

For peer review only - http://bmjopen.bmj.com/site/about/guidelines.xhtml
For peer review only

Cheng 2020 vs GonzalezFraile 2021: 10.7%<br>Cheng 2020 vs Amador-Marin 2017: 7.8%<br>Cheng 2020 vs Lucero 2019: 5.1%<br>Cheng 2020 vs Piersol 2017: 11.5%		
Egan 2018 vs Guay 2017: 17.6%<br>Egan 2018 vs Wang 2020: 25%<br>Egan 2018 vs Leng 2019: 50%<br>Zhao 2019 vs Leng 2019: 50%<br>Leng 2019 vs Guay 2017: 23.5%<br>Zhao 2019 vs Guay 2017: 28.6%<br>Zhao 2019 vs Wang 2020: 30%<br>Guay 2017 vs Wang 2020: 18.8%<br>Guay 2017 vs Sin 2018: 13%	Guay 2017	Zhao, Egan and Leng were already excluded in previous clusters. Between Guay and Sin, we decided to keep Sin, since it had 78 studies, while Guay included only 12. We included Wang from this cluster.
Lins 2014 vs Lucero 2019: 15.8%	Lucero 2019	We already excluded Lucero in previous clusters. Lins was included.
Hopkinson 2019 vs Vernooij-Dassen 2011: 26.3%	Vernooij-Dassen 2011	Between Hopkinson and Vernooij-Dassen, both had meta-analysis and both were about Cognitive Behavioral Therapy exclusively. We decided to include Hopkinson, since it was more recent (2019 vs 2011) and had more studies.
Jensen 2015 vs Wiegelmann 2021: 25%<br>Teahan 2020 vs Wiegelmann 2021: 24.5%<br>Weinbrecht 2016 vs Wiegelmann 2021: 18.4%<br>Jensen 2015 vs Teahan 2020: 14.3%	Jensen 2015<br>Weinbrecht 2016	We already excluded Weinbrecht in previous clusters. Between Jensen and Teahan, Teahan was more recent. Thus, we additionally excluded Jensen from this cluster.
Legg 2011 vs Minshall 2019: 19%	Legg 2011	Between Legg and Minshall, we decided to include Minshall since it was more recent (2019 vs 2011) and included more studies (31 vs 8).
Education and Skills building: excluded 12 out of 32 reviews		
Legg 2011 vs Smith 2019: 22.2%<br>Pucciarelli 2020 vs Smith 2019: 22.2%<br>Legg 2011 vs Minshall 2019: 19%	Legg 2011	Smith had a wider focus, while Legg and Pucciarelli focused on caregivers of patients with stroke. Since the latter two did not have high enough overlap, we decided to include all three reviews. Between Legg and Minshall, we decided to include Minshall only since it was more recent (2019 vs 2011) and included more studies (31 vs 8).
Fu 2017 vs Gabriel 2020: 22.2%	Fu 2017	Fu and Gabriel both focused on caregivers of people with cancer. The quality of the reviews was similar. We decided to include Gabriel since it was more recent.
Hopwood 2018 vs Sin 2018: 22.1%<br>Hopwood 2018 vs Boots 2014: 38.9%<br>Hopwood 2018 vs Egan 2018: 20%<br>Boots 2014 vs Egan 2018: 20%<br>Egan 2018 vs Zhao 2019: 55.6%<br>Hopwood 2018 vs Zhao 2019: 13.5%<br>Hopwood 2018 vs Leng 2020: 24.3%	Boots 2014<br>Egan 2018<br>Zhao 2019	Hopwood, Sin, Boots, Egan, Zhao and Leng all concerned internet-based interventions. All but Sin focused on dementia caregivers, so Sin was sufficiently different. Leng was recent (2020) and included meta-analysis. Egan and Zhao shared 55.6% of studies and were very similar, the only difference being the publication date. Between Hopwood and Boots, Hopwood was more recent. Between Hopwood and Zhao, Hopwood was more informative since it included 36 studies (Zhao includes 6 studies and 5 of them were also included by Hopwood). From this cluster, we decided to include Hopwood, Sin and Leng.
Egan 2018 vs Gonzalez-Fraile 2021: 22.7%<br>Gonzalez-Fraile 2021 vs Lins 2014: 21.1%<br>Gonzalez-Fraile 2021 vs Zhao 2019: 23.8%	Leng 2020<br>Lucero 2019	Gonzalez-Fraile was the most recent (2021) and a Cochrane review. We decided to exclude Egan (already excluded above), Leng, Zhao (already excluded above) and Lucero (similar topic, no meta-

For peer review only - http://bmjopen.bmj.com/site/about/guidelines.xhtml

Gonzalez-Fraile 2021 vs Leng 2020: 34.5%<br>Gonzalez-Fraile 2021 vs Lucero 2019: 15.8%		analysis). We included Lins because of a narrower focus (concerns telephone-based interventions specifically).
Lee 2020 vs Amador-Marin 2017: 25%<br>Lee 2020 vs Weinbrecht 2016: 20.5%<br>Lee 2020 vs Teahan 2020: 17.1%	Amador-Marin 2017<br>Weinbrecht 2016	Lee and Tehan were most recent, each had more than 20 RCTs, and overlap between them was low. Thus, we decided to exclude Amador-Marin and Weinbrecht and instead include Teahan and Lee.
Cheng 2020 vs Weinbrecht 2016: 24.8%<br>Cheng 2020 vs Wiegelmann 2021: 23.8%<br>Cheng 2020 vs Vandepitte 2016: 16.7%<br>Cheng 2020 vs Lins 2014: 7.3%<br>Cheng 2020 vs Zabihi 2020: 5%<br>Cheng 2020 vs Akarsu 2019: 5.1%<br>Cheng 2020 vs Hopkinson 2019: 6.8%<br>Cheng 2020 vs Jensen 2015: 7.1%<br>Cheng 2020 vs Reilly 2015: 6.7%<br>Cheng 2020 vs Teahan 2020: 14.8%<br>Cheng 2020 vs GonzalezFraile 2021: 10.7%<br>Cheng 2020 vs Amador-Marin 2017: 7.8%<br>Cheng 2020 vs Lucero 2019: 5.1%<br>Cheng 2020 vs Piersol 2017: 11.5%	Cheng 2020<br>Vandepitte 2016	Cheng and Wiegelmann were the largest and most recent reviews. However, the overlap between Cheng and most of the smaller reviews was substantial (9 smaller reviews overlapped moderately or highly with Cheng). Therefore, we decided to keep Wiegelmann instead of Cheng, and included the rest of smaller overlapping reviews. In addition, we decided to exclude Vandepitte from this cluster. Therefore, we decided to exclude Weinbrecht (already excluded above) and Vandepitte (already excluded above) from this cluster.
Egan 2018 vs Guay 2017: 17.6%<br>Egan 2018 vs Leng 2019: 50%<br>Zhao 2019 vs Leng 2019: 50%<br>Leng 2019 vs Guay 2017: 23.5%<br>Zhao 2019 vs Guay 2017: 28.6%<br>Guay 2017 vs Sin 2018: 13%	Guay 2017	Zhao, Egan and Leng were already excluded in previous clusters. Between Guay and Sin, we decided to keep Sin, since it had 78 studies, while Guay included only 12.
Lins 2014 vs Lucero 2019: 15.8%		We already excluded Lucero in previous clusters. Lins was included.
Jensen 2015 vs Wiegelmann 2021: 25%<br>Teahan 2020 vs Wiegelmann 2021: 24.5%<br>Weinbrecht 2016 vs Wiegelmann 2021: 18.4%<br>Jensen 2015 vs Teahan 2020: 14.3%	Jensen 2015	We already excluded Weinbrecht in previous clusters. Between Jensen and Teahan, Teahan was more recent. Thus, we additionally excluded Jensen from this cluster.
Respite care: excluded 1 out of 6 reviews		
Shaw 2009 vs Mason 2007: 21.7%	Mason 2007	Shaw was more recent and included more studies (104 vs 22). Thus, we decided to exclude Mason from this cluster.
Relaxation, physical activity, and leisure: excluded 1 out of 7 reviews		
Cheng 2020 vs Wiegelmann 2021: 23.8%	Wiegelmann 2021	Cheng and Wiegelmann were the largest and most recent reviews. Cheng included more studies on the topic, so we decided to exclude Wiegelmann from this cluster.
Mindfulness: excluded 1 out of 6 reviews		
Liu 2018 vs Collins 2019: 30.8%<br>Liu 2018 vs Shim 2020: 18.2%	Collins 2019	All three reviews concerned mindfulness interventions for caregivers of people with dementia. Liu was a Cochrane review (but only included 5 studies), Shim included 20 studies. We decided to exclude Collins from this cluster.

*The number of qualitative reviews in each category was low and overlapping clusters were limited. Due to the scarcity of qualitative evidence and low overlap, we decided not to exclude any qualitative review based on this approach. For full information about overlap among qualitative reviews, see the Citation Matrix in Supplementary File 2.

**We assessed clusters of reviews with very high (33 pairs), high (40 pairs) or moderate overlap (only if one is almost fully included in the other); see the Citation Matrix in Supplementary File 1 for the overlap among all included reviews after quality assessment.

For peer review only

Supplementary Table 4. Application of Hendricks et al.'s mega-aggregation framework synthesis method[1]

**Step 1: Identify a clearly defined review question and objectives**		
This umbrella review aims to explore the effectiveness of interventions targeting informal caregivers' physical and mental health. The fourth objective, which is the one mainly feeding on qualitative evidence, is as follows: how are the proposed interventions experienced by caregivers in terms of effectiveness and implementation outcomes?		
**Step 2: Identify a theoretical framework or model**		
Adapted version of Van Houtven et al.'s framework[2,3]	Intervention, Context of caregiving relationship, Caregiver, Care receiver, Caregiver knowledge, attitude and behaviors, Caregivers' experience of the intervention, Caregiver outcomes	The adapted framework describes the different determinants that affect the effectives of support interventions in addressing health outcomes of caregivers, and how they interact by modulating the other determinants and components
**Step 3: Decide on criteria for considering reviews for inclusion**		
Inclusion and exclusion criteria used to select reviews are presented in Table 1 of the published umbrella review protocol[3]		
**Step 4: Conduct searching and screening**		
The following databases were searched from 1 January 2000 to 26 March 2021: Medline, CINAHL, PsycINFO and Web of Science. In addition, we performed a manual search of the reference lists of included reviews	Screening was done independently and in duplicate by MK and ACL; MK and LM; MK and LS; MK and LD	
**Step 5: Conduct quality appraisal of the included studies**		
An *ad hoc* tool was used, developed and piloted by review authors	Quality appraisal was done independently and induplicate by all co-authors	Reviews of critically low quality were excluded (i.e., those that had 2 or more critical items missing or not reported); 18 reviews contributed to the next step
**Step 6: Data extraction and categorization**		
AA, ACL and MK read all reviews	AA and MK extracted the verbatims and each verified the extraction of the other co-author	Construction of fourth-order constructs (codes, categories, and aggregations) was done in consensus by AA and MK, and then reviewed by third co-author ACL. In the case of codes and categories not fitting the framework, they were inductively abstracted and new themes were generated and added to the framework. All three co-authors then reviewed and discussed the process until consensus was reached by all three co-authors. The results of the categorization and aggregation was then shared with the other co-authors and consensus was reached
**Step 7: Present and synthesize the findings**		
Two themes were constructed (intervention outcomes and implementation outcomes) using 200 codes, with further groupings of codes in categories and sub-categories	Evidence gaps and lines of actions were identified following convergence of the qualitative and quantitative findings, and were later presented in the discussion	
**Step 8: Transparent reporting**		
PRIOR[4] reporting guideline were used as far as possible. The protocol was published (doi:10.1136/bmjopen-2021-053117) and the categorization and aggregation of the third-order constructs to develop the fourth-order constructs was shown in supplementary files. Additionally, differences between the protocol and the manuscript were reported in supplementary files.		

References

1. Hendricks L, Eshun-Wilson I, Rohwer A. A mega-aggregation framework synthesis of the barriers and facilitators to linkage, adherence to ART and retention in care among people living with HIV. *Syst Rev*. 2021;10(1):1-28. doi:10.1186/s13643-021-01582-z
2. Van Houtven CH, Voils CI, Weinberger M. An organizing framework for informal caregiver interventions: Detailing caregiving activities and caregiver and care recipient outcomes to optimize evaluation efforts. *BMC Geriatr*. 2011;11. doi:10.1186/1471-2318-11-77
3. Calderón-Larrañaga A, Kirvalidze M, Dahlberg L, Sacco LB, Morin L. Effectiveness of interventions to address the negative health outcomes of informal caregiving to older adults: Protocol for an umbrella review. *BMJ Open*. 2021;11(11). doi:10.1136/bmjopen-2021-053117
4. Gates M, Gates A, Pieper D, et al. Reporting guideline for overviews of reviews of healthcare interventions: The Preferred Reporting Items for Overviews of Reviews (PRIOR) statement. *MetaArXiv*. Published online 2022. doi:10.31222/osf.io/82wau

For peer review only - http://bmjopen.bmj.com/site/about/guidelines.xhtml

Supplementary Table 5a. AMSTAR-2 quality assessment tool for quantitative reviews

High	Moderate	Low	Critically low (excluded)	
Maayan, 2014	Cheng, 2020	Amador-Marin, 2017	AlDaken, 2018	Van'tLeven, 2013
Forster, 2012	Minshall, 2019	Egan, 2018	Bergstrom, 2018	Zhu, 2021
Liu, 2018	Smith, 2019	Gabriel, 2020	Brodaty, 2003	Wang, 2017
Reilly, 2015	Gonzalez-Fraile, 2021	Heckel, 2019	Bustillo, 2018	Peacock, 2003
Vernooij-Dassen, 2011	Wang, 2020	Hopwood, 2018	Cheng, 2014	Northouse, 2010
Goeman, 2016	Akarsu, 2019	Irons, 2020	Dam, 2016	Dharmawardene, 2016
	Corry, 2019	Leng, 2020	EluvathingolJose, 2013	Brereton, 2007
	Legg, 2011	Shaw, 2009	Grandi, 2019	Liu, 2017
	Lins, 2014	Shim, 2020	Hopkinson, 2012	Thompson, 2007
	Pucciarelli, 2020	Teahan, 2020	Irani, 2020	Eldred, 2008
	Sin, 2018	Zhao, 2019	Kedia, 2020	Regan, 2012
	Lucero, 2019	Collins, 2019	Kor, 2018	Heynsbergh. 2018
	Treanor, 2019	Guay, 2017	Lee, 2007	Abrahams, 2018
		Lee, 2020	Li, 2020	Li, 2016
		Mason, 2007	Li, 2014	Jackson, 2016
		Vandepitte, 2016	Luo, 2020	You, 2012
		Waldron, 2013	Ruggiano, 2018	Lambert, 2016
		Weinbrecht, 2016	Selwood, 2007	Domingues, 2018
		Zabihi, 2020	Sherifali, 2018	Miles, 2020
		Boots, 2014	Smith, 2014	Scott, 2016
		Corvol, 2017	Spencer, 2019	Hurley, 2014
		Cuthbert, 2017	ZabaleguiYarnoz, 2008	Etxeberria, 2020
		Doyle, 2020	Bourne, 2020	Deeken, 2019
		Fu, 2017	Bunn, 2016	Hempel, 2008
		Hopkinson, 2019	Byeon, 2020	Fange, 2020
		Jensen, 2015	Caress, 2009	Lally, 2016
		Maffioletti, 2019	Cassidy, 2021	Ussher, 2009
		Parkinson, 2019	Epps, 2021	Stahl, 2016
		Bennett, 2019	Frias, 2020	Mantovan, 2010
		Wiegelmann, 2021	Kaddour, 2018	Panzeri, 2019
		Greenwood, 2016	Kaltenbaugh, 2015	Poon, 2019
		Piersol, 2017	Kwon, 2017	Pritchard, 2020
			Laver, 2017	Xu, 2020
			Leung, 2017	Ahn, 2020
			Lorca-Cabrera, 2020	Chien, 2011
			Mason, 2007	Cooper, 2007
			McKechnie, 2014	Corbett, 2012
			Pinquart, 2006	Du Preez, 2018
			Pleasant, 2020	Elvish, 2013
			Pusey, 2001	Kleine, 2019
			Smits, 2007	Lee, 2020
			Vandepitte, 2016	Mason, 2008
			Verreault, 2021	MoleroJurado, 2020
			Waller, 2017	Moon, 2013
			Walter, 2020	Moore, 2020
			Wu, 2019	O'Toole, 2017

The definitions of each quality category, critical items and recommendations on grading are available from the original publication: Shea BJ, Reeves BC, Wells G, et al. AMSTAR 2: A critical appraisal tool for systematic reviews that include randomised or non-randomised studies of healthcare interventions, or both. BMJ. 2017;358:j4008. doi:10.1136/bmj.j4008.

For peer review only

For peer review only - http://bmjopen.bmj.com/site/about/guidelines.xhtml

Supplementary Table 5b. ***Ad hoc*** **quality assessment tool* for qualitative reviews**

Qualitative reviews with 2 or more critical flaws**	Qualitative reviews less than 2 critical flaw**
Jackson, 2016	Parkinson, 2019
Sousa, 2016	Sin, 2018
Hempel, 2008	Goeman, 2016
Armstrong, 2019	Li, 2016
Elvish, 2013	Miles, 2020
Moon, 2013	Boots, 2014
Caress, 2009	Du Preez, 2018
EluvathingolJose, 2013	Lins, 2014
Heckel, 2019	Maffioletti, 2019
	Pritchard, 2020
	Bourne, 2020
	Bunn, 2016
	AlDaken, 2018
	Dam, 2016
	Hopwood, 2018
	Irons, 2020
	Shaw, 2009
	Smith, 2014

*The tool can be accessed in the published protocol (doi:10.1136/bmjopen-2021-053117).
**Critical flaws included: transparency and comprehensiveness of the literature search (item #3), using at least two databases for the search (item #4), assessment of methodological quality of primary studies using explicit criteria (item #7), involvement of two or more researchers in data synthesis process (item #11).

For peer review only - http://bmjopen.bmj.com/site/about/guidelines.xhtml

Supplementary Table 6. Repository of all included reviews (*n*=47)

Author, year	Countries of primary studies	Study design	Databases searched	End date of literature search	# studies included in the umbrella review	# participants, mean age, % female	Intervention type	Disease of care receiver	Health outcomes of caregiver	Review type and synthesis method	Quantitative, qualitative, or mixed-methods
Akarsu, 2019[1]	US	RCTs	Embase, cinahl, medline, psycinfo, psycarticles, assia, central, sociological abstracts, spp	2015	13	2056 caregivers, mean age 59 (SD=11.36), 86% female	Psychosocial, Education and skills	Dementia	Depression	Systematic review with meta-analysis	Quantitative
Aldaken, 2018[2]	UK, China, Iran	RCTs, incl. some with qualitative interviews	Ebsco, google scholar, science direct	2017	1	NA	Mindfulness	Cancer	NA	Systematic review	Qualitative
Bennett, 2019[3]	US, Germany, Netherlands, UK, Hong Kong, Australia, Brazil	RCTs	Medline, embase, central, cumulative index to nursing and allied health literature, psycinfo, education resources information Centre, otseeker, pedro, clinical trial registries	Feb-2018	15	2063 dyads, mean age 65, 20%-90% of caregivers were spouses	Education and skills	Dementia	Emotional distress, HR-QoL	Systematic review with meta-analysis	Quantitative
Boots, 2014[4]	NA	Any	Pubmed, psycinfo, cinahl, web of science, cochrane library	Jan-2013	4	NA	Psychosocial, Education and skills	Dementia	NA	Systematic review	Qualitative
Bourne, 2020[5]	UK, US, Australia, New Zealand	Qualitative and mixed method	Psycinfo, medline, web of science, assia, google scholar	Mar-2020	10	NA	Relaxation and leisure	Dementia	NA	Systematic review	Qualitative
Bunn, 2016[6]	UK	Any	Medline, cinahl, scopus, nhs evidence, cochrane library, google scholar	Nov-2012	5	NA	Case management, Psychosocial, Education and skills	Dementia	NA	Systematic review	Qualitative

For peer review only - http://bmjopen.bmj.com/site/about/guidelines.xhtml

Author, year	Countries of primary studies	Study design	Databases searched	End date of literature search	# studies included in the umbrella review	# participants, mean age, % female	Intervention type	Disease of care receiver	Health outcomes of caregiver	Review type and synthesis method	Quantitative, qualitative, or mixed-methods
Cheng, 2020[7]	US, Australia, Brazil, Denmark, Norway, UK, Spain, Greece, Finland, India, Netherlands, Germany, Egypt, Japan, Iran, Canada, Taiwan	RCTs, quasi-experimental	Psycinfo, medline, cinahl	Mid-2018	37	NA	Relaxation and leisure, Mindfulness	Dementia	Depression, anxiety, HR-QoL, physical health	Systematic review with meta-analysis	Quantitative
Corry, 2019[8]	NA	RCTs, quasi-experimental	Central, medline, embase, psycinfo, proquest, cinahl	Nov-2018	21	1690 caregivers, age range 19-87, >70.5% female	Psychosocial, Education and skills	Any	HR-QoL, psychological health (depression, anxiety), physical health	Systematic review with meta-analysis	Quantitative
Cuthbert, 2017[9]	NA	RCTs, quasi-experimental	Medline, pubmed, cinahl	Sep-2015	14	12-137 caregivers, age range 41-73.7	Relaxation and leisure	Any	Depression, anxiety, well-being, QoL, physical strengthening, blood pressure, weight	Systematic review	Quantitative
Dam, 2016[10]	NA	Any	Pubmed, psycinfo, cinahl, web of science, cochrane library	May-2015	4	NA	Psychosocial	Dementia	NA	Systematic review	Qualitative
Doyle, 2020[11]	NA	RCTs, quasi-experimental, case-control, and cohort studies	Pubmed, web of science, cinahl plus, cochrane library, ot seeker, psycinfo, scopus	Apr-2017	11	862 caregivers, mean age 67, 69.4% female	Relaxation and leisure	Any	Depression, anxiety, physical health	Systematic review	Quantitative
Du Preez, 2018[12]	NA	Qualitative studies and mixed methods	Pubmed, web of science, medical database (proquest), medline, bmj best practice, scopus, psycinfo, otseeker, cinahl	Nov-2016	10	NA	Respite	Dementia	NA	Integrative review	Qualitative

For peer review only - http://bmjopen.bmj.com/site/about/guidelines.xhtml

Author, year	Countries of primary studies	Study design	Databases searched	End date of literature search	# studies included in the umbrella review	# participants, mean age, % female	Intervention type	Disease of care receiver	Health outcomes of caregiver	Review type and synthesis method	Quantitative, qualitative, or mixed-methods
Forster, 2012[13]	US, UK, Australia, Sweden, the Netherlands, Taiwan, Thailand	RCTs	Cochrane library, dare, eed, hta database, medline, embase, cinahl, psycinfo	Jun-2012	21	1290 caregivers	Case management, Psychosocial, Education and skills	Stroke	Depression, HR-QoL	Systematic review	Quantitative
Gabriel, 2020[14]	US, France	RCTs, quasi-experimental	Pubmed, medline, cinahl, psycinfo, web of science, who clinical trials registry, international standard rct registry	2019	12	3390 participants (including caregivers), age range for caregivers 43-61	Psychosocial, Education and skills	Cancer	Psychological/emotional and physical domains of QoL	Systematic review	Quantitative
Goeman, 2016[15]	Hong Kong, Netherlands, US, Italy, Finland, UK, Canada, Australia	Any	Medline, cinahl, psycinfo, google scholar	Dec-2014	36	NA	Case management	Dementia	Depression, HR-QoL, general health	Systematic review	Mixed methods
Gonzalez-Fraile, 2021[16]	US, China, Netherlands, France, Canada, Spain, UK	RCTs	Alois, specialised register of the cochrane dementia and cognitive improvement group	Apr-2020	26	2367 caregivers, median age 63, 72% female	Psychosocial, Education and skills	Dementia	Depression, depressive symptoms, HR-QoL	Systematic review with meta-analysis	Quantitative
Greenwood, 2016[17]	US, Spain	RCTs and quasi-experimental	Medline, embase, cochrane library, psycinfo; cinahl plus, applied social sciences index, abstracts and healthcare management information consortium	Jul-2015	4	447 caregivers, age range 61-72, mostly female	Case management, Psychosocial, Education and skills	Dementia, stroke	Depression, general health	Systematic review	Quantitative
Li, 2016[18]	US, the Netherlands, China, Canada	Any	Cochrane library, pubmed, medline, cinahl, psycinfo, web of science, cnki, wanfang data, vip	Feb-2015	6	NA	Mindfulness	Any	NA	Systematic review	Qualitative
Heckel, 2019[19]	Australia	RCTs and quasi-experimental	Medline, cinahl, psycinfo, embase	May-2018	2	NA	Psychosocial, Education and skills	Cancer	Depression, emotional distress	Systematic review	Quantitative

For peer review only - http://bmjopen.bmj.com/site/about/guidelines.xhtml

Author, year	Countries of primary studies	Study design	Databases searched	End date of literature search	# studies included in the umbrella review	# participants, mean age, % female	Intervention type	Disease of care receiver	Health outcomes of caregiver	Review type and synthesis method	Quantitative, qualitative, or mixed-methods
Hopkinson, 2019[20]	Spain, US, UK, Germany, Canada, Brazil, Italy	RCTs and quasi-experimental	Medline, embase, cinahl, psycinfo, cochrane library	Jan-2017	25	Age range 56.6-72.6	Psychosocial	Dementia	Depression, anxiety	Systematic review with meta-analysis	Quantitative
Hopwood, 2018[21]	US, the Netherlands, Spain, Poland, Denmark, Hong Kong, Canada, France, UK, Germany	Any	Cinahl, cochrane library, embase, medline, psycinfo, web of science	Apr-2018	31	NA	Case management, Psychosocial, Education and skills	Dementia	HR-QoL, depression, anxiety, self-rated health	Systematic review	Mixed methods
Irons, 2020[22]	Australia, UK, US, Israel	Any	Medline, pubmed, ebsco, cinahl, embase, psycinfo, cochrane library, scopus, web of science, google scholar	May-2019	17	NA	Relaxation and leisure	Neurological conditions (e.g., dementia, stroke, Parkinson's disease)	HR-QoL, anxiety, depression	Integrative systematic review, meta-ethnography	Mixed methods
Lee, 2020[23]	US, UK, Germany, Spain, Netherlands, Portugal, China, Russia, Peru, Brazil, Denmark, Greece	RCTs	Medline, cinahl, psycinfo, cochrane library	2017	26	3906 caregivers, mean age 60.5, 73.9% female	Case management, Psychosocial, Education and skills, Relaxation and leisure	Dementia	HR-QoL	Systematic review with meta-analysis	Quantitative

For peer review only - http://bmjopen.bmj.com/site/about/guidelines.xhtml
For peer review only

Author, year	Countries of primary studies	Study design	Databases searched	End date of literature search	# studies included in the umbrella review	# participants, mean age, % female	Intervention type	Disease of care receiver	Health outcomes of caregiver	Review type and synthesis method	Quantitative, qualitative, or mixed-methods
Lins, 2014[24]	US, Germany, Canada	Any	Cochrane dementia and cognitive improvement group's specialized register, cochrane library, medline, medline in process, embase, cinahl, psyndex, psycinfo, web of science, dimdi databases, springer database, science direct, trial registers, web of science, google scholar	Feb-2013	11	NA	Psychosocial, Education and skills	Dementia	Depression, anxiety	Systematic review with meta-analysis	Mixed methods
Liu, 2018[25]	US, China	RCTs and quasi-experimental	Alois, specialised register of the cochrane dementia and cognitive improvement group	Sep-2017	5	201 caregivers, age range 57.5-71.3, >80% female	Mindfulness	Dementia	Depressive symptoms, anxiety	Systematic review with meta-analysis	Quantitative
Lucero, 2019[26]	NA	RCTs	Pubmed, cinahl, web of science, psycinfo	Jul-2017	12	NA	Case management	Dementia	Depression, anxiety	Systematic review	Quantitative
Maayan, 2014[27]	US, Canada	RCTs	Alois, specialised register of the cochrane dementia and cognitive improvement group	Dec-2012	4	NA	Respite	Dementia	Depression	Systematic review	Quantitative
Maffioletti, 2019[28]	US, the Netherlands, Norway, Scotland, Iceland, HongKong, Australia, Italy, Sweden, Germany	Any	Pubmed, psycinfo, scopus, scielo	Aug-2018	21	NA	Respite	Dementia	Depression, psychological well-being, health status	Systematic review	Mixed methods
Miles, 2020[29]	NA	Any design with comparison group	Medline, embase, web of science, cinahl, pubmed, scopus, psycinfo, cochrane library	Sep-2018	2	NA	Psychosocial, Education and skills	Dementia	NA	Systematic review	Qualitative

For peer review only - http://bmjopen.bmj.com/site/about/guidelines.xhtml

Author, year	Countries of primary studies	Study design	Databases searched	End date of literature search	# studies included in the umbrella review	# participants, mean age, % female	Intervention type	Disease of care receiver	Health outcomes of caregiver	Review type and synthesis method	Quantitative, qualitative, or mixed-methods
Minshall, 2019[30]	NA	RCTs	Medline, cinahl, psycinfo, socindex, cochrane library, web of science, Scopus databases, grey literature	Sep-2018	21	2079 caregivers, age range 49-76	Psychosocial, Education and skills	Stroke	Depression, HR-QoL	Systematic review with meta-analysis	Quantitative
Parkinson, 2019[31]	NA	Any	Amed, central, cinahl, medline, nursing and allied health database, psycarticles, psycinfo	2017	9	215 caregivers, age range 48.5-65.6	Mindfulness	Any	Anxiety	Systematic review	Mixed methods
Piersol, 2017[32]	NA	Any	Medline, psycinfo, cinahl, otseeker, cochrane database of systematic reviews	Apr-2014	43	NA	Case management, Psychosocial, Education and skills, Relaxation and leisure	Dementia	HR-QoL, depression, anxiety, physical health	Systematic review	Quantitative
Pritchard, 2020[33]	US, Australia, Europe	Qualitative	Medline, cochrane database of systematic reviews, embase, emcare, cinahl, otseeker	May-2018	7	9-495 caregivers, age range 59.8 (SD=13.9) to 68.7 (SD=8.6)	Education and skills	Dementia	NA	Systematic review with meta-analysis, qualitative synthesis (thematic analysis, framework approach)	Qualitative
Pucciarelli, 2020[34]	UK, Spain, Netherlands, US, Taiwan, Australia	RCTs and quasi-experimental	Pubmed, cinahl, psycinfo	May-2019	16	2187 caregivers, mean age 58, 75% female	Case management, Psychosocial, Education and skills	Stroke	Depression, HR-QoL	Systematic review with meta-analysis	Quantitative
Reilly, 2015[35]	Hong Kong, Netherlands, US, India, Canada, UK	RCTs	Cochrane library, medline, embase, psycinfo, cinahl, lilacs, web of science, campbell collaboration/soro database	Mar-2014	11	NA	Case management	Dementia	HR-QoL, depression, general health	Systematic review with meta-analysis	Quantitative

For peer review only - http://bmjopen.bmj.com/site/about/guidelines.xhtml

Author, year	Countries of primary studies	Study design	Databases searched	End date of literature search	# studies included in the umbrella review	# participants, mean age, % female	Intervention type	Disease of care receiver	Health outcomes of caregiver	Review type and synthesis method	Quantitative, qualitative, or mixed-methods
Shaw, 2009[36]	UK, US, Canada, Australia, New Zealand, Germany, Japan	Any	Medline, amed, assia, ibss, bni, cochrane library, dare, hta database, nhs economic evaluation database, cinahl, crisp, econlit, embase, hmic, nrr, psycinfo, scopus, social care online, web of science	2008	174	NA	Respite	Frailty	Depression, anxiety	Systematic review with meta-analysis, meta-synthesis of qualitative studies	Mixed methods
Shim, 2020[37]	NA	RCTs	Pubmed, psycinfo, scopus, cinahl, embase	Feb-2020	9	14-120 caregivers	Mindfulness	Dementia	Cognitive tests, depression, mindfulness, anxiety, biomarkers for stress	Systematic review	Quantitative
Sin, 2018[38]	UK, Australia, US, Hong-Kong, Canada, Sweden, Norway, Netherlands, France, Spain, Denmark, Poland, Germany, Italy, Greece	Any	Medline, psycinfo, cinahl, embase, web of science, assia, central, hta database, dare, eed	Dec-2016	81	4537 caregivers	Psychosocial, Education and skills	Any	Depression, anxiety, distress, HR-QoL	Systematic review	Mixed methods
Smith, 2014[39]	US, UK, Canada	Any	Medline, embase, psycinfo, social policy and practice, cinahl plus, allied and complimentary medicine, social sciences citation index, scopus	Jan-2013	2	NA	Psychosocial, Education and skills	Dementia, stroke	NA	Systematic review	Qualitative

For peer review only - http://bmjopen.bmj.com/site/about/guidelines.xhtml

Author, year	Countries of primary studies	Study design	Databases searched	End date of literature search	# studies included in the umbrella review	# participants, mean age, % female	Intervention type	Disease of care receiver	Health outcomes of caregiver	Review type and synthesis method	Quantitative, qualitative, or mixed-methods
Smith, 2019[40]	US, UK, Australia, Sweden, Canada, Ireland, Germany, India, Spain, Taiwan, Thailand	RCTs and quasi-experimental	Embase, medline, cinahl, pubmed, world health organization international clinical trial registry platform registry, clinicaltrials.gov registry	Mar-2019	23	NA	Education and skills	Stroke, frailty	HR-QoL, depression, anxiety	Systematic review with meta-analysis	Quantitative
Teahan, 2020[41]	US, China, Spain, the Netherlands, Taiwan, UK, Peru, Russia, India	RCTs	Cochrane, pubmed, cinahl, psycinfo, embase, assia	Mar-2016	24	NA	Psychosocial, Education and skills	Dementia	Depression, HR-QoL, general health	Systematic review with meta-analysis	Quantitative
Treanor, 2019[42]	US, UK, Denmark, Canada, Netherlands, Australia	RCTs and quasi-experimental	Cochrane central, medline, embase, psycinfo, proquest, open single, web of science	Dec-2018	19	1967 caregivers	Psychosocial, Education and skills	Cancer	HR-QoL, depression, anxiety, emotional distress, physical health status	Systematic review with meta-analysis	Quantitative
Vandepitte, 2016[43]	Canada, US, Germany, Italy	RCTs and quasi-experimental	Pubmed, web of science	Mar-2015	5	NA	Respite	Dementia	Depression, anxiety	Systematic review	Quantitative
Waldron, 2013[44]	N.A.	RCTs	Medline, psycinfo, embase, cinahl, pubmed, cochrane library	Nov-2010	6	1115 caregivers, mean age 56.5 (SD=3.3), 81.9% female	Psychosocial	Cancer	HR-QoL	Systematic review	Quantitative
Wang, 2020[45]	US, the Netherlands, France	RCTs and quasi-experimental	Cinahl, scopus, embase, medline, psycinfo, web of science, world health organization international clinical trials registry platform	Jul-2019	8	NA	Psychosocial	Dementia	Depression, anxiety	Systematic review with meta-analysis	Quantitative
Wiegelmann, 2021[46]	US, UK, Germany, China	RCTs	Pubmed, psycinfo, scopus, cinahl	Aug-2018	37	NA	Psychosocial, Education and skills	Dementia	HR-QoL, depression	Systematic review	Quantitative

For peer review only - http://bmjopen.bmj.com/site/about/guidelines.xhtml

For peer review only

Author, year	Countries of primary studies	Study design	Databases searched	End date of literature search	# studies included in the umbrella review	# participants, mean age, % female	Intervention type	Disease of care receiver	Health outcomes of caregiver	Review type and synthesis method	Quantitative, qualitative, or mixed-methods
Zabihi, 2020[47]	Hong Kong, US, Spain, Australia	RCTs	Medline, embase, psycinfo, cinahl, central and online trial registers for randomised controlled clinical trials	Dec-2018	14	NA	Psychosocial	Dementia	Depression (symptoms and diagnosis)	Systematic review with meta-analysis	Quantitative

Abbreviations: RCT, Randomized Controlled Trial; QoL, quality of life; HR-QoL, Health-Related Quality of Life.

For peer review only

For peer review only - http://bmjopen.bmj.com/site/about/guidelines.xhtml

Full references of all included reviews (n=47)

1. Akarsu NE, Prince MJ, Lawrence VC, Das-Munshi J. Depression in carers of people with dementia from a minority ethnic background: Systematic review and meta-analysis of randomised controlled trials of psychosocial interventions. *Int J Geriatr Psychiatry*. 2019;34(6):790-806. doi:10.1002/gps.5070
2. Al Daken LI, Ahmad MM. The implementation of mindfulness-based interventions and educational interventions to support family caregivers of patients with cancer: A systematic review. *Perspect Psychiatr Care*. 2018;54(3 PG-441-452):441-452. doi:https://dx.doi.org/10.1111/ppc.12286
3. Bennett S, Laver K, Voigt-Radloff S, et al. Occupational therapy for people with dementia and their family carers provided at home: a systematic review and meta-analysis. *BMJ Open*. 2019;9(11 PG-e026308):e026308. doi:https://dx.doi.org/10.1136/bmjopen-2018-026308
4. Boots LM, de Vugt ME, van Knippenberg RJ, Kempen GI, Verhey FR. A systematic review of Internet-based supportive interventions for caregivers of patients with dementia. *Int J Geriatr Psychiatry*. 2014;29(4 PG-331-344):331-344. doi:https://dx.doi.org/10.1002/gps.4016
5. Bourne P, Camic PM, Crutch SJ. Psychosocial outcomes of dyadic arts interventions for people with a dementia and their informal caregivers: A systematic review. *Heal Soc Care Community*. 2021;29(6):1632-1649. doi:10.1111/hsc.13267
6. Bunn F, Goodman C, Pinkney E, Drennan VM. Specialist nursing and community support for the carers of people with dementia living at home: An evidence synthesis. *Heal Soc Care Community*. 2016;24(1):48-67. doi:10.1111/hsc.12189
7. Cheng ST, Li KK, Losada A, et al. The effectiveness of nonpharmacological interventions for informal dementia caregivers: An updated systematic review and meta-analysis. *Psychol Aging*. 2020;35(1 PG-55-77):55-77. doi:https://dx.doi.org/10.1037/pag0000401
8. Corry M, Neenan K, Brabyn S, Sheaf G, Smith V. Telephone interventions, delivered by healthcare professionals, for providing education and psychosocial support for informal caregivers of adults with diagnosed illnesses. *Cochrane Database Syst Rev*. 2019;5(PG-CD012533):CD012533. doi:https://dx.doi.org/10.1002/14651858.CD012533.pub2
9. Cuthbert CA, King-Shier K, Ruether D, Tapp DM, Culos-Reed SN. What is the state of the science on physical activity interventions for family caregivers? A systematic review and RE-AIM evaluation. *J Phys Act Heal*. 2017;14(7):578-595. doi:10.1123/jpah.2016-0280
10. Dam AE, de Vugt ME, Klinkenberg IP, Verhey FR, van Boxtel MP. A systematic review of social support interventions for caregivers of people with dementia: Are they doing what they promise? *Maturitas*. 2016;85(PG-117-130):117-130. doi:https://dx.doi.org/10.1016/j.maturitas.2015.12.008
11. Doyle KL, Toepfer M, Bradfield AF, et al. Systematic review of exercise for caregiver-care recipient dyads: What is best for spousal caregivers - Exercising together or not at all? *Gerontologist*. 2021;61(6):E283-E301. doi:10.1093/geront/gnaa043
12. Du Preez J, Millsteed J, Marquis R, Richmond J. The Role of Adult Day Services in Supporting the Occupational Participation of People with Dementia and Their Carers: An Integrative Review. *Healthcare*. 2018;6(2 PG-08):8. doi:https://dx.doi.org/10.3390/healthcare6020043
13. Forster A, Brown L, Smith J, et al. Information provision for stroke patients and their caregivers. *Cochrane Database Syst Rev*. Published online November 14, 2012. doi:10.1002/14651858.cd001919.pub3
14. Gabriel I, Creedy D, Coyne E. A systematic review of psychosocial interventions to improve quality of life of people with cancer and their family caregivers. *Nurs Open*. 2020;7(5):1299-1312. doi:10.1002/nop2.543
15. Goeman D, Renehan E, Koch S. What is the effectiveness of the support worker role for people with dementia and their carers? A systematic review. *BMC Health Serv Res*. 2016;16(1). doi:10.1186/s12913-016-1531-2
16. González-Fraile E, Ballesteros J, Rueda JR, Santos-Zorrozúa B, Solà I, McCleery J. Remotely delivered information, training and support for informal caregivers of people with dementia. *Cochrane Database Syst Rev*. 2021;2021(1). doi:10.1002/14651858.CD006440.pub3
17. Greenwood N, Pelone F, Hassenkamp AM. General practice based psychosocial interventions for supporting carers of people with dementia or stroke: a systematic review. *BMC Fam Pract*. 2016;17(PG-3):3. doi:https://dx.doi.org/10.1186/s12875-015-0399-2
18. Li G, Yuan H, Zhang W. The Effects of Mindfulness-Based Stress Reduction for Family Caregivers: Systematic Review. *Arch Psychiatr Nurs*. 2016;30(2):292-299. doi:10.1016/j.apnu.2015.08.014
19. Heckel L, Heynsbergh NL, Livingston PM. Are cancer helplines effective in supporting caregivers? A systematic review. *Support Care Cancer*. 2019;27(9):3219-3231. doi:10.1007/s00520-019-04807-z
20. Hopkinson MD, Reavell J, Lane DA, Mallikarjun P. Cognitive Behavioral Therapy for Depression, Anxiety, and Stress in Caregivers of Dementia Patients: A Systematic Review and Meta-Analysis. *Gerontologist*. 2019;59(4 PG-343-362):e343-e362. doi:https://dx.doi.org/10.1093/geront/gnx217
21. Hopwood J, Walker N, McDonagh L, et al. Internet-Based Interventions Aimed at Supporting Family Caregivers of People With Dementia: Systematic Review. *J Med Internet Res*. 2018;20(6 PG-e216):e216. doi:https://dx.doi.org/10.2196/jmir.9548
22. Yoon Irons J, Garip G, Cross AJ, Sheffield D, Bird J. An integrative systematic review of creative arts interventions for older informal caregivers of people with neurological conditions. *PLoS One*. 2020;15(12 December). doi:10.1371/journal.pone.0243461
23. Lee M, Ryoo JH, Crowder J, Byon HD, Wiiliams IC. A systematic review and meta-analysis on effective interventions for health-related quality of life among caregivers of people with dementia. *J Adv Nurs*. 2020;76(2 PG-475-489):475-489. doi:https://dx.doi.org/10.1111/jan.14262
24. Lins S, Hayder-Beichel D, Rucker G, et al. Efficacy and experiences of telephone counselling for informal carers of people with dementia. *Cochrane Database Syst Rev*. 2014;(9 PG-CD009126):CD009126.

For peer review only - http://bmjopen.bmj.com/site/about/guidelines.xhtml

doi:https://dx.doi.org/10.1002/14651858.CD009126.pub2
25. Liu Z, Chen Q lin, Sun Y ying. Mindfulness training for psychological stress in family caregivers of persons with dementia: A systematic review and meta-analysis of randomized controlled trials. *Clin Interv Aging*. 2017;12:1521-1529. doi:10.2147/CIA.S146213
26. Lucero RJ, Fehlberg EA, Patel AGM, et al. The effects of information and communication technologies on informal caregivers of persons living with dementia: A systematic review. *Alzheimer's Dement Transl Res Clin Interv*. 2019;5:1-12. doi:10.1016/j.trci.2018.11.003
27. Maayan N, Soares-Weiser K, Lee H. Respite care for people with dementia and their carers. *Cochrane Database Syst Rev*. 2014;2014(1). doi:10.1002/14651858.CD004396.pub3
28. Maffioletti VLR, Baptista MAT, Santos RL, Rodrigues VM, Dourado MCN. Effectiveness of day care in supporting family caregivers of people with dementia: A systematic review. *Dement Neuropsychol*. 2019;13(3 PG-268-283):268-283. doi:https://dx.doi.org/10.1590/1980-57642018dn13-030003
29. Miles L, McCausland BMS, Patel HP, Amin J, Osman-Hicks VC. A systematic review of the provision and efficacy of patient and carer information and support (PCIS) interventions for patients with dementia and their informal carers. *Aging Clin Exp Res*. 2020;32(12):2439-2448. doi:10.1007/s40520-019-01428-8
30. Minshall C, Pascoe MC, Thompson DR, et al. Psychosocial interventions for stroke survivors, carers and survivor-carer dyads: a systematic review and meta-analysis. *Top Stroke Rehabil*. 2019;26(7 PG-554-564):554-564. doi:https://dx.doi.org/10.1080/10749357.2019.1625173
31. Parkinson B, Lawrence M, McElhinney E, Booth J. Mindfulness for people with long-term conditions and their family caregivers: A systematic review. *Complement Ther Clin Pract*. 2019;34:76-86. doi:10.1016/j.ctcp.2018.10.019
32. Piersol CV, Canton K, Connor SE, Giller I, Lipman S, Sager S. Effectiveness of Interventions for Caregivers of People With Alzheimer's Disease and Related Major Neurocognitive Disorders: A Systematic Review. *Am J Occup Ther*. 2017;71(5):p1-7105180020. doi:10.5014/ajot.2017.027581
33. Pritchard E, Cussen A, Delafosse V, Swift M, Jolliffe L, Yeates H. Interventions supporting caregiver readiness when caring for patients with dementia following discharge home: A mixed-methods systematic review. *Australas J Ageing*. 2020;39(3 PG-239-250):e239-e250. doi:https://dx.doi.org/10.1111/ajag.12765
34. Pucciarelli G, Lommi M, Magwood GS, et al. Effectiveness of dyadic interventions to improve stroke patient-caregiver dyads' outcomes after discharge: A systematic review and meta-analysis study. *Eur J Cardiovasc Nurs*. 2021;20(1):14-33. doi:10.1177/1474515120926069
35. Reilly S, Miranda-Castillo C, Malouf R, et al. Case management approaches to home support for people with dementia. *Cochrane Database Syst Rev*. 2015;2017(6). doi:10.1002/14651858.CD008345.pub2
36. Shaw C, McNamara R, Abrams K, et al. Systematic review of respite care in the frail elderly. *Health Technol Assess*. 2009;13(20 PG-1-224, iii):1-224, iii. doi:https://dx.doi.org/10.3310/hta13200
37. Shim M, Tilley JL, Im S, Price K, Gonzalez A. A Systematic Review of Mindfulness-Based Interventions for Patients with Mild Cognitive Impairment or Dementia and Caregivers. *J Geriatr Psychiatry Neurol*. 2021;34(6):528-554. doi:10.1177/0891988720957104
38. Sin J, Henderson C, Spain D, Cornelius V, Chen T, Gillard S. eHealth interventions for family carers of people with long term illness: A promising approach? *Clin Psychol Rev*. 2018;60:109-125. doi:10.1016/j.cpr.2018.01.008
39. Smith R, Greenwood N. The impact of volunteer mentoring schemes on carers of people with dementia and volunteer mentors: a systematic review. *Am J Alzheimer's Dis Other Dementias*. 2014;29(1 PG-8-17):8-17. doi:https://dx.doi.org/10.1177/1533317513505135
40. Smith TO, Pearson M, Pfeiffer K, Crotty M, Lamb SE. Caregiver Interventions for Adults Discharged from the Hospital: Systematic Review and Meta-Analysis. *J Am Geriatr Soc*. 2019;67(9):1960-1969. doi:10.1111/jgs.16048
41. Teahan Á, Lafferty A, McAuliffe E, et al. Psychosocial Interventions for Family Carers of People With Dementia: A Systematic Review and Meta-Analysis. *J Aging Health*. 2020;32(9):1198-1213. doi:10.1177/0898264319899793
42. Treanor CJ, Santin O, Prue G, et al. Psychosocial interventions for informal caregivers of people living with cancer. *Cochrane Database Syst Rev*. 2019;2019(6). doi:10.1002/14651858.CD009912.pub2
43. Vandepitte S, Van Den Noortgate N, Putman K, Verhaeghe S, Verdonck C, Annemans L. Effectiveness of respite care in supporting informal caregivers of persons with dementia: a systematic review. *Int J Geriatr Psychiatry*. 2016;31(12):1277-1288. doi:10.1002/gps.4504
44. Waldron EA, Janke EA, Bechtel CF, Ramirez M, Cohen A. A systematic review of psychosocial interventions to improve cancer caregiver quality of life. *Psychooncology*. 2013;22(6 PG-1200-1207):1200-1207. doi:https://dx.doi.org/10.1002/pon.3118
45. Wang S, Bressington DT, Leung AYM, Davidson PM, Cheung DSK. The effects of bibliotherapy on the mental well-being of informal caregivers of people with neurocognitive disorder: A systematic review and meta-analysis. *Int J Nurs Stud*. 2020;109. doi:10.1016/j.ijnurstu.2020.103643
46. Wiegelmann H, Speller S, Verhaert LM, Schirra-Weirich L, Wolf-Ostermann K. Psychosocial interventions to support the mental health of informal caregivers of persons living with dementia - a systematic literature review. *BMC Geriatr*. 2021;21(1 PG-94):94. doi:https://dx.doi.org/10.1186/s12877-021-02020-4
47. Zabihi S, Lemmel FK, Orgeta V. Behavioural Activation for Depression in Informal Caregivers: A Systematic Review and Meta-Analysis of Randomised Controlled Clinical Trials. *J Affect Disord*. 2020;274(PG-1173-1183):1173-1183. doi:10.1016/j.jad.2020.03.124

For peer review only - http://bmjopen.bmj.com/site/about/guidelines.xhtml

Supplementary Table 7. Summary of findings for interventions involving case management

First author, year	Summary of findings on effectiveness
Goeman, 2016	Inconclusive findings with similar number of studies reporting either positive effects or no effects.
Greenwood, 2016	No significant effect.
Hopwood, 2018	No significant effect. However, some multicomponent interventions in this review show promise for reducing caregivers' depressive symptoms and anxiety.
Lee, 2020	No significant effect: Hedges G was 0.135 (95% CI=-0.076, 0.346, *p*=0.210). However, multicomponent interventions were effective: Hedges G 0.255 (95% CI=0.054, 0.457, *p*=0.013).
Lucero, 2019	Telephone-based intervention showed significant decrease in depression.
Piersol, 2017	No significant effect.
Reilly, 2015	Significant reduction in depression and short-term health (MD=-0.08, 95% CI=-0.16, -0.01, n=2888, *p*=0.03), but no effect on longer follow-ups.
Pucciareli, 2020	No significant effect: SMD=-0.19, 95% CI=-0.40, 0.00, *p*=0.05.

Abbreviations: CI, confidence interval; SMD, standardized mean difference; MD, mean difference.

For peer review only

Supplementary Table 8. Summary of findings for psychosocial, psychoeducational, and skills-building interventions

First author, year	Summary of findings on effectiveness
**Reviews that included both psychosocial and educational interventions (n=19)**	
Akarsu, 2019	In random effects MA, interventions (psychological, multicomponent, and educational) to address depression in ethnic minority caregivers relative to the control condition were associated with an overall mean reduction in depression scores (SMD=-0.17, 95% CI=-0.29, -0.05, *p*=0.005). Heterogeneity was negligible (I2=0.0%, *p*=0.547).
Gonzalez-Fraile, 2021	Remotely delivered interventions involving training, support, or both (with or without information) do not affect caregiver depressive symptoms (SMD=-0.05, 95% CI=-0.22, 0.12, I2=9%) or caregiver HR-QoL (SMD=0.10, 95% CI=-0.13, 0.32, I2=0%). However, these interventions may result in a slight improvement in caregiver depressive symptoms (SMD=-0.25, 95% CI=-0.43, -0.06, I2 = 53%) compared to a control condition of information alone. Interventions may result in little or no difference in caregiver HR-QoL when compared with information alone (SMD=-0.03, 95% CI=-0.28, 0.21, I2=0%).
Greenwood, 2016	One study reported no significant effects in the proportion of caregivers with scores of CES-D ≥16 (i.e., at risk of depression) between the intervention and control groups. Another study reported significant positive changes over time (*p*=0.009) for improving depression scores.
Hopwood, 2018	Overall, studies assessing psychological support suggested a positive effect on a variety of factors, including improving caregiver distress, depression, anxiety, and strain. Some multiple-component interventions showed promise in reducing stress, anxiety, and depressive symptoms for family caregivers and in increasing self-efficacy.
Lee, 2020	CBT was effective, Hedges G=0.767 (95% CI=0.142, 1.391, *p*=0.016); Psychoeducation was not effective, Hedges G=0.163 (95% CI=-0.001, 0.328, *p*=0.052); Social support and cognitive rehabilitation were not effective, G=0.231 (95% CI=-0.104, 0.567, *p*=0.176 and G=0.010 (95% CI=-0.208, 0.229, *p*=0.926), respectively. However, multicomponent interventions were effective: Hedges G=0.255 (95% CI=0.054, 0.457, *p*=0.013).
Lins, 2014	Without educational material and workbook: significant pooled difference in depressive symptoms between the telephone counselling group and the control group (SMD=0.32, 95% CI=0.01, 0.63, *p*=0.04). With educational material and workbook: reduction of depressive symptoms in the intervention group and an increase in the control group after months: -4.1% points in the intervention group (from 7.20% to 3.10%) and 0.56% points in the control group (from 7.44% to 8%). The difference between the groups was statistically significant (*p*=0.01).
Piersol, 2017	Communication skills training: increased QoL and well-being; Coaching: reduced stress and depression; Psychoeducation: improved QoL, well-being, reduced depression; Group interventions: strong evidence indicates that in person caregiver support groups led by professionals improved caregiver well-being and reduced depression; CBT: compelling evidence that interventions focused on cognitive reframing and skills training reduced caregiver depression and anxiety.
Teahan, 2020	Interventions were shown to have a significant effect (*p*=0.002) on caregivers' depression levels pre- and postintervention (SMD=-0.36, 95% CI=-0.60, -0.13, 11 trials, N=1856 participants). The interventions were shown to have a significantly small to moderate effect size (*p*<0.01) on the general health scores of caregivers pre- and postintervention (SMD=0.34, 95% CI=0.18, 0.51, 7 trials, N=1503 participants). Although the overall effect on QoL was not statistically significant (*p*=0.12), there was a trend in favor of intervention (SMD=0.63, 95% CI=-0.16, 1.43, 3 interventions, N=201).
Wiegelmann, 2021	4 out of 11 psychoeducational interventions described positive results, while 7 reported no significant effect. 1 out of 4 counselling interventions reported improvement in depression. 4 out of six CBT interventions reported positive effects. 3 out of 8 psychoeducational interventions reported improvement in QoL. 1 out of 6 counselling interventions improved QoL. 1 CBT intervention measuring QoL failed to report significant effects. Neither of the 2 befriending interventions reported positive changes in QoL.
Zabihi, 2020	MA of effects of behavioral activation showed that results significantly favored intervention in reducing caregiver depressive symptoms at post-treatment (8 studies, 815 participants, SMD=-0.68, 95% CI=-1.14,

For peer review only

	-0.22). There was high heterogeneity between studies (I2=89%). Long-term effects were also favorable (2 studies, 235 participants, SMD=-0.99, 95% CI=-1.26, -0.71, I2= 92%. Behavioral activation was also associated with decreased risk of depression diagnosis (3 studies, 854 participants, OR=0.35, 95% CI=0.19, 0.67, efficacy at 3-12 months) and was superior to psychoeducation in one study.
Gabriel, 2020	All 12 studies assessed psychological/emotional well-being; 4 studies report statistically significant improvement for dyads, 2 studies report significant improvement for caregivers. Remaining 6 studies report no significant change. As for physical well-being, all 12 studies assessed it; 5report significant improvement for dyads, while 1 reports it for care receivers only, 6 report no change.
Heckel, 2019	No significant effect on depression (1 study), another study showed positive effects for reducing emotional distress.
Treanor, 2019	Considering the pooled and unpooled findings, psychosocial interventions may improve slightly QoL immediately post-intervention, but this benefit may not be maintained in the longer term (6-2 months later). Pooled and unpooled findings indicated that psychosocial interventions probably have little to no effect on depression, anxiety or emotional distress for caregivers. Evidence for physical outcomes is insufficient.
Waldron, 2013	Nil to small effects on QoL outcomes were found in the 4 studies where QoL effect sizes could be calculated, and 2 of these reported significant changes in QoL.
Corry, 2019	There is little or no difference between telephone support interventions and usual care for QoL at the end of intervention (SMD=-0.02, 95% CI=-0.24, 0.19, 4 studies, 364 caregivers) and at short-term follow-up (3 months) (SMD=0.00, 95% CI=-4.43, 4.43, 1 study, 128 caregivers). The effects of telephone interventions on depression at the end of intervention were uncertain (SMD=-0.37, 95% CI=-0.70, -0.05, 9 studies, 792 caregivers). Telephone interventions may have little or no effect at medium-term follow-up (3-6 months) (SMD=-0.05, 95% CI=-0.56, 0.45, 3 studies, 227 caregivers). Telephone interventions compared with usual care may slightly decrease anxiety levels at the end of intervention and short-term follow-up (2 studies, no MA). Telephone support interventions, when compared to usual care may have little or no effect on caregiver physical health at the end of intervention (SMD=-0.09, 95% CI=-0.35, 0.17, 2 studies, 248 caregivers).
Sin, 2018	Of those studies that assessed effectiveness of interventions, 44% reported equivocal findings (no effect), 12% saw negative effects (control group more favorable than intervention) and only 32% reported positive effects. While caregivers reported positive experiences in using the interventions (as per qualitative findings), no significant changes in their objective outcomes were identified.
Forster, 2012	Out of 3 RCTs measuring depression, 2 showed no significant effect, while 1 reported less depression in the intervention group. Out of 4 RCTs measuring QoL, 3 report no effects, while 1 reported higher QoL in the intervention group.
Minshall, 2019	Based on MA of 6 RCTs measuring depression, the effect is insignificant (pooled effect size=-0.20, 95% CI -0.40, 0.00, n=279 in intervention group, n=282 in control group). One trial reported improvements in caregiver QoL.
Pucciarelli, 2020	Caregivers who received an educational intervention were found to have lower depression levels than those in the control group, although no significant differences were observed between these two groups (SMD=-0.19, 95% CI=-0.40, 0.00, *p*=0.05).
**Reviews that included only psychosocial (n=2) or only educational (n=2) interventions**	
Bennett, 2019 (occupational therapy)	Data from six studies that measured the emotional distress family caregivers felt were combined, and demonstrated a small, statistically significant result in favor of those receiving occupational therapy (SMD=-0.23, 95% CI=-0.42, -0.05, I2=41%). Only 3 studies measured overall QoL of the caregivers of people with dementia. Pooled data from 2 of these studies produced a large, significant between-group difference after the intervention (SMD=0.99, 95% CI=0.66, 1.33, I2=2%).
Smith, 2019 (training)	There was no benefit on caregiver HR-QoL compared with the control group at 3-6 months (SMD=0.20; 95% CI=-1.12, 1.52, N=638, I2=98%) or 12 months (SMD=0.46, 95% CI=-0.34, 1.27, N=415, I2=94%). There was no benefit from the caregiver intervention on caregiver depression compared with the control group at 3 to 6 months (SMD=0.03,95% CI=-0.08, 0.14, N=1239, I2=0%) or 12 months (SMD=-0.71, 95% CI=-2.23, 0.81, N=613, I2=99%). For anxiety, 1

For peer review only - http://bmjopen.bmj.com/site/about/guidelines.xhtml

	study reported significant effect, while another reported no improvement.
Hopkinson, 2019 (psychosocial)	Caregivers receiving CBT demonstrated statistically significant reductions in depression (n=12; 995 participants; SMD=-0.34; 95% CI=-0.47, -0.21, *p*<0.001 relative to comparator groups immediately after the intervention endpoint and in 3 months (n=3; SMD=-0.99, 95% CI=-1.35, -0.64, *p*<0.001). There was no significant difference in caregiver anxiety (n=10, 829 participants, SMD=0.10; 95% CI=-0.18, 0.39, *p*=0.47).
Wang, 2020 (psychosocial)	For depression, the overall effect for the 2 types of bibliotherapy was significant at Z=1.99 (random effect model, SMD=-0.74, 95% CI=-1.47, -0.01, *p*=0.05). However, when considered separately, only the video based bibliotherapy significantly reduced depression at Z=2.78 (random effect model, SMD=-2.11, 95% CI=-3.6, -0.62, *p*=0.005). For anxiety, the heterogeneity of the 3 studies included in MA was low (I2=22%), the overall effect of bibliotherapy on anxiety was significant at Z=2.30, *p*=0.02 (random effect model, SMD=-0.22, 95% CI=-0.41, -0.33).

Abbreviations: RCT, randomized controlled trial; CI, confidence interval; SMD, standardized mean difference; CBT, cognitive behavioral therapy; QoL, quality of life; HR-QoL, health-related quality of life; MA, meta-analysis.

For peer review only

For peer review only - http://bmjopen.bmj.com/site/about/guidelines.xhtml

Supplementary Table 9. Summary of findings for interventions involving respite care

First author, year	Summary of findings on effectiveness
Maayan, 2019	Analysis of the available data showed no significant effects on caregiver outcomes when respite care was compared with no respite care in 3 studies.
Maffioletti, 2019	5 studies reported positive effects on depression, 3 on psychological/psycho-somatic complaints, and 4 studies also report improved health status and greater psychological well-being. 2 studies report no changes in caregiver outcomes.
Vandepitte, 2016	Reported benefits of day care provision for caregivers were small and mixed (33% were effective). The only included in-home respite care program indicated some beneficial effects for caregivers.
Shaw, 2009	MA showed insignificant positive effect in favor of respite care at short term, but no effect in long term. Evidence from narratively reviewed studies indicated that respite does not have a significant impact on psychological well-being or depression when compared with normal care. Few studies (with lower quality of design, not in MA) reported decrease in depression, but at short follow-up times. MA of randomized/quasi-experimental studies measuring caregiver anxiety as an outcome did not demonstrate any significant effects.

Abbreviations: MA, meta-analysis.

For peer review only

Supplementary Table 10. Summary of findings for interventions involving relaxation, physical activity, or leisure

First author, year	Summary of findings on effectiveness
Cheng, 2020	Miscellaneous interventions were shown to reduce stress and depressive symptoms. For this group of interventions, Hedges G=-0.49, n=12, N=758. Interestingly, in this review, multicomponent and miscellaneous interventions had the largest effects.
Lee, 2020	MA of the 2 studies showed that the Hedges G was 0.576 (95% CI=0.035, 1.118, *p*=0.037), indicating that there was a statistically significant difference between the control group and the intervention group on improving caregiver HR-QoL.
Piersol, 2017	Exercise programs showed improved caregiver physical health and decreased stress. Adapted leisure programs showed improved well-being.
Irons, 2020	For outcomes of interest, pre-post effect sizes revealed some benefits of the intervention: large effect sizes were detected for singing on well-being (Cohen's d=1.04), and positive-negative mood (Cohen's d=1.29); and for music making on relaxation (Cohen's d=1.91), comfort (Cohen's d=1.74), and happiness (Cohen's d=1.19). Some studies indicated decreases in some aspects of well-being which were not consistent with other aspects reported. For example, one study reported increases in stress, but decreases in anxiety and depression.
Cuthbert, 2017	Across all studies, statistically significant results for the following outcomes in the physical activity groups were reported: (1) decrease in depression rates; (2) decrease in anger scores; (3) improvement in sleep quality; (4) lower systolic and diastolic blood pressure reactivity.
Doyle, 2020	Results were mixed across and within DyEx and DySplit studies. Of the DyEx studies, 6 examined caregiver psychosocial well-being. Beneficial outcomes in 4 studies indicated significant improvements in mental health and QoL of caregivers. In 3 DySplit interventions, caregivers experienced significant improvements to mental health when they were offered non-exercise interventions of either respite or a dyadic support group, while their care receivers exercised. 3 DyEx studies measured caregiver physical health. A resistance training intervention noted significant increases in muscle mass, strength, and physical function, but no significant difference in gait speed. Self-reported physical outcomes were equivocal.

Abbreviations: CI, confidence interval; QoL, quality of life; HR-QoL, health-related quality of life; DyEx, dyadic interventions; DySplit, non-dyadic interventions.

For peer review only

Supplementary Table 11. Summary of findings for interventions involving mindfulness-based activities

First author, year	Summary of findings on effectiveness
Cheng, 2020	Mindfulness-based interventions reduced depressive symptoms. For subjective well-being G=0.31, k=6, N=212. For depression G=-0.58, k=7, N=258.
Liu, 2018	MBSR vs active controls immediately after intervention: MBSR decreased depressive symptoms of caregivers compared with the active-control interventions: SMD=-0.63 (95% CI=-0.98, -0.28, p<0.001). MBSR could reduce caregivers' anxiety compared with the active-control group immediately after the intervention period: MD=-7.50 (95% CI=-13.11, -1.89, p=0.009). MBSR vs inactive controls immediately after intervention: no clear effect on depressive symptoms of caregivers in the MBSR group compared with the inactive controls immediately after the intervention period. MBSR may reduce caregivers' levels of anxiety compared with the inactive control immediately after the intervention period MD=-7.27 (95% CI=-14.92, 0.38, p=0.06. MBSR vs controls at follow-up: compared with the active-control intervention, the results for depressive symptoms suggested that there may be little or no effect of MBSR MD=-0.16 (95% CI=-0.71, 0.39, p=0.57). Compared with the inactive control, the results for caregivers' depressive symptoms slightly favored MBSR MD=-3.00 (95% CI=-8.52, 2.52, p=0.29).
Shim, 2020	3 studies show that cognitive functioning was significantly improved in MBI conditions compared to both active and passive control conditions. 4 studies reported statistically significant improvements in self-reported measures of stress or distress in the MBI condition at post-treatment or follow-up with treatment effects in the small to medium range (G=-0.078 to -0.602). 3 out of 7 studies examining depression/anxiety reported relative improvements. When compared with passive control interventions, caregivers in MBIs reported significant improvements in QoL. Long term effects were not that pronounced. No treatment differences in outcomes were found at 3- and 9-month follow-up assessments.
Parkinson, 2019	Some improvements were noted for patients' and their caregivers' anxiety scores following intervention, but these changes were not significant at post treatment or at follow-up. 1 study provided a narrative account of results and reported no change in anxiety scores following intervention for participants or family caregivers. Significant (p=0.01) improvements in the tension/anxiety score were reported in 1 study. Caregivers experienced a mean reduction depression score in 5 studies, but the improvements in depression were often small and only significant in 3 studies.

Abbreviations: MBSR, Mindfulness-Based Stress Reduction; MBI, Mindfulness-Based Interventions; MCI, mild cognitive impairment; SMD, standardized mean difference; MD, mean difference; CI, confidence interval.

For peer review only

Supplementary Table 12. Coding table for qualitative data

Theme (and definition)	Category (and definition)	Sub-category (and definition)	Fourth level construct codes	Third level construct verbatim	References
**Intervention outcomes:** This category describes potential beneficial and/or harmful effects to the caregiver from participating in the interventions	**Health outcomes:** Health benefits experienced and expressed by caregivers because of participating in support interventions.	**NA**	Education/skills: hospital skills-building brings less anxiety, depression, burden, better quality of life	When interventions are provided to the caregiver in the context of the hospital setting to enhance these skills, there is a likelihood of reducing anxiety, depression, caregiver burden and improving QoL.	Pritchard
			Mindfulness: benefits include relaxation, less depression, less worry, and anxiety	Triangulated qualitative and quantitative data suggests MBIs can improve relaxation, ease anxiety, and reduce depression for people with LTCs and their family caregivers. […] The reported psychological benefits included increased relaxation, reduced worry, and reductions in anxiety.	Parkinson
**Intervention outcomes:** This category describes potential beneficial and/or harmful effects to the caregiver from participating in the interventions	**Social outcomes:** intervention outcomes related to impact on caregiver's social life, day-to-day routine and relationship with care receiver and other people.	**Practical social outcomes:** changes in caregivers' day-to-day routine, resulting from acquiring new skills or knowledge.	Case management: nurse and carer working together decreases burden	A shared approach to care was found to be vital in decreasing burden among family members.	Goeman
			Case management: prevents crises	Case management offered potential benefit to people with dementia, their carers and community-based professionals through continuity of care by a named trusted individual that could act proactively to prevent a crisis.	Goeman
			Case management: admiral nurses have good relationship with carers and care receivers	Admiral nurse: Positive outcomes in the carer/key worker relationship to be linked to the quality of the relationship and involve the carer and professional care worker actively including and working with the person with dementia.	Goeman
			Psychoeducation: brings satisfaction, coping skills, competence, confidence, less burden, less loneliness	Increased satisfaction with support, coping skills, caregiving competence, confidence, and decreased burden and loneliness.	Smith
			Psychoeducation: brings increased knowledge, coping, confidence, less isolation	Increased knowledge of the disease and caring was described in 3 papers, in addition to increased coping with caregiving (2 papers) confidence in caregiver skills (2 papers) and reduced feelings of isolation (1 paper).	Boots
			Education/skills: Strong relationship between health staff and carers needed for continued informal care	When health professionals build strong relationships with the caregiver, they are more likely to be able to support the readiness of the caregiver to continue their role.	Pritchard
			Education/skills: when needs are met, coping is better	Caregivers identified when personal characteristics of both patient and caregiver are considered, caregivers are included as an integral part of the team (as they requested), and they receive timely and effective information, the level of stress is likely to reduce, and their ability to cope to be ready for discharge is likely to increase.	Pritchard

For peer review only - http://bmjopen.bmj.com/site/about/guidelines.xhtml

			Education/skills: carers' coping mechanisms promoted	The interventions addressed the need for clear information about the disease, identification of psychological response, development of coping mechanisms and assessment of the home environment.	Pritchard
			Counselling: counsellors help with seemingly helpless situations	Being able to describe and discuss problematic situations helps carers in their daily lives when they do not know how what to do next, because, from their perspective, they have tried everything but have not been able to resolve the situation.	Lins
			Counselling: counsellors knowledgeable about services is helpful	Carers find the information provided helpful and are grateful for it. The information and advice help them to do certain things better, which leads to them feeling happier with themselves.	Lins
			Respite: free time used for leisure or chores	The respite time gained through this type of care tended to be used for necessary everyday chores. Also used to carry out pleasurable activities on a regular basis.	Shaw
			Respite: carers more in control of time	Shorter-term respite such as day care was felt to give structure to the carer's week along with a sense of normality as the free time matched the ebb and flow of caregiving activities.	Shaw
			Respite: use of service can be more emotionally and physically demanding than not use	There were many hassles (i.e., inconveniences, irritations and frustrations that were troublesome to deal with) involved in the preparation for respite care. These hassles were costly in terms of the physical and emotional energy involved in dealing with them, and these costs were weighed against the benefits received from respite.	Shaw
			Respite: brings organized routine	Regular attendance is also perceived by carers to provide their care recipients with a sense of structure and routine to their daily lives.	Du Preez
			Creative arts: benefits include learning, stimulation, normalizing care receivers' lives	Reported benefits included new learning, stimulation and the accessibility of the activities, which creates a sense of normalcy and altered perceptions about PWD's abilities.	Bourne
			Creative arts: artmaking helps with coping and resilience	Artmaking was seen to cope and practice resilience, where this approach allowed caregivers to make the most of their lives and to deal with an uncertain future: "It slightly reminds me of the turmoil but at the same time it says to me yes, you got through it […] Because they [a series of paintings] were part of the process of me becoming well again."	Irons
**Intervention outcomes:** This category describes potential beneficial and/or harmful effects to the caregiver from	**Social outcomes:** intervention outcomes related to impact on caregiver's social life, day-to-day routine and relationship	**Emotional social outcomes:** changes in feelings and emotional reflections of caregivers, resulting from an intervention.	Psychoeducation: Online intervention reduces stress	Caregivers subjectively identified that the online intervention helped them cope with the stress of caregiving.	Sin
			Education/skills: When needs are met, stress is reduced	Caregivers identified when personal characteristics of both patient and caregiver are considered, caregivers are included as an integral	Pritchard

For peer review only - http://bmjopen.bmj.com/site/about/guidelines.xhtml

participating in the interventions	with care receiver and other people.		part of the team (as they requested), and they receive timely and effective information, the level of stress is likely to reduce, and their ability to cope to be ready for discharge is likely to increase.	
		Counselling: carers appreciate conversations beyond their nursing duties	Carers also find it helpful to have conversations about other things that do not remind them of their nursing duties.	Lins
		Psychosocial: Benefits of peer support include less discomfort and satisfaction	Qualitative measures on well-being reported improvements on subjective satisfaction, and reduced feelings of discomfort and embarrassment.	Dam
		Psychosocial: group support brings relief through sharing	Qualitative interview data on support groups for only caregivers demonstrated a sense of relief through sharing problems and new social contact.	Dam
		Psychosocial: peer support brings emotional support and enjoyment	Qualitative social support outcomes demonstrated reduced feelings of social isolation, increased emotional support, and mutual sharing and enjoyment after receiving peer support.	Dam
		Counselling: simple chit chat brings relief	The participants in the control group had conversations only about general topics such as the weather, television, movies, news, or social activities. Such a conversation about common topics was found to be a helpful alternative to relieve carers.	Lins
		Counselling: self-expression beneficial	Carers benefit from being able to express their helplessness.	Lins
		Respite: respite gives physical break but not mental break/relaxation	The qualitative review also pointed out that a physical break from the care recipient was probably not sufficient in itself to provide the mental break that was needed by most carers to improve their well-being. A mental break meant freedom from worry and total disengagement from the caring role. This was only achieved in the knowledge that the care recipient was happy, safe and well cared for.	Shaw
		Respite: caregivers feel guilty to not care	The strong commitment to caring and eventual realisation that they cannot cope on their own leads not only to feelings of failure but also to feelings of guilt. The negative connotations of respite and the feelings of abandonment also contribute to guilt.	Shaw
		Respite: sense of failure when admitting need of help	The types of attitudes discussed above that drive the desire to maintain the care recipient at home can lead to a sense of failure when a need for support is acknowledged, particularly in the presence of negative attitudes to respite care itself.	Shaw
		Respite: feelings of guilt and perceived abandonment with respite	In the early stages it was felt by carers that attendance at day care could confront a care recipient's denial of their condition, and so a pretence of normality is maintained as long as possible to maintain	Shaw

For peer review only

For peer review only - http://bmjopen.bmj.com/site/about/guidelines.xhtml

	the integrity of the care recipient's self-image. The outcome in relation to many of the issues described above is the carer's feeling of guilt.	
Respite: more guilt among women	If respite is seen as benefiting only the carer this will be viewed in a selfish light, which can trigger guilt, particularly in women who have spent their lives caring for others	Shaw
Respite improves mood and engagement with care recipient	Carers reported increased engagement with their care recipients upon returning from adult day service attendance. This was due to their improved mood and them sharing interesting events that occurred throughout the day.	Du Preez
Creative arts: singing brings enjoyment	All six studies included comments on improved mood or well-being and three on the extended impact in terms of mood, the enjoyment of singing at home and socializing with other members outside of the group.	Bourne
Creative arts: familiar songs promote reminiscence	Participants also reported gaining enjoyment from singing familiar songs that facilitated reminiscence.	Bourne
Creative arts: benefits include self-esteem, positive affect, enjoyment, relaxation, energy, empowerment, confidence	Experiences including improved personal wellbeing, self-esteem, positive affect, enjoyment, relaxation, feeling energized, empowered and having confidence were captured in this meta-theme.	Irons
Creative arts: socioemotional connection brings sense of belonging	Papers highlighted the socio-emotional connection afforded by participation in weekly creative interventions, which gave both caregiver and care-recipient something to look forward to attending and be part of.	Irons
Creative arts: practicing skills gives cognitive stimulation and engagement	The cognitive impact of intervention participation included cognitive stimulation and engagement, through the practice of previously acquired skills (such as demonstrating musical knowledge).	Irons
Creative arts: reminiscence promoted	Creative interventions were useful for reactivating memories and afforded opportunities for memory recall of events.	Irons
Creative arts: dyads working together feel hopeful	In an intervention where caregivers and care-recipients met to create a database, this evoked positive feelings and hope for the caregivers.	Irons
Creative arts: carers reclaim identity through creative interventions	Creative interventions that allow for self-expression offer opportunities to caregivers to reclaim and transform their identity.	Irons

For peer review only

Creative arts: song hearing fosters emotions	In some cases, the meaning behind song lyrics evolved with the carer journey, with the song resonating and created an emotional response upon hearing the song.	Irons
Creative arts: creative interventions help for self-expression and emotional release	Participation in creative interventions allowed participants to have new experiences and to develop confidence to express oneself. For example, as one participant stated: "It probably unloads a lot of the...all the emotional feelings that you do hang on to that you don't even realise are there sometimes so it helps to get them out...it was good and it was cathartic. Even though I say I don't hold a lot you probably do have a bit of baggage and just talking about it and bringing out all those different words was, good and just very satisfying."	Irons
Creative arts: good for mindfulness	Three papers reported the recognition some caregivers had on the importance of mindfulness and living in the moment, and that these interventions created the space for this to be experienced.	Irons
Creative arts: singing decreases distress	Some papers reported that caregivers found singing allowed them to focus and concentrate on the task of singing and develop present moment awareness, allowing them to let go of other distressing or negative thoughts.	Irons
Creative arts: more clarity in thoughts and rational thinking	Themes related to increased lucidity, where caregivers reported clarity of thoughts and increased rational thinking.	Irons
Creative arts: singing benefits include better mood and well-being	All six studies included comments on improved mood or well-being and three on the extended impact in terms of mood.	Bourne
Creative arts: interventions do not reduce negative emotions, but increase positive ones	Interestingly this meta-theme did not mention the reduction of negative emotions and instead highlighted the increase in positive ones. This suggests the negative feelings were not removed by participation in creative caregiving interventions; however, caregivers and care-recipients experienced positive changes as a result of participation in these creative interventions.	Irons
Creative arts: art gallery is empowering	The structure, ambience and environment of an intervention was key to shaping and facilitating participants' experiences. For example, singing as part of a group was reported as an enabler for participation in interventions aimed at caregivers and care-recipients and an art gallery setting created an empowering space for participants	Irons
Creative arts: carers happy to see care receivers happy	The intervention activities were opportunities for playful experimentation, which was deemed the antithesis of caregiving, bringing about restorative feelings to caregivers and care-recipients. The caregivers experienced an increase in positive mood	Irons

For peer review only

				when they saw care-recipients expressing happiness due to participating in the intervention.	
			Mindfulness: intervention is enjoyable	Qualitatively, participants felt positive about MBSR and enjoyed participating.	Li
			Mindfulness: benefits include compassion and forgiveness	Qualitatively, participants reported the continued use of mindful based exercise and personal benefits of fostered compassion and forgiveness from the program.	Li
**Intervention outcomes:** This category describes potential beneficial and/or harmful effects to the caregiver from participating in the interventions	**Social outcomes:** intervention outcomes related to impact on caregiver's social life, day-to-day routine and relationship with care receiver and other people.	**Relational social outcomes:** changes in caregivers' relationship with either the care receiver or people around them.	Psychosocial: peer support leads to less isolation through mutual sharing	Qualitative social support outcomes demonstrated reduced feelings of social isolation, increased emotional support, and mutual sharing and enjoyment after receiving peer support.	Dam
			Respite: improved dyadic relationship	Carers reported increased engagement with their care recipients upon returning from adult day service attendance.	Du Preez
			Creative arts: dyadic facilitates reciprocity	As the group was for both partners of the dyad and reported to be accessible, this may have had a positive influence on relationships and communication, facilitating a sense of reciprocity.	Bourne
			Creative arts: singing brings more socializing	All six studies included comments on improved mood or well-being and three on the extended impact in terms of mood, the enjoyment of singing at home and socialising with other members outside of the group.	Bourne
			Creative arts: interventions give a feeling of equal dyadic relationships	Caregivers felt participation in the creative interventions allowed for interactions that were more equal and person-centred, as opposed to the usual caregiver–care-recipient dyad experiences that tended to occur outside the intervention.	Irons
			Creative arts: benefits of dyadic intervention: communication within dyads and others	As a result of participating in the intervention together, improved communication was reported by caregivers, not only within the dyads but also with other participants in the interventions and family members.	Irons
			Creative arts: interventions make dyads forget about the disease	Several papers described the opportunity for joint respite for the caregiver and care-recipient dyad to focus on the experience of the creative intervention, without needing to focus on the condition.	Irons
			Creative arts: dyad to dyad collaboration through singing	Furthermore, in a singing intervention, the social proximity of caregivers and their care-recipients to other caregivers and care-recipients were highly valued. The songs represented a shared experience, and this shared experience and collaboration was considered important.	Irons
			Creative arts: interventions make patients care recipients seem normal again	Participation in creative interventions allowed for caregivers to view the care-recipient in a different light, which led to increased satisfaction with caregiving role.	Irons

For peer review only - http://bmjopen.bmj.com/site/about/guidelines.xhtml
For peer review only

			Creative arts: better dyadic relationship	The process of creating a ‘time capsule’ database of music and photos from couples’ lives evoked strong positive feelings and led to improved quality time for caregivers and their care-recipients.	Irons
			Creative arts: improved image of care receivers	The demonstration of memory and recall over the weeks was encouraging for caregivers to see with care-recipients.	Irons
			Creative arts: better relationship based on a new common interest	Meaningful connections made with care-recipient through the creation of art allowed caregivers to build social connections that were based on mutual interests rather than the caregiving.	Irons
			Creative arts: dyadic better for spousal relationships	Additionally, papers reported other social benefits, including enhanced spousal relationship and strengthened reciprocity between caregiver and care-recipient.	Irons
**Implementation outcomes:** the effects of deliberate and purposive actions to implement the interventions	**Acceptability**: degree to which an intervention is perceived to be agreeable.	**Trust, dignity and values:** the role of trust, respect, ethics and dignity in determining acceptability of the intervention.	Case management: carers trust and appreciate admiral nurses they know well	There was evidence that carers valued interacting with a professional that they knew well, and descriptions of Admiral Nurses included ‘my anchor’, ‘life-saving’ ‘an angel’ and ‘worth her weight in gold’.	Bunn
			Counselling: carers want to have a reliable person by their side	Carers express their desire to know they have a reliable person by their side.	Lins
			Counselling: carers reluctant to unknown counsellors’ advice	A carer also expressed reservations about the idea of getting good advice from an unknown person since the disease has a great variety of manifestations.	Lins
			Counselling: carers sensitive to judgement	Carers will consciously or unconsciously notice the emotional attitude of the counsellor, which can lead them to trust the counselling less and experience it as unhelpful.	Lins
			Counselling: counsellors should be familiar with cases and caregivers	Carers can appreciate the counsellor as a person and know that the counsellor is familiar with their situation and can understand the situations the carer describes.	Lins
			Counselling: thorough counselling determines trust from caregiver	At the same time, the level of thoroughness with which counsellors convey the content of the discussion has an impact on how reliable and trustworthy the carer experiences the counsellor.	Lins
			Counselling: knowing the counsellors builds rapport and is more effective	The descriptive theme of 'Knowing each other' on the other hand, might have a positive impact on the counselling, partly because when counselling is not anonymous it helps counsellors to manage the frustrations of the carers, and partly because it lays the groundwork for becoming 'Familiar and trusted', which is the relationship desired by the carers.	Lins

For peer review only - http://bmjopen.bmj.com/site/about/guidelines.xhtml

Counselling: service should be non-judgmental and appreciative	Non-judgmental/appreciative. This descriptive theme describes how the attitude of the counsellor needs to be non-judgemental towards the 'non-professional' care provided by the carers and needs to convey appreciation for their great efforts.	Lins
Counselling: building rapport with caregivers is important	Carers and counsellors agree on the importance of getting to know each other before counselling.	Lins
Respite: dignity of care receiver more important than symptom management	Phinney and Moody argue that community-based services that endeavor to minimize stigma and normalize attendee's experiences, rather than focus on symptom management, may attract greater utilization.	Du Preez
Respite: respect and dignity for care receiver important	Additionally, carers expect their care recipients to be treated with care, respect, and dignity while attending an adult day service.	Du Preez
Respite: separation promotes stigmatization	Whilst separation may lead to stigmatization, group inclusion for attendees with cognitive decline appeared to promote their disengagement from activities requiring cognitive demand.	Du Preez
Respite: privacy violation is problematic	Factors such as concern for privacy violation and cost of service provision diminishes use of services by carers.	Du Preez
Respite: self-sufficiency, privacy and independence determine dyad's resistance to use the service	Values of self-sufficiency, privacy and independence that were displayed by carers were also held by the care recipients themselves, which contributed to resistance to service use.	Shaw
Respite: staff don't respect care receiver	Other negative attitudes towards respite included the view that it is demeaning for the care recipient, with its connotations of babysitting. This was not helped by experiences of a perceived lack of respect for the care recipient from formal care staff.	Shaw
Respite: service is demeaning for care receiver	Other negative attitudes towards respite included the view that it is demeaning for the care recipient, with its connotations of babysitting.	Shaw
Respite: trust is important to accept sitters	Indeed, this was mentioned by carers in several of the studies – the trust that is required of people coming into the home in this capacity.	Shaw
Creative arts: staff values and perspectives important for participants' experiences	The values and perspectives of the staff and those involved in the delivery of interventions were also pertinent to participants' experience of the interventions.	Irons
Creative arts: non-judgmental and active listening environment is important	A non-judgemental group approach was considered important to enable caregivers to voice their experiences with willing listeners, to share joy and sadness and to feel valued by others.	Irons

For peer review only - http://bmjopen.bmj.com/site/about/guidelines.xhtml

**Implementation outcomes:** the effects of deliberate and purposive actions to implement the interventions	**Acceptability**: degree to which an intervention is perceived to be agreeable.	**Person-centeredness:** personalization and flexibility of interventions promote acceptability of the intervention by the caregivers.	Case management: flexibility is valued	The importance of the relational aspect of the role including face-to face contact and opportunities to explain their needs and concerns in a time and manner of their preference were found to be integral to the person with dementia and carer's ability to adjust to change.	Goeman
			Case management: person-centered approach is welcome	A distinguishing characteristic of Admiral Nurses was their carer-centred approach and there was evidence that carers welcomed a service which focused on them.	Bunn
			Psychoeducation: flexibility in access is valued	Common elements of the interventions that were repeatedly highlighted and attributed to high satisfaction included: flexibility in access suiting carers' lifestyles and commitments.	Sin
			Psychoeducation: personalization of interventions is valued	Common elements of the interventions that were repeatedly highlighted and attributed to high satisfaction included: […] availability of self-tailored and -paced programme allowing for individualised information and support.	Sin
			Psychoeducation: individualized approach is preferred	The qualitative data supports the use of patient and carer information and support PCIS but suggests that there is not a one-size-fits-all approach which can be used, as every patient with dementia and their carers will have different needs, preferences and responses.	Miles
			Psychoeducation: personalized information highly valued	When information was individualized, it was considered by caregivers as one of the most useful functionalities of the intervention.	Hopwood
			Psychoeducation: carers don't appreciate generic information	[…] with caregivers expressing frustration when required to review information that did not meet their specific needs.	Hopwood
			Respite: carers' and care receivers' needs-based service is more acceptable	For respite to be acceptable it must respond to both the carer's and the care recipient's needs.	Shaw
			Respite: care receiver's happiness, health and safety cause concern	Beisecker et al. reports that 'perceptions about patient unhappiness, safety, physical health, functional levels and behaviour were viewed as barriers to ADC use'.	Shaw
			Respite: distress of care receivers causes concern	Care recipient distress was a commonly reported impact, which frequently took some time to recover from after return home.	Shaw
			Respite: quality of care of care receivers is essential	A barrier to the uptake of respite services was a concern about the quality of care provided and this concern was most notable in relation to nursing homes, although home care was also sometimes problematic.	Shaw

For peer review only - http://bmjopen.bmj.com/site/about/guidelines.xhtml

			Respite: care receiver's cooperation is important to participate	Gaining the care recipient's co-operation was one of the major hassles involved, which was reported particularly in relation to day care.	Shaw
			Creative arts: dyadic intervention allows for person-centered interaction	Caregivers felt participation in the creative interventions allowed for interactions that were more equal and person-centred, as opposed to the usual caregiver–care-recipient dyad experiences that tended to occur outside the intervention.	Irons
			Creative arts: flexibility is key	One key recommendation was to engage the assistance of a qualified music therapist, who is trained in adapting an intervention according to individual needs and preferences, including addressing behavioural challenge, thus affording flexibility in structure which was seen as key to caregiver satisfaction and continued participation in the intervention.	Irons
			Creative arts: intervention should be tailored to abilities	One caregiver stated their views about their perceptions of their partner with dementia who also participated in the intervention: ". . .she loves the looking. One of the things she mentioned a number of times is how important it is, the silence at the beginning, where they really get a chance to look. And I think that for people with. . . slow processing skills, not poor but slow, that element is just so important."	Irons
			Creative arts: intervention should be tailored to care receiver's abilities	In one study, participants were critical of the intervention protocol, as some caregivers found it difficult to engage the care-recipient or experienced frustration with the care-recipients' lack of focus, suggesting there were lessons to be learned for future enhancements of the intervention.	Irons
**Implementation outcomes:** the effects of deliberate and purposive actions to implement the interventions	**Acceptability**: degree to which an intervention is perceived to be agreeable.	**Culture and language:** ethnic, cultural and religious values influence acceptability of the intervention.	Case management: multicultural staff better at supporting diverse communities	A shared approach to care was found to be vital in decreasing burden among family members and that due to their close relationship and knowledge of families, multicultural workers can offer an important perspective that is invaluable in informing the provision of carer education and support within CALD communities.	Goeman
			Counselling: native language is preferred	Receiving counselling in the native language was also shown to contribute to building trust.	Lins
			Counselling: language can be a barrier in telephone counselling	Telephone counselling of carers who do not speak the national language is problematic.	Lins
			Respite: minorities prefer sitters of same ethnicity and gender	In this context this referred to ensuring that carers were of the same ethnic group, spoke the same language and were preferably of the same gender as the care recipient.	Shaw

For peer review only

For peer review only - http://bmjopen.bmj.com/site/about/guidelines.xhtml

			Respite: culture is important in determining demand for respite	Personal and cultural attitudes to the caring role and to the function and purpose of respite services may impact on the carers' perceptions of their needs for respite.	Shaw
			Respite: respect for culture and religious identity valued	Other important considerations for carers were that food was appropriate for their religion, for example vegetarian, and that the service was sensitive to other cultural and religious differences.	Shaw
**Implementation outcomes:** the effects of deliberate and purposive actions to implement the interventions	**Acceptability**: degree to which an intervention is perceived to be agreeable.	**Setting of the intervention:** environmental, social and structural components of the interventions that promote or hinder acceptability.	Psychoeducation: rapport with professionals and peers harder online	In a small number of studies, despite extensive recruitment efforts and provision of equipment and technical support, recruitment and completion rates still struggled as some carers reported finding it difficult to strike up a rapport with the professionals and their carer-peers and would still prefer the conventional delivery media using face to face group or individual meetings.	Sin
			Psychoeducation: support from carer networks and professionals is valued	Common elements of the interventions that were repeatedly highlighted and attributed to high satisfaction included: […] and network support through online forums with other carers and access to professionals.	Sin
			Psychoeducation: interaction among participants is important	Negative from one study: lack of interaction with other participants.	Boots
			Respite: in home care preferred over day care	Because of difficulties with day care many carers in the studies reported a preference for in-home care as being less disruptive for the care recipient.	Shaw
			Respite: turnover of staff is disruptive	One other major area of difficulty was related to the lack of continuity of care and the high turnover of staff in support services.	Shaw
			Respite: caregivers not informed about activities at respite	Family carers have little to no contact with the adult day service other than to ready their care recipient for the day's attendance and have little knowledge of how their care recipient spends their time while attending adult day service.	Du Preez
			Creative arts: carers appreciate having a special place	One paper found that caregivers in an intervention considered themselves ordinary users of a community place that was "somewhere different" and valued as a special place.	Irons
			Creative arts: group singing is better	The structure, ambience and environment of an intervention was key to shaping and facilitating participants' experiences. For example, singing as part of a group was reported as an enabler for participation in interventions aimed at caregivers and care-recipients.	Irons
			Creative arts: creative interventions offer interactive space	Papers presented themes around caregivers' and care-recipients' appreciation of a time and space afforded by the creative interventions to enable greater communication, and a natural	Irons

For peer review only - http://bmjopen.bmj.com/site/about/guidelines.xhtml

For peer review only

				development of social interactions both with the caregiver and other participants in the interventions.	
			Creative arts: gallery setting and facilitator important for art viewing	Two papers highlighted the importance of the gallery setting and facilitator characteristics.	Bourne
**Implementation outcomes:** the effects of deliberate and purposive actions to implement the interventions	**Acceptability**: degree to which an intervention is perceived to be agreeable.	**Other:** other determinants of acceptability, not groupable.	Psychoeducation: eHealth highly acceptable	In general, carers' perceived acceptability of the eHealth interventions across the studies synthesized was high.	Sin
			Psychoeducation: relatable experiences important	Experiential similarity is seen as important	Smith
			Creative arts: caregivers skeptical about their contributions to the interventions	Prior to joining the interventions, caregivers had deliberations around what to expect, including assumptions that they had nothing to contribute to the intervention.	Irons
			Mindfulness: mindfulness intervention is acceptable	Total of 31 FCs were included in the trial, overall, findings confirmed the feasibility and acceptability of the intervention for FCs, and the interviews, revealed that the intervention was associated with several participant-identified benefits and no severe adverse effects.	Aldaken
**Implementation outcomes:** the effects of deliberate and purposive actions to implement the interventions	**Feasibility:** the extent to which a new intervention can be successfully used or carried out within a given setting. Typically invoked retrospectively as a potential explanation of an intervention's success or failure, as reflected in poor recruitment, retention, or participation rates.	**Recruitment:** factors related to raising awareness about an intervention and recruiting suitable participants.	Case management: lack of clarity on service content	Carers' lack of clarity about the Admiral Nurse role was also observed; 'never really found out what the Admiral Nurse service was offering'.	Bunn
			Psychoeducation: recruitment issues in tech interventions	In a small number of studies, despite extensive recruitment efforts and provision of equipment and technical support, recruitment and completion rates still struggled.	Sin
			Respite: advise to use comes from primary care	The most accessible location for advice is the primary health-care centre.	Shaw
			Respite: lack of information on available services	Not only was there evidence of limited access to respite services but also there was a low awareness of the availability of services.	Shaw
			Respite: GPs not effective providing support despite being most accessible	The most accessible location for advice is the primary health-care centre, but it was suggested by more than one study that GPs do not appear to be providing appropriate support and information.	Shaw
			Respite: awareness on services low in minorities	Not only was there evidence of limited access to respite services but also there was a low awareness of the availability of services.	Shaw
			Respite: medical staff not informed about services	Medical practitioners were identified as having limited knowledge of community support services and access to information resulting in poor referral processes and therefore, poor utilization by family carers and people living with dementia.	Du Preez

For peer review only - http://bmjopen.bmj.com/site/about/guidelines.xhtml

**Implementation outcomes:** the effects of deliberate and purposive actions to implement the interventions	**Feasibility:** the extent to which a new intervention can be successfully used or carried out within a given setting. Typically invoked retrospectively as a potential explanation of an intervention's success or failure, as reflected in poor recruitment, retention, or participation rates.	**Accessibility:** physical, time-related or other external factors that limit or facilitate access to the interventions.	Case management: limiting admissions to confirmed dementia cases only	The failure factors were: […] and not including patients without a confirmed diagnosis of dementia.	Goeman
			Psychoeducation: access and time may be of concern for tech interventions	A few studies reported difficulties in even recruiting and retaining carers due to obstacles of access, cost, and time regarding use of technology.	Sin
			Counselling: telephone counselling is accessible	Carers find the telephone a good tool for receiving counselling as it helps avoid the stress involved in co-ordinating an appointment.	Lins
			Respite: transport may be an issue	There were a number of reports of complaints concerning transport, which included lack of transport in rural areas and a general unreliability of transport services.	Shaw
			Respite: waiting lists problematic	Waiting lists proved to be an issue for nursing home care and night care.	Shaw
			Respite: transport may be an issue	Transport was reported to be a significant barrier to utilization for carers. Use of public transport to access adult day service as opposed to transport being provided was found to be difficult and time-consuming.	Du Preez
			Creative arts: dyadic art interventions are accessible	As the group was for both partners of the dyad and reported to be accessible, this may have had a positive influence on relationships and communication, facilitating a sense of reciprocity.	Bourne
			Creative arts: singing is accessible for all	Caregivers highlighted other parts of the singing group experience to be important. These included the accessibility of singing for all.	Bourne
			Creative arts: art-viewing is accessible	Reported benefits included new learning, stimulation, and the accessibility of the activities, which creates a sense of normalcy and altered perceptions about PWD's abilities.	Bourne
**Implementation outcomes:** the effects of deliberate and purposive actions to implement the interventions	**Feasibility:** the extent to which a new intervention can be successfully used or carried out within a given setting. Typically invoked retrospectively as a potential explanation of an intervention's success or failure, as reflected in poor recruitment, retention, or participation rates.	**Service availability:** factors related to coverage of services in terms of offered times and availability of staff.	Case management: access easy, but contact difficult outside working hours	There was evidence that making and maintaining contact with the service initially was found to be easy in the majority of cases, but only just over half of the respondents knew who to contact outside Admiral Nurse's hours.	Bunn
			Counselling: 24/7 service is a must	Carers agreed that 24-hour availability of telephone counselling is necessary.	Lins
			Counselling: answering machines insufficient	Existing arrangements, such as answering machines for the times when counselling is not available, were not sufficient.	Lins
			Counselling: telephone counselling is flexible	It is a very comfortable means of counselling because carers do not have to leave their homes and its flexibility allows sessions to be fitted into their daily nursing routines.	Lins

For peer review only - http://bmjopen.bmj.com/site/about/guidelines.xhtml

			Respite: flexibility of day care is key	flexibility of the DC in relation to available days and times, and the possibility of a regular or intermittent frequency.	Maffioletti
			Respite: timing and flexibility can be problematic	A major barrier to the uptake of respite services was a lack of response to carer needs in terms of timing and flexibility of service provision.	Shaw
			Respite: working carers not accommodated schedule-wise	In another study day care was only offered for 3 days a week and finished at 3pm, which did not accommodate the needs of working carers.	Shaw
			Respite: weekends and evenings not covered	Problems with day care were the lack of weekend and evening provision.	Shaw
			Creative arts: different preferences on length of intervention	Some participants shared disappointment that the intervention was time-limited, whilst others said that it would be difficult to continue the sessions long-term due to care-giving responsibilities.	Bourne
			Mindfulness: carers appreciate informality and availability of service	Chronic conditions: Qualitative results indicated that the informal practice was particularly helpful and could be used at any time.	Li
			Mindfulness: dyadic mindfulness is more practical to attend	Also, it might be more feasible for caregivers because they would not have to leave care recipients alone.	Li
**Implementation outcomes:** the effects of deliberate and purposive actions to implement the interventions	**Feasibility:** the extent to which a new intervention can be successfully used or carried out within a given setting. Typically invoked retrospectively as a potential explanation of an intervention's success or failure, as reflected in poor recruitment, retention, or participation rates.	**Adoption and retention:** factors related to continued use of services or drop-out from services.	Psychoeducation: technical issues may arise	Usability problems (such as oral communication/chat quality, audio-visual function failure) were also identified as attributing to high drop-out rates (up to 50%) in some studies.	Sin
			Psychoeducation: older spousal carers find technology challenging	Most studies included focused on carers of dementia patients, and this was also the area with the most frequently reported problems in access and usability, as encountered by a group of largely elderly spousal carers who were often not familiar with ICT.	Sin
			Psychoeducation: dropout due to severe patients and carer time	Reasons for participant withdrawal included ill health of the person cared for and carer constraints.	Smith
			Psychoeducation: older adults find technology challenging	There appears to still be a gap between those who use or can use the internet and those who don't, with a study in 2015 highlighting that almost all adults over 70 years of age had difficulty using the intervention.	Hopwood
			Respite: carers need to prepare patients for respite	Carers reported having difficulty assisting their care recipient with their activities of daily living in preparation for the day's attendance.	Du Preez

For peer review only - http://bmjopen.bmj.com/site/about/guidelines.xhtml
For peer review only

			Respite: multicomponent interventions increase utilization	Adult day services that offer comprehensive services that engage dementia caregivers by way of phone calls or one-on-one carer meetings to address their areas of concern, invite carer collaboration in planning meetings, provide carer education, counseling and case management were seen to facilitate increased service utilization and delay early institutionalization.	Du Preez
			Respite: severe patients difficult to prepare and reluctant to participate	Carers reported having difficulty assisting their care recipient with their activities of daily living in preparation for the day's attendance. This was exacerbated by their care recipient's co-morbidities and resistance to attendance.	Du Preez
			Respite: decline in function leads to exclusion from service	Another barrier to use is attendance being conditional upon attendees being able to independently self-care.	Du Preez
			Respite: person centered activities increase utilization	Adult day services' ability or inability to offer a variety of activities that were person-centered and held meaning for attendees was a motivating factor for use.	Du Preez
			Respite: services minimizing stigma leads to greater utilization	Whilst separation may lead to stigmatization, group inclusion for attendees with cognitive decline appeared to promote their disengagement from activities requiring cognitive demand. However, Gaugler found staff overcame these issues through validation and one-to-one interaction.	Du Preez
			Respite: timing and flexibility may be barriers	A major barrier to the uptake of respite services was a lack of response to carer needs in terms of timing and flexibility of service provision.	Shaw
			Respite: severe PwD excluded	With confusion comes inappropriate behaviours, which were difficult for both in-home and institutional respite carers to accommodate, resulting in exclusion of the care recipient from the service.	Shaw
			Respite: service is used for maintaining nutrition	As the care recipient's condition deteriorates carers express concern for safety and the need for a sitter may be expressed. There are also concerns about maintaining care recipient health in relation to maintaining adequate nutrition.	Shaw
			Respite: progression of disease triggers use of service	As the care recipient's condition deteriorates carers express concern for safety and the need for a sitter may be expressed.	Shaw
**Implementation outcomes:** the effects of deliberate and purposive actions to implement the interventions	**Feasibility:** the extent to which a new intervention can be successfully used or carried out within a given setting. Typically invoked retrospectively as a potential	**Systemic factors:** Larger structural factors related to the organization of health and social care services.	Case management: primary care not involved is hindering	The failure factors were: […] little or no involvement of primary care specialists.	Goeman
			Case management: competition to deliver care is a barrier	The failure factors were: […] competition for delivering care.	Goeman

For peer review only - http://bmjopen.bmj.com/site/about/guidelines.xhtml

	explanation of an intervention's success or failure, as reflected in poor recruitment, retention, or participation rates.		Case management: medical staff not collaborating with admiral nurses	At times, carers felt that GPs, specialists and care managers did not work with or communicate with Admiral Nurses; 'Care manager doesn't seem to communicate with Admiral Nurse'.	Bunn
			Education/skills: carers appreciate having one source of information	Caregivers emphasised the importance of having one central source for information, for example a key worker to liaise with during visits.	Pritchard
			Respite: systems do not respond to need	Additionally, imperatives of service delivery systems place carers in a vulnerable position seemingly prioritizing these systems above the needs of caregivers. Utilization of services is often determined by effective referral by health professionals.	Du Preez
			Respite: bureaucracy unavoidable and intruding upon care receiver's privacy	Furthermore, interaction with adult day service providers requires carers to adopt institutional processes and intrudes upon their care recipient's privacy, albeit it to promote that person's quality of life.	Du Preez
**Implementation outcomes:** the effects of deliberate and purposive actions to implement the interventions	**Feasibility:** the extent to which a new intervention can be successfully used or carried out within a given setting. Typically invoked retrospectively as a potential explanation of an intervention's success or failure, as reflected in poor recruitment, retention, or participation rates.	**Other:** other determinants of feasibility, not groupable.	Mindfulness: mindfulness intervention is feasible	Total of 31 FCs were included in the trial, overall, findings confirmed the feasibility and acceptability of the intervention for FCs, and the interviews, revealed that the intervention was associated with several participant-identified benefits and no severe adverse effects.	Aldaken
**Implementation outcomes:** the effects of deliberate and purposive actions to implement the interventions	**Appropriateness:** perceived suitability and usefulness of intervention to address needs of caregivers.	**N.A.**	Case management: some needs are overlooked	Case management offered potential benefit to people with dementia, their carers and community-based professionals through continuity of care by a named trusted individual that could act proactively to prevent a crisis. However, it was also shown that needs may be overlooked.	Goeman
			Case management: multidisciplinary and continued services meet more needs	The specialist multi-agency home support service demonstrated greater flexibility and responsiveness to the particular needs and circumstances of service users and family carers, who were encouraged to participate in routine decision-making and activities. By sharing responsibilities, the specialist service helped reduce carer stress and prevent crises. These outcomes depended on the configuration of the service, including multidisciplinary health and social services input, care worker autonomy and independence, continuous reassessment of clients' circumstances and preferences and the capacity to develop long-term relationships, through care worker continuity. The standard service, which used a task orientated approach, lacked these characteristics.	Goeman

For peer review only - http://bmjopen.bmj.com/site/about/guidelines.xhtml

Case management: integration of care is necessary	It was found that the success for case management in long-term dementia care concern the expert knowledge of case managers; investment in a strong provider network and coherent conditions for effective inter-organisational cooperation to deliver integrated care.	Goeman
Case management: patient-centeredness is appropriate	It is suggested that the appropriate way of offering case management is through a patient-centred approach.	Goeman
Case management: admiral nurse as a liaison among care providers	A commonly reported component of the Admiral Nurse role was facilitation, which included liaison with other health and social care services; 'She makes sure she liaises with all the agencies concerned with mum's care'.	Bunn
Psychoeducation: Internet based support not for all	Qualitative evaluations of the interventions demonstrated positive views from most caregivers toward internet-based support interventions, although it is clear that not all would benefit from such interventions.	Hopwood
Psychoeducation: carers don't appreciate general information	[…] with caregivers expressing frustration when required to review information that did not meet their specific needs.	Hopwood
Psychoeducation: interaction with professionals more beneficial than just information	However, other multiple-component interventions evaluated with qualitative methods found that caregivers found other components, such as interaction with professionals, more beneficial than information.	Hopwood
Education/skills: Face-to-face and repeated information preferred	Appropriate modality and timing of information means the person may require information to be presented in different ways (e.g., in writing, diagrams) repeated on several occasions and in person, not over the phone.	Pritchard
Education/skills: differences between carers should be considered	The interaction between the personal characteristics of both the patient and caregiver is not always considered by health professionals. However, because the skills of the caregiver can impact the patient and vice versa, considering the interaction is paramount. Personal characteristics is also influenced by and influences organisational culture and the level of information provision.	Pritchard
Education/skills: carers' needs addressed related to information, coping mechanisms, assessment of home environment	The interventions addressed the need for clear information about the disease, identification of psychological response, development of coping mechanisms and assessment of the home environment.	Pritchard
Counselling: counsellors inform carers about other suitable services	Together with the carers, counsellors identify the most suitable services, which helps the carers to understand that they have access to an extensive help system in addition to telephone counselling.	Lins

For peer review only - http://bmjopen.bmj.com/site/about/guidelines.xhtml

			Counselling: more severe cases need more counselling	Counselling for carers is most needed, for example, in the case of an accident or during the final phase of the disease.	Lins
			Counselling: service needed to face potentially escalating crises	Carers need assistance most acutely in difficult situations when they feel insecure and see that an escalation is possible.	Lins
			Respite: respite seen as more appropriate for severe patients	Carers of recipients with dementia felt that day care, for example, was more appropriate for more severely demented people who were unaware of their surroundings.	Shaw
			Creative arts: group art not always beneficial	Two studies reported neutral or negative comments regarding participation in the group. These included some caregivers reporting initial apprehensiveness about attending, no effect on themselves but effects on the PWD.	Bourne
			Creative arts: carers cannot relax due to worry about PwD	These included some caregivers reporting initial apprehensiveness about attending, no effect on themselves but effects on the PWD, and difficulties with relaxation due to concerns about the PWD.	Bourne
			Creative arts: song writing addresses needs not met by other groups	The song writing group filled a gap for caregivers that were not met by other support groups.	Irons
			Creative arts: members of dyads don't have same needs	It may have been difficult to structure and time the delivery of an intervention to appeal to both caregiver and care-recipient, as illustrated by this quote: ''I think it drew him out more than it drew me out. And why that is, I don't know."	Irons
			Mindfulness: not all relaxing or benefitting	Although, these potential benefits were not universal, and some participants did report difficulty fully relaxing during the process.	Parkinson
**Implementation outcomes:** the effects of deliberate and purposive actions to implement the interventions	**Sustainability:** factors associated with sustained, long-term use of the intervention.	**N.A.**	Psychoeducation: shared experiences in peer support leads to sustainability	Carers were more likely to have successful peer support relationships and to continue meeting after the intervention ended, if they were similar on the shared experience of caring.	Smith
			Respite: experience of benefit and opportunistic use leads to regular use	The need for respite for particular social events or occasions such as conferences, weddings and holidays triggered the use of respite, and once the benefit was experienced and proved successful it was used again on a more regular basis.	Shaw
			Mindfulness: mindfulness interventions sustained by carers	Qualitatively, participants reported the continued use of mindful based exercise and personal benefits of fostered compassion and forgiveness from the program.	Li
**Implementation outcomes:** the effects of deliberate and purposive actions to implement the interventions	**Cost:** financial costs associated with implementing or using the intervention.	**N.A.**	Case management: funding is an issue	The failure factors were: […] inadequate or no structural funding.	Goeman

For peer review only

For peer review only - http://bmjopen.bmj.com/site/about/guidelines.xhtml

	Psychoeducation: cost is an issue	A few studies reported difficulties in even recruiting and retaining carers due to obstacles of access, cost, and time regarding use of technology.	Sin
	Respite: cost is an issue	Because of the different organizational processes involved in the different countries, cost issues may vary.	Shaw
	Respite: cost is an issue	Factors such as concern for privacy violation and cost of service provision diminishes use of services by carers. […] Affordability is an issue for some carers who constantly weigh their care recipients needs against the economic burden of meeting those need.	Du Preez

Abbreviations: QoL, Quality of Life; MBIs, Mindfulness-Based Interventions; LTCs, Long Term Conditions; PwD, Person with Dementia; MBSR, Mindfulness-Based Stress Reduction; PCIS, Patient and Carer Information and Support; CALD, Culturally And Linguistically Diverse; FC, Family Caregiver; DC, Day Care; ICT, Information and Communication Technology

For peer review only

GROOVE tool: Graphica

DOI: 10.17605/OSF.IO/U2MS4

What is GROOVE?

GROOVE (**G**raphical **R**epresentation of **O**verlap for **OVE**rviews) is a tool that provides a visual display

Starting with a simple matrix of evidence, this tool will automatically inform:
- The corrected covered area for the whole matrix.
- The number of unique primary studies.
- The number of overlapped primary studies (and in how many reviews they are included).
- A detailed assessment of the corrected covered area between every possible pair of reviews (nodes
- The number of nodes with low, moderate, high and very high overlap.
- Optionally, an assessment of the corrected covered area corrected by structural missingness in the

This tool is based on a previously published approach. If you are not familiar with the quantitative ass
Neugebauer EAM, Eikermann M. Systematic review finds overlapping reviews were not mentioned in

How to use this tool:

You only have to enter data on the "1. Matrix" sheet. The matrix supports up to 70 systematic review
You will get all the results on the "GROOVE" sheets (colour or greyscale version).

1. Go to the "1. Matrix" sheet.
2. Write all the systematic reviews ID in the columns, starting in the C5 cell.
3. Write all the primary studies ID in the rows, starting in the A6 cell.
4. Optionally, you can write the topic of your overview in the B2 cell, and the references of each prim
5. Mark with a "1" the intersections representing primary studies included in their respective system
6. Go to the "2.1 GROOVE - colour version" or "2.2 GROOVE - grayscale version" tab. You will find the
moderate, high and very high overlap, and the number of unique primary studies and overlapped pri

Advanced usage: Incorporating structural missingness

While you are building the matrix of evidence, you will notice that it should be impossible to mark a '
published in 2015 can not be included in a systematic review published in 2013; this would be a "chr

The GROOVE tool provides the option to incorporate structural missingness into the results. In order
1. Follow all the steps mentioned in the previous section.
2. When you finish populating the matrix of evidence with "1"s, you can mark the cells with structura
3. Once you finish, go to the "2.1 GROOVE - colour version" or "2.2 GROOVE - grayscale version" tab.
4. The Graphical Representation of Overlap for OVErviews incorporating structural missingness will b
structural missingness in the matrix, this figure will be the same as the one displayed in F1.

Example: How to enter data into the matrix

MATRIX OF EVIDENCE

TOPIC:

		Systematic	
**Primary Studies**			
**Study ID**	**Reference**	**Bonfill 2012**	**Urrútia 2014**
Bonham 1980		1	
Lennon 1980		1	1
Mercury 1991		1	
Harrison 2001			1
Jones 2007			1
McCartney 2010			1
Taylor 2011			
Page 2012			
Deacon 2013		X	
May 2013		X	1
Starr 2019		X	X
Plant 2020		X	X

2. Write all the primary studies ID here (references are optional)

Overall results

Number of columns (number of reviews)	c	4
Number of rows (number of index publications)	r	12
Number of included primary studies (including double counting)	N	20
Covered area	N/(rc)	41,67%
Corrected covered area	(N-r)/(rc-r)	22,22%
Interpretation of overlap	Very High overlap	
Structural Zeros	X	8
Corrected covered area (adjusting by structural zeros)	(N-r)/(rc-r-X)	28,57%

N° of non-overlapped primary studies	In 1 SR	7
Number of overlapped primary studies	In 2 SRs	2
	In 3 SRs	3
	In 4 SRs	0
	In 5 SRs	0
	In 6 SRs	0
	In 7 SRs	0
	In 8 SRs	0
	In 9 SRs	0
	In 10 SRs	0
	In 11 SRs	0
	In 12 SRs	0
	In 13 SRs	0
	In 14 SRs	0
	In 15 or more SRs	0

5. Go to the "2.1 GROOV grayscale version" sheet.

Graphical Representat

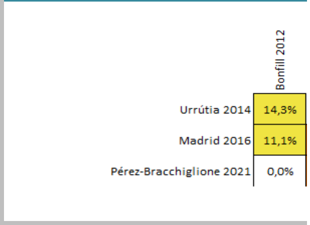

Graphical Represent

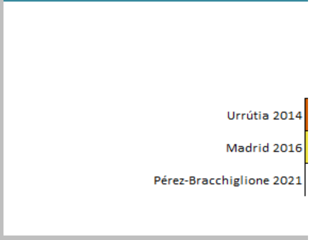

Cochrane Chile

Cochrane Iberoamérica

For peer review only

al Representation of Overl

of the overlap of primary studies among systematic reviev

s), and a graphical representation of this assessment.

matrix.

sessment of overlap using the corrected covered area form

every other overview. Journal of Clinical Epidemiology201

s and 1000 unique primary studies.

nary study in the B column, starting with B6.

atic reviews. It is expected for a single primary study to be

e corrected covered area (CCA) for the whole matrix, the C

imary studies.

"1" in some cells. A cell that can not acquire other value th

onological structural missingness".

to do this, you will have to:

al missingness with an "X". You will notice that the cell will

You will notice that the D9 cell will provide the CCA formu

pe displayed in the "2.1 GROOVE - colour version" and "2.2

iew only

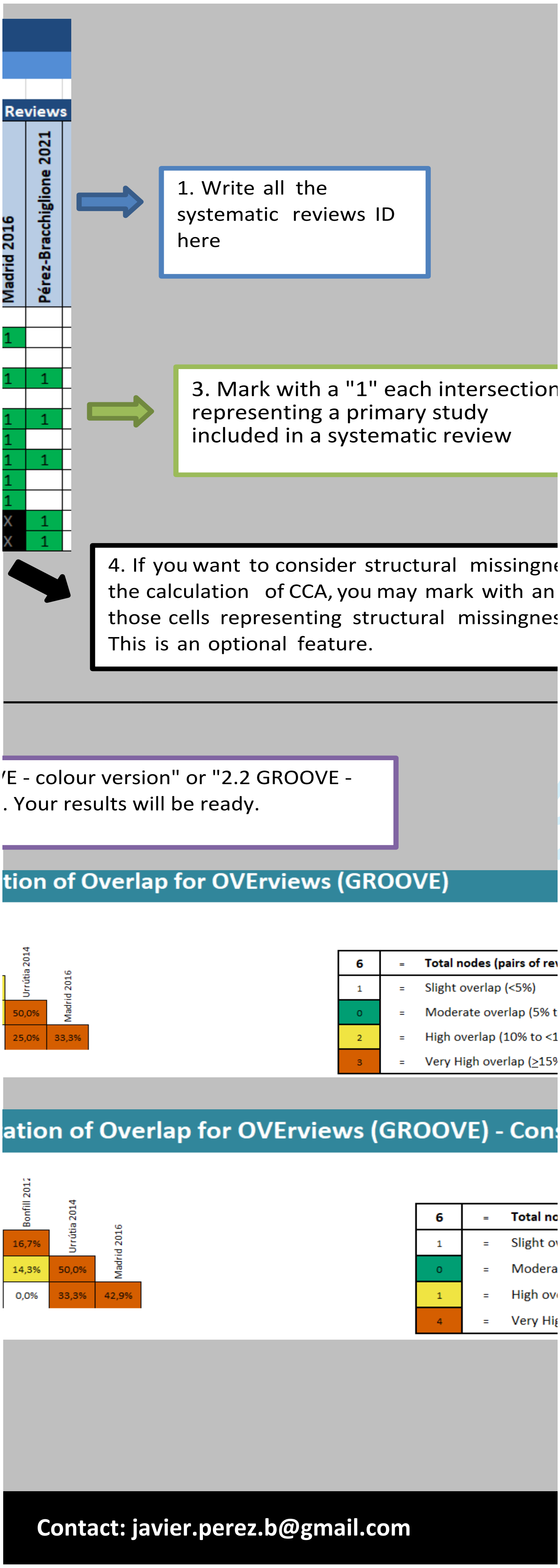
Reviews
Madrid 2016
Pérez-Bracchiglione 2021
1. Write all the systematic reviews ID here
3. Mark with a "1" each intersection representing a primary study included in a systematic review
4. If you want to consider structural missingne the calculation of CCA, you may mark with an those cells representing structural missingnes This is an optional feature.
'E - colour version" or "2.2 GROOVE - . Your results will be ready.
tion of Overlap for OVErviews (GROOVE)
Urrútia 2014
Madrid 2016
50,0%
25,0%
33,3%
6 = Total nodes (pairs of rev
1 = Slight overlap (<5%)
0 = Moderate overlap (5% t
2 = High overlap (10% to <1
3 = Very High overlap (≥15
ation of Overlap for OVErviews (GROOVE) - Cons
Bonfill 2012
Urrútia 2014
Madrid 2016
16,7%
14,3%
50,0%
0,0%
33,3%
42,9%
6 = Total nc
1 = Slight o
0 = Modera
1 = High ov
4 = Very Hig
Contact: javier.perez.b@gmail.com

iew only

Universidad de Valparaíso
CHILE

U[illegible]

For peer review only

ap for OVErviews

ws.

ula, we suggest you to read the original publication: Pieper D, Antoine S-L, Mathes T,
4;67(4):368–75.

e included in multiple systematic reviews.
CCA for each pair (or nodes) of systematic reviews, the number of nodes with low,

han 0 is called "structural missingness" or "structural zero". For example, a primary study

l turn black.
ula corrected by structural missingness.
2 GROOVE - grayscale version" tabs, starting in the cell CA1. If you do not mark any

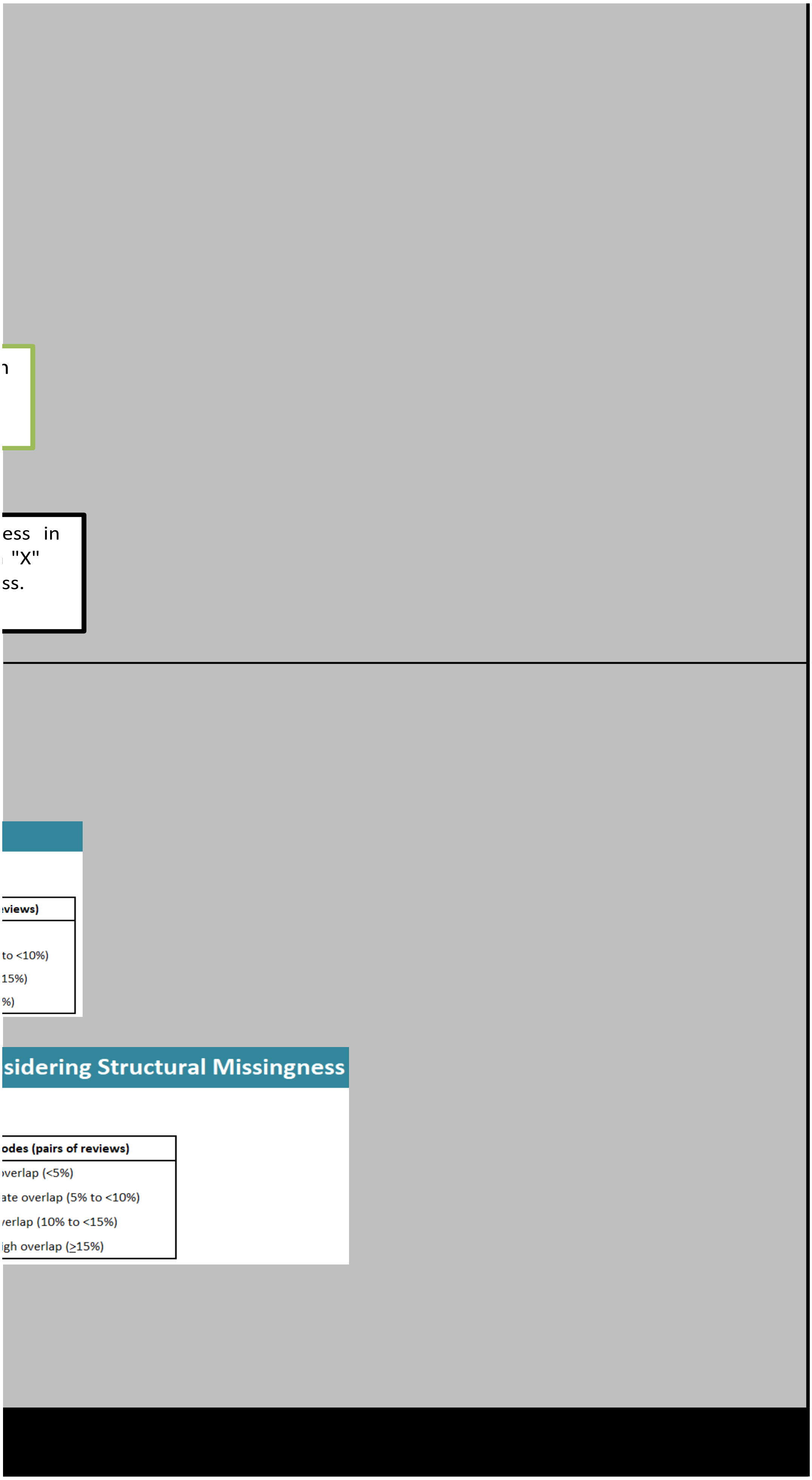
ess in
"X"
ss.
views)
to <10%)
15%)
%)
sidering Structural Missingness
odes (pairs of reviews)
verlap (<5%)
ate overlap (5% to <10%)
erlap (10% to <15%)
gh overlap (≥15%)

UAB
niversitat Autònoma
de Barcelona

CIESAL
CENTRO INTERDISCIPLINARIO
DE ESTUDIOS EN SALUD
UNIVERSIDAD DE VALPARAÍSO

For peer review only

	A	B	C	D	E	F	G	H	I	J
1	**MATRIX OF EVIDENCE**									
2	**TOPIC:**	**Informal caregiving UR**								
3										
4	**Primary Studies**		**Systematic Reviews**							
5	**Study ID**	**Reference (optional)**	**807 Irons 2020**	**1048 Gabriel 202**	**2195 Shim 2020**	**2367 Hopwood 2**	**2370 Guay 2017**	**2491 Vandepitte**	**2524 Zabihi 2020**	**3197 Mason 2007**
6	Camic2014		1							X
7	Bourne2019		1			X	X	X		X
8	Tamplin2018		1				X	X		X
9	Hanser2011		1							X
10	Davidson2014		1							X
11	Camic2011		1							X
12	Baker2018		1							X
13	Baker2012		1							X
14	Badger2013	Segrin, Hepworth		1						X
15	Badger2013	Segrin, Pasvogel		1						X
16	Badger2011			1						X
17	Belgacem2013			1						X
18	Clark2013			1						X
19	Kayser2010			1						X
20	Meyers2011			1						X
21	Northouse2014			1						X
22	Northouse2013			1						X
23	Shaw2016			1						X
24	Titler2017			1				X		X
25	Mosher2018			1			X	X		X
26	Brown2016				1			X		X
27	Oken2010				1					X
28	Black2013				1					X
29	Danucalov2017				1			X		X
30	Lavretsky2013				1					X
31	Leach2015				1					X
32	Waelde2017				1			X		X
33	Dowling2014				1					X
34	Davis2011									X
35	Ducharme2005									
36	Gaugler2015									X
37	McCallion1999									
38	Paun2015									X
39	Robison2007									X
40	Beauchamp2005					1	1			
41	Blom2015					1	1			X
42	Chih2013						1			X
43	Cristancho-Lacroix2015					1	1			X
44	DuBenske2014						1			X
45	Eames2013						1			X
46	Kim2013						1			X
47	McLaughlin2013						1			X

	A	B	C	D	E	F	G	H	I	J
48	Namkoong2012						1			X
49	Pierce2009						1			X
50	Smith2012						1			X
51	Torkamani2014						1			X
52	Lai2012									X
53	Hendrix2013									X
54	Porter2011									X
55	Sherwood2012									X
56	Boele2013									X
57	Fegg2013									X
58	Ledderer2013									X
59	McLean2013									X
60	Mitchell2013									X
61	Barrera2014									X
62	Badr2015									X
63	Dionne-Odom2015									X
64	Leow2015									X
65	Sun2015									X
66	Hendrix2016									X
67	Belle2006								1	
68	Burgio2003									
69	Chodosh2015									X
70	Czaja2013									X
71	Eisdorfer2003									
72	Gallagher-Thompson2003								1	
73	Gallagher-Thompson2007								1	X
74	Gallagher-Thompson2008									X
75	Gallagher-Thompson2015									X
76	Glueckauf2012									X
77	Gonyea2014									X
78	Winter2007							1		X
79	Au2014								1	X
80	Au2015								1	X
81	Coon2003								1	
82	Gallagher-Thompson2000								1	
83	Gonyea2006							1	1	
84	Losada2011								1	X
85	Moore2013								1	X
86	Read2016								1	X
87	Steffen2016								1	X
88	Vasquez2016								1	X
89	ArtasoIrigoyen2002									1
90	Baumgarten2002							1		1
91	Burch2001									1
92	Donaldson1989									1
93	Hedrick1993									1
94	Quayhagen2000									1
95	Zank2002							1		1
96	Zarit1998									1
97	Conlin1992									1
98	Kosloski1993									1
99	Montgomery1989									1
100	Lawton1989									1

For peer review only - http://bmjopen.bmj.com/site/about/guidelines.xhtml

	A	B	C	D	E	F	G	H	I	J
101	Grant2003							1		1
102	Johnson1988									1
103	Montgomery1985									1
104	Neibuhr1989									1
105	Wishart2000									1
106	Robertson2002									1
107	Holm2003									1
108	Wells1987									1
109	Clark2000									1
110	Lund1995									1
111	deRotrou2011							1		X
112	Gavrilova2009							1		X
113	Guerra2011									X
114	Hepburn2001							1		
115	Kurz2010							1		X
116	Martin-Carrasco 2009							1		X
117	Pahlavanzadeh2010									X
118	Newcomer1999									
119	Challis2002									
120	Leung2004									
121	Beland2006									
122	Vickrey2006							1		
123	Callahan2006									
124	ChienLee2008									X
125	Morales-Asencio2008									X
126	Wolff2009									X
127	Hebert2010									X
128	Lam2010									X
129	ChienLee2011									X
130	Jansen2011									X
131	Parsons2012									X
132	Janse2014									X
133	McNeilVroomen2015									X
134	Akkerman2004									
135	Arango-Lasprilla2014									X
136	Bourgeois2002									
137	EtxeberriaArritxabal2005									
138	EtxeberriaArritxabal2014									X
139	Gendron1996									
140	Gonyea2016									X
141	Gonzalez2014									X
142	Hebert2003									
143	Kamkhagi2015									X
144	Livingston2013							1		X
145	Livingston2014							1		X
146	Losada2005									
147	Losada2015									X
148	LosadaBaltar2004									
149	Marquez-Gonzalez2007									X
150	Passoni2014							1		X
151	WilzSoellner2016									X
152	Wilz2017									X
153	Forster2009									X

	A	B	C	D	E	F	G	H	I	J
154	Marsden2010									X
155	Perrin2010									X
156	Shyu2010									X
157	Galvin2011									X
158	Forster2013									X
159	Nayeri2014									X
160	Ostwald2014									X
161	Forster2015									X
162	Wang2015									X
163	Duncan2017									X
164	Aguirrezabal2013									X
165	Bishop2014									X
166	Fens2014									X
167	Chang1999									
168	Farran2007									X
169	Hepburn2005									
170	Marriott2000									
171	Zarit1987									
172	Bass2003									
173	Chu2000									
174	Dias2008									X
175	Eloniemi-Sulkava2001									
176	Eloniemi-Sulkava2009							1		X
177	Hinchliffe1995									
178	Hou2014									X
179	O'Donnel2013									X
180	Whitebird2013									X
181	Chang2004									
182	Davis2004									
183	Finkel2007									X
184	Gant2007									X
185	Glueckauf2007									X
186	Salfi2015									X
187	Steffen2000							1		
188	Tremont2008							1		X
189	Wilz2011									X
190	Draper2007									X
191	Grant2002									
192	Hartke2003									
193	Kalra2004									
194	Larson2005									
195	Mant2000									
196	Pierce2004									
197	Yoo2007									X
198	Banet1997									
199	Chinchai2010									X
200	Chiu2008					1				X
201	Downes1993									
202	Ellis2005									
203	Evans1988									
204	Frank2000									
205	Hoffmann2007									X
206	Johnson2000									

	A	B	C	D	E	F	G	H	I	J
207	Johnston2007									X
208	Lomer1987									
209	Lowe2007									X
210	Maasland2007									X
211	Mant1998									
212	O'Connel2009									X
213	Pain1990									
214	Rodgers1999									
215	Smith2004									
216	Connell2009							1		X
217	Corry2015									X
218	Kwok2013									X
219	Martindale-Adams2013									X
220	NCT00646217, 2008									X
221	Pfeiffer2014									X
222	Piamjariyakul2015									X
223	Powell2014									X
224	Shum2014									X
225	SmithToseland2006									
226	Toye2016									X
227	Wray2010									X
228	Kohle2017									X
229	Lloyd2018									X
230	Badger2007									X
231	Castro2002									
232	Danucalov2013									X
233	Hirano2011									X
234	King1997									
235	King2002									
236	Lowery2013									X
237	VanPuymbroeck2007									X
238	Campbell2007									X
239	Walsh2007									X
240	Carter2006									
241	McMilan2006									
242	Northouse2005									
243	Northouse2007									X
244	Dioquino2016									X
245	Drossel2011									X
246	Epstein-Lubow2011									X
247	Franco2010									X
248	Hoppes2012									X
249	Norouzi2014									X
250	Paller2015									X
251	Burgener2011									X
252	Milbury2015									X
253	Milbury2018									X
254	Winters-Stone2016									X
255	Barnes2015									X
256	Lamb2018									X
257	Maci2012									X
258	Marques2015									X
259	Yu2015									X

For peer review only - http://bmjopen.bmj.com/site/about/guidelines.xhtml

	A	B	C	D	E	F	G	H	I	J
260	Brennan1995					1				
261	Coulehan2011									X
262	Ducharme2011							1		X
263	Glueckauf2004					1				
264	Kelly2004									
265	Lai2013									X
266	Lewis2010					1				X
267	MarzialiGarcia2011					1				X
268	Torp2008									X
269	VanderRoest2010					1				X
270	Logsdon2016									X
271	Kim2012									X
272	Cho2009									X
273	Mossello2008							1		X
274	Droes2006									
275	Gitlin2006							1		
276	Droes2004									
277	Gaugler2003									
278	Zarit2011									X
279	Mavall2007									X
280	Higgins2005							1		
281	Menn2012							1		X
282	Mittelman2006							1		
283	Phung2013							1		X
284	Gitlin2003							1		
285	Andren2008	Scand J caring						1		X
286	Andren2008	Int Psychogeriatr						1		X
287	Drentea2006							1		
288	Berger2004							1		
289	Dahlrup2011							1		X
290	Charlesworth2008							1		X
291	Graff2006							1		
292	Femia2007							1		X
293	Woods2012							1		X
294	Ulstein2007							1		X
295	Ducharme2015							1		X
296	Teri2003							1		
297	Graff2007							1		X
298	Martin-Carrasco2015							1		X
299	RodriguezSanchez2013							1		X
300	Nobili2004							1		
301	Nichols2008							1		
302	Mahoney2003							1		
303	Liddle2012							1		X
304	Joling2012							1		X
305	Gitlin2008							1		X
306	Melis2009							1		X
307	Gitlin2001							1		
308	Mittelman2004	Zarit						1		
309	Mittelman2004	Haley						1		
310	Hepburn2007							1		X
311	Gitlin2010							1		X
312	Bass1998					1				

	A	B	C	D	E	F	G	H	I	J
313	Fowler2016					1				X
314	Hattink2015					1				X
315	Hicken2017					1	X	X		X
316	Kajiyama2013					1				X
317	NunezNaveira2016					1		X		X
318	VanMierlo2015					1				X
319	Davis2015					1				X
320	Griffiths2018					1	X	X		X
321	Kaijiyama2018					1	X	X		X
322	Kwok2014					1				X
323	Lorig2013					1				X
324	Austrom2015					1				X
325	Boots2017					1	X	X		X
326	Boots2016					1		X		X
327	Dam2017					1	X	X		X
328	Hattink2016					1		X		X
329	Jajor2016					1		X		X
330	MarzialiDonahue2006					1				
331	Mckechnie2014					1				X
332	O'Connor2014					1				X
333	Pagan-Ortiz2014					1				X
334	Pot2015					1				X
335	Rentz2010					1				X
336	Schaller2015					1				X
337	Schaller2016					1		X		X
338	Weitzman2016					1		X		X
339	Wilz2017						X	X		X
340	Brijoux2016							X		X
341	Wang2012									X
342	WangChien2011									X
343	Tanner2015									X
344	Orgeta2015									X
345	Schmitter2014									X
346	Korn2009									X
347	Berwig2017						X	X		X
348	Koivisto2016							X		X
349	Chu 2011									X
350	Bruvik2013									X
351	Fortinsky2009									X
352	Gallagher-Thompson2010									X
353	Garand2014									X
354	Judge2013									X
355	Kurz2012									X
356	Logsdon2011									X
357	Spijker2013									X
358	Teri2005									
359	Williams2010									X
360	Alexopoulos2012									X
361	Bakas2009									X
362	Bakas2015									X
363	Cheng2018						X	X		X
364	Clark2003									
365	Ertel2007									X

	A	B	C	D	E	F	G	H	I	J
366	Fang2017						X	X		X
367	Glass2004									
368	Harrington2010									X
369	Hoffmann2015									X
370	Inci2016									X
371	Kendall2007									
372	Markle-Reid2011									X
373	Mitchell2009									X
374	VandenHeuvel2000									
375	VandenHeuvel2002									
376	Watkins2007									X
377	Wong2015									X
378	Au2010									X
379	Barnes2018					X	X	X		X
380	Basu2015									X
381	Butcher2016							X		X
382	Canonici2012									X
383	Charlesworth2016							X		X
384	Chen2015									X
385	Cheng2014									X
386	Cheng2017							X		X
387	Cheng2016							X		X
388	Ducharme2012									X
389	Duggleby2018					X	X	X		X
390	Farran2016							X		X
391	Gitlin 2018					X		X		X
392	Foss2007									X
393	Heydari2017						X	X		X
394	Hirano2016							X		X
395	Horvath2013									X
396	Huang2013									X
397	Kales2018					X	X	X		X
398	KlodnickaKouri2011									X
399	Kunik2017						X	X		X
400	Kuo2016							X		X
401	Kuo2013									X
402	Kuo2017						X	X		X
403	Lee2012									X
404	Li2014									X
405	Lok2017						X	X		X
406	Lord2017						X	X		X
407	Mackenzie2007									X
408	Mahdavi2017						X	X		X
409	Moniz-Cook2008									X
410	Orrell2017						X	X		X
411	Perren2006									X
412	Pankong2018					X	X	X		X
413	Prick2015									X
414	Regan2017						X	X		X
415	Salamizadeh2017						X	X		X
416	Salva2011									X
417	Sarkamo2014									X
418	Schoenmakers2010									X

	A	B	C	D	E	F	G	H	I	J
419	Sepe-Monti2016							X		X
420	Shata2017						X	X		X
421	Spector2015									X
422	Stirling2012									X
423	Thyrian2017						X	X		X
424	Tremont2015									X
425	VanMierlo2012									X
426	Villareal-Reyna2012									X
427	Voigt-Radloff2011									X
428	Waldorff2012									X
429	Wesson2013									X
430	Woods2016							X		X
431	Xiao2016							X		X
432	Zwingmann2017						X	X		X
433	Cameron2015									X
434	Clarke2014									X
435	Franzen-Dahlin2008									X
436	Grasel2005									
437	Lindley2017						X	X		X
438	Pandian2015									X
439	Mant2005									
440	Martinez-Velilla2016							X		X
441	Oupra2010									X
442	Vandenberg2016							X		X
443	Shyu2008									X
444	Boltong2017						X	X		X
445	Boudioni1999									
446	Boudioni2004									
447	Bright2005									
448	Browman1995									
449	Byrne2012									X
450	Chambers2012									X
451	Darrow1998									
452	Davis1998									
453	Dean2007									
454	Ekberg2014									X
455	Fennell2016							X		X
456	Freimuth1993									
457	Hawkes2010									X
458	Heckel2016							X		X
459	Heidrich2003									
460	Hughes2011									X
461	Jefford2005									
462	Klikovac2015									X
463	Lechner1996									
464	Linehan2017						X	X		X
465	Maibach1998									
466	Manning2002									
467	Marcus1993									
468	Marcus2002									
469	Meissner1990									
470	Morris2015									X
471	Namboodiri1993									

	A	B	C	D	E	F	G	H	I	J
472	Reiches1982									
473	Reubsaet2006									
474	Rimer1993									
475	Finney2007									X
476	Salako2016							X		X
477	Slevin1988									
478	Sanders2008									X
479	Vanderpool2011									X
480	Venn1996									
481	Dewing1998									
482	Heckel2018						X	X		X
483	Sutherland2005									
484	Williams2019					X	X	X		X
485	Possin2019					X	X	X		X
486	Metcalfe2019					X	X	X		X
487	Meichsner2019					X	X	X		X
488	Gustafson2019					X	X	X		X
489	Elliott2010									X
490	Mittelman2007									X
491	Au2019					X	X	X		X
492	Hayden2012									X
493	HuisinhetVeld2020		X	X	X	X	X	X	X	X
494	Brodaty1997									
495	Burch1999									
496	Burdz1988									
497	Conlin1992									
498	Milne1993									
499	Richards2003									
500	Riordan1998									
501	Rolleston1994									
502	Rothman1993									
503	Schwarz1997									
504	Leitsch2001									
505	Adler1993									
506	Chi1994									
507	Cox1998									
508	Deimling1992									
509	Desroisers2004									
510	Gilleard1987									
511	Gilleard1984									
512	Harper1993									
513	Homer1994									
514	Hoskins2005									
515	Theis1994									
516	Tremont2013									X
517	Duncan2011									X
518	Candell2003									
519	Cernvall2015									X
520	Grover2011									X
521	Hoyle2013									X
522	Klemm2014									X
523	Petranovich2015									X
524	Rotonti2005									

	A	B	C	D	E	F	G	H	I	J
525	Swallow2015									X
526	Wade2012									X
527	Antonini2012									X
528	Barbabella2016							X		X
529	Berk2013									X
530	Binford2013									X
531	Blusi2014									X
532	Chiu2009									X
533	Clifford2013									X
534	Dew2004									
535	Fidika2015									X
536	Lorig2012									X
537	Lucas2011									X
538	Sander2009									X
539	Scott2013									X
540	Song2015									X
541	Zimmerman2014									X
542	Paller2015									X
543	Birnie2010									X
544	Cash2016						X			X
545	Hankin2010									X
546	Lengacher2012									X
547	Dooley2004									
548	Gerner2013									X
549	Clare2010									
550	O'connor2019					X	X	X		X
551	Winter2006									
552	Baucom2009									X
553	Blanchard1996									
554	Budin2008									X
555	Bultz2000									
556	Couper2015									X
557	Heinrich1985									
558	Kozachik2001									
559	Kuijer2004									
560	Kurtz2005									
561	Manne2004									
562	Manne2005									
563	Manne2011									X
564	McCorkle2007									X
565	Nicolaisen2018						X	X		X
566	Tsianakas2015									X
567	Bierlein2006									
568	Bird2007									X
569	Brodaty2009									X
570	Brown2012									X
571	Burns2003									
572	Cartmell2011									X
573	Chodosh2012									X
574	Clare2005									
575	Clark2004									
576	Duru2009									X
577	Edgecombe2009									X

	A	B	C	D	E	F	G	H	I	J
578	Fischer2003									
579	Gaugler2008									X
580	Iliffe2014									X
581	King2011									X
582	LaFontaine2011									X
583	Marek2006									
584	McAiney2010									X
585	Moore2011									X
586	Onder2008									X
587	Onder2007									X
588	Specht2009									X
589	Stevenson2006									
590	Tam-Tham2013									X
591	VandenBosch2009									X
592	Weaver2008									X
593	Woods2003									
594	Nichols2011									X
595	Fortinsky2014									X
596	Andren2008									X
597	Bartfay2013									X
598	Bass2013									X
599	Carbonneau2011									X
600	Corbett2012									X
601	Donofrio2015									X
602	Egan2010									X
603	Eggenberger2013									X
604	Elvish2013									X
605	Farran2008									X
606	Gaugler2011									X
607	Gitlin2010	Gerontechnology								X
608	Gitlin2010	JAMA								X
609	Kouri2011									X
610	Marim2013									X
611	Ponce2011									X
612	Rowe2010									X
613	Soylemez2016							X		X
614	Tang2018					X	X	X		X
615	Lowery2014									X
616	Gaugler2016							X		X
617	Geschke2012									X
618	Meichsner2018					X	X	X		X
619	Wilz2018					X	X	X		X
620										
621										
622										
623										
624										
625										
626										
627										
628										
629										
630										

	A	B	C	D	E	F	G	H	I	J
631										
632										
633										
634										
635										
636										
637										
638										
639										
640										
641										
642										
643										
644										
645										
646										
647										
648										
649										
650										
651										
652										
653										
654										
655										
656										
657										
658										
659										
660										
661										
662										
663										
664										
665										
666										
667										
668										
669										
670										
671										
672										
673										
674										
675										
676										
677										
678										
679										
680										
681										
682										
683										

iew only

	A	B	C	D	E	F	G	H	I	J
684										
685										
686										
687										
688										
689										
690										
691										
692										
693										
694										
695										
696										
697										
698										
699										
700										
701										
702										
703										
704										
705										
706										
707										
708										
709										
710										
711										
712										
713										
714										
715										
716										
717										
718										
719										
720										
721										
722										
723										
724										
725										
726										
727										
728										
729										
730										
731										
732										
733										
734										
735										
736										

ew only

	A	B	C	D	E	F	G	H	I	J
737										
738										
739										
740										
741										
742										
743										
744										
745										
746										
747										
748										
749										
750										
751										
752										
753										
754										
755										
756										
757										
758										
759										
760										
761										
762										
763										
764										
765										
766										
767										
768										
769										
770										
771										
772										
773										
774										
775										
776										
777										
778										
779										
780										
781										
782										
783										
784										
785										
786										
787										
788										
789										

ew only

	A	B	C	D	E	F	G	H	I	J
790										
791										
792										
793										
794										
795										
796										
797										
798										
799										
800										
801										
802										
803										
804										
805										
806										
807										
808										
809										
810										
811										
812										
813										
814										
815										
816										
817										
818										
819										
820										
821										
822										
823										
824										
825										
826										
827										
828										
829										
830										
831										
832										
833										
834										
835										
836										
837										
838										
839										
840										
841										
842										

ew only

	A	B	C	D	E	F	G	H	I	J
843										
844										
845										
846										
847										
848										
849										
850										
851										
852										
853										
854										
855										
856										
857										
858										
859										
860										
861										
862										
863										
864										
865										
866										
867										
868										
869										
870										
871										
872										
873										
874										
875										
876										
877										
878										
879										
880										
881										
882										
883										
884										
885										
886										
887										
888										
889										
890										
891										
892										
893										
894										
895										

For peer review only - http://bmjopen.bmj.com/site/about/guidelines.xhtml

	A	B	C	D	E	F	G	H	I	J
896										
897										
898										
899										
900										
901										
902										
903										
904										
905										
906										
907										
908										
909										
910										
911										
912										
913										
914										
915										
916										
917										
918										
919										
920										
921										
922										
923										
924										
925										
926										
927										
928										
929										
930										
931										
932										
933										
934										
935										
936										
937										
938										
939										
940										
941										
942										
943										
944										
945										
946										
947										
948										

iew only

	A	B	C	D	E	F	G	H	I	J
949										
950										
951										
952										
953										
954										
955										
956										
957										
958										
959										
960										
961										
962										
963										
964										
965										
966										
967										
968										
969										
970										
971										
972										
973										
974										
975										
976										
977										
978										
979										
980										
981										
982										
983										
984										
985										
986										
987										
988										
989										
990										
991										
992										
993										
994										
995										
996										
997										
998										
999										
1000										
1001										

	A	B	C	D	E	F	G	H	I	J
1002										
1003										
1004										
1005										
1008	[redacted]									

For peer review only

	K	L	M	N	O	P	Q	R	S	T	U	V	W	X	Y	Z	AA	AB	AC
1																			
2																			
3																			
4																			
5	5257 Wiegelman	3552 Akarsu 2019	3734 Fu 2017	3939 Jensen 2015	3965 Corvol 2017	3970 Hopkinson 2	4267 Pucciarelli 2	4415 Vernooij-Da	4452 Reilly 2015	4530 Liu 2018	4531 Lins 2014	4534 Legg 2011	4567 Forster 201	4588 Corry 2019	5731 Piersol 2017	2104 Cuthbert 20	3453 Collins 2019	615 Waldron 201	3544 Boots 2014
6								X				X	X						
7		X	X	X	X	X		X	X	X	X	X	X	X	X	X	X	X	X
8			X	X	X			X	X	X	X	X	X		X	X		X	X
9								X				X							
10								X				X	X						
11																			
12				X				X		X	X	X	X		X			X	X
13								X				X	X						
14			1					X				X	X						
15								X				X	X						
16																			
17			1					X				X	X						
18			1					X				X	X						
19																			
20			1					X				X							
21								X				X	X						
22			1					X				X	X						
23			1	X				X	X		X	X	X	1				X	X
24				X				X	X		X	X	X					X	X
25			X	X	X			X	X	X	X	X	X		X	X		X	X
26								X	X	1	X						1	X	X
27										1					1		1		
28								X				X	X					X	
29	1		X	X	X			X	X		X	X	X		X	X		X	X
30								X				X	X						
31				X				X	X		X	X	X					X	X
32			X	X	X			X	X		X	X	X		X	X		X	X
33								X			X	X	X					X	X
34								X				X		1	1				
35																			
36				X				X	X	X		X	X						
37																			
38				X				X	X	X		X	X						
39																			
40								1											1
41	1			X				X				X	X						
42								X				X	X						
43				X				X	X		X	X	X					X	X
44			1					X			X	X	X					X	X
45							1	X				X	X						
46								X				X	X						
47												X	X					X	

	K	L	M	N	O	P	Q	R	S	T	U	V	W	X	Y	Z	AA	AB	AC
48												X	X						
49																			
50							1	X				X	X						
51								X			X	X	X					X	X
52			1					X				X	X						
53			1					X				X	X						
54			1					X				X							
55			1					X				X	X						
56			1					X				X	X						
57			1					X				X	X						
58			1					X				X	X					X	
59			1					X				X	X					X	
60			1					X				X	X					X	
61			1					X			X	X	X					X	X
62			1	X				X	X		X	X	X					X	X
63			1	X				X	X		X	X	X					X	X
64			1	X				X	X		X	X	X					X	X
65			1	X				X	X		X	X	X					X	X
66			1	X				X	X		X	X	X					X	X
67		1																	
68		1																	
69		1		X				X	X		X	X	X					X	X
70	1	1						X				X	X					X	
71		1																	
72		1				1													
73		1				1		1						1					
74		1				1													
75		1		X				X	X		X	X	X					X	X
76		1						X				X	X	1	1				
77		1						X			X	X	X					X	X
78		1									1			1					
79								X			X							X	X
80	1			X				X	X		X	X	X					X	X
81						1		1											
82																			
83																			
84						1		X	X			X							
85	1							X				X	X		1			X	
86				X				X	X		X	X	X					X	X
87	1			X				X	X		X	X	X					X	X
88				X				X	X		X	X	X	1				X	X
89																			
90																			
91																			
92																			
93																			
94						1													
95																			
96																			
97																			
98																			
99																			
.00																			

For peer review only

	K	L	M	N	O	P	Q	R	S	T	U	V	W	X	Y	Z	AA	AB	AC
101																			
102																			
103																			
104																			
105																			
106																			
107																			
108																			
109																			
110																			
111	1			1															
112	1			1															
113	1			1															
114				1															
115	1			1															
116	1			1															
117				1															
118					1				1										
119					1														
120					1														
121					1														
122					1				1										
123					1				1										
124					1				1						1				
125					1														
126					1														
127					1														
128					1				1						1				
129					1			X	1			X			1				
130					1			X	1			X			1				
131					1			X				X	X						
132					1			X				X	X						
133					1			X			X	X	X					X	X
134						1		1											
135						1		X			X	X	X					X	X
136						1													
137						1													
138						1		X			X	X	X					X	X
139						1													
140				X		1		X	X		X	X	X					X	X
141						1		X			X	X	X					X	X
142						1		1											
143	1			X		1		X	X		X	X	X					X	X
144	1					1		X				X	X		1			X	
145	1					1		X			X	X	X					X	X
146						1													
147	1			X		1		X	X		X	X	X				1	X	X
148						1													
149						1		1											
150						1		X			X	X	X					X	X
151				X		1		X	X		X	X	X	1				X	X
152				X		1		X	X		X	X	X			X		X	X
153							1												

	K	L	M	N	O	P	Q	R	S	T	U	V	W	X	Y	Z	AA	AB	AC
154							1												
155							1												
156							1												
157							1	X				X							
158							1	X				X	X					X	
159							1	X			X	X	X					X	X
160							1	X			X	X	X					X	X
161				X			1	X	X		X	X	X					X	X
162				X			1	X	X		X	X	X					X	X
163				X			1	X	X		X	X	X			X		X	X
164							1	X				X	X					X	
165							1	X			X	X	X	1				X	X
166							1	X			X	X	X					X	X
167								1			1								
168								1											
169								1											
170								1											
171								1											
172									1										
173									1										
174									1										
175									1										
176									1										
177									1										
178								X		1	X	X	X					X	X
179								X		1		X	X				1	X	
180								X		1		X	X		1		1	X	
181											1								
182											1								
183											1								
184											1								
185											1								
186				X				X	X		1	X	X					X	
187											1								
188											1			1					
189								X			1	X							
190												1	1						
191												1							
192												1							
193												1	1						
194												1	1						
195												1							
196												1							
197												1							
198													1						
199													1						
200													1						1
201													1						
202													1						
203													1						
204													1						
205													1						
206													1						

For peer review only

	K	L	M	N	O	P	Q	R	S	T	U	V	W	X	Y	Z	AA	AB	AC
207													1						
208													1						
209													1						
210													1						
211													1						
212													1						
213													1						
214													1						
215													1						
216	1													1	1	1			
217				X				X				X	X	1				X	
218	1							X				X	X	1				X	
219								X				X	X	1				X	
220														1					
221								X				X	X	1				X	
222				X				X				X	X	1				X	
223								X				X	X	1				X	
224								X				X	X	1				X	
225														1					
226				X				X	X		X	X	X	1				X	X
227								X				X	X	1					
228				X				X			X	X	X			X		X	X
229				X				X			X	X	X			X		X	X
230																1			
231																1			
232								X				X				1		X	
233								X				X				1			
234																1			
235																1			
236								X				X				1		X	
237																1			
238																		1	
239																		1	
240																		1	
241																		1	
242																		1	
243																		1	
244				X				X	X		X	X	X				1	X	X
245								X				X					1		
246								X				X					1		
247																	1		
248								X				X	X		1		1		
249								X			X	X	X				1	X	X
250				X				X	X			X	X				1	X	
251								X											
252				X				X	X		X	X	X					X	X
253				X				X	X		X	X	X					X	X
254				X				X	X		X	X	X					X	X
255				X				X	X		X	X	X					X	X
256				X				X	X		X	X	X					X	X
257								X				X	X						
258				X				X	X		X	X	X					X	X
259				X				X	X		X	X	X					X	X

For peer review only

	K	L	M	N	O	P	Q	R	S	T	U	V	W	X	Y	Z	AA	AB	AC
260																			1
261								X				X							1
262								X				X			1				1
263																			1
264																			1
265								X				X	X					X	1
266																			1
267								X				X			1				1
268																			1
269																			1
270								X	X			X	X					X	
271								X				X	X						
272																			
273																			
274																			
275																			
276																			
277																			
278								X				X							
279																			
280																			
281								X				X							
282																			
283								X				X			1			X	
284																			
285															1				
286															1				
287																			
288																			
289								X				X							
290																			
291																			
292																			
293								X				X	X						
294																			
295				X				X	X		X	X	X					X	X
296																			
297																			
298	1			X				X	X		X	X	X					X	X
299								X				X	X					X	
300																			
301																			
302																			
303								X				X	X						
304	1							X				X	X		1				
305																			
306																			
307																			
308																			
309																			
310																			
311	1														1				
312																			

For peer review only

	K	L	M	N	O	P	Q	R	S	T	U	V	W	X	Y	Z	AA	AB	AC
313				X				X	X		X	X	X					X	X
314				X				X	X		X	X	X					X	X
315			X	X	X			X	X		X	X	X		X			X	X
316								X				X	X		1			X	
317				X				X	X		X	X	X					X	X
318				X				X	X		X	X	X					X	X
319				X				X	X		X	X	X					X	X
320			X	X	X			X	X	X	X	X	X		X			X	X
321			X	X	X			X	X	X	X	X	X		X			X	X
322								X				X	X					X	X
323								X				X	X					X	
324				X				X	X		X	X	X					X	X
325			X	X	X			X	X		X	X	X		X			X	X
326				X				X	X		X	X	X					X	X
327			X	X	X			X	X		X	X	X		X			X	X
328				X				X	X		X	X	X					X	X
329				X				X	X		X	X	X					X	X
330																			
331								X			X	X	X					X	X
332								X			X	X	X					X	X
333								X			X	X	X					X	X
334				X				X	X		X	X	X					X	X
335																			
336				X				X	X		X	X	X					X	X
337				X				X	X		X	X	X					X	X
338				X				X	X		X	X	X					X	X
339			X	X	X			X	X		X	X	X		X	X		X	X
340	1			X				X	X		X	X	X					X	X
341								X				X	X		1				
342								X				X							
343				X				X	X		X	X	X					X	X
344				X				X	X		X	X	X					X	X
345								X			X	X	X					X	X
346																			
347	1		X	X	X			X	X		X	X	X		X	X		X	X
348				X				X	X		X	X	X					X	X
349								X				X							
350								X				X	X					X	
351	1																		
352															1				
353								X			X	X	X					X	X
354	1							X				X	X					X	
355								X				X							
356								X				X							
357								X				X	X					X	
358																			
359																			
360								X				X	X						
361																			
362				X				X	X		X	X	X					X	X
363			X	X	X			X	X		X	X	X		X	X		X	X
364																			
365																			

	K	L	M	N	O	P	Q	R	S	T	U	V	W	X	Y	Z	AA	AB	AC
366			X	X	X			X	X		X	X	X		X	X		X	X
367																			
368																			
369				X				X	X		X	X	X					X	X
370				X				X	X		X	X	X					X	X
371																			
372								X				X							
373																			
374																			
375																			
376																			
377				X				X	X		X	X	X					X	X
378																			
379			X	X	X			X	X	X	X	X	X		X	X		X	X
380				X				X	X		X	X	X					X	X
381				X				X	X		X	X	X					X	X
382								X				X	X						
383	1			X				X	X		X	X	X					X	X
384	1			X				X	X		X	X	X					X	X
385								X			X	X	X					X	X
386	1		X	X	X			X	X		X	X	X		X	X		X	X
387	1			X				X	X		X	X	X					X	X
388								X				X	X						
389			X	X	X			X	X	X	X	X	X		X	X		X	X
390				X				X	X		X	X	X					X	X
391	1		X	X	X			X	X	X	X	X	X		X	X		X	X
392																			
393			X	X	X			X	X		X	X	X		X	X		X	X
394	1			X				X	X		X	X	X					X	X
395								X				X	X					X	
396								X				X	X					X	
397			X	X	X			X	X	X	X	X	X		X	X		X	X
398								X				X							
399	1		X	X	X			X	X		X	X	X		X	X		X	X
400				X				X	X		X	X	X					X	X
401								X				X	X		1			X	
402	1		X	X	X			X	X		X	X	X		X	X		X	X
403								X				X	X		1				
404								X			X	X	X					X	X
405			X	X	X			X	X		X	X	X		X	X		X	X
406			X	X	X			X	X		X	X	X		X	X		X	X
407																			
408	1		X	X	X			X	X		X	X	X		X	X		X	X
409																			
410			X	X	X			X	X		X	X	X		X	X		X	X
411																			
412			X	X	X			X	X	X	X	X	X		X	X		X	X
413	1			X				X	X		X	X	X					X	X
414			X	X	X			X	X		X	X	X		X	X		X	X
415			X	X	X			X	X		X	X	X		X	X		X	X
416								X				X							
417								X			X	X	X					X	X
418																			

For peer review only

	K	L	M	N	O	P	Q	R	S	T	U	V	W	X	Y	Z	AA	AB	AC
419				X				X	X		X	X	X					X	X
420			X	X	X			X	X		X	X	X		X	X		X	X
421				X				X	X		X	X	X					X	X
422								X				X	X						
423			X	X	X			X	X		X	X	X		X	X		X	X
424	1			X				X	X		X	X	X					X	X
425								X				X	X						
426								X				X	X						
427								X				X							
428								X				X	X						
429								X				X	X					X	
430	1			X				X	X		X	X	X					X	X
431				X				X	X		X	X	X					X	X
432			X	X	X			X	X		X	X	X		X	X		X	X
433				X				X	X		X	X	X					X	X
434								X			X	X	X					X	X
435																			
436																			
437			X	X	X			X	X		X	X	X		X	X		X	X
438				X				X	X		X	X	X					X	X
439																			
440				X				X	X		X	X	X					X	X
441																			
442				X				X	X		X	X	X					X	X
443																			
444			X	X	X			X	X		X	X	X		X	X		X	X
445																			
446																			
447																			
448																			
449								X				X	X						
450								X				X	X						
451																			
452																			
453																			
454								X			X	X	X					X	X
455				X				X	X		X	X	X					X	X
456																			
457																			
458				X				X	X		X	X	X					X	X
459																			
460								X				X							
461																			
462				X				X	X		X	X	X					X	X
463																			
464			X	X	X			X	X		X	X	X		X	X		X	X
465																			
466																			
467																			
468																			
469																			
470				X					X		X	X	X					X	X
471																			

For peer review only

	K	L	M	N	O	P	Q	R	S	T	U	V	W	X	Y	Z	AA	AB	AC
472																			
473																			
474																			
475																			
476				X					X		X	X	X					X	X
477																			
478																			
479								X				X							
480																			
481																			
482			X	X	X				X	X	X	X	X		X	X		X	X
483																			
484		X	X	X	X	X		X	X	X	X	X	X	X	X	X	X	X	X
485		X	X	X	X	X		X	X	X	X	X	X	X	X	X	X	X	X
486		X	X	X	X	X		X	X	X	X	X	X	X	X	X	X	X	X
487		X	X	X	X	X		X	X	X	X	X	X	X	X	X	X	X	X
488		X	X	X	X	X		X	X	X	X	X	X	X	X	X	X	X	X
489															1				
490																			
491		X	X	X	X	X		X	X	X	X	X	X	X	X	X	X	X	X
492								X				X	X						
493		X	X	X	X	X	X	X	X	X	X	X	X	X	X	X	X	X	X
494																			
495																			
496																			
497																			
498																			
499																			
500																			
501																			
502																			
503																			
504																			
505																			
506																			
507																			
508																			
509																			
510																			
511																			
512																			
513																			
514																			
515																			
516								X				X						X	
517								X				X							
518																			
519				X				X		X	X	X	X					X	X
520								X				X							
521								X				X	X					X	
522								X			X	X	X					X	X
523				X				X		X	X	X	X					X	X
524																			

For peer review only

	K	L	M	N	O	P	Q	R	S	T	U	V	W	X	Y	Z	AA	AB	AC
525				X				X		X	X	X	X					X	X
526								X				X	X						
527								X				X	X						
528				X				X	X		X	X	X					X	X
529								X				X	X					X	
530								X				X	X					X	
531								X			X	X	X					X	X
532																			
533								X				X	X					X	
534																			
535				X				X	X		X	X	X					X	X
536								X				X	X						
537								X				X							
538																			
539								X				X	X					X	
540				X				X	X		X	X	X					X	X
541								X			X	X	X					X	X
542				X				X	X		X	X	X					X	X
543																			
544				X				X	X		X	X	X					X	X
545																			
546								X				X	X						
547																			
548								X				X	X					X	
549																			
550		X	X	X	X	X		X	X	X	X	X	X	X	X	X	X	X	X
551																			
552																			
553																			
554																			
555																			
556				X				X	X		X	X	X					X	X
557																			
558																			
559																			
560																			
561																			
562																			
563								X				X							
564																			
565			X	X	X			X		X	X	X	X		X			X	X
566				X				X	X		X	X	X					X	X
567																			
568																			
569																			
570								X				X	X						
571																			
572								X				X							
573								X				X	X						
574																			
575																			
576																			
577																			

For peer review only

	K	L	M	N	O	P	Q	R	S	T	U	V	W	X	Y	Z	AA	AB	AC
578																			
579																			
580								X			X	X	X					X	X
581								X				X							
582								X				X							
583																			
584																			
585								X				X							
586																			
587																			
588																			
589																			
590								X				X	X					X	
591																			
592																			
593																			
594								X											
595								X				X	X					X	
596															1				
597								X				X	X		1			X	
598								X				X	X		1			X	
599								X				X			1				
600								X				X	X		1				
601				X				X	X			X	X		1			X	X
602															1				
603								X				X	X		1			X	
604								X				X	X		1			X	
605															1				
606								X				X			1				
607															1				
608	1														1				
609								X				X			1				
610								X				X	X		1			X	
611								X				X			1				
612															1				
613	1			X				X	X		X	X	X					X	X
614	1		X	X	X			X	X	X	X	X	X		X	X		X	X
615	1							X			X	X	X					X	X
616	1			X				X	X		X	X	X					X	X
617	1							X				X	X						
618	1		X	X	X			X	X	X	X	X	X		X	X		X	X
619	1		X	X	X			X	X	X	X	X	X		X	X		X	X
620																			
621																			
622																			
623																			
624																			
625																			
626																			
627																			
628																			
629																			
630																			

For peer review only

	K	L	M	N	O	P	Q	R	S	T	U	V	W	X	Y	Z	AA	AB	AC
631																			
632																			
633																			
634																			
635																			
636																			
637																			
638																			
639																			
640																			
641																			
642																			
643																			
644																			
645																			
646																			
647																			
648																			
649																			
650																			
651																			
652																			
653																			
654																			
655																			
656																			
657																			
658																			
659																			
660																			
661																			
662																			
663																			
664																			
665																			
666																			
667																			
668																			
669																			
670																			
671																			
672																			
673																			
674																			
675																			
676																			
677																			
678																			
679																			
680																			
681																			
682																			
683																			

For peer review only

	K	L	M	N	O	P	Q	R	S	T	U	V	W	X	Y	Z	AA	AB	AC
684																			
685																			
686																			
687																			
688																			
689																			
690																			
691																			
692																			
693																			
694																			
695																			
696																			
697																			
698																			
699																			
700																			
701																			
702																			
703																			
704																			
705																			
706																			
707																			
708																			
709																			
710																			
711																			
712																			
713																			
714																			
715																			
716																			
717																			
718																			
719																			
720																			
721																			
722																			
723																			
724																			
725																			
726																			
727																			
728																			
729																			
730																			
731																			
732																			
733																			
734																			
735																			
736																			

For peer review only

	K	L	M	N	O	P	Q	R	S	T	U	V	W	X	Y	Z	AA	AB	AC
737																			
738																			
739																			
740																			
741																			
742																			
743																			
744																			
745																			
746																			
747																			
748																			
749																			
750																			
751																			
752																			
753																			
754																			
755																			
756																			
757																			
758																			
759																			
760																			
761																			
762																			
763																			
764																			
765																			
766																			
767																			
768																			
769																			
770																			
771																			
772																			
773																			
774																			
775																			
776																			
777																			
778																			
779																			
780																			
781																			
782																			
783																			
784																			
785																			
786																			
787																			
788																			
789																			

For peer review only

	K	L	M	N	O	P	Q	R	S	T	U	V	W	X	Y	Z	AA	AB	AC
790																			
791																			
792																			
793																			
794																			
795																			
796																			
797																			
798																			
799																			
800																			
801																			
802																			
803																			
804																			
805																			
806																			
807																			
808																			
809																			
810																			
811																			
812																			
813																			
814																			
815																			
816																			
817																			
818																			
819																			
820																			
821																			
822																			
823																			
824																			
825																			
826																			
827																			
828																			
829																			
830																			
831																			
832																			
833																			
834																			
835																			
836																			
837																			
838																			
839																			
840																			
841																			
842																			

For peer review only

	K	L	M	N	O	P	Q	R	S	T	U	V	W	X	Y	Z	AA	AB	AC
843																			
844																			
845																			
846																			
847																			
848																			
849																			
850																			
851																			
852																			
853																			
854																			
855																			
856																			
857																			
858																			
859																			
860																			
861																			
862																			
863																			
864																			
865																			
866																			
867																			
868																			
869																			
870																			
871																			
872																			
873																			
874																			
875																			
876																			
877																			
878																			
879																			
880																			
881																			
882																			
883																			
884																			
885																			
886																			
887																			
888																			
889																			
890																			
891																			
892																			
893																			
894																			
895																			

For peer review only

	K	L	M	N	O	P	Q	R	S	T	U	V	W	X	Y	Z	AA	AB	AC
896																			
897																			
898																			
899																			
900																			
901																			
902																			
903																			
904																			
905																			
906																			
907																			
908																			
909																			
910																			
911																			
912																			
913																			
914																			
915																			
916																			
917																			
918																			
919																			
920																			
921																			
922																			
923																			
924																			
925																			
926																			
927																			
928																			
929																			
930																			
931																			
932																			
933																			
934																			
935																			
936																			
937																			
938																			
939																			
940																			
941																			
942																			
943																			
944																			
945																			
946																			
947																			
948																			

For peer review only

	K	L	M	N	O	P	Q	R	S	T	U	V	W	X	Y	Z	AA	AB	AC
949																			
950																			
951																			
952																			
953																			
954																			
955																			
956																			
957																			
958																			
959																			
960																			
961																			
962																			
963																			
964																			
965																			
966																			
967																			
968																			
969																			
970																			
971																			
972																			
973																			
974																			
975																			
976																			
977																			
978																			
979																			
980																			
981																			
982																			
983																			
984																			
985																			
986																			
987																			
988																			
989																			
990																			
991																			
992																			
993																			
994																			
995																			
996																			
997																			
998																			
999																			
1000																			
1001																			

For peer review only

	K	L	M	N	O	P	Q	R	S	T	U	V	W	X	Y	Z	AA	AB	AC
1002																			
1003																			
1004																			
1005																			
1008	[redacted]																		

For peer review only

	AD	AE	AF	AG	AH	AI	AJ	AK	AL	AM	AN	AO	AP	AQ	AR	AS	AT	AU	AV
1																			
2																			
3																			
4																			
5	**3984 Doyle 2020**	**4285 Maffioletti 2**	**2570 Lee 2020**	**2809 Weinbrecht**	**3014 Wang 2020**	**152 Minshall 201**	**588 Cheng 2020**	**1611 Smith 2019**	**248 Heckel 2019**	**1596 Egan 2018**	**1977 Zhao 2019**	**1992 Leng 2020**	**XT Maayan 2014**	**3195 Shaw 2009**	**2520 Teahan 202**	**4562 GonzalezFra**	**3528 Amador-Ma**	**5049 Sin 2018**	**4806 Parkinson 2**
6														X					
7				X		X		X	X	X	X		X	X			X	X	X
8				X						X			X	X			X	X	
9														X					
10														X					
11														X					
12										X			X	X				X	
13														X					
14														X					
15														X					
16														X					
17														X					
18														X					
19														X					
20														X					
21														X				1	
22														X					
23				X									X	X					
24				X									X	X					
25				X						X			X	X			X	X	
26				X			1						X	X					
27							1							X					
28														X					
29			1	X									X	X			X		
30							1							X					
31							1						X	X					
32				X			1						X	X			X		
33							1						X	X		1			
34														X					
35																			
36			1				1							X					
37																			
38														X					
39																			
40				1	1					1	1	1							
41					1		1			1	1	1		X		1		1	
42														X					
43					1		1			1	1	1	X	X		1		1	
44													X	X				1	
45								1						X					
46														X					
47														X				1	

For peer review only

	AD	AE	AF	AG	AH	AI	AJ	AK	AL	AM	AN	AO	AP	AQ	AR	AS	AT	AU	AV
48														X					
49								1						X				1	
50						1								X				1	
51			1	1			1				1	1	X	X		1		1	
52														X					
53														X					
54														X					
55														X					
56														X					
57														X					
58														X					
59														X					
60														X					
61													X	X					
62													X	X					
63													X	X					
64													X	X					
65													X	X					
66				X									X	X					
67				1			1												
68					1														
69							1						X	X					
70				1			1							X		1			
71																		1	
72																			
73							1												
74				1			1												
75							1						X	X					
76														X					
77													X	X					
78				1			1									1			
79													X	X					
80							1						X	X		1			
81																			
82																			
83							1												
84				1			1							X					
85							1							X	1				
86				X									X	X					
87				X	1		1						X	X					
88				X									X	X					
89																			
90														1					
91																			
92																			
93																			
94														1					
95		1												1					
96		1												1					
97																			
98														1					
99														1					
100													1	1					

For peer review only

	AD	AE	AF	AG	AH	AI	AJ	AK	AL	AM	AN	AO	AP	AQ	AR	AS	AT	AU	AV
101													1	1					
102														1					
103																			
104																			
105													1						
106																			
107																			
108														1					
109																			
110																			
111				1			1							X					
112			1				1							X	1				
113			1				1							X	1				
114																			
115				1			1							X			1		
116				1			1							X	1				
117							1							X					
118																			
119																			
120																			
121																			
122			1				1												
123				1			1												
124							1												
125																			
126														X					
127														X					
128				1										X					
129			1				1							X	1		1		
130			1	1			1							X					
131														X					
132														X					
133													X	X					
134																			
135													X	X					
136																			
137																			
138													X	X					
139																			
140				X			1						X	X					
141							1						X	X					
142																			
143							1						X	X					
144			1	1			1							X	1		1		
145							1						X	X			1		
146																			
147							1						X	X					
148																			
149							1												
150													X	X					
151				X									X	X					
152				X									X	X					
153														X					

	AD	AE	AF	AG	AH	AI	AJ	AK	AL	AM	AN	AO	AP	AQ	AR	AS	AT	AU	AV
154														X					
155								1						X					
156						1		1						X					
157								1						X					
158								1						X					
159													X	X					
160						1		1					X	X					
161						1							X	X					
162								1					X	X					
163				X									X	X					
164														X					
165						1		1					X	X					
166													X	X					
167																			
168																			
169																			
170																			
171																			
172																			
173																			
174				1			1								1				
175																			
176														X					
177																			
178													X	X					
179														X					
180							1							X	1				
181																			
182									1							1			
183							1											1	
184							1									1			
185							1												
186														X					
187					1														
188							1									1			
189							1							X					
190						1													
191						1		1											
192						1													
193								1											
194						1		1											
195								1											
196																			
197																			
198																			
199																			
200																			
201																			
202																			
203																			
204																			
205																			
206																			

For peer review only

	AD	AE	AF	AG	AH	AI	AJ	AK	AL	AM	AN	AO	AP	AQ	AR	AS	AT	AU	AV
207						1													
208																			
209																			
210																			
211																			
212																			
213																			
214						1													
215								1											
216							1												
217														X					
218		1					1							X	1	1			
219							1							X		1			
220																			
221														X					
222														X					
223														X					
224														X					
225																			
226				X				1					X	X					
227														X					
228													X	X					
229													X	X				X	
230	1																		
231																			
232							1							X					
233							1							X					
234																			
235																			
236	1													X					
237																			
238																			
239																			
240																			
241																			
242																			
243																			
244				X									X	X					
245														X					
246														X					
247														X					
248														X					
249													X	X					
250														X					
251	1													X					
252	1												X	X					
253	1			X									X	X				X	
254	1			X									X	X					
255	1												X	X					
256	1			X									X	X				X	
257	1													X					
258	1												X	X					
259	1												X	X					

For peer review only

	AD	AE	AF	AG	AH	AI	AJ	AK	AL	AM	AN	AO	AP	AQ	AR	AS	AT	AU	AV
260										1		1				1			
261														X					
262							1							X					
263																		1	
264																			
265										1				X				1	
266														X					
267														X				1	
268																			
269																			
270		1		X										X					
271		1												X					
272		1												X					
273		1																	
274		1																	
275		1																	
276		1																	
277		1												1					
278		1												X					
279		1																	
280		1												1					
281														X					
282																			
283			1	1										X			1		
284																			
285																			
286																			
287																			
288																			
289														X					
290			1	1			1												
291			1	1			1												
292																			
293				1			1							X					
294							1												
295													X	X					
296																			
297							1								1				
298			1	1			1						X	X	1		1		
299			1											X					
300																			
301																			
302																1		1	
303							1							X					
304			1	1			1							X	1				
305							1												
306														X					
307																			
308																			
309																			
310																			
311							1							X	1				
312																			

For peer review only

	AD	AE	AF	AG	AH	AI	AJ	AK	AL	AM	AN	AO	AP	AQ	AR	AS	AT	AU	AV
313				X									X	X				1	
314										1	1	1	X	X		1			
315				X								1	X	X			X		
316			1	1			1			1	1	1		X		1	1	1	
317				X			1					1	X	X		1		1	
318												1	X	X					
319													X	X					
320				X						X			X	X			X	X	
321				X						X			X	X			X	X	
322														X				1	
323														X					
324													X	X				1	
325				X									X	X			X		
326				X									X	X				1	
327				X									X	X			X		
328				X									X	X					
329				X									X	X					
330										1								1	
331													X	X				1	
332													X	X				1	
333													X	X				1	
334													X	X					
335														X					
336													X	X					
337				X									X	X				1	
338				X									X	X					
339			1	X									X	X			X		
340			1	X									X	X					
341			1				1							X			1		
342			1				1							X					
343			1				1						X	X			1		
344			1										X	X					
345			1										X	X					
346			1				1						1	X	1				
347			1	X			1						X	X			X		
348			1	X			1						X	X					
349				1			1							X					
350				1			1							X					
351				1			1							X					
352				1			1							X					
353				1			1						X	X					
354				1			1							X	1				
355				1			1							X					
356				1										X					
357				1										X					
358				1															
359					1									X					
360						1								X					
361						1								X					
362						1		1					X	X					
363				X		1							X	X			X	X	
364						1		1											
365						1													

	AD	AE	AF	AG	AH	AI	AJ	AK	AL	AM	AN	AO	AP	AQ	AR	AS	AT	AU	AV
366				X		1							X	X			X		
367						1													
368						1								X					
369						1							X	X					
370				X		1							X	X					
371						1													
372						1								X					
373						1								X					
374						1													
375						1													
376						1													
377						1							X	X					
378							1							X					
379				X			1			X			X	X			X	X	
380							1						X	X	1				
381				X			1						X	X					
382							1							X					
383				X			1						X	X					
384							1						X	X	1				
385							1						X	X					
386				X			1						X	X			X		
387				X			1						X	X					
388							1							X					
389				X			1			X		1	X	X		1	X	X	
390				X			1						X	X					
391				X			1			X			X	X			X	X	
392							1												
393				X			1						X	X			X		
394				X			1						X	X					
395							1							X					
396							1							X					
397				X			1			X		1	X	X			X	X	
398							1							X					
399				X			1						X	X			X		
400				X			1						X	X					
401							1							X	1				
402				X			1						X	X			X		
403							1							X					
404							1						X	X					
405				X			1						X	X			X		
406				X			1						X	X			X		
407							1												
408				X			1						X	X			X		
409							1												
410				X			1						X	X			X		
411							1												
412				X			1						X	X			X	X	
413							1						X	X	1				
414				X			1						X	X			X		
415				X			1						X	X			X		
416							1							X					
417							1						X	X					
418							1							X					

	AD	AE	AF	AG	AH	AI	AJ	AK	AL	AM	AN	AO	AP	AQ	AR	AS	AT	AU	AV
419				X			1						X	X					
420				X			1						X	X			X		
421							1						X	X					
422							1							X					
423				X			1						X	X			X		
424							1						X	X	1	1			
425							1							X					
426							1							X					
427							1							X					
428							1							X			1		
429							1							X					
430				X			1						X	X					
431				X			1						X	X					
432				X			1						X	X			X		
433								1					X	X					
434								1					X	X					
435								1											
436								1											
437				X				1					X	X			X		
438								1					X	X					
439								1											
440				X				1					X	X					
441								1						X					
442				X				1					X	X					
443								1											
444				X					1				X	X			X		
445									1										
446									1										
447									1										
448									1										
449									1					X					
450									1					X					
451									1										
452									1										
453									1										
454									1				X	X					
455				X					1				X	X					
456									1										
457									1					X					
458				X					1				X	X					
459									1										
460									1					X					
461									1										
462									1				X	X					
463									1										
464				X					1				X	X			X		
465									1										
466									1										
467									1										
468									1										
469									1										
470									1				X	X					
471									1										

For peer review only

	AD	AE	AF	AG	AH	AI	AJ	AK	AL	AM	AN	AO	AP	AQ	AR	AS	AT	AU	AV
472									1										
473									1										
474									1										
475									1										
476				X					1				X						
477									1										
478									1										
479									1					X					
480									1										
481									1										
482				X					1	X			X	X			X		
483									1										
484		X		X		X		X	X	X	X	1	X	X			X	X	X
485		X		X		X		X	X	X	X	1	X	X			X	X	X
486		X		X		X		X	X	X	X	1	X	X		1	X	X	X
487		X		X		X		X	X	X	X	1	X	X			X	X	X
488		X		X		X		X	X	X	X	1	X	X		1	X	X	X
489														X	1				
490															1				
491		X		X		X		X	X	X	X		X	X			X	X	X
492														X		1			
493	X	X	X	X	X	X	X	X	X	X	X	X	X	X	X	1	X	X	X
494														1					
495														1					
496														1					
497														1					
498														1					
499														1					
500														1					
501														1					
502														1					
503														1					
504														1					
505														1					
506														1					
507														1					
508														1					
509														1					
510														1					
511														1					
512														1					
513														1					
514														1					
515														1					
516														X			1		
517														X			1		
518																		1	
519													X	X				1	
520														X				1	
521														X				1	
522													X	X				1	
523													X	X				1	
524																		1	

	AD	AE	AF	AG	AH	AI	AJ	AK	AL	AM	AN	AO	AP	AQ	AR	AS	AT	AU	AV
525													X	X				1	
526														X				1	
527														X				1	
528				X									X	X				1	
529														X				1	
530														X				1	
531													X	X				1	
532														X				1	
533														X				1	
534																		1	
535													X	X				1	
536														X				1	
537														X				1	
538														X				1	
539														X				1	
540													X	X				1	
541													X	X				1	
542													X	X					1
543														X					1
544				X									X	X					1
545														X					1
546														X					1
547																			
548														X					
549														X					
550		X		X		X		X	X	X	X		X	X			X	X	X
551																			
552														X					
553																			
554																			
555																			
556													X	X					
557																			
558																			
559																			
560																			
561																			
562																			
563														X					
564																			
565										X			X	X			X	X	
566													X	X					
567																			
568																			
569														X					
570														X					
571																			
572														X					
573														X					
574																			
575																			
576														X					
577																			

For peer review only

	AD	AE	AF	AG	AH	AI	AJ	AK	AL	AM	AN	AO	AP	AQ	AR	AS	AT	AU	AV
578														X					
579																			
580													X	X					
581														X					
582														X					
583																			
584														X					
585														X					
586																			
587																			
588														X					
589																			
590														X					
591														X					
592																			
593																			
594														X					
595													X	X					
596																			
597														X					
598														X					
599														X					
600														X					
601													X	X					
602														X					
603														X					
604														X					
605																			
606														X					
607														X					
608														X					
609														X					
610														X					
611														X					
612														X					
613				X									X	X					
614				X						X			X	X			X	X	
615													X	X					
616				X									X	X					
617														X					
618				X						X			X	X			X	X	
619				X						X			X	X			X	X	
620																			
621																			
622																			
623																			
624																			
625																			
626																			
627																			
628																			
629																			
630																			

	AD	AE	AF	AG	AH	AI	AJ	AK	AL	AM	AN	AO	AP	AQ	AR	AS	AT	AU	AV
631																			
632																			
633																			
634																			
635																			
636																			
637																			
638																			
639																			
640																			
641																			
642																			
643																			
644																			
645																			
646																			
647																			
648																			
649																			
650																			
651																			
652																			
653																			
654																			
655																			
656																			
657																			
658																			
659																			
660																			
661																			
662																			
663																			
664																			
665																			
666																			
667																			
668																			
669																			
670																			
671																			
672																			
673																			
674																			
675																			
676																			
677																			
678																			
679																			
680																			
681																			
682																			
683																			

For peer review only

	AD	AE	AF	AG	AH	AI	AJ	AK	AL	AM	AN	AO	AP	AQ	AR	AS	AT	AU	AV
684																			
685																			
686																			
687																			
688																			
689																			
690																			
691																			
692																			
693																			
694																			
695																			
696																			
697																			
698																			
699																			
700																			
701																			
702																			
703																			
704																			
705																			
706																			
707																			
708																			
709																			
710																			
711																			
712																			
713																			
714																			
715																			
716																			
717																			
718																			
719																			
720																			
721																			
722																			
723																			
724																			
725																			
726																			
727																			
728																			
729																			
730																			
731																			
732																			
733																			
734																			
735																			
736																			

For peer review only

	AD	AE	AF	AG	AH	AI	AJ	AK	AL	AM	AN	AO	AP	AQ	AR	AS	AT	AU	AV
737																			
738																			
739																			
740																			
741																			
742																			
743																			
744																			
745																			
746																			
747																			
748																			
749																			
750																			
751																			
752																			
753																			
754																			
755																			
756																			
757																			
758																			
759																			
760																			
761																			
762																			
763																			
764																			
765																			
766																			
767																			
768																			
769																			
770																			
771																			
772																			
773																			
774																			
775																			
776																			
777																			
778																			
779																			
780																			
781																			
782																			
783																			
784																			
785																			
786																			
787																			
788																			
789																			

For peer review only

	AD	AE	AF	AG	AH	AI	AJ	AK	AL	AM	AN	AO	AP	AQ	AR	AS	AT	AU	AV
790																			
791																			
792																			
793																			
794																			
795																			
796																			
797																			
798																			
799																			
800																			
801																			
802																			
803																			
804																			
805																			
806																			
807																			
808																			
809																			
810																			
811																			
812																			
813																			
814																			
815																			
816																			
817																			
818																			
819																			
820																			
821																			
822																			
823																			
824																			
825																			
826																			
827																			
828																			
829																			
830																			
831																			
832																			
833																			
834																			
835																			
836																			
837																			
838																			
839																			
840																			
841																			
842																			

For peer review only

	AD	AE	AF	AG	AH	AI	AJ	AK	AL	AM	AN	AO	AP	AQ	AR	AS	AT	AU	AV
843																			
844																			
845																			
846																			
847																			
848																			
849																			
850																			
851																			
852																			
853																			
854																			
855																			
856																			
857																			
858																			
859																			
860																			
861																			
862																			
863																			
864																			
865																			
866																			
867																			
868																			
869																			
870																			
871																			
872																			
873																			
874																			
875																			
876																			
877																			
878																			
879																			
880																			
881																			
882																			
883																			
884																			
885																			
886																			
887																			
888																			
889																			
890																			
891																			
892																			
893																			
894																			
895																			

For peer review only

	AD	AE	AF	AG	AH	AI	AJ	AK	AL	AM	AN	AO	AP	AQ	AR	AS	AT	AU	AV
896																			
897																			
898																			
899																			
900																			
901																			
902																			
903																			
904																			
905																			
906																			
907																			
908																			
909																			
910																			
911																			
912																			
913																			
914																			
915																			
916																			
917																			
918																			
919																			
920																			
921																			
922																			
923																			
924																			
925																			
926																			
927																			
928																			
929																			
930																			
931																			
932																			
933																			
934																			
935																			
936																			
937																			
938																			
939																			
940																			
941																			
942																			
943																			
944																			
945																			
946																			
947																			
948																			

For peer review only

	AD	AE	AF	AG	AH	AI	AJ	AK	AL	AM	AN	AO	AP	AQ	AR	AS	AT	AU	AV
949																			
950																			
951																			
952																			
953																			
954																			
955																			
956																			
957																			
958																			
959																			
960																			
961																			
962																			
963																			
964																			
965																			
966																			
967																			
968																			
969																			
970																			
971																			
972																			
973																			
974																			
975																			
976																			
977																			
978																			
979																			
980																			
981																			
982																			
983																			
984																			
985																			
986																			
987																			
988																			
989																			
990																			
991																			
992																			
993																			
994																			
995																			
996																			
997																			
998																			
999																			
1000																			
1001																			

For peer review only

	AD	AE	AF	AG	AH	AI	AJ	AK	AL	AM	AN	AO	AP	AQ	AR	AS	AT	AU	AV
1002																			
1003																			
1004																			
1005																			
1008	[redacted]																		

For peer review only

	AW	AX	AY	AZ	BA	BB	BC	BD	BE	BF	BG	BH	BI	BJ	BK	BL	BM	BN	BO
1																			
2																			
3																			
4																			
5	5174 Bennett 201	5810 Lucero 2019	4421 Treanor 201	5244 Goeman 20	5375 Greenwood														
6																			
7	X	X	X	X	X														
8				X	X														
9																			
10																			
11																			
12				X	X														
13																			
14																			
15																			
16																			
17																			
18																			
19																			
20																			
21																			
22																			
23																			
24				X	X														
25				X	X														
26				X	X														
27																			
28																			
29				X	X														
30																			
31																			
32				X	X														
33																			
34																			
35																			
36																			
37																			
38																			
39																			
40																			
41																			
42																			
43																			
44																			
45																			
46																			
47																			

For peer review only

	AW	AX	AY	AZ	BA	BB	BC	BD	BE	BF	BG	BH	BI	BJ	BK	BL	BM	BN	BO
48																			
49																			
50																			
51																			
52																			
53																			
54																			
55																			
56																			
57																			
58			1																
59																			
60																			
61																			
62																			
63																			
64																			
65																			
66																			
67																			
68																			
69																			
70																			
71				1															
72																			
73																			
74																			
75																			
76																			
77																			
78																			
79																			
80																			
81																			
82																			
83																			
84																			
85																			
86																			
87																			
88																			
89																			
90																			
91																			
92																			
93																			
94																			
95																			
96																			
97																			
98																			
99																			
100																			

For peer review only

	AW	AX	AY	AZ	BA	BB	BC	BD	BE	BF	BG	BH	BI	BJ	BK	BL	BM	BN	BO
101																			
102																			
103																			
104																			
105																			
106																			
107																			
108																			
109																			
110																			
111																			
112																			
113																			
114																			
115																			
116																			
117																			
118																			
119																			
120																			
121				1															
122				1															
123				1															
124				1															
125				1															
126																			
127																			
128	1			1															
129				1															
130				1															
131																			
132																			
133																			
134																			
135																			
136																			
137																			
138																			
139																			
140																			
141																			
142				1															
143																			
144																			
145																			
146																			
147																			
148																			
149																			
150																			
151				X	X														
152				X	X														
153																			

For peer review only

	AW	AX	AY	AZ	BA	BB	BC	BD	BE	BF	BG	BH	BI	BJ	BK	BL	BM	BN	BO
154																			
155																			
156																			
157																			
158																			
159																			
160																			
161																			
162																			
163				X	X														
164																			
165																			
166																			
167		1																	
168																			
169																			
170																			
171																			
172		1		1															
173																			
174																			
175																			
176				1															
177																			
178																			
179																			
180																			
181																			
182																			
183																			
184		1																	
185																			
186																			
187																			
188		1																	
189																			
190																			
191																			
192																			
193																			
194																			
195																			
196																			
197																			
198																			
199																			
200																			
201																			
202																			
203																			
204																			
205																			
206																			

For peer review only

	AW	AX	AY	AZ	BA	BB	BC	BD	BE	BF	BG	BH	BI	BJ	BK	BL	BM	BN	BO
207																			
208																			
209																			
210																			
211																			
212																			
213																			
214																			
215																			
216		1																	
217																			
218																			
219		1																	
220																			
221																			
222																			
223																			
224																			
225																			
226																			
227				1															
228																			
229																			
230																			
231																			
232																			
233																			
234																			
235																			
236																			
237																			
238			1																
239																			
240																			
241																			
242																			
243			1																
244																			
245																			
246																			
247																			
248																			
249																			
250																			
251																			
252																			
253																			
254																			
255																			
256																			
257																			
258																			
259																			

For peer review only

	AW	AX	AY	AZ	BA	BB	BC	BD	BE	BF	BG	BH	BI	BJ	BK	BL	BM	BN	BO
260		1																	
261																			
262																			
263																			
264																			
265																			
266																			
267																			
268																			
269																			
270																			
271																			
272																			
273																			
274																			
275																			
276																			
277																			
278																			
279																			
280																			
281																			
282				1															
283																			
284	1																		
285																			
286																			
287																			
288																			
289																			
290																			
291	1																		
292																			
293																			
294																			
295																			
296				1															
297																			
298																			
299					1														
300				1															
301																			
302		1		1															
303																			
304																			
305	1																		
306																			
307	1																		
308				1															
309																			
310																			
311	1																		
312																			

For peer review only

	AW	AX	AY	AZ	BA	BB	BC	BD	BE	BF	BG	BH	BI	BJ	BK	BL	BM	BN	BO
313				X	X														
314																			
315				X	X														
316		1																	
317				X	X														
318																			
319																			
320				X	X														
321				X	X														
322																			
323																			
324																			
325				X	X														
326				X	X														
327				X	X														
328				X	X														
329				X	X														
330																			
331																			
332																			
333																			
334																			
335																			
336																			
337				X	X														
338				X	X														
339				X	X														
340				X	X														
341																			
342																			
343																			
344																			
345																			
346																			
347				X	X														
348				X	X														
349																			
350																			
351				1															
352		1																	
353																			
354																			
355																			
356																			
357																			
358																			
359																			
360																			
361																			
362																			
363				X	X														
364																			
365																			

For peer review only

	AW	AX	AY	AZ	BA	BB	BC	BD	BE	BF	BG	BH	BI	BJ	BK	BL	BM	BN	BO
366				X	X														
367																			
368																			
369																			
370																			
371																			
372																			
373																			
374																			
375																			
376																			
377																			
378																			
379				X	X														
380																			
381				X	X														
382																			
383				X	X														
384																			
385																			
386				X	X														
387				X	X														
388																			
389				X	X														
390				X	X														
391	1			X	X														
392																			
393				X	X														
394				X	X														
395																			
396																			
397				X	X														
398																			
399				X	X														
400				X	X														
401																			
402				X	X														
403																			
404																			
405				X	X														
406				X	X														
407																			
408				X	X														
409																			
410				X	X														
411																			
412				X	X														
413																			
414				X	X														
415				X	X														
416																			
417																			
418																			

For peer review only

	AW	AX	AY	AZ	BA	BB	BC	BD	BE	BF	BG	BH	BI	BJ	BK	BL	BM	BN	BO
419				X	X														
420				X	X														
421																			
422																			
423				X	X														
424		1																	
425																			
426																			
427	1																		
428																			
429																			
430				X	X														
431				X	X														
432				X	X														
433																			
434				1															
435																			
436																			
437				X	X														
438																			
439																			
440				X	X														
441																			
442				X	X														
443																			
444				X	X														
445																			
446																			
447																			
448																			
449																			
450																			
451																			
452																			
453																			
454																			
455				X	X														
456																			
457																			
458				X	X														
459																			
460																			
461																			
462																			
463																			
464				X	X														
465																			
466																			
467																			
468																			
469																			
470																			
471																			

For peer review only

	AW	AX	AY	AZ	BA	BB	BC	BD	BE	BF	BG	BH	BI	BJ	BK	BL	BM	BN	BO
472																			
473																			
474																			
475																			
476				X	X														
477																			
478																			
479																			
480																			
481																			
482				X	X														
483																			
484	X	X	X	X	X														
485	X	X	X	X	X														
486	X	X	X	X	X														
487	X	X	X	X	X														
488	X	X	X	X	X														
489																			
490																			
491	X	X	X	X	X														
492																			
493	X	X	X	X	X														
494																			
495																			
496																			
497																			
498																			
499																			
500																			
501																			
502																			
503																			
504																			
505																			
506																			
507																			
508																			
509																			
510																			
511																			
512																			
513																			
514																			
515																			
516																			
517																			
518																			
519																			
520																			
521																			
522																			
523																			
524																			

For peer review only

	AW	AX	AY	AZ	BA	BB	BC	BD	BE	BF	BG	BH	BI	BJ	BK	BL	BM	BN	BO
525																			
526																			
527																			
528				X	X														
529																			
530																			
531																			
532																			
533																			
534																			
535																			
536																			
537																			
538																			
539																			
540																			
541																			
542																			
543																			
544																			
545																			
546																			
547	1																		
548	1																		
549	1																		
550	1	X	X	X	X														
551		1																	
552			1																
553			1																
554			1																
555			1																
556			1																
557			1																
558			1																
559			1																
560			1																
561			1																
562			1																
563			1																
564			1																
565			1	X	X														
566			1																
567				1															
568				1															
569				1															
570				1															
571				1	1														
572				1															
573				1															
574				1															
575				1															
576				1															
577				1															

For peer review only

	AW	AX	AY	AZ	BA	BB	BC	BD	BE	BF	BG	BH	BI	BJ	BK	BL	BM	BN	BO
578				1															
579				1															
580				1															
581				1															
582				1															
583				1															
584				1															
585				1															
586				1															
587				1															
588				1															
589				1															
590				1															
591				1															
592				1															
593				1															
594					1														
595					1														
596																			
597																			
598																			
599																			
600																			
601																			
602																			
603																			
604																			
605																			
606																			
607																			
608																			
609																			
610																			
611																			
612																			
613				X	X														
614				X	X														
615																			
616				X	X														
617																			
618				X	X														
619				X	X														
620																			
621																			
622																			
623																			
624																			
625																			
626																			
627																			
628																			
629																			
630																			

For peer review only

	AW	AX	AY	AZ	BA	BB	BC	BD	BE	BF	BG	BH	BI	BJ	BK	BL	BM	BN	BO
631																			
632																			
633																			
634																			
635																			
636																			
637																			
638																			
639																			
640																			
641																			
642																			
643																			
644																			
645																			
646																			
647																			
648																			
649																			
650																			
651																			
652																			
653																			
654																			
655																			
656																			
657																			
658																			
659																			
660																			
661																			
662																			
663																			
664																			
665																			
666																			
667																			
668																			
669																			
670																			
671																			
672																			
673																			
674																			
675																			
676																			
677																			
678																			
679																			
680																			
681																			
682																			
683																			

For peer review only

	AW	AX	AY	AZ	BA	BB	BC	BD	BE	BF	BG	BH	BI	BJ	BK	BL	BM	BN	BO
684																			
685																			
686																			
687																			
688																			
689																			
690																			
691																			
692																			
693																			
694																			
695																			
696																			
697																			
698																			
699																			
700																			
701																			
702																			
703																			
704																			
705																			
706																			
707																			
708																			
709																			
710																			
711																			
712																			
713																			
714																			
715																			
716																			
717																			
718																			
719																			
720																			
721																			
722																			
723																			
724																			
725																			
726																			
727																			
728																			
729																			
730																			
731																			
732																			
733																			
734																			
735																			
736																			

For peer review only

	AW	AX	AY	AZ	BA	BB	BC	BD	BE	BF	BG	BH	BI	BJ	BK	BL	BM	BN	BO
737																			
738																			
739																			
740																			
741																			
742																			
743																			
744																			
745																			
746																			
747																			
748																			
749																			
750																			
751																			
752																			
753																			
754																			
755																			
756																			
757																			
758																			
759																			
760																			
761																			
762																			
763																			
764																			
765																			
766																			
767																			
768																			
769																			
770																			
771																			
772																			
773																			
774																			
775																			
776																			
777																			
778																			
779																			
780																			
781																			
782																			
783																			
784																			
785																			
786																			
787																			
788																			
789																			

For peer review only

	AW	AX	AY	AZ	BA	BB	BC	BD	BE	BF	BG	BH	BI	BJ	BK	BL	BM	BN	BO
790																			
791																			
792																			
793																			
794																			
795																			
796																			
797																			
798																			
799																			
800																			
801																			
802																			
803																			
804																			
805																			
806																			
807																			
808																			
809																			
810																			
811																			
812																			
813																			
814																			
815																			
816																			
817																			
818																			
819																			
820																			
821																			
822																			
823																			
824																			
825																			
826																			
827																			
828																			
829																			
830																			
831																			
832																			
833																			
834																			
835																			
836																			
837																			
838																			
839																			
840																			
841																			
842																			

For peer review only

	AW	AX	AY	AZ	BA	BB	BC	BD	BE	BF	BG	BH	BI	BJ	BK	BL	BM	BN	BO
843																			
844																			
845																			
846																			
847																			
848																			
849																			
850																			
851																			
852																			
853																			
854																			
855																			
856																			
857																			
858																			
859																			
860																			
861																			
862																			
863																			
864																			
865																			
866																			
867																			
868																			
869																			
870																			
871																			
872																			
873																			
874																			
875																			
876																			
877																			
878																			
879																			
880																			
881																			
882																			
883																			
884																			
885																			
886																			
887																			
888																			
889																			
890																			
891																			
892																			
893																			
894																			
895																			

For peer review only

	AW	AX	AY	AZ	BA	BB	BC	BD	BE	BF	BG	BH	BI	BJ	BK	BL	BM	BN	BO
896																			
897																			
898																			
899																			
900																			
901																			
902																			
903																			
904																			
905																			
906																			
907																			
908																			
909																			
910																			
911																			
912																			
913																			
914																			
915																			
916																			
917																			
918																			
919																			
920																			
921																			
922																			
923																			
924																			
925																			
926																			
927																			
928																			
929																			
930																			
931																			
932																			
933																			
934																			
935																			
936																			
937																			
938																			
939																			
940																			
941																			
942																			
943																			
944																			
945																			
946																			
947																			
948																			

For peer review only

	AW	AX	AY	AZ	BA	BB	BC	BD	BE	BF	BG	BH	BI	BJ	BK	BL	BM	BN	BO
949																			
950																			
951																			
952																			
953																			
954																			
955																			
956																			
957																			
958																			
959																			
960																			
961																			
962																			
963																			
964																			
965																			
966																			
967																			
968																			
969																			
970																			
971																			
972																			
973																			
974																			
975																			
976																			
977																			
978																			
979																			
980																			
981																			
982																			
983																			
984																			
985																			
986																			
987																			
988																			
989																			
990																			
991																			
992																			
993																			
994																			
995																			
996																			
997																			
998																			
999																			
1000																			
1001																			

For peer review only

	AW	AX	AY	AZ	BA	BB	BC	BD	BE	BF	BG	BH	BI	BJ	BK	BL	BM	BN	BO
1002																			
1003																			
1004																			
1005																			
1008	[redacted]																		

For peer review only

	BP	BQ	BR	BS	BT
1					
2					
3					
4					
5					
6					
7					
8					
9					
10					
11					
12					
13					
14					
15					
16					
17					
18					
19					
20					
21					
22					
23					
24					
25					
26					
27					
28					
29					
30					
31					
32					
33					
34					
35					
36					
37					
38					
39					
40					
41					
42					
43					
44					
45					
46					
47					

For peer review only

	BP	BQ	BR	BS	BT
48					
49					
50					
51					
52					
53					
54					
55					
56					
57					
58					
59					
60					
61					
62					
63					
64					
65					
66					
67					
68					
69					
70					
71					
72					
73					
74					
75					
76					
77					
78					
79					
80					
81					
82					
83					
84					
85					
86					
87					
88					
89					
90					
91					
92					
93					
94					
95					
96					
97					
98					
99					
.00					

For peer review only

	BP	BQ	BR	BS	BT
101					
102					
103					
104					
105					
106					
107					
108					
109					
110					
111					
112					
113					
114					
115					
116					
117					
118					
119					
120					
121					
122					
123					
124					
125					
126					
127					
128					
129					
130					
131					
132					
133					
134					
135					
136					
137					
138					
139					
140					
141					
142					
143					
144					
145					
146					
147					
148					
149					
150					
151					
152					
153					

For peer review only

	BP	BQ	BR	BS	BT
154					
155					
156					
157					
158					
159					
160					
161					
162					
163					
164					
165					
166					
167					
168					
169					
170					
171					
172					
173					
174					
175					
176					
177					
178					
179					
180					
181					
182					
183					
184					
185					
186					
187					
188					
189					
190					
191					
192					
193					
194					
195					
196					
197					
198					
199					
200					
201					
202					
203					
204					
205					
206					

For peer review only

	BP	BQ	BR	BS	BT
207					
208					
209					
210					
211					
212					
213					
214					
215					
216					
217					
218					
219					
220					
221					
222					
223					
224					
225					
226					
227					
228					
229					
230					
231					
232					
233					
234					
235					
236					
237					
238					
239					
240					
241					
242					
243					
244					
245					
246					
247					
248					
249					
250					
251					
252					
253					
254					
255					
256					
257					
258					
259					

For peer review only

	BP	BQ	BR	BS	BT
260					
261					
262					
263					
264					
265					
266					
267					
268					
269					
270					
271					
272					
273					
274					
275					
276					
277					
278					
279					
280					
281					
282					
283					
284					
285					
286					
287					
288					
289					
290					
291					
292					
293					
294					
295					
296					
297					
298					
299					
300					
301					
302					
303					
304					
305					
306					
307					
308					
309					
310					
311					
312					

For peer review only

	BP	BQ	BR	BS	BT
313					
314					
315					
316					
317					
318					
319					
320					
321					
322					
323					
324					
325					
326					
327					
328					
329					
330					
331					
332					
333					
334					
335					
336					
337					
338					
339					
340					
341					
342					
343					
344					
345					
346					
347					
348					
349					
350					
351					
352					
353					
354					
355					
356					
357					
358					
359					
360					
361					
362					
363					
364					
365					

For peer review only

	BP	BQ	BR	BS	BT
366					
367					
368					
369					
370					
371					
372					
373					
374					
375					
376					
377					
378					
379					
380					
381					
382					
383					
384					
385					
386					
387					
388					
389					
390					
391					
392					
393					
394					
395					
396					
397					
398					
399					
400					
401					
402					
403					
404					
405					
406					
407					
408					
409					
410					
411					
412					
413					
414					
415					
416					
417					
418					

For peer review only

	BP	BQ	BR	BS	BT
419					
420					
421					
422					
423					
424					
425					
426					
427					
428					
429					
430					
431					
432					
433					
434					
435					
436					
437					
438					
439					
440					
441					
442					
443					
444					
445					
446					
447					
448					
449					
450					
451					
452					
453					
454					
455					
456					
457					
458					
459					
460					
461					
462					
463					
464					
465					
466					
467					
468					
469					
470					
471					

For peer review only

	BP	BQ	BR	BS	BT
472					
473					
474					
475					
476					
477					
478					
479					
480					
481					
482					
483					
484					
485					
486					
487					
488					
489					
490					
491					
492					
493					
494					
495					
496					
497					
498					
499					
500					
501					
502					
503					
504					
505					
506					
507					
508					
509					
510					
511					
512					
513					
514					
515					
516					
517					
518					
519					
520					
521					
522					
523					
524					

For peer review only

	BP	BQ	BR	BS	BT
525					
526					
527					
528					
529					
530					
531					
532					
533					
534					
535					
536					
537					
538					
539					
540					
541					
542					
543					
544					
545					
546					
547					
548					
549					
550					
551					
552					
553					
554					
555					
556					
557					
558					
559					
560					
561					
562					
563					
564					
565					
566					
567					
568					
569					
570					
571					
572					
573					
574					
575					
576					
577					

For peer review only

	BP	BQ	BR	BS	BT
578					
579					
580					
581					
582					
583					
584					
585					
586					
587					
588					
589					
590					
591					
592					
593					
594					
595					
596					
597					
598					
599					
600					
601					
602					
603					
604					
605					
606					
607					
608					
609					
610					
611					
612					
613					
614					
615					
616					
617					
618					
619					
620					
621					
622					
623					
624					
625					
626					
627					
628					
629					
630					

For peer review only

	BP	BQ	BR	BS	BT
631					
632					
633					
634					
635					
636					
637					
638					
639					
640					
641					
642					
643					
644					
645					
646					
647					
648					
649					
650					
651					
652					
653					
654					
655					
656					
657					
658					
659					
660					
661					
662					
663					
664					
665					
666					
667					
668					
669					
670					
671					
672					
673					
674					
675					
676					
677					
678					
679					
680					
681					
682					
683					

For peer review only

	BP	BQ	BR	BS	BT
684					
685					
686					
687					
688					
689					
690					
691					
692					
693					
694					
695					
696					
697					
698					
699					
700					
701					
702					
703					
704					
705					
706					
707					
708					
709					
710					
711					
712					
713					
714					
715					
716					
717					
718					
719					
720					
721					
722					
723					
724					
725					
726					
727					
728					
729					
730					
731					
732					
733					
734					
735					
736					

For peer review only

	BP	BQ	BR	BS	BT
737					
738					
739					
740					
741					
742					
743					
744					
745					
746					
747					
748					
749					
750					
751					
752					
753					
754					
755					
756					
757					
758					
759					
760					
761					
762					
763					
764					
765					
766					
767					
768					
769					
770					
771					
772					
773					
774					
775					
776					
777					
778					
779					
780					
781					
782					
783					
784					
785					
786					
787					
788					
789					

For peer review only

	BP	BQ	BR	BS	BT
790					
791					
792					
793					
794					
795					
796					
797					
798					
799					
800					
801					
802					
803					
804					
805					
806					
807					
808					
809					
810					
811					
812					
813					
814					
815					
816					
817					
818					
819					
820					
821					
822					
823					
824					
825					
826					
827					
828					
829					
830					
831					
832					
833					
834					
835					
836					
837					
838					
839					
840					
841					
842					

For peer review only

	BP	BQ	BR	BS	BT
843					
844					
845					
846					
847					
848					
849					
850					
851					
852					
853					
854					
855					
856					
857					
858					
859					
860					
861					
862					
863					
864					
865					
866					
867					
868					
869					
870					
871					
872					
873					
874					
875					
876					
877					
878					
879					
880					
881					
882					
883					
884					
885					
886					
887					
888					
889					
890					
891					
892					
893					
894					
895					

For peer review only

	BP	BQ	BR	BS	BT
896					
897					
898					
899					
900					
901					
902					
903					
904					
905					
906					
907					
908					
909					
910					
911					
912					
913					
914					
915					
916					
917					
918					
919					
920					
921					
922					
923					
924					
925					
926					
927					
928					
929					
930					
931					
932					
933					
934					
935					
936					
937					
938					
939					
940					
941					
942					
943					
944					
945					
946					
947					
948					

For peer review only

	BP	BQ	BR	BS	BT
949					
950					
951					
952					
953					
954					
955					
956					
957					
958					
959					
960					
961					
962					
963					
964					
965					
966					
967					
968					
969					
970					
971					
972					
973					
974					
975					
976					
977					
978					
979					
980					
981					
982					
983					
984					
985					
986					
987					
988					
989					
990					
991					
992					
993					
994					
995					
996					
997					
998					
999					
1000					
1001					

For peer review only

	BP	BQ	BR	BS	BT
1002					
1003					
1004					
1005					
1008	[redacted]				

For peer review only

Overall results		
Number of columns (number of reviews)	c	51
Number of rows (number of index publications)	r	614
Number of included primary studies (including double counting)	N	1071
Covered area	N/(rc)	3.42%
Corrected covered area	(N-r)/(rc-r)	1.49%
Interpretation of overlap	Slight overlap	
Structural Zeros	X	3598
Corrected covered area (adjusting by structural zeros)	(N-r)/(rc-r-X)	1.69%
N° of non-overlapped primary studies	In 1 SR	395
Number of overlapped primary studies	In 2 SRs	107
	In 3 SRs	45
	In 4 SRs	23
	In 5 SRs	15
	In 6 SRs	10
	In 7 SRs	5
	In 8 SRs	2
	In 9 SRs	3
	In 10 SRs	1
	In 11 SRs	0
	In 12 SRs	1
	In 13 SRs	0
	In 14 SRs	0
	In 15 or more SRs	0

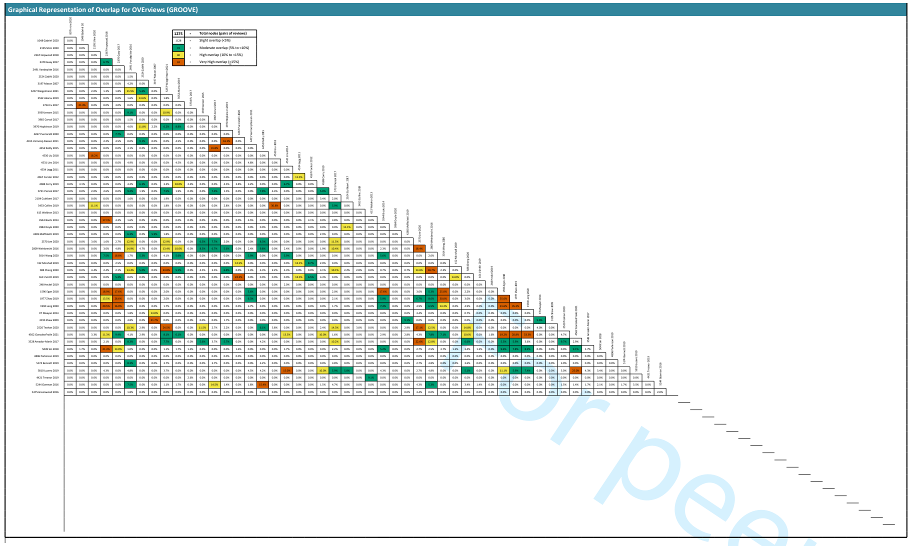

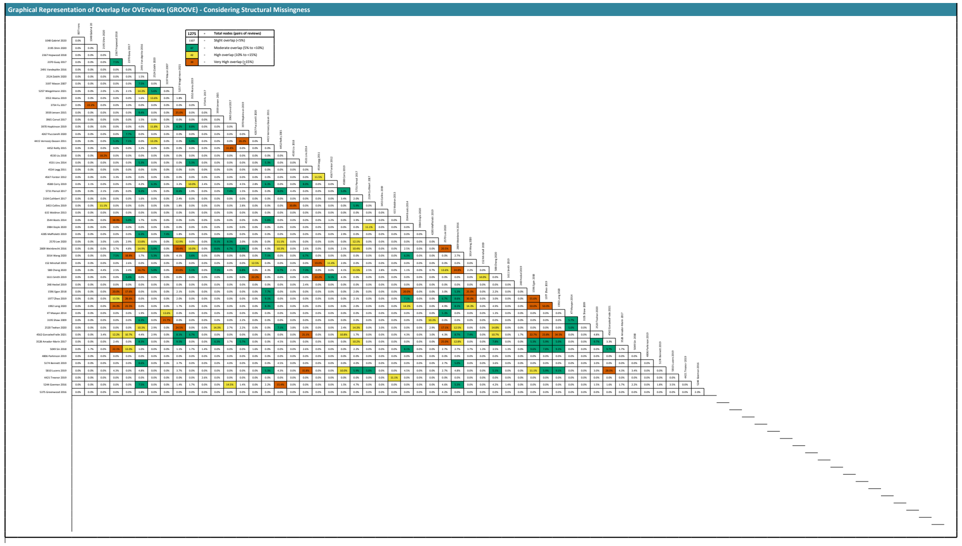

For peer review only - http://bmjopen.bmj.com/site/about/guidelines.xhtml

Overall results

Number of columns (number of reviews)	c	51
Number of rows (number of index publications)	r	614
Number of included primary studies (including double counting)	N	1071
Covered area	N/(rc)	3.42%
Corrected covered area	(N-r)/(rc-r)	1.49%
Interpretation of overlap	**Slight overlap**	
Structural Zeros	X	3598
Corrected covered area (adjusting by structural zeros)	(N-r)/(rc-r-X)	1.69%

N° of non-overlapped primary studies	In 1 SR	395
Number of overlapped primary studies	In 2 SRs	107
	In 3 SRs	45
	In 4 SRs	23
	In 5 SRs	15
	In 6 SRs	10
	In 7 SRs	5
	In 8 SRs	2
	In 9 SRs	3
	In 10 SRs	1
	In 11 SRs	0
	In 12 SRs	1
	In 13 SRs	0
	In 14 SRs	0
	In 15 or more SRs	0

G

For peer review only

raphical Representation of Overlap for OVErviews (GRO

	807 Irons 2020	1048 Gabriel 202	2195 Shim 2020	2367 Hopwood 2018	2370 Guay 2017	2491 Vandepitte 2016	2524 Zabihi 2020	3197 Mason 2007	5257 Wiegelmann 2021	3552 Akarsu 2019
1048 Gabriel 2020	0.0%									
2195 Shim 2020	0.0%	0.0%								
2367 Hopwood 2018	0.0%	0.0%	0.0%							
2370 Guay 2017	0.0%	0.0%	0.0%	6.7%						
2491 Vandepitte 2016	0.0%	0.0%	0.0%	0.0%	0.0%					
2524 Zabihi 2020	0.0%	0.0%	0.0%	0.0%	0.0%	1.5%				
3197 Mason 2007	0.0%	0.0%	0.0%	0.0%	0.0%	4.2%	0.0%			
5257 Wiegelmann 2021	0.0%	0.0%	2.0%	1.3%	1.8%	11.5%	5.6%	0.0%		
3552 Akarsu 2019	0.0%	0.0%	0.0%	0.0%	0.0%	1.6%	13.6%	0.0%	1.8%	
3734 Fu 2017	0.0%	21.4%	0.0%	0.0%	3.0%	0.0%	0.0%	0.0%	0.0%	0.0%
3939 Jensen 2015	0.0%	0.0%	0.0%	0.0%	0.0%	9.1%	0.0%	0.0%	10.9%	0.0%
3965 Corvol 2017	0.0%	0.0%	0.0%	0.0%	0.0%	1.5%	0.0%	0.0%	0.0%	0.0%
3970 Hopkinson 2019	0.0%	0.0%	0.0%	0.0%	0.0%	4.0%	11.8%	2.2%	6.2%	8.8%
4267 Pucciarelli 2020	0.0%	0.0%	0.0%	0.0%	7.7%	0.0%	0.0%	0.0%	0.0%	0.0%
4415 Vernooij-Dassen 2011	0.0%	0.0%	0.0%	2.2%	4.5%	0.0%	9.1%	0.0%	0.0%	4.5%
4452 Reilly 2015	0.0%	0.0%	0.0%	0.0%	0.0%	3.1%	0.0%	0.0%	0.0%	0.0%
4530 Liu 2018	0.0%	0.0%	18.2%	0.0%	0.0%	0.0%	0.0%	0.0%	0.0%	0.0%
4531 Lins 2014	0.0%	0.0%	0.0%	0.0%	0.0%	4.9%	0.0%	0.0%	0.0%	4.5%
4534 Legg 2011	0.0%	0.0%	0.0%	0.0%	0.0%	0.0%	0.0%	0.0%	0.0%	0.0%
4567 Forster 2012	0.0%	0.0%	0.0%	1.8%	0.0%	0.0%	0.0%	0.0%	0.0%	0.0%
4588 Corry 2019	0.0%	3.1%	0.0%	0.0%	0.0%	4.2%	6.3%	0.0%	3.2%	10.0%
5731 Piersol 2017	0.0%	0.0%	2.0%	2.6%	0.0%	9.2%	1.9%	0.0%	7.5%	1.9%
2104 Cuthbert 2017	0.0%	0.0%	0.0%	0.0%	0.0%	1.6%	0.0%	0.0%	1.9%	0.0%
3453 Collins 2019	0.0%	0.0%	11.1%	0.0%	0.0%	0.0%	0.0%	0.0%	1.8%	0.0%
615 Waldron 2013	0.0%	0.0%	0.0%	0.0%	0.0%	0.0%	0.0%	0.0%	0.0%	0.0%
3544 Boots 2014	0.0%	0.0%	0.0%	17.1%	4.3%	1.6%	0.0%	0.0%	0.0%	0.0%
3984 Doyle 2020	0.0%	0.0%	0.0%	0.0%	0.0%	0.0%	0.0%	0.0%	0.0%	0.0%
4285 Maffioletti 2019	0.0%	0.0%	0.0%	0.0%	0.0%	6.3%	0.0%	5.9%	1.8%	0.0%
2570 Lee 2020	0.0%	0.0%	3.0%	1.6%	2.7%	12.9%	0.0%	0.0%	12.9%	0.0%
2809 Weinbrecht 2016	0.0%	0.0%	0.0%	3.0%	4.8%	14.9%	4.7%	0.0%	13.4%	10.0%
3014 Wang 2020	0.0%	0.0%	0.0%	7.5%	18.8%	1.7%	5.3%	0.0%	4.1%	5.6%
152 Minshall 2019	0.0%	0.0%	0.0%	0.0%	2.5%	0.0%	0.0%	0.0%	0.0%	0.0%
588 Cheng 2020	0.0%	0.0%	4.4%	2.4%	2.1%	13.4%	5.0%	0.0%	23.8%	5.1%

1275
1128
74
40
33

For peer review only

1611 Smith 2019	0.0%	0.0%	0.0%	0.0%	5.3%	0.0%	0.0%	0.0%	0.0%	0.0%
248 Heckel 2019	0.0%	0.0%	0.0%	0.0%	0.0%	0.0%	0.0%	0.0%	0.0%	0.0%
1596 Egan 2018	0.0%	0.0%	0.0%	18.9%	17.6%	0.0%	0.0%	0.0%	2.0%	0.0%
1977 Zhao 2019	0.0%	0.0%	0.0%	13.5%	28.6%	0.0%	0.0%	0.0%	2.0%	0.0%
1992 Leng 2020	0.0%	0.0%	0.0%	20.5%	16.0%	0.0%	0.0%	0.0%	1.7%	0.0%
XT Maayan 2014	0.0%	0.0%	0.0%	0.0%	0.0%	1.8%	0.0%	13.0%	0.0%	0.0%
3195 Shaw 2009	0.0%	0.0%	0.0%	0.0%	0.0%	4.8%	0.0%	21.7%	0.0%	0.0%
2520 Teahan 2020	0.0%	0.0%	0.0%	0.0%	0.0%	10.3%	2.9%	0.0%	24.5%	0.0%
4562 GonzalezFraile 2021	0.0%	0.0%	3.3%	11.3%	9.4%	4.1%	2.9%	0.0%	8.1%	6.1%
3528 Amador-Marin 2017	0.0%	0.0%	0.0%	2.1%	0.0%	8.3%	0.0%	0.0%	7.7%	0.0%
5049 Sin 2018	0.0%	1.7%	0.0%	21.4%	13.0%	1.0%	0.0%	0.0%	1.1%	1.7%
4806 Parkinson 2019	0.0%	0.0%	0.0%	0.0%	0.0%	0.0%	0.0%	0.0%	0.0%	0.0%
5174 Bennett 2019	0.0%	0.0%	0.0%	0.0%	0.0%	8.3%	0.0%	0.0%	3.7%	0.0%
5810 Lucero 2019	0.0%	0.0%	0.0%	4.3%	0.0%	4.8%	0.0%	0.0%	3.7%	0.0%
4421 Treanor 2019	0.0%	0.0%	0.0%	0.0%	0.0%	0.0%	0.0%	0.0%	0.0%	0.0%
5244 Goeman 2016	0.0%	0.0%	0.0%	0.0%	0.0%	7.5%	0.0%	0.0%	1.1%	1.7%
5375 Greenwood 2016	0.0%	0.0%	0.0%	0.0%	0.0%	1.8%	0.0%	0.0%	0.0%	0.0%

For peer review only

OVE)

=	**Total nodes (pairs of reviews)**
=	Slight overlap (<5%)
=	Moderate overlap (5% to <10%)
=	High overlap (10% to <15%)
=	Very High overlap (≥15%)

3734 Fu 2017	3939 Jensen 2015	3965 Corvol 2017	3970 Hopkinson 2019	4267 Pucciarelli 2020	4415 Vernooij-Dassen 2011	4452 Reilly 2015	4530 Liu 2018	4531 Lins 2014	4534 Legg 2011	4567 Forster 2012	4588 Corry 2019	5731 Piersol 2017	2104 Cuthbert 2017	3453 Collins 2019
0.0%														
0.0%	0.0%													
0.0%	0.0%	0.0%												
0.0%	0.0%	0.0%	0.0%											
0.0%	0.0%	0.0%	16.1%	0.0%										
0.0%	0.0%	31.8%	0.0%	0.0%	0.0%									
0.0%	0.0%	0.0%	0.0%	0.0%	0.0%	0.0%								
0.0%	0.0%	0.0%	0.0%	0.0%	4.8%	0.0%	0.0%							
0.0%	0.0%	0.0%	0.0%	0.0%	0.0%	0.0%	0.0%	0.0%						
0.0%	0.0%	0.0%	0.0%	0.0%	0.0%	0.0%	0.0%	0.0%	11.5%					
2.4%	0.0%	0.0%	4.5%	2.8%	3.2%	0.0%	0.0%	6.7%	0.0%	0.0%				
0.0%	0.0%	7.4%	1.5%	0.0%	0.0%	7.8%	4.4%	0.0%	0.0%	0.0%	5.0%			
0.0%	0.0%	0.0%	0.0%	0.0%	0.0%	0.0%	0.0%	0.0%	0.0%	0.0%	3.4%	2.0%		
0.0%	0.0%	0.0%	2.8%	0.0%	0.0%	0.0%	30.8%	0.0%	0.0%	0.0%	0.0%	5.9%	0.0%	
0.0%	0.0%	0.0%	0.0%	0.0%	0.0%	0.0%	0.0%	0.0%	0.0%	0.0%	0.0%	0.0%	0.0%	0.0%
0.0%	0.0%	0.0%	0.0%	0.0%	4.5%	0.0%	0.0%	0.0%	0.0%	3.1%	0.0%	3.8%	0.0%	0.0%
0.0%	0.0%	0.0%	0.0%	0.0%	0.0%	0.0%	0.0%	0.0%	0.0%	0.0%	0.0%	0.0%	11.1%	0.0%
0.0%	0.0%	0.0%	0.0%	0.0%	0.0%	0.0%	0.0%	0.0%	0.0%	0.0%	2.9%	0.0%	0.0%	0.0%
0.0%	6.5%	7.7%	2.0%	0.0%	0.0%	8.3%	0.0%	0.0%	0.0%	0.0%	0.0%	11.5%	0.0%	0.0%
0.0%	8.3%	6.7%	5.6%	0.0%	2.4%	9.8%	0.0%	2.4%	0.0%	0.0%	1.9%	10.4%	0.0%	0.0%
0.0%	0.0%	0.0%	0.0%	0.0%	5.9%	0.0%	0.0%	5.9%	0.0%	0.0%	0.0%	0.0%	0.0%	0.0%
0.0%	0.0%	0.0%	0.0%	12.5%	0.0%	0.0%	0.0%	0.0%	12.1%	8.7%	2.0%	0.0%	0.0%	0.0%
0.0%	4.5%	3.5%	6.8%	0.0%	1.4%	4.3%	2.2%	4.3%	0.0%	0.0%	4.1%	10.1%	2.2%	2.8%

For peer review only

0.0%	0.0%	0.0%	0.0%	22.2%	0.0%	0.0%	0.0%	0.0%	12.5%	6.5%	4.3%	0.0%	0.0%	0.0%
0.0%	0.0%	0.0%	0.0%	0.0%	0.0%	0.0%	0.0%	2.0%	0.0%	0.0%	0.0%	0.0%	0.0%	0.0%
0.0%	0.0%	0.0%	0.0%	0.0%	5.6%	0.0%	0.0%	0.0%	0.0%	0.0%	0.0%	2.0%	0.0%	0.0%
0.0%	0.0%	0.0%	0.0%	0.0%	6.3%	0.0%	0.0%	0.0%	0.0%	0.0%	0.0%	2.1%	0.0%	0.0%
0.0%	0.0%	0.0%	0.0%	0.0%	3.7%	0.0%	0.0%	0.0%	0.0%	0.0%	0.0%	1.7%	0.0%	0.0%
0.0%	0.0%	0.0%	0.0%	0.0%	0.0%	0.0%	0.0%	0.0%	0.0%	0.0%	0.0%	0.0%	0.0%	0.0%
0.0%	0.0%	0.0%	1.7%	0.0%	0.0%	0.0%	0.0%	0.0%	0.0%	0.0%	0.0%	0.0%	0.0%	0.0%
0.0%	11.5%	2.7%	2.2%	0.0%	0.0%	6.1%	3.8%	0.0%	0.0%	0.0%	2.4%	14.3%	0.0%	3.0%
0.0%	0.0%	0.0%	0.0%	0.0%	0.0%	0.0%	0.0%	13.3%	0.0%	0.0%	10.0%	1.6%	0.0%	0.0%
0.0%	5.6%	3.7%	5.7%	0.0%	0.0%	4.2%	0.0%	0.0%	0.0%	0.0%	0.0%	10.2%	0.0%	0.0%
1.4%	0.0%	0.0%	0.0%	1.6%	0.0%	0.0%	0.0%	1.7%	0.0%	0.0%	0.0%	2.2%	0.0%	0.0%
0.0%	0.0%	0.0%	0.0%	0.0%	0.0%	0.0%	0.0%	0.0%	0.0%	0.0%	0.0%	0.0%	0.0%	0.0%
0.0%	0.0%	3.7%	0.0%	0.0%	0.0%	4.2%	0.0%	0.0%	0.0%	0.0%	0.0%	3.8%	0.0%	0.0%
0.0%	0.0%	0.0%	0.0%	0.0%	4.5%	4.2%	0.0%	15.0%	0.0%	0.0%	10.0%	5.9%	5.0%	0.0%
2.6%	0.0%	0.0%	0.0%	0.0%	0.0%	0.0%	0.0%	0.0%	0.0%	0.0%	0.0%	0.0%	0.0%	0.0%
0.0%	0.0%	14.5%	1.4%	0.0%	1.8%	15.4%	0.0%	0.0%	0.0%	0.0%	1.5%	4.7%	0.0%	0.0%
0.0%	0.0%	0.0%	0.0%	0.0%	0.0%	0.0%	0.0%	0.0%	0.0%	0.0%	0.0%	0.0%	0.0%	0.0%

For peer review only

[redacted]

	615 Waldron 2013	3544 Boots 2014	3984 Doyle 2020	4285 Maffioletti 2019	2570 Lee 2020	2809 Weinbrecht 2016	3014 Wang 2020	152 Minshall 2019	588 Cheng 2020	Smith 2019	2019
	0.0%										
	0.0%	0.0%									
	0.0%	0.0%	0.0%								
	0.0%	0.0%	0.0%	0.0%							
	0.0%	2.3%	0.0%	0.0%	18.4%						
	0.0%	5.6%	0.0%	0.0%	0.0%	2.6%					
	0.0%	0.0%	0.0%	0.0%	0.0%	0.0%	0.0%				
	0.0%	0.7%	0.0%	0.7%	13.6%	18.7%	2.2%	0.0%			

For peer review only

0.0%	0.0%	0.0%	0.0%	0.0%	0.0%	0.0%	14.0%	0.0%	1611 :					
0.0%	0.0%	0.0%	0.0%	0.0%	0.0%	0.0%	0.0%	0.0%	0.0%	248 Heckel				
0.0%	17.6%	0.0%	0.0%	3.0%	5.3%	25.0%	0.0%	2.2%	0.0%	0.0%	1596 Egan 2018			
0.0%	5.9%	0.0%	0.0%	6.7%	8.6%	30.0%	0.0%	3.0%	0.0%	0.0%	55.6%	1977 Zhao 2019		
0.0%	7.4%	0.0%	0.0%	4.9%	6.5%	14.3%	0.0%	4.9%	0.0%	0.0%	31.6%	35.3%	1992 Leng 2020	
0.0%	0.0%	0.0%	0.0%	3.4%	0.0%	0.0%	0.0%	0.7%	0.0%	0.0%	0.0%	0.0%	0.0%	XT Maayan 2014
0.0%	0.0%	0.0%	9.1%	0.0%	0.0%	0.0%	0.0%	0.0%	0.0%	0.0%	0.0%	0.0%	0.0%	5.6%
0.0%	0.0%	0.0%	2.9%	17.1%	12.5%	0.0%	0.0%	14.8%	0.0%	0.0%	0.0%	0.0%	0.0%	4.0%
0.0%	2.9%	0.0%	2.8%	4.3%	7.8%	7.1%	0.0%	10.6%	0.0%	1.6%	19.2%	20.8%	33.3%	0.0%
0.0%	0.0%	0.0%	0.0%	22.6%	12.8%	0.0%	0.0%	6.6%	0.0%	0.0%	5.3%	5.9%	3.6%	0.0%
0.0%	5.2%	0.0%	0.0%	2.7%	2.5%	3.7%	1.3%	3.4%	1.3%	0.0%	9.6%	7.8%	8.2%	0.0%
0.0%	0.0%	0.0%	0.0%	0.0%	0.0%	0.0%	0.0%	0.0%	0.0%	0.0%	0.0%	0.0%	0.0%	0.0%
0.0%	0.0%	0.0%	0.0%	2.7%	4.8%	0.0%	0.0%	3.6%	0.0%	0.0%	0.0%	0.0%	0.0%	0.0%
0.0%	4.3%	0.0%	0.0%	2.7%	4.8%	0.0%	0.0%	5.1%	0.0%	0.0%	11.1%	5.9%	7.4%	0.0%
9.1%	0.0%	0.0%	0.0%	0.0%	0.0%	0.0%	0.0%	0.0%	0.0%	0.0%	0.0%	0.0%	0.0%	0.0%
0.0%	0.0%	0.0%	0.0%	4.3%	5.3%	0.0%	0.0%	3.4%	1.4%	0.0%	0.0%	0.0%	0.0%	0.0%
0.0%	0.0%	0.0%	0.0%	3.4%	0.0%	0.0%	0.0%	0.0%	0.0%	0.0%	0.0%	0.0%	0.0%	0.0%

For peer review only

[redacted]

For peer review only

3195 Shaw 2009	2520 Teahan 2020	4562 GonzalezFraile 2021	3528 Amador-Marin 2017	5049 Sin 2018	4806 Parkinson 2019	5174 Bennett 2019	5810 Lucero 2019	4421 Treanor 2019	5244 Goeman 2016
0.0%									
0.0%	4.7%								
0.0%	9.7%	2.9%							
0.0%	0.0%	9.1%	1.7%						
0.0%	0.0%	0.0%	0.0%	0.0%					
0.0%	3.0%	0.0%	0.0%	0.0%	0.0%				
0.0%	3.0%	25.0%	4.3%	3.4%	0.0%	0.0%			
0.0%	0.0%	0.0%	0.0%	0.0%	0.0%	0.0%	0.0%		
0.0%	1.5%	1.4%	1.7%	2.1%	0.0%	1.7%	3.5%	0.0%	
0.0%	0.0%	0.0%	0.0%	0.0%	0.0%	0.0%	0.0%	0.0%	2.0%

For peer review only

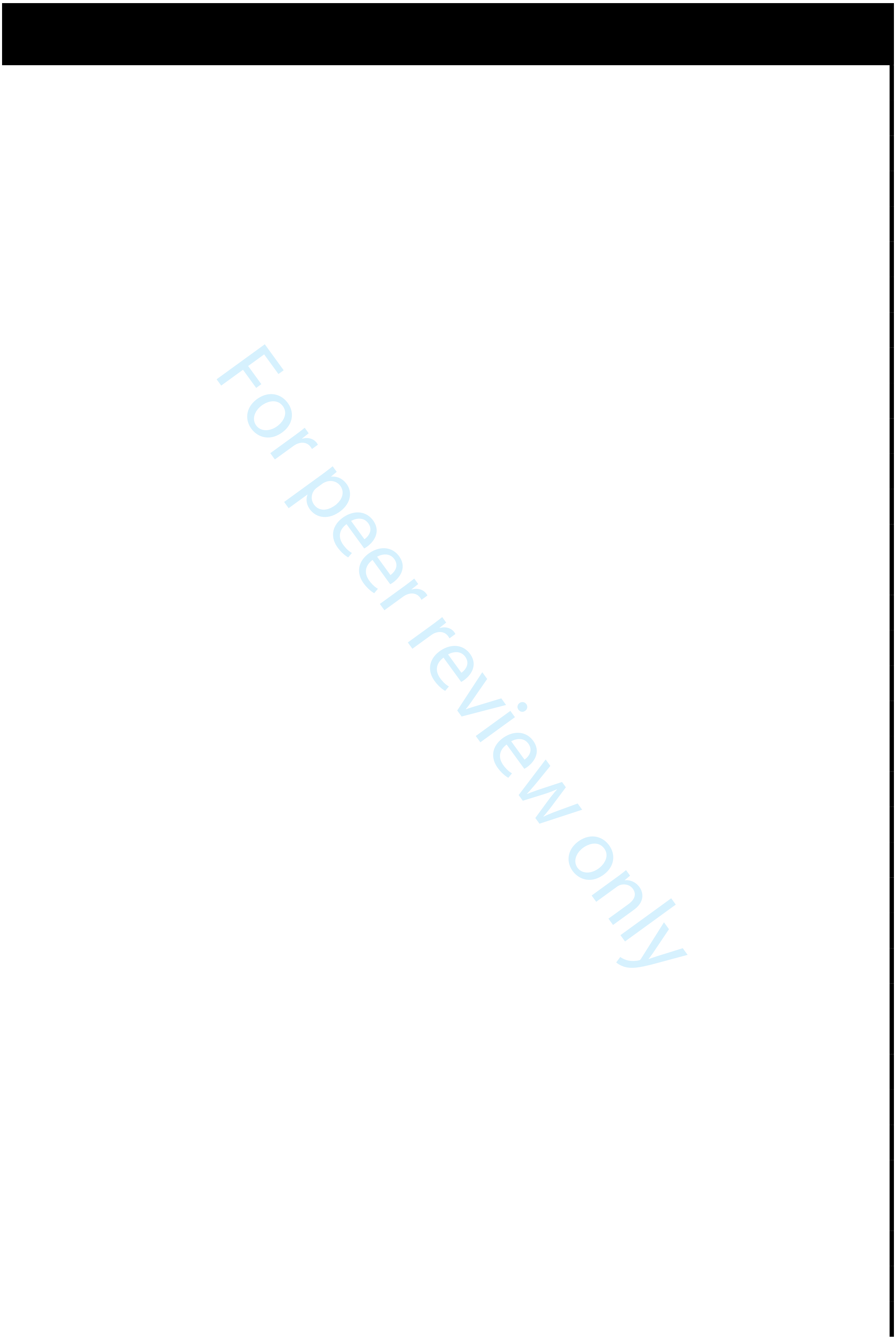

For peer review only

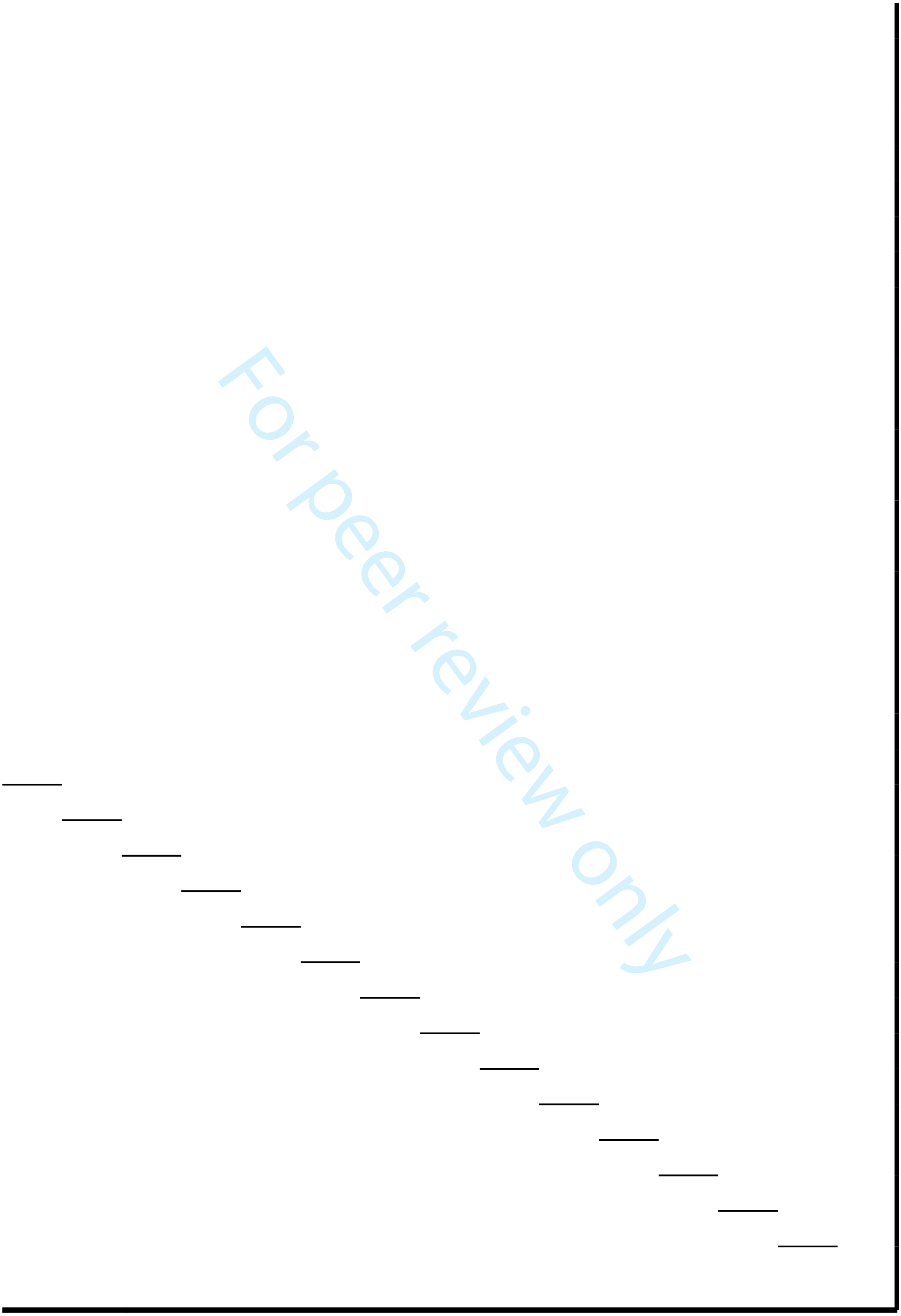

For peer review only

Graphical Representation of Overlap for OVErviews (G

	807 Irons 2	1048 Gabriel 202	2195 Shim 2020	2367 Hopwood 2018	2370 Guay 2017	2491 Vandepitte 2016	2524 Zabihi 2020	3197 Mason 2007
1048 Gabriel 2020	0.0%							
2195 Shim 2020	0.0%	0.0%						
2367 Hopwood 2018	0.0%	0.0%	0.0%					
2370 Guay 2017	0.0%	0.0%	0.0%	7.5%				
2491 Vandepitte 2016	0.0%	0.0%	0.0%	0.0%	0.0%			
2524 Zabihi 2020	0.0%	0.0%	0.0%	0.0%	0.0%	1.5%		
3197 Mason 2007	0.0%	0.0%	0.0%	0.0%	0.0%	7.5%	0.0%	
5257 Wiegelmann 2021	0.0%	0.0%	2.0%	1.3%	2.1%	14.3%	5.6%	0.0%
3552 Akarsu 2019	0.0%	0.0%	0.0%	0.0%	0.0%	1.6%	13.6%	0.0%
3734 Fu 2017	0.0%	22.2%	0.0%	0.0%	3.0%	0.0%	0.0%	0.0%
3939 Jensen 2015	0.0%	0.0%	0.0%	0.0%	0.0%	9.4%	0.0%	0.0%
3965 Corvol 2017	0.0%	0.0%	0.0%	0.0%	0.0%	1.5%	0.0%	0.0%
3970 Hopkinson 2019	0.0%	0.0%	0.0%	0.0%	0.0%	4.0%	11.8%	3.2%
4267 Pucciarelli 2020	0.0%	0.0%	0.0%	0.0%	7.7%	0.0%	0.0%	0.0%
4415 Vernooij-Dassen 2011	0.0%	0.0%	0.0%	5.3%	7.1%	0.0%	13.3%	0.0%
4452 Reilly 2015	0.0%	0.0%	0.0%	0.0%	0.0%	3.2%	0.0%	0.0%
4530 Liu 2018	0.0%	0.0%	18.2%	0.0%	0.0%	0.0%	0.0%	0.0%
4531 Lins 2014	0.0%	0.0%	0.0%	0.0%	0.0%	5.3%	0.0%	0.0%
4534 Legg 2011	0.0%	0.0%	0.0%	0.0%	0.0%	0.0%	0.0%	0.0%
4567 Forster 2012	0.0%	0.0%	0.0%	3.3%	0.0%	0.0%	0.0%	0.0%
4588 Corry 2019	0.0%	3.1%	0.0%	0.0%	0.0%	4.2%	6.3%	0.0%
5731 Piersol 2017	0.0%	0.0%	2.1%	2.8%	0.0%	9.2%	1.9%	0.0%
2104 Cuthbert 2017	0.0%	0.0%	0.0%	0.0%	0.0%	1.6%	0.0%	0.0%
3453 Collins 2019	0.0%	0.0%	11.1%	0.0%	0.0%	0.0%	0.0%	0.0%
615 Waldron 2013	0.0%	0.0%	0.0%	0.0%	0.0%	0.0%	0.0%	0.0%
3544 Boots 2014	0.0%	0.0%	0.0%	38.9%	5.0%	1.7%	0.0%	0.0%
3984 Doyle 2020	0.0%	0.0%	0.0%	0.0%	0.0%	0.0%	0.0%	0.0%
4285 Maffioletti 2019	0.0%	0.0%	0.0%	0.0%	0.0%	6.3%	0.0%	7.4%
2570 Lee 2020	0.0%	0.0%	3.0%	1.6%	2.9%	13.8%	0.0%	0.0%
2809 Weinbrecht 2016	0.0%	0.0%	0.0%	3.7%	4.8%	14.9%	5.0%	0.0%
3014 Wang 2020	0.0%	0.0%	0.0%	7.5%	18.8%	1.7%	5.3%	0.0%
152 Minshall 2019	0.0%	0.0%	0.0%	0.0%	2.6%	0.0%	0.0%	0.0%
588 Cheng 2020	0.0%	0.0%	4.4%	2.5%	2.4%	16.7%	5.0%	0.0%

For peer review only

1611 Smith 2019	0.0%	0.0%	0.0%	0.0%	5.4%	0.0%	0.0%	0.0%
248 Heckel 2019	0.0%	0.0%	0.0%	0.0%	0.0%	0.0%	0.0%	0.0%
1596 Egan 2018	0.0%	0.0%	0.0%	20.0%	17.6%	0.0%	0.0%	0.0%
1977 Zhao 2019	0.0%	0.0%	0.0%	13.5%	28.6%	0.0%	0.0%	0.0%
1992 Leng 2020	0.0%	0.0%	0.0%	24.3%	23.5%	0.0%	0.0%	0.0%
XT Maayan 2014	0.0%	0.0%	0.0%	0.0%	0.0%	1.9%	0.0%	13.6%
3195 Shaw 2009	0.0%	0.0%	0.0%	0.0%	0.0%	6.3%	0.0%	21.7%
2520 Teahan 2020	0.0%	0.0%	0.0%	0.0%	0.0%	10.3%	2.9%	0.0%
4562 GonzalezFraile 2021	0.0%	0.0%	3.4%	12.2%	10.7%	4.4%	2.9%	0.0%
3528 Amador-Marin 2017	0.0%	0.0%	0.0%	2.4%	0.0%	8.3%	0.0%	0.0%
5049 Sin 2018	0.0%	1.7%	0.0%	22.1%	13.0%	1.0%	0.0%	0.0%
4806 Parkinson 2019	0.0%	0.0%	0.0%	0.0%	0.0%	0.0%	0.0%	0.0%
5174 Bennett 2019	0.0%	0.0%	0.0%	0.0%	0.0%	8.6%	0.0%	0.0%
5810 Lucero 2019	0.0%	0.0%	0.0%	4.3%	0.0%	4.8%	0.0%	0.0%
4421 Treanor 2019	0.0%	0.0%	0.0%	0.0%	0.0%	0.0%	0.0%	0.0%
5244 Goeman 2016	0.0%	0.0%	0.0%	0.0%	0.0%	7.5%	0.0%	0.0%
5375 Greenwood 2016	0.0%	0.0%	0.0%	0.0%	0.0%	1.8%	0.0%	0.0%

For peer review only

ROOVE) - Considering Structural Missingness

**1275**	=	**Total nodes (pairs of reviews)**
1107	=	Slight overlap (<5%)
87	=	Moderate overlap (5% to <10%)
42	=	High overlap (10% to <15%)
39	=	Very High overlap (≥15%)

5257 Wiegelmann 2021	3552 Akarsu 2019	3734 Fu 2017	3939 Jensen 2015	3965 Corvol 2017	3970 Hopkinson 2019	4267 Pucciarelli 2020	4415 Vernooij-Dassen 2011	4452 Reilly 2015	4530 Liu 2018	4531 Lins 2014	4534 Legg 2011	4567 Forster 2012	4588 Corry 2019
1.8%													
0.0%	0.0%												
25.0%	0.0%	0.0%											
0.0%	0.0%	0.0%	0.0%										
6.2%	8.8%	0.0%	0.0%	0.0%									
0.0%	0.0%	0.0%	0.0%	0.0%	0.0%								
0.0%	5.9%	0.0%	0.0%	0.0%	26.3%	0.0%							
0.0%	0.0%	0.0%	0.0%	31.8%	0.0%	0.0%	0.0%						
0.0%	0.0%	0.0%	0.0%	0.0%	0.0%	0.0%	0.0%	0.0%					
0.0%	5.3%	0.0%	0.0%	0.0%	0.0%	0.0%	5.3%	0.0%	0.0%				
0.0%	0.0%	0.0%	0.0%	0.0%	0.0%	0.0%	0.0%	0.0%	0.0%	0.0%			
0.0%	0.0%	0.0%	0.0%	0.0%	0.0%	0.0%	0.0%	0.0%	0.0%	0.0%	11.5%		
3.2%	10.0%	2.4%	0.0%	0.0%	4.5%	2.8%	6.3%	0.0%	0.0%	8.0%	0.0%	0.0%	
8.6%	1.9%	0.0%	0.0%	7.4%	1.5%	0.0%	0.0%	8.0%	4.4%	0.0%	0.0%	0.0%	5.0%
2.4%	0.0%	0.0%	0.0%	0.0%	0.0%	0.0%	0.0%	0.0%	0.0%	0.0%	0.0%	0.0%	3.4%
1.8%	0.0%	0.0%	0.0%	0.0%	2.8%	0.0%	0.0%	0.0%	30.8%	0.0%	0.0%	0.0%	0.0%
0.0%	0.0%	0.0%	0.0%	0.0%	0.0%	0.0%	0.0%	0.0%	0.0%	0.0%	0.0%	0.0%	0.0%
0.0%	0.0%	0.0%	0.0%	0.0%	0.0%	0.0%	5.6%	0.0%	0.0%	0.0%	0.0%	3.2%	0.0%
0.0%	0.0%	0.0%	0.0%	0.0%	0.0%	0.0%	0.0%	0.0%	0.0%	0.0%	0.0%	0.0%	0.0%
1.8%	0.0%	0.0%	0.0%	0.0%	0.0%	0.0%	0.0%	0.0%	0.0%	0.0%	0.0%	0.0%	2.9%
12.9%	0.0%	0.0%	9.1%	8.3%	2.0%	0.0%	0.0%	11.1%	0.0%	0.0%	0.0%	0.0%	0.0%
18.4%	10.0%	0.0%	8.6%	6.7%	5.9%	0.0%	4.0%	10.3%	0.0%	2.6%	0.0%	0.0%	2.1%
4.1%	5.6%	0.0%	0.0%	0.0%	0.0%	0.0%	7.1%	0.0%	0.0%	6.7%	0.0%	0.0%	0.0%
0.0%	0.0%	0.0%	0.0%	0.0%	0.0%	12.5%	0.0%	0.0%	0.0%	0.0%	19.0%	11.4%	2.0%
23.8%	5.1%	0.0%	7.1%	4.0%	6.8%	0.0%	4.3%	6.7%	2.3%	7.3%	0.0%	0.0%	4.1%

For peer review only

0.0%	0.0%	0.0%	0.0%	0.0%	0.0%	22.2%	0.0%	0.0%	0.0%	0.0%	22.2%	9.1%	4.3%
0.0%	0.0%	0.0%	0.0%	0.0%	0.0%	0.0%	0.0%	0.0%	0.0%	2.4%	0.0%	0.0%	0.0%
2.1%	0.0%	0.0%	0.0%	0.0%	0.0%	0.0%	7.7%	0.0%	0.0%	0.0%	0.0%	0.0%	0.0%
2.0%	0.0%	0.0%	0.0%	0.0%	0.0%	0.0%	9.1%	0.0%	0.0%	0.0%	0.0%	0.0%	0.0%
1.7%	0.0%	0.0%	0.0%	0.0%	0.0%	0.0%	8.3%	0.0%	0.0%	0.0%	0.0%	0.0%	0.0%
0.0%	0.0%	0.0%	0.0%	0.0%	0.0%	0.0%	0.0%	0.0%	0.0%	0.0%	0.0%	0.0%	0.0%
0.0%	0.0%	0.0%	0.0%	0.0%	2.2%	0.0%	0.0%	0.0%	0.0%	0.0%	0.0%	0.0%	0.0%
24.5%	0.0%	0.0%	14.3%	2.7%	2.2%	0.0%	0.0%	7.1%	3.8%	0.0%	0.0%	0.0%	2.4%
8.1%	6.7%	0.0%	0.0%	0.0%	0.0%	0.0%	0.0%	0.0%	0.0%	21.1%	0.0%	0.0%	10.8%
9.5%	0.0%	0.0%	6.3%	3.7%	5.7%	0.0%	0.0%	4.5%	0.0%	0.0%	0.0%	0.0%	0.0%
1.1%	1.7%	1.4%	0.0%	0.0%	0.0%	1.6%	0.0%	0.0%	0.0%	2.6%	0.0%	0.0%	0.0%
0.0%	0.0%	0.0%	0.0%	0.0%	0.0%	0.0%	0.0%	0.0%	0.0%	0.0%	0.0%	0.0%	0.0%
3.7%	0.0%	0.0%	0.0%	4.0%	0.0%	0.0%	0.0%	4.5%	0.0%	0.0%	0.0%	0.0%	0.0%
3.7%	0.0%	0.0%	0.0%	0.0%	0.0%	0.0%	5.3%	4.3%	0.0%	15.8%	0.0%	0.0%	10.0%
0.0%	0.0%	2.6%	0.0%	0.0%	0.0%	0.0%	0.0%	0.0%	0.0%	0.0%	0.0%	0.0%	0.0%
1.4%	1.7%	0.0%	0.0%	14.5%	1.4%	0.0%	2.2%	15.4%	0.0%	0.0%	0.0%	0.0%	1.5%
0.0%	0.0%	0.0%	0.0%	0.0%	0.0%	0.0%	0.0%	0.0%	0.0%	0.0%	0.0%	0.0%	0.0%

For peer review only

[redacted]

For peer review only

5731 Piersol 2017	2104 Cuthbert 2017	3453 Collins 2019	615 Waldron 2013	3544 Boots 2014	3984 Doyle 2020	4285 Maffioletti 2019	2570 Lee 2020	2809 Weinbrecht 2016	3014 Wang 2020	152 Minshall 2019	588 Cheng 2020	Smith 2019	2019
2.0%													
5.9%	0.0%												
0.0%	0.0%	0.0%											
3.9%	0.0%	0.0%	0.0%										
0.0%	11.1%	0.0%	0.0%	0.0%									
0.0%	0.0%	0.0%	0.0%	0.0%	0.0%								
12.1%	0.0%	0.0%	0.0%	0.0%	0.0%	0.0%							
10.4%	0.0%	0.0%	0.0%	2.5%	0.0%	0.0%	20.5%						
0.0%	0.0%	0.0%	0.0%	6.3%	0.0%	0.0%	0.0%	2.7%					
0.0%	0.0%	0.0%	0.0%	0.0%	0.0%	0.0%	0.0%	0.0%	0.0%				
11.5%	2.5%	2.8%	0.0%	1.1%	0.0%	0.7%	13.6%	24.8%	2.2%	0.0%			

0.0%	0.0%	0.0%	0.0%	0.0%	0.0%	0.0%	0.0%	0.0%	0.0%	14.0%	0.0%	1611 :	248 Heckel
0.0%	0.0%	0.0%	0.0%	0.0%	0.0%	0.0%	0.0%	0.0%	0.0%	0.0%	0.0%	0.0%	
2.0%	0.0%	0.0%	0.0%	20.0%	0.0%	0.0%	3.0%	5.3%	25.0%	0.0%	2.2%	0.0%	0.0%
2.1%	0.0%	0.0%	0.0%	7.1%	0.0%	0.0%	6.7%	8.6%	30.0%	0.0%	3.0%	0.0%	0.0%
2.0%	0.0%	0.0%	0.0%	14.3%	0.0%	0.0%	4.9%	8.1%	14.3%	0.0%	4.9%	0.0%	0.0%
0.0%	0.0%	0.0%	0.0%	0.0%	0.0%	0.0%	5.3%	0.0%	0.0%	0.0%	1.3%	0.0%	0.0%
0.0%	0.0%	0.0%	0.0%	0.0%	0.0%	10.3%	0.0%	0.0%	0.0%	0.0%	0.0%	0.0%	0.0%
14.3%	0.0%	3.0%	0.0%	0.0%	0.0%	2.9%	17.1%	12.5%	0.0%	0.0%	14.8%	0.0%	0.0%
1.7%	0.0%	0.0%	0.0%	4.3%	0.0%	3.0%	4.3%	8.7%	7.4%	0.0%	10.7%	0.0%	1.7%
10.2%	0.0%	0.0%	0.0%	0.0%	0.0%	0.0%	25.0%	12.8%	0.0%	0.0%	7.8%	0.0%	0.0%
2.2%	0.0%	0.0%	0.0%	8.1%	0.0%	0.0%	2.7%	2.7%	3.7%	1.3%	3.5%	1.3%	0.0%
0.0%	0.0%	0.0%	0.0%	0.0%	0.0%	0.0%	0.0%	0.0%	0.0%	0.0%	0.0%	0.0%	0.0%
4.0%	0.0%	0.0%	0.0%	0.0%	0.0%	0.0%	2.7%	5.0%	0.0%	0.0%	3.6%	0.0%	0.0%
5.9%	5.0%	0.0%	0.0%	4.5%	0.0%	0.0%	2.7%	4.8%	0.0%	0.0%	5.1%	0.0%	0.0%
0.0%	0.0%	0.0%	11.1%	0.0%	0.0%	0.0%	0.0%	0.0%	0.0%	0.0%	0.0%	0.0%	0.0%
4.7%	0.0%	0.0%	0.0%	0.0%	0.0%	0.0%	4.6%	5.3%	0.0%	0.0%	4.2%	1.4%	0.0%
0.0%	0.0%	0.0%	0.0%	0.0%	0.0%	0.0%	4.2%	0.0%	0.0%	0.0%	0.0%	0.0%	0.0%

For peer review only

[redacted]

For peer review only

1596 Egan 2018	1977 Zhao 2019	1992 Leng 2020	XT Maayan 2014	3195 Shaw 2009	2520 Teahan 2020	4562 GonzalezFraile 2021	3528 Amador-Marin 2017	5049 Sin 2018	4806 Parkinson 2019	5174 Bennett 2019	5810 Lucero 2019	4421 Treanor 2019	5244 Goeman 2016
55.6%													
50.0%	50.0%												
0.0%	0.0%	0.0%											
0.0%	0.0%	0.0%	5.7%										
0.0%	0.0%	0.0%	5.0%	0.0%									
22.7%	23.8%	34.5%	0.0%	0.0%	4.8%								
5.3%	5.9%	5.0%	0.0%	0.0%	9.7%	3.3%							
9.6%	7.8%	9.3%	0.0%	0.0%	0.0%	9.7%	1.7%						
0.0%	0.0%	0.0%	0.0%	0.0%	0.0%	0.0%	0.0%	0.0%					
0.0%	0.0%	0.0%	0.0%	0.0%	3.0%	0.0%	0.0%	0.0%	0.0%				
11.1%	5.9%	9.1%	0.0%	0.0%	3.0%	28.0%	4.3%	3.4%	0.0%	0.0%			
0.0%	0.0%	0.0%	0.0%	0.0%	0.0%	0.0%	0.0%	0.0%	0.0%	0.0%	0.0%		
0.0%	0.0%	0.0%	0.0%	0.0%	1.5%	1.6%	1.7%	2.2%	0.0%	1.8%	3.5%	0.0%	
0.0%	0.0%	0.0%	0.0%	0.0%	0.0%	0.0%	0.0%	0.0%	0.0%	0.0%	0.0%	0.0%	2.0%

For peer review only

[redacted]

For peer review only

For peer review only

For peer review only

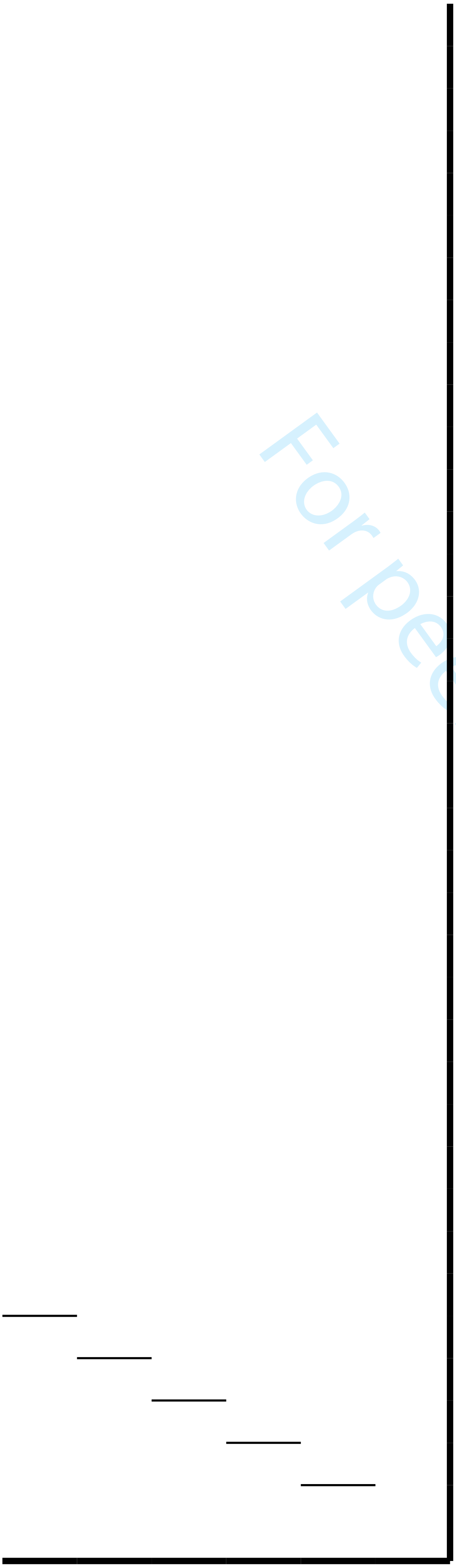

For peer review only

GROOVE tool: Graphica

DOI: 10.17605/OSF.IO/U2MS4

What is GROOVE?

GROOVE (**G**raphical **R**epresentation of **O**verlap for **OVE**rviews) is a tool that provides a visual display o

Starting with a simple matrix of evidence, this tool will automatically inform:
- The corrected covered area for the whole matrix.
- The number of unique primary studies.
- The number of overlapped primary studies (and in how many reviews they are included).
- A detailed assessment of the corrected covered area between every possible pair of reviews (nodes),
- The number of nodes with low, moderate, high and very high overlap.
- Optionally, an assessment of the corrected covered area corrected by structural missingness in the m

This tool is based on a previously published approach. If you are not familiar with the quantitative asse
Neugebauer EAM, Eikermann M. Systematic review finds overlapping reviews were not mentioned in e

How to use this tool:

You only have to enter data on the "1. Matrix" sheet. The matrix supports up to 70 systematic reviews
You will get all the results on the "GROOVE" sheets (colour or greyscale version).

1. Go to the "1. Matrix" sheet.
2. Write all the systematic reviews ID in the columns, starting in the C5 cell.
3. Write all the primary studies ID in the rows, starting in the A6 cell.
4. Optionally, you can write the topic of your overview in the B2 cell, and the references of each prima
5. Mark with a "1" the intersections representing primary studies included in their respective systemat
6. Go to the "2.1 GROOVE - colour version" or "2.2 GROOVE - grayscale version" tab. You will find the c
moderate, high and very high overlap, and the number of unique primary studies and overlapped prim

Advanced usage: Incorporating structural missingness

While you are building the matrix of evidence, you will notice that it should be impossible to mark a "1
published in 2015 can not be included in a systematic review published in 2013; this would be a "chron

The GROOVE tool provides the option to incorporate structural missingness into the results. In order t
1. Follow all the steps mentioned in the previous section.
2. When you finish populating the matrix of evidence with "1"s, you can mark the cells with structural
3. Once you finish, go to the "2.1 GROOVE - colour version" or "2.2 GROOVE - grayscale version" tab. Y
4. The Graphical Representation of Overlap for OVErviews incorporating structural missingness will be
structural missingness in the matrix, this figure will be the same as the one displayed in F1.

Example: How to enter data into the matrix

MATRIX OF EVIDENCE

TOPIC:

Study ID	Reference	Bonfill 2012	Urrútia 2014	Madrid 201[illegible]
	**Primary Studies**	**Systematic** [illegible]		
Bonham 1980		1		
Lennon 1980		1	1	1
Mercury 1991		1		
Harrison 2001			1	1
Jones 2007			1	
McCartney 2010			1	1
Taylor 2011				1
Page 2012				1
Deacon 2013		X		1
May 2013		X	1	1
Starr 2019		X	X	X
Plant 2020		X	X	X

2. Write all the primary studies ID here (references are optional)

Overall results

Number of columns (number of reviews)	c	4
Number of rows (number of index publications)	r	12
Number of included primary studies (including double counting)	N	20
Covered area	N/(rc)	41,67%
Corrected covered area	(N-r)/(rc-r)	22,22%
Interpretation of overlap	**Very High overlap**	
Structural Zeros	X	8
Corrected covered area (adjusting by structural zeros)	(N-r)/(rc-r-X)	28,57%

N° of non-overlapped primary studies	In 1 SR	7
Number of overlapped primary studies	In 2 SRs	2
	In 3 SRs	3
	In 4 SRs	0
	In 5 SRs	0
	In 6 SRs	0
	In 7 SRs	0
	In 8 SRs	0
	In 9 SRs	0
	In 10 SRs	0
	In 11 SRs	0
	In 12 SRs	0
	In 13 SRs	0
	In 14 SRs	0
	In 15 or more SRs	0

5. Go to the "2.1 GROO[illegible] - grayscale version" she[illegible]

Graphical Representat[illegible]

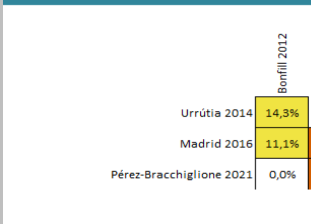

Graphical Represent[illegible]

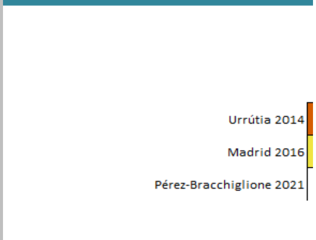

Cochrane Chile

Cochrane Iberoamérica

For peer review only

al Representation of Overla

of the overlap of primary studies among systematic reviews

, and a graphical representation of this assessment.

natrix.

essment of overlap using the corrected covered area formul
every other overview. Journal of Clinical Epidemiology2014;

and 1000 unique primary studies.

ary study in the B column, starting with B6.
tic reviews. It is expected for a single primary study to be i
corrected covered area (CCA) for the whole matrix, the CC
nary studies.

1" in some cells. A cell that can not acquire other value tha
nological structural missingness".

to do this, you will have to:

missingness with an "X". You will notice that the cell will t
You will notice that the D9 cell will provide the CCA formula
e displayed in the "2.1 GROOVE - colour version" and "2.2 G

iew only

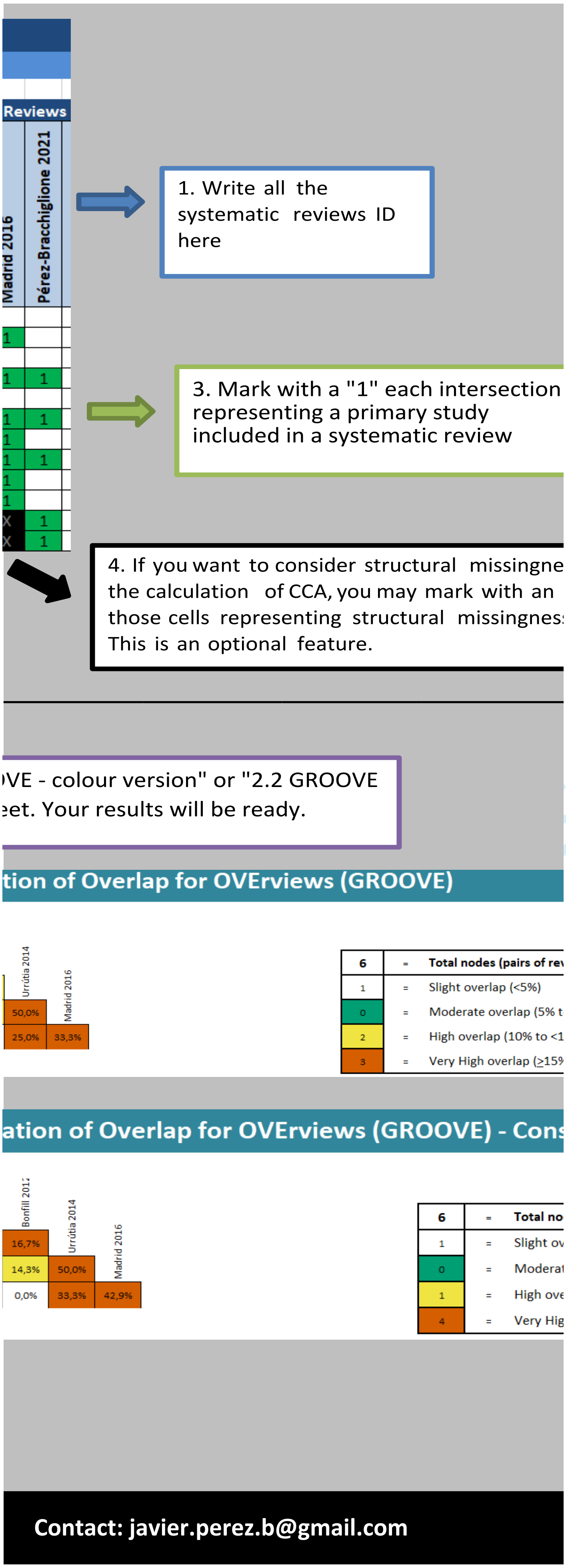
Reviews
Madrid 2016
Pérez-Bracchiglione 2021
1. Write all the systematic reviews ID here
3. Mark with a "1" each intersection representing a primary study included in a systematic review
4. If you want to consider structural missingne
the calculation of CCA, you may mark with an
those cells representing structural missingnes
This is an optional feature.
VE - colour version" or "2.2 GROOVE
eet. Your results will be ready.
tion of Overlap for OVErviews (GROOVE)
Urrútia 2014
Madrid 2016
50,0%
25,0%
33,3%
6 = Total nodes (pairs of rev
1 = Slight overlap (<5%)
0 = Moderate overlap (5% t
2 = High overlap (10% to <1
3 = Very High overlap (≥15%
ation of Overlap for OVErviews (GROOVE) - Cons
Bonfill 2012
Urrútia 2014
Madrid 2016
16,7%
14,3%
50,0%
0,0%
33,3%
42,9%
6 = Total no
1 = Slight ov
0 = Moderat
1 = High ove
4 = Very Hig
Contact: javier.perez.b@gmail.com

iew only

Universidad de Valparaíso CHILE

U[illegible]

For peer review only

ap for OVErviews

s.

la, we suggest you to read the original publication: Pieper D, Antoine S-L, Mathes T,
:67(4):368–75.

ncluded in multiple systematic reviews.
A for each pair (or nodes) of systematic reviews, the number of nodes with low,

n 0 is called "structural missingness" or "structural zero". For example, a primary study

urn black.
a corrected by structural missingness.
GROOVE - grayscale version" tabs, starting in the cell CA1. If you do not mark any

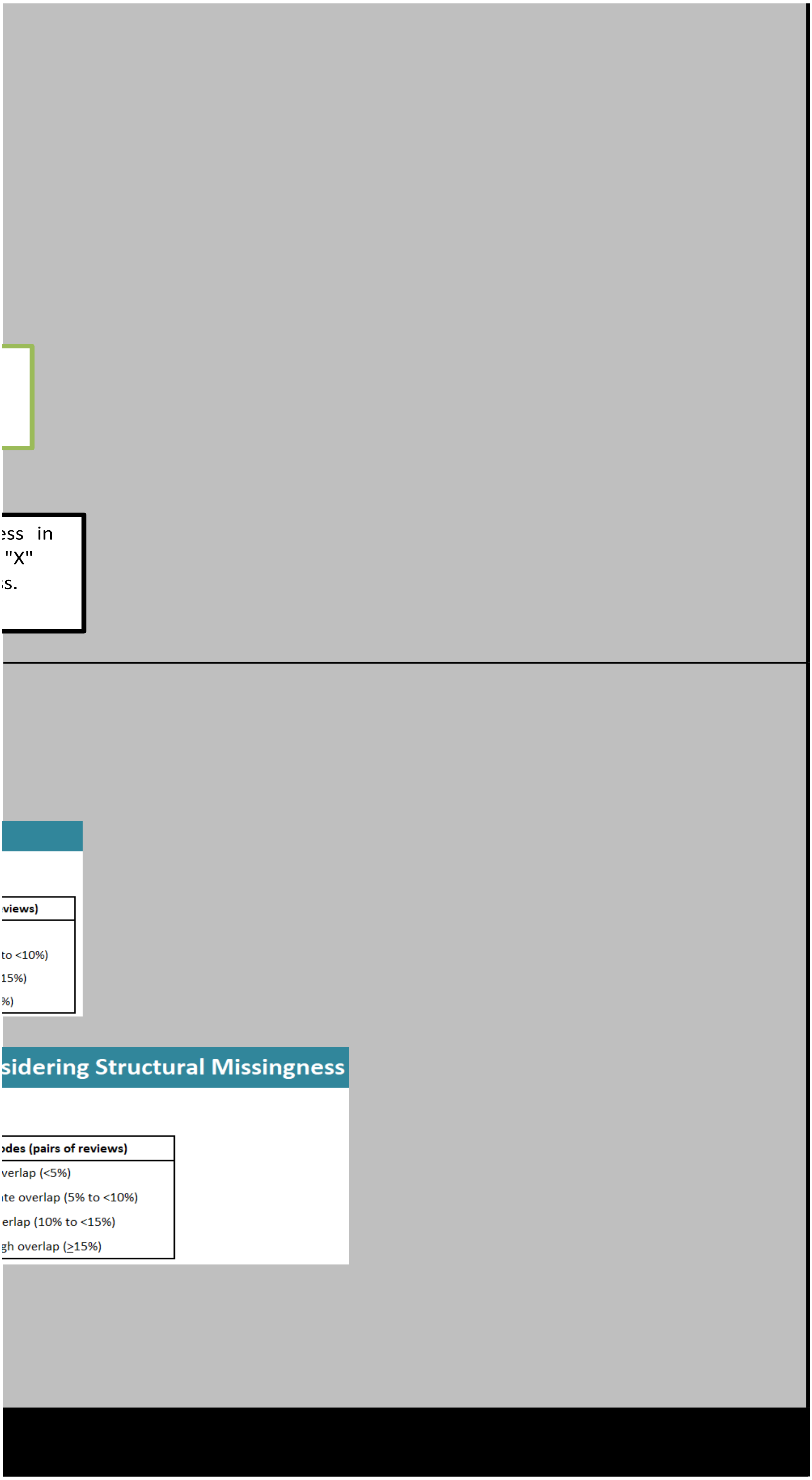
ess in
"X"
s.
views)
to <10%)
15%)
%)
sidering Structural Missingness
odes (pairs of reviews)
verlap (<5%)
te overlap (5% to <10%)
erlap (10% to <15%)
gh overlap (≥15%)

UAB
Universitat Autònoma de Barcelona

CIESAL
CENTRO INTERDISCIPLINARIO DE ESTUDIOS EN SALUD
UNIVERSIDAD DE VALPARAÍSO

For peer review only

MATRIX OF EVIDENCE

TOPIC: Informal caregiving UR

Primary Studies		Systematic Reviews								
**Study ID**	**Reference**	5244 Goeman 20	3366 Bunn 2016	3544 Boots 2014	4285 Maffioletti 2	4531 Lins 2014	5481 Pritchard 20	3368 Bourne 202	3718 DuPreez 20	807 Irons 2020
BustonHope2005			1							
Hibberd2011			1							
Quinn2009			1							
WoodsAlgar2009			1							
Ducharme2011				1						
Kelly2004				1						
Torp2008				1						
Chiu2009				1						
Rokstad2017		X	X	X	1	X				
Tretteteig2017		X	X	X	1	X				
Liu2015				X	1	X				
Zarit2014				X	1	X				
Gustafsdottir2014				X	1	X			1	
Philipson2012					1					
deJong2009					1					
Chang2004						1				
Salfi2005						1				
Acharya2015				X		X	1			
Bauer2011							1			
DouglasDunbar2007				X		X	1			
Jamieson2014				X		X	1			
Mockford2017		X	X	X		X	1			
Moyle2016		X	X				1			
Naylor2005							1			
Camic2014				X		X		1		1
Eekelaar2012								1		
McGuigan2015				X		X		1		
Flatt2015				X		X		1		1
Camic2016		X	X	X		X		1		
Phinney2011									1	
Donath2011									1	
Gill2011									1	
Lloyd2011									1	
Robinson2012									1	
Gaugler2014				X		X			1	
Stirling2014				X		X			1	
Hynes2016				X		X			1	
Logsdon2016		X	X	X		X			1	
Unadkat2017		X	X			X				1
Osman2016		X	X			X				1

Hunt2018						X				1
Dassa2018						X				1
Clark2018						X				1
Burnside2015						X				1
Baker2017		X	X			X				1
Stockle2016		X	X			X				
Kovaleva2017		X	X			X				
Marziali2018		X	X			X				
Marziali2006										
Span2015						X				
Stewart2006										
Ledderer2013										
Vandenhurk2015						X				
Wittenberg2017		X	X			X				
Bamford2000										
Bowes2003										
Butterworth1995										
Gillies2000										
Innes2005										
Koffman1997										
Pearson1988										
Pickard2001										
Upton2005										
Watkins1997										
Wenger2002										
Ashworth2000										
Ashworth1996										
Hanson1999										
Heenan2000										
Jarrett1999										
Kerr2001										
Lane2003										
Low2004										
Low2005										
McGarry2001										
McLean1991										
Netto1998										
Skilbeck2005										
Ward1997										
Beisecker1996										
Brody1989										
Cotrell1996										
Dziegielewski2000										
Farran2003										
King1995										
Lampley2001										
Liken1995										
MaloneBeach1992										
Martin-Cook2000										
McGrath2000										
Tietelman2004										
Winslow2003										
Wykle1991										
Bedini1996										

Caserta1987										
Hennessy1996										
Lewis1995										
Piercy2004										
Valadez2005										
Worcester1997										
Morgan2002										
Perry2001										
Strang1998										
Strang2000										
Strang2001										
Wuest2001										
Michallet2001										
Ritchie2003										
Strang2002										
Wiles2003										
Gilmour2002										
Leong2001										
King2005										
Kristjanson2004										
Witt2004										
Yamamoto1998										
Young2002										
Ericson2001										
Jansson1998										
Bergs2002										
Berthold1991										
Johansson1991										
Hawranick2007										
Adams2006										
Scharlach2006										
Zepart2007										
Salin2007										
Mcllfatrick2006										
Gerdner2007										
Greenwood2013										
Cohen1998										
Diehl2003										
McKechnie2014				X		X				
Woods2014				X		X				
Sommerland2014				X		X				
Epstein-Lubow2011		1								
Hoppes2012		1								
Marconi2016		X	X	X		X				
Jain2014				X		X				
Kubo2018		X	X	X		X				
Cottingham2018		X	X	X		X				
Bazzano2015				X		X				
Hou2014				X		X				
Minor2006										
Vandenhurk2013										
Lliffe2014		1		X		X				
Verkade2010		1								
Minkman2009		1								

Rothera2008		1								
Boughtwood2011		1								
Burton2005		1								
Dewing2005		1								
Duane2013		1								
McGhee2010		1								
Quinn2013		1								
Blusi2013										
Damianakis2016		X		X		X				
Lichenstein2013										
Marziali2005										
Perron2002										
Torp2013										
Trondsen2014				X		X				
Smith2015				X		X				

For peer review only - http://bmjopen.bmj.com/site/about/guidelines.xhtml

view only

iew only

view only

view only

view only

iew only

view only

iew only

iew only

iew only

view only

view only

iew only

view only

897 AlDaken 201	2367 Hopwood 2	6171 Smith 2014	5049 Sin 2018	3195 Shaw 2009	1459 Dam 2016	5843 Miles 2020	4806 Parkinson 2	5510 Li 2016											
				X															
				X															
				X															
				X															
				X															
		X		X	X			X											
		X		X	X			X											
		X		X															
		X		X															
		X		X															
				X															
				X															
		X		X															
				X															
		X		X															
		X		X	X			X											
		X		X	X			X											
		X		X															
				X															
		X		X															
		X		X															
		X		X	X			X											
				X															
				X															
				X															
				X															
				X															
		X		X															
		X		X															
		X		X															
		X		X	X			X											
		X		X	X			X											
		X		X	X			X											

For peer review only

		X		X															
		X		X															
		X		X															
		X		X															
		X		X	X			X											
1		X		X	X			X											
	1	X		X	X			X											
	1	X		X	X			X											
	1																		
	1	X		X															
		1																	
				X															
		X		X															
		X		X	X		1	X											
				1															
				1															
				1															
				1															
				1															
				1															
				1															
				1															
				1															
				1															
				1															
				1															
				1															
				1															
				1															
				1															
				1															
				1															
				1															
				1															
				1															
				1															
				1															
				1															
				1															
				1															
				1															
				1															
				1															
				1															
				1															
				1															
				1															
				1															
				1															
				1															
				1															
				1															
				1															
				1															

For peer review only

				1															
				1															
				1															
				1															
				1															
				1															
				1															
				1															
				1															
				1															
				1															
				1															
				1															
				1															
				1															
				1															
				1															
				1															
				1															
				1															
				1															
				1															
				1															
				1															
				1															
				1															
				1															
				1															
				1															
				1															
				1															
				1															
				1															
				1															
				1															
					1														
					1														
					1														
		X		X															
		X		X		1													
		X		X		1													
				X															
				X															
		X		X	X		1	X											
		X		X															
X	X	X	X	X	X			X											
X	X	X	X	X	X			X											
		X		X				1											
		X		X				1											
								1											
				X				1											
		X		X															
				X															
				X															

For peer review only

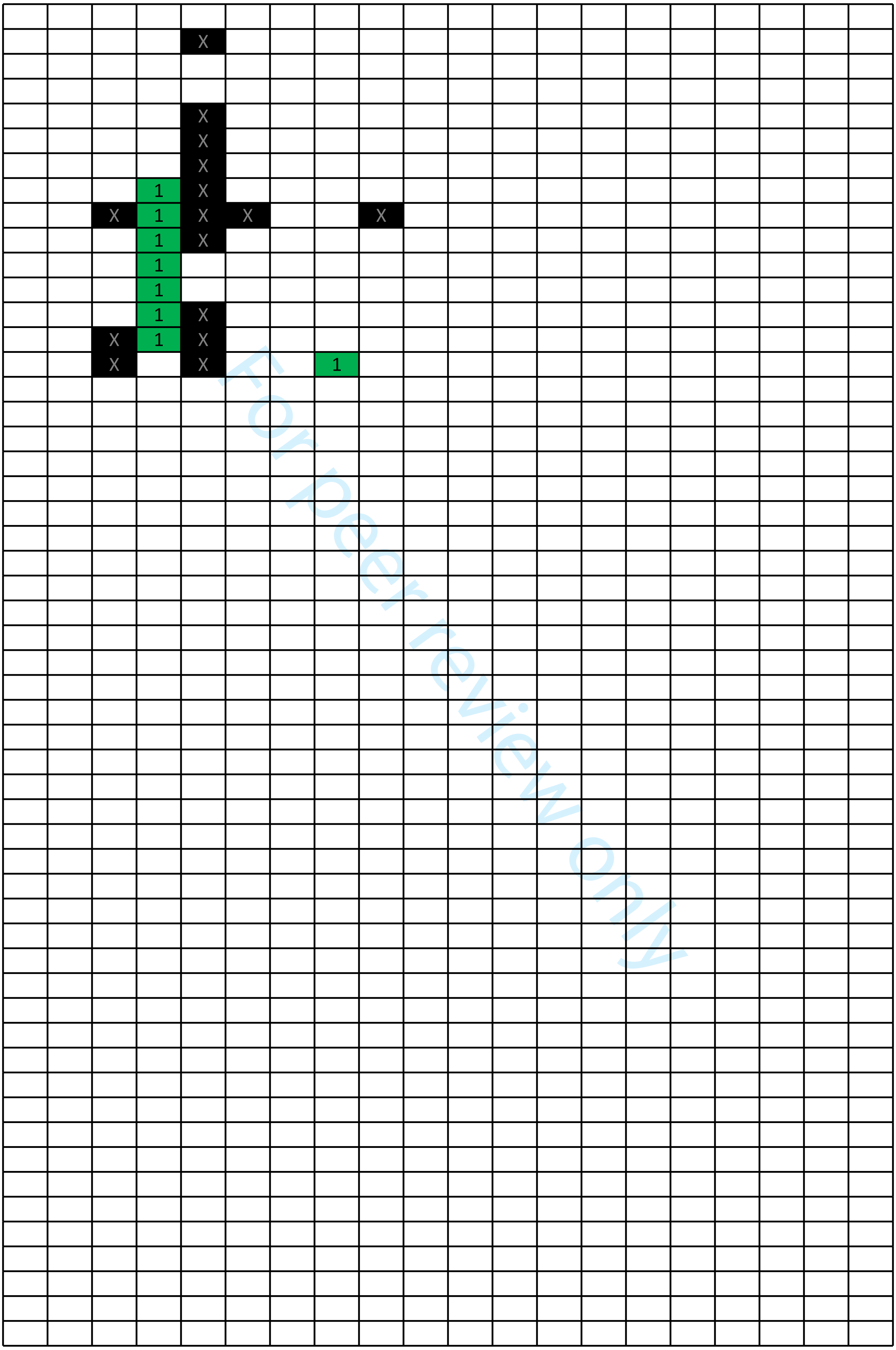

For peer review only

For peer review only

For peer review only

For peer review only

For peer review only

For peer review only

For peer review only

For peer review only

For peer review only

For peer review only

For peer review only

For peer review only

For peer review only

For peer review only

For peer review only

For peer review only

For peer review only

For peer review only

For peer review only

For peer review only

For peer review only

For peer review only

For peer review only

For peer review only

For peer review only

For peer review only

For peer review only

For peer review only

For peer review only

For peer review only

For peer review only

For peer review only

For peer review only

For peer review only

For peer review only

For peer review only

For peer review only

For peer review only

For peer review only

For peer review only

For peer review only

For peer review only

For peer review only

For peer review only

For peer review only

For peer review only

For peer review only

For peer review only

For peer review only

For peer review only

For peer review only

For peer review only

For peer review only

For peer review only

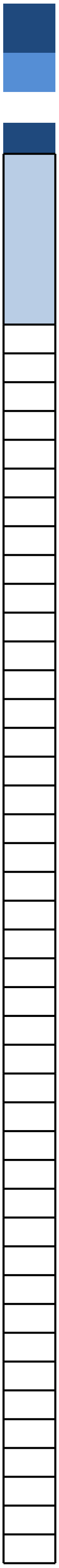

For peer review only

For peer review only

For peer review only

For peer review only

For peer review only

For peer review only

For peer review only

For peer review only

For peer review only

For peer review only

For peer review only

For peer review only

For peer review only

For peer review only

For peer review only

For peer review only

For peer review only

For peer review only

For peer review only

Overall results

Number of columns (number of reviews)	c	18
Number of rows (number of index publications)	r	163
Number of included primary studies (including double counting)	N	160
Covered area	N/(rc)	5.45%
Corrected covered area	(N-r)/(rc-r)	-0.11%
Interpretation of overlap	**Slight overlap**	
Structural Zeros	X	259
Corrected covered area (adjusting by structural zeros)	(N-r)/(rc-r-X)	-0.12%

N° of non-overlapped primary studies	In 1 SR	154
Number of overlapped primary studies	In 2 SRs	3
	In 3 SRs	0
	In 4 SRs	0
	In 5 SRs	0
	In 6 SRs	0
	In 7 SRs	0
	In 8 SRs	0
	In 9 SRs	0
	In 10 SRs	0
	In 11 SRs	0
	In 12 SRs	0
	In 13 SRs	0
	In 14 SRs	0
	In 15 or more SRs	0

G

For peer review only

raphical Representation of Overlap for OVErviews (GRO

	5244 Goeman 20	3366 Bunn 2016	3544 Boots 2014	4285 Maffioletti 2019	4531 Lins 2014	5481 Pritchard 2020	3368 Bourne 2020	3718 DuPreez 2018	807 Irons 2020	897 AlDaken 2018
3366 Bunn 2016	0.0%									
3544 Boots 2014	0.0%	0.0%								
4285 Maffioletti 2019	0.0%	0.0%	0.0%							
4531 Lins 2014	0.0%	0.0%	0.0%	0.0%						
5481 Pritchard 2020	0.0%	0.0%	0.0%	0.0%	0.0%					
3368 Bourne 2020	0.0%	0.0%	0.0%	0.0%	0.0%	0.0%				
3718 DuPreez 2018	0.0%	0.0%	0.0%	6.3%	0.0%	0.0%	0.0%			
807 Irons 2020	0.0%	0.0%	0.0%	0.0%	0.0%	0.0%	16.7%	0.0%		
897 AlDaken 2018	0.0%	0.0%	0.0%	0.0%	0.0%	0.0%	0.0%	0.0%	0.0%	
2367 Hopwood 2018	0.0%	0.0%	0.0%	0.0%	0.0%	0.0%	0.0%	0.0%	0.0%	0.0%
6171 Smith 2014	0.0%	0.0%	0.0%	0.0%	0.0%	0.0%	0.0%	0.0%	0.0%	0.0%
5049 Sin 2018	0.0%	0.0%	0.0%	0.0%	0.0%	0.0%	0.0%	0.0%	0.0%	0.0%
3195 Shaw 2009	0.0%	0.0%	0.0%	0.0%	0.0%	0.0%	0.0%	0.0%	0.0%	0.0%
1459 Dam 2016	0.0%	0.0%	0.0%	0.0%	0.0%	0.0%	0.0%	0.0%	0.0%	0.0%
5843 Miles 2020	0.0%	0.0%	0.0%	0.0%	0.0%	0.0%	0.0%	0.0%	0.0%	0.0%
4806 Parkinson 2019	0.0%	0.0%	0.0%	0.0%	0.0%	0.0%	0.0%	0.0%	0.0%	0.0%
5510 Li 2016	0.0%	0.0%	0.0%	0.0%	0.0%	0.0%	0.0%	0.0%	0.0%	0.0%

**153**
151
1
0
1

For peer review only

For peer review only

OVE)

=	**Total nodes (pairs of reviews)**
=	Slight overlap (<5%)
=	Moderate overlap (5% to <10%)
=	High overlap (10% to <15%)
=	Very High overlap (≥15%)

2367 Hopwood 2018	6171 Smith 2014	5049 Sin 2018	3195 Shaw 2009	1459 Dam 2016	5843 Miles 2020	4806 Parkinson 2019
0.0%						
0.0%	0.0%					
0.0%	0.0%	0.0%				
0.0%	0.0%	0.0%	0.0%			
0.0%	0.0%	0.0%	0.0%	0.0%		
0.0%	0.0%	0.0%	0.0%	0.0%	0.0%	
0.0%	0.0%	0.0%	0.0%	0.0%	0.0%	0.0%

For peer review only

For peer review only

For peer review only

For peer review only

For peer review only

For peer review only

For peer review only

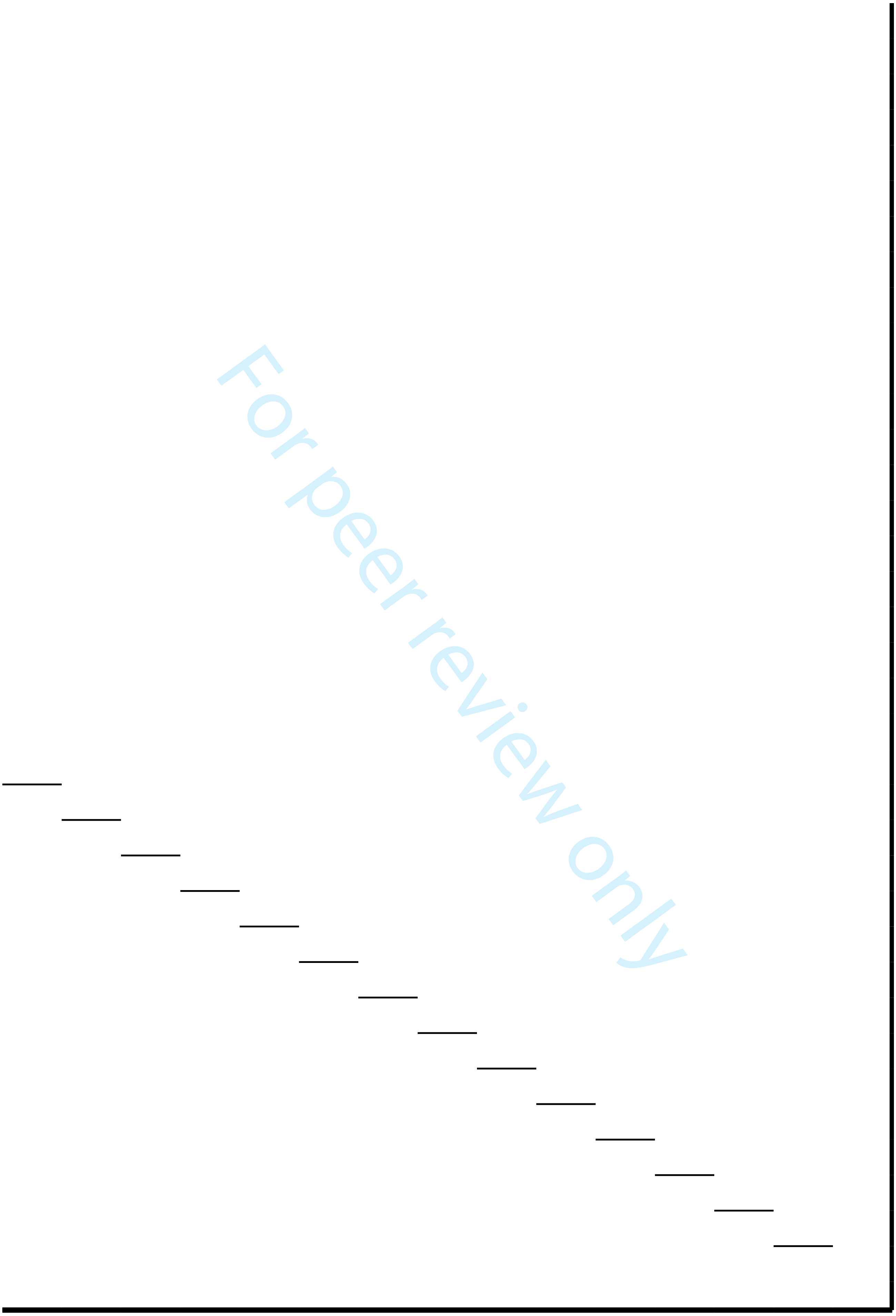

For peer review only

Graphical Representation of Overlap for OVErviews (G

	5244 Goem	3366 Bunn 2016	3544 Boots 2014	4285 Maffioletti 2019	4531 Lins 2014	5481 Pritchard 2020	3368 Bourne 2020	3718 DuPreez 2018
3366 Bunn 2016	0.0%							
3544 Boots 2014	0.0%	0.0%						
4285 Maffioletti 2019	0.0%	0.0%	0.0%					
4531 Lins 2014	0.0%	0.0%	0.0%	0.0%				
5481 Pritchard 2020	0.0%	0.0%	0.0%	0.0%	0.0%			
3368 Bourne 2020	0.0%	0.0%	0.0%	0.0%	0.0%	0.0%		
3718 DuPreez 2018	0.0%	0.0%	0.0%	6.3%	0.0%	0.0%	0.0%	
807 Irons 2020	0.0%	0.0%	0.0%	0.0%	0.0%	0.0%	16.7%	0.0%
897 AlDaken 2018	0.0%	0.0%	0.0%	0.0%	0.0%	0.0%	0.0%	0.0%
2367 Hopwood 2018	0.0%	0.0%	0.0%	0.0%	0.0%	0.0%	0.0%	0.0%
6171 Smith 2014	0.0%	0.0%	0.0%	0.0%	0.0%	0.0%	0.0%	0.0%
5049 Sin 2018	0.0%	0.0%	0.0%	0.0%	0.0%	0.0%	0.0%	0.0%
3195 Shaw 2009	0.0%	0.0%	0.0%	0.0%	0.0%	0.0%	0.0%	0.0%
1459 Dam 2016	0.0%	0.0%	0.0%	0.0%	0.0%	0.0%	0.0%	0.0%
5843 Miles 2020	0.0%	0.0%	0.0%	0.0%	0.0%	0.0%	0.0%	0.0%
4806 Parkinson 2019	0.0%	0.0%	0.0%	0.0%	0.0%	0.0%	0.0%	0.0%
5510 Li 2016	0.0%	0.0%	0.0%	0.0%	0.0%	0.0%	0.0%	0.0%

For peer review only

For peer review only

ROOVE) - Considering Structural Missingness

**153**	=	**Total nodes (pairs of reviews)**
151	=	Slight overlap (<5%)
1	=	Moderate overlap (5% to <10%)
0	=	High overlap (10% to <15%)
1	=	Very High overlap (≥15%)

807 Irons 2020	897 AlDaken 2018	2367 Hopwood 2018	6171 Smith 2014	5049 Sin 2018	3195 Shaw 2009	1459 Dam 2016	5843 Miles 2020	4806 Parkinson 2019
0.0%								
0.0%	0.0%							
0.0%	0.0%	0.0%						
0.0%	0.0%	0.0%	0.0%					
0.0%	0.0%	0.0%	0.0%	0.0%				
0.0%	0.0%	0.0%	0.0%	0.0%	0.0%			
0.0%	0.0%	0.0%	0.0%	0.0%	0.0%	0.0%		
0.0%	0.0%	0.0%	0.0%	0.0%	0.0%	0.0%	0.0%	
0.0%	0.0%	0.0%	0.0%	0.0%	0.0%	0.0%	0.0%	0.0%

For peer review only

For peer review only

For peer review only

For peer review only

For peer review only

For peer review only

For peer review only

For peer review only

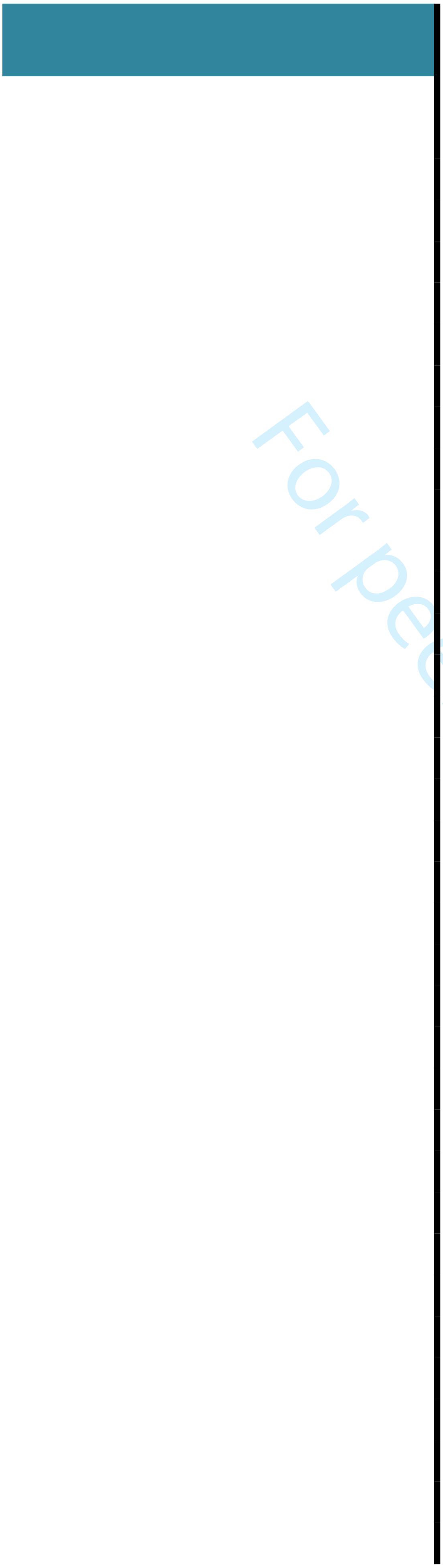

For peer review only

For peer review only

Overall results

Number of columns (number of reviews)	c	18
Number of rows (number of index publications)	r	163
Number of included primary studies (including double counting)	N	160
Covered area	N/(rc)	5.45%
Corrected covered area	(N-r)/(rc-r)	-0.11%
Interpretation of overlap	**Slight overlap**	
Structural Zeros	X	259
Corrected covered area (adjusting by structural zeros)	(N-r)/(rc-r-X)	-0.12%

N° of non-overlapped primary studies	In 1 SR	154
Number of overlapped primary studies	In 2 SRs	3
	In 3 SRs	0
	In 4 SRs	0
	In 5 SRs	0
	In 6 SRs	0
	In 7 SRs	0
	In 8 SRs	0
	In 9 SRs	0
	In 10 SRs	0
	In 11 SRs	0
	In 12 SRs	0
	In 13 SRs	0
	In 14 SRs	0
	In 15 or more SRs	0

G

For peer review only

Graphical Representation of Overlap for OVErviews (GRO

	5244 Goeman 20	3366 Bunn 2016	3544 Boots 2014	4285 Maffioletti 2019	4531 Lins 2014	5481 Pritchard 2020	3368 Bourne 2020	3718 DuPreez 2018	807 Irons 2020	897 AlDaken 2018
3366 Bunn 2016	0.0%									
3544 Boots 2014	0.0%	0.0%								
4285 Maffioletti 2019	0.0%	0.0%	0.0%							
4531 Lins 2014	0.0%	0.0%	0.0%	0.0%						
5481 Pritchard 2020	0.0%	0.0%	0.0%	0.0%	0.0%					
3368 Bourne 2020	0.0%	0.0%	0.0%	0.0%	0.0%	0.0%				
3718 DuPreez 2018	0.0%	0.0%	0.0%	6.3%	0.0%	0.0%	0.0%			
807 Irons 2020	0.0%	0.0%	0.0%	0.0%	0.0%	0.0%	16.7%	0.0%		
897 AlDaken 2018	0.0%	0.0%	0.0%	0.0%	0.0%	0.0%	0.0%	0.0%	0.0%	
2367 Hopwood 2018	0.0%	0.0%	0.0%	0.0%	0.0%	0.0%	0.0%	0.0%	0.0%	0.0%
6171 Smith 2014	0.0%	0.0%	0.0%	0.0%	0.0%	0.0%	0.0%	0.0%	0.0%	0.0%
5049 Sin 2018	0.0%	0.0%	0.0%	0.0%	0.0%	0.0%	0.0%	0.0%	0.0%	0.0%
3195 Shaw 2009	0.0%	0.0%	0.0%	0.0%	0.0%	0.0%	0.0%	0.0%	0.0%	0.0%
1459 Dam 2016	0.0%	0.0%	0.0%	0.0%	0.0%	0.0%	0.0%	0.0%	0.0%	0.0%
5843 Miles 2020	0.0%	0.0%	0.0%	0.0%	0.0%	0.0%	0.0%	0.0%	0.0%	0.0%
4806 Parkinson 2019	0.0%	0.0%	0.0%	0.0%	0.0%	0.0%	0.0%	0.0%	0.0%	0.0%
5510 Li 2016	0.0%	0.0%	0.0%	0.0%	0.0%	0.0%	0.0%	0.0%	0.0%	0.0%

**153**
151
1
0
1

For peer review only

For peer review only

OVE)

=	Total nodes (pairs of reviews)
=	Slight overlap (<5%)
=	Moderate overlap (5% to <10%)
=	High overlap (10% to <15%)
=	Very High overlap (>15%)

2367 Hopwood 2018	6171 Smith 2014	5049 Sin 2018	3195 Shaw 2009	1459 Dam 2016	5843 Miles 2020	4806 Parkinson 2019
0.0%						
0.0%	0.0%					
0.0%	0.0%	0.0%				
0.0%	0.0%	0.0%	0.0%			
0.0%	0.0%	0.0%	0.0%	0.0%		
0.0%	0.0%	0.0%	0.0%	0.0%	0.0%	
0.0%	0.0%	0.0%	0.0%	0.0%	0.0%	0.0%

For peer review only

For peer review only

[redacted]

For peer review only

For peer review only

[redacted]

For peer review only

For peer review only

For peer review only

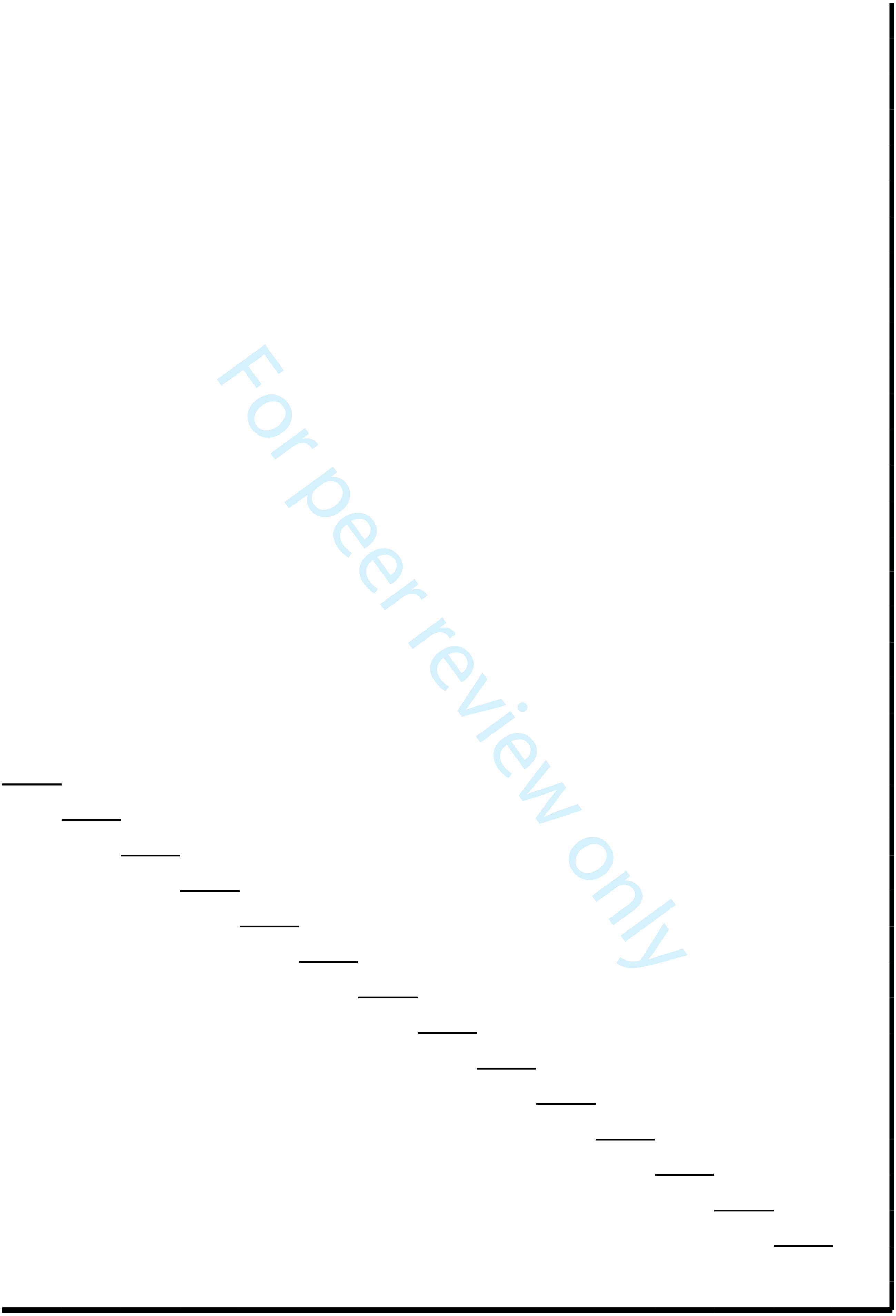

For peer review only

Graphical Representation of Overlap for OVErviews (G

	5244 Goem	3366 Bunn 2016	3544 Boots 2014	4285 Maffioletti 2019	4531 Lins 2014	5481 Pritchard 2020	3368 Bourne 2020	3718 DuPreez 2018
3366 Bunn 2016	0.0%							
3544 Boots 2014	0.0%	0.0%						
4285 Maffioletti 2019	0.0%	0.0%	0.0%					
4531 Lins 2014	0.0%	0.0%	0.0%	0.0%				
5481 Pritchard 2020	0.0%	0.0%	0.0%	0.0%	0.0%			
3368 Bourne 2020	0.0%	0.0%	0.0%	0.0%	0.0%	0.0%		
3718 DuPreez 2018	0.0%	0.0%	0.0%	6.3%	0.0%	0.0%	0.0%	
807 Irons 2020	0.0%	0.0%	0.0%	0.0%	0.0%	0.0%	16.7%	0.0%
897 AlDaken 2018	0.0%	0.0%	0.0%	0.0%	0.0%	0.0%	0.0%	0.0%
2367 Hopwood 2018	0.0%	0.0%	0.0%	0.0%	0.0%	0.0%	0.0%	0.0%
6171 Smith 2014	0.0%	0.0%	0.0%	0.0%	0.0%	0.0%	0.0%	0.0%
5049 Sin 2018	0.0%	0.0%	0.0%	0.0%	0.0%	0.0%	0.0%	0.0%
3195 Shaw 2009	0.0%	0.0%	0.0%	0.0%	0.0%	0.0%	0.0%	0.0%
1459 Dam 2016	0.0%	0.0%	0.0%	0.0%	0.0%	0.0%	0.0%	0.0%
5843 Miles 2020	0.0%	0.0%	0.0%	0.0%	0.0%	0.0%	0.0%	0.0%
4806 Parkinson 2019	0.0%	0.0%	0.0%	0.0%	0.0%	0.0%	0.0%	0.0%
5510 Li 2016	0.0%	0.0%	0.0%	0.0%	0.0%	0.0%	0.0%	0.0%

For peer review only

For peer review only

;ROOVE) - Considering Structural Missingness

153	=	**Total nodes (pairs of reviews)**
151	=	Slight overlap (<5%)
1	=	Moderate overlap (5% to <10%)
0	=	High overlap (10% to <15%)
1	=	Very High overlap (≥15%)

807 Irons 2020	897 AlDaken 2018	2367 Hopwood 2018	6171 Smith 2014	5049 Sin 2018	3195 Shaw 2009	1459 Dam 2016	5843 Miles 2020	4806 Parkinson 2019
0.0%								
0.0%	0.0%							
0.0%	0.0%	0.0%						
0.0%	0.0%	0.0%	0.0%					
0.0%	0.0%	0.0%	0.0%	0.0%				
0.0%	0.0%	0.0%	0.0%	0.0%	0.0%			
0.0%	0.0%	0.0%	0.0%	0.0%	0.0%	0.0%		
0.0%	0.0%	0.0%	0.0%	0.0%	0.0%	0.0%	0.0%	
0.0%	0.0%	0.0%	0.0%	0.0%	0.0%	0.0%	0.0%	0.0%

For peer review only

For peer review only - http://bmjopen.bmj.com/site/about/guidelines.xhtml

For peer review only

[redacted]

For peer review only

For peer review only

[redacted]

For peer review only

For peer review only

[redacted]

For peer review only

For peer review only

For peer review only

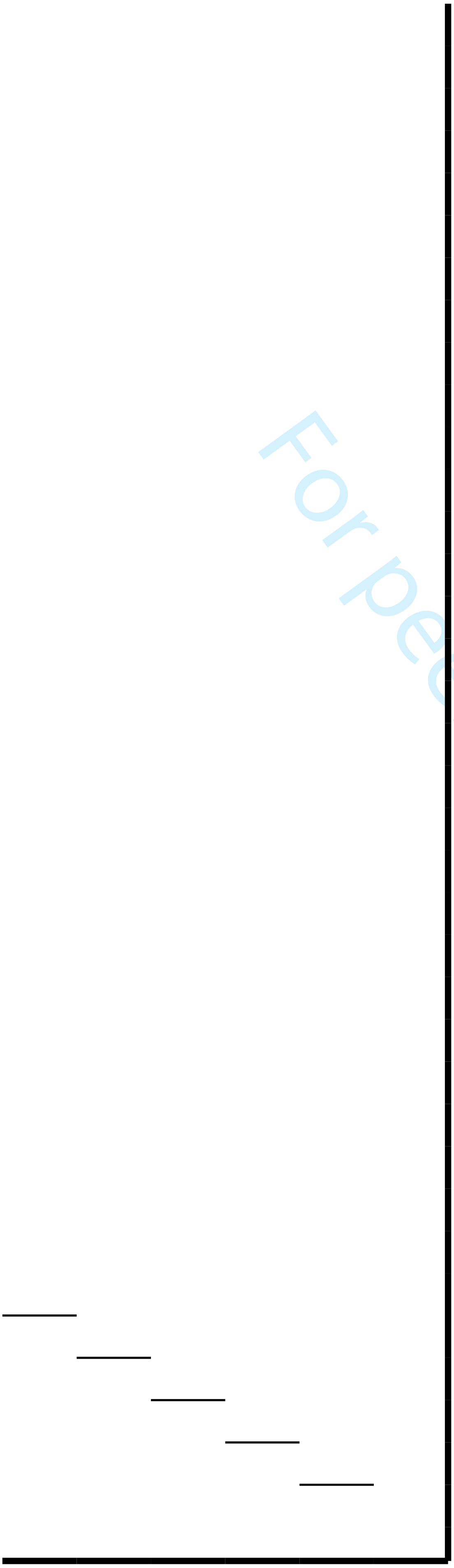

For peer review only
